# The perceptual geometry and chromaticity of near-death experience spaces

**DOI:** 10.3389/fpsyg.2026.1760389

**Published:** 2026-10-15

**Authors:** France Lerner, Paulo Cezar Rocha dos Santos, Guillaume Tahar

**Affiliations:** 1 Contemplative Neuroscience and Neurophenomenology Lab, Department of Education, University of Haifa, Haifa, Israel; 2 Department/Institute for Research and Education (IDOR), Rio de Janeiro, RJ, Brazil; 3 Guangdong Technion - Israel Institute of Technology (GTIIT), Shantou, Guangdong, China

**Keywords:** bodily self-consciousness, NDEs, OBEs, self-location, visual-field phenomenology

## Abstract

Near-Death Experiences (NDEs) challenge conventional research methods, as they are often described as exceeding the limits of verbal expression. While most studies rely on narrative accounts, little attention has been given to graphic representations as a complementary mode of investigation. This study employs a hybrid approach combining a semi-structured digital questionnaire with a graphic reconstruction task. Participants first segmented their Out-of-Body Experience (OBE) and NDE into distinct experiential sequences by assigning chronological numbers to each phase. This sequencing enabled the identification of what we term the experiential NDE Core, defined here as the NDE sequence or sequences distinguished from the Dissociative and Associative OBE phases, thereby facilitating comparisons across participants. The graphic reconstructions identified recurrent Visual-Field boundary configurations, including conical forms, elliptic arcs with variable Visual-Field Extents (VFE), and ellipsoidal configurations. Exploratory analyses indicated variation in chromatic perceptual qualities across these configurations, although evidence for differences among configurations was not uniform across statistical analyses. The reported emergence of Visual Mental Imagery also appeared to vary across Visual-Field configurations. This dual-method design was intended to capture experiential features of OBEs and NDEs that may not be conveyed through verbal reports alone. Notably, OBEs have rarely been examined in terms of their independence from, or conjunction with, NDEs. In the present study, their temporal placement was defined operationally by the sequencing protocol to permit isolation of the NDE Core.

## 1 Introduction

NDEs are profound, often transformative experiences that have been reported in association with a range of circumstances, including cardiac arrest (Parnia et al., 2001), respiratory failure, coma, brain disorders (Facco and Agrillo, 2012b), post-traumatic coma (Hou et al., 2013), syncope, states of emotional shock (Greyson, 2014), and other critical or subjectively threatening events (Owens et al., 1990). These experiences are frequently characterized by vivid multisensory mental imagery, often perceived as subjectively real (Thonnard et al., 2013; Palmieri et al., 2014; Martial et al., 2019b). Within the broader field of mental imagery, Visual Mental Imagery refers to internally generated visual representations of people, places, objects, colors, shapes, letters, or spatial configurations in the absence of corresponding external visual input (Spagna et al., 2024).

Recent studies estimate that 4%–8% of the general population has had an NDE, with a frequency up to 10% in a cross-cultural sample of 1,034 individuals across 35 countries (Kondziella et al., 2019). Incidence is higher among clinical populations, with 6%–23% of cardiac-arrest survivors reporting such experiences (Van Lommel et al., 2001; Schwaninger et al., 2002; Klemenc-Ketis, 2013). Despite their prevalence, NDEs remain difficult to investigate due to ineffability often linked to their emotional intensity (Thonnard et al., 2013). Moreover, NDE memories have been reported to resemble real memories phenomenologically (Moore and Greyson, 2017), electrophysiological studies have identified temporal theta activity during NDE-memory recall, consistent with episodic memory processes (Palmieri et al., 2014). Their vivid perceptual and affective content may exceed the expressive capacity of verbal reports, supporting the use of alternative methodologies to access and represent spatial and visual structure.

Among commonly reported features, OBEs hold a distinctive place. They are characterized by a sense of disembodiment, in which the self is experienced as located outside the physical body, and often the physical body itself is perceived from an extracorporeal first-person visuospatial perspective. As Blanke (2012) defines it, “self-location, that is, the experience of where I am in space” is typically at the location of the body and closely tied to the first-person perspective; in OBEs, this Self-Location is transposed to an extracorporeal space. Neurophysiological evidence from focal electrical stimulation of the right angular gyrus suggests that such experiences may result from a breakdown in the brain's integration of complex somatosensory and vestibular signals, leading to altered whole-body perception and displacement (Blanke and Arzy, 2005; Blanke et al., 2002).

In the general population, 10%–20% report at least one OBE (Blackmore, 1984; Murray and Fox, 2005). In the context of NDEs, 45%–80% report OBEs during the episode (Greyson, 2014; Van Lommel et al., 2001; Parnia et al., 2014), typically early in the NDE and often coinciding with observing one's physical body from an elevated, external viewpoint. These frequencies suggest OBEs may constitute a recurrent component of NDEs phenomenological structure. Although phenomenological studies have identified recurring features such as tunnel perception, encounters with deceased individuals, and out-of-body perspectives (Zaleski, 1987; Blackmore, 1993; Belanti et al., 2008; Greyson et al., 2009; Greyson, 2015), less attention has been directed to the visuo-spatial structure of these experiences and to how individuals visually perceive and kinesthetically experience their Self-Motion within them.

The study of NDEs progressed from early philosophical and theological inquiry to empirical investigation within the human and medical sciences. The first systematic collection was published by Heim (1892), and the term “near-death experience” was introduced by Egger (1896) in *The Self of the Dying*. Early explorations by parapsychologists, including Barrett (1926/1986) in *Death-Bed Visions*, were met with skepticism, delaying their integration into mainstream scientific research. Interest reemerged in the late 20th century through foundational contributions in psychology and psychiatry (Moody, 1978; Ring, 1980; Greyson and Flynn, 1984; Sabom, 1982). This renewed empirical interest subsequently expanded into the neurosciences, where explanatory models have been developed in neurology (Vanhaudenhuyse et al., 2009; Blanke et al., 2016), neurochemistry (Martial et al., 2019a), neurophysiology (Morse et al., 1989), and neurobiology (Saavedra-Aguilar and Gómez-Jeria, 1989; Britton and Bootzin, 2004).

Historically, both Western and non-Western artistic depictions have represented altered states, including NDEs (León, 2013). Iconographic parallels appear in Bosch's *Visions of the Hereafter* (*Ascent of the Blessed*, 1500–1504), Fuseli's *The Nightmare* (1781), and Doré's *Vision of the Empyrean* (1868). Modern cinema reiterates NDE motifs in *The Wizard of Oz* (Fleming, 1939), *Flatliners* (Schumacher, 1990), and *Brainstorm* (Trumbull, 1983). For OBEs, both Western artistic traditions (e.g., Schiavonetti after William Blake's *The Soul Hovering Over the Body*, 1805–08) and non-Western forms such as Aboriginal Australian painting (e.g., Western Desert works by Clifford Possum Tjapaltjarri) depict elevated, extracorporeal viewpoints.

Research on NDE phenomenology has used several complementary approaches. Alongside written reports, thematic analyses, and artistic representations (Kennedy, 1997; Rominger, 2004; Tang, 2019), quantitative psychometric studies have examined the organization of NDE phenomenology using Item Response Theory. Applying Rasch modeling to the Greyson Near-Death Experience Scale, Lange et al. (2004) showed that the scale items form a unidimensional probabilistic hierarchy along a latent dimension of NDE intensity. This hierarchy describes the probability with which different phenomenological features are endorsed across experiencers rather than the chronological order in which they occur within an individual experience. More recently, Pehlivanova et al. (2026) replicated this Rasch item hierarchy in an independent sample.

The present study complements this psychometric framework by examining dimensions of NDE phenomenology that are not represented by the probabilistic organization of scale items. Specifically, the graphic reconstruction procedure investigates the spatial organization of the experienced Visual Field, the position of Self-Location, the dynamics of Self-Motion, and their chronological organization across reconstructed experiential phases. Rather than proposing an alternative organization of canonical NDE features, this approach provides a complementary visuo-spatial and sequential characterization of NDE phenomenology. To examine the relationship between these complementary characterizations empirically, we additionally tested whether the presence of each Visual Field category was associated with NDE intensity as measured by the Greyson NDE Scale and NDE-C total scores.

Our study builds upon previous research, including earlier investigations into the spatial organization of OBEs and NDEs (Lerner, 2016; Lerner et al., 2021), by introducing structured sequencing of visuo-spatial configurations and systematically mapping Self-Location, Self-Motion, and the spatial localization of Visual Mental Imagery within these configurations. Through this approach, we aim to characterize recurrent Visual-Field boundary configurations in OBEs and NDEs and to examine how the emergence of Visual Mental Imagery, defined here as internally experienced visual imagery perceived within the visual field during the NDE, varies across different Visual-Field configurations.

In the present study, Visual Mental Imagery does not imply that the reported experience was exclusively visual. Rather, the term is used to designate the visual-spatial component of a broader multisensory experience. This usage is consistent with contemporary mental imagery research, in which visual mental imagery is understood as a heterogeneous construct comprising multiple imagery domains, including visuospatial imagery, faces, colors, shapes, and letters (Spagna et al., 2024). Spagna et al. (2024) further propose that these imagery processes involve both domain-general and domain-specific mechanisms supported by partially overlapping neural networks rather than a single isolated imagery system. Accordingly, the present study focuses specifically on the visual-field structure, boundary geometry, viewpoint organization, Self-Location, and Self-Motion of NDE imagery, while acknowledging that these visual-spatial configurations may co-occur with auditory, tactile, vestibular, proprioceptive, somatic, and affective dimensions.

Previous psychophysical studies of mental imagery have shown that imagined visual fields possess measurable spatial boundaries and directional asymmetries, and that their extent varies as a function of perceptual resolution (Finke and Kurtzman, 1981). We interpret the Visual Fields represented in NDErs graphic representations as instances of what Haun and Tononi (2025) describe as the intrinsic structure of visual experience: “It is not necessarily the representation of the space of the outside world (commonly referred to as visual space). The spatial structure of the visual field is largely invariant to the organization of the world it mediates.”

In our study, each NDEr's boundary-outline graphical representation represents the intrinsic spatial structure of the Visual Field as experienced during the NDE. Consistent with Gibson's (2014) ecological approach to visual perception, in which visual experience is understood as spatially structured relative to the perceiver and the environment, the Visual Field is understood here as the spatial organization and extent of visual awareness during the experience. Within this framework, Visual Field Extent (VFE) refers to the perceived angular or spatial extent of the Visual Field boundary relative to the participant's marked Self-Location.

The graphic representations therefore not only depict the boundaries of the experienced Visual Field, but also map the NDErs' first-person spatial dynamics within it through the spatial positioning of Self-Locations and the directional trajectories of Self-Motion. By analyzing the marked Self-Locations and Self-Motion trajectories relative to each Visual Field boundary and its Visual Field Extent, we examined how these factors influenced the perceived curvature and spatial organization of the Visual Field. Ultimately, we aim to establish a systematic framework for analyzing the spatial organization of OBEs and NDEs, contributing to a more precise characterization of their experiential structure and content.

## 2 Materials and methods

### 2.1 Ethical approval

This study was approved by the Ethics Committee of the Unit for Interdisciplinary Studies, Bar-Ilan University, Ramat Gan (IRB No. ISU202208001). All participants provided informed consent, and the study adhered to ethical standards protecting participants' rights and privacy.

### 2.2 Participants

Eligibility required fluency in English, age ≥18, and absence of mental disability or guardianship. Participants were recruited online through a research participation call distributed via the International Association for Near-Death Studies (IANDS, USA) website under the study title “The sense of space, feelings of motion, emotions, and sensations in NDEs” (IANDS research participation page).

Eligibility for inclusion in the present study as a Near-Death Experiencer (NDEr) was determined through completion of a preliminary questionnaire including two validated psychometric instruments: the Greyson NDE Scale (Greyson, 1983) and the NDE Content Scale (NDE-C; Martial et al., 2020) (Supplementary material, *Preliminary Questionnaire*). The Greyson Scale comprises 16 items scored from 0 to 2, with a total score of ≥7 used to identify an NDE. The NDE-C comprises 20 items scored from 0 to 4, with a validated threshold of ≥27. Only participants meeting both thresholds (Greyson ≥7 and NDE-C ≥27) were eligible for inclusion in the main study.

The preliminary questionnaire also asked participants to report the circumstances associated with their experience. Participants who had been hospitalized were asked to report the diagnosis or etiology communicated to them by medical personnel, whereas those who had not been hospitalized were asked to describe the physiological circumstances and psychological context of the experience. These data were based on retrospective self-report and were not independently verified from medical records.

All 101 volunteers completed the preliminary questionnaire (Supplementary material, *Preliminary Questionnaire Answers*). Of these, 97 met both validated psychometric cutoffs and were invited to participate in the second phase of the study. One participant met the threshold of the Greyson NDE Scale (≥7) but scored below the NDE-C cutoff (< 27). The remaining three participants failed to meet either psychometric threshold, scoring below both the Greyson NDE Scale cutoff and the NDE-C cutoff, and were therefore not eligible for inclusion in the main study.

Fifty participants subsequently completed the main questionnaire and graphic reconstruction procedure. The second phase required approximately 1.5–3 h of synchronous participation, which may have contributed to the lower completion rate. Participants who did not complete the second phase did not schedule a session, and their individual reasons for not doing so were not recorded. Participation was voluntary, and participants could withdraw from the study at any time without providing a reason by terminating the session and closing their browser.

### 2.3 Methodology

Our study characterized NDEs and OBEs perceptual structures as experienced (Husserl, 1982). This approach emphasizes the perspectival, horizon-structured, and embodied nature of visual experience (Gibson, 2014; Thompson, 2010; Madary, 2016). First-person reports were analyzed to identify recurrent NDE Visual Field types and perceptual features. These categories were then operationalized as quantitative variables, following principles of experimental phenomenology by merging qualitative descriptions with systematic quantification of perceptual properties (Albertazzi et al., 2011; Albertazzi, 2013). This integration parallels experimental neurophenomenology, in which first-person accounts are treated as structured data suitable for coding and statistical analysis (Thompson, 2010), thus ensuring reproducibility and comparability.

### 2.4 Questionnaire design and administration

Before the live session, participants received an instructional email outlining the procedure and were asked to print a reference sheet defining the key terms and graphic symbols used during the reconstruction tasks. This reference document contained the catalog of NDE pictograms, the lexicon, and the legend symbols used in the reconstruction procedure (Supplementary material, *Catalog*), together with illustrative examples of Dissociative OBE (Supplementary material, *Examples*, p. 1), NDE space types A–C (pp. 2–4), and Associative OBE (p. 5).

The catalog of NDE pictograms was developed from an earlier phenomenological reconstruction methodology described in Lerner's (2016) doctoral thesis (pp. 45–47). In that study, participants produced unconstrained graphic reconstructions without predefined geometric categories, pictorial templates, or reference images. The original protocol used “blind drawing," meaning that participants drew without visually monitoring the marks they were making. This procedure was intended to reduce deliberate aesthetic correction, such as correcting, regularizing, or beautifying the drawing while it was being produced, thereby encouraging a spontaneous representation of the remembered spatial experience. The resulting corpus of unconstrained graphic reconstructions constituted the primary phenomenological material from which recurrent Visual Field boundary organizations were subsequently identified and abstracted into the simplified pictogram catalog used in the present study. Accordingly, the catalog served as a standardized reconstruction aid and descriptive framework for systematic comparison across participants.

In the present protocol, participants first selected the pictogram that most closely approximated the remembered organization of their Visual Field. This initial selection served only as a starting point, after which participants redrew and modified the configuration according to their own experience by adjusting its orientation, curvature, opening angle, proportions, and Visual Field Extent. They could also modify the position of the Self-Location, represent Self-Motion trajectories, and add or reposition internal visual elements whenever necessary to reconstruct the remembered scene. If none of the proposed pictograms adequately represented their experience, participants selected “Other Space” and produced an entirely unconstrained freehand reconstruction. Consequently, the analyses were based on each participant's final reconstruction rather than on the initial catalog pictogram.

Within this standardized reconstruction framework, participants completed a semi-structured, researcher-assisted digital questionnaire during synchronous remote sessions conducted via Google Meet. The Google Meet sessions were not audio- or video-recorded; the retained study records consisted of participants' questionnaire responses and submitted graphic reconstructions. The questionnaire and graphic reconstruction tasks were completed concurrently, with the researcher providing guidance and clarification when needed. The questionnaire, implemented in Google Forms, consisted primarily of closed-ended checkbox-style items to ensure response consistency and support quantitative analysis, supplemented by open-ended questions capturing qualitative details (Supplementary material, *Main Questionnaire*). The main questionnaire was organized into repeated sets corresponding to the number of experiential sequences reported by each participant. Because the questionnaire structure and items were identical across all experiential sequences, only the first set is presented in the Supplementary material as a representative example. Additional sets (parts 2–10) contained the same questions and organizational structure and were administered only when participants reported multiple experiential sequences.

Session duration ranged from approximately 1.5–3 h, depending on the complexity of the participant's OBE and/or NDE. The researcher-assisted sessions were intended to guide participants through the procedural requirements of the reconstruction task and to clarify the operational meaning of questionnaire items, graphical symbols, and spatial reconstruction instructions. Participants were not informed of the study's geometric hypotheses or expected Visual Field categories prior to the reconstruction process. Importantly, the researcher did not select, recommend, rank, or adjudicate between geometric pictograms on behalf of participants. Participants themselves selected the pictogram that they considered to most closely approximate the remembered Visual Field boundary. If a participant considered the available pictogram only an approximation, the participant modified its geometry freehand to more accurately represent the remembered experience. Researcher clarification was restricted to procedural aspects of the task, including the use of graphical symbols, the sequencing of experiential parts, and the conventions for representing Self-Location and Self-Motion. Accordingly, the broad geometric family used in the analysis derived from the participant's own pictogram selection, while subsequent freehand modifications refined the reconstructed geometry without introducing a second, *post hoc* researcher-assigned classification of the final drawing.

### 2.5 Instructions for recalling and structuring OBE and NDE

Participants were first guided through the lexicon to ensure a shared understanding of all terminology and graphical conventions used during the reconstruction procedure. They were then encouraged to take as much time as needed to recall their OBE and/or NDE and were invited to close their eyes if this facilitated memory retrieval.

After this clarification phase, participants were asked to divide their experience into sequential experiential parts, which could include OBE phases, NDE Core phases, or both. To facilitate segmentation, metaphorical analogies such as scenes in a film, acts in a play, chapters in a book, or movements in music were proposed, and participants selected whichever best matched the perceived structure of their experience. In this study, the term “NDE Core” refers to the sequence, or set of sequences, phenomenologically identified as constituting the NDE after the Dissociative and Associative OBE phases had been distinguished during the chronological reconstruction. The term is therefore used operationally to designate the temporally organized NDE component of the reconstructed experience. This usage differs from the concept of a “core” near-death experience examined by Lange et al. (2004). In their psychometric framework, the Greyson NDE Scale items form a probabilistic hierarchy along a unidimensional latent dimension of NDE phenomenology whose meaning remained invariant across participant characteristics and variations in NDE intensity. In Lange et al.'s framework, the term does not refer to a fixed chronological sequence or to a temporally delimited segment of an individual experience.

Each participant first identified whether the experience contained an OBE component. When present, the OBE was classified as Dissociative, Associative, or both. A Dissociative OBE involved adopting a parasomatic viewpoint and perceiving the physical body and/or surrounding physical space while experiencing a felt separation from it. In contrast, an Associative OBE involved the same parasomatic viewpoint and perception of the physical body and/or physical space, but was accompanied by a felt re-embodiment into it. Each OBE type counted as one experiential part; if both occurred, they were recorded separately, yielding two distinct OBE parts.

Participants then chronologically organized all experiential parts, with the Dissociative OBE preceding the NDE Core sequence and the Associative OBE following it. One printed reconstruction page was assigned to each experiential part, such that the total number of printed pages equalled the total number of experiential parts, summarized by the formula: “X parts = Y OBE parts + Z NDE parts.” Each page therefore represented a single temporally ordered experiential sequence.

Finally, participants numbered all graphic reconstruction pages in chronological order and reviewed the complete sequence, including OBE phases and NDE Core parts, before proceeding to the next stage.

### 2.6 Graphic reconstruction and drawing digitization

For the graphic representation tasks of OBEs and NDEs, participants used a blue pen (1.00 mm tip).

For the OBE reconstructions, participants first reviewed illustrative examples (Supplementary material, *Examples*, pp. 1, 5), then drew the outline boundaries of the physical space in which the OBE occurred and annotated it with standardized symbols: a circle for the physical body, a cross for the Self-Location, an arrow for the direction of Self-Motion, and squares for other beings (e.g., medical staff or animals).

For the NDE reconstructions, participants first reviewed illustrative examples (Supplementary material, *Examples*, pp. 2–4), then, for each experiential sequence, consulted the catalog of NDE pictograms (Supplementary material, *Catalog*, pp. 1–3) and selected the pictogram that best matched the perceived boundary of the space they saw or experienced themselves as being within during that specific experiential sequence of their NDE. This selection was made by the participant: the researcher did not indicate which pictogram was preferable or decide between neighboring geometric alternatives. This initial pictogram-selection phase provided a standardized geometric descriptive framework for the quantitative classification and comparison of participants' reconstructions.

Because participants were recruited through the International Association for Near-Death Studies (IANDS), a nonprofit organization focused on the study and dissemination of information related to near-death experiences, participants may have been familiar with common NDE narratives and terminology. To reduce semantic priming effects, the reconstruction task focused primarily on abstract visuo-spatial boundary geometries rather than predefined semantic categories. Terms commonly associated with NDE narratives, such as “tunnel,” “light,” or “heavenly landscape,” were intentionally not used within the reconstruction task itself. Instead, participants selected and modified geometric pictograms representing perceived spatial boundaries (Supplementary material, *Catalog*, pp. 1–3).

Importantly, participants were not restricted to the predefined pictograms. If none of the proposed pictograms corresponded to the visuo-spatial configuration perceived during a given experiential sequence, participants selected “Other Space" and produced a freehand drawing of that configuration. If the experiential sequence did not include any spatial representation, participants selected “No Space” (Supplementary material, *Catalog*, p. 3). If the same space was experienced more than once, the same pictogram could be reused across sequences.

After completing the spatial boundary reconstruction for that experiential sequence, participants added standardized graphical symbols to indicate Self-Locations (S-L) and Self-Motion (S-M).

Self-Locations were marked with numbered crosses ( × ) and labeled using the abbreviation S-L, for example S-L1 and S-L2. The numbering reflected their chronological order of emergence within the experiential sequence.

Self-Motion was represented with arrows ( → ) indicating directional trajectories, with U-shaped arrows denoting reversals in direction.

Squares marked the spatial location of the Visual Mental Imagery, such as non-physical presences (e.g., spiritual guides, angels, or deceased relatives) (Supplementary material, *Catalog*, p. 3).

The graphic reconstructions therefore encoded the NDE spatial boundary, the temporal organization of Self-Locations, the trajectories of Self-Motion, and the spatial location of Visual Mental Imagery within the visuo-spatial configuration of each experiential sequence.

For OBE Dissociative and OBE Associative sequences, graphical annotations followed the legend system provided in the Main Questionnaire (Supplementary material, *Main Questionnaire*, p. 6) and are therefore not described in further detail here.

All drawings followed a fixed spatial convention: the bottom of the page represented the ground or physical floor, and the top indicated the ceiling, sky, or upward extension, maintaining a vertical frame aligned with gravitational orientation. Upon completion of the reconstruction task, participants scanned the original drawings and submitted them to the researcher by email as JPEG files. Blue ink was used to distinguish participants' handwritten annotations from the pre-printed questionnaire pages. The submitted drawings were then anonymized and converted to grayscale using Adobe Photoshop 2024 (version 25.4; Adobe Inc., San Jose, CA, USA).

### 2.7 Digital questionnaire data collection

The questionnaire comprised both closed-ended check-box items and open-ended text fields. The open-ended section elicited qualitative descriptions of each NDE space sequence (Supplementary Data, pp. 3–20). To preserve the authenticity of these descriptions, only minor spelling corrections were made. The closed-ended section systematically recorded, for each spatial sequence: (i) the total number of Self-Locations mapped within the sequence, (ii) the Self-Motion velocity profiles, (iii) the emotional valence and intensity ratings associated with each Self-Location, and (iv) the sensory/perceptual modalities experienced in the NDE space (e.g., visual, auditory, haptic, etc.).

For each experiential sequence, participants completed the corresponding questionnaire section while keeping the associated graphic reconstruction in view. This ensured that the visual record they had just produced directly informed their categorical and descriptive responses, reducing recall bias and supporting accurate mapping between drawn and verbal content. The questionnaire therefore combined graphic reconstruction tasks with digital self-report measures assessing spatial boundaries, Self-Locations, Self-Motion trajectories and velocity profiles, emotional valence and intensity, sensory-perceptual modalities, and qualitative reports associated with the reconstructed spaces (Supplementary material, *Main Questionnaire*).

In the present study, only variables (i) and (ii) were analyzed, and within the verbal descriptions we examined only the perceptual appearance of the NDE spaces, specifically their chromaticity qualities rather than the full narrative content.

### 2.8 Statistical analysis

The unit of analysis depended on the question being examined. Participant-level analyses, including demographic characteristics, the number of reconstructed NDE parts per participant, and the co-occurrence analysis presented in Figure 1, were based on the 50 participants who completed the main questionnaire. As a secondary non-response analysis, NDE-C and Greyson NDE Scale scores were compared between eligible volunteers who proceeded to the main study and those who did not respond to subsequent invitations using Welch's independent-samples *t*-tests.

**Figure 1:**
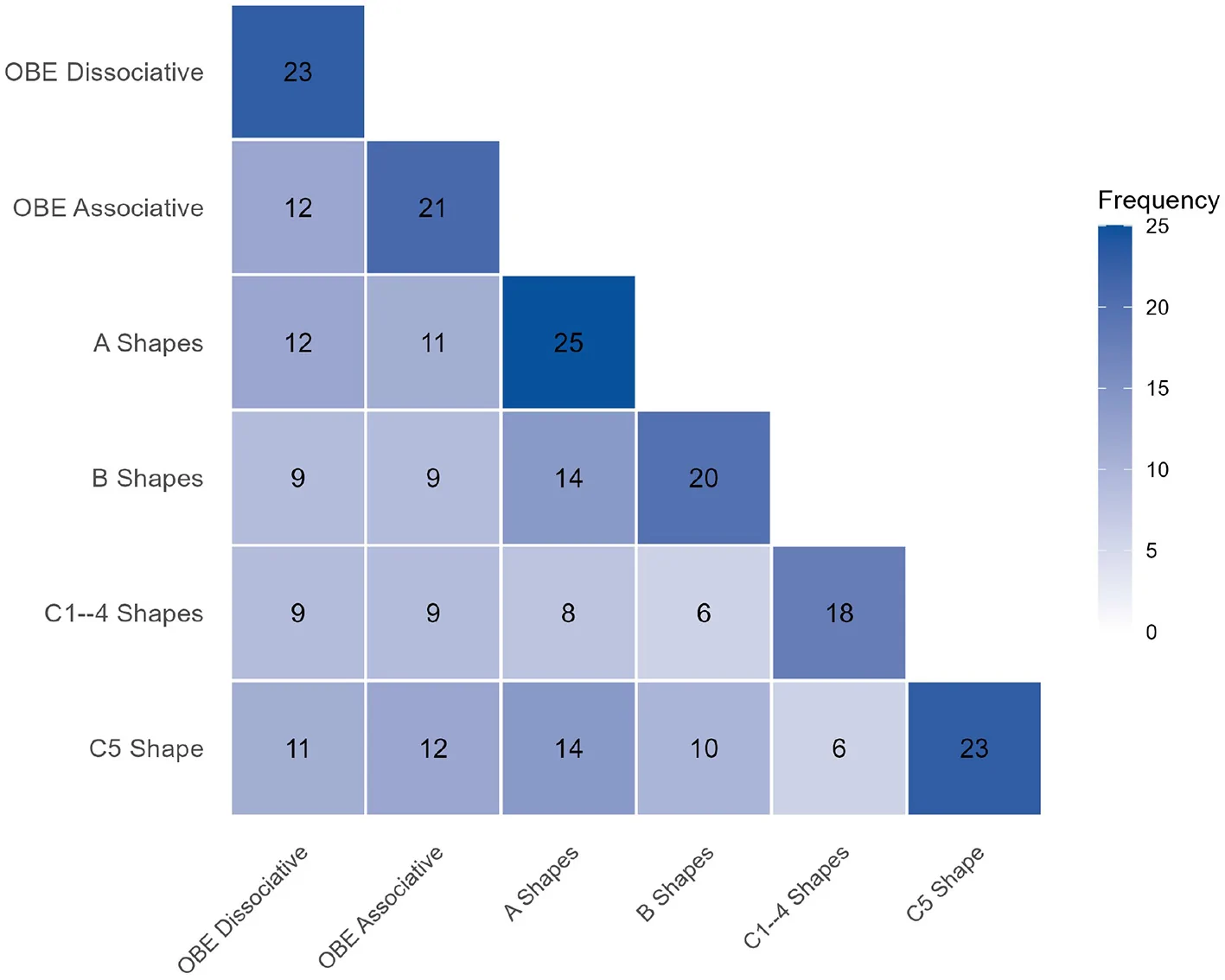
Participant-level co-occurrence matrix of OBE parts and NDE visual-space categories. The matrix was computed across all 50 participants, including participants with Other Space or No Space responses, although these categories are not displayed in the matrix. Each cell indicates the number of participants who reported both categories at least once within their complete reconstructed sequence. Diagonal values indicate the number of participants who reported each category at least once. Repeated spaces from the same category within the same participant were counted only once. These are participant-level counts and should not be interpreted as the total number of reconstructed NDE spaces.

Analyses of shape category, chromaticity, Visual Field characteristics, and positional relationships were conducted at the reconstructed-space level because participants could contribute more than one reconstructed NDE space. Analyses comparing the predefined Visual Field categories were restricted to the 108 reconstructed spaces classified as A, B, C1–C4, or C5; the three “Other Space” cases and the single “No Space” case were retained in the descriptive accounting of NDE experiential parts but excluded from category-based analyses.

The association between Visual Field category and chromaticity was examined at the reconstructed-space level using the Fisher–Freeman–Halton exact test as the omnibus exact test for the 4 × 2 contingency table, alongside the Pearson χ^2^ statistic and Cramer's *V* effect size. Pairwise comparisons between categories were conducted using Fisher's exact tests with Holm correction.

To account for the non-independence of repeated reconstructed spaces contributed by the same participant, the association between Visual Field category and chromaticity was also estimated using generalized estimating equations (GEE). Chromaticity was modeled as a binary outcome, with Visual Field category as the predictor of interest. The model used a binomial distribution with a logit link, participant-level clustering, an exchangeable working correlation structure, and robust sandwich standard errors. The participant identifier was used as the clustering variable; 47 of the 50 participants contributed at least one reconstructed space classified as A, B, C1–4, or C5 and therefore contributed to the GEE analysis. Analyses were conducted in Python using the statsmodels package. As a sensitivity analysis, variance estimates were also recomputed using an approximate Mancl–DeRouen bias-corrected sandwich estimator (Mancl and DeRouen, 2001), given the moderate number of clusters (*n* = 47).

The middle-position analysis was retained as a descriptive reconstructed-space-level analysis of the relative frequency of shape categories across sequence positions. It was not interpreted as a participant-level clustered inference.

To examine whether NDE intensity was associated with the visuo-spatial categories, we conducted an exploratory participant-level analysis using the Greyson NDE Scale and NDE-C total scores. For each visuo-spatial category, participants were classified according to whether they reported at least one A, B, C1–4, or C5 space. Greyson and NDE-C scores were then compared between participants with vs. without each category using Welch's independent-samples *t*-tests. For each comparison, we report group sizes, descriptive statistics, Hedges' *g* with bootstrap 95% confidence intervals (based on 10,000 resamples with independent resampling within each group, random seed = 42), uncorrected *p*-values, and Holm-corrected *p*-values. Holm corrections were computed both across all eight comparisons and within each psychometric scale family.

## 3 Results

The results are presented in three sections.

1. We examined the distribution of OBE components (Dissociative, Associative, or both) and the categorization of NDE spatial forms into type A, B and C shapes.
2. We analyzed the geometric characteristics of NDE Visual Fields, focusing on Visual Field Extent.
3. We analyzed the relationship between Self-Location, Self-Motion, and Visual Field Extent outlines to determine how these factors influenced the perceptual qualities of the Visual Field boundary.

### 3.1 Distribution of OBE and NDE shape categories (A, B, C)

Among the 97 eligible volunteers, NDE-C scores did not differ significantly between those who proceeded to the main study (*M* = 55.12, *SD* = 13.20) and those who did not respond to subsequent invitations (*M* = 51.98, *SD* = 13.50; Welch's *t*-test, *p* = 0.250). Greyson NDE Scale scores were likewise similar between those who proceeded (*M* = 19.52, *SD* = 5.46) and those who did not respond (*M* = 18.72, *SD* = 6.71; Welch's *t*-test, *p* = 0.524). Thus, no evidence was found that non-response following eligibility screening was associated with NDE intensity as measured by either psychometric scale. The final study sample comprised 50 participants who completed both the preliminary questionnaire and the main questionnaire and graphic reconstruction procedure. Participants had a mean age of 52.3 years at the time of the study (*SD* = 13.9; median = 51.8; range = 22.4–85.0), and the sample comprised 35 women (70%) and 15 men (30%). Greyson NDE Scale scores had a mean of 19.52 (*SD* = 5.46; median = 20; range = 7–29), and NDE-C scores had a mean of 55.12 (*SD* = 13.20; median = 54.5; range = 31–76).

The reported NDEs had occurred a median of 15.1 years before study participation (*IQR* = 2.8–32.6 years; mean = 20.0, *SD* = 19.5; range = 0.14–79.2 years). Forty-two participants (84%) retrospectively characterized the circumstances of their experience as life-threatening, whereas eight (16%) characterized them as non-life-threatening. As described in Section 2.2, life-threatening status was based on retrospective self-report and was not independently medically verified. The self-reported circumstances associated with the experiences were heterogeneous, and the participant-level descriptions of these circumstances are provided in the Supplementary material (*Preliminary Anonymized Questionnaire Answers*).

Fifty subjects completed the main digital questionnaire and produced graphic representations according to its protocol. They were asked to specify the number of parts in their NDE, indicate whether they experienced an OBE, and locate it chronologically within the NDE. Participants were also instructed to distinguish between a Dissociative OBE (subjective impression of separating from the physical body) and an Associative OBE (subjective impression of reuniting with the physical body). Among the 50 participants, 18 reported only a NDE without any OBE component (Subjects 2, 3, 16, 18, 22, 23, 25, 26, 27, 28, 31, 32, 35, 36, 38, 43, 45, 48). Eleven described a Dissociative OBE component with an NDE but not the Associative type (Subjects 1, 4, 7, 8, 13, 17, 19, 37, 41, 47, 50). Nine reported an Associative OBE component but not the Dissociative type (Subjects 9, 10, 12, 15, 20, 21, 40, 46, 49). Twelve experienced both Dissociative and Associative OBEs within the same sequence (Subjects 5, 6, 11, 14, 24, 29, 30, 33, 34, 39, 42, 44). This distribution shows that OBEs are frequent but not universal within NDEs, with Dissociative and Associative types occurring in nearly equal measure, and some participants experiencing both as transitional phases within the broader NDE structure (Figure 1).

Table 1 shows the distribution of the number of NDE sequences reported by the 50 participants. The median number of segments is 2, while the mean is 2.24. For the 21 participants who reported a single NDE sequence, the distribution of the selected shapes is presented in Supplementary Table S1, while all available shape categories are illustrated in the Supplementary material (*Catalog*, pp. 1–3).

**Table 1:** Distribution of the number of NDE parts per subject (*n* = 50).

Number of parts	1	2	3	4	5	6	8
Count	21	14	7	3	2	2	1
Relative frequency	42%	28%	14%	6%	4%	4%	2%

The median number of segments is 2, and the mean is 2.24.

Across the 50 participants, 112 NDE experiential parts were reconstructed. Of these, 108 were classified within the predefined Visual Field categories: 28 A spaces, 31 B spaces, 20 C1–C4 spaces, and 29 C5 spaces. The remaining four experiential parts comprised three “Other Space” reconstructions and one “No Space” response. These four parts were not included in analyses comparing the A, B, C1–C4, and C5 Visual Field categories.

These shape categories are described precisely in Supplementary Data (pp. 1–2). We briefly recall their meaning:

*Type A shapes*: cone-shaped forms delimited by a pair of lines. The numbered subclasses A1–A12 differ according to whether these lines are parallel, convergent, or divergent, as well as according to their orientation in the drawing.

*Type B shapes*: portions of circles or ellipses. The subclasses B1–B12 differ depending on whether these portions cover a quarter, a half, or three quarters of a clock face.

*Type C1–C4 shapes*: almost flat circular arcs covering a small angular amplitude (less than 30°), representing what is obtained by zooming in on a boundary point of a circle. The subclasses C1–C4 differ according to their orientation in the drawing.

*Type C5 shapes*: a full circle or a full ellipse.

For the OBE, Supplementary Table S2 presents the Self-Motion orientations of the 10 participants who experienced both a Dissociative and an Associative OBE with rectilinear Self-Motion trajectories (Subjects 6 and 24 experienced both a Dissociative and an Associative OBE but their Self-Motion was rotational). The angles were measured in a reference frame where 0° corresponds to the horizontal axis pointing right and 90° to the vertical axis pointing upward on the page. The last column reports the difference between these two angles, which represents the angular deviation undergone by Self-Motion during the experiment, from the Dissociative OBE at the beginning to the Associative OBE at the end.

We observed that A shapes (cone-shaped forms delimited by a pair of lines) tend to appear at the beginning or end of the sequence. To test this, we examined NDEs comprising more than one part and measured how often each shape type appeared in the middle (i.e., neither first nor last). Supplementary Table S3 reports the proportion of middle occurrences for each shape category together with Fisher's exact tests, risk differences, and 95% confidence intervals comparing each category with A shapes. This analysis was conducted at the level of individual reconstructed spaces, as it focuses on the relative frequency of shape categories across sequence positions rather than on subject-level inference. Statistical significance was assessed using a Bonferroni-corrected threshold of *p* < 0.0167.

C5 was reported by 23 participants in total. Among them, 18 reported sequences containing more than one reconstructed NDE space. Supplementary Table S4 shows which shapes appeared before and after C5, and Supplementary Table S6 indicates the pages in Supplementary material, *Graphic Representations* that contain the drawings of the OBE for each subject.

To further examine the relationships between different NDE space types and OBE components, we analyzed their co-occurrence at the participant level, without considering their sequential order (Figure 1). In this analysis, each cell represents the number of participants who reported both categories at least once within their complete reconstructed sequence, whereas diagonal values represent the number of participants who reported each category at least once.

To examine whether NDE intensity was associated with Visual Field geometry, we conducted an exploratory participant-level analysis using the Greyson NDE Scale and NDE-C total scores. For each Visual Field category, participants were classified according to whether they reported at least one A, B, C1–C4, or C5 space. Group sizes were A: 25 with vs. 25 without; B: 20 with vs. 30 without; C1–C4: 18 with vs. 32 without; and C5: 23 with vs. 27 without.

Participants reporting at least one A space had higher Greyson NDE Scale scores than participants without an A space [*M* = 21.2 vs. *M* = 17.8; Hedges' *g* = 0.648, 95% CI (0.107, 1.307), uncorrected *p* = 0.024] and higher NDE-C scores [*M* = 59.2 vs. *M* = 51.1; Hedges' *g* = 0.627, 95% CI (0.087, 1.288), uncorrected *p* = 0.029]. NDE-C scores were also higher among participants reporting at least one B space [*M* = 59.7 vs. *M* = 52.1; Hedges' *g* = 0.588, 95% CI (0.043, 1.235), uncorrected *p* = 0.040] and among those reporting at least one C5 space [*M* = 59.1 vs. *M* = 51.7; Hedges' *g* = 0.565, 95% CI (0.036, 1.193), uncorrected *p* = 0.046]. The corresponding Greyson comparisons for B and C5 were in the same direction but were not statistically significant (*p* = 0.10 and *p* = 0.12, respectively). No differences were observed for C1–C4 spaces on either scale (Greyson: *p* = 0.58; NDE-C: *p* = 0.70).

After Holm correction for multiple comparisons, none of the eight comparisons remained statistically significant, either when correcting across all comparisons or within each psychometric scale family. Thus, although moderate uncorrected differences were observed for A, B, and C5 categories, the analysis does not provide robust evidence for category-specific associations between Visual Field geometry and psychometric NDE intensity.

### 3.2 NDE Visual Field geometric characteristics

Based on the classification of space types (Supplementary Data, pp 1–2), we quantified the geometric properties of Visual Field boundaries relative to Self-Location. In each drawing, participants marked their Self-Location within the depicted space. For B and C1–4 shapes, the Visual Field Extent was defined as the angle subtended by the shape when viewed from the Self-Location.

The A shapes A5 through A12 represent conical shapes delimited by two boundary lines, for which we denote the opening angle at the apex by θ. By convention, we set θ = 0 in the limiting case where the two boundary lines either coincide or are parallel, corresponding to the A shapes A1 through A4. For the A shapes, the VFE is the set of directions from the Self-Location that remain within the wedge indefinitely (the recession cone). This angle necessarily has the same measure as the opening angle at the apex. In particular, it is zero when θ = 0 (shapes A1, A2, A3, and A4). In this case, the visual field degenerates into a half-line. Among the 28 A shapes, θ = 0 for 6 of them.

Figure 2 illustrates these definitions: the lower panel shows the opening angle θ between the two boundary lines of an A shape, while VFE represents the enclosed Visual Field Extent at the Self-Location.

**Figure 2:**
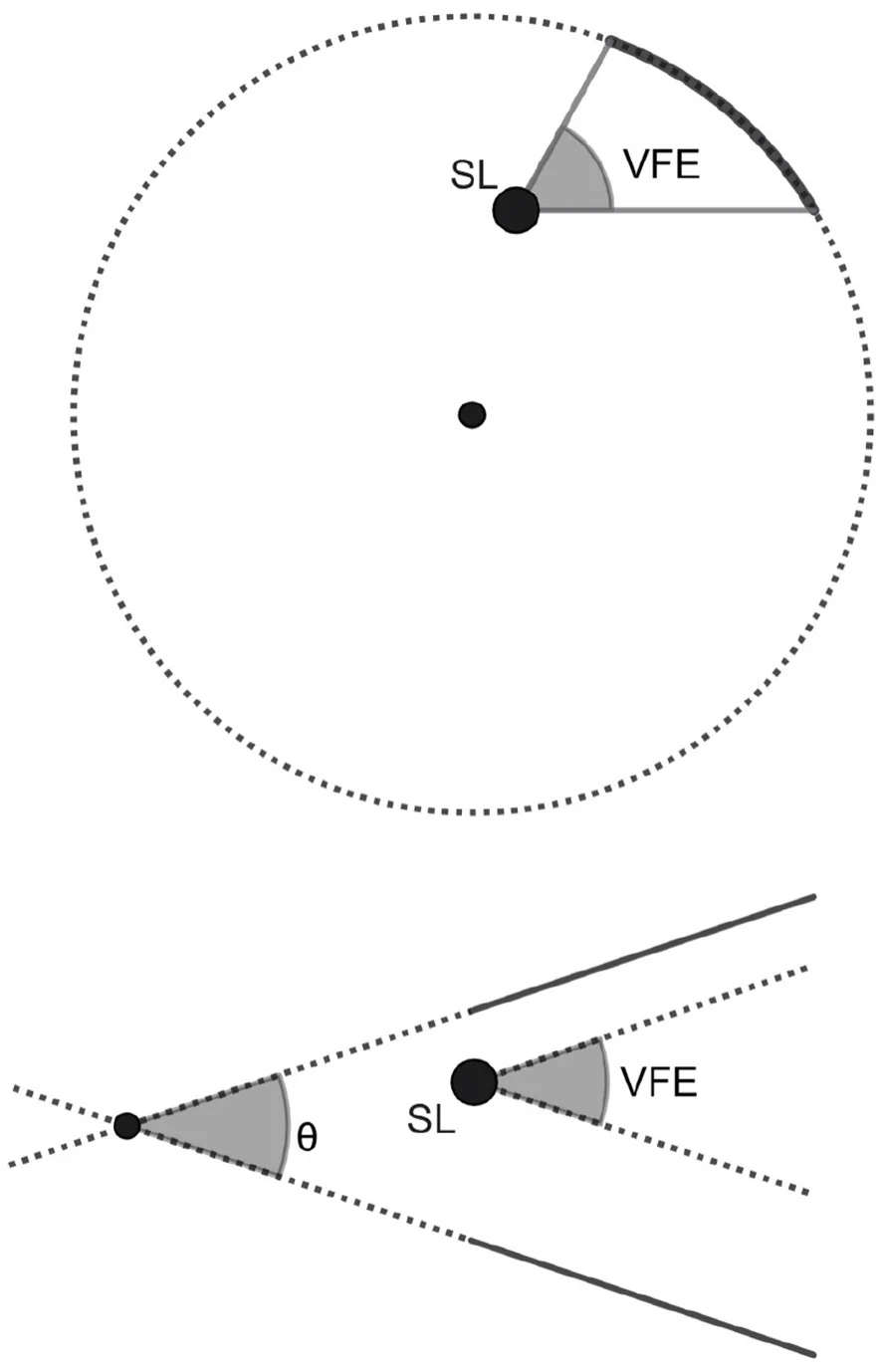
Measurement of Visual Field Extent (VFE) and opening angle in NDE graphic representations. **Upper panel:** ellipsoidal shape with Self-Location (SL), where VFE spans the two edges visible from SL; **Lower panel:** opening angle θ for an A shape, together with the VFE with respect to some Self-Location.

The upper panel shows a circular shape with a Self-Location, where VFE is the angular extent between the Self-Location and the two edges, indicating the perceived range of vision. These measures enabled quantitative comparison of visual perspectives reported by NDErs across spatial configurations.

For A shapes, the distribution of opening angles is shown in Supplementary Figure S1. Supplementary Figure S2 presents the distributions of Visual Field Extents for B shapes and C1–4 shapes (excluding C5). In both figure panels, box plots display the median, and histograms display the mean (orange lines).

We additionally examined, on an exploratory basis, whether the opening angle of an A shape correlated with the Visual Field Extent of the Self-Location in a subsequent ellipsoidal shape (B or C1–4) within the same NDE sequence. When there were multiple Self-Locations, we measured the VFE from the first one, as it was the one immediately following the A shape. Across the 12 available paired occurrences (Supplementary Table S5), the Pearson correlation between A-shape opening angle and subsequent VFE was close to zero and not significant [*r* = −0.057, *n* = 12, *p* = 0.860, 95% CI (−0.611, 0.534)]. The wide confidence interval indicates that this exploratory analysis does not allow firm conclusions regarding the presence or absence of a linear relationship between A-shape opening angle and subsequent VFE.

Figure 3 upper panel illustrates B7, where the arc has a high angular amplitude (half-circle) and pronounced curvature. The Self-Location lies relatively close to the arc, resulting in a larger Visual Field Extent. In contrast, the lower panel illustrates C3, where the arc has low curvature and the angular amplitude is reduced, approaching a flat profile. Here, the Self-Location is located farther from the arc relative to its radius of curvature, yielding a smaller Visual Field Extent. Together, these examples demonstrate how both curvature and Self-Location distance modulate the Visual Field Extent.

**Figure 3:**
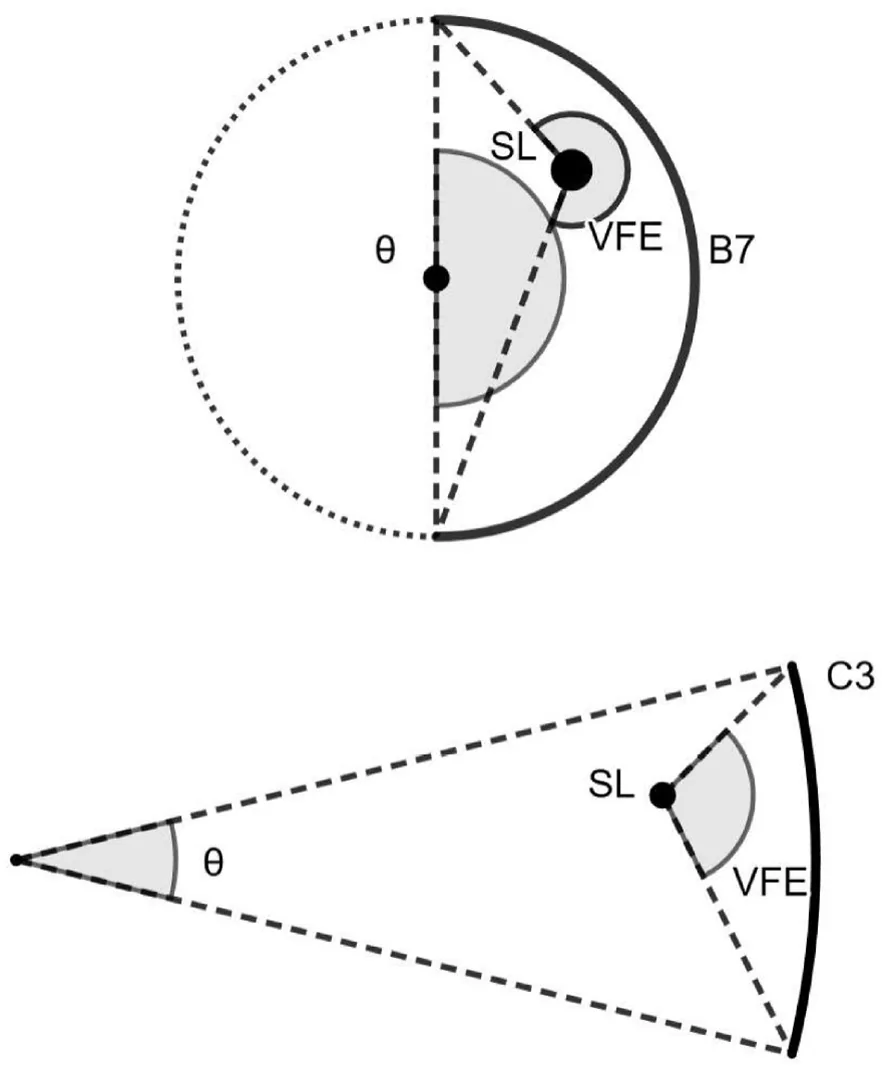
Examples illustrating how curvature and Self-Location distance modulate Visual Field Extent. The central angle of the shape is denoted by θ. **Upper panel:** B7 with high angular amplitude and pronounced curvature; **Lower panel:** C3 with low curvature and reduced angular amplitude.

### 3.3 NDE Visual Field perceptual qualities and Visual Mental Imagery

Figure 4 presents the distribution of chromatic vs. achromatic appearances across shape categories, with C5 treated separately. The chromaticity classification was derived from participants' responses to Question 33 of the Supplementary material (*Main Questionnaire*, p. 29), in which they indicated whether the experienced NDE space was chromatic (“all the colors, e.g., pink”) or achromatic (“black, gray, white and light textures”). Type A shape spaces (*n* = 28) were mostly achromatic (22 spaces, 78.6%), whereas type B shape spaces (*n* = 31) were predominantly chromatic (27 spaces, 87.1%). C1–4 spaces (*n* = 20) showed a more balanced pattern (13 chromatic spaces, 65.0%; 7 achromatic spaces, 35.0%), and C5 spaces (*n* = 29) showed a mixed chromatic–achromatic distribution (17 chromatic spaces, 58.6%; 12 achromatic spaces, 41.4%).

**Figure 4:**
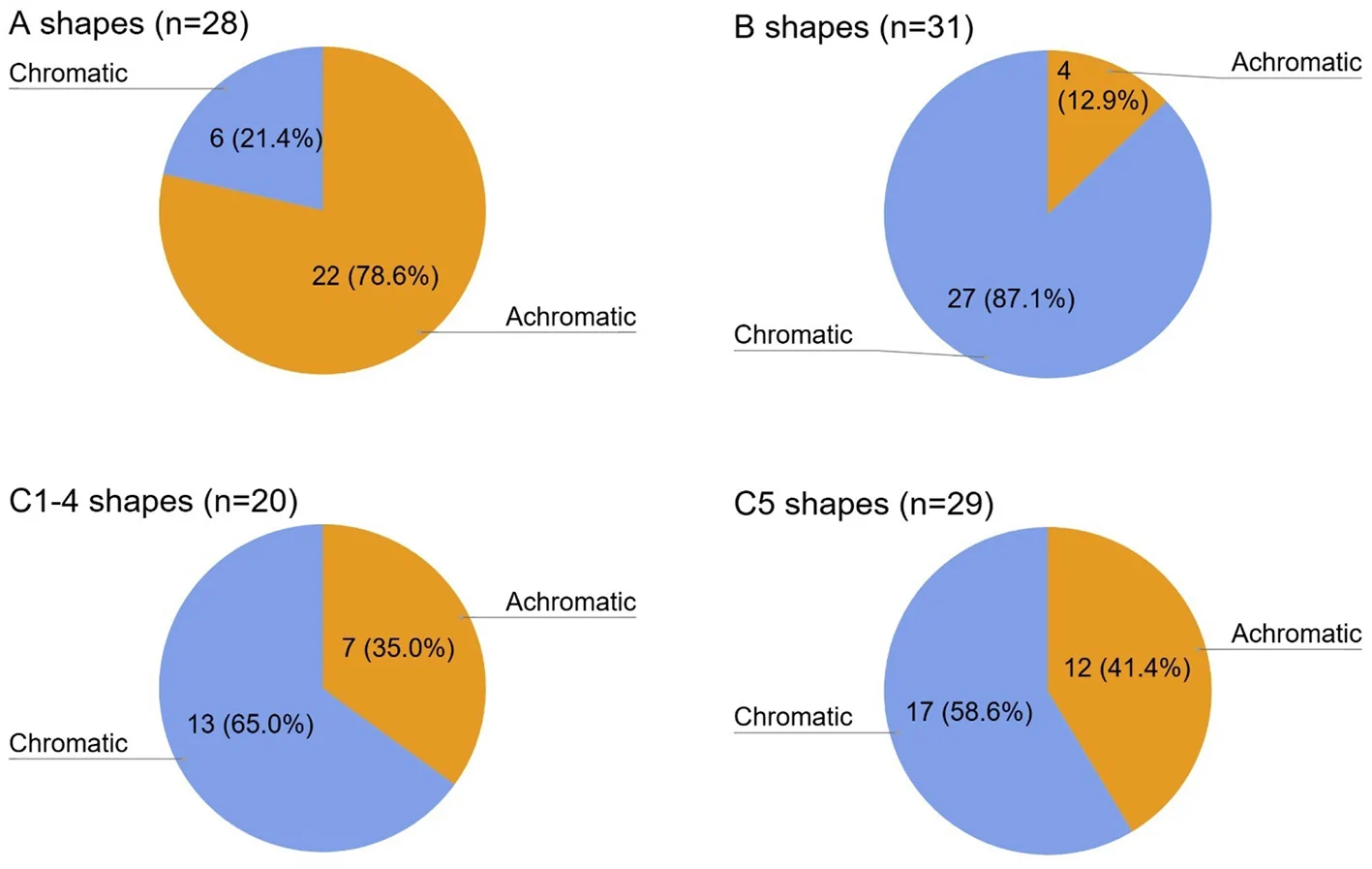
Distribution of color palette of spaces by shape category (A, B, C1–4, C5), where chromatic refers to all hues (e.g. pink, red, blue), and achromatic refers to black, white, gray, and light textures.

The corresponding chromatic proportions together with their 95% Wilson confidence intervals are shown in Supplementary Figure S3. A Pearson χ^2^ test indicated a significant association between Visual Field category and chromaticity ($\chi_{\left(3\right)}^{2}=26.61$, *p* = 7.11 × 10^−6^; Cramer's *V* = 0.496). A Fisher–Freeman–Halton exact test confirmed this association (*p* = 3.71 × 10^−6^). Pairwise Fisher's exact tests with Holm correction showed that this association was driven by the A category: a differed significantly from B (*p*_Holm_ < 0.0001), C1–4 (*p*_Holm_ = 0.016), and C5 (*p*_Holm_ = 0.027), whereas no significant differences were found between B and C1–4 (*p*_Holm_ = 0.171), B and C5 (*p*_Holm_ = 0.057), or C1–4 and C5 (*p*_Holm_ = 0.769).

To account for the non-independence of repeated reconstructed spaces contributed by the same participant, the analyses were repeated using generalized estimating equations (GEE) with a logit link function, an exchangeable working correlation structure, and robust sandwich variance estimators. Analyses were conducted in Python using the statsmodels package. Relative to type A spaces, the odds of a chromatic report were significantly higher for type B spaces [OR = 18.4, 95% CI (4.73, 71.87), *p* = 2.7 × 10^−5^], C1–4 spaces [OR = 5.31, 95% CI (1.28, 22.00), *p* = 0.021], and C5 spaces [OR = 4.20, 95% CI (1.29, 13.71), *p* = 0.017]. These results are shown in Supplementary Figure S4a.

To examine whether sequence position within the NDE (early vs. late) influenced chromatic reports, the GEE models were re-run with normalized sequence position included as a covariate. Sequence position was coded using the Weibull plotting position *k*/(*n*+1), where *k* is the rank of the space in a sequence of *n* spaces (Weibull, 1939). The odds ratios remained essentially unchanged [B: OR = 18.8, 95% CI (4.43, 79.45); C1–4: OR = 5.43, 95% CI (1.24, 23.74); C5: OR = 4.29, 95% CI (1.33, 13.80)]. There was no evidence of an association between sequence position and chromaticity [OR = 0.89, 95% CI (0.16, 5.08), *p* = 0.89]; however, the wide confidence interval precludes a precise estimate of the effect. These results are shown in Supplementary Figure S4b.

With *n* = 47 clusters, conventional sandwich variance estimates may underestimate the sampling variability of the regression coefficients (Mancl and DeRouen, 2001). As a sensitivity analysis, we therefore recomputed the variance estimates using an approximate Mancl–DeRouen bias-corrected sandwich estimator for the GEE model with a logit link, exchangeable working correlation, and participant-level clustering. Under this correction, the odds ratios were unchanged, while the confidence intervals widened [B: 95% CI (3.45, 98.50); C1–4: 95% CI (0.99, 28.56); C5: 95% CI (0.96, 18.31)]. The association for type B remained statistically supported, whereas the confidence intervals for C1–4 and C5 included unity. Taken together with the Holm-corrected pairwise Fisher tests, the most consistent contrast across analyses was between A and B. Evidence for differences between A and C1–4 and between A and C5 was sensitive to analytic method, while B, C1–4, and C5 were not significantly different from one another in the pairwise Fisher tests.

For C5, there were 12 achromatic and 17 chromatic occurrences. The achromatic occurrences included Subjects 1, 5, 10, 14, 17, 20, 21, 25, 40, 42, 43, and 48; the chromatic occurrences included Subjects 6 (two spaces), 11 (three spaces), 18, 21, 22, 23, 39, 41, 43, 44, 45, 46 (two spaces), and 47. Subjects 21 and 43 are included in both groups because C5 occurred twice within their NDE sequence, with each occurrence receiving a different chromaticity classification. For Subject 21, the first C5 occurrence (second space in the NDE sequence) was chromatic, whereas the second C5 occurrence (sixth space) was achromatic (Supplementary material, *Graphic Representations*, Subject 21, pp. 101 and 105; *Sequences*, Subject 21, p. 8). For Subject 43, the first C5 occurrence (first space in the NDE sequence) was achromatic, whereas the second C5 occurrence (second space) was chromatic (Supplementary material, *Graphic Representations*, Subject 43, pp. 181–182; *Sequences*, Subject 43, p. 12).

## 4 Discussion

Most NDE studies have not systematically examined how the experience unfolds through the characterization of distinct Visual Field boundary types. In contrast, the present study uses participants' segmentation of their NDEs into discrete sequences, each linked to a distinct visuo-spatial configuration. This enables a chronologically ordered analysis of recurrent Visual Field features and perceptual transitions across the temporal course of the experience.

Recent large-scale work associated with the Dreamachine project demonstrates the use of participant drawings as a method for documenting subjective visual phenomenology under controlled stroboscopic stimulation. A corpus of 10,598 drawings produced following the Dreamachine immersive experience was analyzed, providing visual representations of experiences elicited by stroboscopic white-light stimulation (Grove et al., 2026). Building on this methodological precedent, the present Discussion considers how participants' graphic reconstructions and written reports together contribute to a multidimensional description of the spatio-phenomenological organization of OBEs and NDEs.

The discussion is organized around four key points emerging from the analysis:

1. Sequencing of OBE and NDE experiences;
2. Differentiation between OBEs and NDEs;
3. NDE Visual Field boundaries;
4. Chromatic qualities within the Visual Field.

### 4.1 Sequencing OBEs and NDEs: isolating the structure of the experiential NDE Core

The sequencing task enabled participants to divide their experiences into temporally ordered phases, thereby delineating an NDE Core that served as the basis for the sequential analyses presented below.

After reviewing the lexicon (Supplementary material, *Catalog*, p. 4), participants segmented their experience into distinct phases and numbered them according to their chronological order, assigning a corresponding pictogram to each phase (Supplementary material, *Catalog*, pp. 1–3). OBEs were consistently structured into two components, Dissociative and Associative, visually encoded using standardized symbols: a circle for the participant's physical body, a cross for Self-Location, arrows for Self-Motion direction, and squares representing other physical beings present in the physical space (e.g., nurses, relatives, physicians, or animals). The Dissociative OBE was characterized by upward or lateral Self-Motion away from the body, whereas the Associative OBE involved a downward Self-Motion with a sense of re-embodiment. A list of subjects and corresponding graphic representations for each phase is provided in Supplementary Table S6.

Participants' graphic representations revealed consistent differences in Self-Motion orientation between the two OBE phases. Self-Motion was measured using a shared reference frame, with 0° defined as horizontal Self-Motion to the right of the page. The relative angle between Dissociative and Associative orientations was independent of this convention. Across subjects, relative angles were typically close to 180°, reflecting opposite orientations between the two OBEs (Supplementary Table S2). This pattern should be interpreted in relation to the operational definitions used in the protocol, in which Dissociative and Associative OBEs were distinguished by separation from and re-embodiment into the physical body, respectively. Exceptions, such as Subject 24, involved rotational Self-Motions of 90°, geometrically conjugated by 180° rotation (Supplementary material, *Graphic Representations*, pp. 115–117).

The sequencing protocol instructed participants to position Dissociative OBEs at the onset and Associative OBEs at the conclusion of their NDE sequencing, enabling consistent isolation of a NDE Core, the experiential sequence(s) situated between the two OBEs. The resulting tripartite framework: Dissociative OBE → NDE Core → Associative OBE provided a coherent structure for cross-subject spatial analyses (Supplementary material, *Sequences*). The overall participant-level co-occurrence between Dissociative and Associative OBEs and the NDE shape categories A1–12, B1–12, C1–4, and C5 is summarized in Figure 1, which shows the number of participants reporting each pair of categories at least once within their complete reconstructed sequence.

### 4.2 The differentiation of OBEs and NDEs

A key empirical distinction between OBEs and NDEs in our study lies in how participants represented the physical body and its physical space. In OBE reconstructions, particularly during the Dissociative phase, both body and spatial context (e.g., the room) were systematically depicted, showing that bodily visibility and spatial grounding were integral to the experience. These depictions indicate that the self was perceived as extracorporeally located yet still visually tethered to the body and its surroundings (Blanke, 2004; Blanke et al., 2016). In contrast, the physical body and surrounding physical space were not depicted in the NDE reconstructions. However, because physical-body location was explicitly prompted in the OBE drawing task but not in the NDE drawing task, this difference in representation cannot by itself be interpreted as evidence that the physical body and surrounding environment ceased to serve as reference points for Self-Location. The distinction can be clarified through the framework of Haun and Tononi (2019), who state: “The term space can be taken to refer to a variety of concepts: there is the physical space of the world that we take to exist independent of our knowledge of it (‘outer space'); there is spatial cognition, the psychological model of physical space that an animal builds and uses to navigate and understand the world; and there is phenomenal space, the fact that some aspects of experience have a spatial structure.” Interpreted in this context, OBE reconstructions represented physical space with a reference frame that included the physical body, whereas the NDE reconstructions focused on the experienced phenomenal space. Given the asymmetry between the two drawing tasks, however, this representational difference cannot independently establish that physical-body anchoring disappears during the NDE. Importantly, the distinction between OBE components and the NDE Core was not inferred solely from the presence or absence of physical-body representation. The operational definitions and procedure for distinguishing these experiential components were specified *a priori* in the questionnaire: an OBE was defined by an extracorporeal viewpoint involving perception of the physical body and/or surrounding physical space, and participants first identified whether their experience contained an OBE component. When no OBE was reported, participants proceeded directly to the segmentation of their NDE into numbered experiential parts. The NDE Core was subsequently identified through this participant-led segmentation and chronological organization of the experiential components.

In NDEs, the physical body is often reported as no longer felt and is replaced by a self lived through a first-person viewpoint. The OBE–NDE transition can thus be interpreted phenomenologically as a shift of embodiment from the physical body, now perceived as insentient, to a self lived through a first-person viewpoint, consistent with Lucci and Pazzaglia (2015).

To illustrate this distinction, two representative graphic reconstructions were contrasted. The first is a Dissociative OBE followed by an NDE space of any ABC type; in one example, the Dissociative OBE is followed by a conical A1 shape (Supplementary material, *Graphic Representations*, Subject 5, p. 18 for the OBE Dissociative and p. 19 for A1). The second example shows an Associative OBE preceded by a conical A5 shape (Supplementary material, *Graphic Representations*, Subject 9, p. 40 for A5 and p. 41 for the OBE Associative). Together, these examples highlight a progressive shift in self-perception: in OBEs, the experiential viewpoint transitions from the physical body (represented by a circle) to a parasomatic Self-Location (represented by a cross). In the NDE reconstructions, Self-Location was consistently represented from a first-person perspective across the ABC-type sequences. This recurrent feature contributed to the characterization of the NDE Core identified through the combined segmentation, chronological organization, and graphic reconstruction procedure.

### 4.3 Recurrent visual-field boundary configurations of Core NDE spaces

The categorization into the type A shape family was based on geometric properties of participants' selections, with all type A shapes conceived as conical forms differing primarily in orientation either parallel or convergent (Supplementary material, *Catalog*, p. 1). The geometric parameters used to describe these Visual Field configurations are defined in Figure 2, which illustrates the measurement of the opening angle (θ) in conical type A shapes and the Visual Field Extent in ellipsoidal configurations. A key finding is that while the underlying conical geometric organization is preserved, changes in Self-Motion direction or Self-Location rotation can make the same shape appear inverted or rotated. For instance, in Subject 10, the sequence progressed from A5 to A9 through spaces B6, C5, and B7. A9 appears as the image of A5 under a 180° rotation (Supplementary material, *Sequences*, p. 5; *Graphic Representations*, A5 p. 43; A9 p. 47). This inversion reflects the reversal of Self-Motion direction, while the underlying conical geometry remains constant. Another example, from Subject 23, shows a transition from A9 to A8 through C5 (Supplementary material, *Sequences*, p. 8). Here, the type A shape rotates rather than inverts, reflecting a rotation of Self-Location within C5. The graphic representation shows A9 (Supplementary material, *Graphic Representations*, p. 111) becoming A8 under a 225° clockwise rotation (Supplementary material, *Graphic Representations*, p. 113), with a residual trace of A9's outline visible along the edge of C5 (Supplementary material, *Graphic Representations*, p. 112). Together, these cases suggest that the conical geometry of type A shapes is preserved as a recurrent geometric feature of Core NDE spaces, even when inversions or orientation changes arise relative to preceding spatial frames. The distribution of these type A, B, C1–4, and C5 type geometries across subjects with single-part NDEs is summarized in Supplementary Table S1.

The categorization into B and C shape families was likewise based on geometric properties of participants' pictogram selections, all conceived as elliptic arcs except C5, which represents a complete ellipse. By construction, the angular amplitude and curvature are high for B1–12 and low (almost flat) for C1–4. The Visual Field Extent increases as the distance between Self-Location and the arc decreases relative to the radius of curvature. Figure 3 illustrates how curvature and Self-Location distance jointly modulate the amplitude of the Visual Field Extent.

The B1–12 and C1–4 forms correspond to smaller or larger arcs of the complete ellipse represented by C5. Importantly, absolute Self-Location distance from the Visual Field boundary should not be interpreted independently of arc curvature; Visual Field Extent depends on Self-Location distance relative to the radius of curvature. Subject 20 provides an illustrative example of variation in absolute distance: in this participant's graphic representation, the Self-Location in B6 lies farther from the Visual Field boundary than in the following C2 arc, where it is closer (Supplementary material, *Graphic Representations*, pp. 95–96). This individual example is not intended to indicate a general difference in Self-Location distance between B and C1–4 categories. The relative frequency of these shapes in middle positions of NDE sequences is reported in Supplementary Table S3. Rotational shifts of Self-Location may reorient Visual Field boundaries without altering intrinsic geometry (Supplementary material, *Graphic Representations*, Subject 25, B12-C5-B10, pp. 119–121). Translational Self-Motion may also modify perceived Visual Field Extent structure. Subject 47's spaces B10 and B11 (pp. 197–198) show a transition between two elliptic arcs where the Visual Field Extent changes along a translational trajectory.

Empirically, C5 graphic representations exhibit varying ellipse eccentricities from nearly spherical or circular to distinctly ellipsoidal depending on the ratio of major and minor axes. More elongated ellipsoidal geometries appear in Subjects 1, 39, and 40 (Supplementary material, *Graphic Representations*, p. 6; p. 165; p. 168), whereas nearly spherical forms are found in Subjects 5, 6, and 41 (Supplementary material, Graphic Representations, p. 20; p. 29; p. 172). If B1–12 and C1–4 elliptic arcs derive from the complete C5 ellipsoid, their geometry may also vary with perceived C5 eccentricity, as illustrated in Subjects 6 and 12 (Supplementary material, *Graphic Representations*, pp. 25–26; pp. 63–64). These rare exceptions illustrate that the pictogram methodology was able to accommodate variations in participants' reconstructions of NDE Visual Field boundaries (Supplementary material, *Catalog*, pp. 1–2).

Within the present pictogram-based reconstruction framework, participants' graphic reconstructions were organized into three broad geometric families: conical (A1–12), partial elliptic (B1–12; C1–4), or full ellipsoidal (C5) forms. These configurations provide an exploratory descriptive organization of the reported visuo-spatial characteristics of NDEs, distinguishing conical from partial or complete ellipsoidal structures and transformations between successive Visual Field configurations (Figure 2). Because each experiential part was assigned a separate reconstruction page and associated Visual Field configuration, these successive configurations provided the basis for the temporal segmentation used in the reconstruction protocol.

This framework differentiates OBE and NDE phenomenology and characterizes recurrent Visual Field boundary configurations and their associated chromatic qualities. It can also be extended to analyze additional experiential dimensions, including self-motion velocity profiles, sense of agency, and affective components. Sequential segmentation thus provides a structured exploratory basis for examining relationships between experiential events and their reconstructed visuo-spatial characteristics. Although participants' reactions to the reconstruction procedure were not systematically analyzed, informal feedback obtained during the live sessions was predominantly positive. Participants generally reported that the proposed pictograms closely approximated the perceived boundaries of the spaces they experienced, often spontaneously commenting that particular geometric forms corresponded closely to the spatial organization they remembered. In some cases, participants initially hesitated between neighboring pictograms because the finite catalog could not represent every possible angular orientation or geometric variation of the Visual Field boundary. This hesitation was not resolved by the researcher selecting or recommending one of the alternatives. Rather, the participant selected the pictogram that most closely approximated the remembered configuration and, where necessary, modified it freehand by adjusting its orientation, curvature, proportions, width, height, or opening angle. The final reconstruction therefore reflected the participant's own selected and modified configuration rather than a geometric category assigned by the researcher.

Several participants further reported that the reconstruction process helped them organize and communicate aspects of their experience that had previously been difficult to verbalize, consistent with work suggesting that external graphic representations can facilitate the externalization, spatial organization, and communication of complex internal representations (Tversky, 2013). Several participants also spontaneously described the emergence of additional spatial, perceptual, or motion-related memories during the reconstruction process itself, particularly while locating Self-Locations and reconstructing Self-Motion trajectories.

Although not formally investigated in the present study, these observations may relate to the role of spatial and sensori-motor organization in autobiographical memory retrieval and embodied cognition, in which spatial orientation, movement trajectories, and viewpoint transformations contribute to embodied recall processes (Berthoz, 1997, 2000). From this perspective, reconstructing Self-Locations and Self-Motion trajectories may have partially reactivated perceptual, proprioceptive, and motor aspects of the original experience, consistent with theories proposing that memory retrieval relies on the simulation of previously experienced perceptual and bodily states (Barsalou, 1999). In this respect, the reconstruction procedure may have functioned not only as a descriptive phenomenological tool, but also as a structured visuo-spatial support facilitating the externalization and spatial organization of experiential aspects that participants reported as difficult to communicate through verbal description alone. The exploratory analysis of psychometric NDE intensity did not identify statistically reliable category-specific associations after correction for multiple comparisons. Although moderate uncorrected differences were observed for A, B, and C5 categories, none remained significant after Holm correction, and no differences were observed for C1–4. Thus, in this sample, the presence of a particular Visual Field category was not reliably associated with overall NDE intensity as measured by the Greyson NDE Scale or NDE-C total scores. This finding is consistent with the possibility that the visuo-spatial characterization captures a dimension at least partly distinct from global psychometric intensity, but the present exploratory analysis does not establish statistical independence or complementarity.

### 4.4 Chromatic perceptual qualities across core NDE Visual Fields

To examine the perceptual qualities within the Visual Field boundaries, we analyzed the distribution of chromatic (containing color) and achromatic (black, white, gray, or luminous textures without color) appearances across all NDE Core spatial categories: A (A1–A12), B (B1–B12), C (C1–C4), and C5, treated here as a distinct category. The following discussion addresses the A category first, then B and C, and concludes with the C5 visuo-spatial configuration. Having established chromatic–achromatic distributions across A, B, C1–4, and C5 categories (Figure 4), the distributions differed descriptively across the four Visual Field categories. A1–12 shapes were predominantly achromatic (78.6%), whereas B1–12 shapes were predominantly chromatic (87.1%). C1–4 and C5 showed more mixed chromatic–achromatic distributions. Across the statistical analyses, the most consistent chromaticity contrast was between A and B, whereas evidence for differences involving C1–4 and C5 was sensitive to analytic method.

#### 4.4.1 Category of conical Visual Field boundaries (A1–A12)

In our sample, 22 of 28 type A spaces (78.6%) were achromatic, whereas 6 (21.4%) were chromatic. Participants' accounts emphasized conical geometry and achromatic perceptual qualities. For instance, Subject 5 (A1): “all the colors around me were rather dark, gray, black of different shades” (Supplementary Data, pp. 4–5); Subject 6 (A1): “the space was silent and black…” (p. 5); Subject 13 (A5): “felt I was going through star fields; black. Saw stars that were bright but they were stationary; I was moving” (p. 9); Subject 15 (A5): “it looks like night time…” (p. 9); Subject 21 (A9): “escalator in a pure black space. Floating upwards through pitch blackness. 45 degrees, constant motion” (p. 11). Light modulation was salient in Subject 22 (A9): “moved through tunnel, started to lighten as I progressed, started black as night, finished as gray early dawn” (p. 12).

Among the 28 type A spaces, six were chromatic (Subjects 4, 7, 10, 16, 19, and 42), whereas the remaining 22 were achromatic. Five of the six chromatic A spaces occurred first in the reconstructed sequence, while Subject 42's occurred second. The occurrence of chromatic A spaces therefore indicates that the association between conical geometry and achromaticity is probabilistic rather than absolute. In some individual sequences, participants' descriptions suggest phenomenological continuity or perceptual overlap across successive experiential spaces; however, this possibility should not be interpreted as an established explanation for the chromatic A cases.

To examine whether the geometry–chromaticity association could instead reflect sequence position, we repeated the GEE analysis with normalized sequence position included as a covariate (Section 3.3). There was no evidence of an association between sequence position and chromaticity [OR = 0.89, 95% CI (0.16, 5.08), *p* = 0.89]; however, the wide confidence interval precludes a precise estimate of the effect. The estimated associations between Visual Field category and chromaticity remained essentially unchanged.

For Subject 4, the sequence began with A1 followed by B10 (Supplementary material, *Graphic Representations*, A1 p. 15; B10 p. 16). In A1, Self-Location faced the “garden of heaven”; the next space, B10, corresponded to the “pictures around wall of life.” The participant described A1: “Dark to start…saw images on the walls of my life (life review) in vivid color with sound, like a movie.” B10: “The being at the end was pure light…The garden was huge with rolling hills, tall grass…children playing, animals roaming.” This suggests that Self-Location within A1 sometimes allowed perceptual access to the next chromatic B10 panorama hosting life-review imagery. Similarly, Subject 10's sequence began with A5 followed by B6, both showing transitional chromaticity. In A5 (p. 43), Self-Location lies at the end of the conical form; in B6 (p. 44), entities appear within the expanded field (Supplementary material, *Graphic Representations*, A5 p. 43; B6 p. 44). A5: “moved very fast. Walls seemed organic with colors of green and brown…opening wider toward the end.” B6: “pattern in wall…honeycombed with thin lines of white and blue light.” (Supplementary Data, p. 7). The widening of A5 thus marks the transition to the panoramic B6 Visual Field.

The achromatic predominance of type A shapes shows phenomenological similarities to constricted Visual Fields reported in conditions associated with peripheral visual loss, including hypotensive syncope (Lambert and Wood, 1946), ocular pathologies (Edmeads, 2003), and neurological disorders (Trauzettel-Klosinski, 2010). In such conditions, peripheral color sensitivity is reduced, and constricted Visual Fields may be experienced with diminished chromatic content (Hansen et al., 2009). Similar observations have been discussed in relation to the retinotopic organization of the visual cortex, where the fovea is disproportionately represented relative to the visual periphery (Facco and Agrillo, 2012a; Blackmore and Troscianko, 1989). The conical organization of type A spaces also bears a phenomenological resemblance to reports of dark tunnels containing a distant light source frequently described in NDE narratives (Mobbs and Watt, 2011; Parnia and Fenwick, 2002).

The directional and constricted structure of these conical Visual Fields may likewise be compared with descriptions of egocentric motion perception and visually induced self-motion, in which peripheral visual organization contributes to the perception of directional motion and tunnel-like dynamics (Brandt et al., 1973). Comparable forms of Visual Field constriction have also been documented under conditions of retinal ischemia, hypoxia, and cortical anoxia, where peripheral visual processing is preferentially affected.

The achromatic predominance of type A spaces may also be compared with visual conditions in which chromatic processing is reduced. Under low illumination, vision shifts from cone-mediated photopic to rod-mediated scotopic processing, resulting in diminished color perception (Zele and Cao, 2014). Likewise, the magnocellular visual pathway is primarily involved in processing motion and luminance and exhibits limited chromatic sensitivity (Livingstone and Hubel, 1987).

Although these comparisons may help contextualize the present findings and suggest possible points of correspondence with known visual phenomena, the current study is phenomenological in nature and does not permit conclusions regarding the underlying neurophysiological mechanisms.

#### 4.4.2 Categories of elliptic arcs Visual Field boundaries (B1–12 and C1–4)

The Visual Field boundaries in categories B1–12 and C1–4 are characterized by elliptical arcs subtending less than a full ellipse. In our data, B shapes were predominantly chromatic, with 27 of 31 spaces (87.1%) featuring color. C1–4 shapes showed a smaller chromatic majority, with 13 of 20 spaces (65%) chromatic and 7 (35%) achromatic. C5 was treated separately due to its distinct Visual Field boundary, corresponding to a complete ellipse rather than an elliptic arc. In C5, the chromatic proportion was slightly lower than in C1–4, with 17 of 29 spaces (58.6%) chromatic and 12 of 29 (41.4%) achromatic.

The chromatic–achromatic distribution (Figure 4) shows descriptive variation among families: B shapes display strong chromatic predominance (87.1 vs. 12.9%), while C1–4 and C5 show higher achromatic proportions (35 and 41.4%, respectively).

To illustrate these findings, we examine participants' descriptions of B1–12 through both verbal reports and graphic representations. These reveal the chromatic qualities of the Visual Field boundaries and show that, in participants' graphic representations, B1–12 exhibit high curvature, while C1–4 display low curvature (almost flat).

Participants frequently described phenomena within type B Visual Field boundaries as vividly chromatic, with rich spectral variation. For example: “…a glass spectrum of all colors like a prism, like the Pink Floyd triangle with the rainbow spectrum” (Supplementary Data, Subject 1, Space 2, B2, p. 3; *Graphic Representations*, p. 5). “Many colors, especially greens and purple were vivid. Lavender, pink, magenta and deeper greens from bright to dark intensity! Very high clarity, more than on earth!” (Supplementary Data, Subject 17, Space 2, B10, p. 10; *Graphic Representations*, p. 84). “Life review …It appeared to be like microfilm moving through it very fast. It would pause on individual photos of myself and immediate family.” (Supplementary Data, Subject 10, Space 4, B7, p. 7; Supplementary material, *Graphic Representations*, p. 46). “The room was made of pink and gold light and was porous…” (Supplementary Data, Subject 28, Space 3, B11, p. 14; Supplementary material, *Graphic Representations*, p. 130). “It's a vast open space… completely silent, and in my consciousness the color of that place where I sat is brown and orange like soil. I was looking at planet Earth.” (Supplementary Data, Subject 27, Space 1, B6, p. 14; Supplementary material, *Graphic Representations*, p. 126). “… the “garden" was huge with rolling hills, tall grass… vivid people walking and talking, children playing, animals roaming… There were the most wonderful flowers and trees in amazing colors.” (Supplementary Data, Subject 4, Space 2, B10, p. 4; Supplementary material *Graphic Representations*, p. 16).

These illustrative descriptions convey strong chromatic richness and a pronounced sense of spatial expansiveness within type B Visual Field boundaries. Previously, we noted that rotations of Self-Location may influence the perceived orientation of the Visual Field Extent. In examining chromaticity, we further observed that the orientation of the Visual Field Extent can also be influenced by the sudden appearance of salient elements, such as Visual Mental Imagery (entities) arising within the Visual Field boundaries. Such emergent imagery may bias visual attention and redirect the Self-Location orientation accordingly.

For instance, in Subject 21's graphic representation of C5 (Supplementary material, *Graphic Representations*, p. 101), the entity appears at approximately the 1 o'clock position within the encompassing ellipsoidal Visual Field boundaries. In the subsequent representation of space B2 (Supplementary material, *Graphic Representations*, p. 102), only the elliptic arc of the Visual Field boundary onto which the entity is positioned is preserved. This selective attentional focus is further illustrated in space C3 (Supplementary material, *Graphic Representations*, p. 103), where the Self-Location moves closer to the Visual Field boundary at the location where the entity appears. This produces a first type of transformation, characterized by a change in size: the Visual Field boundary expands, forming a broader elliptical arc and a greater Visual Field Extent. A second type of transformation is observed in space C1 (Supplementary material, *Graphic Representations*, p. 104), where both the entity and the Visual Field boundary shift to the opposite side of the representation. This reflects a change in orientation, suggesting that C1 is the mirror image of C3.

Finally, in the subsequent space, the C5 ellipsoidal Visual Field complete enclosing boundary appears, but the entity is no longer depicted (Supplementary material, *Graphic Representations*, p. 105).

These empirical observations suggest that attentional saliency and Self-Location orientation may jointly modulate the perceived orientation and size of the elliptic Visual Field arcs (B1–B12, C1–C4).

As a qualitative impression from the graphic reconstructions, rather than a systematically coded variable, type A Visual Field boundaries, defined by conical geometries, appeared to show a directional organization characterized by a distinct front–back axis, whereas the elliptic Visual Field arcs (B1–B12, C1–C4) and the C5 ellipsoidal Visual Field boundary appeared comparatively more stable relative to the surrounding experiential space.

Having detailed the chromatic richness of type B Visual Field spaces, we now turn to the type C Visual Field category. Compared to B shapes, which were overwhelmingly chromatic (87.1%) and rarely achromatic (12.9%), C shapes (C1–4, C5) showed lower chromatic proportions (65.0% for C1–4 shapes and 58.6% for C5 shapes) and higher achromatic reports (35.0% for C1–4 shapes and 41.4% for C5 shapes). Descriptively, C1–4 and C5 were more often chromatic than type A, but statistical evidence for differences between A and C1–4 and between A and C5 varied according to the analytic method used.

Some participants' descriptions of C1–4 spaces included vivid references to bright or achromatic light, as illustrated by the following examples: “I was in an infinite space, transparent space, facing a brilliant source of light.” (Supplementary Data, Subject 36, Space 1, C2, p. 16; Supplementary material, *Graphic Representations*, p. 157); “…nobody, space was dark (back, left) and turning lighter (in front, right) turning lighter toward shape C5 (the light) light was a round circle, drifting toward the light drawn toward it like a magnet…when I got closer it was turning more a white light, the light was opaque, unified, circular” (Supplementary Data, Subject 30, Space 1, C2, p. 15; Supplementary material, *Graphic Representations*, p. 139); “The light was it was a bright sunny day with love. I felt contained and embraced in the space…The light was pulsating and sparkling. It was retracting and expanding. It would pulsate randomly when talking. The tempo of the light was almost at the speed of a heartbeat.” (Supplementary Data, Subject 31, Space 1, C2, p. 15; Supplementary material, *Graphic Representations*, p. 142). While some C1–C4 descriptions included bright achromatic or near-achromatic light, the category as a whole included both chromatic and achromatic spaces. C5 emerged as a distinct geometric category characterized by a complete ellipsoidal Visual Field boundary, marking a transition from partial arc segments to a full enclosing visuo-spatial configuration.

#### 4.4.3 Categories of ellipsoidal Visual Field boundaries: C5

C5 is the only complete ellipsoidal Visual Field boundary, unlike the partial arcs of B1–12 and C1–4. In our dataset, 17 of 29 spaces (58.6%) were chromatic and 12 (41.4%) were achromatic, showing a lower chromatic proportion than type B spaces. Although its chromatic–achromatic proportions resemble those of C1–4 (Figure 4), C5 differs through its complete ellipsoidal geometry.

Some participants described C5 in terms of a 360° enclosure of light that completely surrounded them, as illustrated by the following accounts: “I was in the center of an immense bubble of light…it enveloped me completely, I can say that I was part of it…it was at the perfect temperature…” (Supplementary Data, Subject 5, Space 2, C5, p. 5). Another participant described C5 as “pure white divine light behind the dark corridor, like behind a door; that room was like opening the sun” (Supplementary Data, Subject 1, Space 3, C5, p. 3).

For Subject 40, C5 appeared as a state of ontological–affective fusion, echoing descriptions of ego dissolution and phenomenological unity reported in altered states of consciousness (Millière, 2017; Fort et al., 2025): One massive entity, brighter than anything I've ever experienced…I was connected…in total unity with it and everything else…It was like the Beatles line, “Limitless undying love, which shines around me like a million suns and draws me on and on across the Universe.” “It was achromatic and very physical.” (Supplementary Data, Subject 40, Space 1, C5, p. 17).

Phenomenologically, the sense of immersion in C5 resembles aspects of the Ganzfeld phenomenon, in which the visual field becomes perceptually homogeneous or uniform. Some of the illustrative C5 accounts described a space entirely filled with light and associated with a reduced distinction between the self and the surrounding experiential space. Consistent with Haun and Tononi (2019), conscious spatial experience may persist as an “extended canvas” even when perceptual content is empty or uniform. Although purely descriptive and retrospective, these reports may also phenomenologically resemble experiences of ego dissolution. Whether the C5 configuration could serve as a visual marker associated with such experiences remains an open question for future research, particularly in light of recent models of ego dissolution and self-boundary alteration (Stoliker et al., 2022).

## 5 Conclusion

This study proposes a structured phenomenological framework for describing recurrent Visual Field boundary configurations reported during Near-Death Experiences, based on participants' graphic reconstructions and written descriptions. Four principal categories were identified: conical (A1–12), elliptic arc (B1–12), low-curvature elliptic arc (C1–4), and ellipsoidal (C5). These recurrent configurations were associated with differences in Visual Field boundary organization and perceptual chromaticity. In particular, type A Visual Fields were predominantly achromatic, whereas type B Visual Fields were predominantly chromatic. C1–4 and C5 showed more mixed chromatic–achromatic distributions. Across the statistical analyses, the most consistent chromaticity contrast was between A and B; evidence for differences between A and C1–4 and between A and C5 was sensitive to analytic method, while B, C1–4, and C5 were not significantly different from one another in the pairwise Fisher tests.

The sequential organization of participants' reconstructions further suggested recurrent transitions between directional conical configurations and progressively expanded elliptic or ellipsoidal Visual Field organizations. In several cases, type B and type C configurations appeared phenomenologically related to partial segments or transformations of the enclosing C5 ellipsoidal structure. These observations suggest that recurrent forms of visuo-spatial organization may emerge across participants' reports, while remaining compatible with substantial interindividual variability.

Taken together, these findings suggest that some perceptual qualities observed across the identified Visual Field configurations show descriptive similarities with alterations of Visual Field organization reported in perceptual and neurological research, including constricted or partially restricted visual fields. However, the present findings remain descriptive, phenomenological, and retrospective in nature. Accordingly, the proposed framework should be understood as an exploratory methodological approach for systematically describing, reconstructing, and comparing recurrent visuo-spatial characteristics reported across NDEs, rather than as evidence for universal geometric invariants.

The present study has several methodological limitations. Although the pictogram catalog was derived empirically from earlier unconstrained freehand reconstructions rather than from theoretical assumptions, presenting participants with a standardized set of pictograms may nevertheless have influenced their subsequent reconstructions. Likewise, the researcher-assisted procedure may have introduced subtle forms of guidance, despite efforts to restrict interactions to procedural clarification. In addition, the researcher conducting the sessions was not blinded to the study hypotheses. The Google Meet sessions were not audio- or video-recorded; consequently, the elicitation process cannot be retrospectively checked or independently reviewed beyond the retained questionnaire responses and graphic reconstructions. The substantial and highly variable retrospective interval between the reported NDE and study participation may also have affected reconstruction fidelity. The differing instructions for the OBE and NDE graphic reconstruction tasks, particularly the explicit marking of physical-body location in the OBE task, should also be considered when interpreting differences in bodily representation between these reconstruction types. These methodological features should be considered when interpreting the recurrent visual-field patterns identified in participants' reconstructions, which should be regarded as exploratory rather than as independently validated or universal geometric configurations. Whether this geometric framework generalizes to independent NDE samples, other populations, or other altered states of consciousness remains to be established through replication. The archived final drawings were not independently classified by a second rater; consequently, the extent to which the A, B, and C Visual Field families can be reliably recovered from the finished drawings independently of participants' original pictogram selections was not assessed. A further limitation is that the present study did not include a comparison condition in which another altered or vividly recalled experiential state was reconstructed using the same protocol; consequently, the current data cannot determine whether the recurrent Visual Field configurations observed here are specific to NDEs or may also arise when the same reconstruction procedure is applied to other experiential states. Future work should therefore include comparison experiential states reconstructed using the same protocol, as well as comparisons with fully unconstrained freehand reconstruction procedures and prospectively standardized or independently administered reconstruction protocols, in order to distinguish possible state-specific effects from effects of pictorial cueing and researcher-assisted elicitation.

Prospectively, the present reconstruction framework is currently being extended through the development of the Mapping Interface for Altered States of Consciousness (MIST), a three-dimensional interactive reconstruction and mapping environment. This extension will broaden the present framework beyond Near-Death Experiences to encompass other non-ordinary states of consciousness, including meditative and drug-induced states, thereby enabling systematic comparisons of their visuo-spatial organization. Unlike the present protocol, which relies on two-dimensional graphic representations, MIST will enable participants to construct and continuously modify their experienced spaces directly in three dimensions. The platform is being designed to support continuous spatial reconstruction and precise XYZ encoding of orientation, curvature, dimensionality, Self-Location, and Self-Motion, thereby providing a more flexible and quantitative framework for reconstructing, analyzing, and comparing visuo-spatial organization across non-ordinary states of consciousness.

## Data Availability

The original contributions presented in the study are included in the article/Supplementary material, further inquiries can be directed to the corresponding author.

## References

[B1] Albertazzi L. (2013). Handbook of Experimental Phenomenology: Visual Perception of Shape, Space and Appearance. Hoboken, NJ: John Wiley & Sons. doi: 10.1002/9781118329016

[B2] Albertazzi L. Van Tonder G. J. Vishwanath D. (2011). Perception Beyond Inference: The Information Content of Visual Processes. Boston, MA: MIT Press. doi: 10.7551/mitpress/8594.001.0001

[B3] Barrett W. (1926/1986). Death-Bed Visions: The Psychical Experiences of the Dying. Wellingborough: Aquarian Press.

[B4] Barsalou L. W. (1999). Perceptual symbol systems. Behav. Brain Sci. 22, 577–660. doi: 10.1017/S0140525X99002149 11301525

[B5] Belanti J. Perera M. Jagadheesan K. (2008). Phenomenology of near-death experiences: a cross-cultural perspective. Transcult. Psychiatry 45, 121–133. doi: 10.1177/1363461507088001 18344255

[B6] Berthoz A. (1997). Le sens du mouvement. Paris: Odile Jacob.

[B7] Berthoz A. (2000). The Brain's Sense of Movement. Cambridge, MA: Harvard University Press.

[B8] Blackmore S. J. (1984). A postal survey of obes and other experiences. J. Soc. Psych. Res. 52, 225–244.

[B9] Blackmore S. J. (1993). Near-death experiences in india: they have tunnels too. J. Near-Death Stud. 11, 205–217. doi: 10.17514/JNDS-1993-11-4-p205-217

[B10] Blackmore S. J. Troscianko T. S. (1989). The physiology of the tunnel. J. Near-Death Stud. 8, 15–28. doi: 10.17514/JNDS-1989-8-1-p15-28

[B11] Blanke O. (2004). Out-of-body experiences and their neural basis. BMJ 329, 1414–1415. doi: 10.1136/bmj.329.7480.1414 PMC535951 15604159

[B12] Blanke O. (2012). Multisensory brain mechanisms of bodily self-consciousness. Nat. Rev. Neurosci. 13, 556–571. doi: 10.1038/nrn3292 22805909

[B13] Blanke O. Arzy S. (2005). The out-of-body experience: disturbed self-processing at the temporo-parietal junction. Neuroscientist 11, 16–24. doi: 10.1177/1073858404270885 15632275

[B14] Blanke O. Faivre N. Dieguez S. (2016). “Leaving body and life behind: out-of-body and near-death experience," in The Neurology of Consciousness, eds. S. Laureys, O. Gosseries, and G. Tononi (Amsterdam: Elsevier), 323–347. doi: 10.1016/B978-0-12-800948-2.00020-0

[B15] Blanke O. Ortigue S. Landis T. Seeck M. (2002). Stimulating illusory own-body perceptions. Nature 419, 269–270. doi: 10.1038/419269a 12239558

[B16] Brandt T. Dichgans J. Koenig E. (1973). Differential effects of central versus peripheral vision on egocentric and exocentric motion perception. Exp. Brain Res. 16, 476–491. doi: 10.1007/BF00234474 4695777

[B17] Britton W. B. Bootzin R. R. (2004). Near-death experiences and the temporal lobe. Psychol. Sci. 15, 254–258. doi: 10.1111/j.0956-7976.2004.00661.x 15043643

[B18] Edmeads J. G. (2003). What is causing “tunnel vision”? Postgrad. Med. 113, 4–4. doi: 10.3810/pgm.2003.03.1393 12661547

[B19] Egger V. (1896). Le moi des mourants. Rev. Philos. Fr. Etrang. 41, 26–38.

[B20] Facco E. Agrillo C. (2012a). Near-death experiences between science and prejudice. Front. Hum. Neurosci. 6:209. doi: 10.3389/fnhum.2012.00209 PMC3399124 22826697

[B21] Facco E. Agrillo C. (2012b). Near-death-like experiences without life-threatening conditions or brain disorders: a hypothesis from a case report. Front. Psychol. 3:490. doi: 10.3389/fpsyg.2012.00490 23162522 PMC3498963

[B22] Finke R. A. Kurtzman H. S. (1981). Mapping the visual field in mental imagery. J. Exp. Psychol. 110:501. doi: 10.1037//0096-3445.110.4.501 6459405

[B23] Fleming V. (Director). (1939). The Wizard of Oz [Film]. Metro-Goldwyn-Mayer.

[B24] Fort L. D. Costines C. Wittmann M. Demertzi A. Schmidt T. T. (2025). Classification schemes of altered states of consciousness. Neurosci. Biobehav. Rev. 175:106178. doi: 10.1016/j.neubiorev.2025.106178 40294668

[B25] Gibson J. J. (2014). The Ecological Approach to Visual Perception. Hove: Psychology Press. doi: 10.4324/9781315740218

[B26] Greyson B. (1983). The near-death experience scale: construction, reliability, and validity. J. Nerv. Ment. Dis. 171, 369–375. doi: 10.1097/00005053-198306000-00007 6854303

[B27] Greyson B. (2014). “Near-death experiences.” in Varieties of Anomalous Experience: Examining the Scientific Evidence, 2nd edn, eds E. Cardeña, S. J. Lynn, and S. Krippner (Washington, DC: American Psychological Association), 333–367. doi: 10.1037/14258-01242185006

[B28] Greyson B. (2015). Western scientific approaches to near-death experiences. Humanities 4, 775–796. doi: 10.3390/h4040775

[B29] Greyson B. Flynn C. P. (1984). The Near-Death Experience: Problems, Prospects, Perspectives. Springfield, IL: Charles C Thomas.

[B30] Greyson B. Kelly E. W. Kelly E. F. (2009). “Explanatory models for near-death experiences," in The Handbook of Near-Death Experiences: Thirty Years of Investigation, eds J. M. Holden, B. Greyson, and D. James (Santa Barbara, CA: Praeger), 213–234. doi: 10.5040/9798400661389.ch-010

[B31] Grove E. J. Hewitt T. Seth A. K. Macpherson F. Schwartzman D. J. (2026). A large-scale computer-vision mapping of the geometric structures of stroboscopically-induced visual hallucinations. bioRxiv. [preprint]. doi: 10.64898/2026.02.18.705710

[B32] Hansen T. Pracejus L. Gegenfurtner K. R. (2009). Color perception in the intermediate periphery of the visual field. J. Vis. 9:26. doi: 10.1167/9.4.26 19757935

[B33] Haun A. Tononi G. (2019). Why does space feel the way it does? Towards a principled account of spatial experience. Entropy 21:1160. doi: 10.3390/e21121160

[B34] Haun A. Tononi G. (2025). The unfathomable richness of seeing. Trends Cogn. Sci. 29, 892–902. doi: 10.1016/j.tics.2025.06.001 40615267

[B35] Heim A. (1892). Notizen über den Tod durch Absturz. Jahrb. Schweiz. Alpenclub 27, 327–337.

[B36] Hou Y. Huang Q. Prakash R. Chaudhury S. (2013). Infrequent near death experiences in severe brain injury survivors-a quantitative and qualitative study. Ann. Indian Acad. Neurol. 16, 75–81. doi: 10.4103/0972-2327.107715 23661968 PMC3644787

[B37] Husserl E. (1982). Ideas Pertaining to a Pure Phenomenology and to a Phenomenological Philosophy, First Book: General Introduction to a Pure Phenomenology. The Hague: Martinus Nijhoff. Translated by F. Kersten. (Original work published 1913).

[B38] Kennedy M. (1997). Art therapy and the near death experience: A spiritual journey of transformation (Doctoral dissertation). Mount Mary College.

[B39] Klemenc-Ketis Z. (2013). Life changes in patients after out-of-hospital cardiac arrest: the effect of near-death experiences. Int. J. Behav. Med. 20, 7–12. doi: 10.1007/s12529-011-9209-y 22161220

[B40] Kondziella D. Dreier J. P. Olsen M. H. (2019). Prevalence of near-death experiences in people with and without rem sleep intrusion. PeerJ 7:e7585. doi: 10.7717/peerj.7585 31523519 PMC6716500

[B41] Lambert E. H. Wood E. H. (1946). The problem of blackout and unconsciousness in aviators. Med. Clin. N Am. 30, 833–844. doi: 10.1016/S0025-7125(16)35922-3 20992942

[B42] Lange R. Greyson B. Houran J. (2004). A rasch scaling validation of a ‘core'near-death experience. Br. J. Psychol. 95, 161–177. doi: 10.1348/000712604773952403 15142300

[B43] León A. (2013). “Near-death experiences throughout the history of art and science: challenges for the XXI century,” in Death, Dying, Culture: An Interdisciplinary Interrogation, eds. J. Steffen, and N. Hinerman (Brill), (Oxford: Inter-Disciplinary Press), 205–215. doi: 10.1163/9781848881730_020

[B44] Lerner F. (2016). Matière des déplacements des états de mort imminente (e-motion, création, réparation) (PhD thesis), UCL-Université Catholique de Louvain.

[B45] Lerner F. Laureys S. Charland-Verville V. Botbol Baum M. (2021). Self-location kinematics influence the generation of near-death experience cognitive and affective perceptions. J. Near-Death Stud. 39, 3–51. doi: 10.17514/JNDS-2021-39-1-p3-51

[B46] Livingstone M. S. Hubel D. H. (1987). Psychophysical evidence for separate channels for the perception of form, color, movement, and depth. J. Neurosci. 7, 3416–3468. doi: 10.1523/JNEUROSCI.07-11-03416.1987 3316524 PMC6569044

[B47] Lucci G. Pazzaglia M. (2015). Towards multiple interactions of inner and outer sensations in corporeal awareness. Front. Hum. Neurosci. 9:163. doi: 10.3389/fnhum.2015.00163 25883564 PMC4381648

[B48] Madary M. (2016). Visual Phenomenology. Cambridge, MA: MIT Press. Available online at http://www.jstor.org/stable/j.ctt1jktr2s (Accessed October 15, 2025).

[B49] Mancl L. A. DeRouen T. A. (2001). A covariance estimator for gee with improved small-sample properties. Biometrics 57, 126–134. doi: 10.1111/j.0006-341X.2001.00126.x 11252587

[B50] Martial C. Cassol H. Charland-Verville V. Pallavicini C. Sanz C. Zamberlan F. . (2019a). Neurochemical models of near-death experiences: a large-scale study based on the semantic similarity of written reports. Conscious. Cogn. 69, 52–69. doi: 10.1016/j.concog.2019.01.011 30711788

[B51] Martial C. Mensen A. Charland-Verville V. Vanhaudenhuyse A. Rentmeister D. Bahri M. A. . (2019b). Neurophenomenology of near-death experience memory in hypnotic recall: a within-subject eeg study. Sci. Rep. 9:14047. doi: 10.1038/s41598-019-50601-6 31575924 PMC6773844

[B52] Martial C. Simon J. Puttaert N. Gosseries O. Charland-Verville V. Nyssen A.-S. . (2020). The near-death experience content (NDE-C) scale: development and psychometric validation. Conscious. Cogn. 86:103049. doi: 10.1016/j.concog.2020.103049 33227590

[B53] Millière R. (2017). Looking for the self: phenomenology, neurophysiology and philosophical significance of drug-induced ego dissolution. Front. Hum. Neurosci. 11:245. doi: 10.3389/fnhum.2017.00245 28588463 PMC5441112

[B54] Mobbs D. Watt C. (2011). There is nothing paranormal about near-death experiences: how neuroscience can explain seeing bright lights, meeting the dead, or being convinced you are one of them. Trends Cogn. Sci. 15, 447–449. doi: 10.1016/j.tics.2011.07.010 21852181

[B55] Moody R. A. (1978). Reflections on Life After Life. New York, NY: Bantam Books. doi: 10.1177/004057367803500208

[B56] Moore L. E. Greyson B. (2017). Characteristics of memories for near-death experiences. Conscious. Cogn. 51, 116–124. doi: 10.1016/j.concog.2017.03.003 28329717

[B57] Morse M. L. Venecia Jr D. Milstein J. (1989). Near-death experiences: a neurophysiologic explanatory model. J. Near-Death Stud. 8, 45–53. doi: 10.1007/BF01076138

[B58] Murray C. D. Fox J. (2005). Dissociational body experiences: differences between respondents with and without prior out-of-body-experiences. Br. J. Psychol. 96, 441–456. doi: 10.1348/000712605X49169 16248935

[B59] Owens J. Cook E. W. Stevenson I. (1990). Features of “near-death experience” in relation to whether or not patients were near death. Lancet 336, 1175–1177. doi: 10.1016/0140-6736(90)92780-L 1978037

[B60] Palmieri A. Calvo V. Kleinbub J. R. Meconi F. Marangoni M. Barilaro P. . (2014). “Reality" of near-death-experience memories: evidence from a psychodynamic and electrophysiological integrated study. Front. Hum. Neurosci. 8:429. doi: 10.3389/fnhum.2014.00429 24994974 PMC4063168

[B61] Parnia S. Fenwick P. (2002). Near death experiences in cardiac arrest: visions of a dying brain or visions of a new science of consciousness. Resuscitation 52, 5–11. doi: 10.1016/S0300-9572(01)00469-5 11801343

[B62] Parnia S. Spearpoint K. De Vos G. Fenwick P. Goldberg D. Yang J. . (2014). Aware—awareness during resuscitation—a prospective study. Resuscitation 85, 1799–1805. doi: 10.1016/j.resuscitation.2014.09.004 25301715

[B63] Parnia S. Waller D. G. Yeates R. Fenwick P. (2001). A qualitative and quantitative study of the incidence, features and aetiology of near death experiences in cardiac arrest survivors. Resuscitation 48, 149–156. doi: 10.1016/S0300-9572(00)00328-2 11426476

[B64] Pehlivanova M. Lange R. Greyson B. Houran J. (2026). Operationalizing near-death experiences: stability of the NDE rasch hierarchy over two decades. Conscious. Cogn. 139:103979. doi: 10.1016/j.concog.2025.103979 41554189

[B65] Ring K. (1980). Life At Death: A Scientific Investigation of the Near-Death Experience. New York, NY: Coward, McCann & Geoghegan.

[B66] Rominger R. A. (2004). Exploring the integration of the aftereffects of the near-death experience: An intuitive and artistic inquiry (Doctoral dissertation), Institute of Transpersonal Psychology.

[B67] Saavedra-Aguilar J. C. Gómez-Jeria J. S. (1989). A neurobiological model for near-death experiences. J. Near-Death Stud. 7, 205–222. doi: 10.17514/JNDS-1989-7-4-p205-222

[B68] Sabom M. B. (1982). Recollections of Death: A Medical Investigation. New York, NY: Harper & Row.

[B69] Schumacher J. (Director). (1990). Flatliners [Film]. Columbia Pictures.

[B70] Schwaninger J. Eisenberg P. R. Schechtman K. B. Weiss A. N. (2002). A prospective analysis of near-death experiences in cardiac arrest patients. J. Near-Death Stud. 20, 215–232. doi: 10.17514/JNDS-2002-20-4-p215-232

[B71] Spagna A. Heidenry Z. Miselevich M. Lambert C. Eisenstadt B. E. Tremblay L. . (2024). Visual mental imagery: evidence for a heterarchical neural architecture. Phys. Life Rev. 48, 113–131. doi: 10.1016/j.plrev.2023.12.012 38217888

[B72] Stoliker D. Egan G. F. Friston K. J. Razi A. (2022). Neural mechanisms and psychology of psychedelic ego dissolution. Pharmacol. Rev. 74, 876–917. doi: 10.1124/pharmrev.121.000508 36786290

[B73] Tang P. K. (2019). Art at the intersection of near-death experience. Lancet Neurol. 18:33. doi: 10.1016/S1474-4422(18)30438-1

[B74] Thompson E. (2010). Mind in Life: Biology, Phenomenology, and the Sciences of Mind. Cambridge, MA: Harvard University Press. Available online at https://www.hup.harvard.edu/books/9780674057517 (Accessed September 29, 2026).

[B75] Thonnard M. Charland-Verville V. Brédart S. Dehon H. Ledoux D. Laureys S. . (2013). Characteristics of near-death experiences memories as compared to real and imagined events memories. PLoS ONE 8:e57620. doi: 10.1371/journal.pone.0057620 23544039 PMC3609762

[B76] Trauzettel-Klosinski S. (2010). Rehabilitation for visual disorders. J. Neuro-Ophthalmol. 30, 73–84. doi: 10.1097/WNO.0b013e3181ce7e8f 20182214

[B77] Trumbull D. (Director). (1983). Brainstorm [Film]. JF Productions.

[B78] Tversky B. (2013). “Visualizing thought,” in Handbook of Human Centric Visualization, ed W. Huang (New York, NY: Springer), 3–40. doi: 10.1007/978-1-4614-7485-2_1

[B79] Van Lommel P. Van Wees R. Meyers V. Elfferich I. (2001). Near-death experience in survivors of cardiac arrest: a prospective study in the netherlands. Lancet 358, 2039–2045. doi: 10.1016/S0140-6736(01)07100-8 11755611

[B80] Vanhaudenhuyse A. Thonnard M. Laureys S. (2009). “Towards a neuro-scientific explanation of near-death experiences?,” in Yearbook of Intensive Care and Emergency Medicine, ed. J.-L. Vincent (Berlin, Heidelberg: Springer), 961–968. doi: 10.1007/978-3-540-92276-6_85

[B81] Weibull W. (1939). A statistical theory of the strength of materials. Ingeniörsvetenskapsakademiens Handlingar, 151, 1–45.

[B82] Zaleski C. (1987). Otherworld Journeys: Accounts of Near-Death Experience in Medieval and Modern Times. Oxford: Oxford University Press.

[B83] Zele A. J. Cao D. (2014). Vision under mesopic and scotopic illumination. Front. Psychol. 5:1594. doi: 10.3389/fpsyg.2014.01594 25657632 PMC4302711

