# Supplementary material for: The perceptual geometry and chromaticity of near-death experience spaces
Source: Front Psychol. 2026 Oct 15;17:1760389. doi: 10.3389/fpsyg.2026.1760389 (PMC13716671; doi:10.3389/fpsyg.2026.1760389)
Supplement: Supplementary file 1 [file Data_Sheet_1.pdf]

Catalogue of NDE Space Pictograms & Lexicon and Legend Symbol

Pictograms A, B, C:

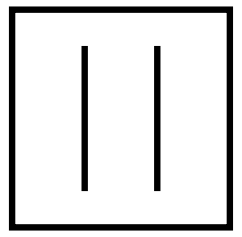

A1

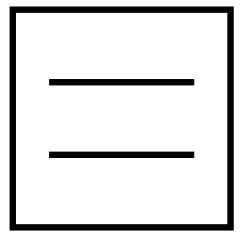

A2

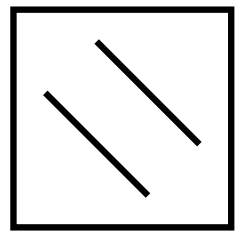

A3

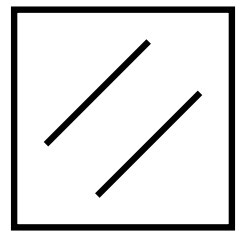

A4

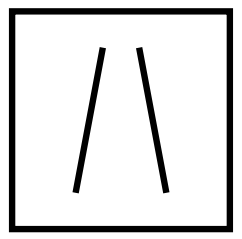

A5

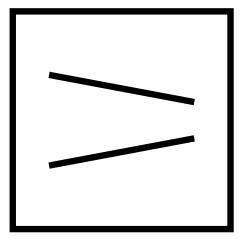

A6

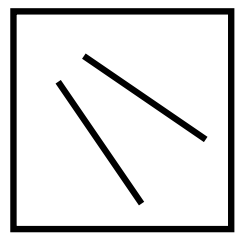

A7

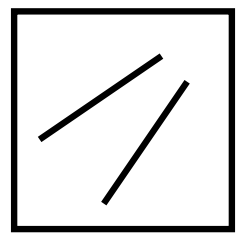

A8

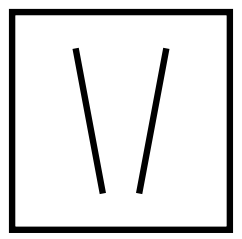

A9

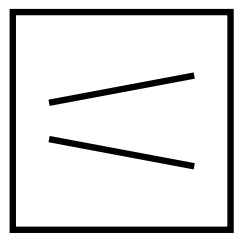

A10

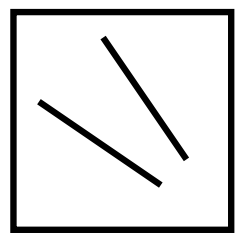

A11

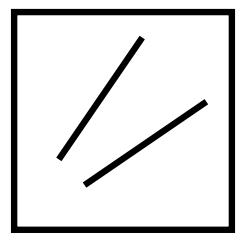

A12

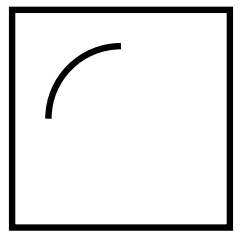

B1

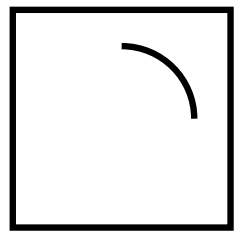

B2

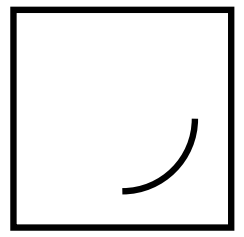

B3

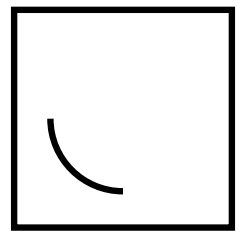

B4

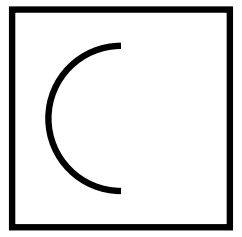

B5

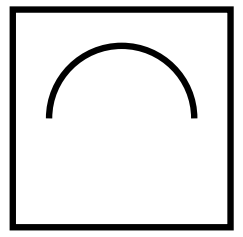

B6

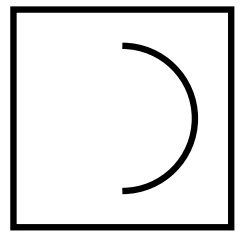

B7

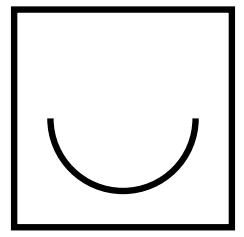

B8

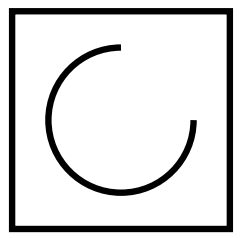

B9

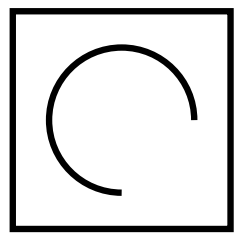

B10

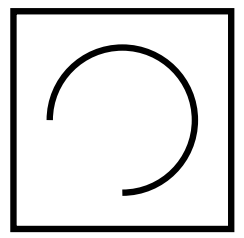

B11

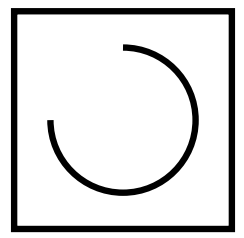

B12

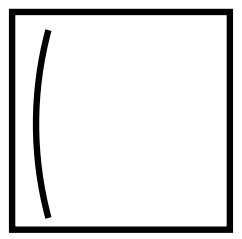

C1

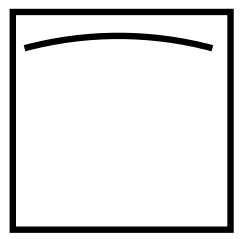

C2

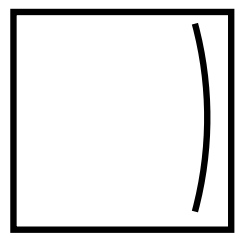

C3

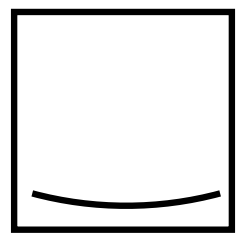

C4

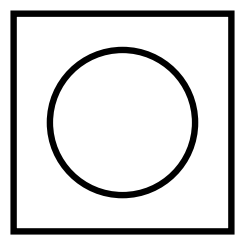

C5

Exceptions:

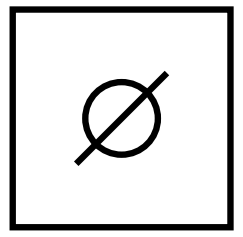

No space

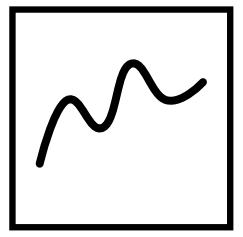

Other space (freely hand-drawn)

Legend Symbols:

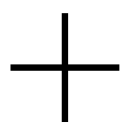

Self-Location

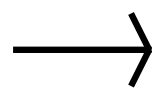

Self-Motion Direction

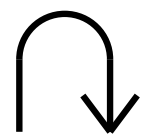

Self-Motion U-Turn

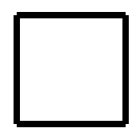

Entities/Beings

Lexicon:

Near-Death Experience (NDE): A **Near-Death Experience (NDE)** is a profound transcendental mental experience that typically occurs following a life-threatening situation (e.g., cardiac arrest, coma, traumatic brain injury). However, NDEs can also arise in circumstances that do not jeopardize life, such as during meditation or migraines.

Out-of-Body Experience (OBE): An **Out-of-Body Experience (OBE)** is a phenomenon in which an individual perceives the physical world and/or their own physical body from an extracorporeal point of view. This experience is accompanied by the impression of separating from or departing from the physical body and/or the physical world, followed by a sense of reuniting with or returning to the physical body and/or the physical world.

Types of Out-of-Body Experiences (OBEs):

1. **Dissociative Out-of-Body Experience (Dissociative OBE):** A phenomenon in which an individual perceives the physical world and/or their own physical body from an extracorporeal point of view, with the impression of separating from or departing from their physical body and/or the physical world.
2. **Associative Out-of-Body Experience (Associative OBE):** A phenomenon in which an individual perceives the physical world and/or their own physical body from an extracorporeal point of view, with the impression of reuniting with or returning to their physical body and/or the physical world.

Key Concepts Related to NDE and OBE:

- **Physical Body:** The corporeal body that has material existence.
- **Extracorporeal Point of View:** The perspective from which an individual perceives the physical world and/or their physical body while experiencing the sensation of being outside their corporeal body.
- **Extracorporeal Space:** The space from which an individual perceives their surroundings while having the impression of being outside their corporeal body.

Self Mapping in NDE and OBE:

- **Self-Location (S-L):** The place where an individual feels their "self" is located while being in an extracorporeal space and/or experiencing an extracorporeal point of view.
- **Self-Motion (S-M):** The sensation of movement experienced by the self while having the impression of being outside the physical body in an extracorporeal space and/or from an extracorporeal point of view.
- **Self-Motion U-Turn:** A sensation of making a U-turn while outside the physical body in an extracorporeal space and/or from an extracorporeal point of view. In NDEs, this U-turn sensation is often perceived as a turning point where the self, either voluntarily or involuntarily, decides to return to the physical body and/or the physical world.
