# Supplementary material for: The perceptual geometry and chromaticity of near-death experience spaces
Source: Front Psychol. 2026 Oct 15;17:1760389. doi: 10.3389/fpsyg.2026.1760389 (PMC13716671; doi:10.3389/fpsyg.2026.1760389)

EXAMPLE OBE DISSOCIATIVE

Given name initials & Family name or Pseudonym: J. DOE

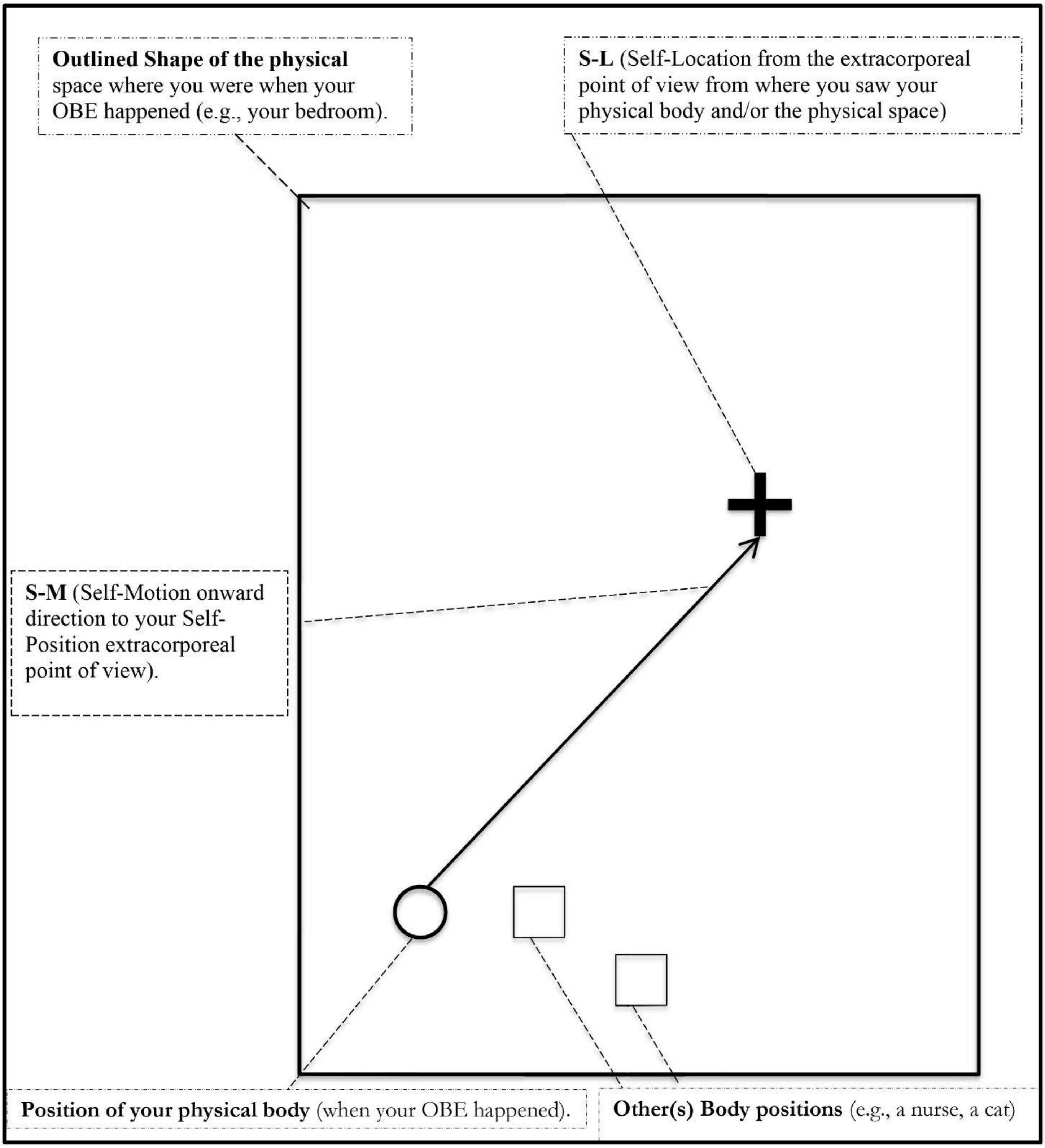

EXAMPLE: TYPE A NDE SPACES SHAPES OUTLINES

Given name initials & Family name or Pseudonym: J. DOE

Part number according to the chronological order of your NDE: 1/5

NDE Code Spaces Shapes Outlines Number (or OTHER): A5

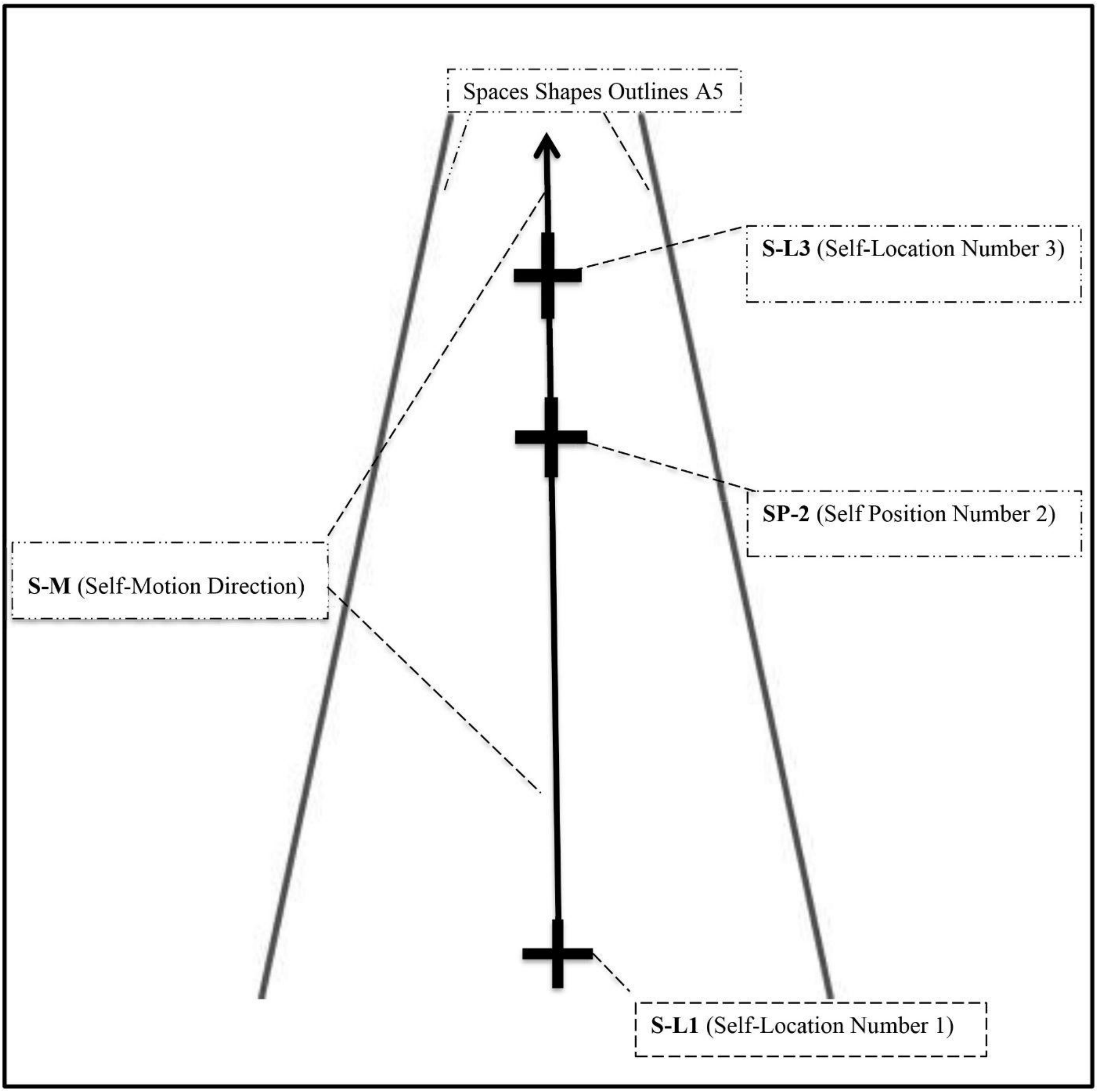

EXAMPLE: TYPE B NDE SPACES SHAPES OUTLINES

Given name initials & Family name or Pseudonym:	J. DOE
Part number according to the chronological order of your NDE:	2/3
NDE Code Spaces Shapes Outlines Number (or OTHER)	B10

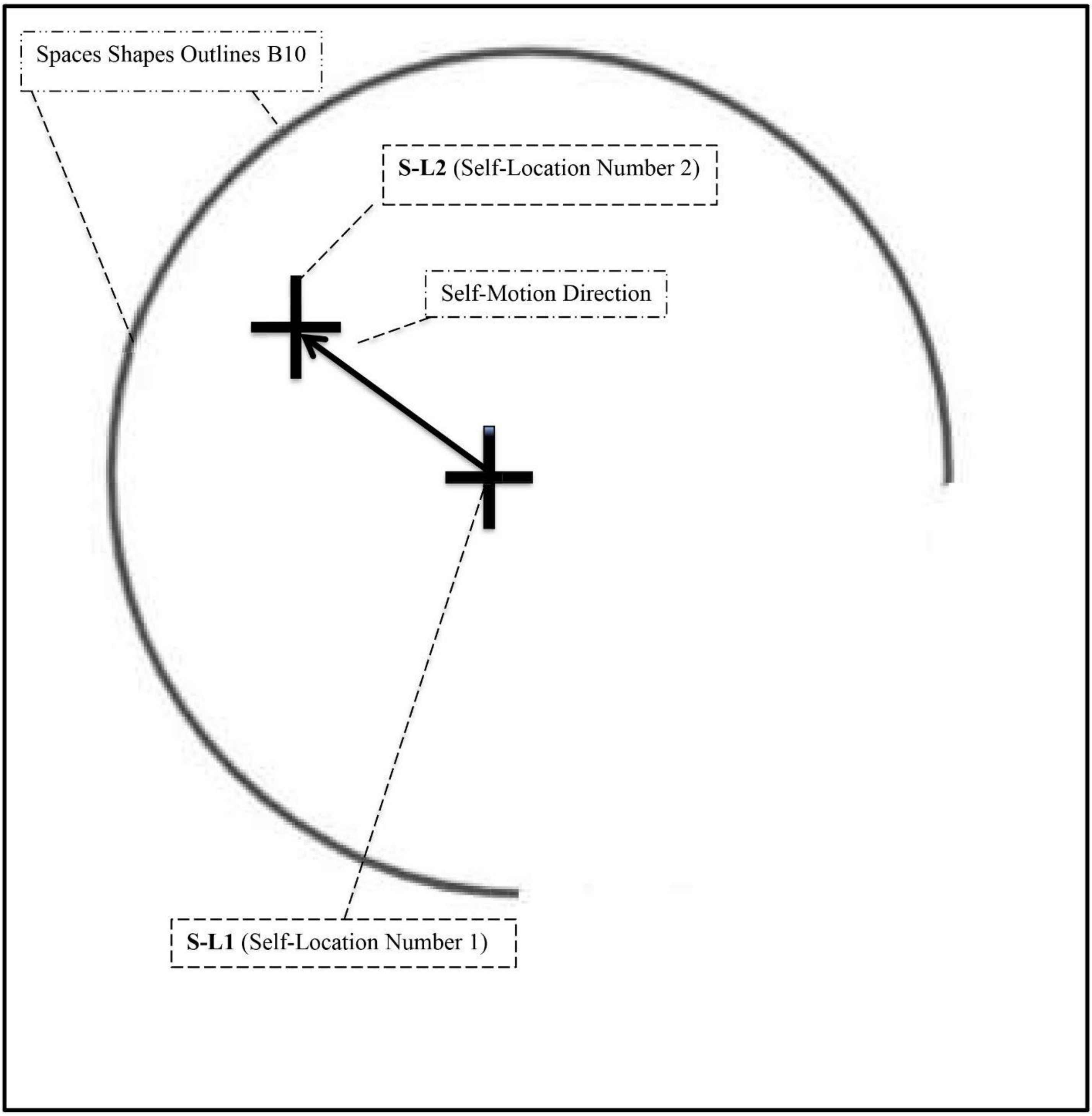

EXAMPLE: TYPE C NDE SPACES SHAPES OUTLINES

Given name initials & Family name or Pseudonym: J. DOE

Part number according to the chronological order of your NDE: 3/3

NDE Code Spaces Shapes Outlines Number (or OTHER): C5

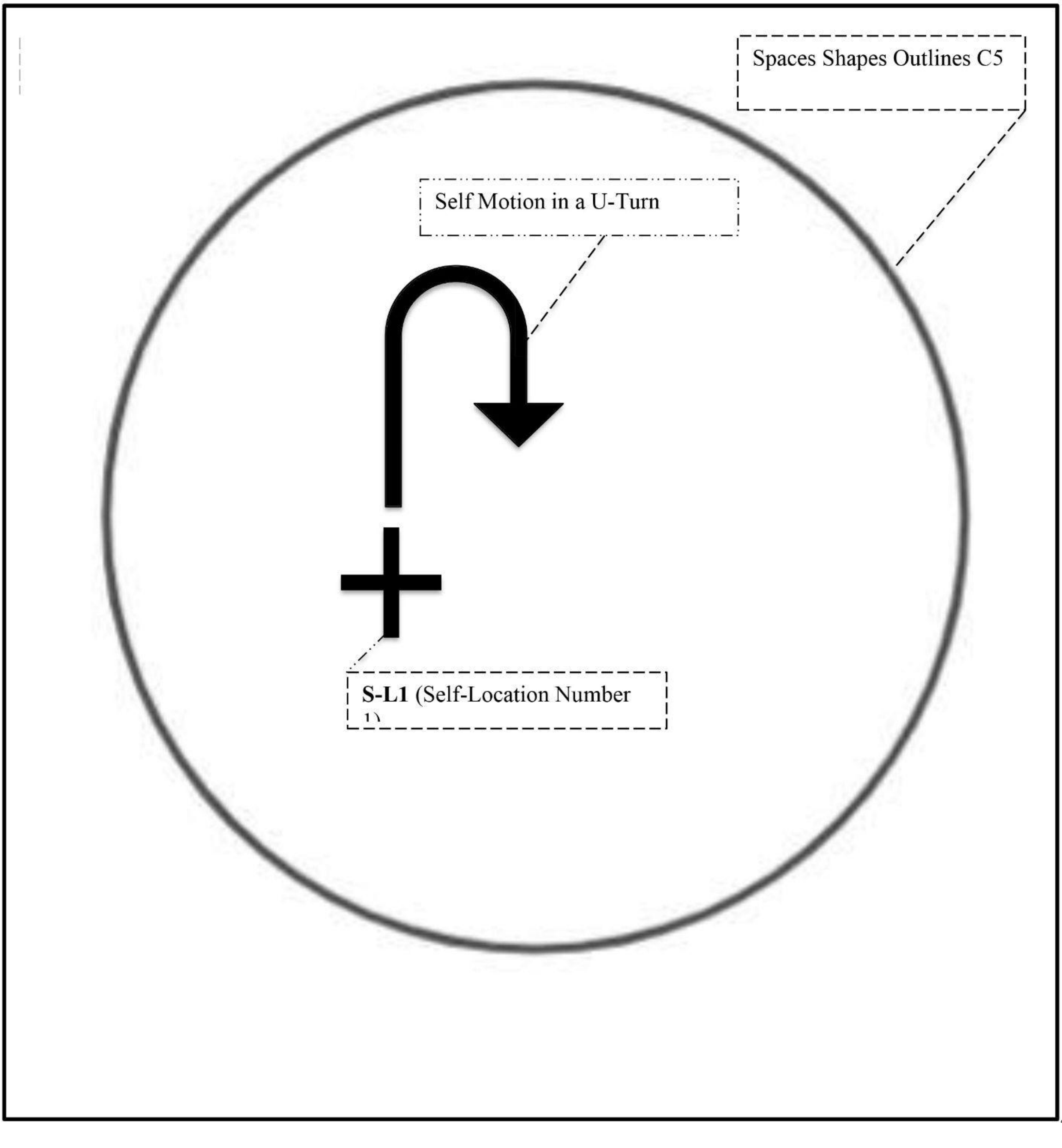

EXAMPLE OBE ASSOCIATIVE

Given name initials & Family name or Pseudonym: J. DOE

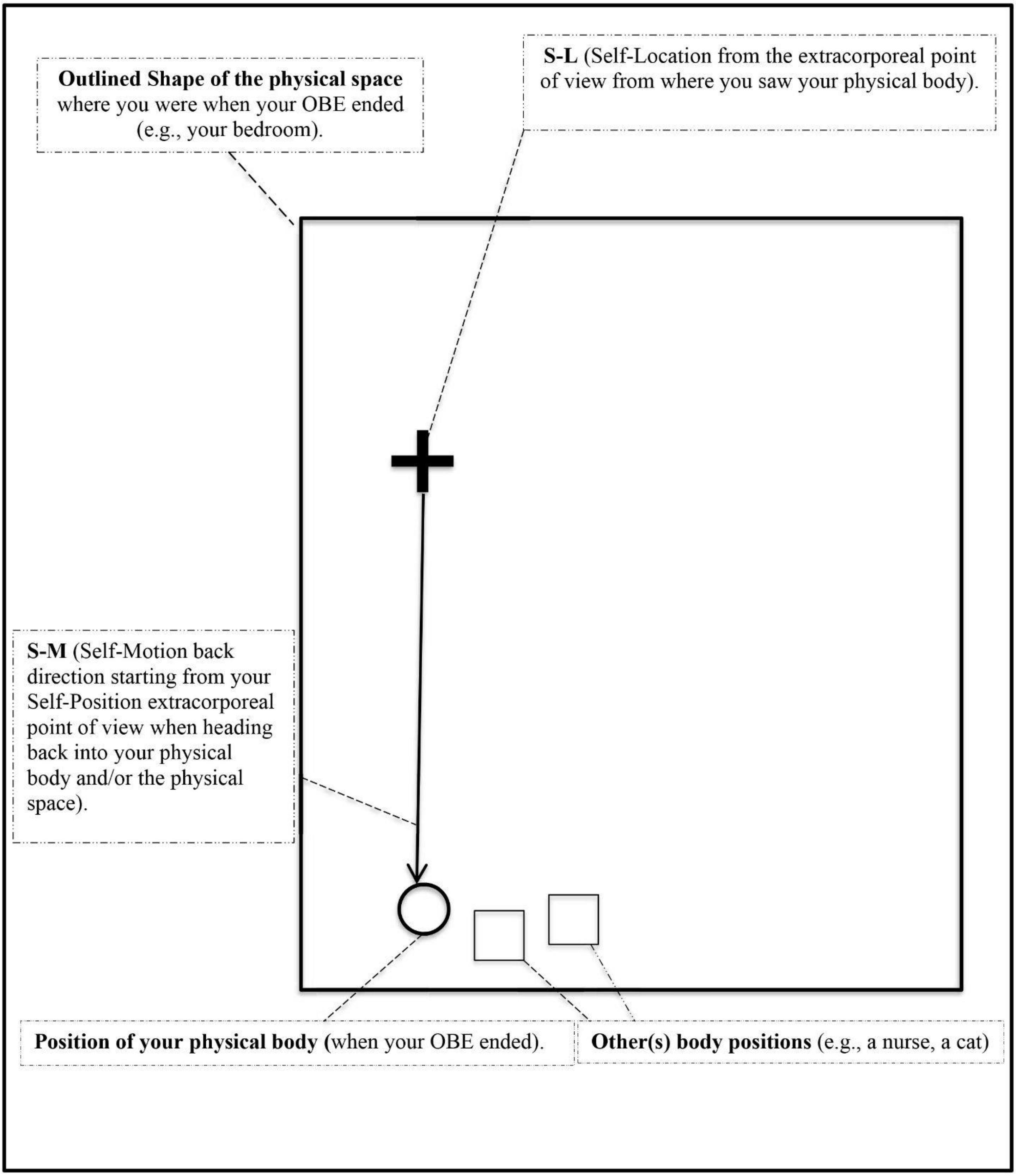

Supplement: Supplementary file 2 [file Data_Sheet_2.pdf]
