# Supplementary material for: The perceptual geometry and chromaticity of near-death experience spaces
Source: Front Psychol. 2026 Oct 15;17:1760389. doi: 10.3389/fpsyg.2026.1760389 (PMC13716671; doi:10.3389/fpsyg.2026.1760389)
Supplement: Supplementary file 3 [file Data_Sheet_3.pdf]

Self-reporting Questionnaire Extracorporeal Affects & Bodily Sensations in Near-Death Experiences Spaces (1-4).

- For your convenience in the filling up of the questionnaire, we suggest you to use a fairly large surface to have your computer and the printed documents on the same plane. This will enable you to answer side by side in parallel the online digital questionnaire and the written graphic part.

* Indique une question obligatoire

1. Adresse e-mail *

2. Family name & Given name (pseudonym optional) *

3. Date of birth *

Exemple : 7 janvier 2019

Before starting to answer the questionnaire please consult the definitions below to understand which type of experience you had.

- **An extracorporeal point of view** is the point of view from which you perceive while having the impression of being outside your physical body.
- **By physical body** we mean the corporeal body which has a material existence.
- **Out-of Body Experiences (OBEs)** are phenomenon where you perceived the physical world and/or your physical body from an extracorporeal point of view (namely a point of view which is located outside you physical body) with the impression of being separating or departing from your physical body or/and the physical world and reuniting or returning to your physical body or/and the physical world.
- **A Dissociative Out-of Body Experience (OBE Dissociative)** is a phenomenon where you perceived the physical world and/or your physical body from an extracorporeal point of view
(namely a point of view which is located outside you physical body) with the impression of being separating or departing from your physical body or/and the physical world.
- **An Associative Out-of Body Experience (OBE Associative)** is a phenomenon where you perceived the physical world and/or your physical body from an extracorporeal point of view

  (namely a point of view which is located outside you physical body) with the impression of reuniting with or returning into your physical body or/and the physical world.
- **Near-Death Experiences (NDEs)** are deep transcendental mental experiences typically following a life-threatening situation (e.g. cardiac arrest, coma, traumatic brain injury) but also associated with circumstances that do not jeopardize life (e.g. meditation, migraine).

Important note: Your Near-Death Experience (NDE) may or may not include an Out-of Body Experience (OBE).

REMEMBERING YOUR OBE & NDE

Take the time you need to recall your OBE and/or NDE. To enhance the recall of your memory, you might want to close your eyes.

1. Precise the OBE type you had according to its matching with the following description:

- For the OBE Dissociative type it will be in the case where you saw from an extracorporeal point of view (namely from outside your physical body) the physical world or/and your physical body with the impression of separating or departing from your physical body or/and the physical world.
- For the OBE Associative type it will be in the case where you saw from an extracorporeal point of view (namely from outside your physical body) the physical world or/and your physical body with the impression of reuniting with or returning into your physical body or/and the physical world.

2. Indicate the number of OBE parts you had:

- An OBE Dissociative is accounted as 1 part.
- An OBE Associative is accounted as 1 part.
- If you had both Dissociative and Associative OBE type you will have 2 OBE parts.
- If you did not have any Dissociative and /or Associative OBE type, you will have 0 OBE part.

3. Divide your NDE into numbered part(s) (starting from 1) using the storytelling mode which is the most appealing for you (eg. movie sequence, book chapter, theater play-act, opera act, music movement). We have set a maximum threshold of 10 parts for the NDE parts. Thus, the part number of your NDE cannot exceed 10 parts).

4. Ordering your documents

- If you had a Dissociative OBE part, its drawing document should be placed on your desk before all your NDE part(s) drawing document(s).
- If you had an Associative OBE part, its drawing document should be placed on your desk after all your NDE part(s) drawing document(s).

- If you had both a Dissociative OBE part and an Associative OBE part, the OBE Dissociative drawing document should be placed before all your NDE part(s) drawing document(s) and the OBE Associative drawing document should be placed after all your NDE part(s) drawing document(s).

Now, please answer the question below:

4. In your total experience, how many parts are OBE parts and how many parts are NDE parts? *
(Eg. if your total experience contains 6 parts from which 2 are OBE parts and 4 are NDE parts you will have to write 6 parts = 2 OBE parts + 4 NDE parts).

PRINTING DOCUMENTS INSTRUCTIONS
Please print the following documents below while being careful to avoid two-sided printing.
- The "NOTICE" (12 pages):
https://drive.google.com/file/d/1gZsaogZ2uSgAdrCXygYVEto_EbHN7odb/view?usp=share_li
- If you had a Dissociative OBE print 1 time the "Dissociative OBE" document:
https://drive.google.com/file/d/1N_5_rT7O8BRtLe4OAkBk9sCRQFX137MV/view?usp=share_link
- If you had an Associative OBE print 1 time the "Associative OBE" document:
https://drive.google.com/file/d/1mPT-oXUfb93pSG JdxmMQGU54g19rHr_/view?usp=share_link
- Print the "NDE" document as many times as the number of NDE part you had:
https://drive.google.com/file/d/1o-HG2sFvWvzcgNzPcp_0SD7pndUexh0L/view?usp=share_link

The total number of printed pages must coincide with the total number of parts of your OBE or/and NDE.
(Eg. if your experience contained 6 parts including 1 OBE Dissociative part + 1 OBE Associative part + 4 NDE parts, you should have printed 6 pages).

ORGANIZING YOUR DRAWING DOCUMENTS
*Important note: to fill-up all the drawing documents please only use a blue pen with a thickness of 1.00 mm and write in capital letters.

Line up all your drawing documents on your table according to the chronological order of your entire OBE or/and NDE.

Then complete each drawing document in this way:
- Write in the first textbox the initial of your given name followed by your family name or if you prefer write your pseudonym.
- Write in the second textbox the NDE part number in the chronological order you have experienced it, eg: if it was the part number 1 of your NDE you should write: 1.

Then follows the total number of NDE part(s) you had, eg: if you have divided you NDE in 3 because in total you have experienced 3 NDE parts you should write: 3.

So, in all you have should written: 1/3.

5. If you had an OBE, what type of OBE did you have? *

Une seule réponse possible.

- I had only a Dissociative OBE.
- I had only an Associative OBE. *Passer à la section 11 (OBE Associative).*
- I had both a Dissociative and Associative OBE.
- I did not have an OBE. *Passer à la section 17 (NDE).*

OBE

OBE Dissociative

TASK INSTRUCTIONS FOR THE "OBE DISSOCIATIVE" DRAWING.

To help you complete the drawing task you must first familiarized yourself with the "Lexicon & Legend" (see. "Notice" page 1) and consult the OBE Dissociative example (see. Notice" page 6).

*Important note:
In this drawing document we ask you to describe and draw ONLY your OBE DISSOCIATIVE PART.
We advise you to keep all these documents in view while you are executing the drawing task. Please use the entire space of the drawing textbox:

1. Draw the outlines shape of the physical space you saw (eg room walls).

2. Draw a circle ○ at the position where your physical body was located.

3. Draw a cross + for the Self-Location (SL) from where you saw your physical body from an extracorporeal point of view.

4. Draw an arrow → to indicate the direction of your Self-Motion (SM) trajectory towards an extracorporeal point of view.

5. If other physical bodies were present in the physical space, draw as many squares □ as the number of other physical bodies to indicate where they were located in the physical space (eg a nurse, a cat).

Now, you have finished the drawing task. Please replace the OBE drawing document(s) in the chronological order according to the part number you have written on your document.

While keeping your eyes on the drawing you have just made, please answer the questions below.

OBE Dissociative digital questionnaire

A. OBE Dissociative: Physical Body Position

By physical body we mean the corporeal body which has a material existence.

6. 1. When my Dissociative OBE happened the position of my physical body was? *

Une seule réponse possible.

- Lying face down.
- Lying face up.
- Lying on the right side of the body.
- Lying on the left side of the body.
- Sitting.
- Standing.

B. OBE Dissociative: Self-Location/ Self-Motion Speed & Direction

7. 2. At your Dissociative Self-Location or during your Self-Motion onward trajectory towards an extracorporeal point of view while being out-of your physical body you felt: *

Plusieurs réponses possibles.

- [ ] Heavy.
- [ ] Floating.
- [ ] Weightless.
- [ ] Flying.
- [ ] I did not feel my physical body.
- [ ] I did not see my physical body.

8. 3. Your Self-Motion onward trajectory while you were heading towards an extracorporeal point of view was felt as a:
(You can thick more than one answer)

Plusieurs réponses possibles.

- ☐ Progressive acceleration.
- ☐ Progressive deceleration.
- ☐ In one shot.
- ☐ Alternately back and forth (oscillating between your physical body and an extracorporeal point of view).
- ☐ No motion.

9. 4. What type of Self-Motion onward trajectory did you feel while you were heading towards an extracorporeal point of view:

Plusieurs réponses possibles.

- ☐ Linear motion (you felt you were moving from one place to another in a straight line).
- ☐ Rotational motion (you felt you were moving along a circular path).
- ☐ U-Turn motion (a point in space where you felt you turned back voluntarily or involuntarily).

10. 4 bis. If you have experienced a linear self-motion while you were heading towards an extracorporeal point of view, use the hour markers of the clock dial below to specify the angle of your motion.

____

Clock dial

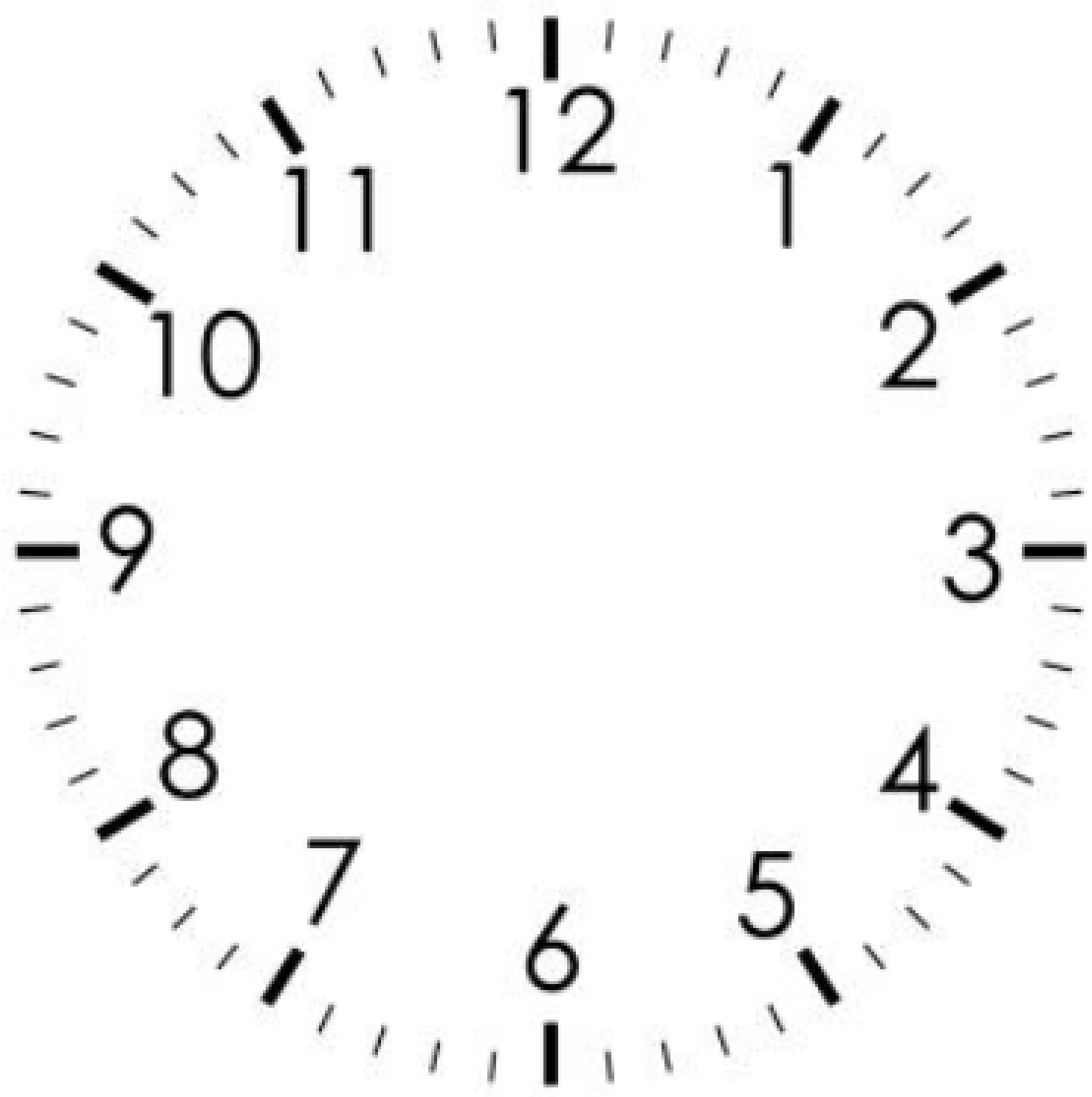

11. 5. During your Self-Motion onward trajectory towards an extracorporeal point of view, did you feel that you were actively controlling your Self-Motion trajectory direction or that you were passively observing your Self-Motion trajectory direction?

Une seule réponse possible.

- I was actively controlling my motion (I moved).
- I was passively observing my motion (I was moved).

12. **6. If you were actively controlling your Self-Motion onward trajectory while you were heading towards an extracorporeal point of view, did you feel a delay between your decision to move and your motion?**

Une seule réponse possible.

- I felt a delay between my decision of moving and my motion.
- I did not felt a delay between my decision of moving and my motion.

C. OBE Dissociative: Self-Motion: Emotion-Feeling-Perception

13. 7. During your Self-Motion onward trajectory, while you were heading towards an extracorporeal point of view, were your emotion(s) / feeling(s) positive, negative or neutral? *

(you can tick more than one answer).

Plusieurs réponses possibles.

- [ ] Positive.
- [ ] Negative.
- [ ] Neutral.

14. 8. If during your Self-Motion onward trajectory while you were heading towards an extracorporeal point of view, your emotion(s)/ feeling(s) were positive, do as follow:

A. NAME the positive emotion/feeling you had.

B. Write a number between 0 and 10 to express the INTENSITY LEVEL of each of the positive emotion/feeling you have felt.

C. Look at the Body Maps page 11-12 of the Notice :
*If you have felt some positive emotions on specific part(s) of the body, write in brackets the specific part(s) to indicate the positive emotions(s) location.
*If you did not feel some positive emotions on your physical body write: NO BODY
*If you felt some positive emotions all over your physical body write: ALL BODY.
*If you felt some positive emotions on your Self-Location (the place where you felt your sense of self or consciousness was spatially located) write: SELF

Example:
JOY: 5 (25).
WELL-BEING: 4 (ALL BODY)
HAPPINESS: 7 (1, 2, 3, 23).

15. **9. If during your Self-Motion onward trajectory while you were heading towards an extracorporeal point of view, your emotion(s)/ feeling(s) were negative, do as follow:**

A. NAME the negative emotion/feeling you had.

B. Choose a number between 0 and 10 to express the INTENSITY LEVEL of each of the negative emotion/feeling you have felt.

C. Look at the Body Maps page 11-12 of the Notice :
*If you have felt some negative emotions on specific part(s) of the body, write the specific part(s) to indicate the negative emotions(s) location.
*If you did not feel some negative emotions on your physical body write: NO BODY
*If you felt some negative emotions all over your physical body write: ALL BODY.
*If you felt some negative emotions on your Self-Location (the place where you felt your sense of self or consciousness was spatially located) write: SELF

Examples :
FEAR: 8 (46, 47).
SADNESS: 6 (15, 26, 27).
DISGUST: 3 (4, 3, 25).

16. 10. If during your Self-Motion onward trajectory while you were heading towards an extracorporeal point of view, your emotion(s)/ feeling(s) were neutral, do as follow:

A. NAME the neutral emotion/feeling you had.

B. Choose a number between 0 and 10 to express the INTENSITY LEVEL of each of the neutral emotion/feeling you have felt.

C. Look at the Body Maps page 11-12 of the Notice :
*if you have felt some neutral emotions on specific part(s) of the body, write the specific part(s) to indicate the neutral emotions(s) location.
*If you did not feel some neutral emotions on your physical body write: NO BODY
*If you felt some neutral emotions all over your physical body write: ALL BODY
*If you felt some neutral emotions on your Self-Location (the place where you felt your sense of self or consciousness was spatially located) write: SELF

Examples:
INDIFFERENCE: 9 (NO BODY).
SURPRISE: 2 (1,2).
SERENITY: 10 (NO BODY).

17. 11. What type of sensory modalities did you experienced during the Dissociative part of your OBE? *
(You can tick more than one answer)

Plusieurs réponses possibles.

- [ ] Vision.
- [ ] Hearing (audition).
- [ ] Smell (olfaction).
- [ ] Taste (gustation).
- [ ] Touch (somatosensation).
- [ ] Temperature (thermoception), (e.g. I felt cold/hot).
- [ ] Pain (nociception).

18. Now you have finished answering the OBE Dissociative questionnaire.

BOX A:
If your only experienced a Dissociative OBE PART, we thank you very much for the time you took to participate in our questionnaire and we kindly ask you to check box A to go to the final section of our questionnaire.

BOX B:
If you have also experienced an Associative OBE PART, please check box B to proceed to the OBE Associative questionnaire.

BOX C:
If you had only NDE PART(S), please check box C to proceed to the next stage of our NDE questionnaire.

Une seule réponse possible.

- ( ) A: Go to the final section of our questionnaire.
  Passer à la question 276
- ( ) B: Proceeding to the OBE Associative questionnaire.
  Passer à la section 11 (OBE Associative).
- ( ) C: Proceeding to the next stage. *Passer à la section 17 (NDE).*

OBE Associative

OBE Associative: Graphic Questionnaire

TASK INSTRUCTIONS FOR THE "OBE ASSOCIATIVE" DRAWING.

- To help you complete the drawing task you must first familiarized yourself with the "Lexicon & Legend" (see. "Notice" page 1) and consult the OBE Associative example (see. "Notice" page 10).
- We advise you to keep all these documents in view while you are executing the drawing task. Please use the entire space of the drawing textbox:

*Important note: in this drawing document we ask you to describe and draw ONLY your OBE ASSOCIATIVE PART.

1. Draw the outlines shape of the physical space you saw (e.g. room walls).

2. Draw a circle ○ at the position where your physical body was located.

3. Draw a cross + at the Self-Location (S-L) from where you saw your physical body from an extracorporeal point of view.

4. Draw an arrow → to indicate the return direction of your Self-Motion (S-M) trajectory from an extracorporeal point of view into your physical body and/or the physical world.

5. if other physical bodies were present, draw as many squares □ as the number of other physical bodies to indicate where they were located in the physical space (e.g. a nurse, a cat).

Now, you have finished the drawing task. Please replace the OBE drawing document(s) in the chronological order according to the part number you have written on your document.

While keeping your eyes on the drawing you have just made, please answer the questions below.

OBE Associative: Digital Questionnaire

A. OBE Associative: Physical Body Position

By physical body we mean the corporeal body which has a material existence.

19. 1. When my Associative OBE ended the position of my physical body was? *

Une seule réponse possible.

- Lying face down.
- Lying face up.
- Lying on the right side of the body.
- Lying on the left side of the body.
- Sitting.
- Standing.

B. OBE Associative: Self-Location/Self-Motion Speed & Direction

20. 2. At your Associative Self-Location or During Self-Motion return trajectory while you were heading back from your extracorporeal point of view into your physical body and/or the physical space you felt: *

Plusieurs réponses possibles.

- [ ] Heavy.
- [ ] Floating.
- [ ] Weightless.
- [ ] Flying.
- [ ] I did not feel my physical body.
- [ ] I did not see my physical body.

21. 3. Your Self-Motion return trajectory back while you were heading from your extracorporeal point of view into your physical body and/or the physical word was felt as a:

Plusieurs réponses possibles.

- [ ] Progressive acceleration.
- [ ] Progressive deceleration.
- [ ] In one shot.
- [ ] Alternately back and forth (oscillating between your physical body and your extracorporeal point of view).
- [ ] No motion.

22. 4. What type of Self-Motion return trajectory while you were heading back from your extracorporeal point of view into your physical body and/or the physical world, did you experienced?

Plusieurs réponses possibles.

- [ ] Linear motion (you felt you were moving from one place to another in a straight line).
- [ ] Rotational motion (you felt you were moving along a circular path).
- [ ] U-Turn motion (a point in space where you felt you turned back voluntarily or involuntarily).

23. 4 bis. If you have experienced a linear motion while you were heading back from your extracorporeal point of view into your physical body and/or the physical world, use the hour markers of the clock dial below to specify the angle of your motion.

____

Clock dial

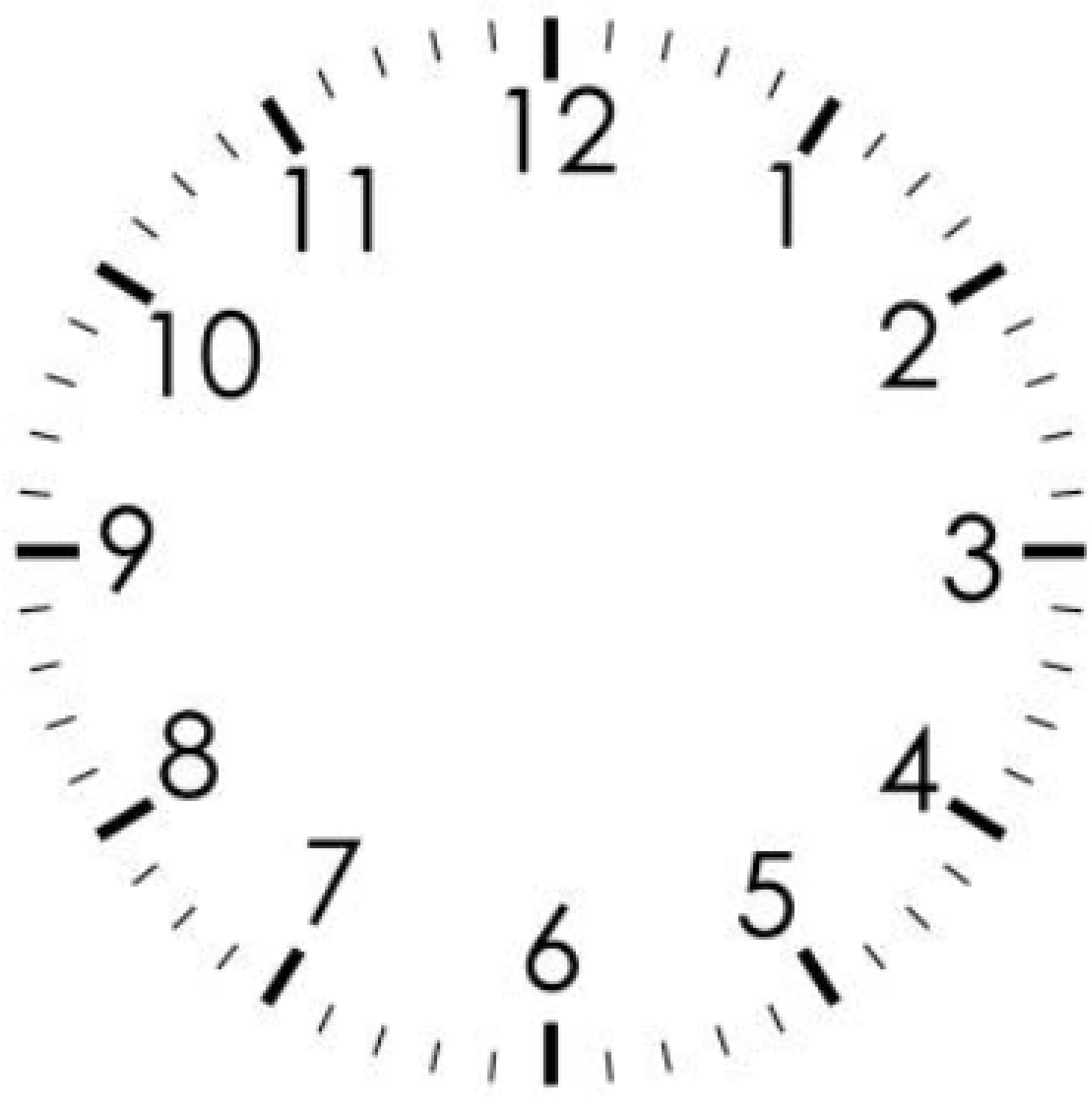

24. 5. During your Self-Motion return trajectory while you were heading back from your extracorporeal point of view into your physical body and/or the physical world, did you feel that you were actively controlling your Self-Motion return trajectory direction or that you were passively observing your Self-Motion return trajectory direction?

Une seule réponse possible.

- [ ] I was actively controlling my motion (I moved).
- [ ] I was passively observing my motion (I was moved).

25. **6. If you were actively controlling your Self-Motion return trajectory while you were heading back from your extracorporeal point of view into your physical body and/or the physical world, did you feel a delay between your decision to move and your motion?**

Une seule réponse possible.

- ( ) I felt a delay between my decision of moving and my motion.
- ( ) I did not felt a delay between my decision of moving and my motion.

C. OBE Associative: Self-Motion & Emotion-Feeling-Perception

26. 7. During your Self-Motion return trajectory while you were heading back from your extracorporeal point of view into your physical body and/or the physical world, your emotion(s)/ feeling(s) were positive, negative or neutral?
(you can tick more than one answer). *

Plusieurs réponses possibles.

- [ ] Positive.
- [ ] Negative.
- [ ] Neutral.

27. 8. If during your Self-Motion return trajectory while you were heading back from your extracorporeal point of view into your physical body and/or the physical world, your emotion(s) / feeling(s) were positive, do as follow:

A. NAME the positive emotion/feeling you had.

B. Choose a number between 0 and 10 to express the INTENSITY LEVEL of each of the positive emotion/feeling you have felt.

C. Look at the Body Maps page 11-12 of the Notice :
*If you have felt some positive emotions on specific part(s) of the body, write in brackets the specific part(s) to indicate the positive emotions(s) location.
*If you did not feel some positive emotions on your physical body write: NO BODY
*If you felt some positive emotions all over your physical body write: ALL BODY.

*If you felt some positive emotions on your Self-Location (the place where you felt your sense of self or consciousness was spatially located) write: SELF

Example:
JOY: 5 (25).
WELL-BEING: 4 (ALL BODY)
HAPPINESS: 7 (1, 2, 3, 23).

28. 9. If during your Self-Motion onward trajectory while you were heading towards an extracorporeal point of view, your emotion(s)/ feeling(s) were negative, do as follow:

A. NAME the negative emotion/feeling you had.

B. Write a number between 0 and 10 to express the INTENSITY LEVEL of each of the negative emotion/feeling you have felt.

C. Look at the Body Maps page 11-12 of the Notice :
*If you have felt some negative emotions on specific part(s) of the body, write in brackets the specific part(s) to indicate the negative emotions(s) location.
*If you did not feel some negative emotions on your physical body write: NO BODY
*If you felt some negative emotions all over your physical body write: ALL BODY.
*If you felt some negative emotions on your Self-Location (the place where you felt your sense of self or consciousness was spatially located) write: SELF

Examples :
FEAR: 8 (46, 47).
SADNESS: 6 (15, 26, 27).
DISGUST: 3 (4, 3, 25).

29. 10. If during your Self-Motion onward trajectory while you were heading towards an extracorporeal point of view, your emotion(s)/ feeling(s) were neutral, do as follow:

A. NAME the neutral emotion/feeling you had.

B. Choose a number between 0 and 10 to express the INTENSITY LEVEL of each of the neutral emotion/feeling you have felt.

C. Look at the Body Maps page 11-12 of the Notice :
*If you have felt some neutral emotions on specific part(s) of the body, write the specific part(s) to indicate the neutral emotions(s) location.
*If you did not feel some neutral emotions on your physical body write: NO BODY
*If you felt some neutral emotions all over your physical body write: ALL BODY.
*If you felt some neutral emotions on your Self-Location (the place where you felt your sense of self or consciousness was spatially located) write: SELF

Examples:
INDIFFERENCE: 9 (NO BODY).
SURPRISE: 2 (1,2).
SERENITY: 10 (NO BODY).

30. 11. What type of sensory modalities did you experienced during the Associative part of your OBE? *
(You can tick more than one answer)

Plusieurs réponses possibles.

- [ ] Vision.
- [ ] Hearing (audition).
- [ ] Smell (olfaction).
- [ ] Taste (gustation).
- [ ] Touch (somatosensation).
- [ ] Temperature (thermoception), e.g. I felt cold/hot.
- [ ] Pain (nociception).

31. Now you have finished answering the OBE Associative questionnaire.

BOX A: If you only experienced OBE PART(S) (Associative part or both Dissociative and Associative parts), we thank you very much for the time you took to participate in our questionnaire and we kindly ask you to check box A to go to the final section of our questionnaire.

BOX B: If you experienced OBE PART(S) and also NDE PART(S), please check box B to proceed to the next stage of our NDE questionnaire.

Une seule réponse possible.

( ) A: Go to the final section of our questionnaire. *Passer à la question 276*

( ) B: Proceeding to the next stage. *Passer à la section 17 (NDE).*

NDE

NDE Part 1: First Space Graphic Questionnaire

*Important note: in this drawing document we ask you to describe and draw ONLY YOUR NDE PART 1.

ORGANIZING YOUR OBE/NDE DRAWING DOCUMENTS

If you had a Dissociative or/and an Associative OBE you must have already completed the OBE written graphic and digital questionnaire.

FOR THE CHRONOLOGICAL ORDERING OF YOUR OBE and/ or NDE DRAWING DOCUMENTS do as follow:

Place on your table all your NDE drawing documents in the chronological order of the NDE part number written in the second textbox of your NDE drawing document.

If you had a Dissociative OBE part, its drawing document should be placed before all your NDE part(s) drawing document(s).

If you had an Associative OBE part, its drawing document should be placed after all your NDE part(s) drawing document(s).

If you had both a Dissociative OBE part and an Associative OBE part, the OBE Dissociative drawing document should be placed before all your NDE part(s) drawing document(s) and the OBE Associative drawing document should be placed after all your NDE part(s) drawing document(s).

Now you are going to fill your NDE PART 1 drawing document.

INSTRUCTIONS TO FILL IN THE "NDE" DRAWING DOCUMENT

To help you complete your NDE drawing document(s), please read carefully the 4 documents below (you have previously printed):

1. The "Lexicon & Legend" ("Notice" page 1).

2. The "Preface of the catalogue of NDE spaces shapes outlines" ("Notice" page 2).

3. The "Catalogue of NDE spaces shapes outlines" ("Notice" page 3, 4, 5).

4. The "Examples of NDE spaces shapes outlines of types A, B, C ("Notice" page 7, 8,9).

Now, that you have closely reviewed the 4 documents, please follow these steps:

REMEMBERING THE FIRST SPACE SHAPE OUTLINES OF YOUR NDE (PART 1).

Take your time to remember your FIRST NDE SPACE SHAPE OUTLINES. To help you visualizing its space shape outlines you might want to close your eyes.

LOOK at the A, B, C types of shape outlines in the "Notice" (page 3, 4, 5) and choose the type of space shape outlines which is the closest to the space shape outlines of your FIRST NDE SPACE.

CHOOSE among the A, B, C types of spaces shapes outlines in the "Notice" (page 3, 4, 5) ONLY 1 SPACE SHAPE OUTLINES FOR YOUR FIRST NDE SPACE.

If 1 shape outlines among the A, B, C types of spaces shapes outlines corresponds to your FIRST NDE SPACE SHAPE OUTLINES, write in the third textbox of your NDE drawing document the NDE code space shape outlines number you have chosen in the "Notice" (page 3, 4, 5).
Eg. write A3 for the first row and the third column for a space shape outlines of type A.

If no space shape outlines corresponds to the space shape outlines of your FIRST NDE SPACE SHAPE OUTLINES you saw, write “other” in the third textbox of your NDE PART 1 drawing document.

DRAWING TASK INSTRUCTIONS FOR "NDE" DOCUMENT

To guide you through the next steps of your drawing task, consult the Legend & Lexicon ("Notice" page 1) and the NDE types A, B, C examples of the "Notice" (page 7, 8,9). We advise you to keep all these documents in view during your drawing task.

Please use the entire space of the drawing textbox to execute your drawing.

If you have written in the third textbox a NDE code space shape outlines number please REDRAW the space shape outlines you have chosen to describe your FIRST SPACE SHAPE OUTLINES from the "Notice (page 3, 4, 5) in the textbox of your NDE part 1 drawing document.

If you have written in the third textbox “other” because no space shape outlines corresponded to the space shape outlines of your FIRST NDE SPACE SHAPE OUTLINES, DRAW FREELY in the drawing textbox your FIRST NDE SPACE SHAPE OUTLINES.

Now that you have finished reproducing or drawing freely your FIRST NDE SPACE SHAPE OUTLINES, you will have to indicate on it your SELF-LOCATION(s) (S-L) as well as the POSITION(s) of OTHER BEINGS (if any other beings were present) and your SELF-MOTION(S) (S-M) .

To do so, please follow the instructions below:

1. Draw a cross + to represent each of your Self-Location (S-L) namely, each place where your feel your self is located and/or has stopped.

2. Write a number next to each of the Self-Location (S-L) cross to give the chronological order in which you have experienced them. (Eg. Self-Location number 1= S-L1; Self-Location number 2= S-L2).

3. Draw an arrow → to represent the direction of your Self-Motion trajectory in between each of your Self-Location (S-L).

4. If you have felt in this space that your Self has made a U-turn, draw a U-turn arrow ↶ to indicate the exact location of your U-turn.

5. if they were any kind of other beings, draw one square □ to represent each being positions .

Now, you have completed the drawing task, please replace the NDE drawing document(s) in the chronological order accordingly to the NDE part number you have written in the third textbox.

While keeping your eyes on the drawing you have just made, please answer the questions below:

NDE Part 1: First Space Digital Questionnaire

A. NDE Part 1: First Space Appearances & Beings

The way your first NDE space look like and which beings you encountered.

32. 1. Was your first NDE space a two-dimensional or a three-dimensional space? *

A "2D" (two-dimensional), simply means the shape is flat, (e.g. a circle).

A "3D" (three-dimensional) shape is a solid shape. It has three dimensions. This is its length, width and depth, (e.g. a sphere).

Une seule réponse possible.

- Two-dimensional.
- Three-dimensional.

33. 2. Was your first NDE space chromatic or achromatic? *

Une seule réponse possible.

- Chromatic (all the colors: eg. pink).
- Achromatic (black, grey, white and light textures).

34. 3. What size did you feel your first NDE space was? *

Une seule réponse possible.

- Infinitely big.
- Human size.
- Infinitely small.

35. 4. If in your first NDE space you have encountered any kind of beings, please specify how many and give a brief description of each one. If you did not encounter any beings you can specify the appearances of the space or any phenomena that happened in that space (eg. hearing voices, feeling of being touched/ enveloped or fusing with the space itself).

______

______

______

______

______

B. NDE Part 1: First Space Self-Motion & Speed

36. 5. In your first NDE space, did you experienced Self-Motion? *

(What we imply by Self-Motion is your sensation of spontaneous or voluntary motion in an extracorporeal space).

Une seule réponse possible.

- ( ) Yes, I had the sensation of moving in this space.
- ( ) No, I did not have the sensation of moving in this space. *Passer à la question 41*

37. 6. In your first NDE space the direction of your Self-Motion trajectory was: (you can tick more than one answer)

Plusieurs réponses possibles.

- [ ] Up
- [ ] Down
- [ ] Forward
- [ ] Backward
- [ ] Left
- [ ] Right
- [ ] Moving around a center
- [ ] At the center
- [ ] U-Turn motion

38. 7. In your first NDE space, the speed of your Self-Motion was: (you can tick more than one answer)

Plusieurs réponses possibles.

- [ ] "At the speed of light".
- [ ] Extremely fast.
- [ ] Fast.
- [ ] Moderate.
- [ ] Slow.
- [ ] Extremely slow.
- [ ] Progressive acceleration (I felt the speed increasing on the way).
- [ ] Progressive deceleration (I felt the speed decreasing on the way).
- [ ] In one shot (I have not stopped on the way).

39. **8. In your first NDE space, were you actively controlling the direction of your Self-Motion or passively observing the direction of your Self-Motion?**

Une seule réponse possible.

- I was actively controlling the direction of my Self-Motion (I moved).
- I was passively observing the direction of my Self-Motion (I was moved).

40. **9. In your first NDE space, if you were actively controlling the direction of your Self-Motion, did you feel a delay between your decision to move and your motion?**

Une seule réponse possible.

- I felt a delay between my decision of moving and my motion.
- I did not felt a delay between my decision of moving and my motion.

C. NDE Part 1: First Space Perception

41. **10. What type of sensory modalities did you experienced in your first NDE space?**
(You can tick more than one answer) *

Plusieurs réponses possibles.

- [ ] Vision.
- [ ] Hearing (audition).
- [ ] Smell (olfaction).
- [ ] Taste (gustation).
- [ ] Touch (somatosensation).
- [ ] Temperature (thermoception), e.g. I felt cold/hot.
- [ ] Pain (nociception).
- [ ] No sensory perception.

D. NDE Part 1: First Space Self-Location & Emotion-Feeling

Now, please look at the drawing of your first NDE space (NDE part 1) and count the total number of crosses of the Self-Location (S-L) you had in this space, this will give you the answer to the question below.

42. 11. How many Self-Location (S-L) did you count in total in your first NDE space? *

Une seule réponse possible.

1	2	3	4	5	6	7	8	9	10
◯	◯	◯	◯	◯	◯	◯	◯	◯	◯

a. NDE Part 1: First NDE Space Self-Location Number 1

43. 1. In your first NDE space at your Self-Location number 1 (S-L1), your emotions/ feelings were positive, negative or neutral? (you can tick more than one answer). *

Plusieurs réponses possibles.

- [ ] Positive.
- [ ] Negative.
- [ ] Neutral.

44. 2. If in your first NDE space at your Self-Location number 1 (S-L1), your emotion(s) / feeling(s) were positive, do as follow:

A. NAME the positive emotion/feeling you had.
B. Write a number between 0 and 10 to express the INTENSITY LEVEL of each of the positive emotion/feeling you have felt.

C. Look at the Body Maps page 11-12 of the Notice :

*If you have felt some positive emotions on specific part(s) of the physical body, write in brackets the specific part(s) to indicate the positive emotions(s) location.

*If you did not feel some positive emotions on your physical body write: NO BODY

*If you felt some positive emotions all over your physical body write: ALL BODY

*If you felt some positive emotions on your Self-Location (the place where you felt your sense of self or consciousness was spatially located) write: SELF

Example:
JOY: 5 (25).
WELL-BEING: 4 (ALL BODY)
HAPPINESS: 7 (1, 2, 3, 23).

45. 3. If in your first NDE space at your Self-Location number 1 (S-L1), your emotion(s)/ feeling(s) were negative, do as follow:

A. NAME the negative emotion/feeling you had.
B. Write a number between 0 and 10 to express the INTENSITY LEVEL of each of the negative emotion/feeling you have felt.

C. Look at the Body Maps page 11-12 of the Notice :

*If you have felt some negative emotions on specific part(s) of the body, write in brackets the specific part(s) to indicate the negative emotions(s) location.

*If you did not feel some negative emotions on your physical body write: NO BODY

*If you felt some negative emotions all over your physical body write: ALL BODY

*If you felt some negative emotions on your Self-Location (the place where you felt your sense of self or consciousness was spatially located) write: SELF

Examples :
FEAR: 8 (SELF).
SADNESS: 6 (SELF).
DISGUST: 3 (SELF).

46. 4. If in your first NDE space at your Self-Location number 1 (S-L1), your emotion(s) / feeling(s) were neutral, do as follow:

A. NAME the neutral emotion/feeling you had.

B. Choose a number between 0 and 10 to express the INTENSITY LEVEL of each of the neutral emotion/feeling you have felt.

C. Look at the Body Maps page 11-12 of the Notice :
*If you have felt some neutral emotions on specific part(s) of the body, write the specific part(s) to indicate the neutral emotions(s) location.
*If you did not feel some neutral emotions on your physical body write: NO BODY
*If you felt some neutral emotions all over your physical body write: ALL BODY
*If you felt some neutral emotions on your Self-Location (the place where you felt your sense of self or consciousness was spatially located) write: SELF

Examples:
INDIFFERENCE: 9 (NO BODY).
SURPRISE: 2 (1,2).
SERENITY: 10 (NO BODY).

47. Tick the right answer

Une seule réponse possible.

- ( ) In your first NDE space (NDE part 1) you only had 1 Self-Location. *Passer à la question 92*
- ( ) In your first NDE space (NDE part 1) you had 2 or more than 2 Self-Locations.

b. NDE Part 1: First NDE Space Self-Location Number 2

48. 1. In your first NDE space at your Self-Location number 2 (S-L2), your emotions/ feelings were positive, negative or neutral? (you can tick more than one answer). *

Plusieurs réponses possibles.

- [ ] Positive.
- [ ] Negative.
- [ ] Neutral.

49. 2. If in your first NDE space at your Self-Location number 2 (S-L2), your emotion(s) / feeling(s) were positive, do as follow:

A. NAME the positive emotion/feeling you had.

B. Write a number between 0 and 10 to express the INTENSITY LEVEL of each of the positive emotion/feeling you have felt.

C. Look at the Body Maps page 11-12 of the Notice :

*If you have felt some positive emotions on specific part(s) of the physical body, write in brackets the specific part(s) to indicate the positive emotions(s) location.

*If you did not feel some positive emotions on your physical body write: NO BODY

*If you felt some positive emotions all over your physical body write: ALL BODY

*If you felt some positive emotions on your Self-Location (the place where you felt your sense of self or consciousness was spatially located) write: SELF

Example:
JOY: 5 (25).
WELL-BEING: 4 (ALL BODY)
HAPPINESS: 7 (1, 2, 3, 23).

50. 3. If in your first NDE space at your Self-Location number 2 (S-L2), your emotion(s)/ feeling(s) were negative, do as follow:

A. NAME the negative emotion/feeling you had.

B. Write a number between 0 and 10 to express the INTENSITY LEVEL of each of the negative emotion/feeling you have felt.
C. Look at the Body Maps page 11-12 of the Notice :

*If you have felt some negative emotions on specific part(s) of the body, write in brackets the specific part(s) to indicate the negative emotions(s) location.

*If you did not feel some negative emotions on your physical body write: NO BODY

*If you felt some negative emotions all over your physical body write: ALL BODY

*If you felt some negative emotions on your Self-Location (the place where you felt your sense of self or consciousness was spatially located) write: SELF

Examples :
FEAR: 8 (SELF).
SADNESS: 6 (SELF).
DISGUST: 3 (SELF).

51. 4. If in your first NDE space at your Self-Location number 2 (S-L2), your emotion(s) / feeling(s) were neutral, do as follow:

A. NAME the neutral emotion/feeling you had.

B. Choose a number between 0 and 10 to express the INTENSITY LEVEL of each of the neutral emotion/feeling you have felt.

C. Look at the Body Maps page 11-12 of the Notice :
*If you have felt some neutral emotions on specific part(s) of the body, write the specific part(s) to indicate the neutral emotions(s) location.
*If you did not feel some neutral emotions on your physical body write: NO BODY
*If you felt some neutral emotions all over your physical body write: ALL BODY
*If you felt some neutral emotions on your Self-Location (the place where you felt your sense of self or consciousness was spatially located) write: SELF

Examples:
INDIFFERENCE: 9 (NO BODY).
SURPRISE: 2 (1,2).
SERENITY: 10 (NO BODY).

52. Tick the right answer

Une seule réponse possible.

- In your first NDE space (NDE part 1) you only had 2 Self-Locations. *Passer à la question 92*
- In your first NDE space (NDE part 1) you had 3 or more than 3 Self-Locations.

c. NDE Part 1: First NDE Space Self-Location Number 3

53. 1. In your first NDE space at your Self-Location number 3 (S-L3), your emotions/feelings were positive, negative or neutral? *
(you can tick more than one answer).

Plusieurs réponses possibles.

- [ ] Positive.
- [ ] Negative.
- [ ] Neutral.

54. 2. If in your first NDE space at your Self-Location number 3 (S-L3), your emotion(s) / feeling(s) were positive, do as follow:

A. NAME the positive emotion/feeling you had.

B. Write a number between 0 and 10 to express the INTENSITY LEVEL of each of the positive emotion/feeling you have felt.

C. Look at the Body Maps page 11-12 of the Notice :

*If you have felt some positive emotions on specific part(s) of the physical body, write in brackets the specific part(s) to indicate the positive emotions(s) location.

*If you did not feel some positive emotions on your physical body write: NO BODY

*If you felt some positive emotions all over your physical body write: ALL BODY

*If you felt some positive emotions on your Self-Location (the place where you felt your sense of self or consciousness was spatially located) write: SELF

Example:
JOY: 5 (25).
WELL-BEING: 4 (ALL BODY)
HAPPINESS: 7 (1, 2, 3, 23).

55. **3. If in your first NDE space at your Self-Location number 3 (S-L3), your emotion(s)/ feeling(s) were negative, do as follow:**

A. NAME the negative emotion/feeling you had.

B. Write a number between 0 and 10 to express the INTENSITY LEVEL of each of the negative emotion/feeling you have felt.
C. Look at the Body Maps page 11-12 of the Notice :

*If you have felt some negative emotions on specific part(s) of the body, write in brackets the specific part(s) to indicate the negative emotions(s) location.

*If you did not feel some negative emotions on your physical body write: NO BODY

*If you felt some negative emotions all over your physical body write: ALL BODY

*If you felt some negative emotions on your Self-Location (the place where you felt your sense of self or consciousness was spatially located) write: SELF

Examples :
FEAR: 8 (SELF).
SADNESS: 6 (SELF).
DISGUST: 3 (SELF).

56. 4. If in your first NDE space at your Self-Location number 3 (S-L3), your emotion(s) / feeling(s) were neutral, do as follow:

A. NAME the neutral emotion/feeling you had.

B. Choose a number between 0 and 10 to express the INTENSITY LEVEL of each of the neutral emotion/feeling you have felt.

C. Look at the Body Maps page 11-12 of the Notice :
*If you have felt some neutral emotions on specific part(s) of the body, write the specific part(s) to indicate the neutral emotions(s) location.
*If you did not feel some neutral emotions on your physical body write: NO BODY
*If you felt some neutral emotions all over your physical body write: ALL BODY
*If you felt some neutral emotions on your Self-Location (the place where you felt your sense of self or consciousness was spatially located) write: SELF

Examples:
INDIFFERENCE: 9 (NO BODY).
SURPRISE: 2 (1,2).
SERENITY: 10 (NO BODY).

57. Tick the right answer

Une seule réponse possible.

- In your first NDE space (NDE part 1) you only had 3 Self-Locations. *Passer à la question 92*
- In your first NDE space (NDE part 1) you had 4 or more than 4 Self-Locations.

d. NDE Part 1: First NDE Space Self-Location Number 4

58. 1. In your first NDE space at your Self-Location number 4 (S-L4), your emotions/ feelings were positive, negative or neutral? *
(you can tick more than one answer).

Plusieurs réponses possibles.

- [ ] Positive.
- [ ] Negative.
- [ ] Neutral.

59. 2. If in your first NDE space at your Self-Location number 4 (S-L4), your emotion(s) / feeling(s) were positive, do as follow:
A. NAME the positive emotion/feeling you had.

B. Write a number between 0 and 10 to express the INTENSITY LEVEL of each of the positive emotion/feeling you have felt.

C. Look at the Body Maps page 11-12 of the Notice :

*If you have felt some positive emotions on specific part(s) of the physical body, write in brackets the specific part(s) to indicate the positive emotions(s) location.

*If you did not feel some positive emotions on your physical body write: NO BODY

*If you felt some positive emotions all over your physical body write: ALL BODY

*If you felt some positive emotions on your Self-Location (the place where you felt your sense of self or consciousness was spatially located) write: SELF

Example:
JOY: 5 (25).
WELL-BEING: 4 (ALL BODY)
HAPPINESS: 7 (1, 2, 3, 23).

60. 3. If in your first NDE space at your Self-Location number 4 (S-L4), your emotion(s)/ feeling(s) were negative, do as follow:

A. NAME the negative emotion/feeling you had.

B. Write a number between 0 and 10 to express the INTENSITY LEVEL of each of the negative emotion/feeling you have felt.
C. Look at the Body Maps page 11-12 of the Notice :

*If you have felt some negative emotions on specific part(s) of the body, write in brackets the specific part(s) to indicate the negative emotions(s) location.

*If you did not feel some negative emotions on your physical body write: NO BODY

*If you felt some negative emotions all over your physical body write: ALL BODY

*If you felt some negative emotions on your Self-Location (the place where you felt your sense of self or consciousness was spatially located) write: SELF

Examples :
FEAR: 8 (SELF).
SADNESS: 6 (SELF).
DISGUST: 3 (SELF).

61. 4. If in your first NDE space at your Self-Location number 4 (S-L4), your emotion(s) / feeling(s) were neutral, do as follow:

A. NAME the neutral emotion/feeling you had.

B. Choose a number between 0 and 10 to express the INTENSITY LEVEL of each of the neutral emotion/feeling you have felt.

C. Look at the Body Maps page 11-12 of the Notice :
*If you have felt some neutral emotions on specific part(s) of the body, write the specific part(s) to indicate the neutral emotions(s) location.
*If you did not feel some neutral emotions on your physical body write: NO BODY
*If you felt some neutral emotions all over your physical body write: ALL BODY
*If you felt some neutral emotions on your Self-Location (the place where you felt your sense of self or consciousness was spatially located) write: SELF

Examples:
INDIFFERENCE: 9 (NO BODY).
SURPRISE: 2 (1,2).
SERENITY: 10 (NO BODY).

62. Tick the right answer

Une seule réponse possible.

- In your first NDE space (NDE part 1) you only had 4 Self-Locations . *Passer à la question 92*
- In your first NDE space (NDE part 1) you had 5 or more than 5 Self-Locations.

e. NDE Part 1: First NDE Space Self-Location Number 5

63. 1. In your first NDE space at your Self-Location number 5 (S-L5), your emotions/ feelings were positive, negative or neutral? *
(you can tick more than one answer).

Plusieurs réponses possibles.

- [ ] Positive.
- [ ] Negative.
- [ ] Neutral.
- [ ] Autre : ______________________

64. 2. If in your first NDE space at your Self-Location number 5 (S-L5), your emotion(s) / feeling(s) were positive, do as follow:

A. NAME the positive emotion/feeling you had.

B. Write a number between 0 and 10 to express the INTENSITY LEVEL of each of the positive emotion/feeling you have felt.

C. Look at the Body Maps page 11-12 of the Notice :

*If you have felt some positive emotions on specific part(s) of the physical body, write in brackets the specific part(s) to indicate the positive emotions(s) location.

*If you did not feel some positive emotions on your physical body write: NO BODY

*If you felt some positive emotions all over your physical body write: ALL BODY

*If you felt some positive emotions on your Self-Location (the place where you felt your sense of self or consciousness was spatially located) write: SELF

Example:
JOY: 5 (25).
WELL-BEING: 4 (ALL BODY)
HAPPINESS: 7 (1, 2, 3, 23).

65. **3. If in your first NDE space at your Self-Location number 5 (S-L5), your emotion(s)/ feeling(s) were negative, do as follow:**

A. NAME the negative emotion/feeling you had.

B. Write a number between 0 and 10 to express the INTENSITY LEVEL of each of the negative emotion/feeling you have felt.
C. Look at the Body Maps page 11-12 of the Notice :

*If you have felt some negative emotions on specific part(s) of the body, write in brackets the specific part(s) to indicate the negative emotions(s) location.

*If you did not feel some negative emotions on your physical body write: NO BODY

*If you felt some negative emotions all over your physical body write: ALL BODY

*If you felt some negative emotions on your Self-Location (the place where you felt your sense of self or consciousness was spatially located) write: SELF

Examples :
FEAR: 8 (SELF).
SADNESS: 6 (SELF).
DISGUST: 3 (SELF).

66. 4. If in your first NDE space at your Self-Location number 5 (S-L5), your emotion(s) / feeling(s) were neutral, do as follow:

A. NAME the neutral emotion/feeling you had.

B. Choose a number between 0 and 10 to express the INTENSITY LEVEL of each of the neutral emotion/feeling you have felt.

C. Look at the Body Maps page 11-12 of the Notice :
*If you have felt some neutral emotions on specific part(s) of the body, write the specific part(s) to indicate the neutral emotions(s) location.
*If you did not feel some neutral emotions on your physical body write: NO BODY
*If you felt some neutral emotions all over your physical body write: ALL BODY
*If you felt some neutral emotions on your Self-Location (the place where you felt your sense of self or consciousness was spatially located) write: SELF

Examples:
INDIFFERENCE: 9 (NO BODY).
SURPRISE: 2 (1,2).
SERENITY: 10 (NO BODY).

67. Tick the right answer

Une seule réponse possible.

- In your first NDE space (NDE part 1) you only had 5 Self-Locations. *Passer à la question 92*
- In your first NDE space (NDE part 1) you had 6 or more than 6 Self-Locations.

f. NDE Part 1: First NDE Space Self-Location Number 6

68. 1. In your first NDE space at your Self-Location number 6 (S-L6), your emotions/feelings were positive, negative or neutral? (you can tick more than one answer). *

Plusieurs réponses possibles.

- [ ] Positive.
- [ ] Negative.
- [ ] Neutral.

69. 2. If in your first NDE space at your Self-Location number 6 (S-L6), your emotion(s) / feeling(s) were positive, do as follow:

A. NAME the positive emotion/feeling you had.

B. Write a number between 0 and 10 to express the INTENSITY LEVEL of each of the positive emotion/feeling you have felt.

C. Look at the Body Maps page 11-12 of the Notice :

*If you have felt some positive emotions on specific part(s) of the physical body, write in brackets the specific part(s) to indicate the positive emotions(s) location.

*If you did not feel some positive emotions on your physical body write: NO BODY

*If you felt some positive emotions all over your physical body write: ALL BODY

*If you felt some positive emotions on your Self-Location (the place where you felt your sense of self or consciousness was spatially located) write: SELF

Example:
JOY: 5 (25).
WELL-BEING: 4 (ALL BODY)
HAPPINESS: 7 (1, 2, 3, 23).

70. 3. If in your first NDE space at your Self-Location number 6 (S-L6), your emotion(s)/ feeling(s) were negative, do as follow:

A. NAME the negative emotion/feeling you had.

B. Write a number between 0 and 10 to express the INTENSITY LEVEL of each of the negative emotion/feeling you have felt.
C. Look at the Body Maps page 11-12 of the Notice :

*If you have felt some negative emotions on specific part(s) of the body, write in brackets the specific part(s) to indicate the negative emotions(s) location.

*If you did not feel some negative emotions on your physical body write: NO BODY

*If you felt some negative emotions all over your physical body write: ALL BODY

*If you felt some negative emotions on your Self-Location (the place where you felt your sense of self or consciousness was spatially located) write: SELF

Examples :
FEAR: 8 (SELF).
SADNESS: 6 (SELF).
DISGUST: 3 (SELF).

71. 4. If in your first NDE space at your Self-Location number 6 (S-L6), your emotion(s) / feeling(s) were neutral, do as follow:

A. NAME the neutral emotion/feeling you had.

B. Choose a number between 0 and 10 to express the INTENSITY LEVEL of each of the neutral emotion/feeling you have felt.

C. Look at the Body Maps page 11-12 of the Notice :
*If you have felt some neutral emotions on specific part(s) of the body, write the specific part(s) to indicate the neutral emotions(s) location.
*If you did not feel some neutral emotions on your physical body write: NO BODY
*If you felt some neutral emotions all over your physical body write: ALL BODY
*If you felt some neutral emotions on your Self-Location (the place where you felt your sense of self or consciousness was spatially located) write: SELF

Examples:
INDIFFERENCE: 9 (NO BODY).
SURPRISE: 2 (1,2).
SERENITY: 10 (NO BODY).

72. Tick the right answer

Une seule réponse possible.

- ( ) In your first NDE space (NDE part 1) you only had 6 Self-Locations. *Passer à la question 92*
- ( ) In your first NDE space (NDE part 1) you had 7 or more than 7 Self-Locations.

g. NDE Part 1: First NDE Space Self-Location Number 7

73. 1. In your first NDE space at your Self-Location number 7 (S-L7), your emotions/ feelings were positive, negative or neutral? (you can tick more than one answer). *

Plusieurs réponses possibles.

- [ ] Positive.
- [ ] Negative.
- [ ] Neutral.

74. **2.** If in your first NDE space at your Self-Location number 7 (S-L7), your emotion(s) / feeling(s) were positive, do as follow:

A. NAME the positive emotion/feeling you had.

B. Write a number between 0 and 10 to express the INTENSITY LEVEL of each of the positive emotion/feeling you have felt.

C. Look at the Body Maps page 11-12 of the Notice :

*If you have felt some positive emotions on specific part(s) of the physical body, write in brackets the specific part(s) to indicate the positive emotions(s) location.

*If you did not feel some positive emotions on your physical body write: NO BODY

*If you felt some positive emotions all over your physical body write: ALL BODY

*If you felt some positive emotions on your Self-Location (the place where you felt your sense of self or consciousness was spatially located) write: SELF

Example:
JOY: 5 (25).
WELL-BEING: 4 (ALL BODY)
HAPPINESS: 7 (1, 2, 3, 23).

75. 3. If in your first NDE space at your Self-Location number 7 (S-L7), your emotion(s)/ feeling(s) were negative, do as follow:

A. NAME the negative emotion/feeling you had.

B. Write a number between 0 and 10 to express the INTENSITY LEVEL of each of the negative emotion/feeling you have felt.
C. Look at the Body Maps page 11-12 of the Notice :

*If you have felt some negative emotions on specific part(s) of the body, write in brackets the specific part(s) to indicate the negative emotions(s) location.

*If you did not feel some negative emotions on your physical body write: NO BODY

*If you felt some negative emotions all over your physical body write: ALL BODY

*If you felt some negative emotions on your Self-Location (the place where you felt your sense of self or consciousness was spatially located) write: SELF

Examples :
FEAR: 8 (SELF).
SADNESS: 6 (SELF).
DISGUST: 3 (SELF).

76. 4. If in your first NDE space at your Self-Location number 7 (S-L7), your emotion(s) / feeling(s) were neutral, do as follow:

A. NAME the neutral emotion/feeling you had.

B. Choose a number between 0 and 10 to express the INTENSITY LEVEL of each of the neutral emotion/feeling you have felt.

C. Look at the Body Maps page 11-12 of the Notice :
*If you have felt some neutral emotions on specific part(s) of the body, write the specific part(s) to indicate the neutral emotions(s) location.
*If you did not feel some neutral emotions on your physical body write: NO BODY
*If you felt some neutral emotions all over your physical body write: ALL BODY
*If you felt some neutral emotions on your Self-Location (the place where you felt your sense of self or consciousness was spatially located) write: SELF

Examples:
INDIFFERENCE: 9 (NO BODY).
SURPRISE: 2 (1,2).
SERENITY: 10 (NO BODY).

77. Tick the right answer

Une seule réponse possible.

- In your first NDE space (NDE part 1) you only had 7 Self-Locations. *Passer à la question 92*
- In your first NDE space (NDE part 1) you had 8 or more than 8 Self-Locations.

h. NDE Part 1: First NDE Space Self-Location Number 8

78. 1. In your first NDE space at your Self-Location number 8 (S-L8), your emotions/ feelings were positive, negative or neutral? *
(you can tick more than one answer).

Plusieurs réponses possibles.

- [ ] Positive.
- [ ] Negative.
- [ ] Neutral.

79. 2. If in your first NDE space at your Self-Location number 8 (S-L8), your emotion(s) / feeling(s) were positive, do as follow:

A. NAME the positive emotion/feeling you had.

B. Write a number between 0 and 10 to express the INTENSITY LEVEL of each of the positive emotion/feeling you have felt.

C. Look at the Body Maps page 11-12 of the Notice :

*If you have felt some positive emotions on specific part(s) of the physical body, write in brackets the specific part(s) to indicate the positive emotions(s) location.

*If you did not feel some positive emotions on your physical body write: NO BODY

*If you felt some positive emotions all over your physical body write: ALL BODY

*If you felt some positive emotions on your Self-Location (the place where you felt your sense of self or consciousness was spatially located) write: SELF

Example:
JOY: 5 (25).
WELL-BEING: 4 (ALL BODY)
HAPPINESS: 7 (1, 2, 3, 23).

80. 3. If in your first NDE space at your Self-Location number 8 (S-L8), your emotion(s)/ feeling(s) were negative, do as follow:

A. NAME the negative emotion/feeling you had.

B. Write a number between 0 and 10 to express the INTENSITY LEVEL of each of the negative emotion/feeling you have felt.
C. Look at the Body Maps page 11-12 of the Notice :

*If you have felt some negative emotions on specific part(s) of the body, write in brackets the specific part(s) to indicate the negative emotions(s) location.

*If you did not feel some negative emotions on your physical body write: NO BODY

*If you felt some negative emotions all over your physical body write: ALL BODY

*If you felt some negative emotions on your Self-Location (the place where you felt your sense of self or consciousness was spatially located) write: SELF

Examples :
FEAR: 8 (SELF).
SADNESS: 6 (SELF).
DISGUST: 3 (SELF).

81. 4. If in your first NDE space at your Self-Location number 8 (S-L8), your emotion(s) / feeling(s) were neutral, do as follow:

A. NAME the neutral emotion/feeling you had.

B. Choose a number between 0 and 10 to express the INTENSITY LEVEL of each of the neutral emotion/feeling you have felt.

C. Look at the Body Maps page 11-12 of the Notice :
*If you have felt some neutral emotions on specific part(s) of the body, write the specific part(s) to indicate the neutral emotions(s) location.
*If you did not feel some neutral emotions on your physical body write: NO BODY
*If you felt some neutral emotions all over your physical body write: ALL BODY
*If you felt some neutral emotions on your Self-Location (the place where you felt your sense of self or consciousness was spatially located) write: SELF

Examples:
INDIFFERENCE: 9 (NO BODY).
SURPRISE: 2 (1,2).
SERENITY: 10 (NO BODY).

82. Tick the right answer

Une seule réponse possible.

- In your first NDE space (NDE part 1) you only had 8 Self-Locations. *Passer à la question 92*
- In your first NDE space (NDE part 1) you had 9 or more than 9 Self-Locations.

i. NDE Part 1: First NDE Space Self-Location Number 9

83. 1. In your first NDE space at your Self-Location number 9 (S-L9), your emotions/ feelings were positive, negative or neutral? (you can tick more than one answer). *

Plusieurs réponses possibles.

- [ ] Positive.
- [ ] Negative.
- [ ] Neutral.

84. 2. If in your first NDE space at your Self-Location number 9 (S-L9), your emotion(s) / feeling(s) were positive, do as follow:
A. NAME the positive emotion/feeling you had.

B. Write a number between 0 and 10 to express the INTENSITY LEVEL of each of the positive emotion/feeling you have felt.

C. Look at the Body Maps page 11-12 of the Notice :

*If you have felt some positive emotions on specific part(s) of the physical body, write in brackets the specific part(s) to indicate the positive emotions(s) location.

*If you did not feel some positive emotions on your physical body write: NO BODY

*If you felt some positive emotions all over your physical body write: ALL BODY

*If you felt some positive emotions on your Self-Location (the place where you felt your sense of self or consciousness was spatially located) write: SELF

Example:
JOY: 5 (25).
WELL-BEING: 4 (ALL BODY)
HAPPINESS: 7 (1, 2, 3, 23).

85. 3. If in your first NDE space at your Self-Location number 9 (S-L9), your emotion(s)/ feeling(s) were negative, do as follow:

A. NAME the negative emotion/feeling you had.

B. Write a number between 0 and 10 to express the INTENSITY LEVEL of each of the negative emotion/feeling you have felt.
C. Look at the Body Maps page 11-12 of the Notice :

*If you have felt some negative emotions on specific part(s) of the body, write in brackets the specific part(s) to indicate the negative emotions(s) location.

*If you did not feel some negative emotions on your physical body write: NO BODY

*If you felt some negative emotions all over your physical body write: ALL BODY

*If you felt some negative emotions on your Self-Location (the place where you felt your sense of self or consciousness was spatially located) write: SELF

Examples :
FEAR: 8 (SELF).
SADNESS: 6 (SELF).
DISGUST: 3 (SELF).

86. 4. If in your first NDE space at your Self-Location number 9 (S-L9), your emotion(s) / feeling(s) were neutral, do as follow:

A. NAME the neutral emotion/feeling you had.

B. Choose a number between 0 and 10 to express the INTENSITY LEVEL of each of the neutral emotion/feeling you have felt.

C. Look at the Body Maps page 11-12 of the Notice :
*If you have felt some neutral emotions on specific part(s) of the body, write the specific part(s) to indicate the neutral emotions(s) location.
*If you did not feel some neutral emotions on your physical body write: NO BODY
*If you felt some neutral emotions all over your physical body write: ALL BODY
*If you felt some neutral emotions on your Self-Location (the place where you felt your sense of self or consciousness was spatially located) write: SELF

Examples:
INDIFFERENCE: 9 (NO BODY).
SURPRISE: 2 (1,2).
SERENITY: 10 (NO BODY).

87. Tick the right answer

Une seule réponse possible.

- [ ] In your first NDE space (NDE part 1) you only had 9 Self-Locations. *Passer à la question 92*
- [ ] In your first NDE space (NDE part 1) you had 10 Self-Locations.

j. NDE Part 1: First NDE Space Self-Location Number 10

88. 1. In your first NDE space at your Self-Location number 10 (S-L10), your emotions/ feelings were positive, negative or neutral? *
(you can tick more than one answer).

Plusieurs réponses possibles.

- [ ] Positive.
- [ ] Negative.
- [ ] Neutral.

89. 2. If in your first NDE space at your Self-Location number 10 (S-L10), your emotion(s) / feeling(s) were positive, do as follow:

A. NAME the positive emotion/feeling you had.

B. Write a number between 0 and 10 to express the INTENSITY LEVEL of each of the positive emotion/feeling you have felt.

C. Look at the Body Maps page 11-12 of the Notice :

*If you have felt some positive emotions on specific part(s) of the physical body, write in brackets the specific part(s) to indicate the positive emotions(s) location.

*If you did not feel some positive emotions on your physical body write: NO BODY

*If you felt some positive emotions all over your physical body write: ALL BODY

*If you felt some positive emotions on your Self-Location (the place where you felt your sense of self or consciousness was spatially located) write: SELF

Example:
JOY: 5 (25).
WELL-BEING: 4 (ALL BODY)
HAPPINESS: 7 (1, 2, 3, 23).

90. 3. If in your first NDE space at your Self-Location number 10 (S-L10), your emotion(s)/ feeling(s) were negative, do as follow:

A. NAME the negative emotion/feeling you had.

B. Write a number between 0 and 10 to express the INTENSITY LEVEL of each of the negative emotion/feeling you have felt.
C. Look at the Body Maps page 11-12 of the Notice :

*If you have felt some negative emotions on specific part(s) of the body, write in brackets the specific part(s) to indicate the negative emotions(s) location.

*If you did not feel some negative emotions on your physical body write: NO BODY

*If you felt some negative emotions all over your physical body write: ALL BODY

*If you felt some negative emotions on your Self-Location (the place where you felt your sense of self or consciousness was spatially located) write: SELF

Examples :
FEAR: 8 (SELF).
SADNESS: 6 (SELF).
DISGUST: 3 (SELF).

91. 4. If in your first NDE space at your Self-Location number 10 (S-L10), your emotion(s) / feeling(s) were neutral, do as follow:

A. NAME the neutral emotion/feeling you had.

B. Choose a number between 0 and 10 to express the INTENSITY LEVEL of each of the neutral emotion/feeling you have felt.

C. Look at the Body Maps page 11-12 of the Notice :
*If you have felt some neutral emotions on specific part(s) of the body, write the specific part(s) to indicate the neutral emotions(s) location.
*If you did not feel some neutral emotions on your physical body write: NO BODY
*If you felt some neutral emotions all over your physical body write: ALL BODY
*If you felt some neutral emotions on your Self-Location (the place where you felt your sense of self or consciousness was spatially located) write: SELF

Examples:
INDIFFERENCE: 9 (NO BODY).
SURPRISE: 2 (1,2).
SERENITY: 10 (NO BODY).

NDE Part 1: Final

92. Now you have finished answering the NDE PART 1 questionnaire.

BOX A: If you had only 1 NDE PART, we thank you very much for the time you took to participate in our questionnaire and we kindly ask you to check Box A to go to the final section of our questionnaire.

BOX B:
If you had 2 NDE PARTS or more than 2 NDE PARTS, please check Box B to proceed to the next stage of our NDE questionnaire.

Une seule réponse possible.

- [ ] A: Go to the final section of our questionnaire.
  Passer à la question 276
- [ ] B: Proceeding to the next stage.

NDE Part 2: Second Space Graphic Questionnaire

*Important note: in this drawing document we ask you to describe and draw ONLY YOUR NDE PART 2.
