# Supplementary material for: The perceptual geometry and chromaticity of near-death experience spaces
Source: Front Psychol. 2026 Oct 15;17:1760389. doi: 10.3389/fpsyg.2026.1760389 (PMC13716671; doi:10.3389/fpsyg.2026.1760389)
Supplement: Supplementary file 4 [file Data_Sheet_4.pdf]

NDE Preliminary Questionnaire

* Indique une question obligatoire

1. Adresse e-mail *

Information & Consent

To be eligible to participate to our study you must be fluent in English be 18 years old or more, with no mental disabilities or need for a guardian.
Your participation in this research is completely voluntary and at any time of your choice you can remove yourself from the study, to end up your participation you will have to just close your browser.
Your identity details will be anonymized and any connection between your identity and the results will be kept confidential. At no time your name will be used or any identifying information revealed. However if you have agreed to participate in this questionnaire, the researcher will need to collect your e-mail to send you the results of the two NDE validation scales you will have completed in our preliminary questionnaire. If you have reached a cut-off score identifying you as Near-Death Experiencer (NDER), you will then receive in the same e-mail a link to proceed to the second part of our questionnaire untitled “Extracorporeal affects and bodily sensations in Near-Death Experiences Spaces”.

You will be able to fill in the questionnaire alone or if you wish you will be able to do it under the guidance of the researcher by scheduling an online appointment with the researcher at lerner.france@gmail.com. In any problem related to the research, you can also contact the researcher at lerner.france@gmail.com for further consultation. In the unlikely event that completing the questionnaire would trigger distressing affects. The participant should report it to the researcher which will refer him/her to a qualified and licensed professional counselor. I have read the above information regarding this research study on Near-Death Experiences (NDEs) and I have fully understood the above and I consent to participate in the study which if I am selected to proceed to the next stage will include the online self reporting questionnaire untitled: “Extracorporeal affects and bodily sensations in Near-Death Experiences Spaces”.

2. I hereby declare by ticking the box below that the information I am giving are true and I give my consent to participate in the study of my own free will and that I understood all. *

Plusieurs réponses possibles.

☐ Yes

Personal Details

3. Family name & Given name (pseudonym optional) *

4. Date of birth *

Exemple : 7 janvier 2019

5. Place of birth *

6. Gender: how do you identify? *

Une seule réponse possible.

- Male
- Female
- Non-binary
- Prefer to self-describe (please fill the section below)

7. Self-describe (gender)

OUT- OF BODY EXPERIENCE
DEFINITION:

An Out-of Body Experience (OBE) is a phenomenon where you perceived the physical world and/or your physical body from an extracorporeal point of view with the impression of being separating or departing from your physical body or/and the physical world and reuniting or returning to your physical body or/and the physical world.
A Dissociative Out-of Body Experience (OBE) is a phenomenon where you perceived the physical world and/or your physical body from an extracorporeal point of view with the impression of being separating or departing from your physical body or/and the physical world.

An Associative Out-of Body Experience (OBE) is a phenomenon where you perceived the physical world and/or your physical body from an extracorporeal point of view with the impression of reuniting with or returning into your physical body or/and the physical world.

8. What type of OBE did you have according to the above definition? *

Une seule réponse possible.

- Dissociative
- Associative
- Dissociative and Associative
- None

NEAR-DEATH EXPERIENCES (NDEs)
Near-Death Experiences (NDEs) are deep transcendental mental experiences typically following a life-threatening situation (e.g. cardiac arrest, coma, traumatic brain injury) but also associated with circumstances that do not jeopardize life (e.g. meditation, migraine).

9. How many NDE did you have? *

10. If you had more then one NDE please give the date of each NDE in its chronological order;
if you only had one NDE give its date. *

If you had more than one NDE choose to answer our questionnaire the NDE which you consider to be the most significant for you in terms of 3 criterions:

1.The vividness of its visual imagery.
2.The intensity of its emotional content.
3.The activity of your Self-Motion during the NDE. (What we imply by the term Self-Motion (S-M) is the motion sensation your self experiences when you had the impression of moving while being out-of his physical body in an extracorporeal space and/or from an extracorporeal point of view. An extracorporeal point of view is the point of view from which you perceive the physical world and/or your physical body while having the impression of being out-of your physical body).
*Important note: from now on you will have to describe in our survey ONLY This NDE. Please write again in your answer to the question below the date of this specific NDE you chose to describe.

11. Please give the date of the NDE you have chosen to describe in this questionnaire *

Exemple : 7 janvier 2019

12. IF YOU WERE HOSPITALIZED, please specify: *
1-The etiology you were diagnosed with by the doctors. Namely, the name given to you to describe your medical condition (e.g. coma, cardiac arrest, brain injury, traumatic complication, suicide).
2- The name and place of the department or unit where you were hospitalized: (e.g. neurologic unit, intensive care).

IF YOU WERE NOT HOSPITALIZED, please specify:
1- The physiological set of causes which have led to your NDE, (eg. migraine, meditation, REM sleep, syncope, recreational drug or alcohol).
2-The psychological frame of mind you were in at the time.

____

13. Would you qualify your NDE as a life-threatening event or as a non life-threatening event? *

Une seule réponse possible.

- [ ] Life-threatening event
- [ ] Non life-threatening event

14. Would you qualify your NDE as a pleasant experience or an unpleasant experience. *

Une seule réponse possible.

- [ ] Pleasant experience
- [ ] Unpleasant experience

NEAR-DEATH EXPERIENCES (NDE) VALIDATION SCALES
We use two self-report scales to assess, measure and quantify the depth of your NDE. The Greyson Scale (16 Items) and The NDE-C Scale (20 Items).

The Greyson Scale (16 Items)

15. 1. Did time seem to speed up or slow down? *

Une seule réponse possible.

- No.
- Time seemed to go faster or slower than usual.
- Everything seemed to be happening at once; or time stopped or lost all meaning.

16. 2. Were your thoughts speeded up? *

Une seule réponse possible.

- No.
- Faster than usual.
- Incredibly fast.

17. 3. Did scenes from your past come back to you? *

Une seule réponse possible.

- No.
- I remembered many past events.
- My past flashed before me, out of my control.

18. 4. Did you suddenly seem to understand everything? *

Une seule réponse possible.

- No.
- Everything about myself or others.
- Everything about the universe.

19. 5. Did you have a feeling of peace or pleasantness? *

Une seule réponse possible.

- No.
- Relief or calmness.
- Incredible peace or pleasantness.

20. 6. Did you have a feeling of joy? *

Une seule réponse possible.

- No.
- Happiness.
- Incredible joy.

21. 7. Did you feel a sense of harmony or unity with the universe? *

Une seule réponse possible.

- No.
- I felt no longer in conflict with nature.
- I felt united or one with the world.

22. 8. Did you see, or feel surrounded by, a brilliant light? *

Une seule réponse possible.

- No.
- An unusually bright light.
- A light clearly of mystical or other-worldly origin.

23. 9. Were your senses more vivid than usual? *

Une seule réponse possible.

- No.
- More vivid than usual.
- Incredibly more vivid.

24. 10. Did you seem to be aware of things going on elsewhere, as if by extrasensory perception (ESP)? *

Une seule réponse possible.

- No.
- Yes, but the facts have not been checked out.
- Yes, and the facts have been checked out.

25. 11. Did scenes from the future come to you? *

Une seule réponse possible.

- No.
- Scenes from my personal future.
- Scenes from the world's future.

26. 12. Did you feel separated from your body? *

Une seule réponse possible.

- No.
- I lost awareness of my body.
- I clearly left my body and existed outside it.

27. 13. Did you seem to enter some other, unearthly world? *

Une seule réponse possible.

- No.
- Some unfamiliar and strange place.
- A clearly mystical or unearthly realm.

28. 14. Did you seem to encounter a mystical being or presence, or hear an unidentifiable voice? *

Une seule réponse possible.

- No.
- I heard a voice I could not identify.
- I encountered a definite being, or a voice clearly of mystical or unearthly origin.

29. 15. Did you see deceased or religious spirits? *

Une seule réponse possible.

- No.
- I sensed their presence.
- I actually saw them.

30. 16. Did you come to a border or point of no return? *

Une seule réponse possible.

- No.
- I came to a definite conscious decision to “return” to life.
- I came to a barrier that I was not permitted to cross; or was “sent back” against my will.

The NDE-C scale (20 items)

The Near-Death Experience Content (NDE-C) scale (Martial et., al, 2020). We use the self-report NDE-C Scale (20 items) to assess, measure and quantify the depth of your NDE. We would like you to answer the 20 following statements according to your feelings and thoughts at the time of the experience (not before, nor after) by choosing the answer that seems the most appropriate (only ONE answer by statement is allowed).
Every experience or sensation varies in intensity, which is why we would like you to specify the intensity of your experience using the rating scale (from 1 to 4) described below for each statement if, on the contrary, you did not experience the phenomenon described in the statement, please choose use the rating scale below. If you happen to have experienced several times the same phenomenon during the experience, please answer by considering the most striking phenomenon.

Rating Scale:
0 - No
1 - Slightly.
2 - Moderately.
3 - Strongly; equivalent in degree to any other strong experience lived so far.
4 - Extremely; more than any other time in my life and stronger than 3.

31. **1. Your perception of time was altered.** *

Une seule réponse possible.

0	1	2	3	4
◯	◯	◯	◯	◯

32. **2. Your thoughts speeded up.** *

Une seule réponse possible.

0	1	2	3	4
◯	◯	◯	◯	◯

33. **3. You heard one or several voices which did not have any material incarnation.** *

Une seule réponse possible.

0	1	2	3	4
◯	◯	◯	◯	◯

34. **4. You had the feeling of suddenly understanding everything about yourself, the others and/or the universe.** *

Une seule réponse possible.

0	1	2	3	4
◯	◯	◯	◯	◯

35. **5. You had a feeling of peace and/or well-being.** *

Une seule réponse possible.

0	1	2	3	4
◯	◯	◯	◯	◯

36. **6. You felt a sense of harmony or unity, as if you belonged to a larger whole.** *

Une seule réponse possible.

0	1	2	3	4
◯	◯	◯	◯	◯

37. **7. You saw or felt surrounded by a bright light without any determined material origin.** *

Une seule réponse possible.

0	1	2	3	4
◯	◯	◯	◯	◯

38. **8. You experienced unusual sensations (sight, hearing, smell, touch and/or taste).** *

Une seule réponse possible.

0	1	2	3	4
◯	◯	◯	◯	◯

39. **9. You were aware of things beyond what your senses can usually perceive.** *

Une seule réponse possible.

0	1	2	3	4
◯	◯	◯	◯	◯

40. **10. You gained insightful knowledge about the future.** *

Une seule réponse possible.

0	1	2	3	4
◯	◯	◯	◯	◯

41. **11. You had the impression of being outside of, or separated from your own body.** *

Une seule réponse possible.

0	1	2	3	4
◯	◯	◯	◯	◯

42. **12. You had the sensation of leaving the earthly world or of entering a new dimension and/or environment.** *

Une seule réponse possible.

0	1	2	3	4
◯	◯	◯	◯	◯

43. **13. You saw or lived again events from your past.** *

Une seule réponse possible.

0	1	2	3	4
◯	◯	◯	◯	◯

44. **14. You encountered a presence and/or an entity (who might be deceased).** *

Une seule réponse possible.

0	1	2	3	4
◯	◯	◯	◯	◯

45. **15. You had a feeling of non-existence, of being in a total void, and/or of fear.** *

Une seule réponse possible.

0	1	2	3	4
◯	◯	◯	◯	◯

46. **16. You came close to a border and/or point of no return.** *

Une seule réponse possible.

0	1	2	3	4
◯	◯	◯	◯	◯

47. **17. You made the decision, or were forced, to come back from the experience.** *

Une seule réponse possible.

0	1	2	3	4
◯	◯	◯	◯	◯

48. **18. You had the feeling of dying and/or being dead.** *

Une seule réponse possible.

0	1	2	3	4
◯	◯	◯	◯	◯

49. **19. You saw or entered a gateway (for instance a tunnel or a door).** *

Une seule réponse possible.

0	1	2	3	4
◯	◯	◯	◯	◯

50. **20. You sense that the experience cannot be described adequately in words.** *

Une seule réponse possible.

0	1	2	3	4
◯	◯	◯	◯	◯

We warmly thank you for your generous participation and for the time you took to complete our preliminary survey.

Ce contenu n'est ni rédigé, ni cautionné par Google.

Google Forms
