# Supplementary material for: The perceptual geometry and chromaticity of near-death experience spaces
Source: Front Psychol. 2026 Oct 15;17:1760389. doi: 10.3389/fpsyg.2026.1760389 (PMC13716671; doi:10.3389/fpsyg.2026.1760389)
Supplement: Supplementary file 5 [file Data_Sheet_5.pdf]

Legend of Pictograms Representing NDE Spaces

NDE Spaces of Conical Type: A Shapes

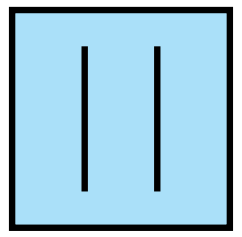
A1

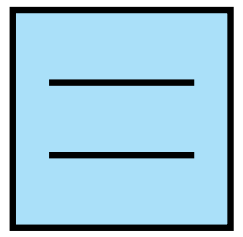
A2

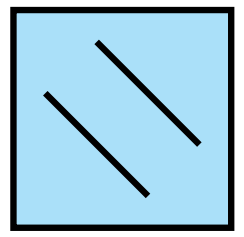
A3

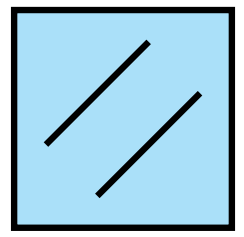
A4

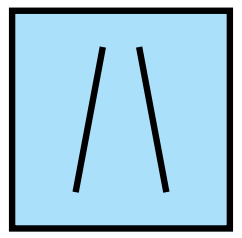
A5

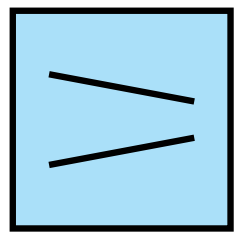
A6

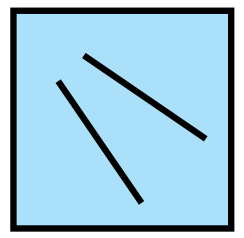
A7

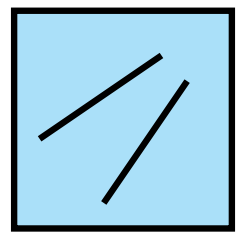
A8

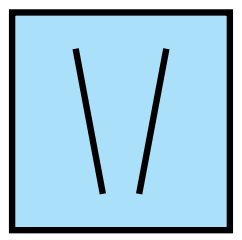
A9

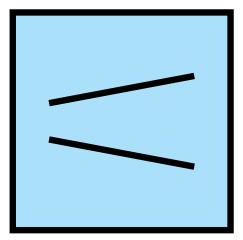
A10

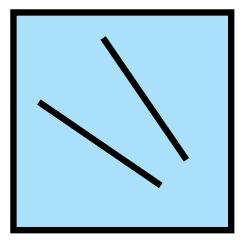
A11

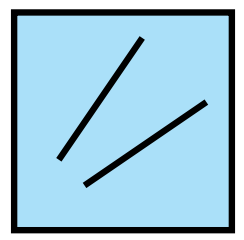
A12

NDE Spaces of Elliptic Type: B and C Shapes

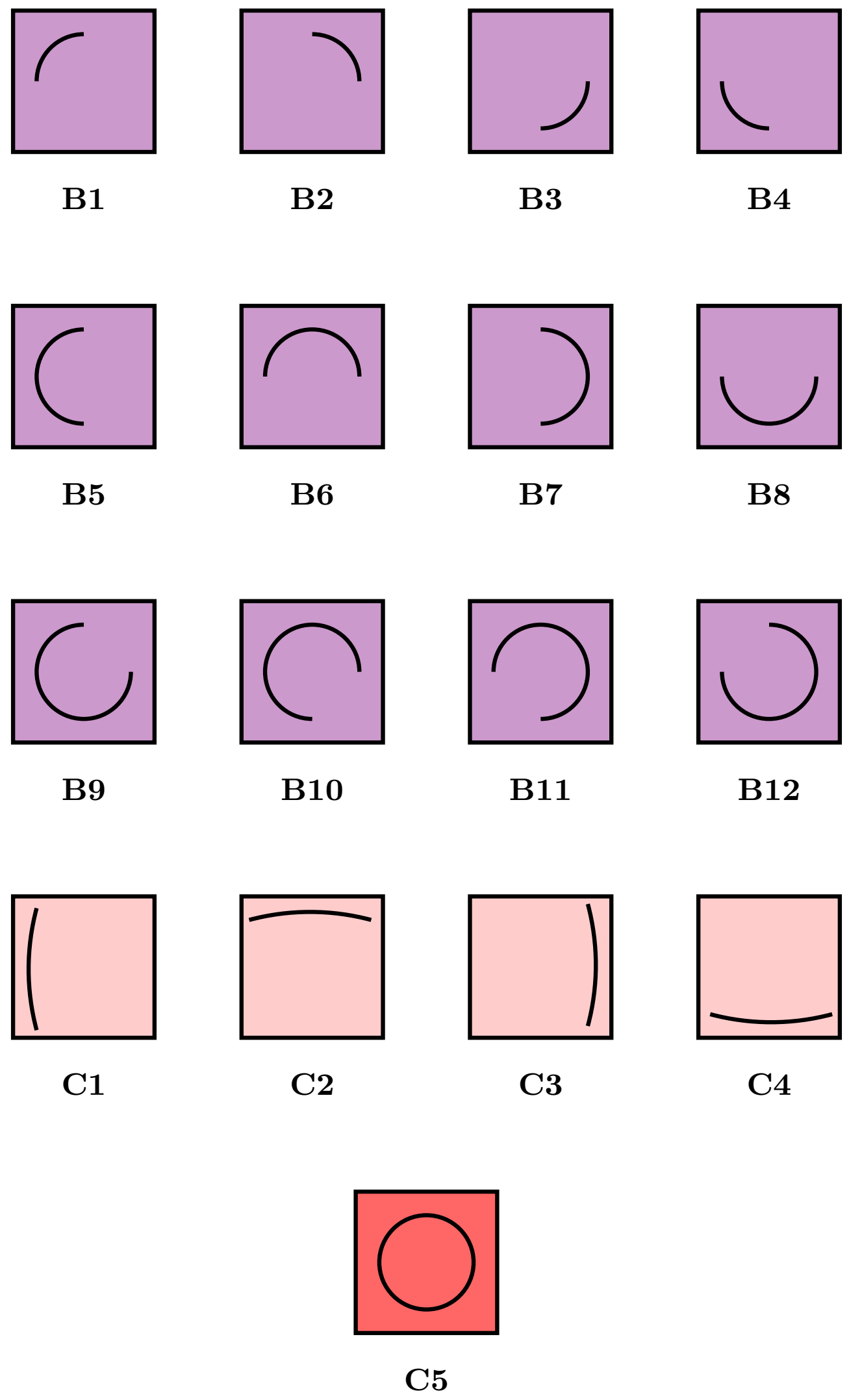

Exceptions

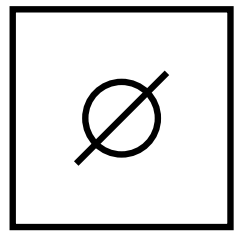

No space

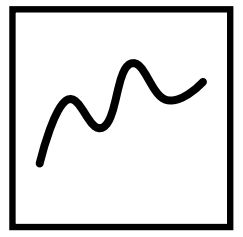

Other space (freely hand-drawn)

**Subject Number**	**OBE Dissociative**	**Sequence of NDE Spaces**	**OBE Associative**
1	YES	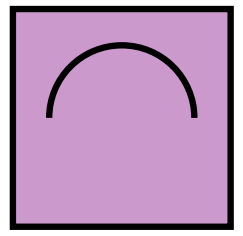 **B6** 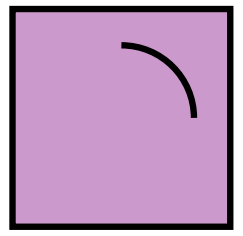 **B2** 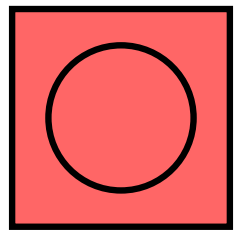 **C5** 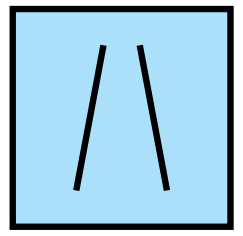 **A5**	NO
2	NO	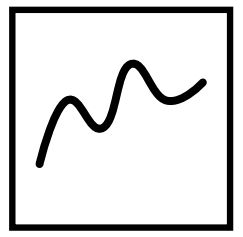 **Other space (freely hand-drawn)**	NO
3	NO	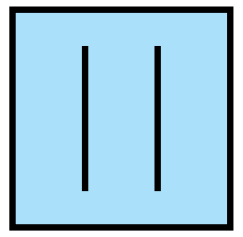 **A1** 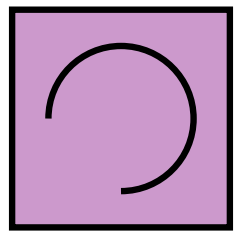 **B11**	NO
4	YES	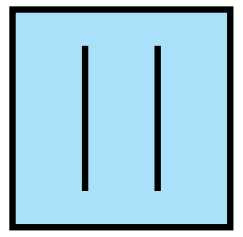 **A1** 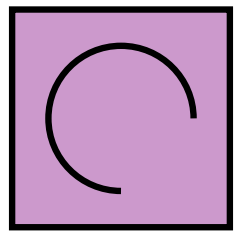 **B10**	NO
5	YES	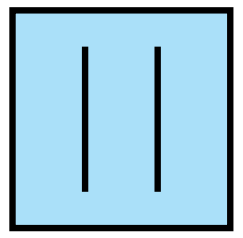 **A1** 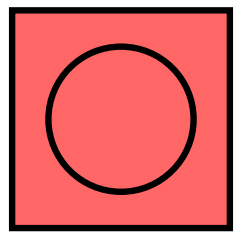 **C5**	YES

Subject Number	OBE Dissociative	Sequence of NDE Spaces	OBE Associative
6	YES	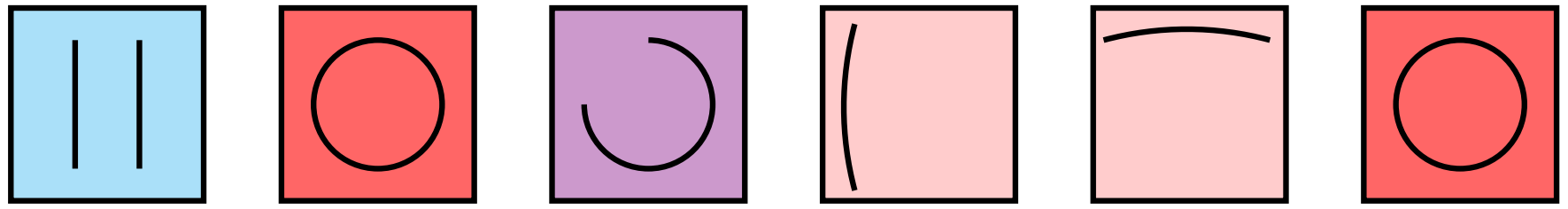 A1 C5 B12 C1 C2 C5	YES
7	YES	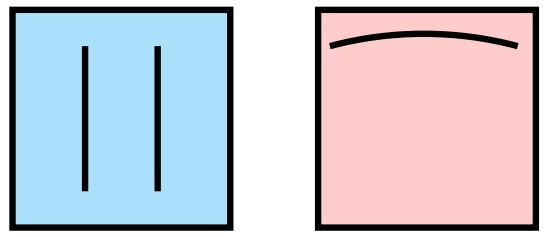 A1 C2	NO
8	YES	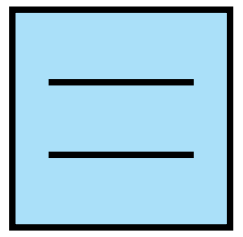 A2	NO
9	NO	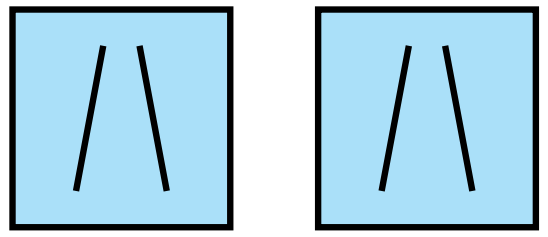 A5 A5	YES
10	NO	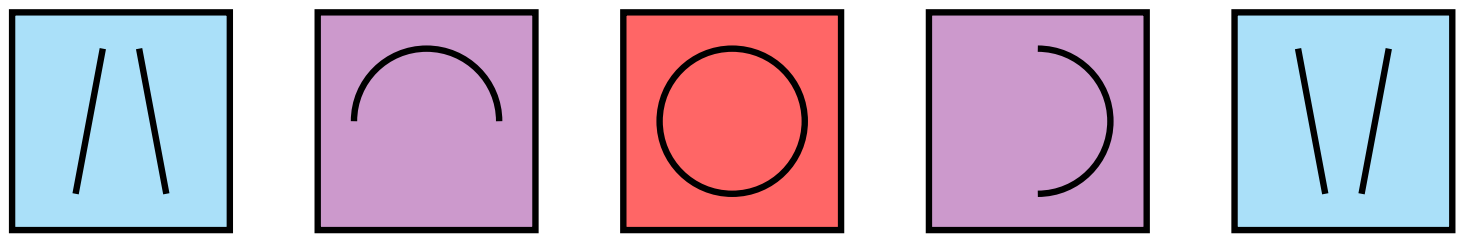 A5 B6 C5 B7 A9	YES

Subject Number	OBE Dissociative	Sequence of NDE Spaces	OBE Associative
11	YES	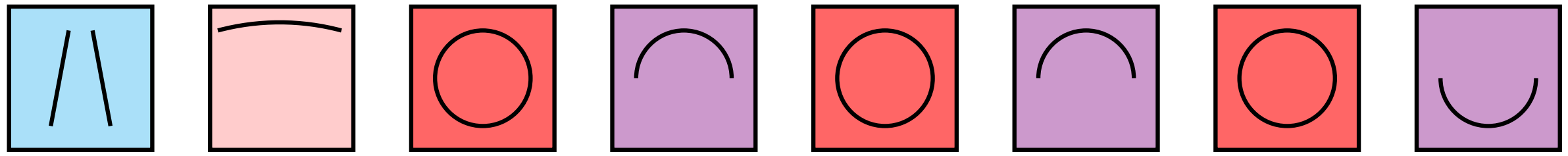 **A5 C2 C5 B6 C5 B6 C5 B8**	YES
12	NO	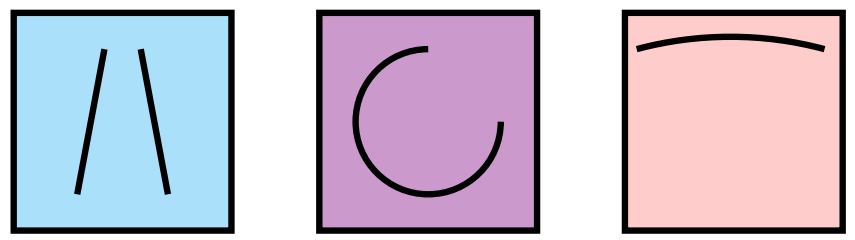 **A5 B9 C2**	YES
13	YES	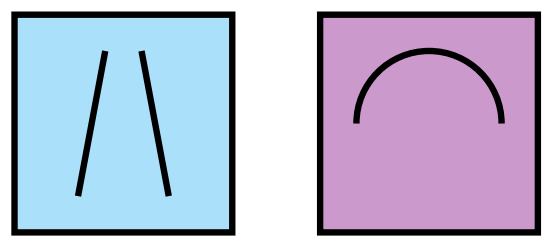 **A5 B6**	NO
14	YES	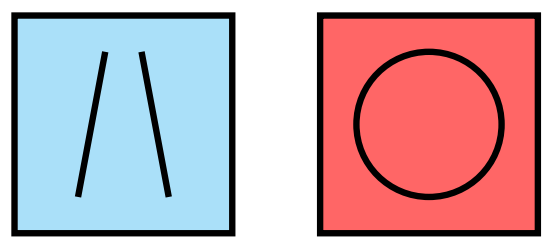 **A5 C5**	YES
15	NO	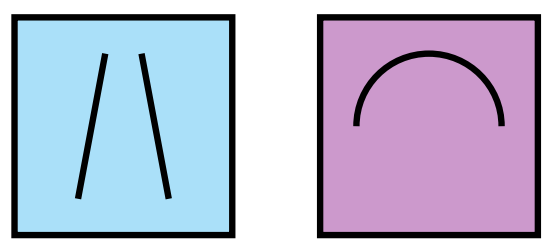 **A5 B6**	YES

Subject Number	OBE Dissociative	Sequence of NDE Spaces	OBE Associative
16	NO	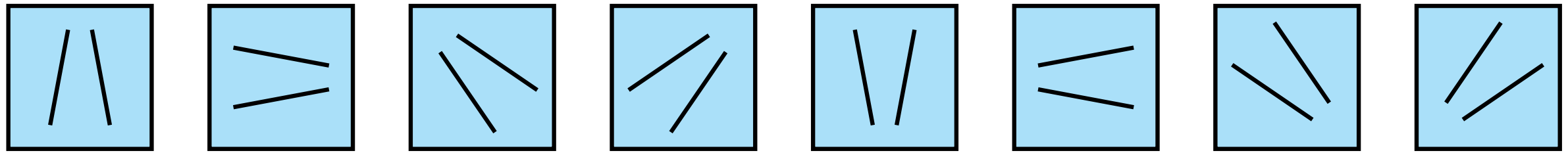 A5 A6 A7 A8 A9 A10 A11 A12	NO
17	YES	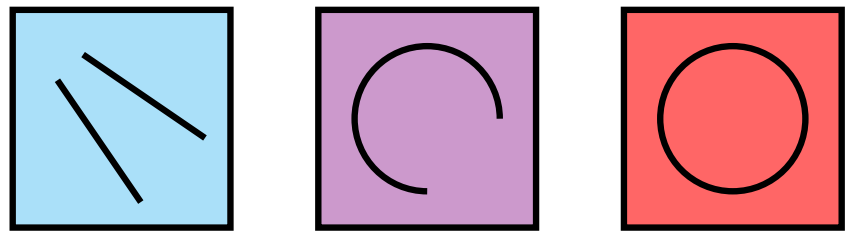 A7 B10 C5	NO
18	NO	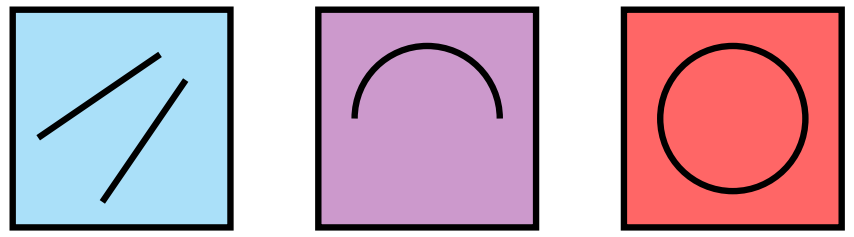 A8 B6 C5	NO
19	YES	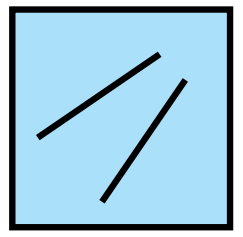 A8	NO
20	NO	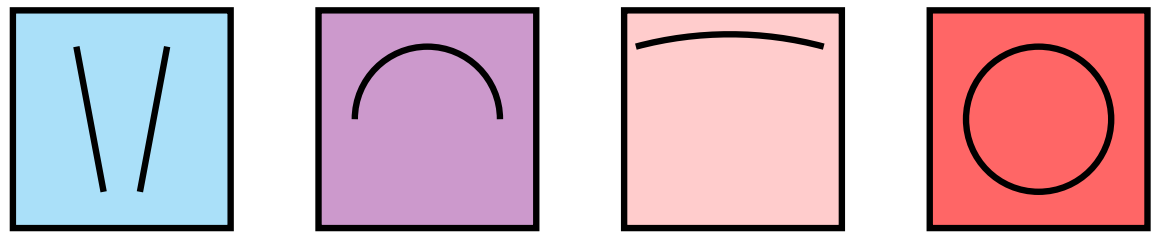 A9 B6 C2 C5	YES

Subject Number	OBE Dissociative	Sequence of NDE Spaces	OBE Associative
21	NO	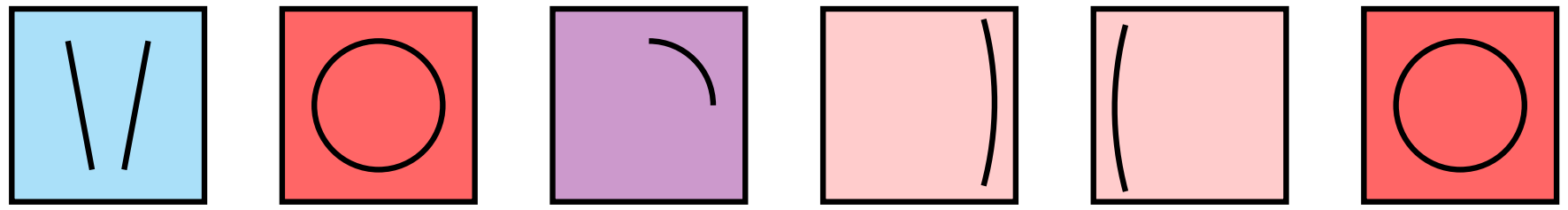 **A9** **C5** **B2** **C3** **C1** **C5**	YES
22	NO	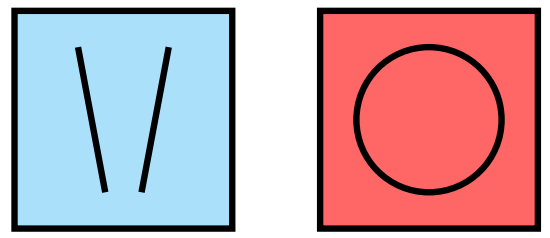 **A9** **C5**	NO
23	NO	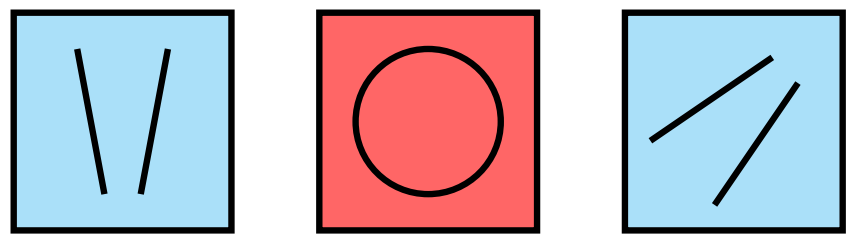 **A9** **C5** **A8**	NO
24	YES	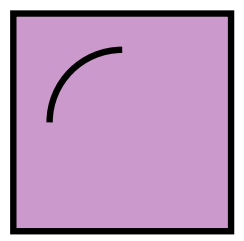 **B1**	YES
25	NO	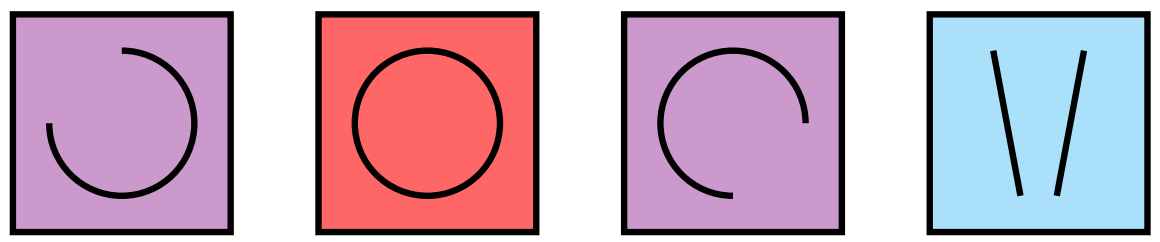 **B12** **C5** **B10** **A9**	NO

Subject Number	OBE Dissociative	Sequence of NDE Spaces	OBE Associative
26	NO	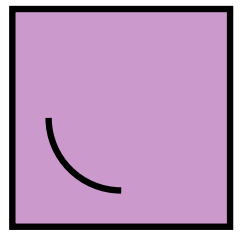 **B4**	NO
27	NO	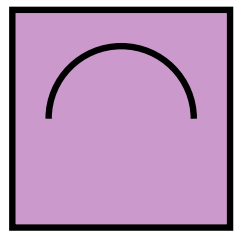 **B6**	NO
28	NO	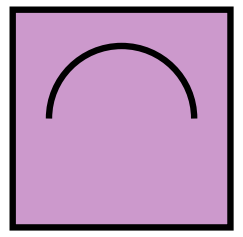 **B6** 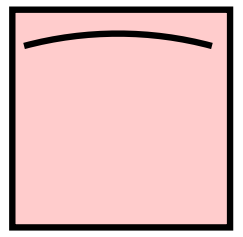 **C2** 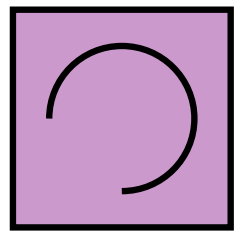 **B11**	NO
29	YES	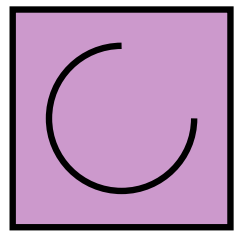 **B9** 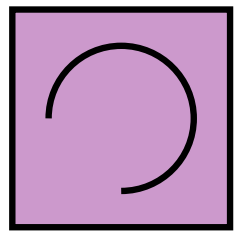 **B11** 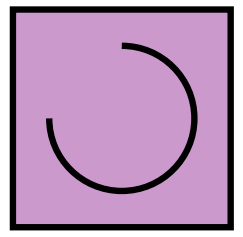 **B12**	YES
30	YES	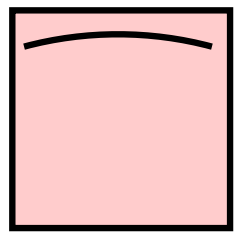 **C2**	YES

**Subject Number**	**OBE Dissociative**	**Sequence of NDE Spaces**	**OBE Associative**
31	NO	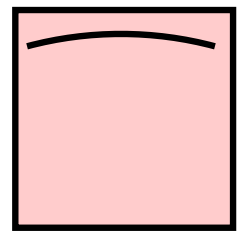 **C2**	NO
32	NO	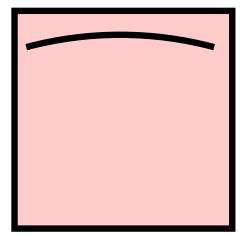 **C2** 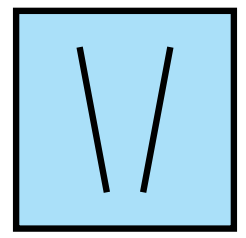 **A9**	NO
33	YES	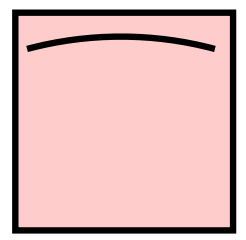 **C2**	YES
34	YES	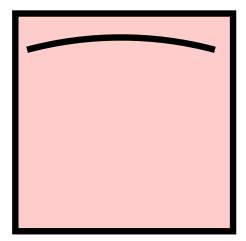 **C2**	YES
35	NO	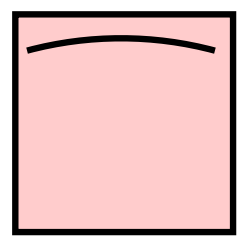 **C2**	NO

**Subject Number**	**OBE Dissociative**	**Sequence of NDE Spaces**	**OBE Associative**
36	NO	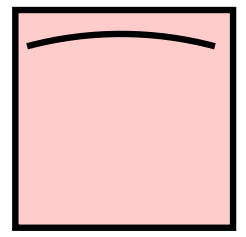 **C2**	NO
37	YES	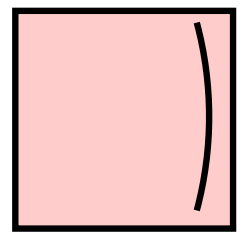 **C3**	NO
38	NO	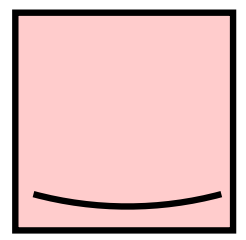 **C4**	NO
39	YES	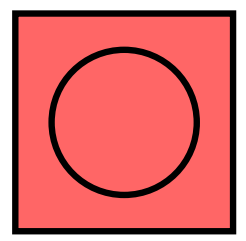 **C5**	YES
40	NO	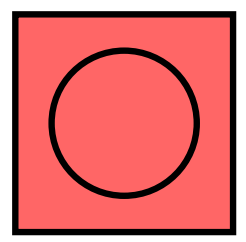 **C5**	YES

**Subject Number**	**OBE Dissociative**	**Sequence of NDE Spaces**	**OBE Associative**
41	YES	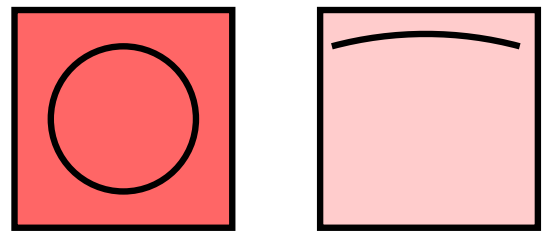 **C5** **C2**	NO
42	YES	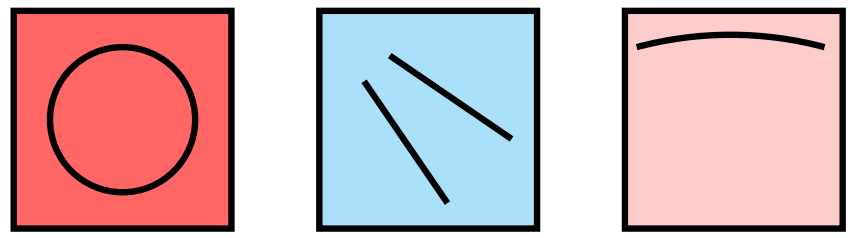 **C5** **A7** **C2**	YES
43	NO	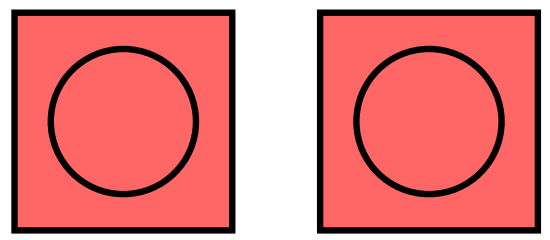 **C5** **C5**	NO
44	YES	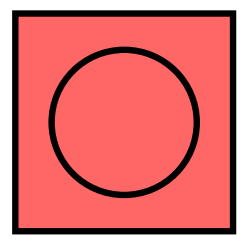 **C5**	YES
45	NO	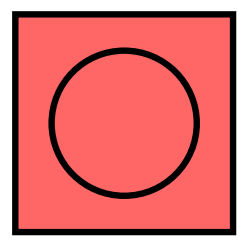 **C5**	NO

Subject Number	OBE Dissociative	Sequence of NDE Spaces	OBE Associative
46	NO	**C5** **C5**	YES
47	YES	**C5** **B9** **B10** **B11** **B12**	NO
48	NO	**C5**	NO
49	NO	**No space**	YES
50	YES	**Other space (freely hand-drawn)** **Other space (freely hand-drawn)**	NO
