# Supplementary material for: The perceptual geometry and chromaticity of near-death experience spaces
Source: Front Psychol. 2026 Oct 15;17:1760389. doi: 10.3389/fpsyg.2026.1760389 (PMC13716671; doi:10.3389/fpsyg.2026.1760389)
Supplement: Supplementary file 7 [file Data_Sheet_7.pdf]

Out-of-Body and Near-Death Experience Graphic Representations

A Study of 50 Subjects

Subject 1

Sequence: part/space

1 OBE + 4 NDE

- OBE Dissociative
- B6
- B2
- C5
- A5

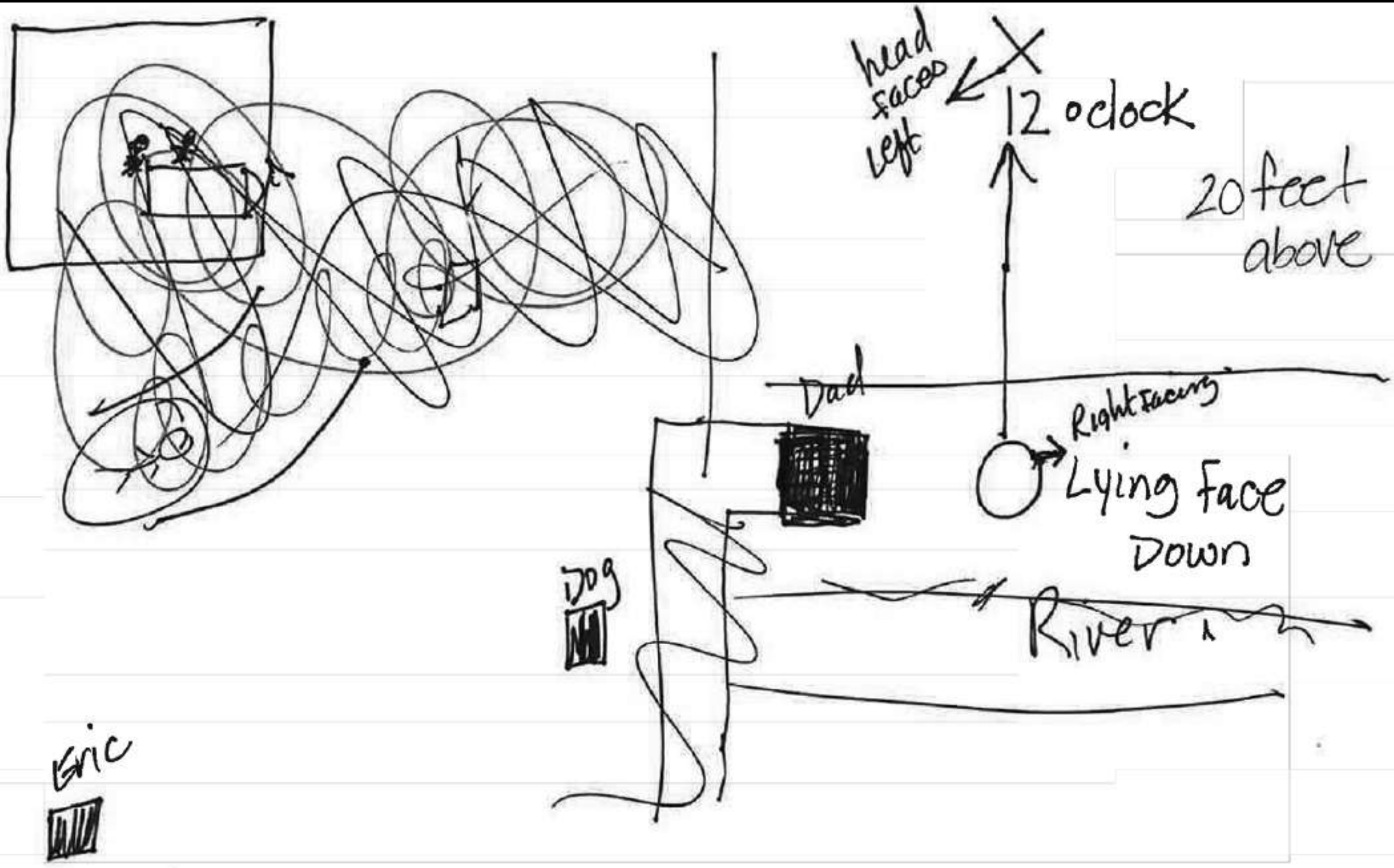

• OBE Dissociative

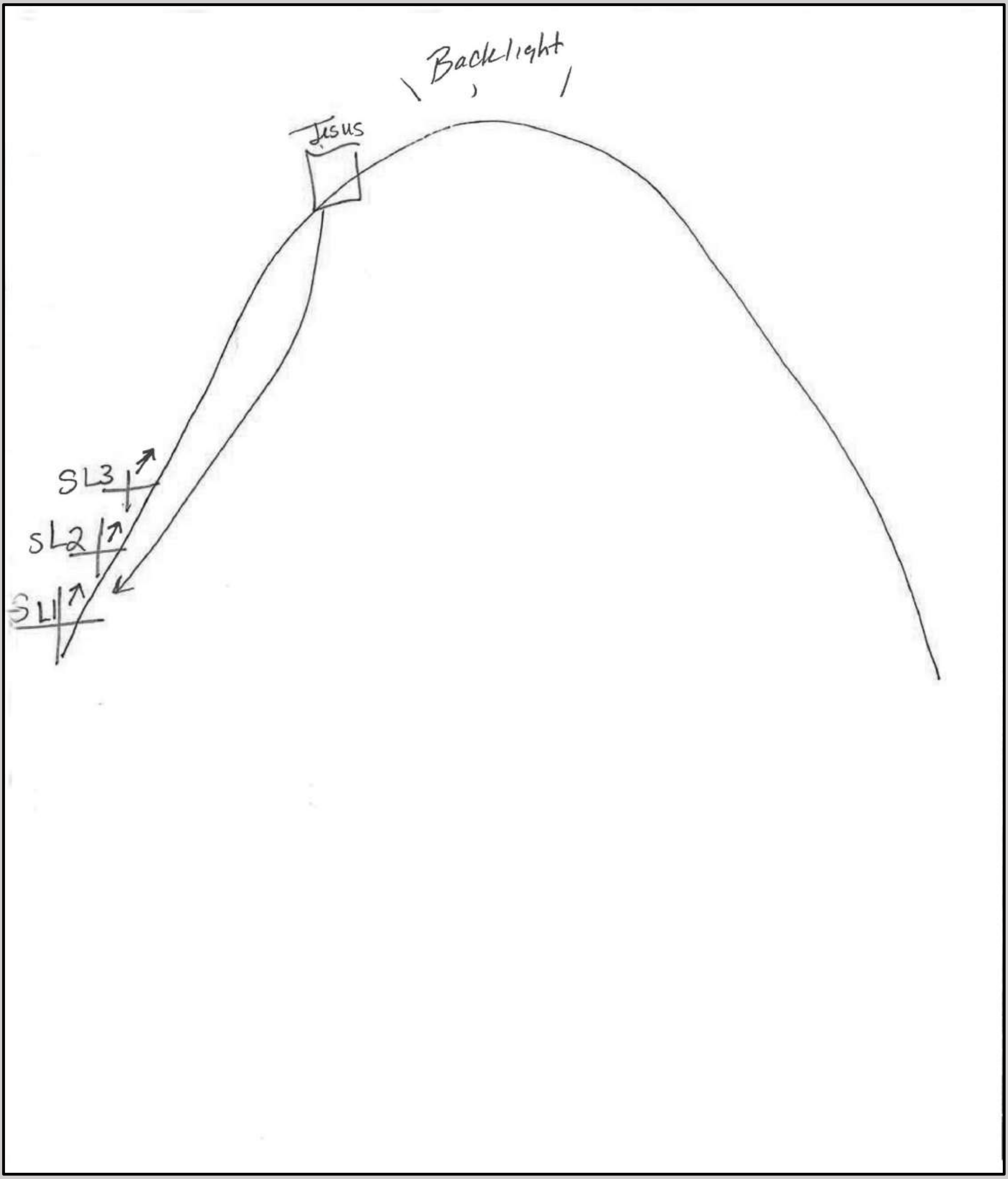

- B6

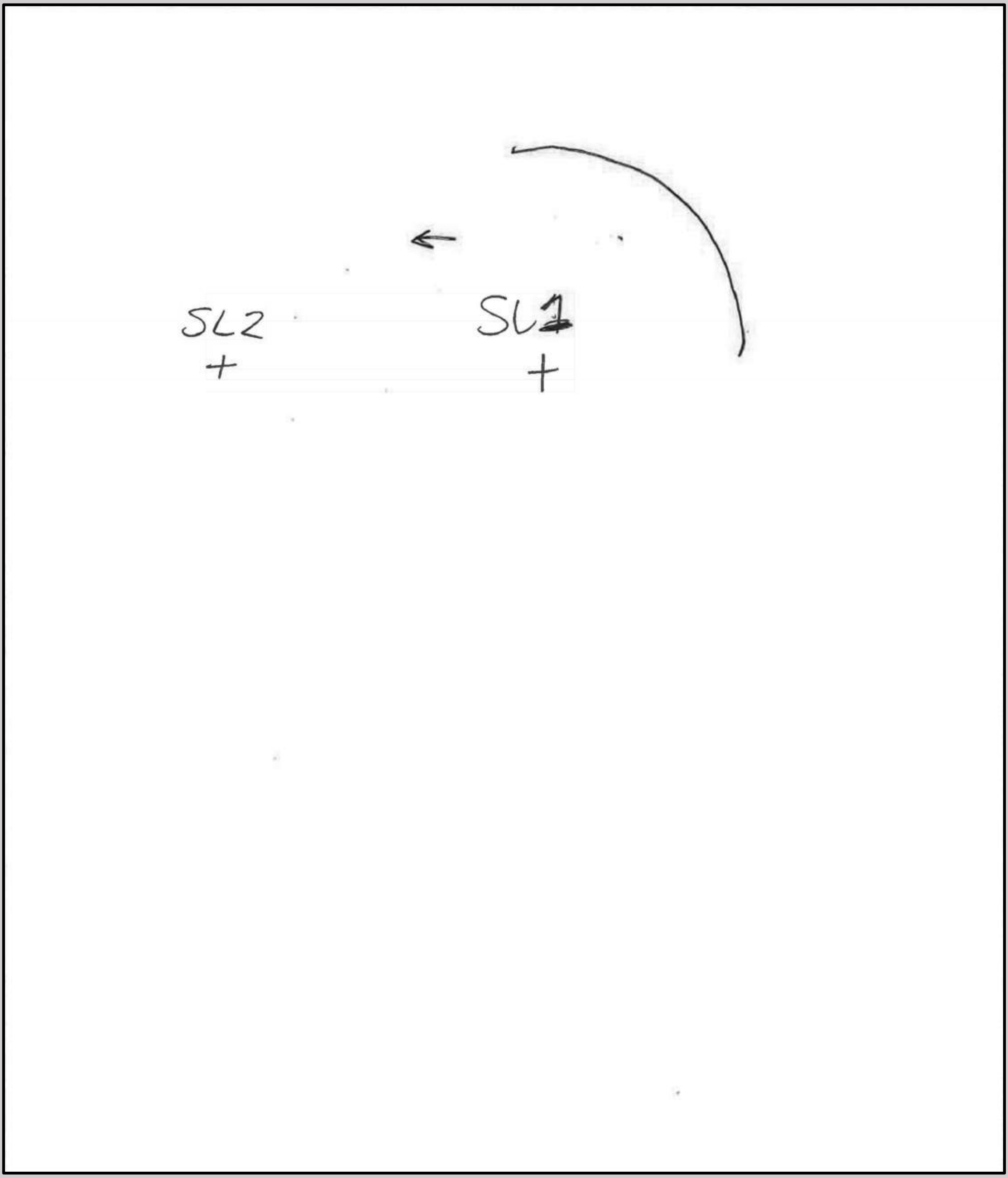

• B2

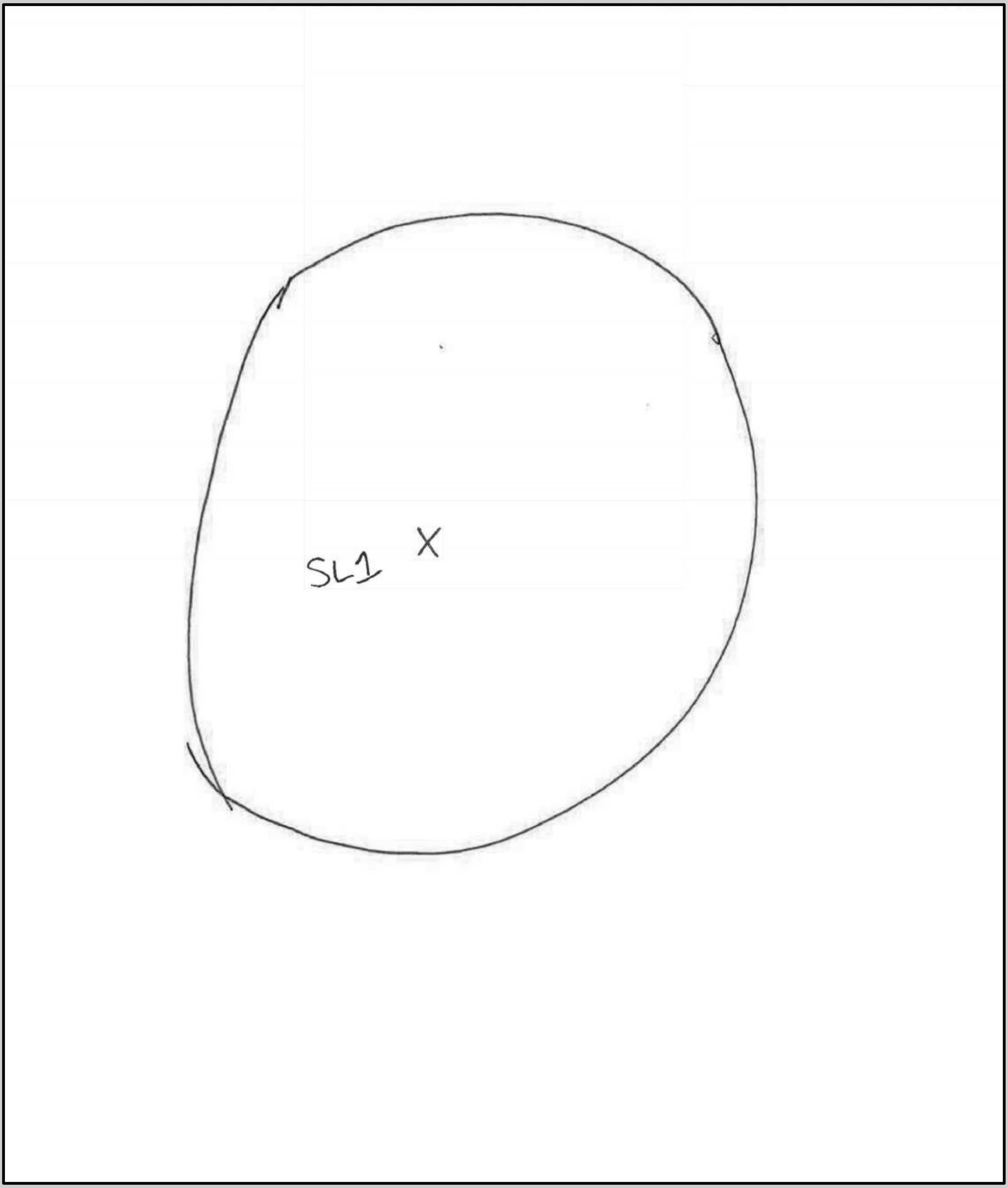

- C5

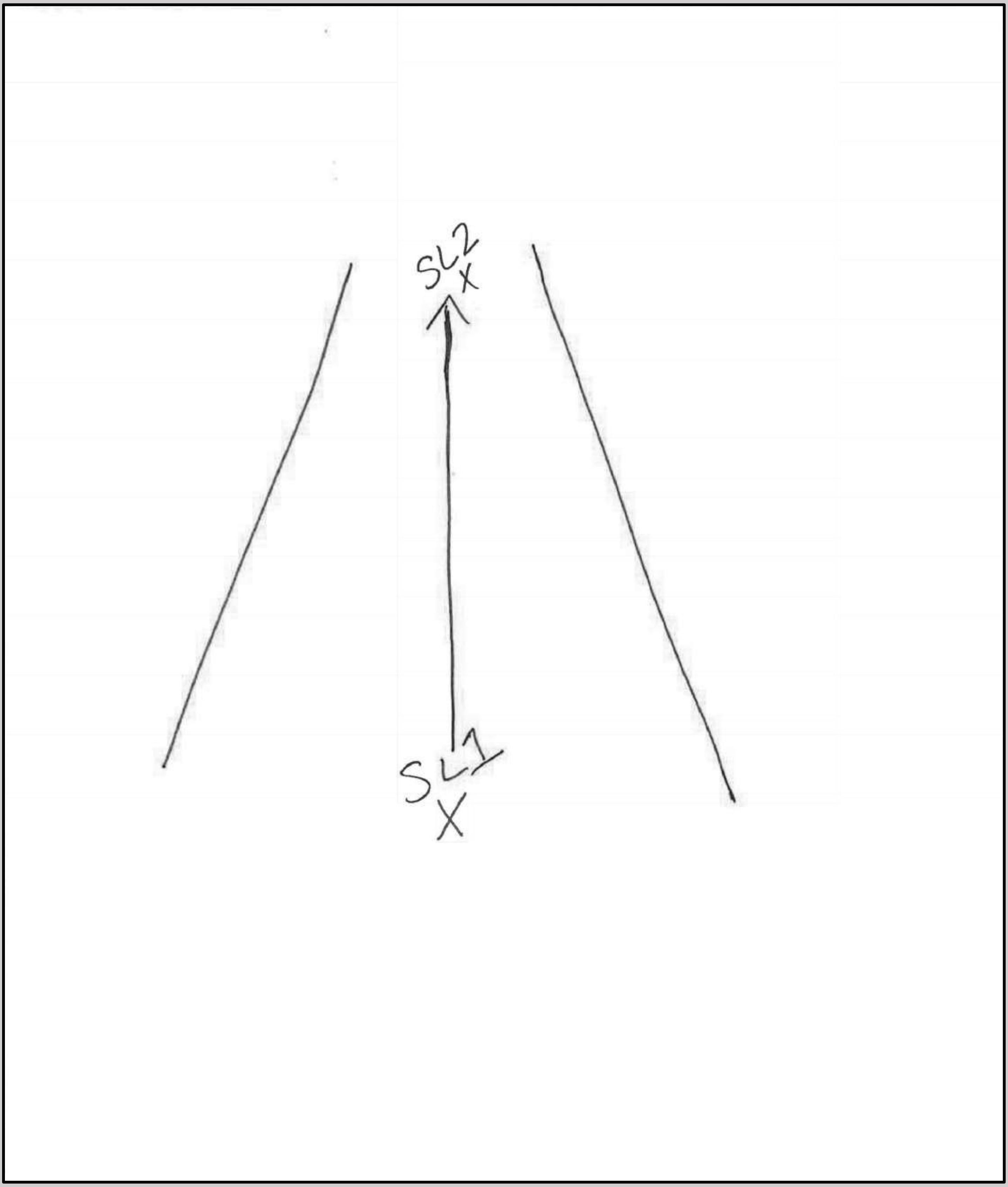

• A5

Subject 2

Sequence: part/space

1 NDE

- Other space (freely hand-drawn)

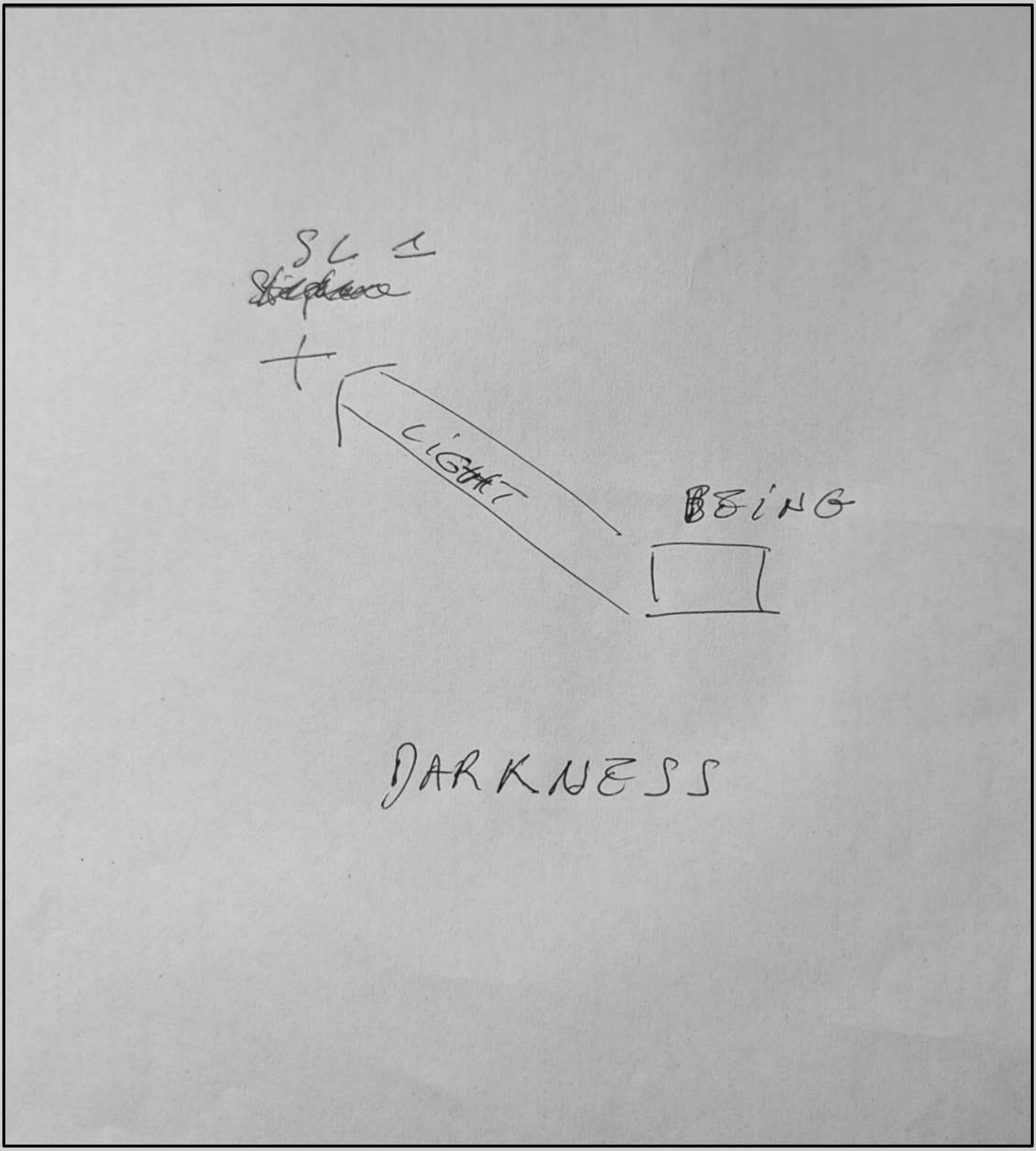

- Other space (freely hand-drawn)

Subject 3

Sequence: part/space

2 NDE

- A1
- B11

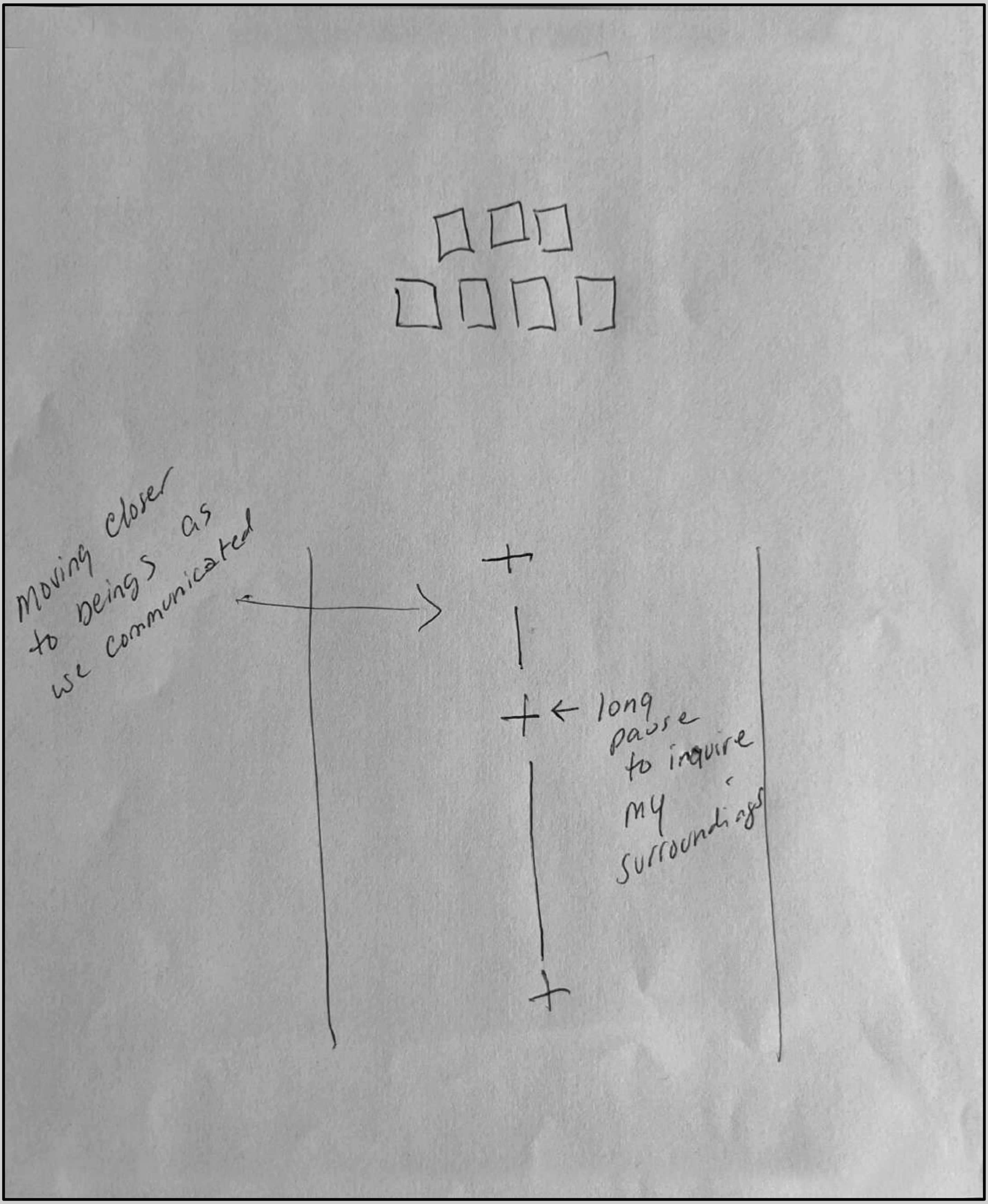
Moving closer to beings as we communicated
long pause to inquire my surroundings

• A1

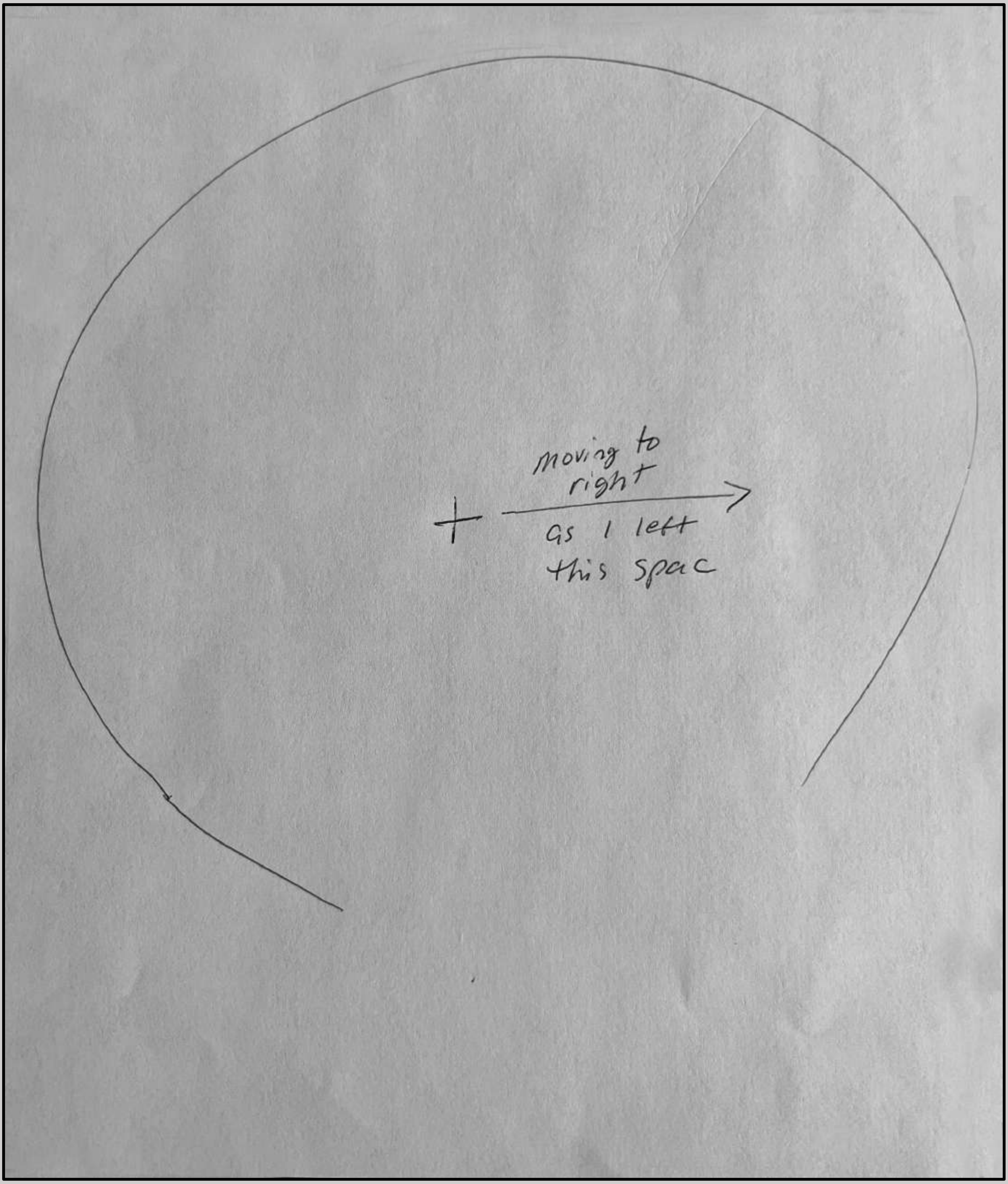

• B11

Subject 4

Sequence: part/space

1 OBE + 2 NDE

- OBE Dissociative
- A1
- B10

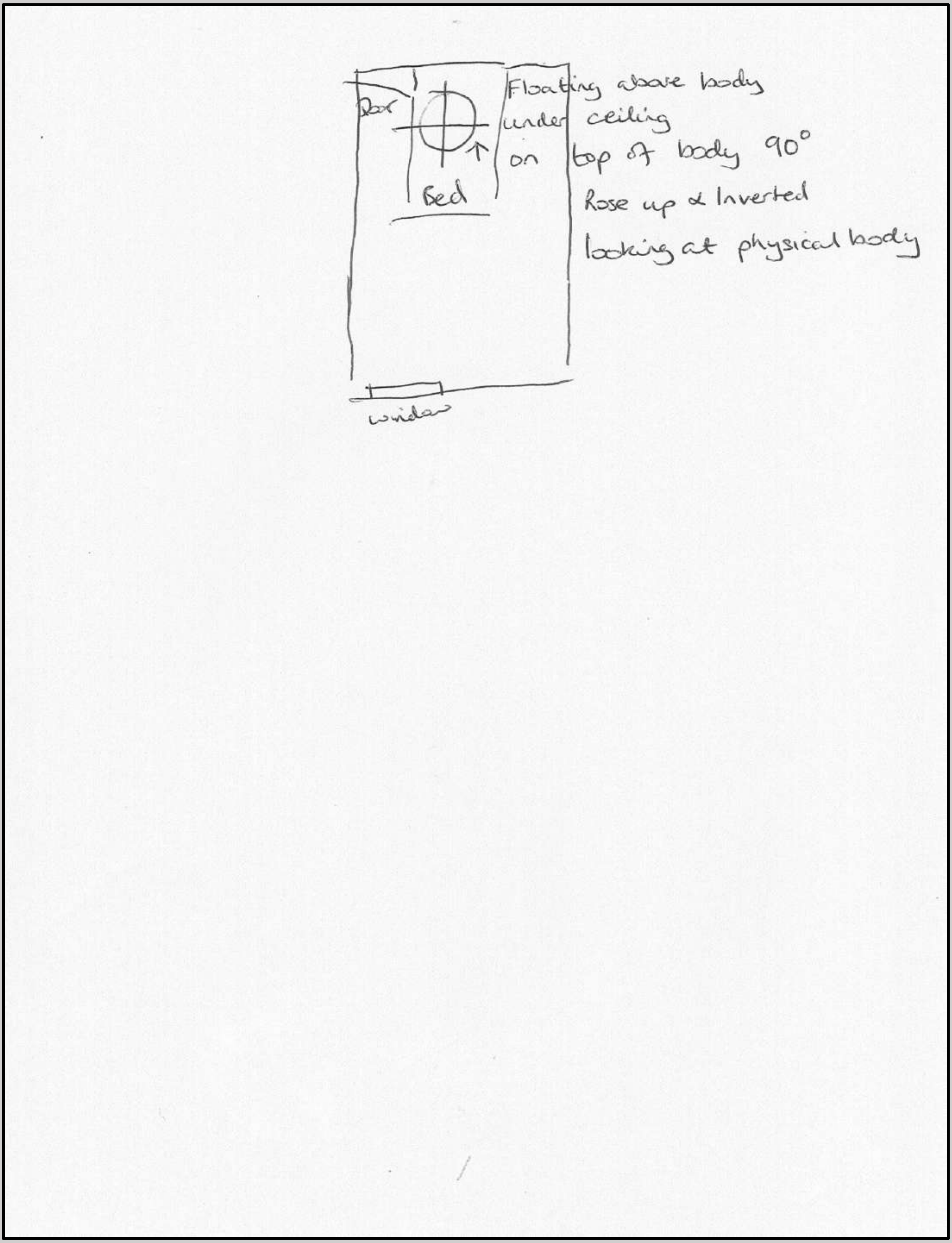

- OBE Dissociative

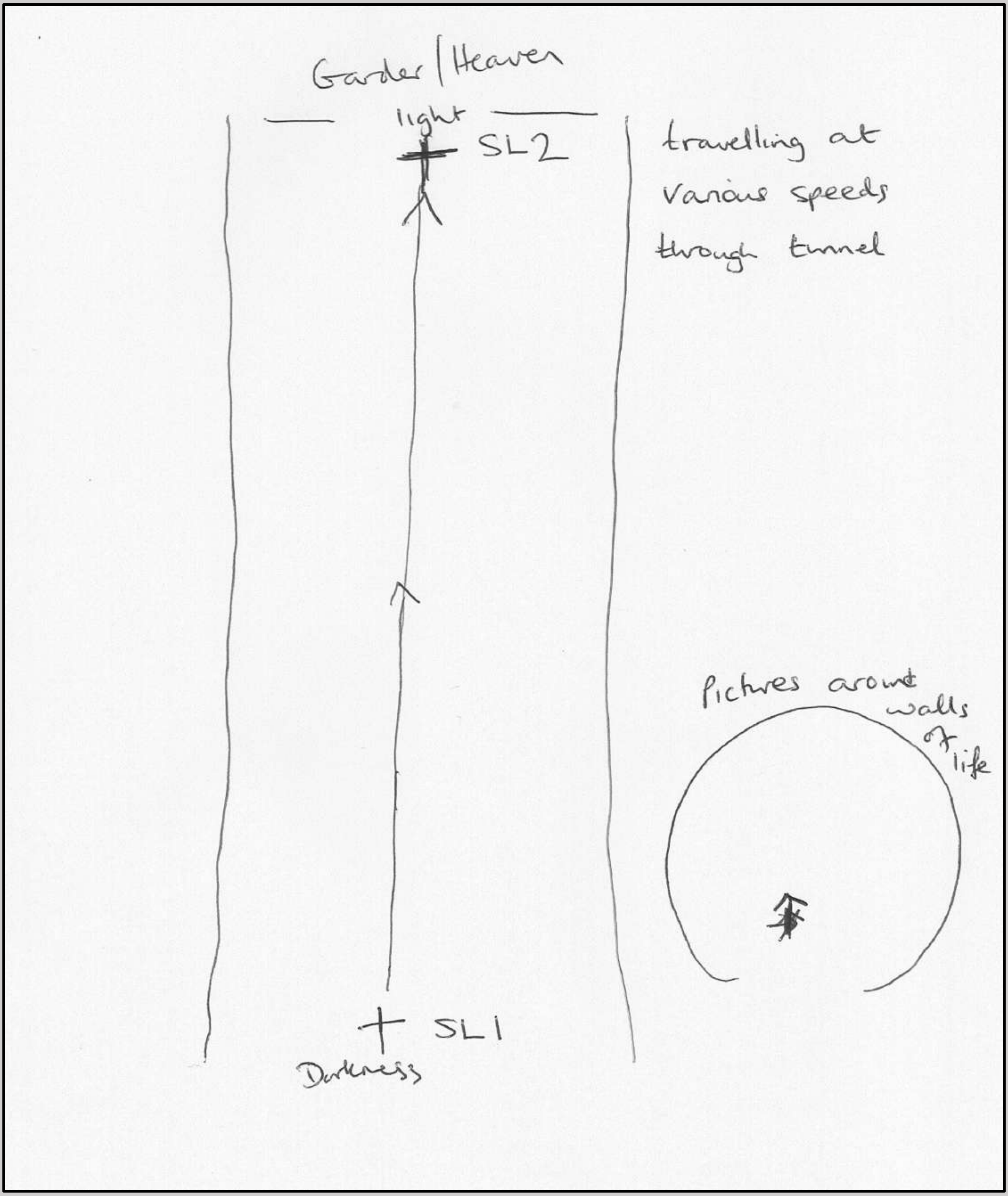
Garden | Heaven
light
SL2
travelling at
various speeds
through tunnel
Pictures around walls of life
SL1
Darkness

- A1

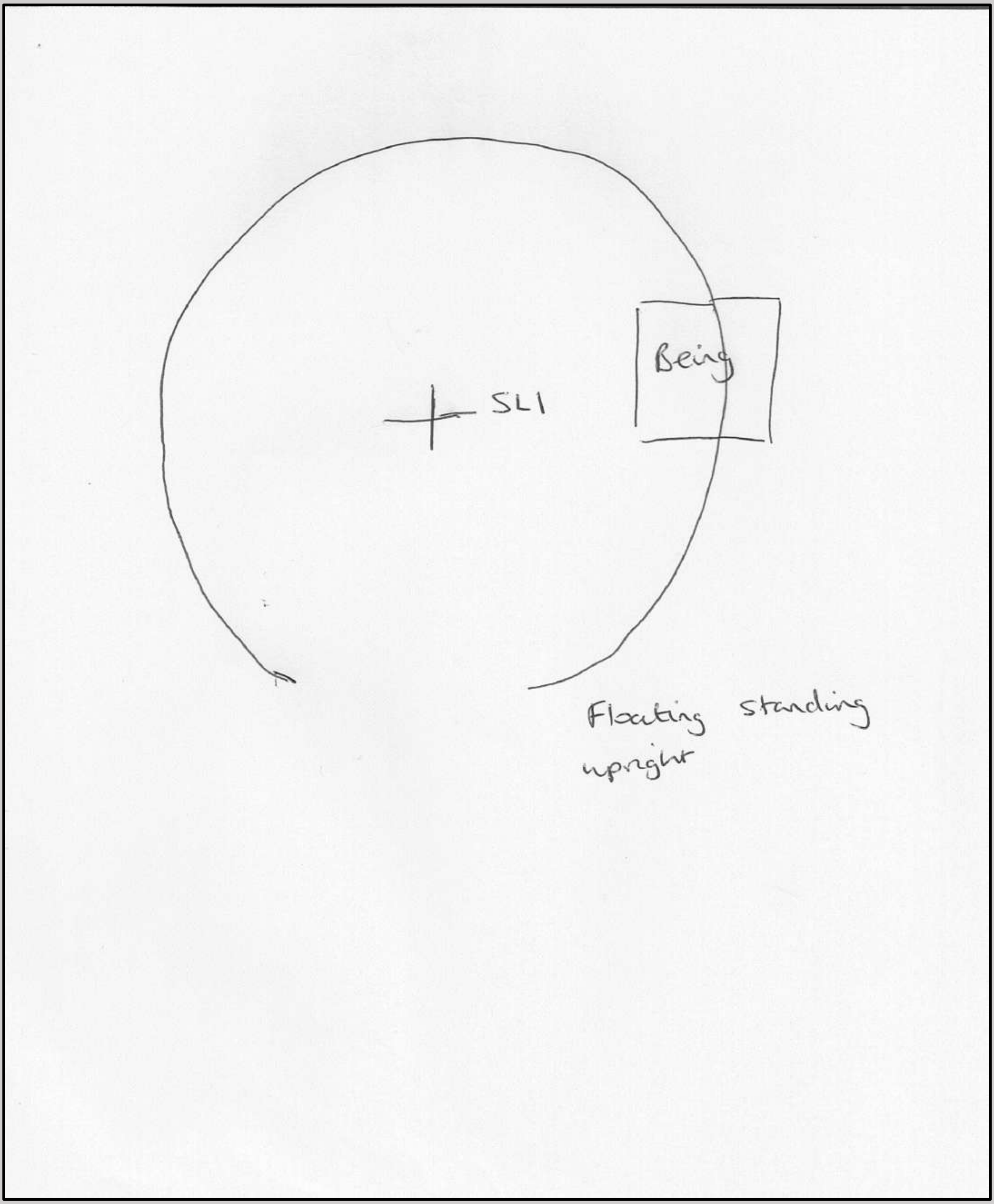

• B10

Subject 5

Sequence: part/space

2 OBE + 4 NDE

- OBE Dissociative
- A1
- C5
- OBE Associative

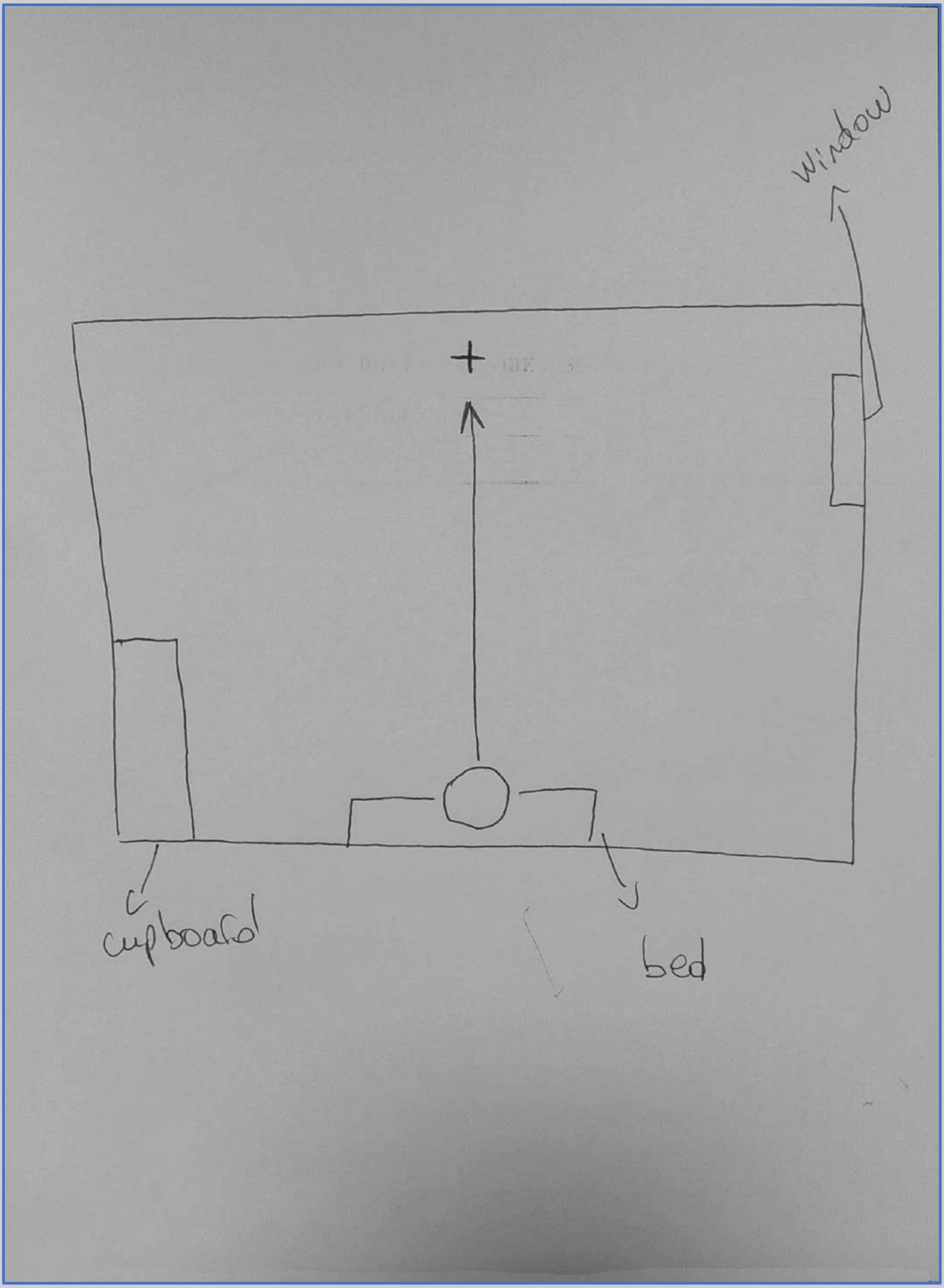

- OBE Dissociative

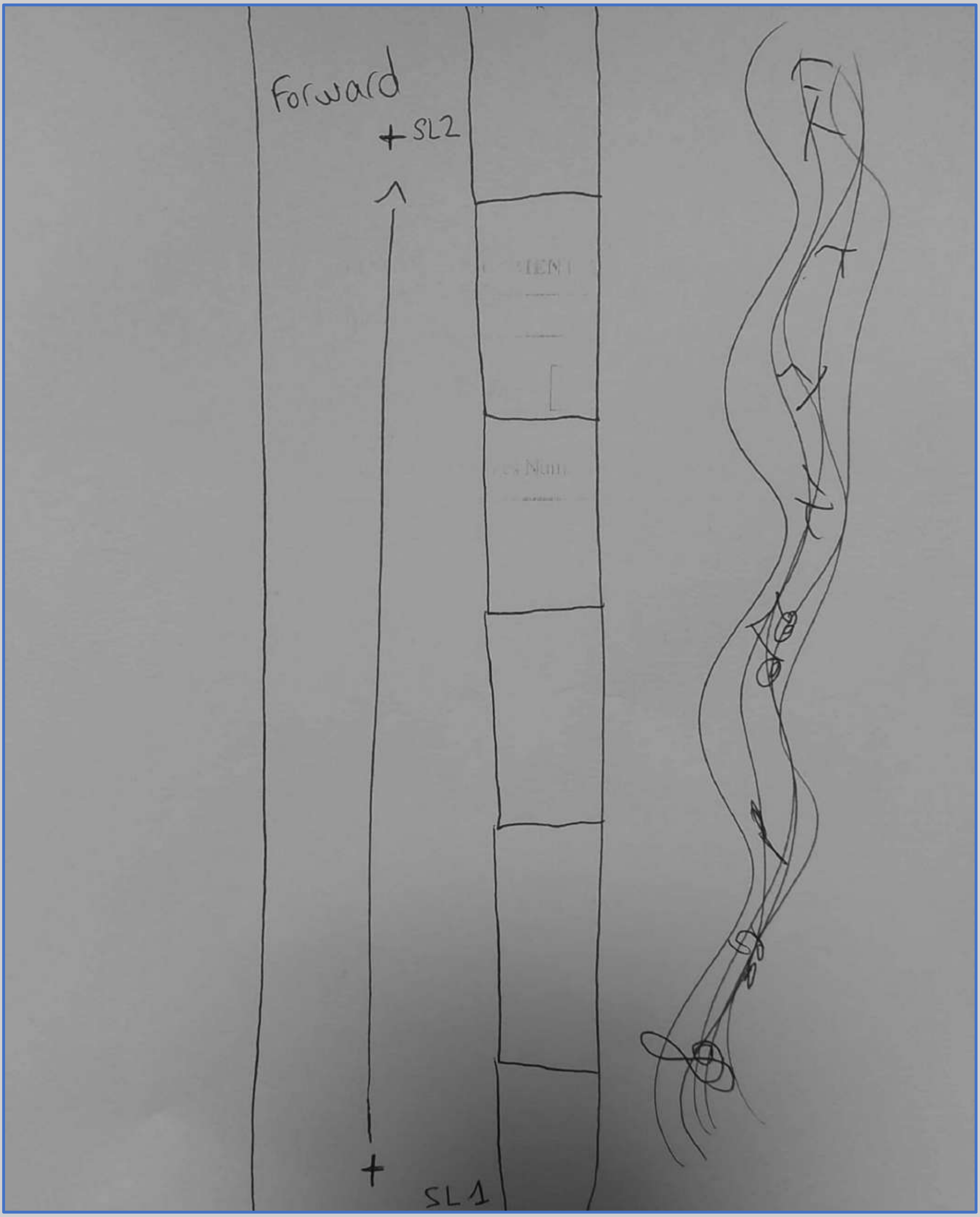

- A1

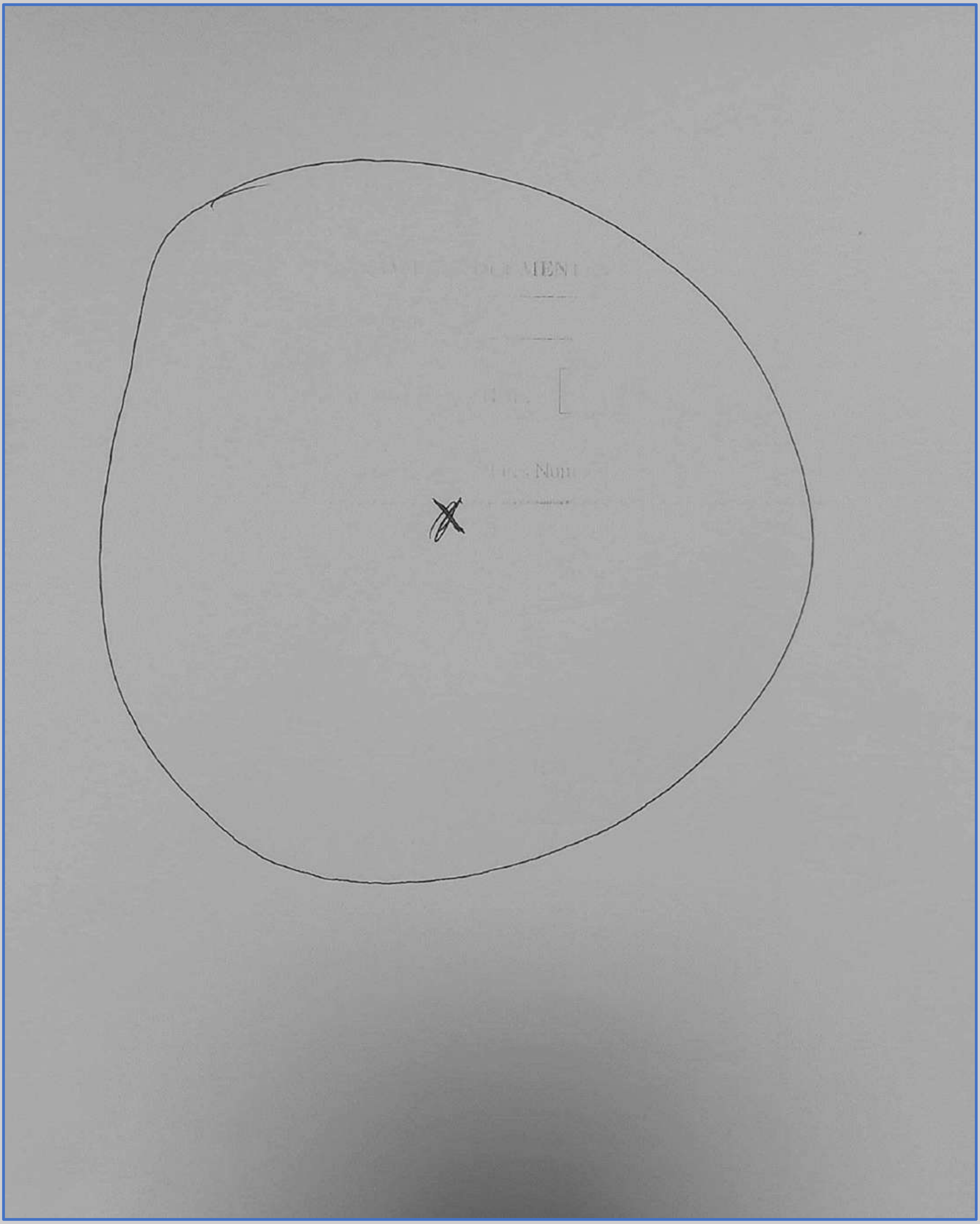

- C5

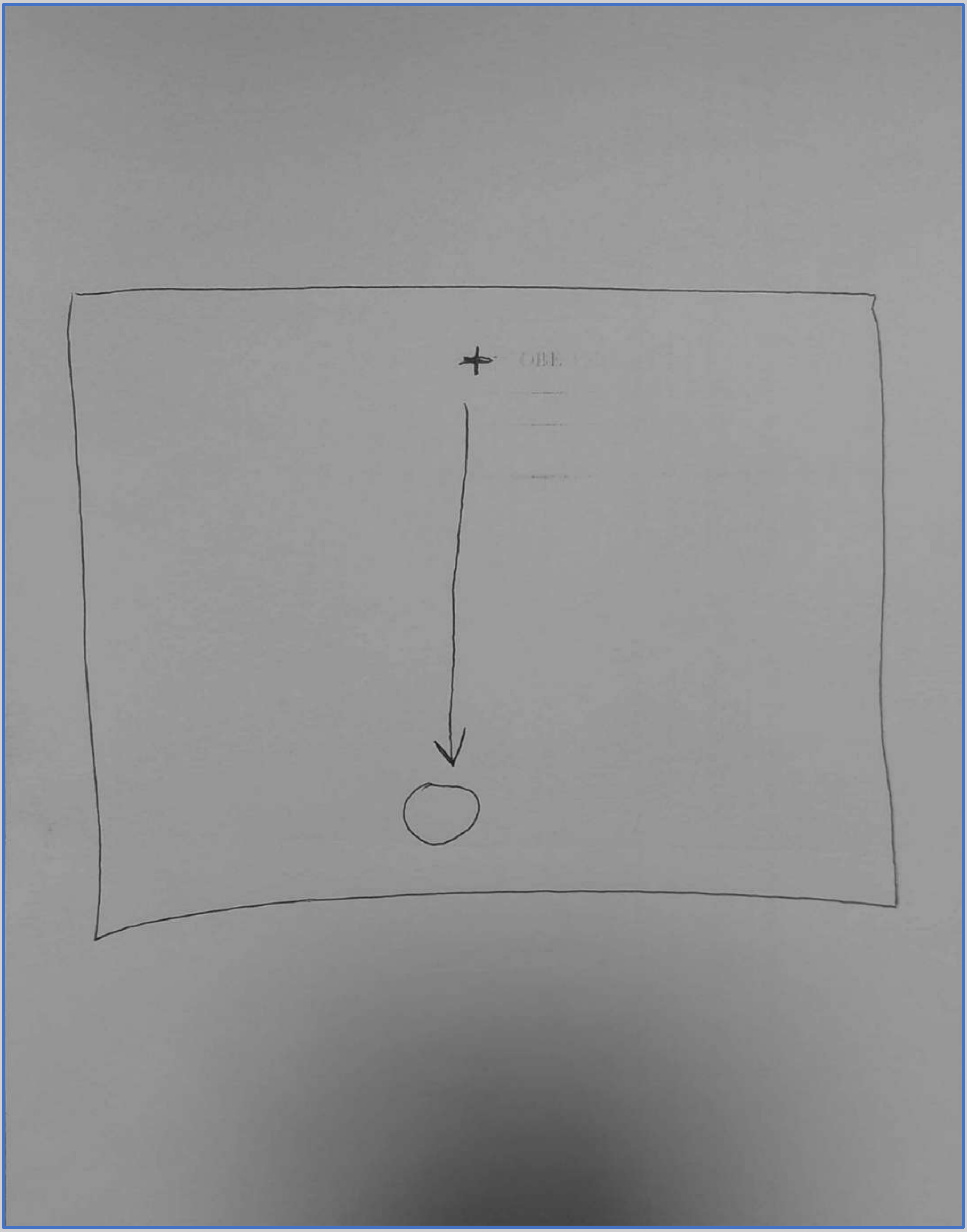

• OBE Associative

Subject 6

Sequence: part/space

2 OBE + 6 NDE

- OBE Dissociative
- A1
- C5
- B12
- C1
- C2
- C5
- OBE Associative

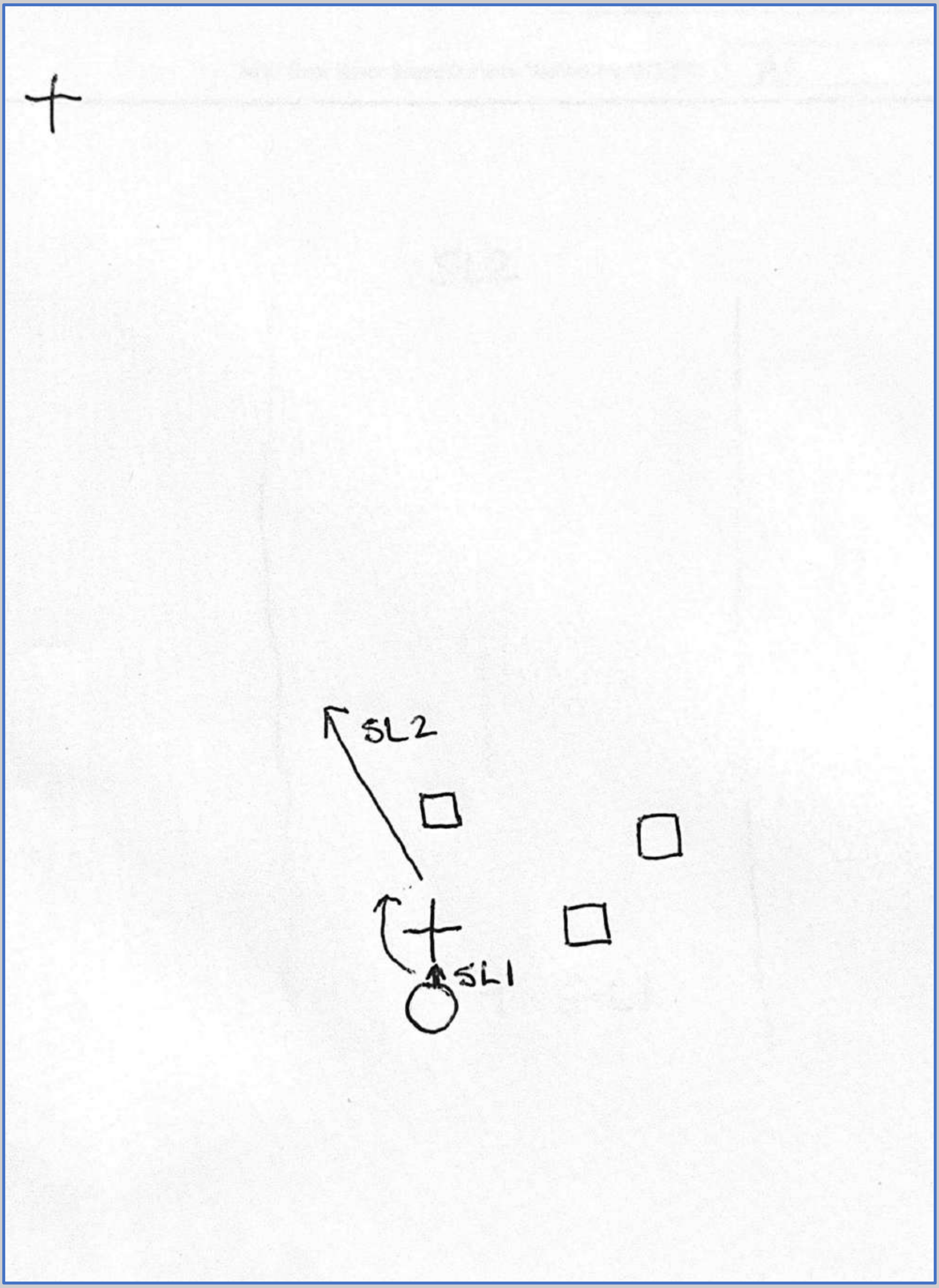

- OBE Dissociative

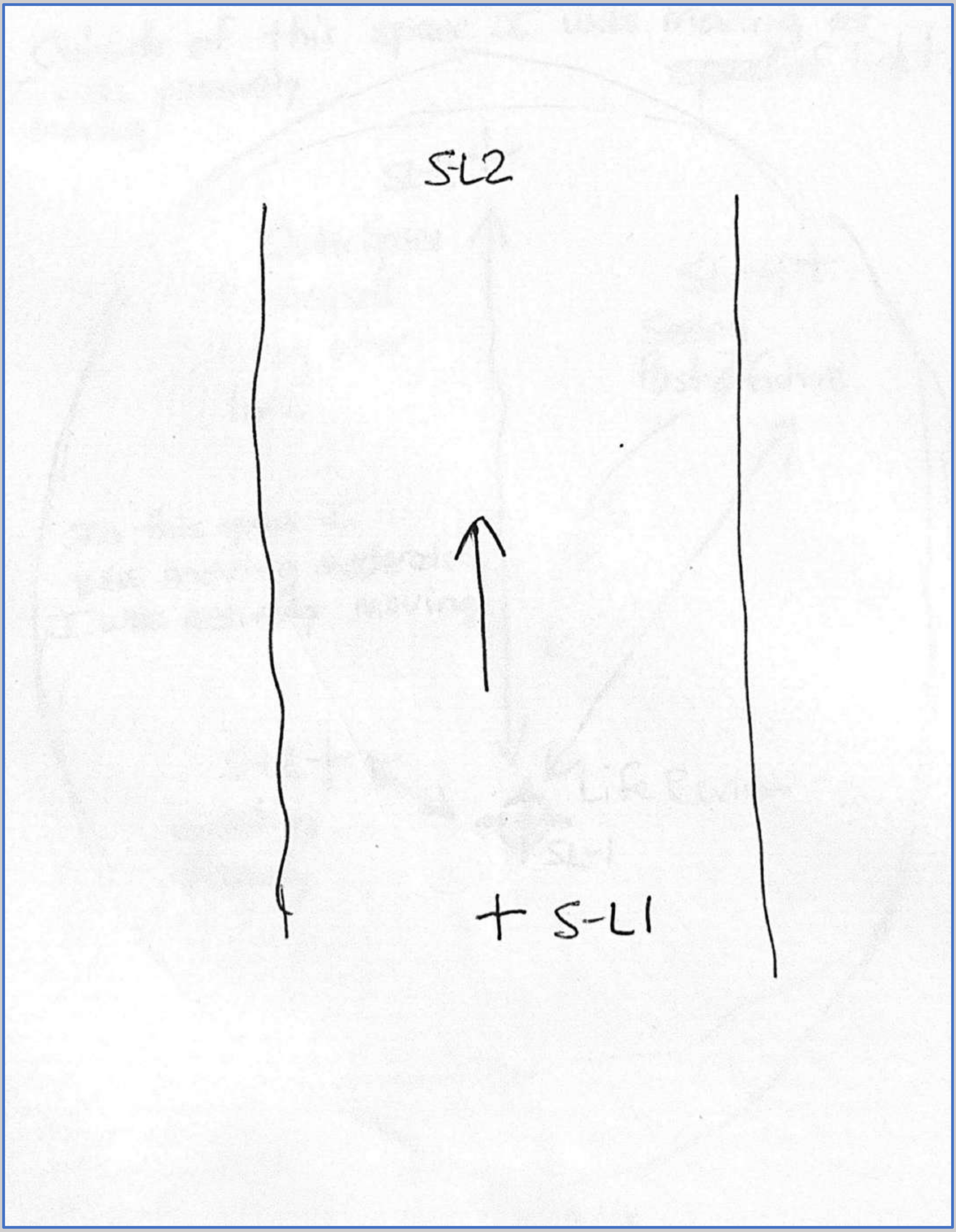

• A1

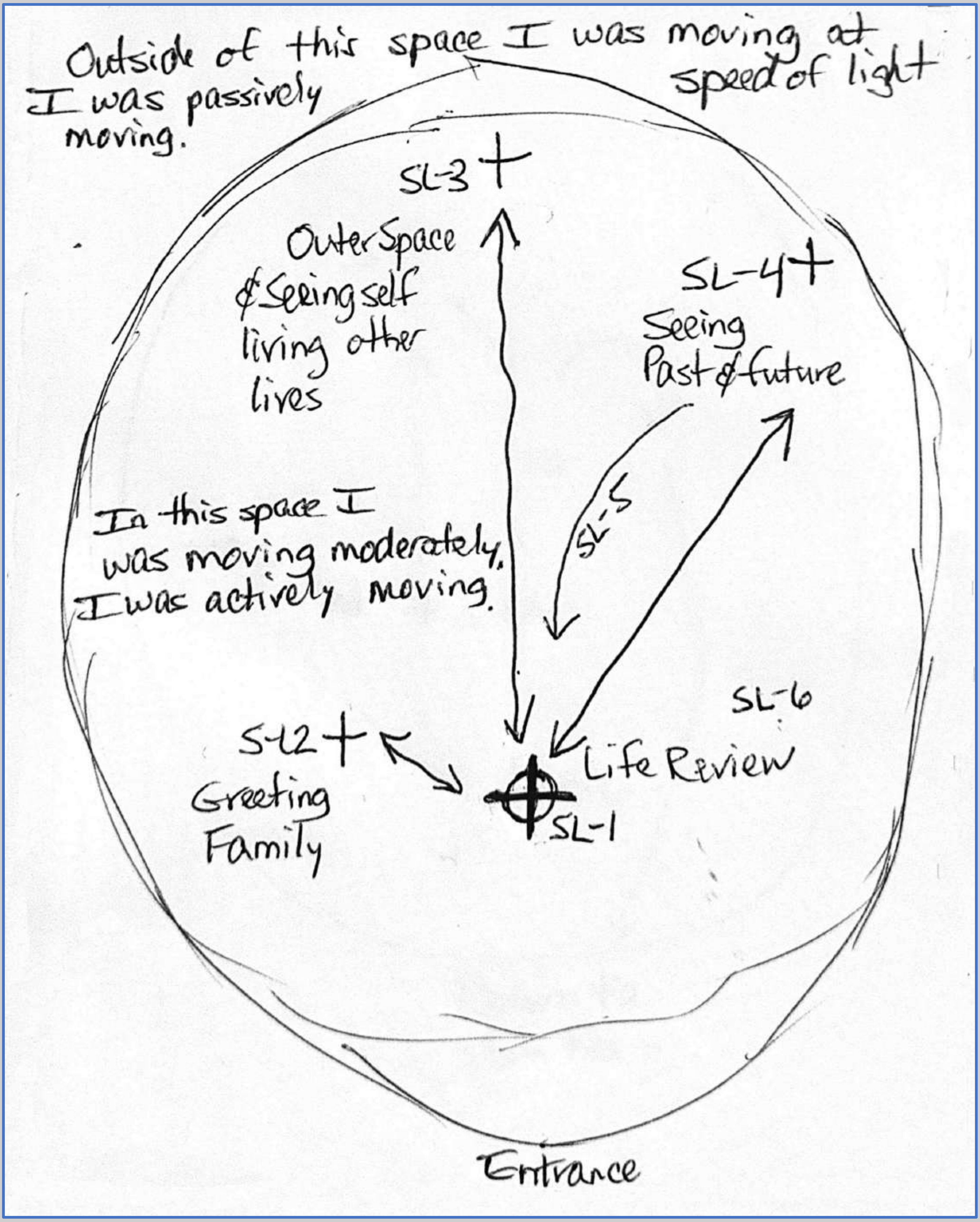

Outside of this space I was moving at speed of light
I was passively moving.
SL-3
Outer Space & Seeing self living other lives
SL-4
Seeing Past & future
SL-5
In this space I was moving moderately. I was actively moving.
SL-6
SL-2
Greeting Family
Life Review
SL-1
Entrance

• C5

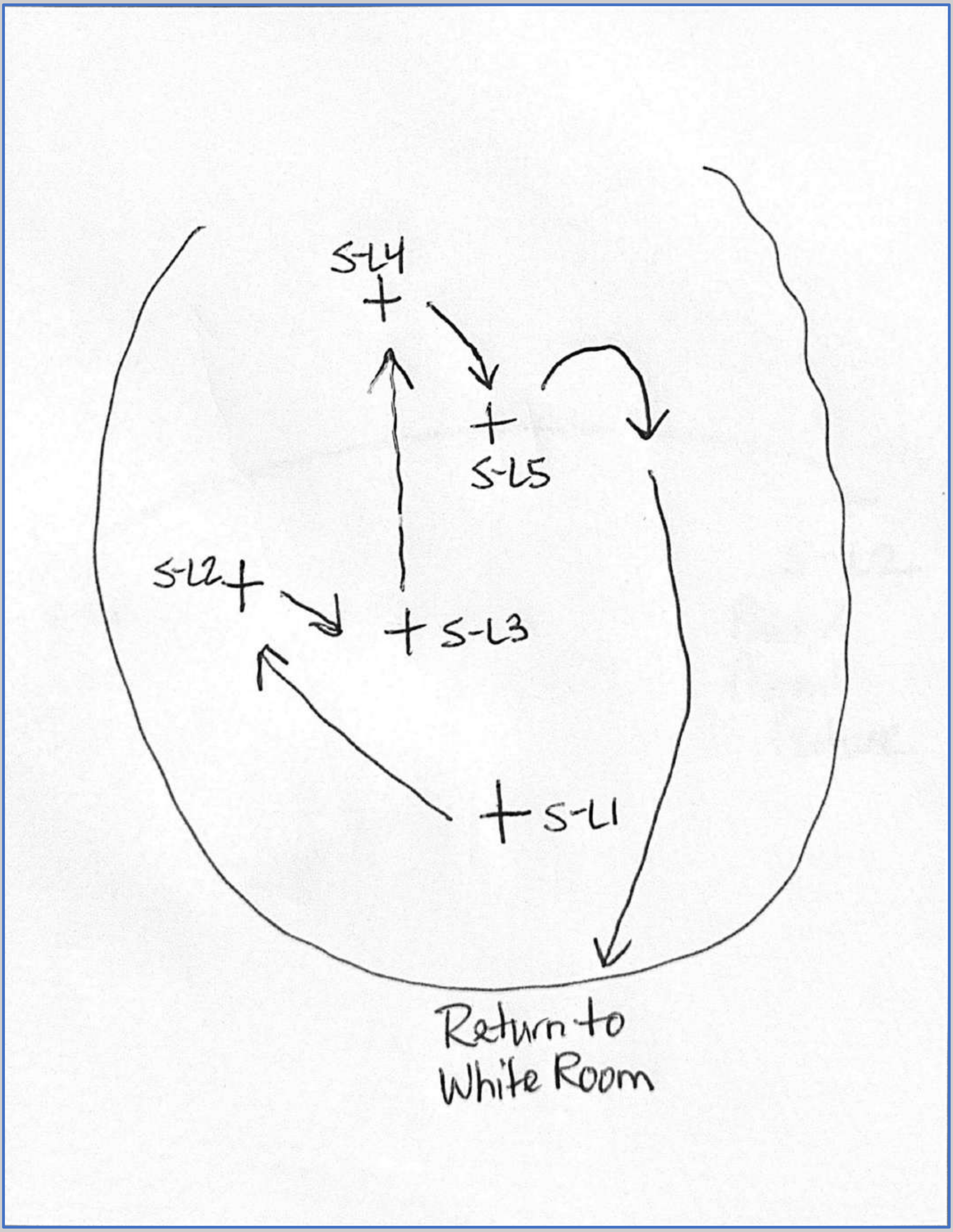

• B12

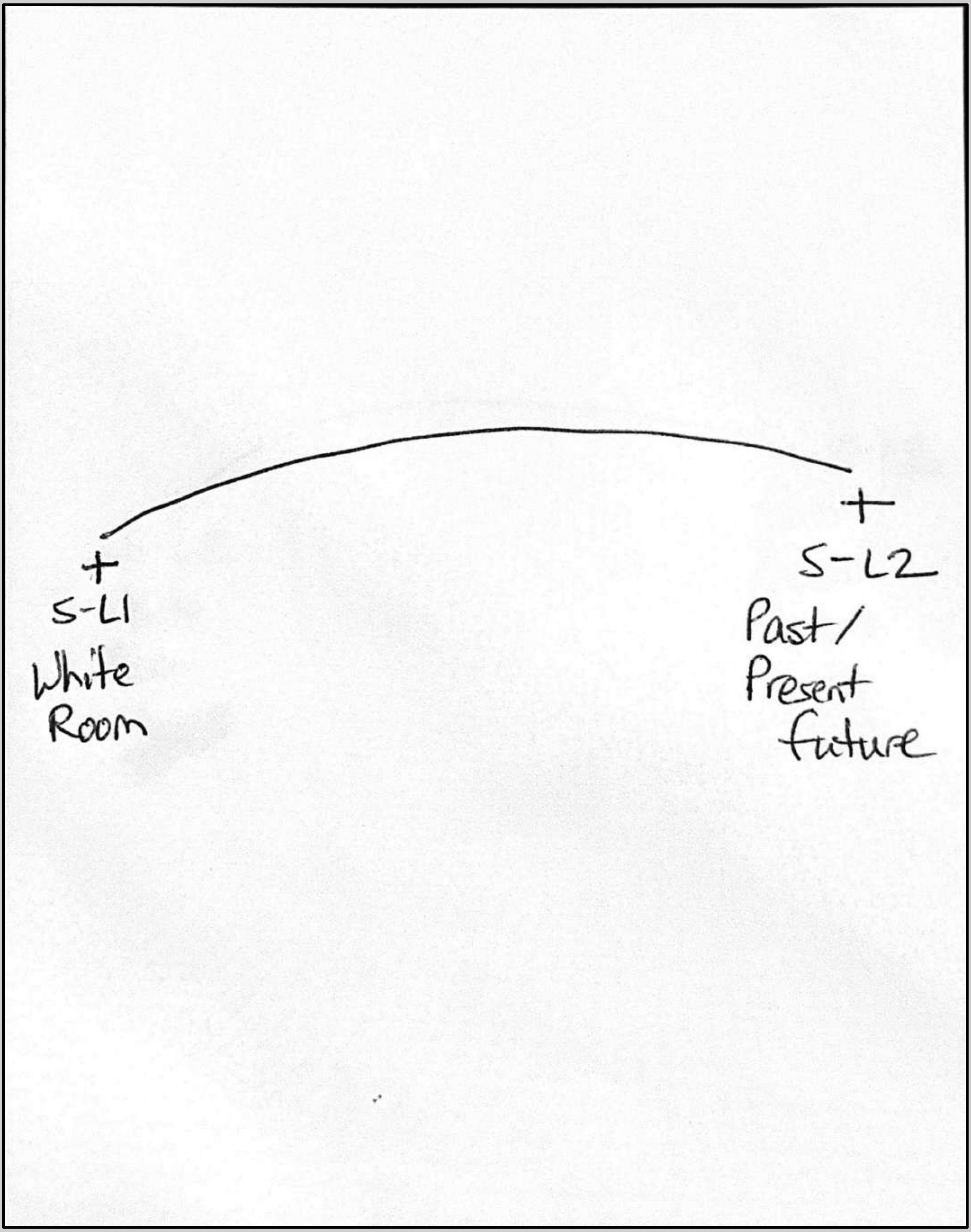

• C1

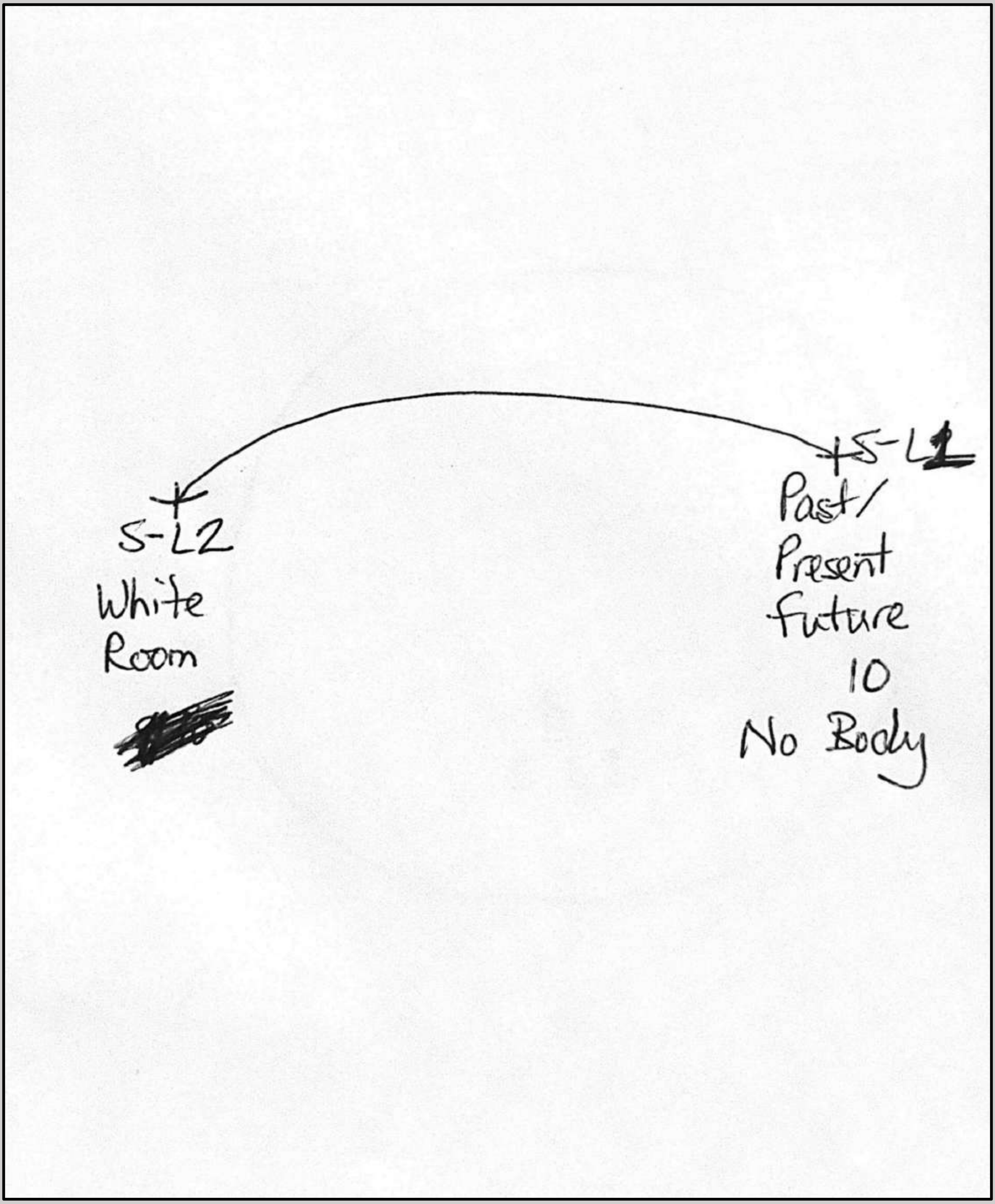

• C2

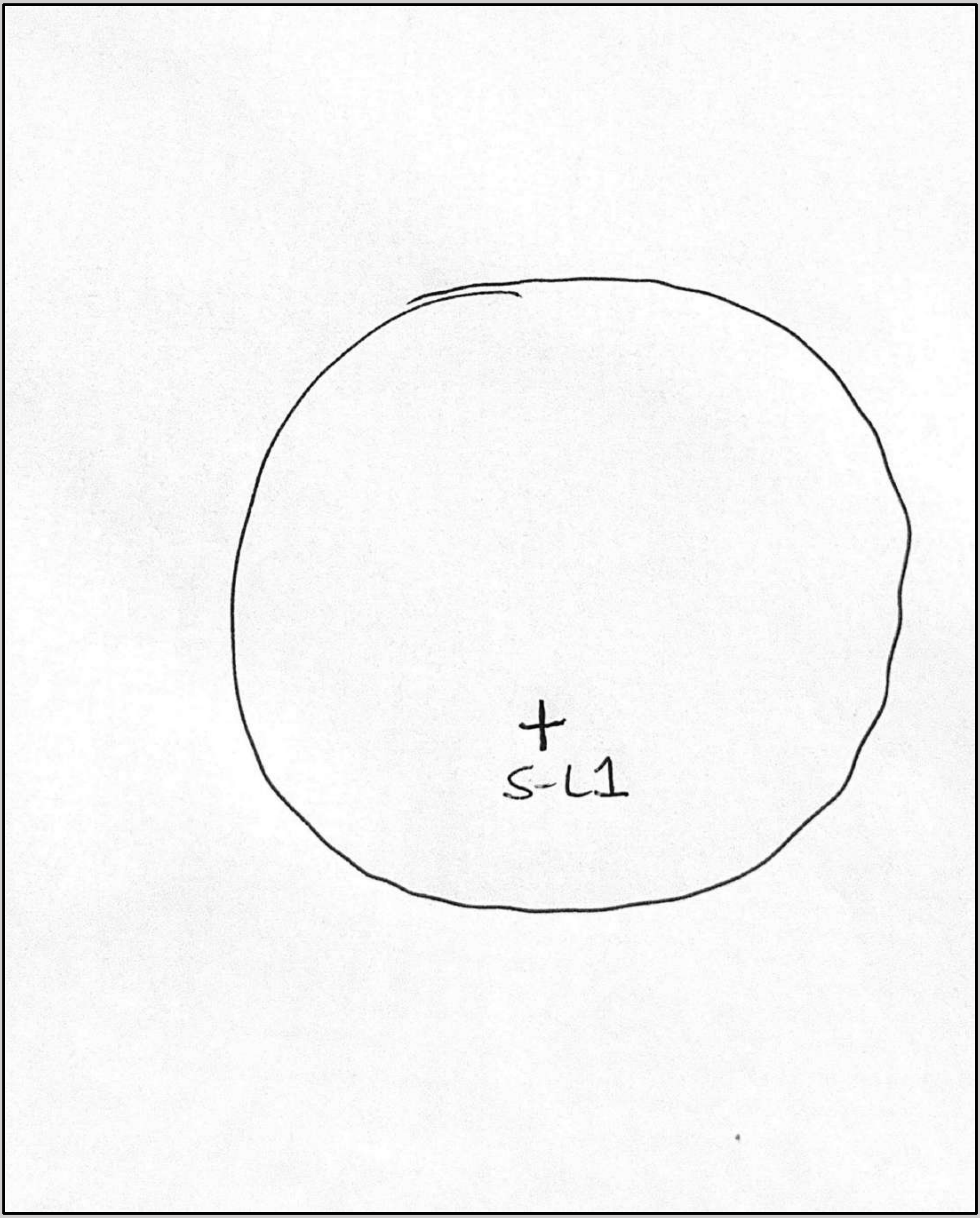

- C5

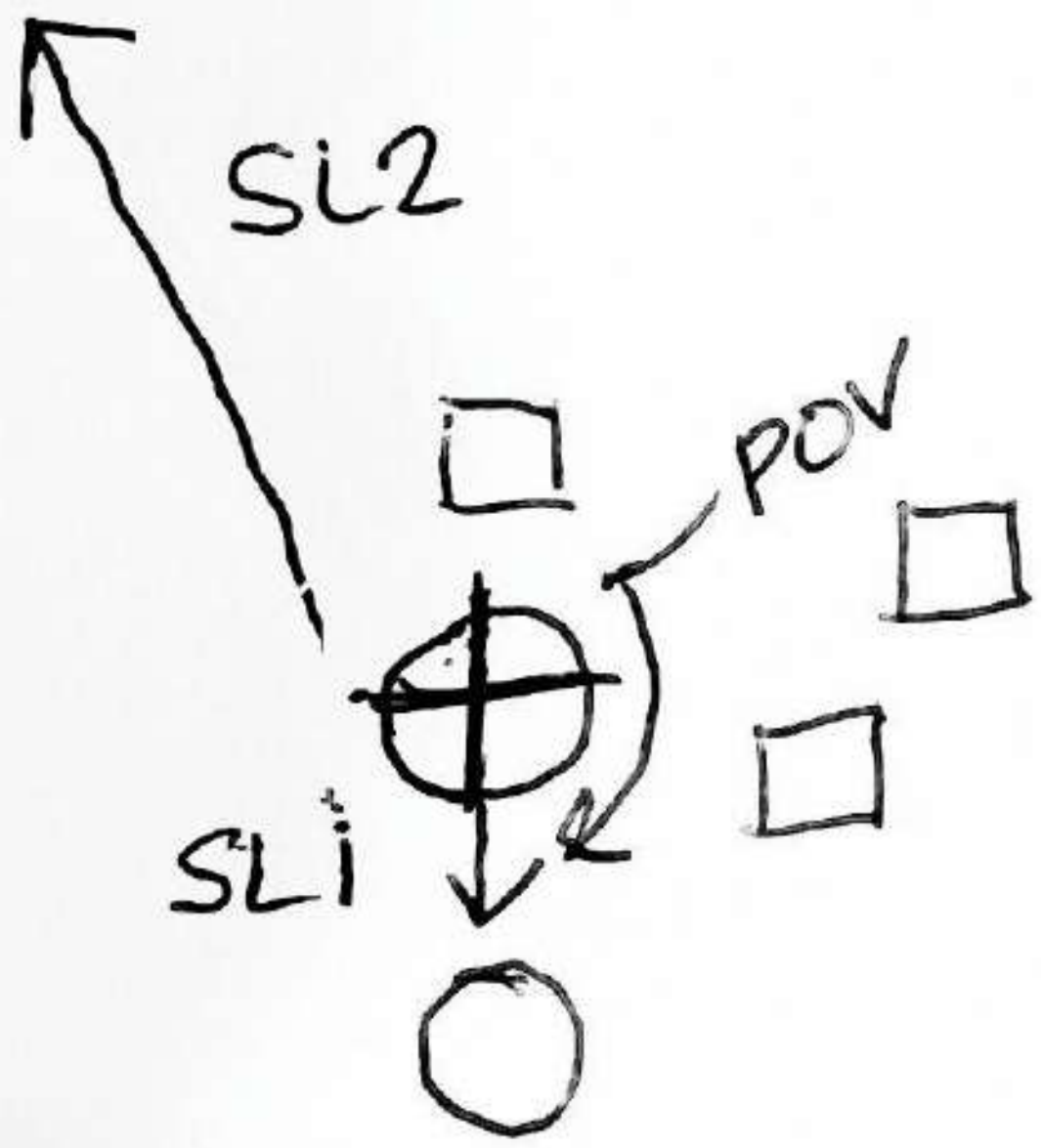

• OBE Associative

Subject 7

Sequence: part/space

1 OBE + 2 NDE

- OBE Dissociative
- A1
- C2

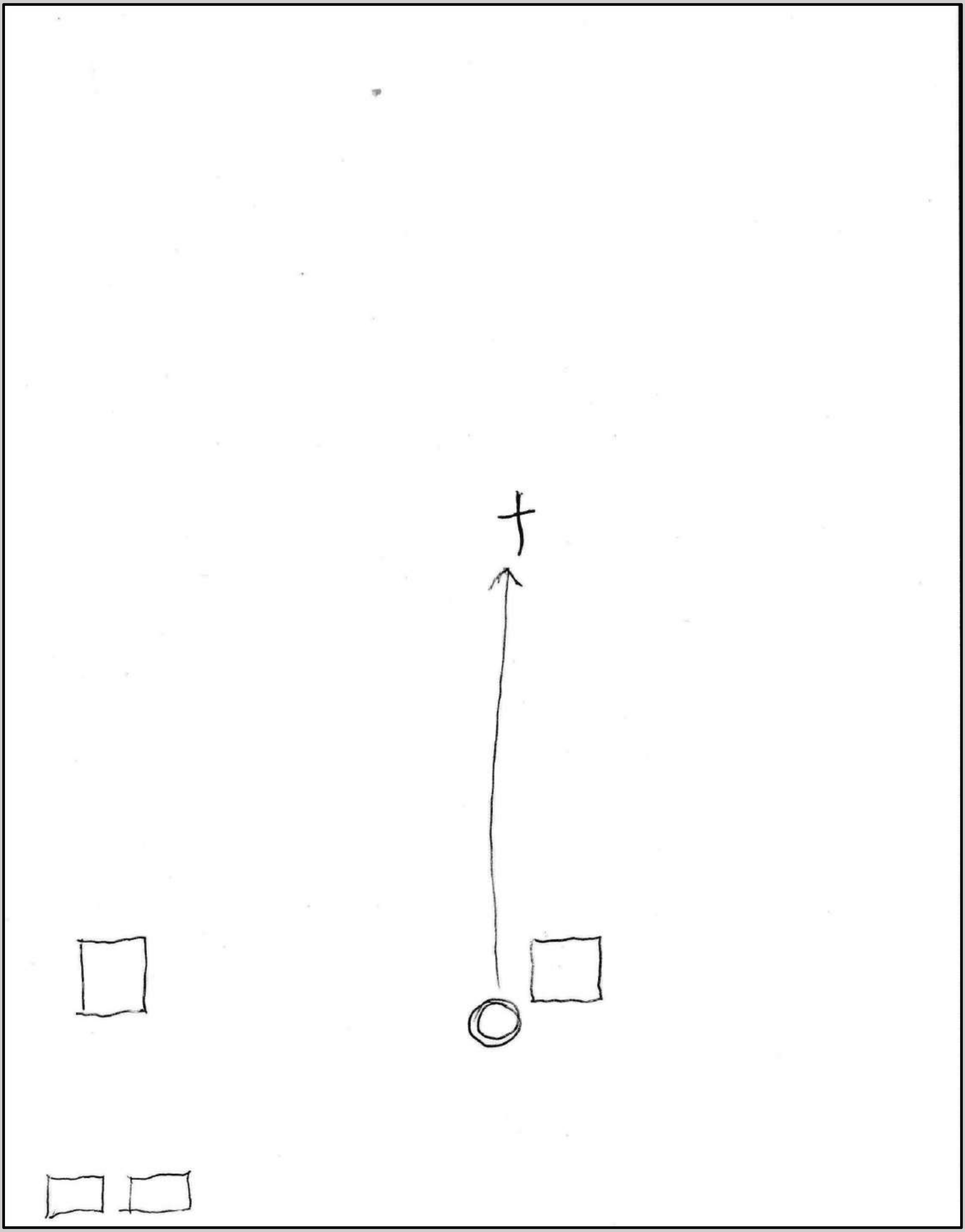

- OBE Dissociative

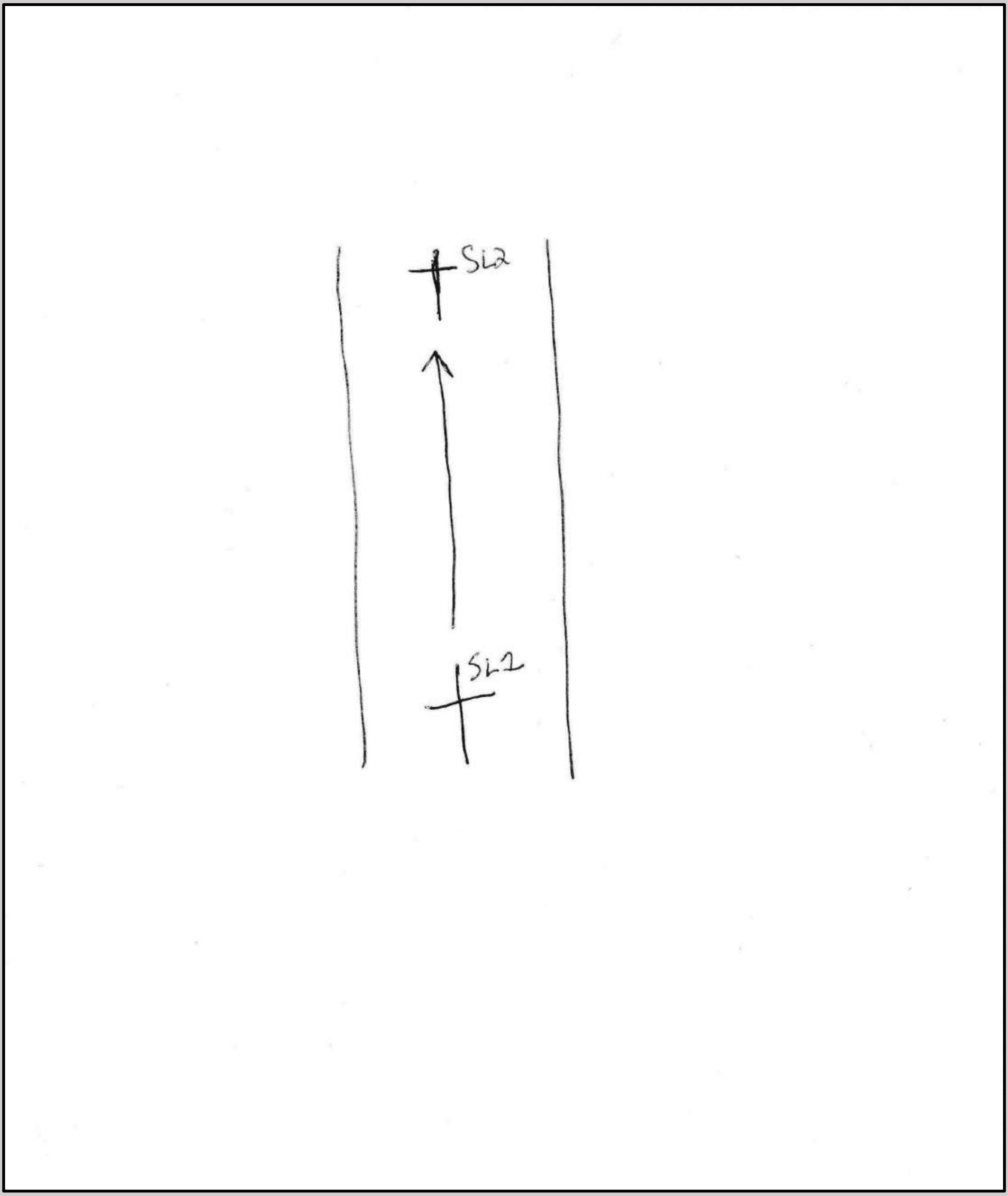

- A1

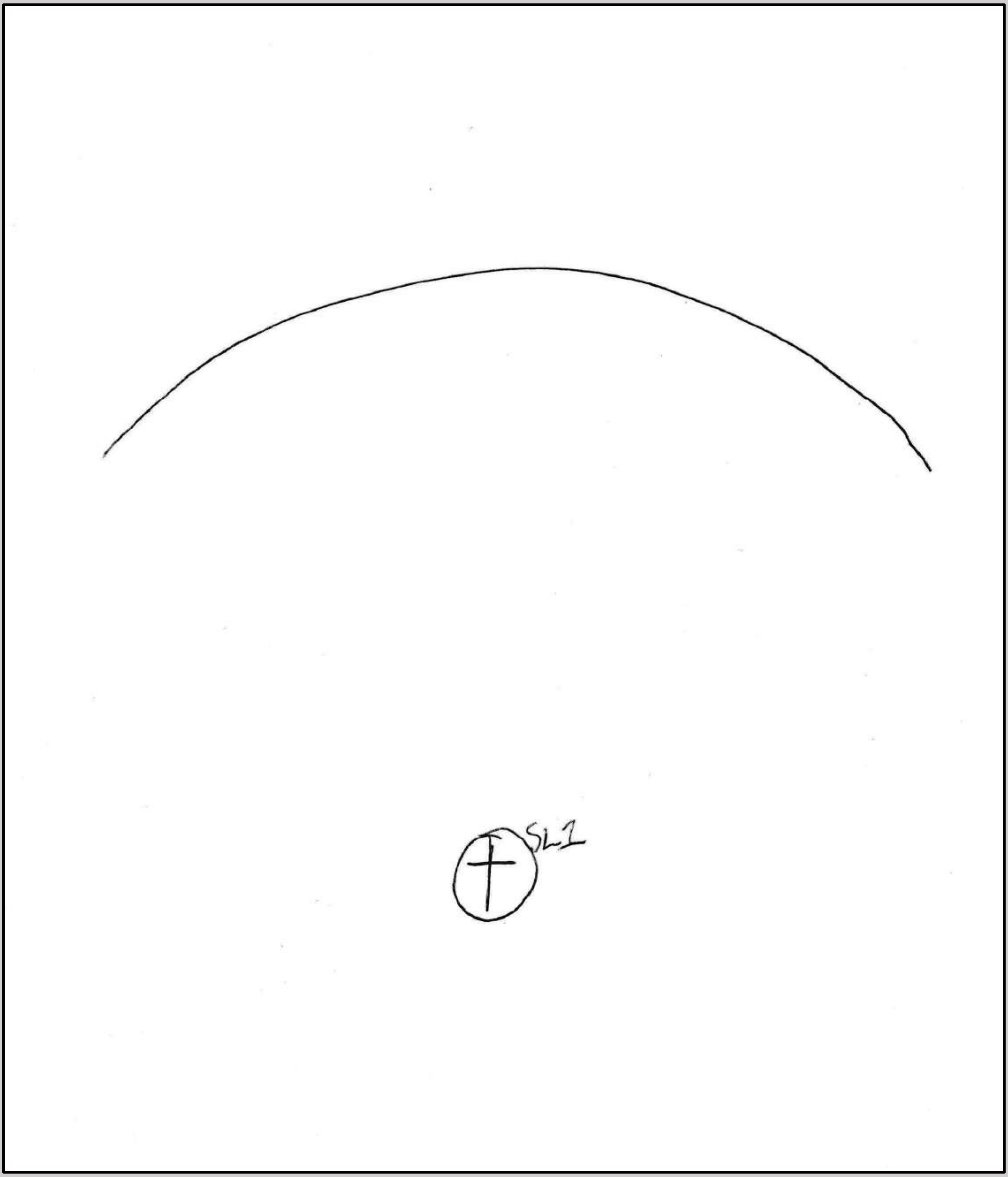

- C2

Subject 8

Sequence: part/space

1 OBE + 1 NDE

- OBE Dissociative
- A2

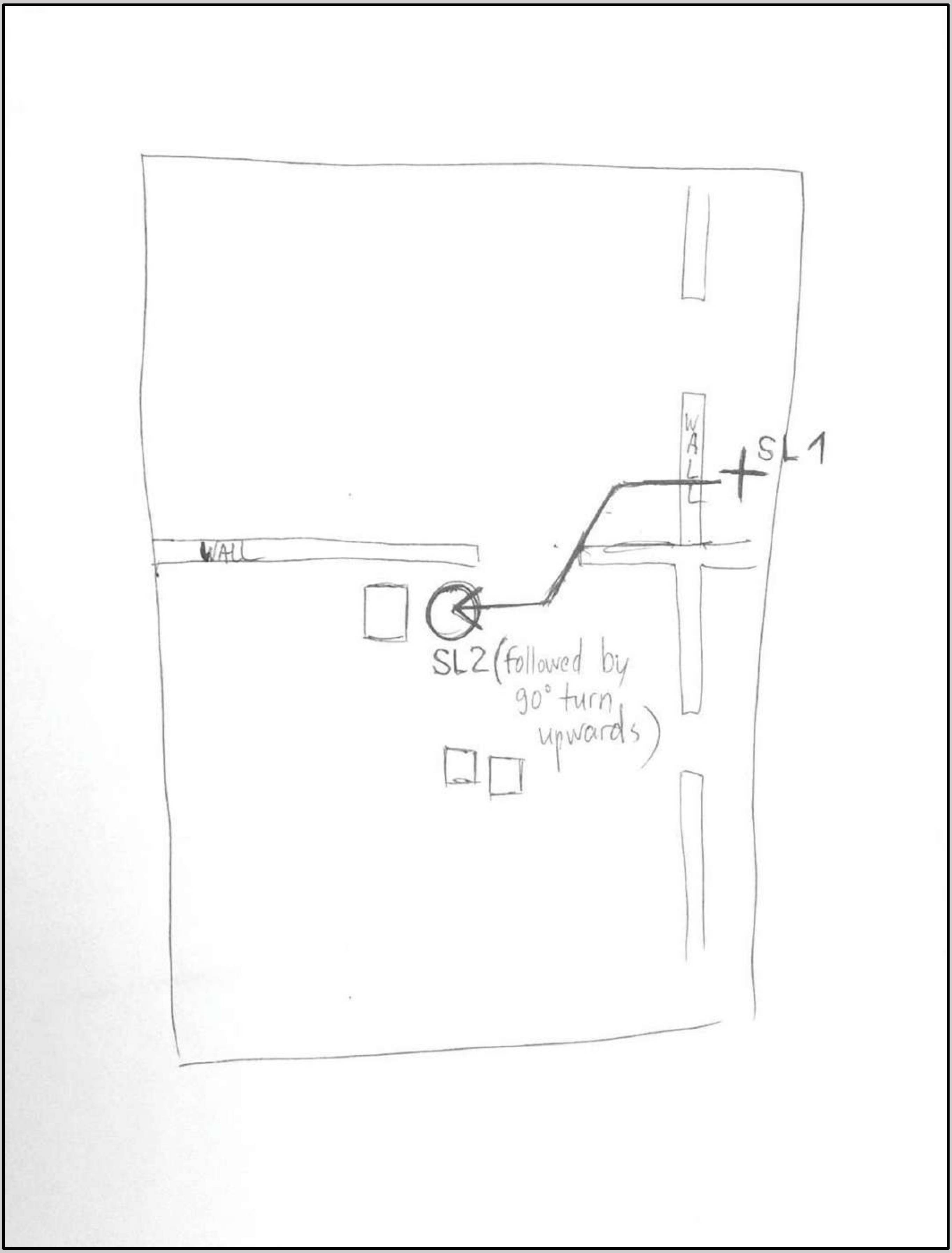

- OBE Dissociative

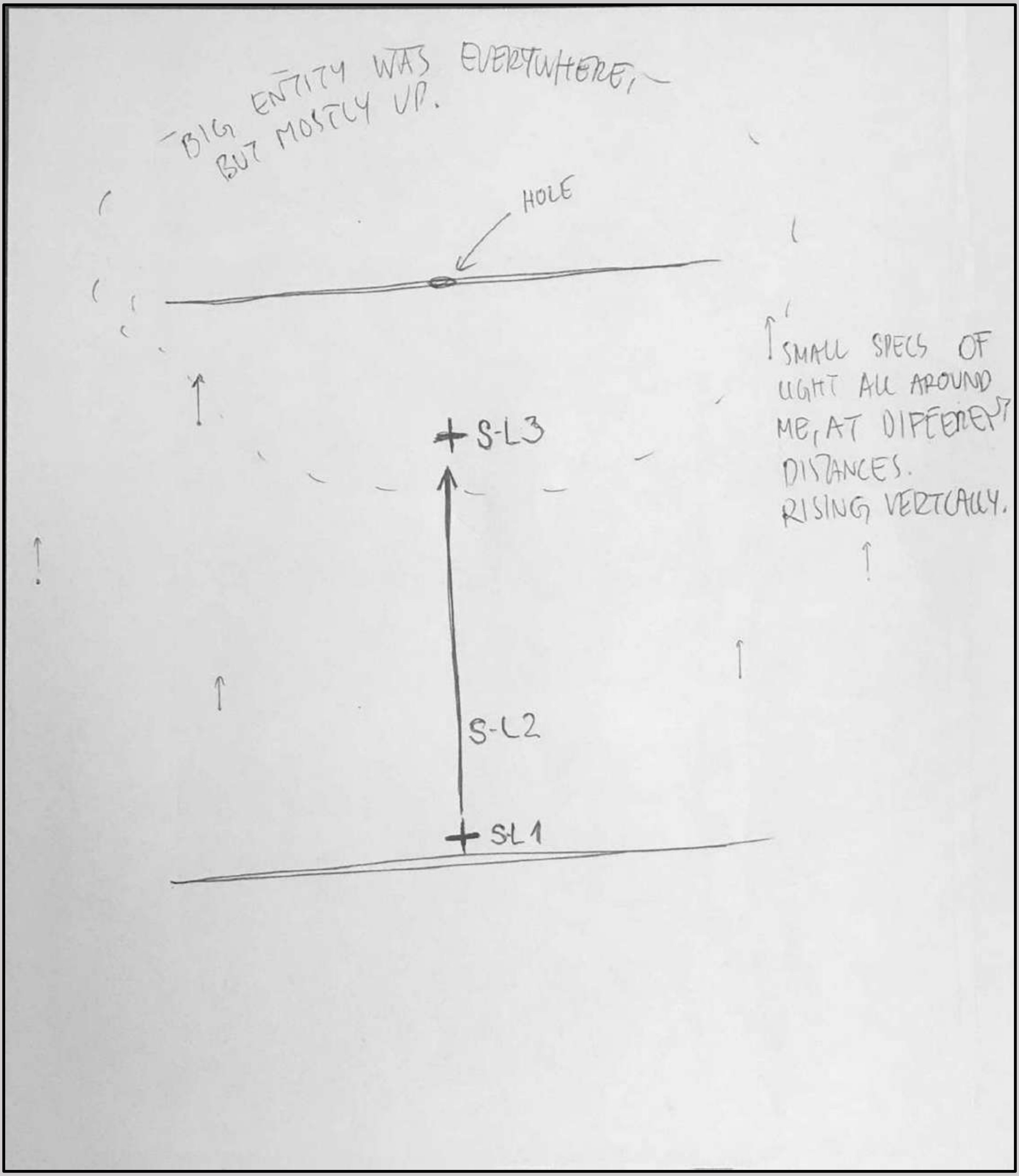
BIG ENTITY WAS EVERYWHERE, BUT MOSTLY UP.
HOLE
SMALL SPECS OF LIGHT ALL AROUND ME, AT DIFFERENT DISTANCES. RISING VERTICALLY.
S-L3
S-L2
S-L1

- A2

Subject 9

Sequence: part/space

1 OBE + 2 NDE

- A5
- A5
- OBE Associative

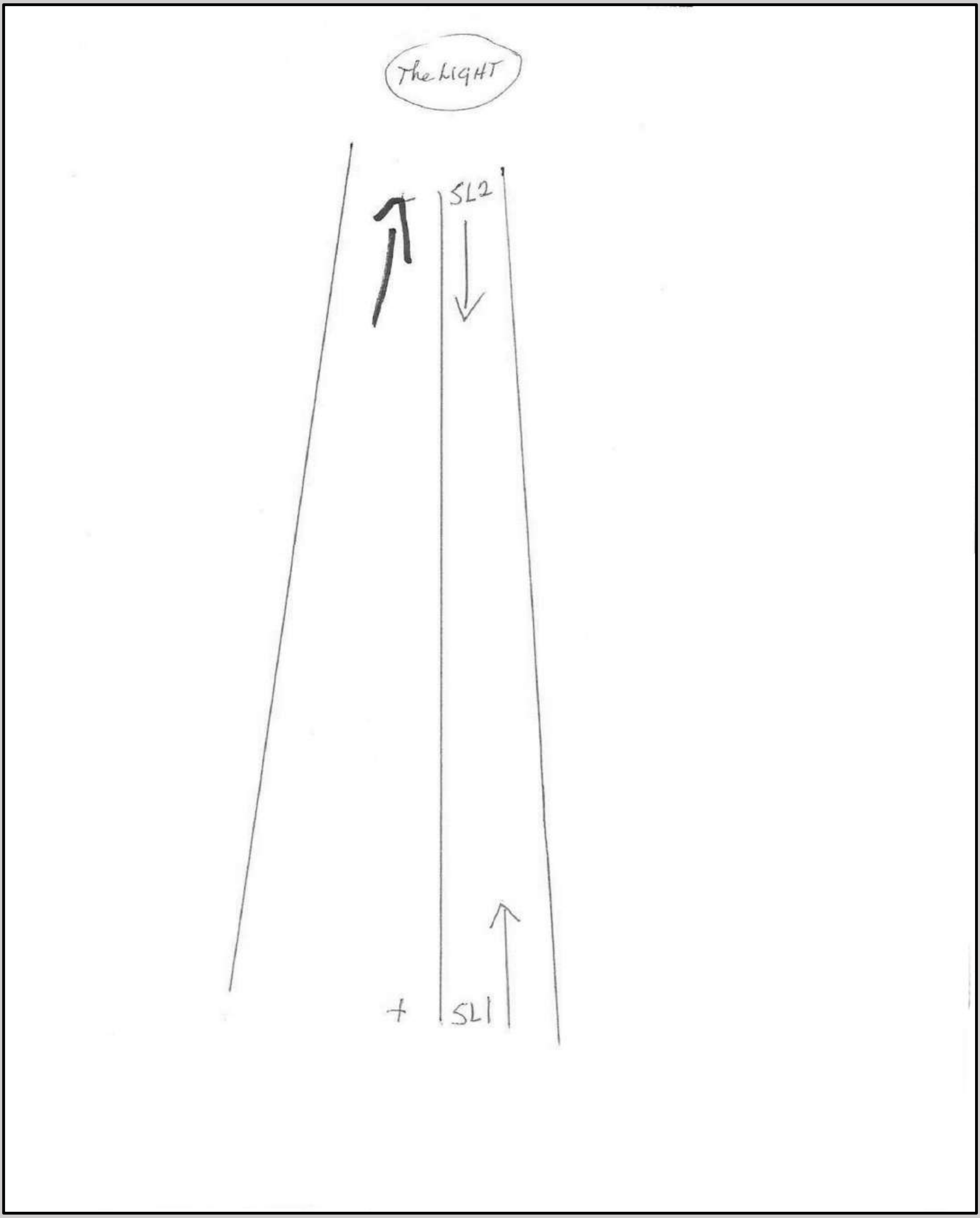

• A5

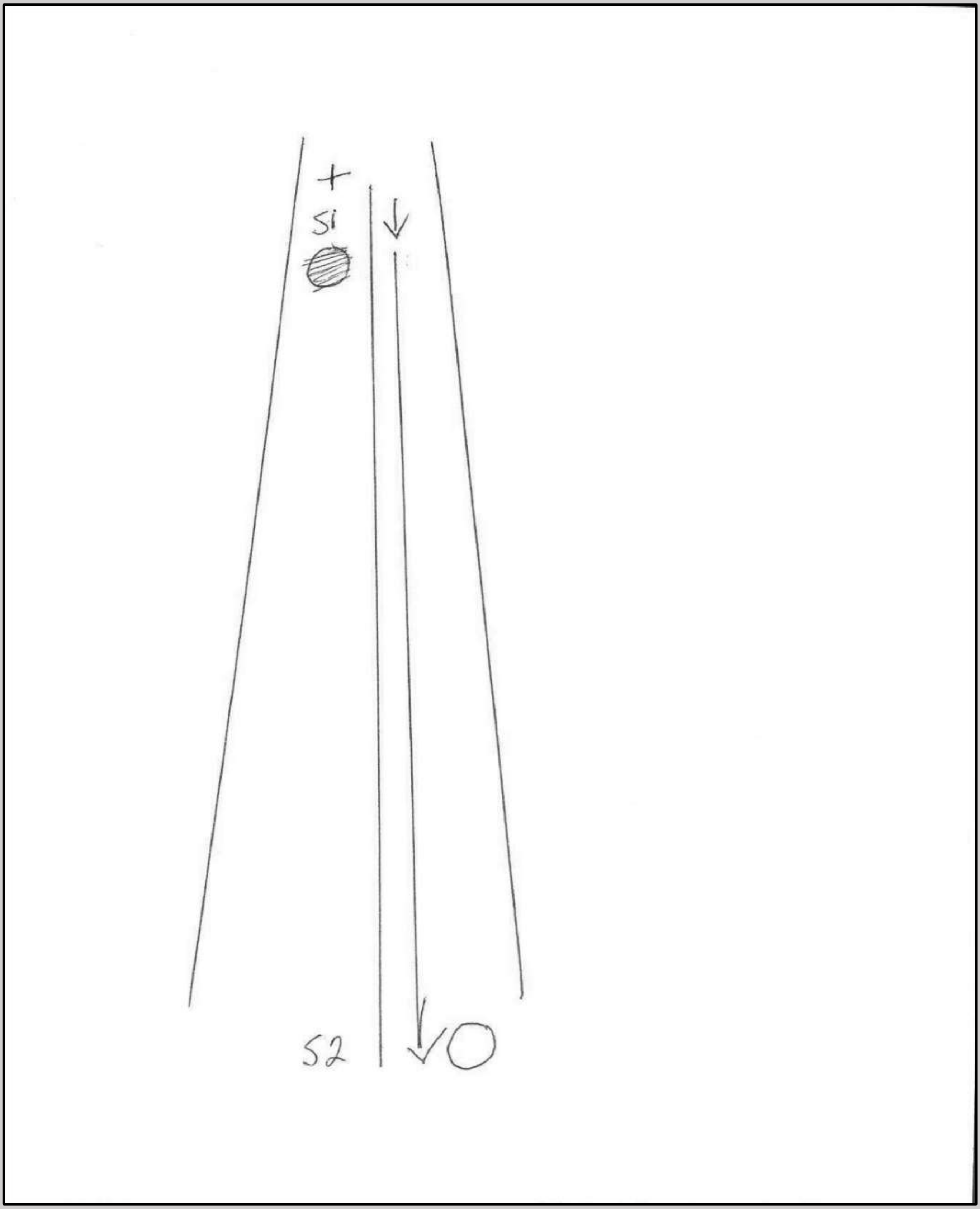

- A5

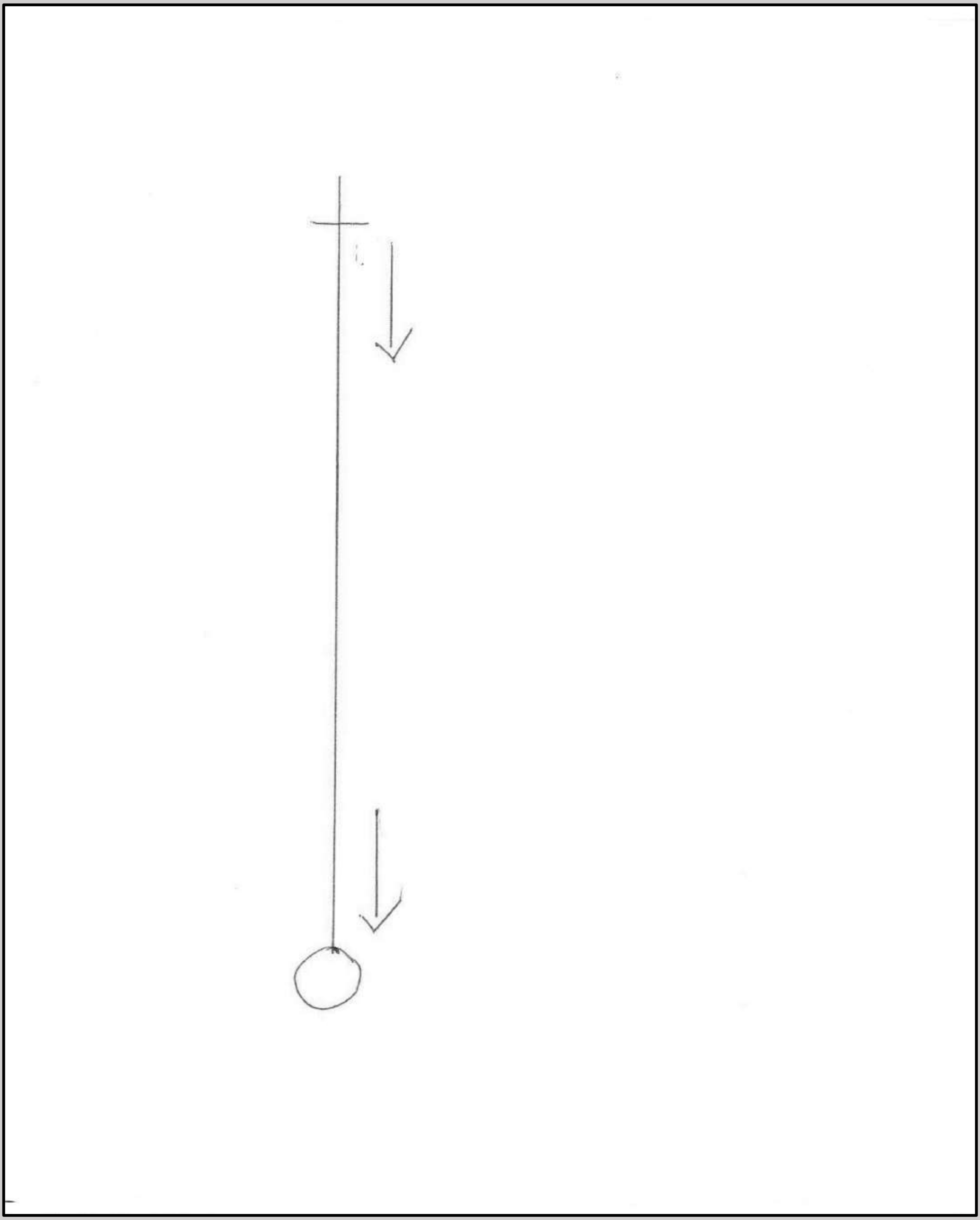

- OBE Associative

Subject 10

Sequence: part/space

1 OBE + 5 NDE

- A5
- B6
- C5
- B7
- A9
- OBE Associative

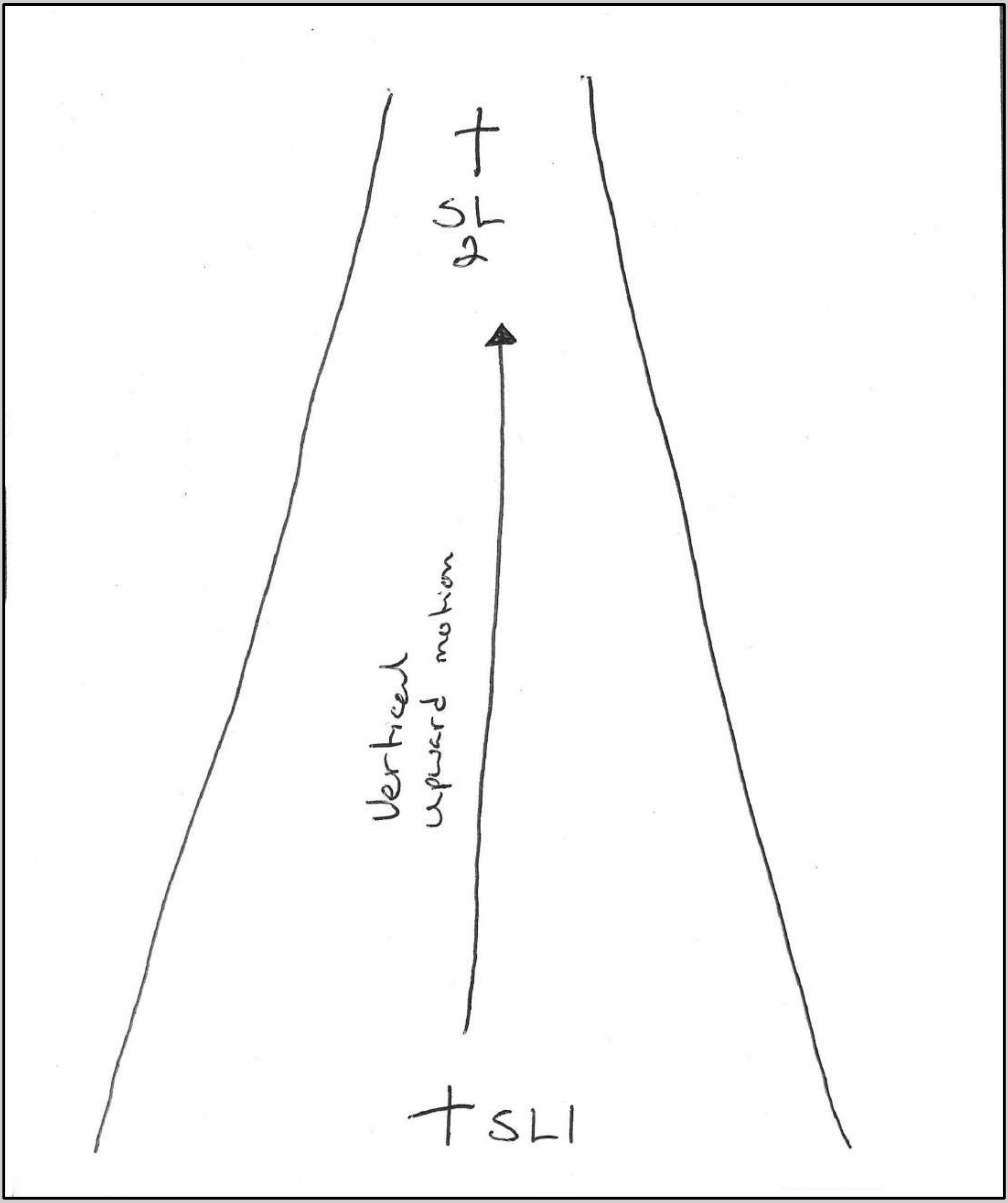

- A5

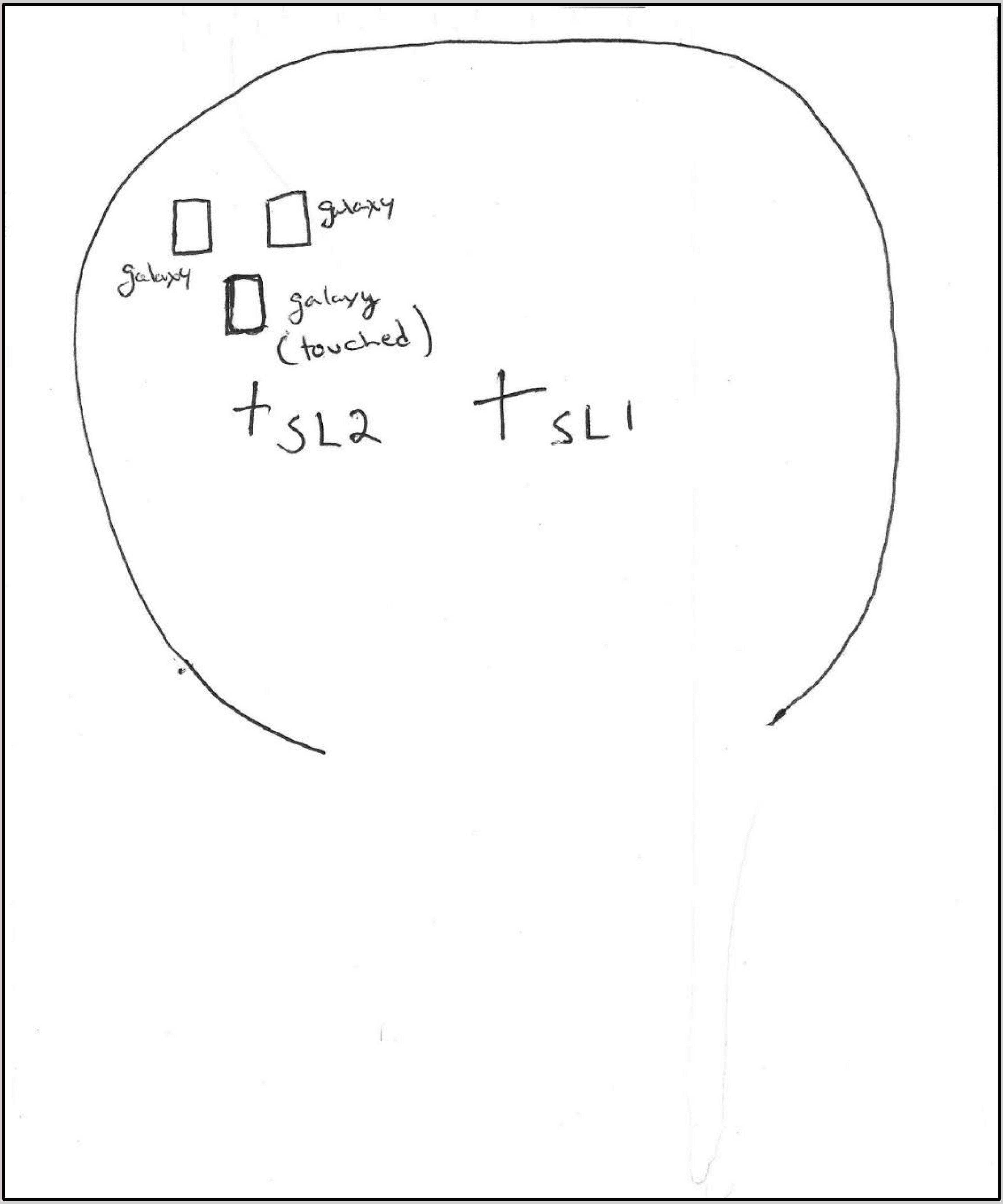

- B6

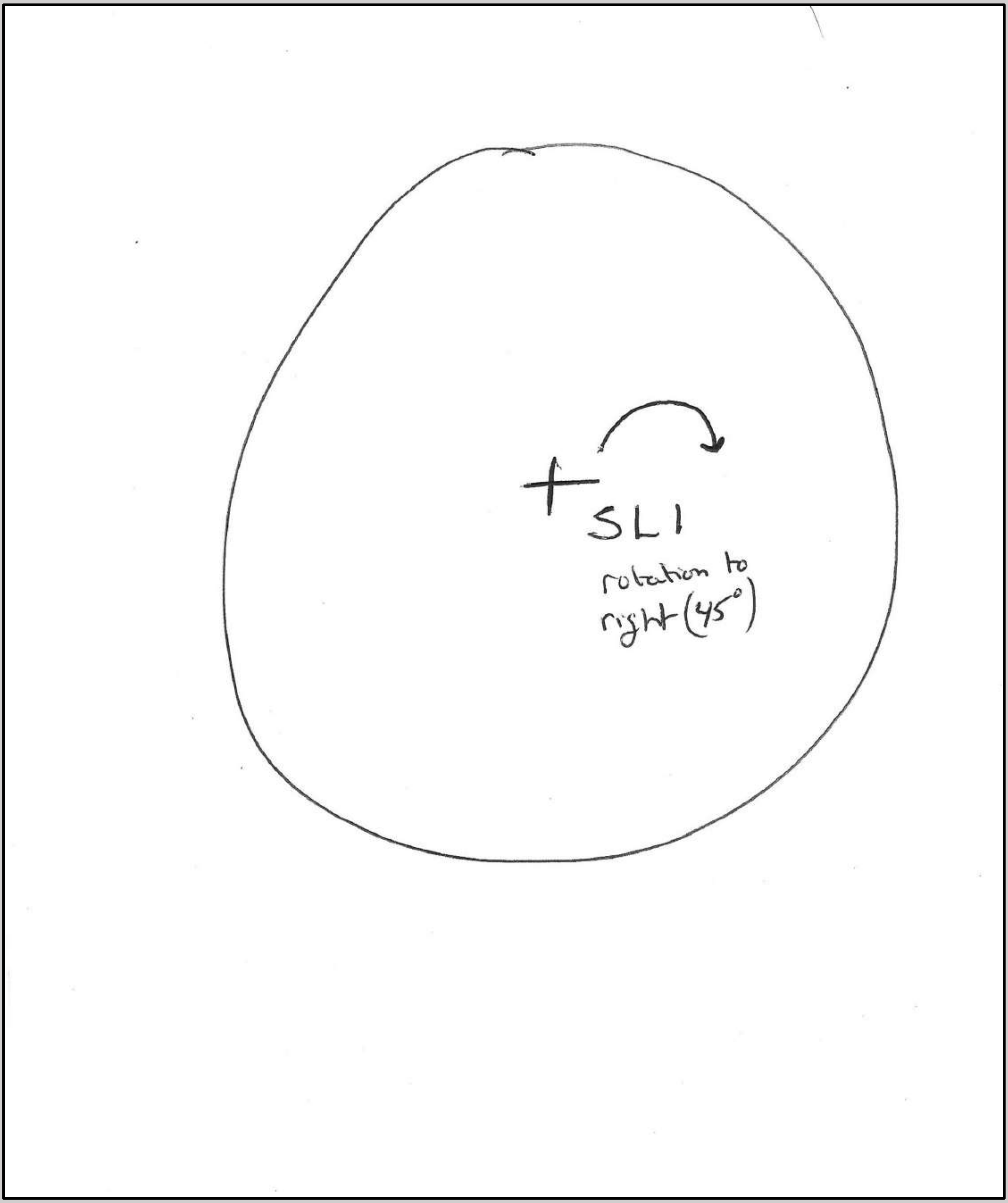

- C5

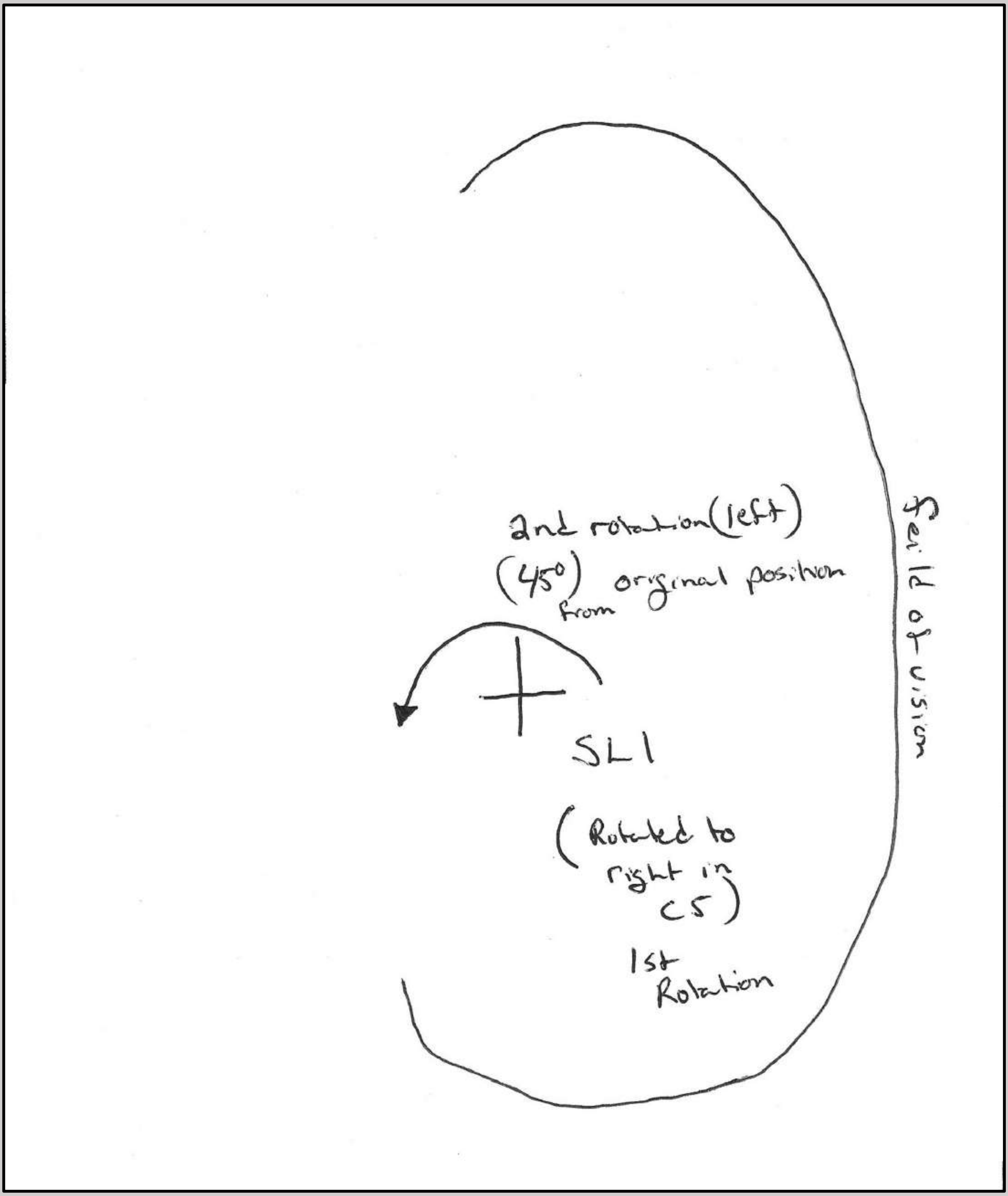
2nd rotation(left)
(45°) from original position
SL1
(Rotated to right in C5)
1st Rotation
feild of vision

- B7

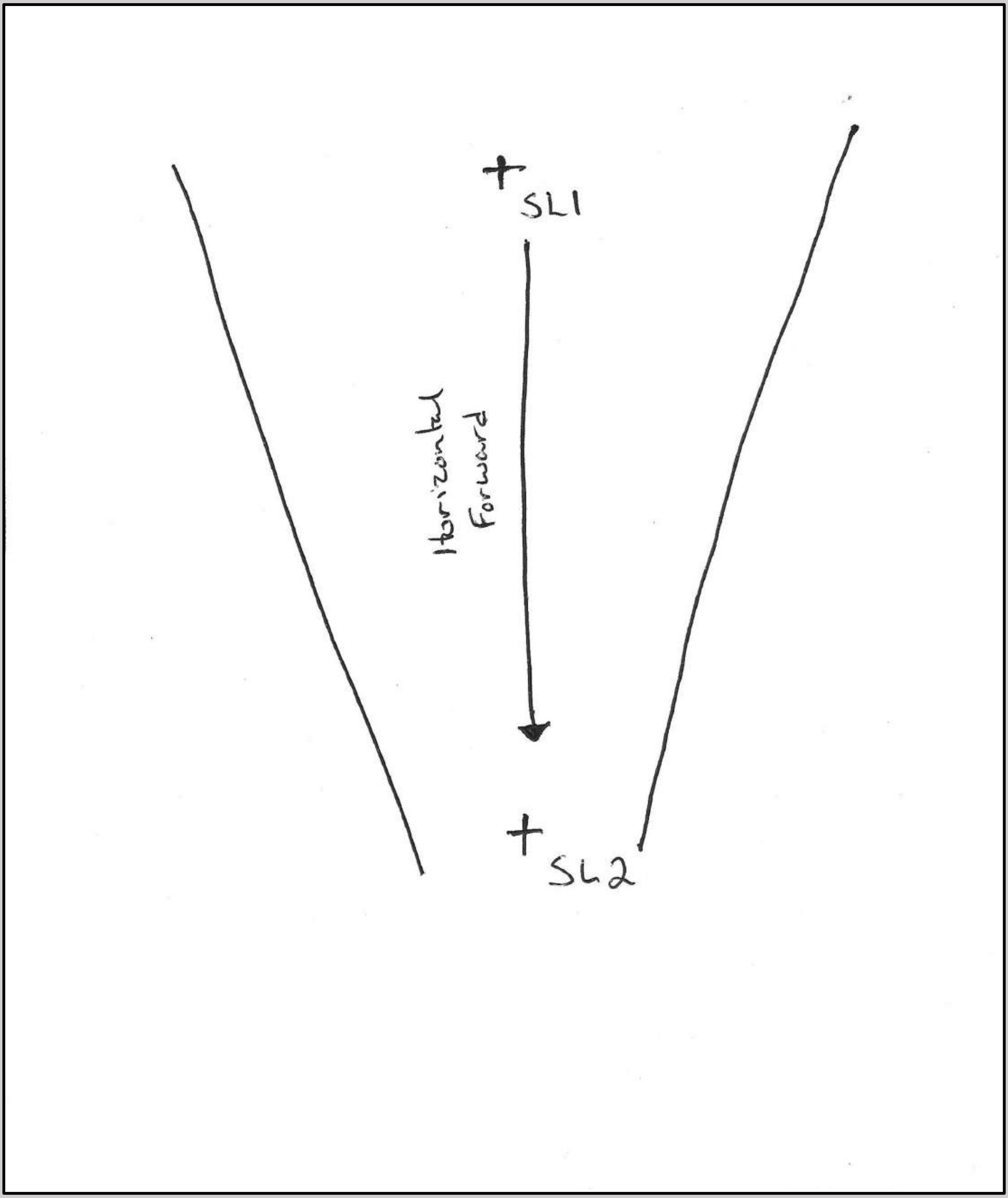

- A9

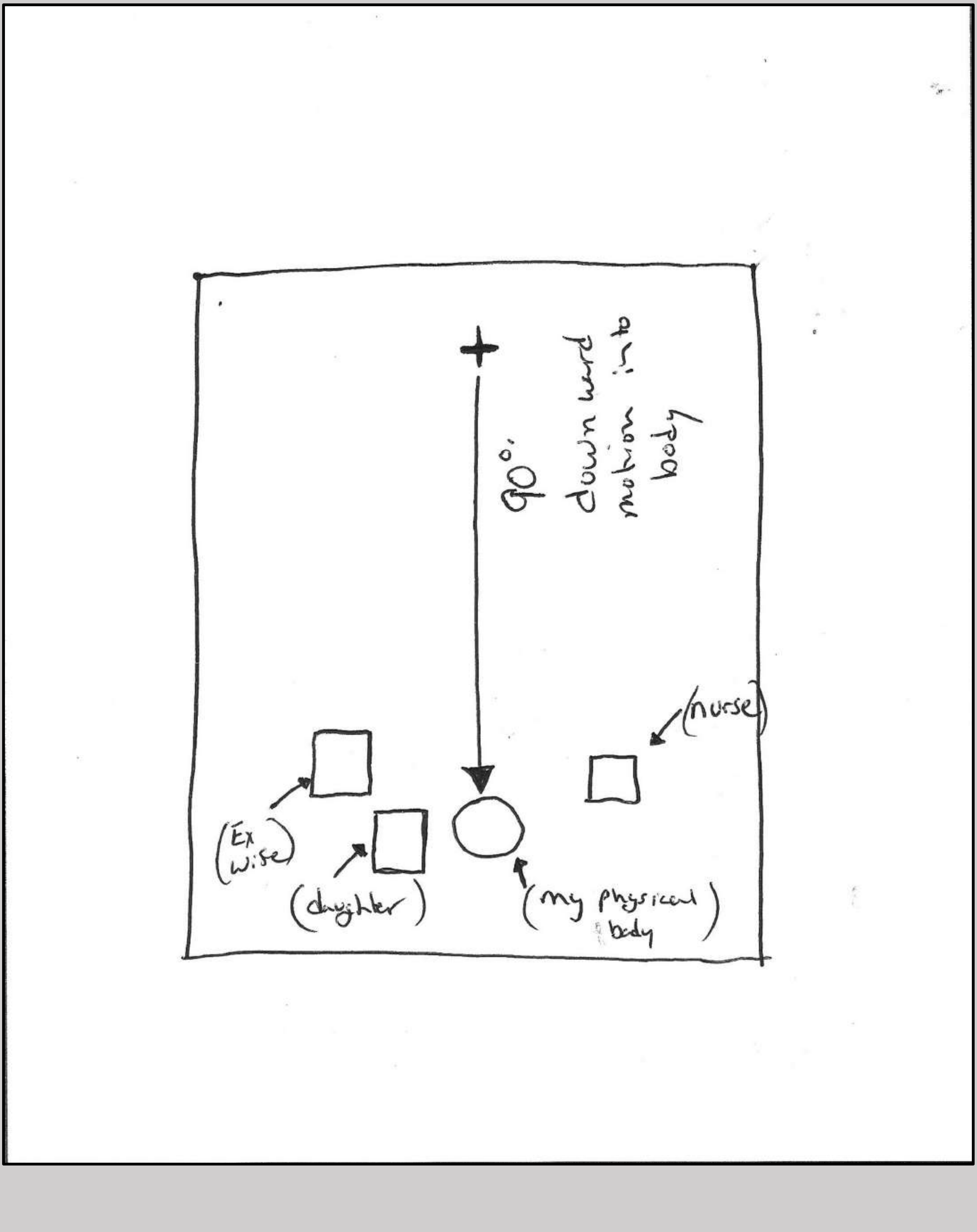

• OBE Associative

Subject 11

Sequence: part/space

2 OBE + 8 NDE

- OBE Dissociative
- A5 (But in his experience summary according to the shape he drew it looks like A8 and not A5)
- C2
- C5
- B6
- C5
- B6
- C5
- B8
- OBE Associative
- OBE/NDE Sequence summary

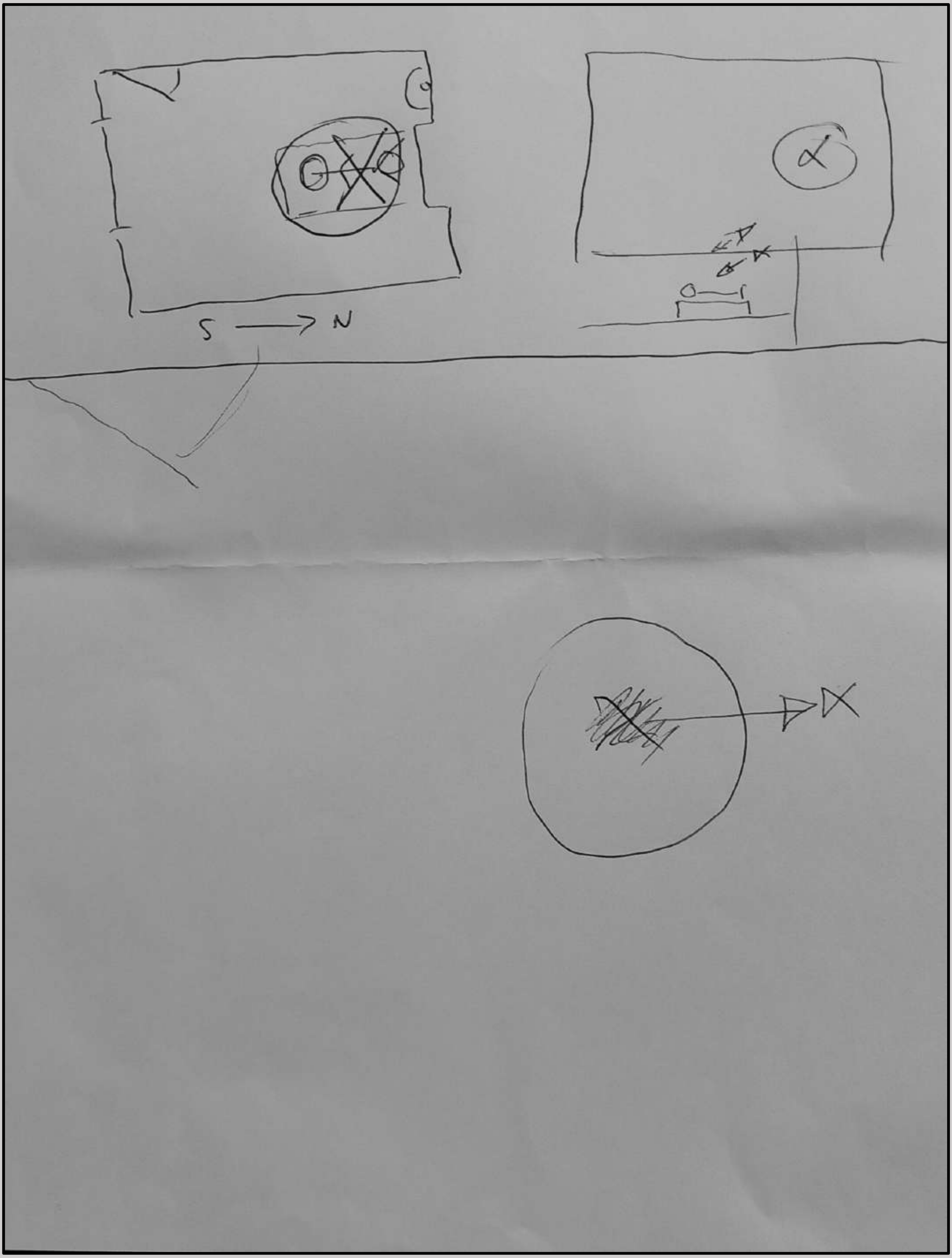

- OBE Dissociative

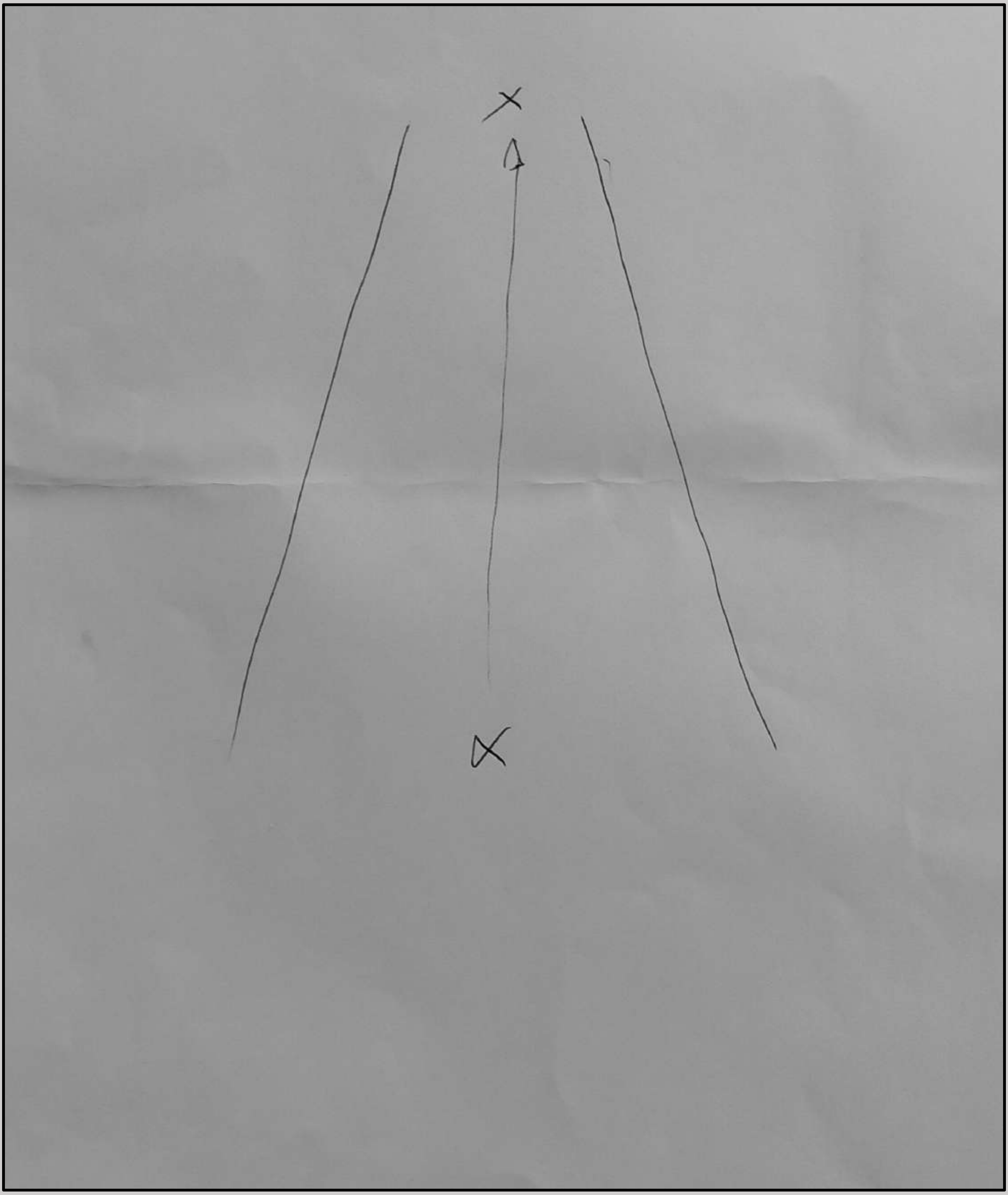

- A5

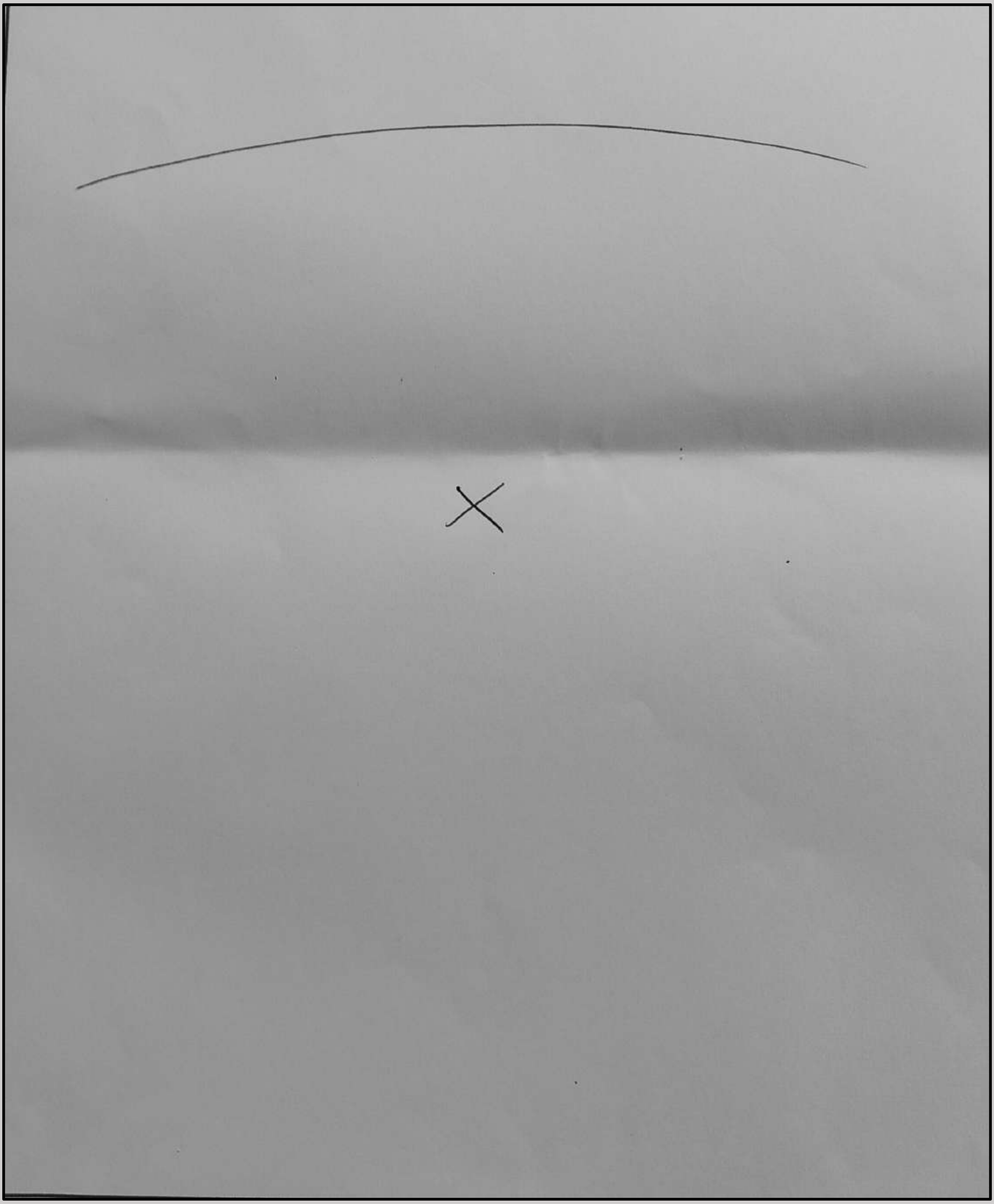

- C2

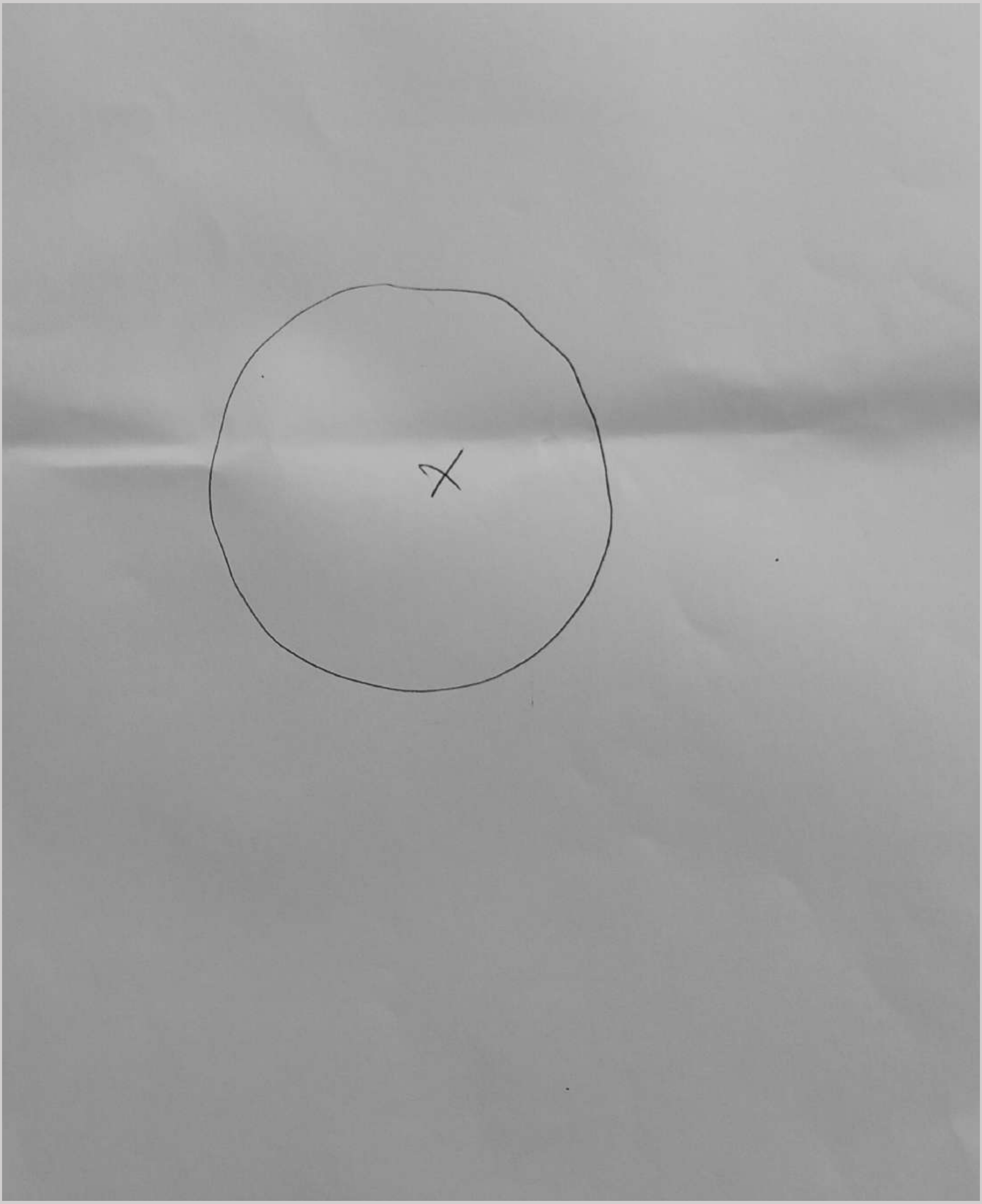

- C5

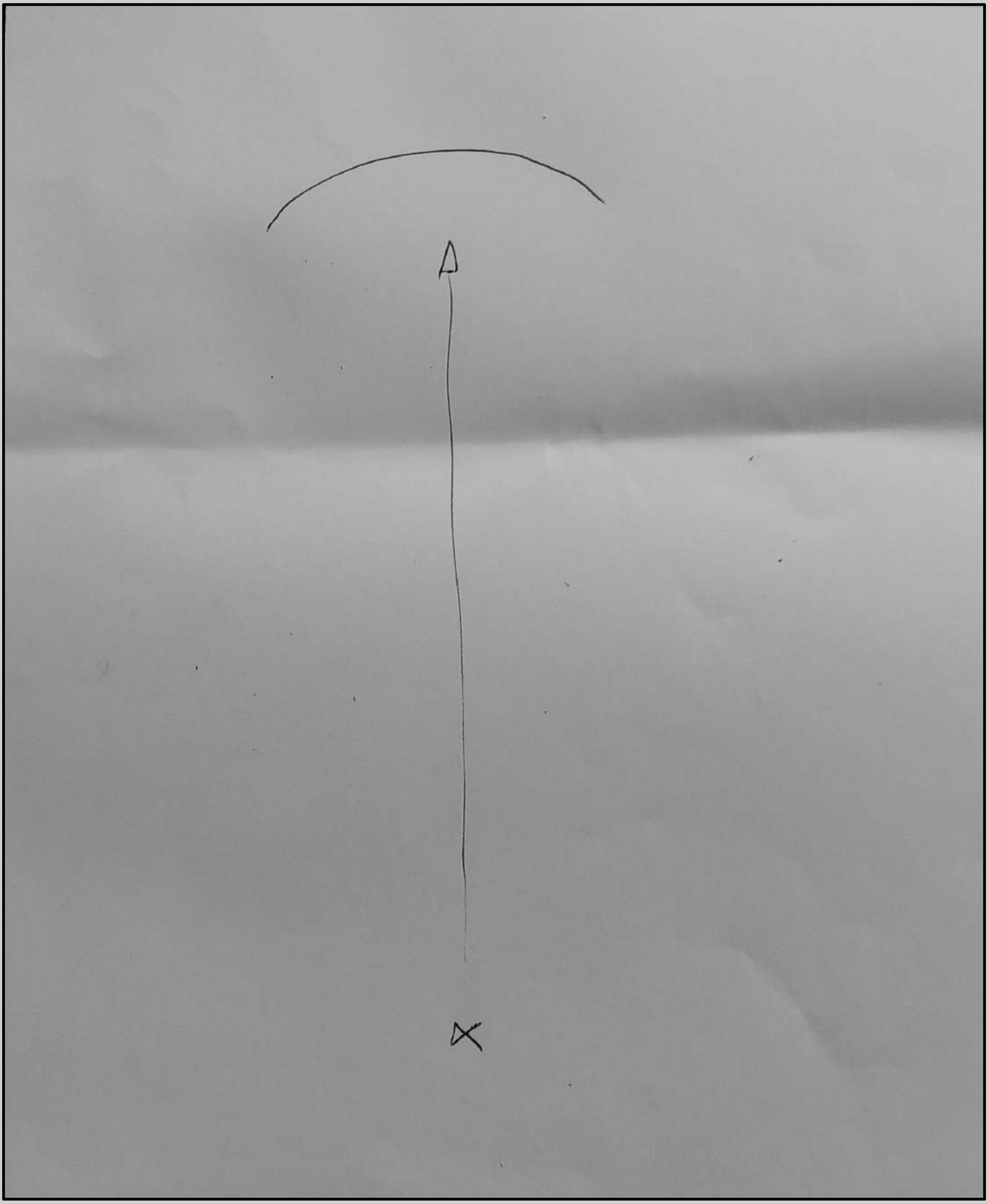

- B6

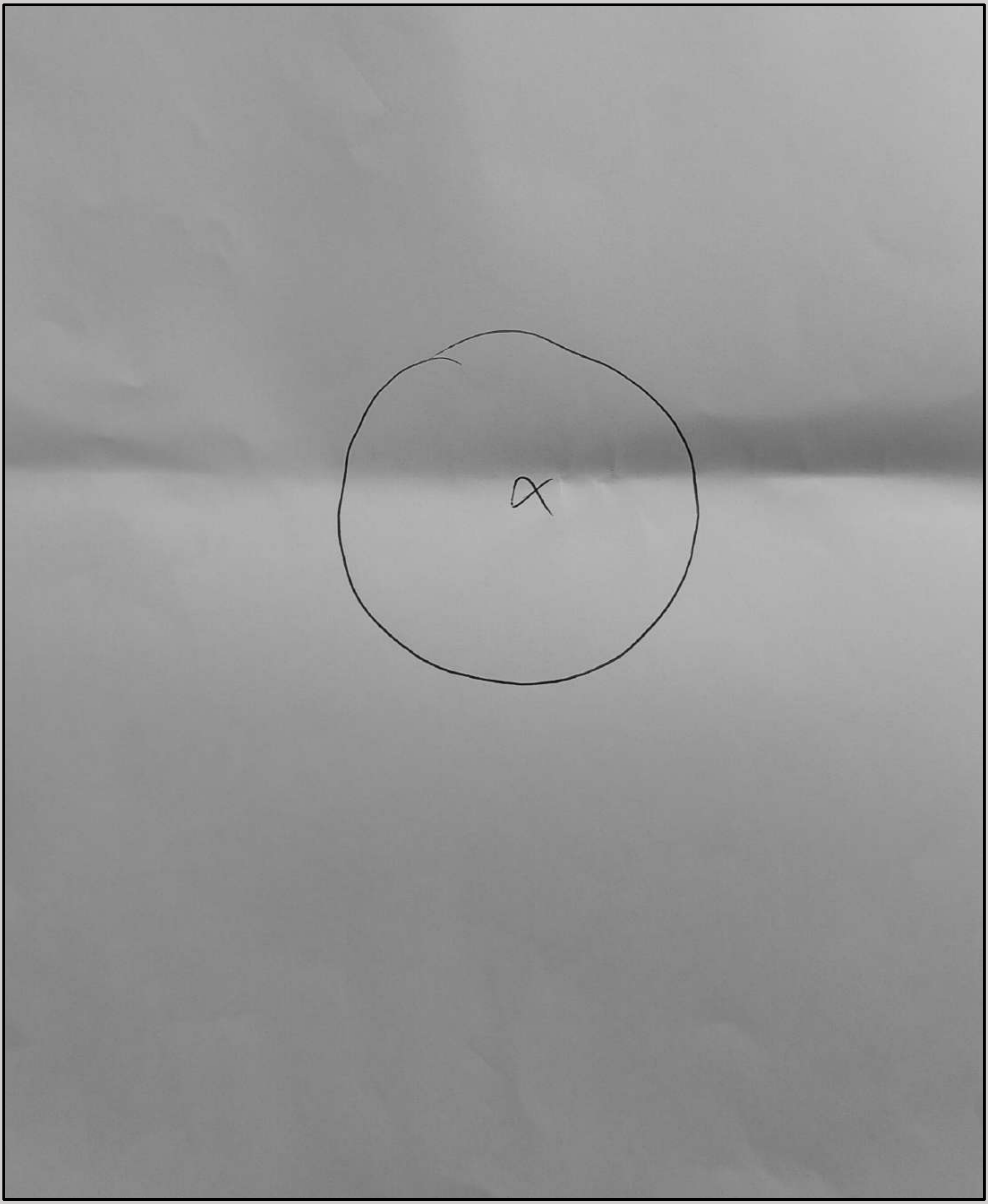

- C5

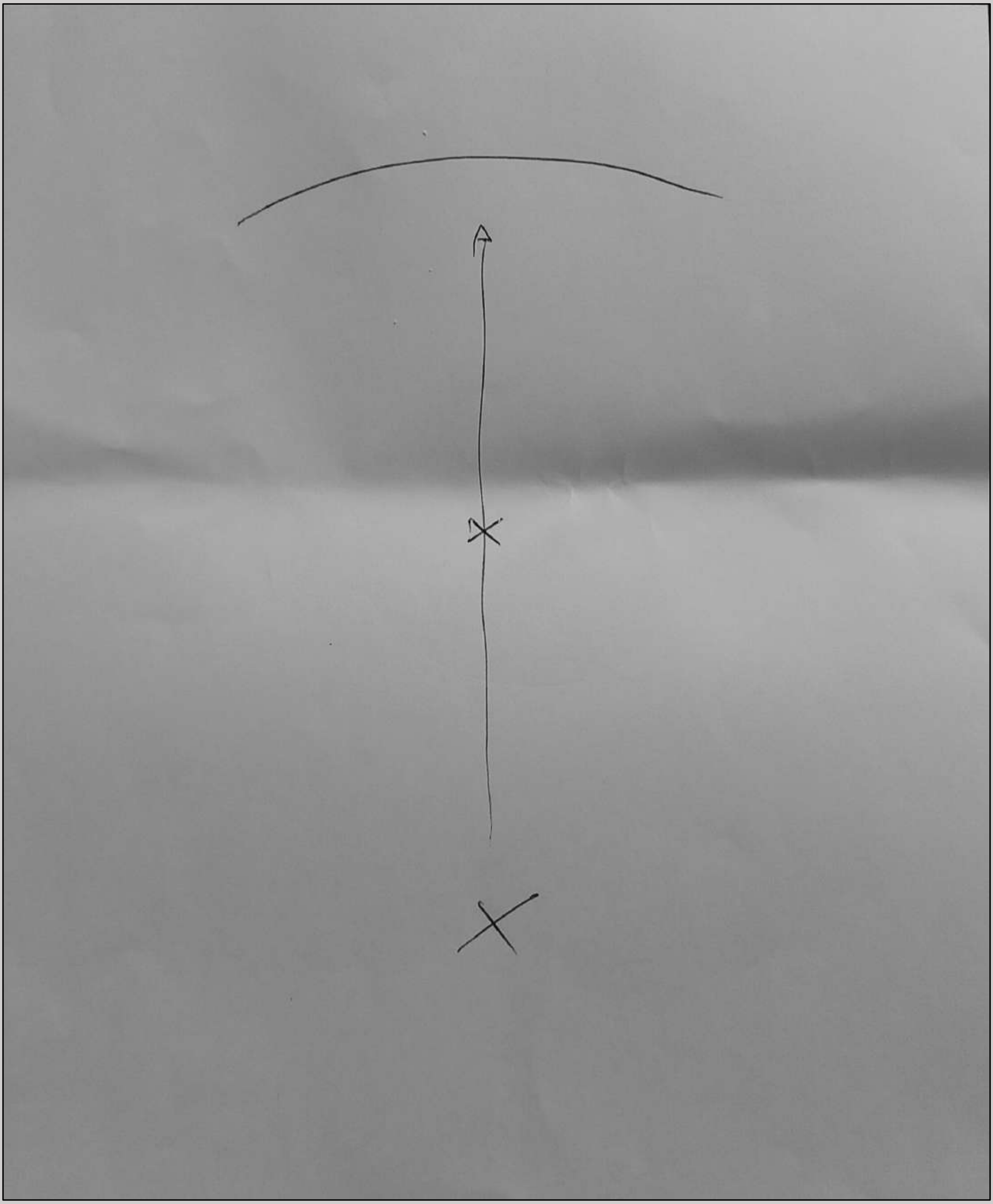

- B6

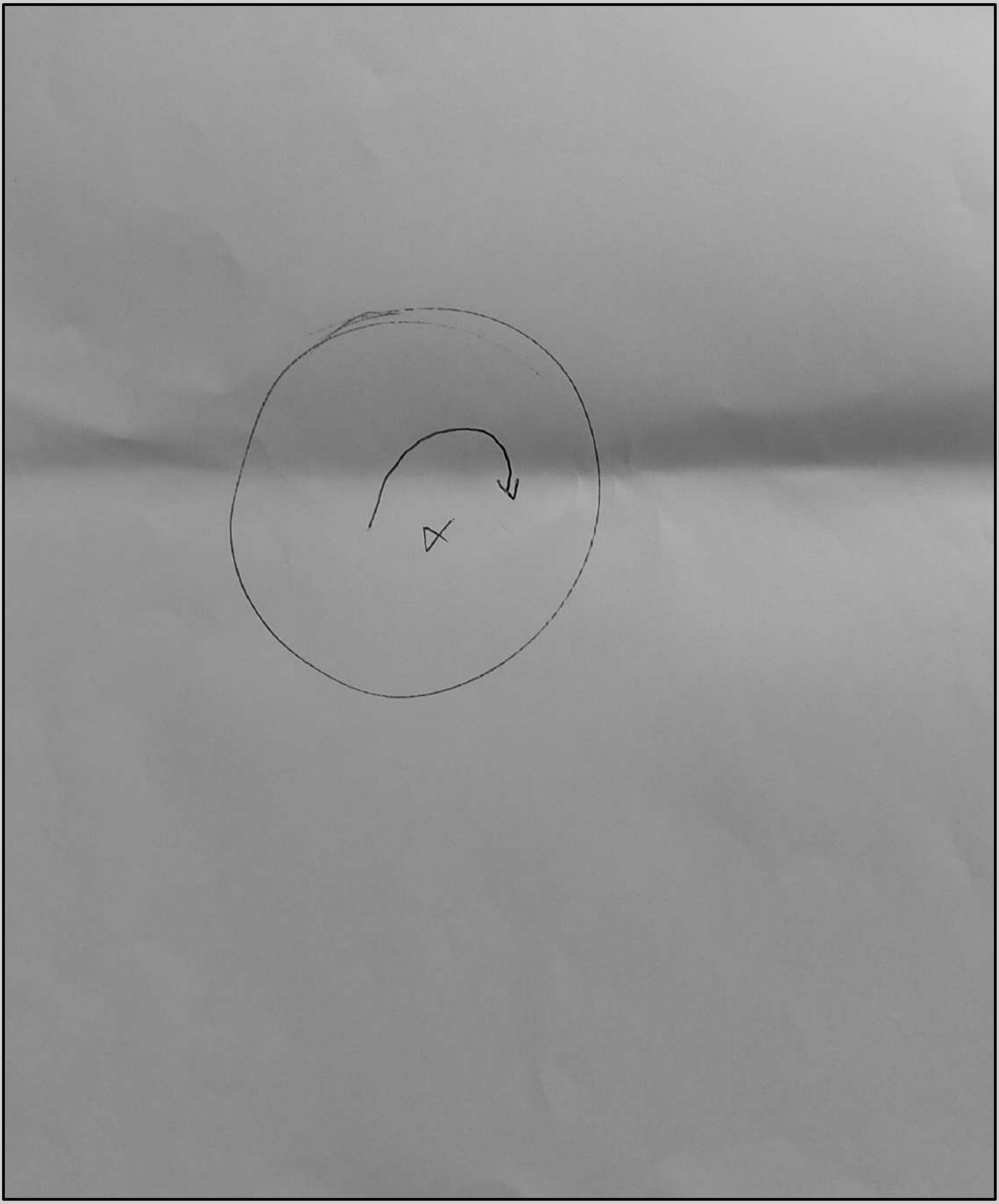

- C5

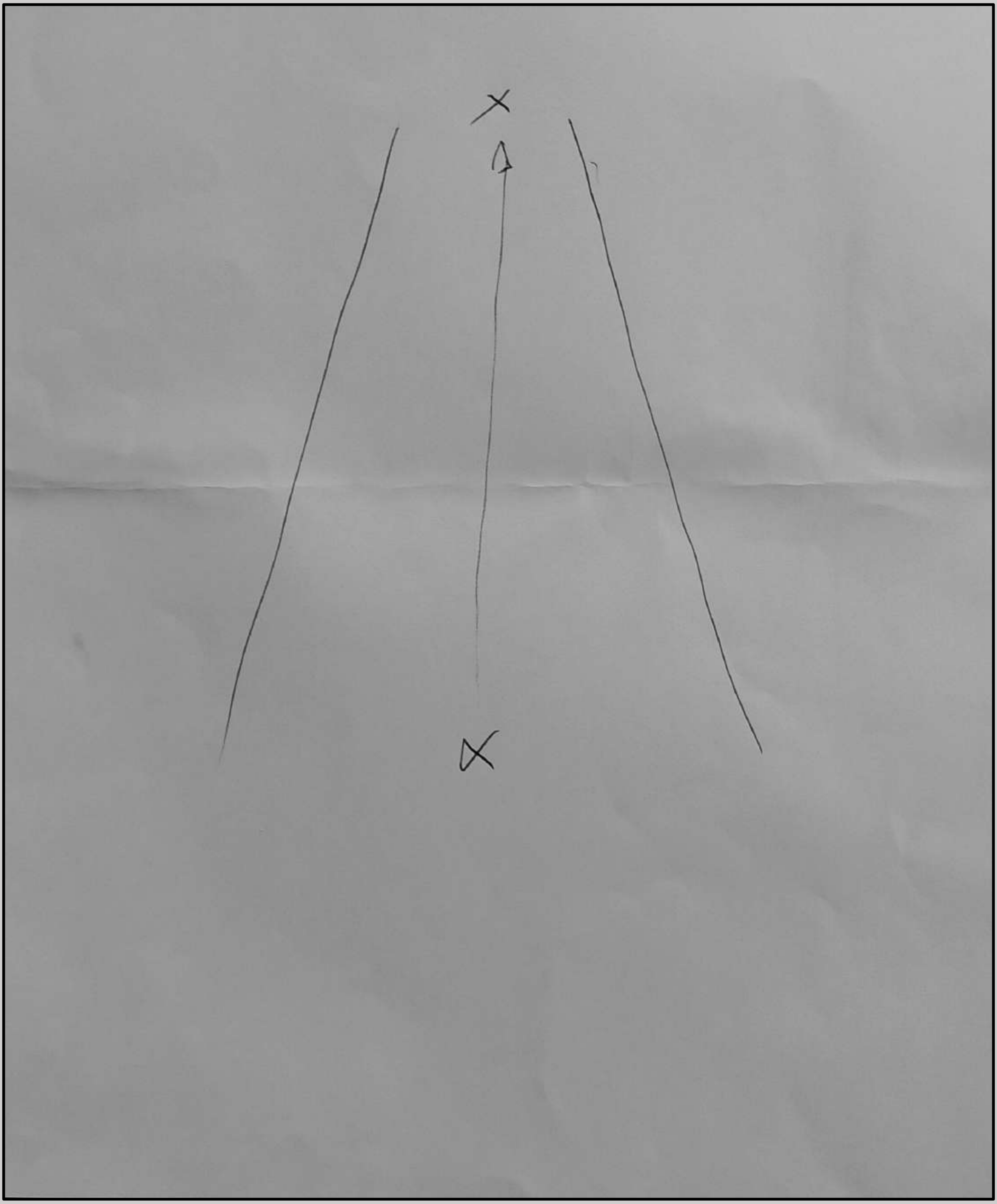

- B8

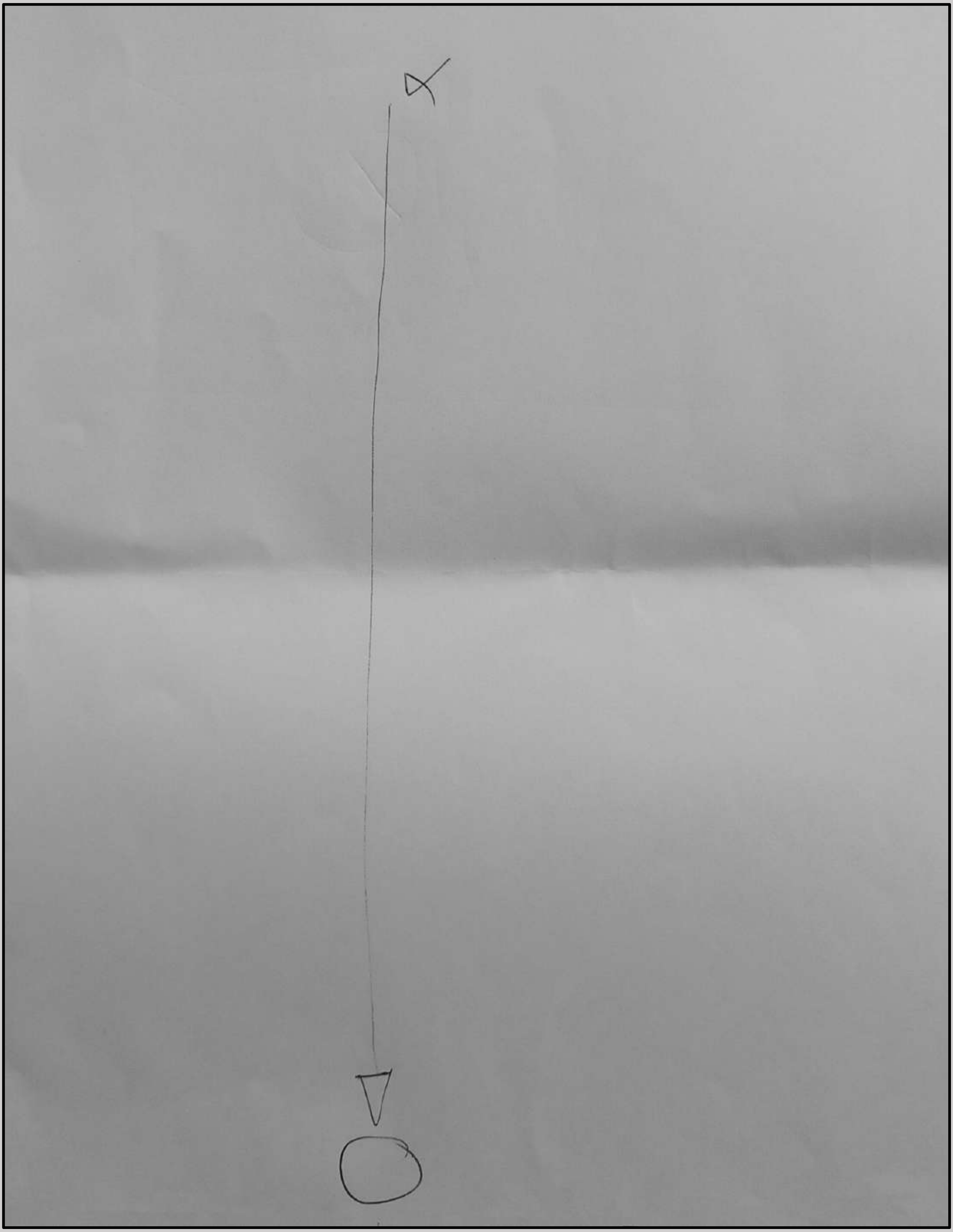

- OBE Associative

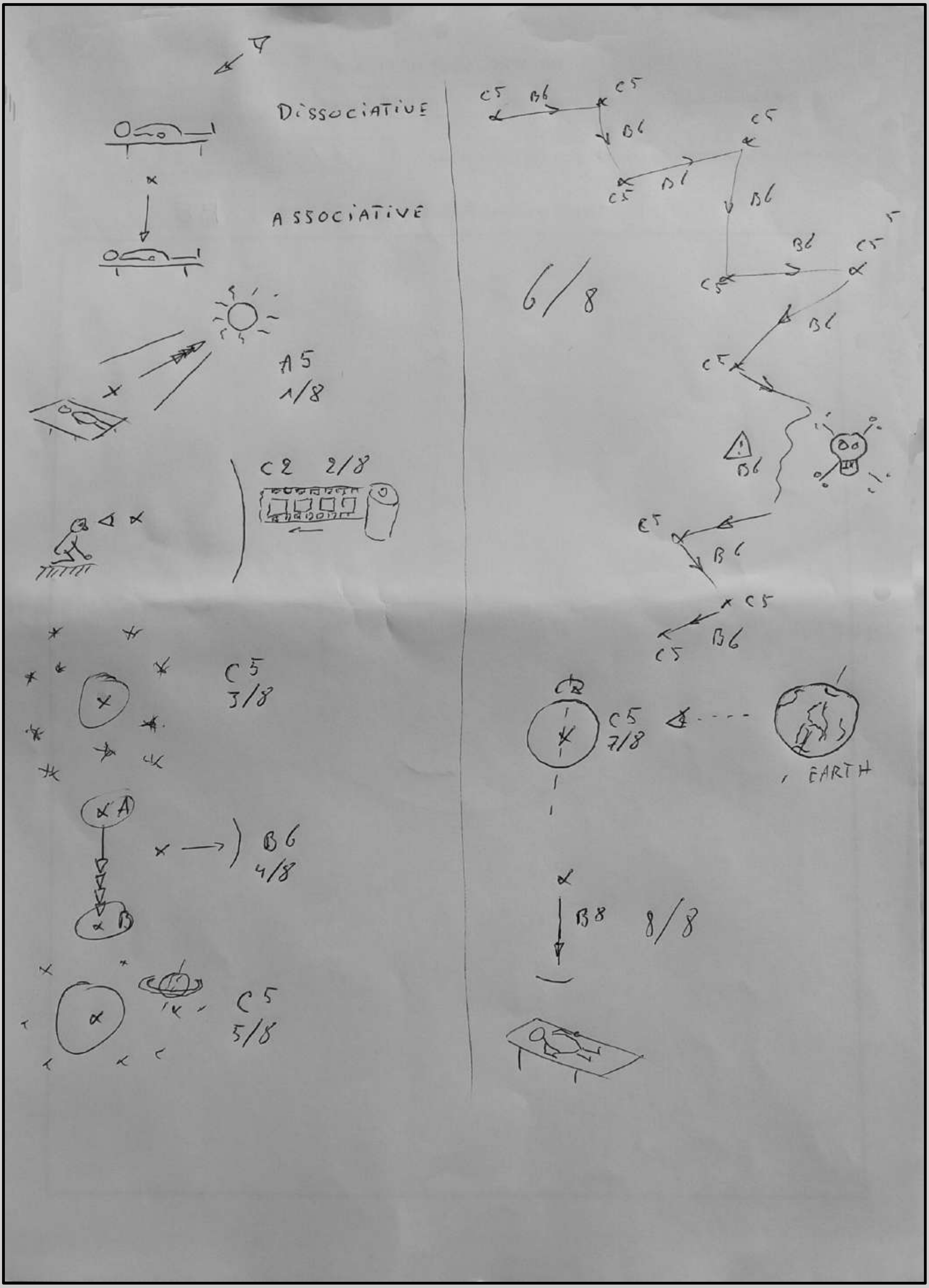

- OBE/NDE Sequence summary

Subject 12

Sequence: part/space

1 OBE + 3 NDE

- A5
- B9
- C2
- OBE Associative

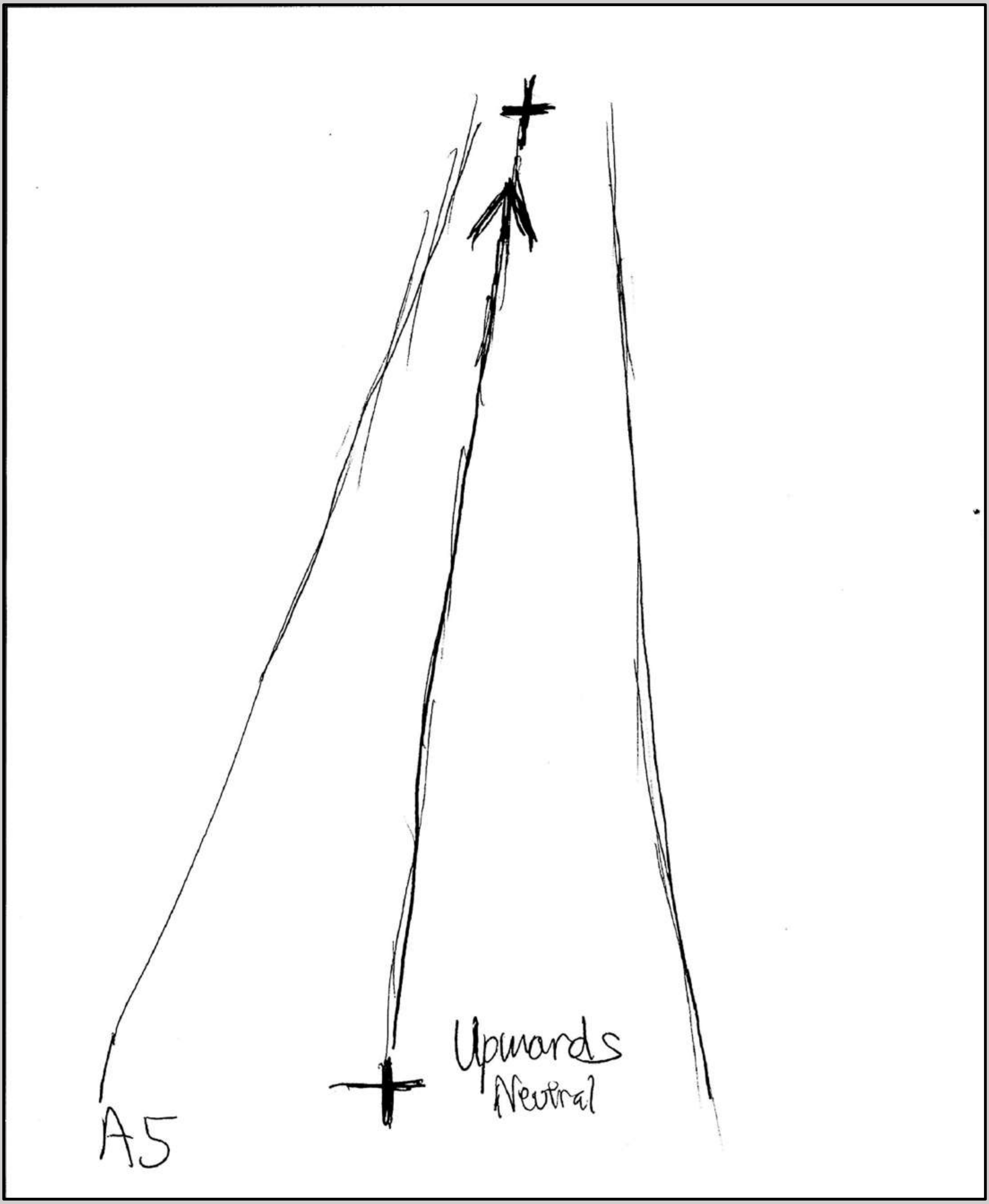

- A5

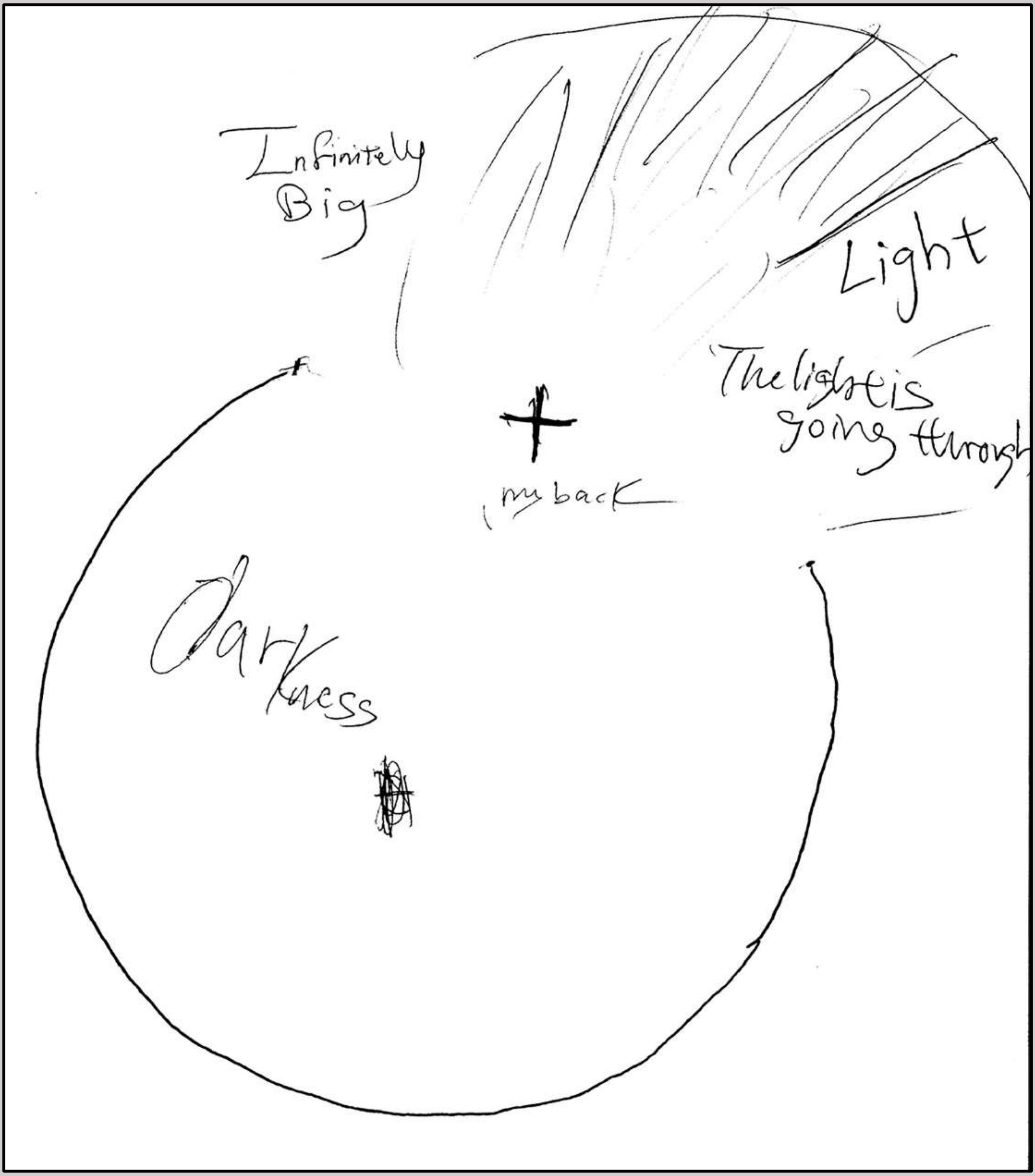
Infinitely Big
Light
The light is going through
my back
darkness

- B9

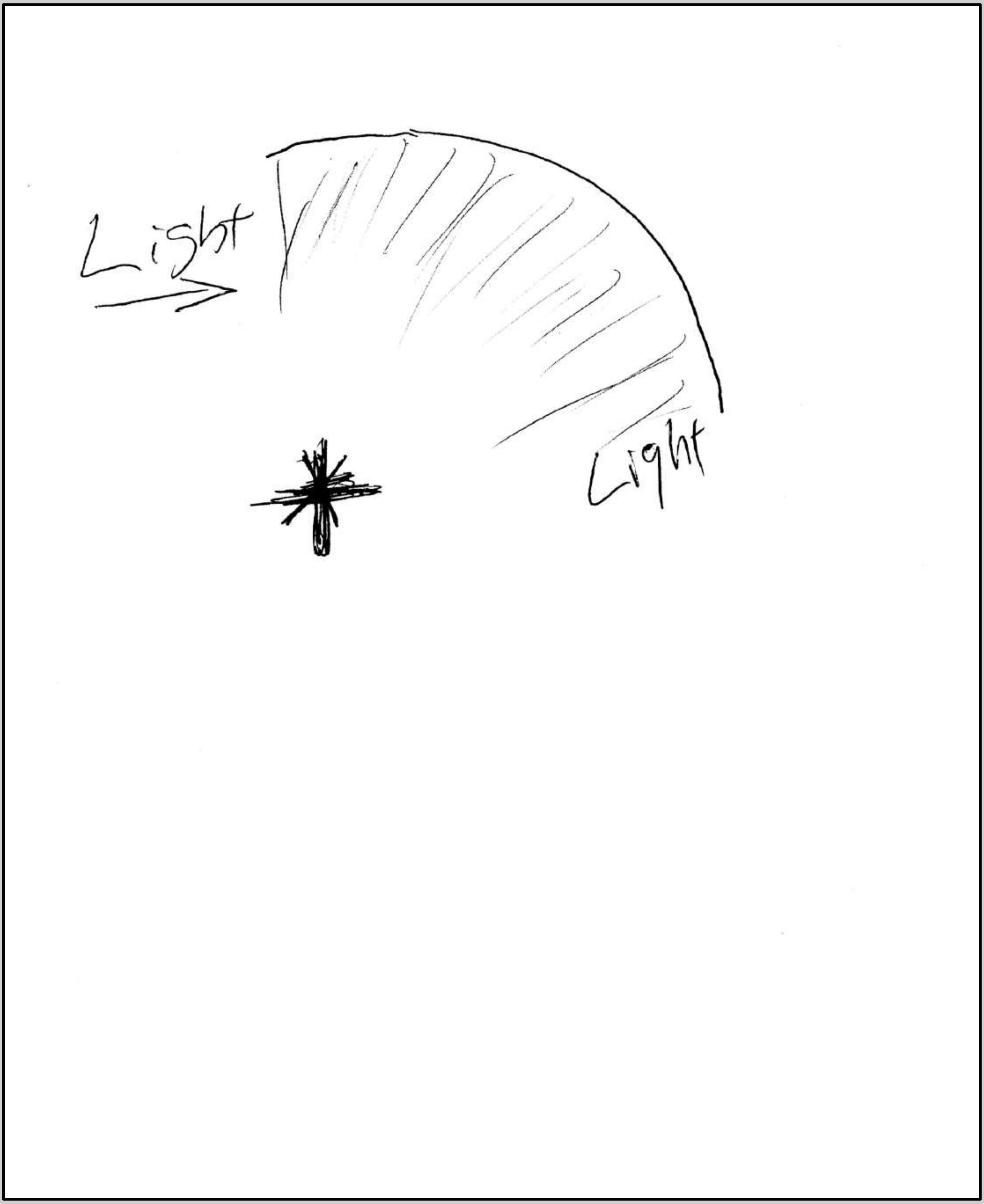

• C2

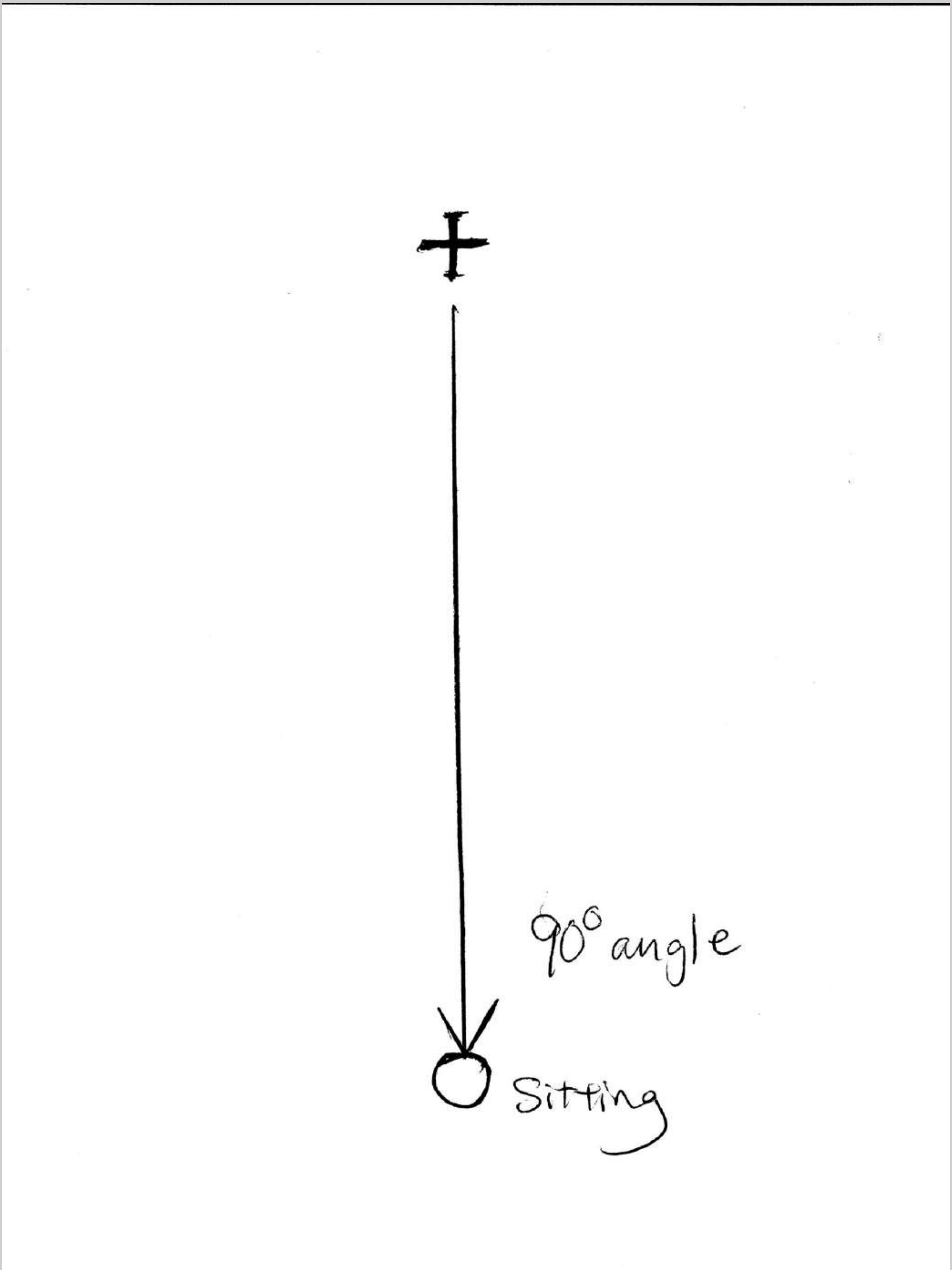

- OBE Associative

Subject 13

Sequence: part/space

1 OBE + 2 NDE

- OBE Dissociative
- A5
- B6

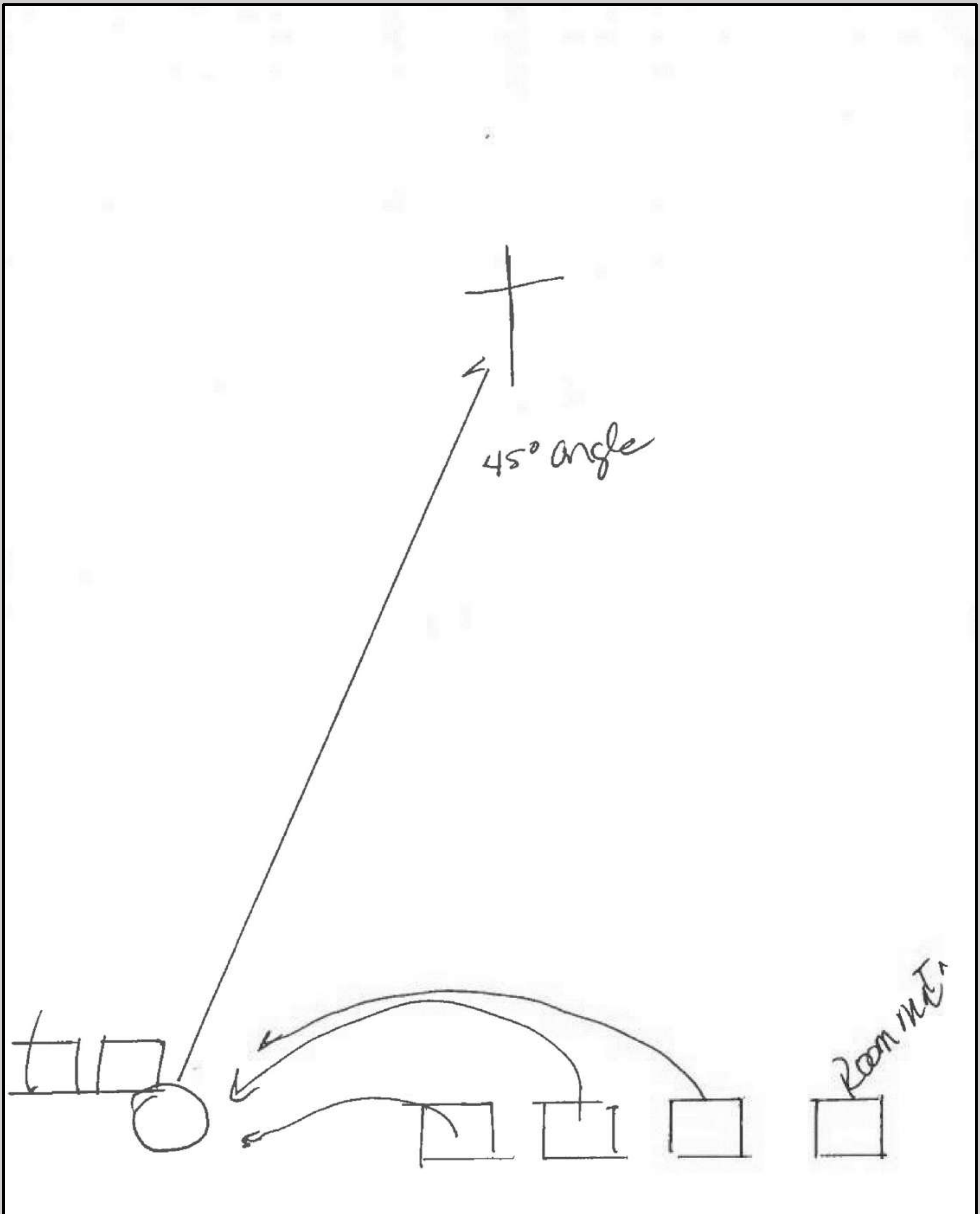

- OBE Dissociative

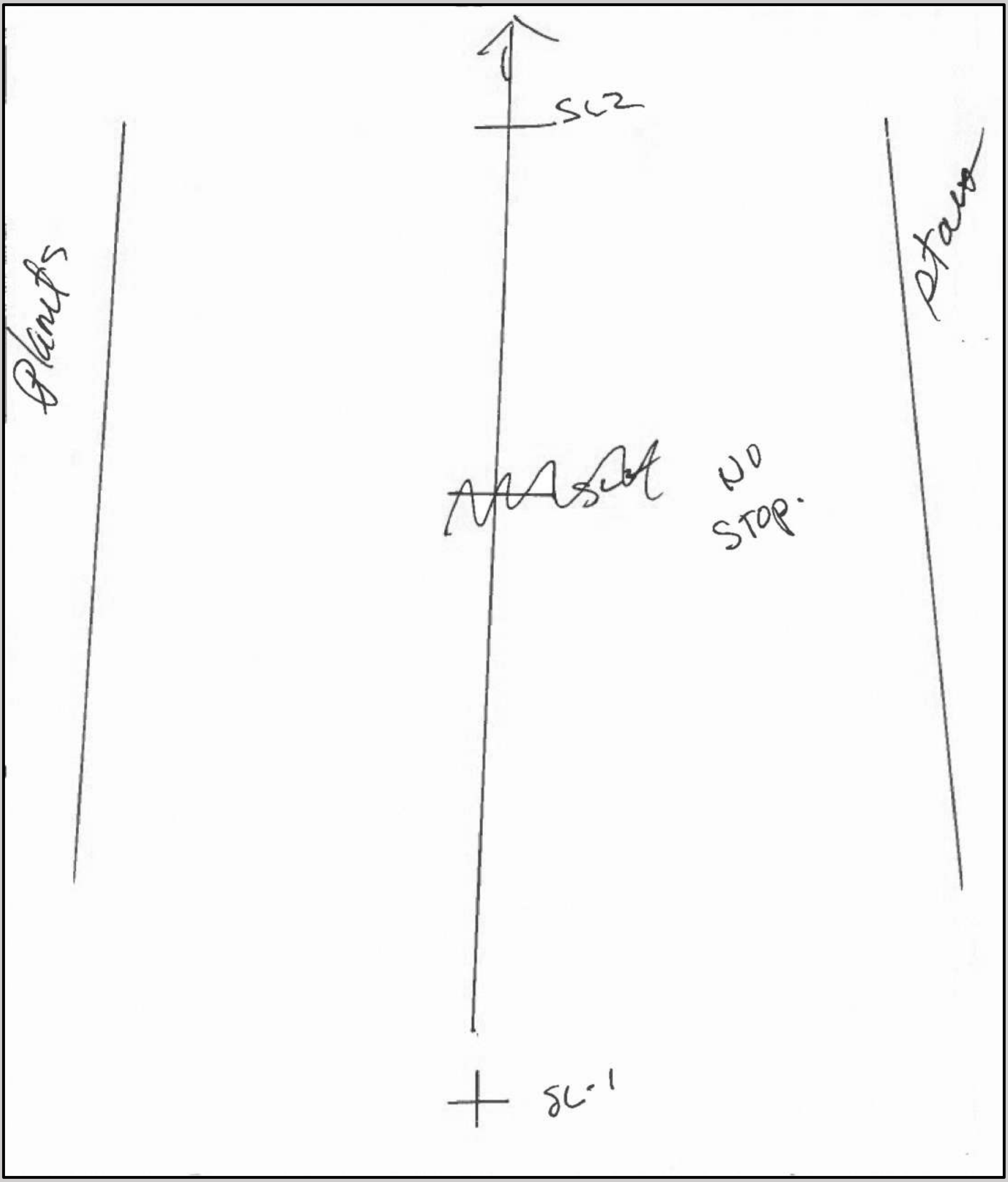

- A5

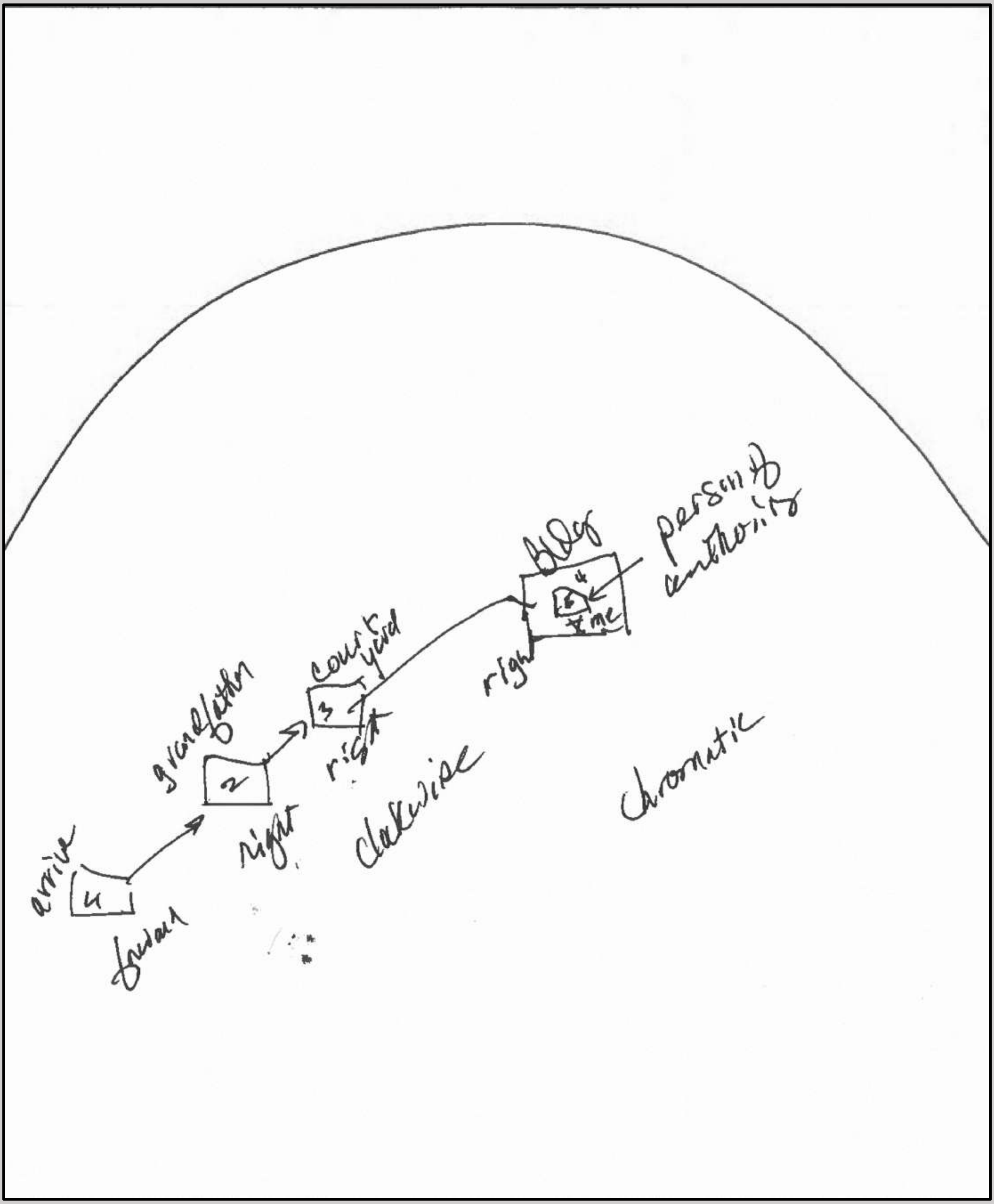

arrive
forward
grandfather
right
court yard
right
clockwise
right
Bldg
person of authority
me
chromatic

- B6

Subject 14

Sequence: part/space

2 OBE + 2 NDE

- OBE Dissociative
- A5
- C5
- OBE Associative

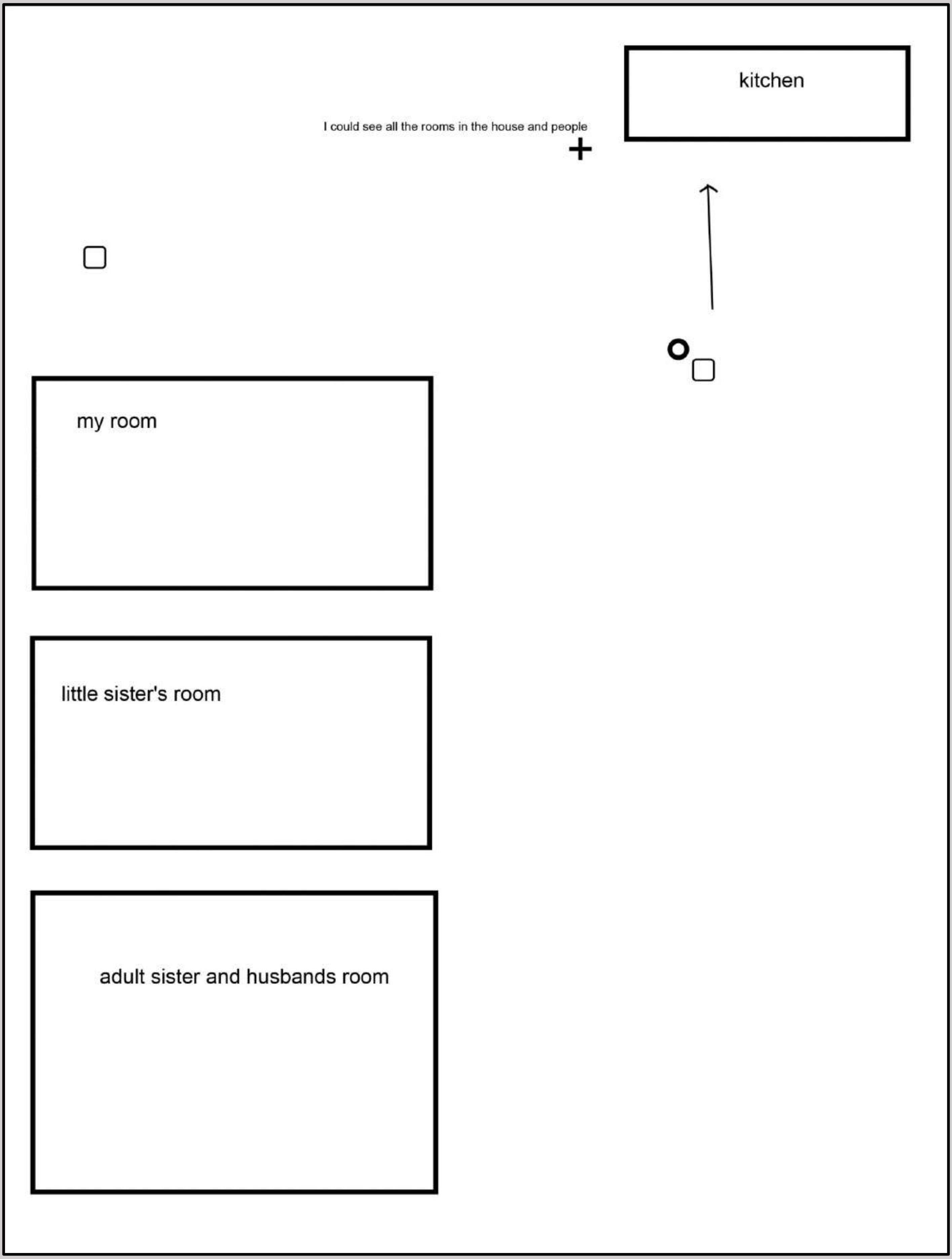

- OBE Dissociative

Horizontal Plane

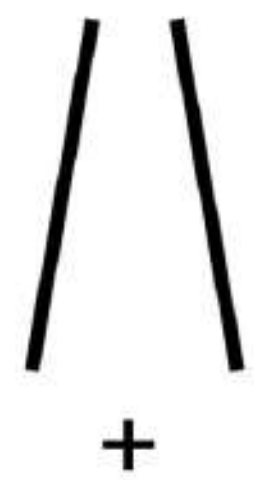

- A5

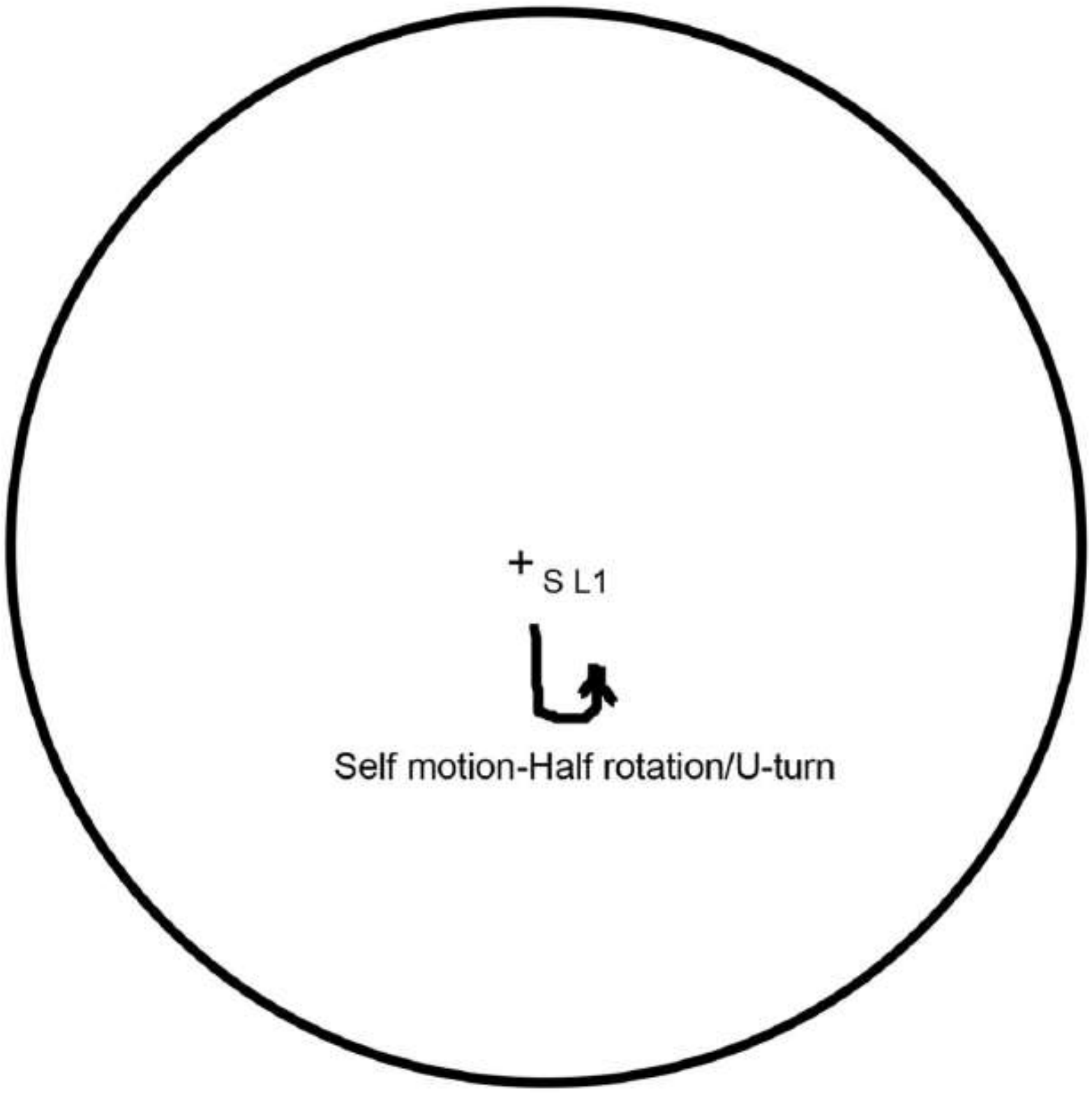

Within this sphere there were more that were vibrating and each got brighter and brighter towards me.

I could feel them, like ripples.

- C5

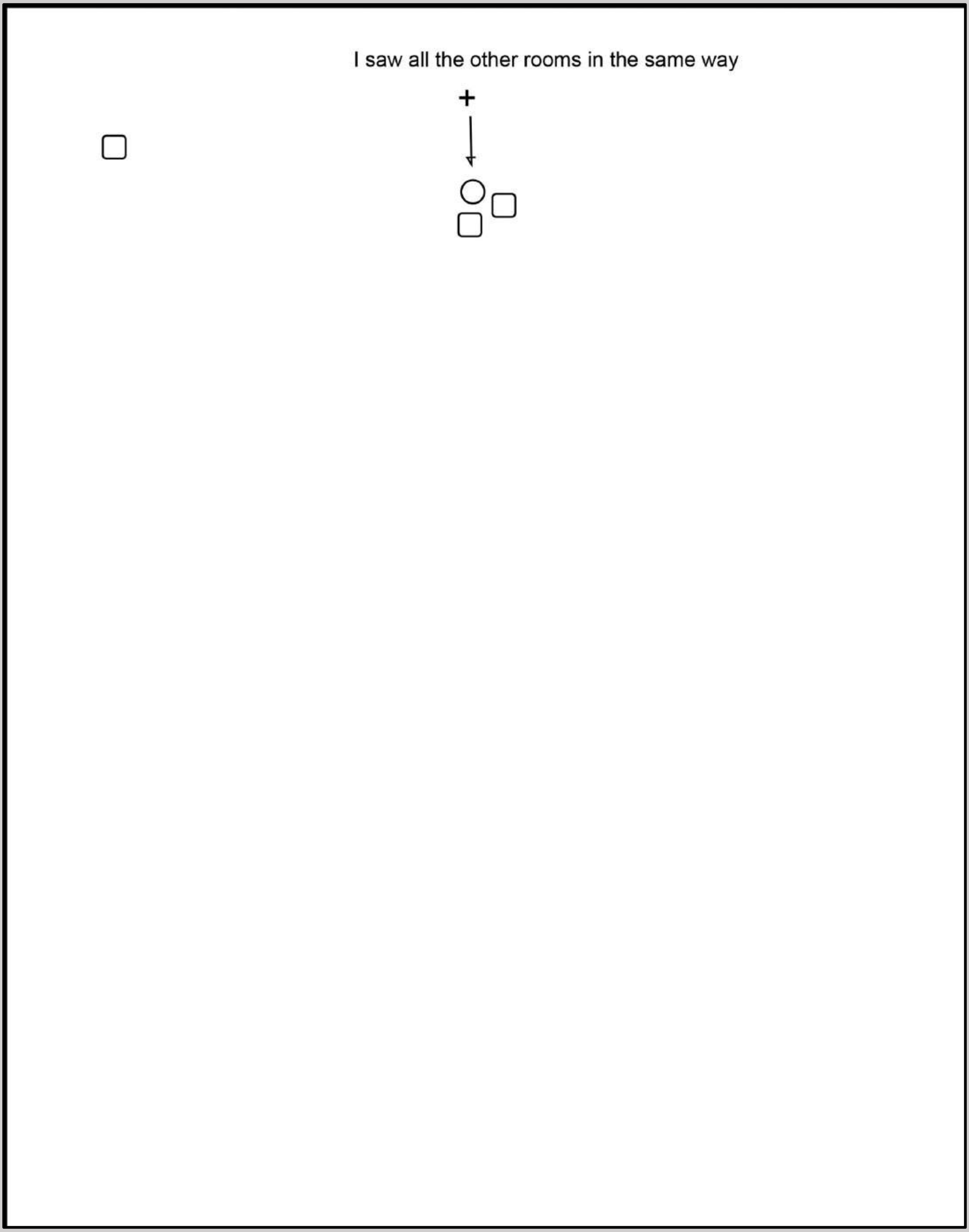

- OBE Associative

Subject 15

Sequence: part/space

1 OBE + 2 NDE

- A5
- B6
- OBE Associative

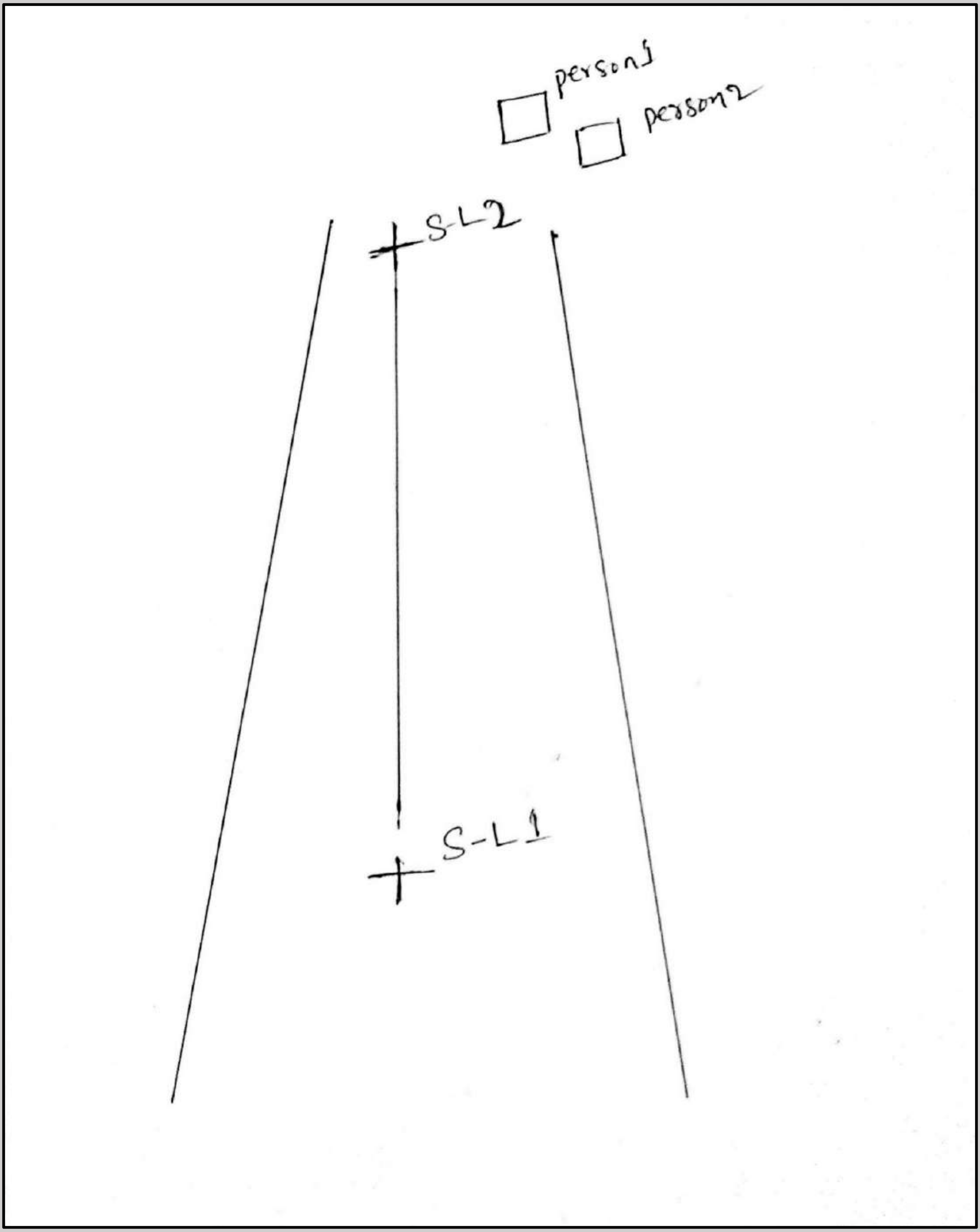

• A5

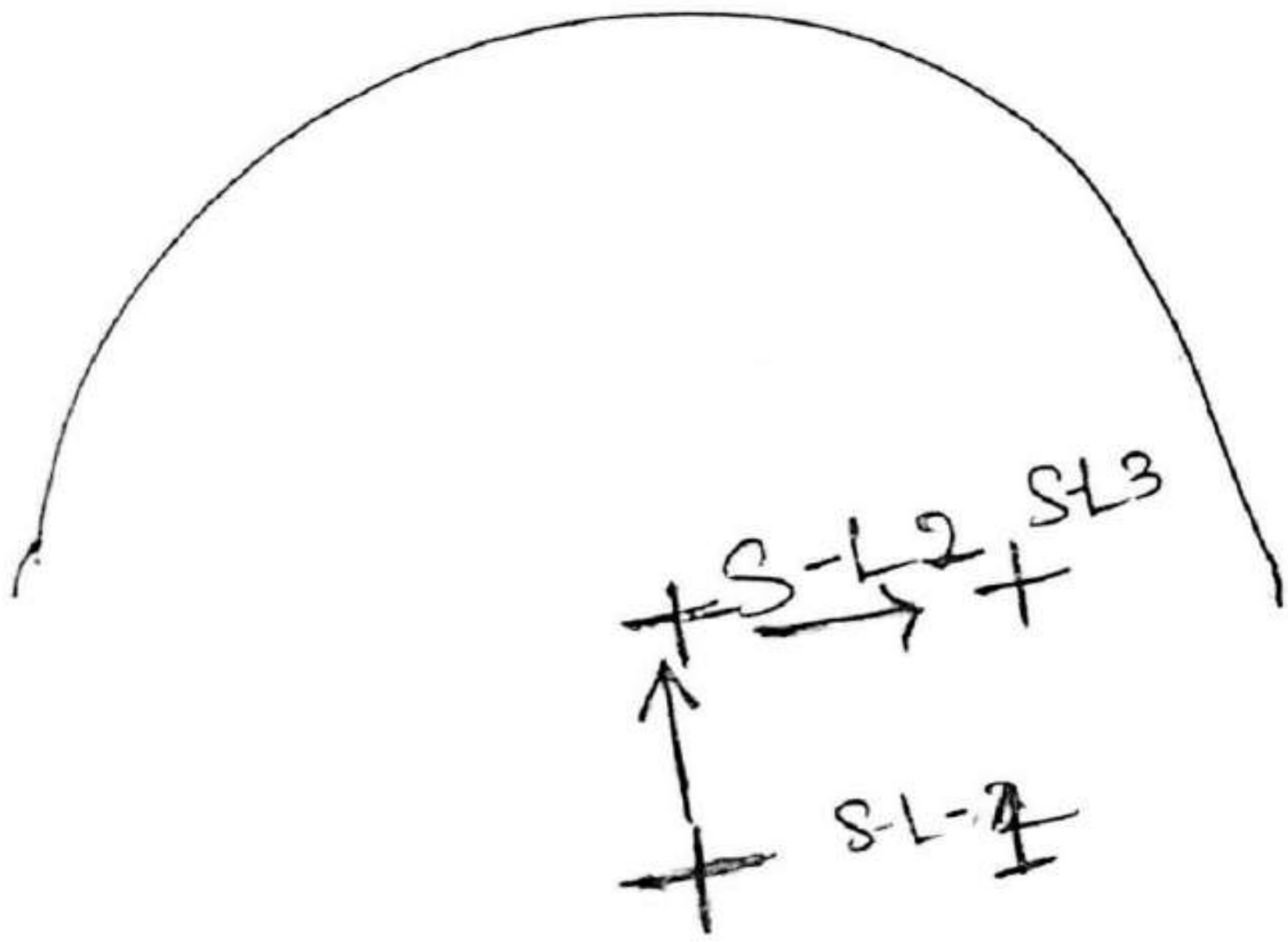

- B6

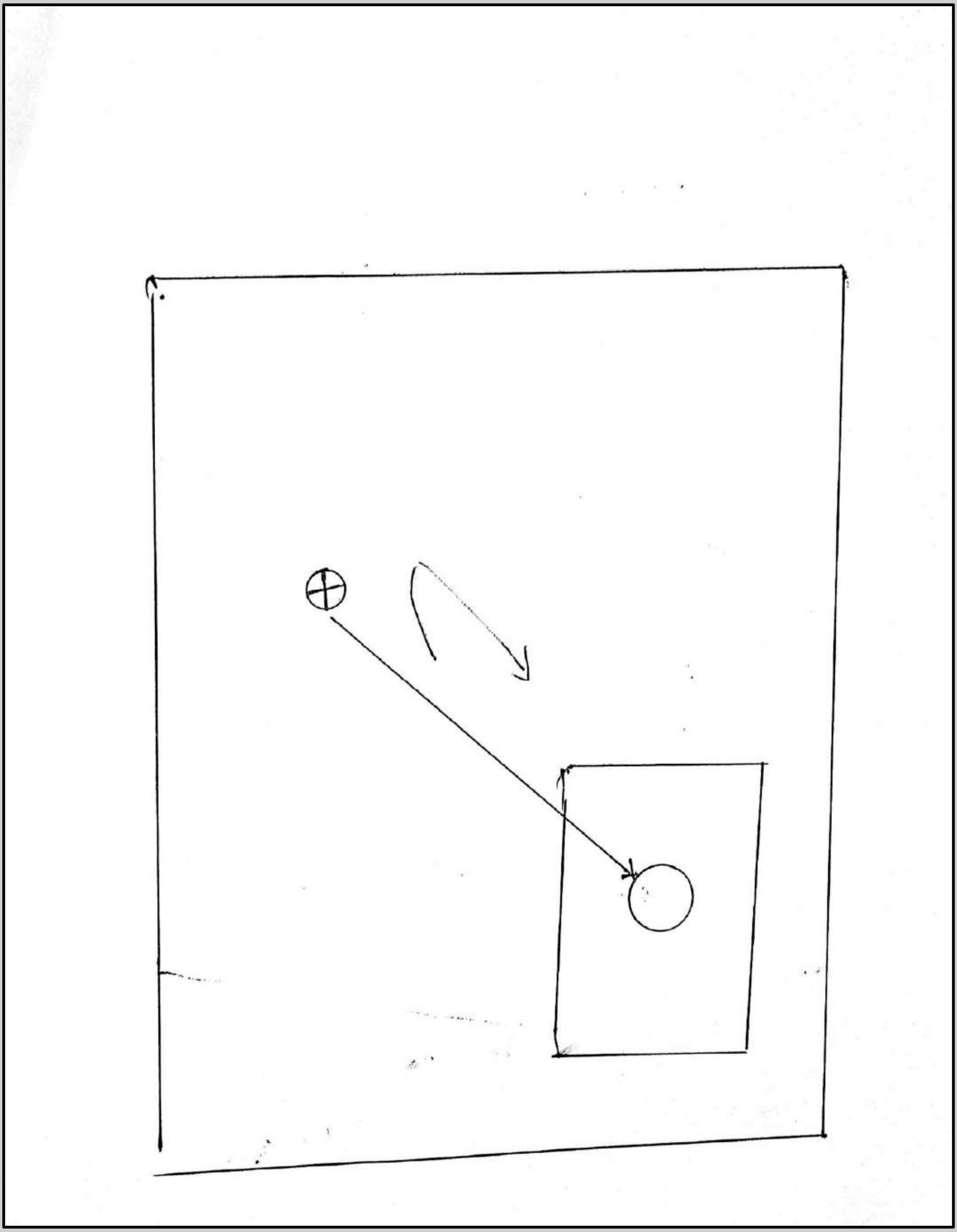

- OBE Associative

Subject 16

Sequence: part/space

1 NDE

- A5, A6, A7, A8, A9, A10, A11, A12

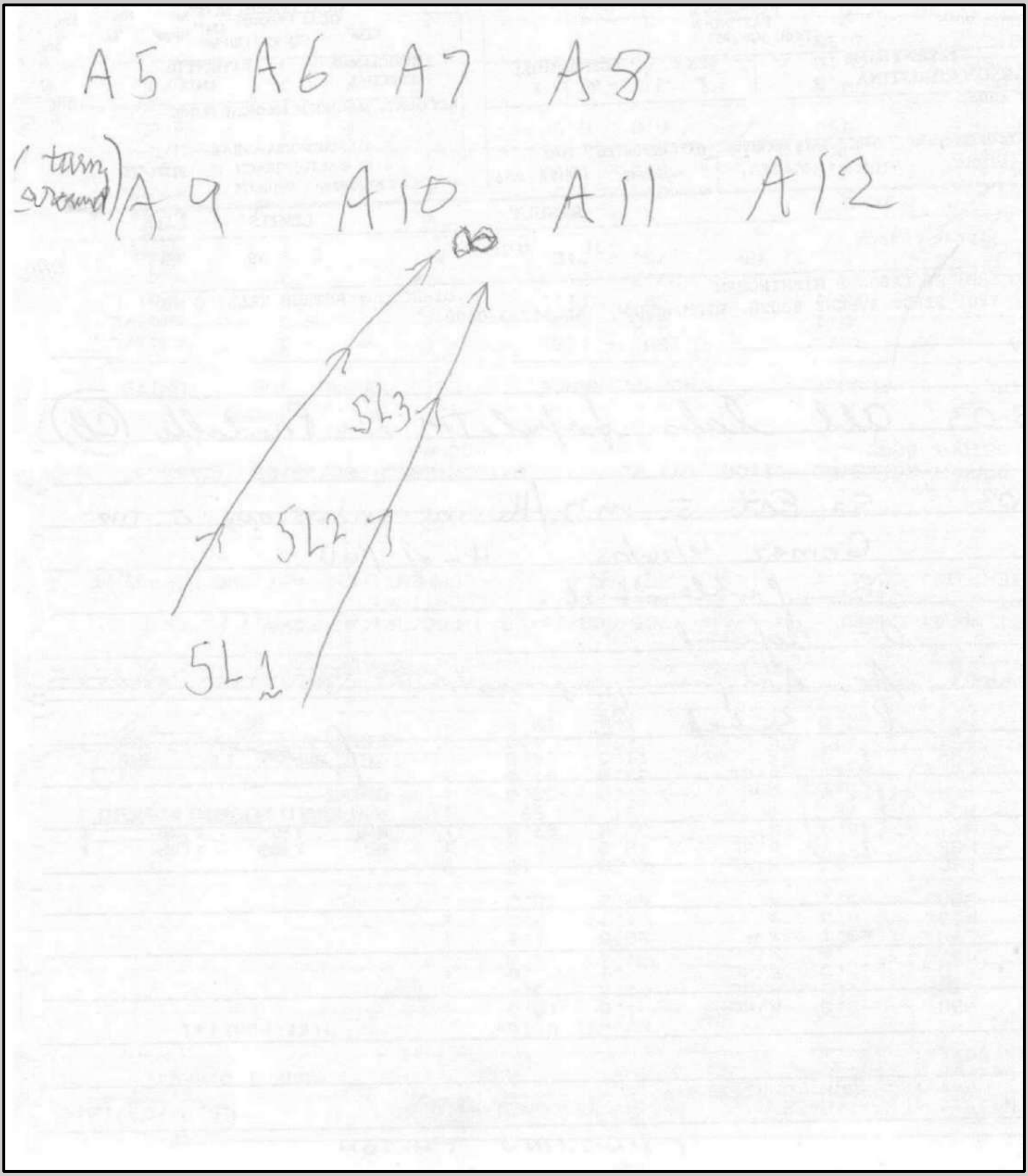

- A5-A 12

Subject 17

Sequence: part/space

1 OBE + 3 NDE

- OBE Dissociative
- A7
- B10
- C5

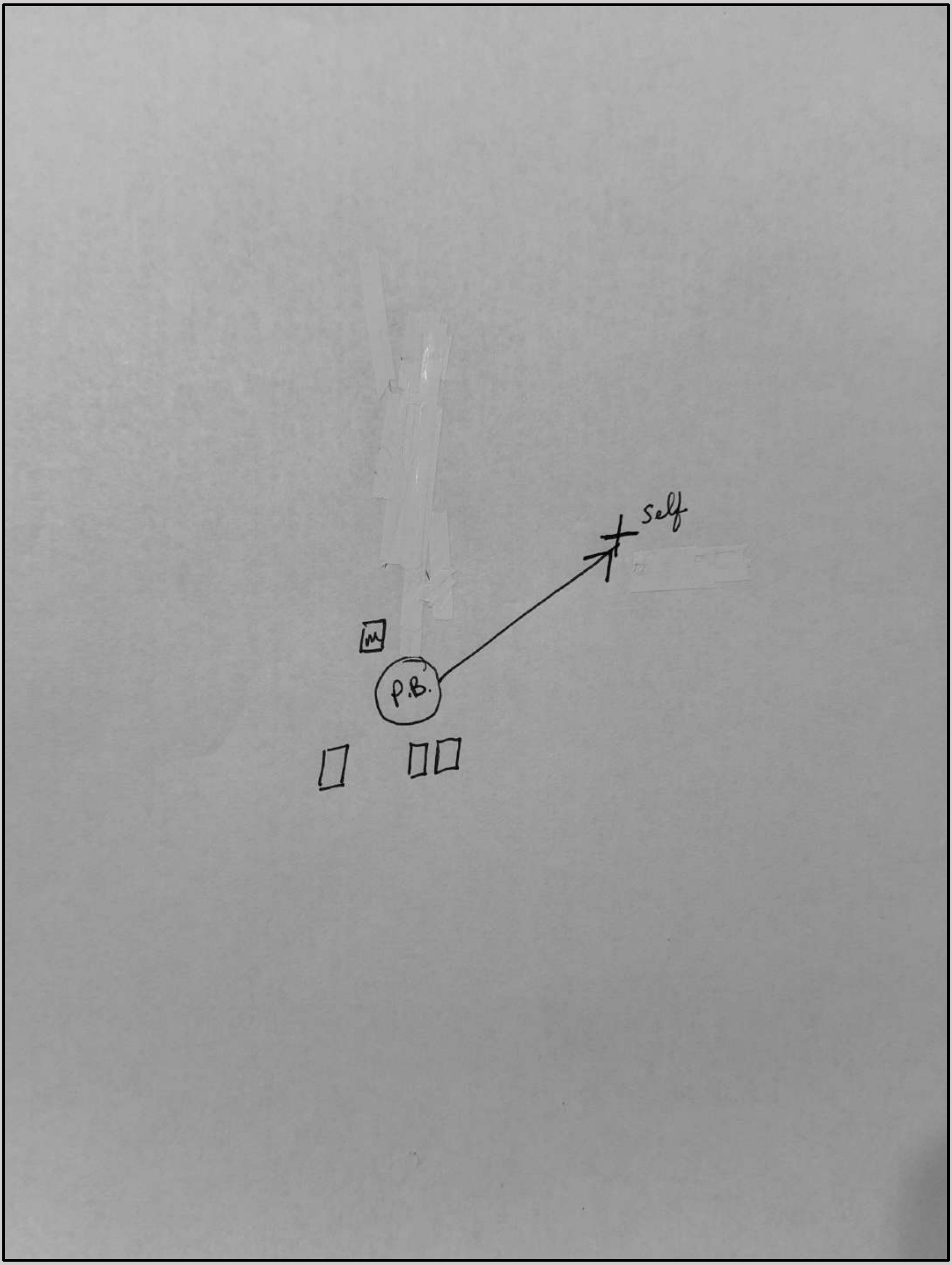

- OBE Dissociative

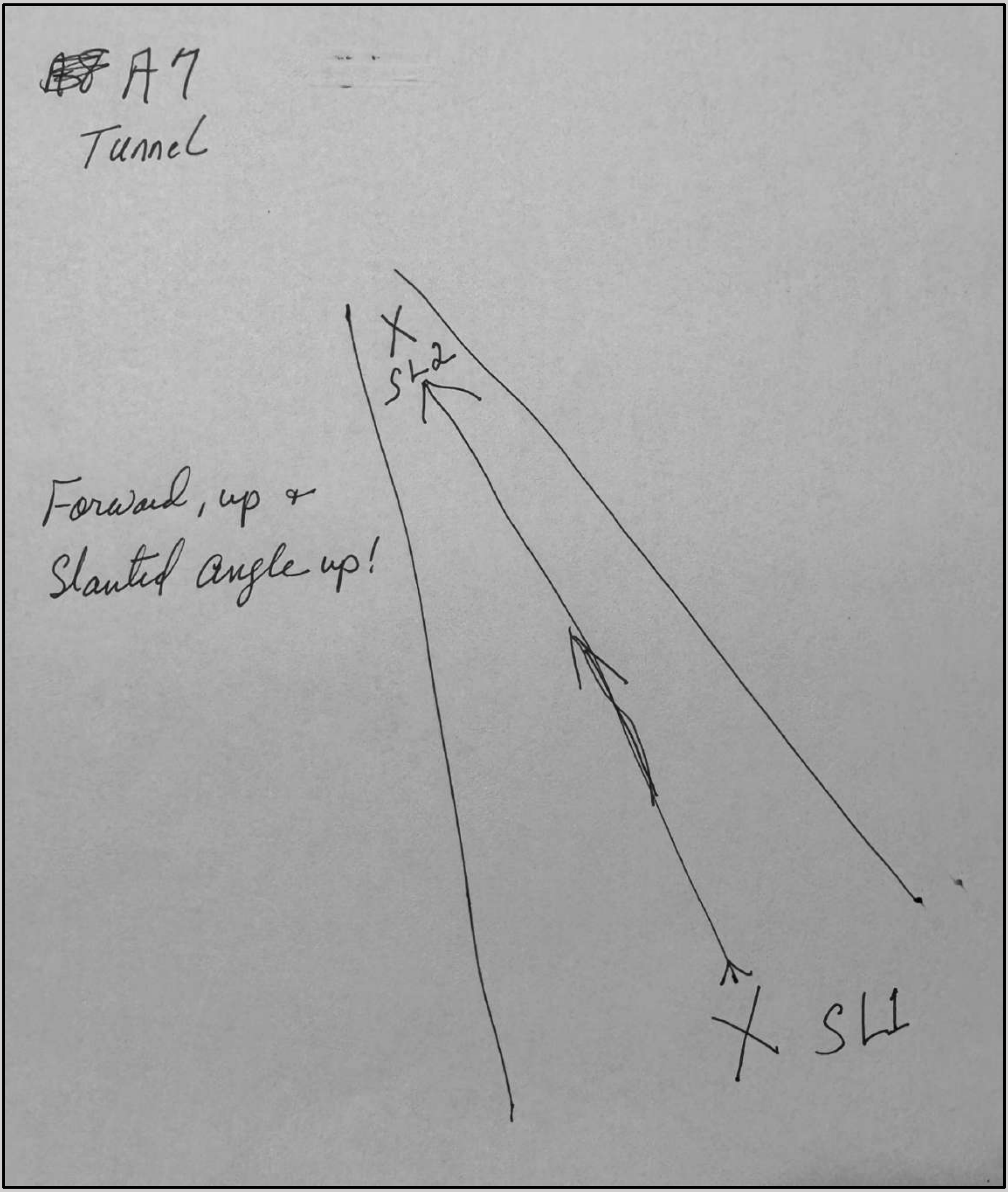

- A7

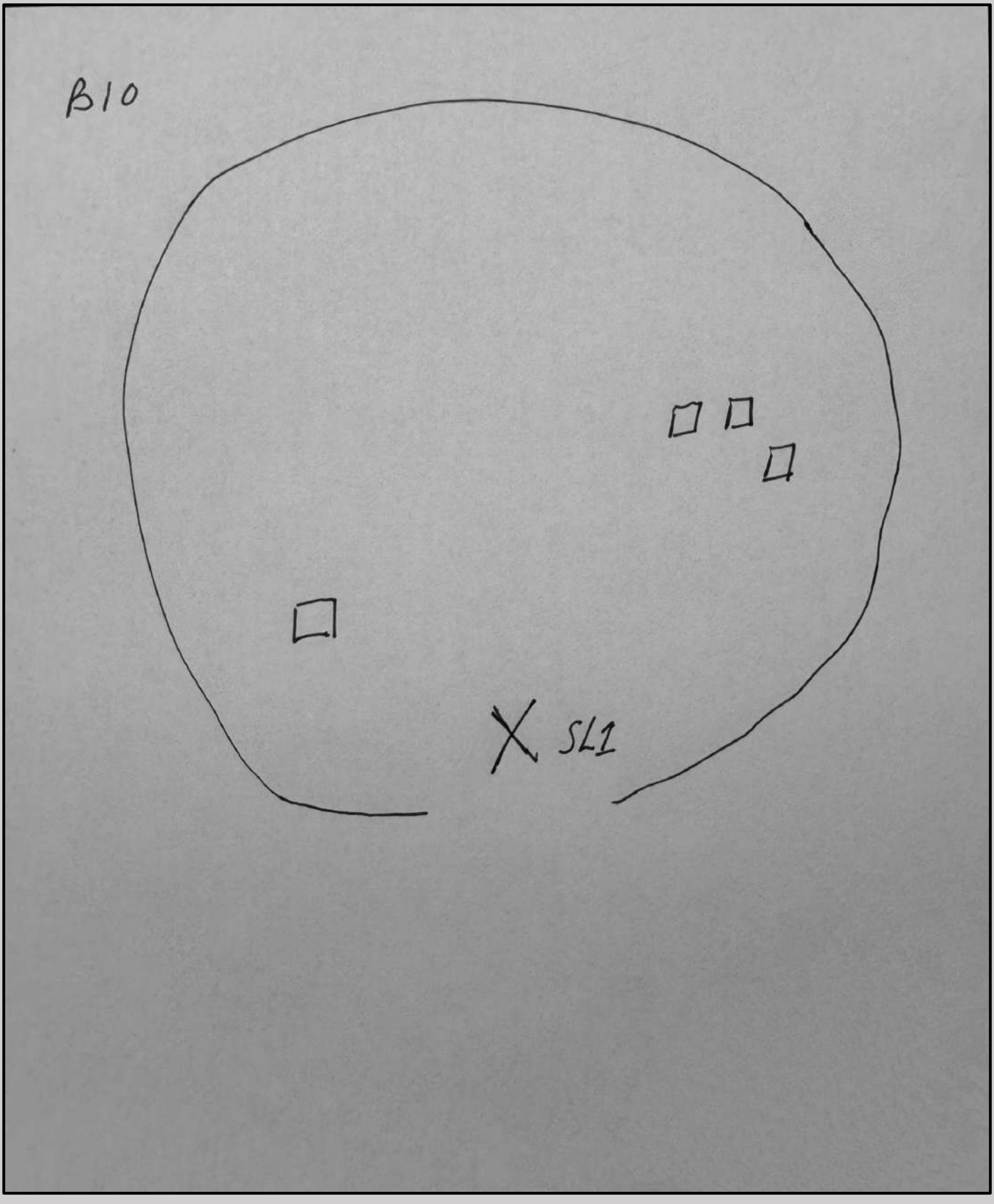

- B10

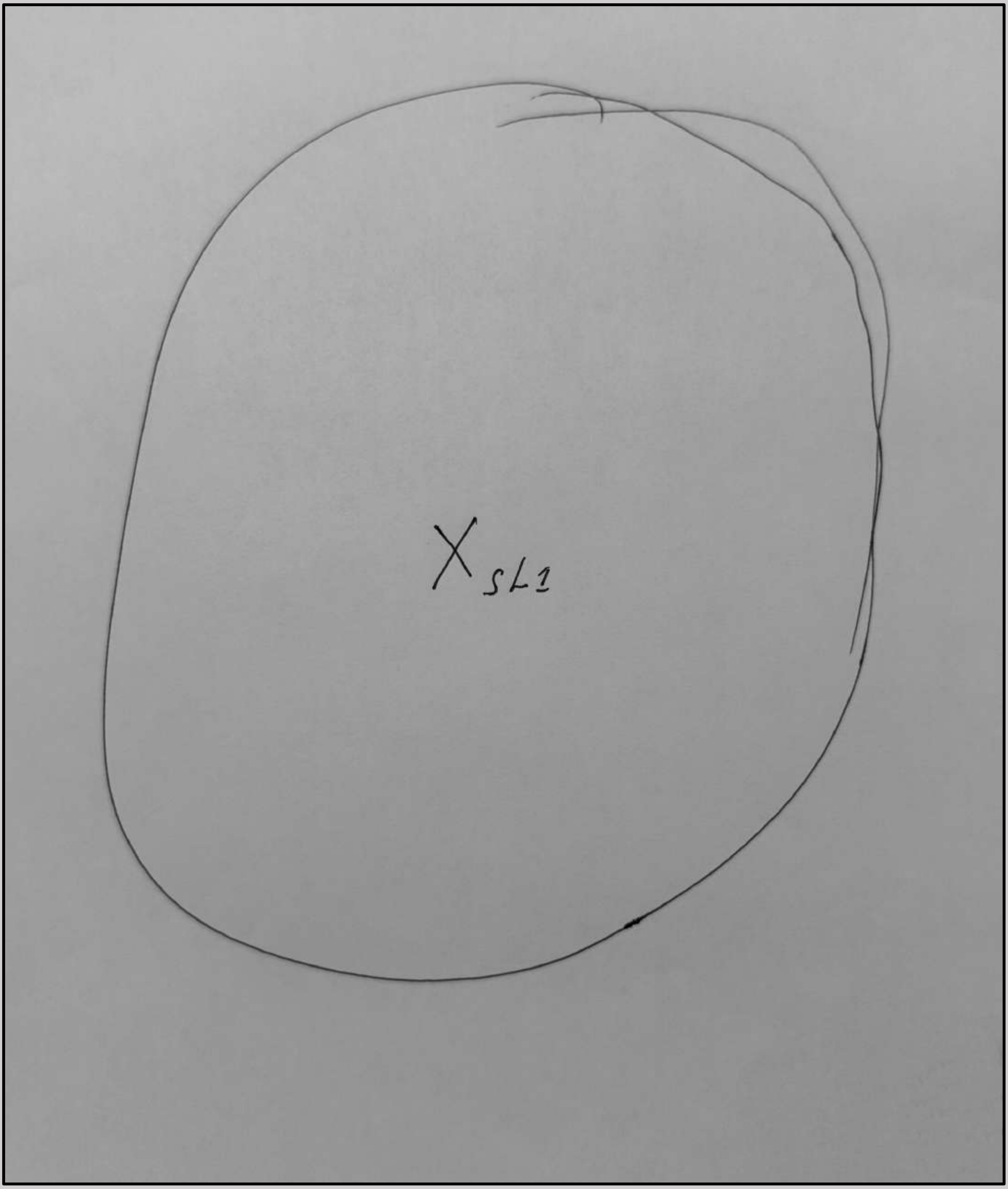

- C5

Subject 18

Sequence: part/space

3 NDE

- A8
- B6
- C5

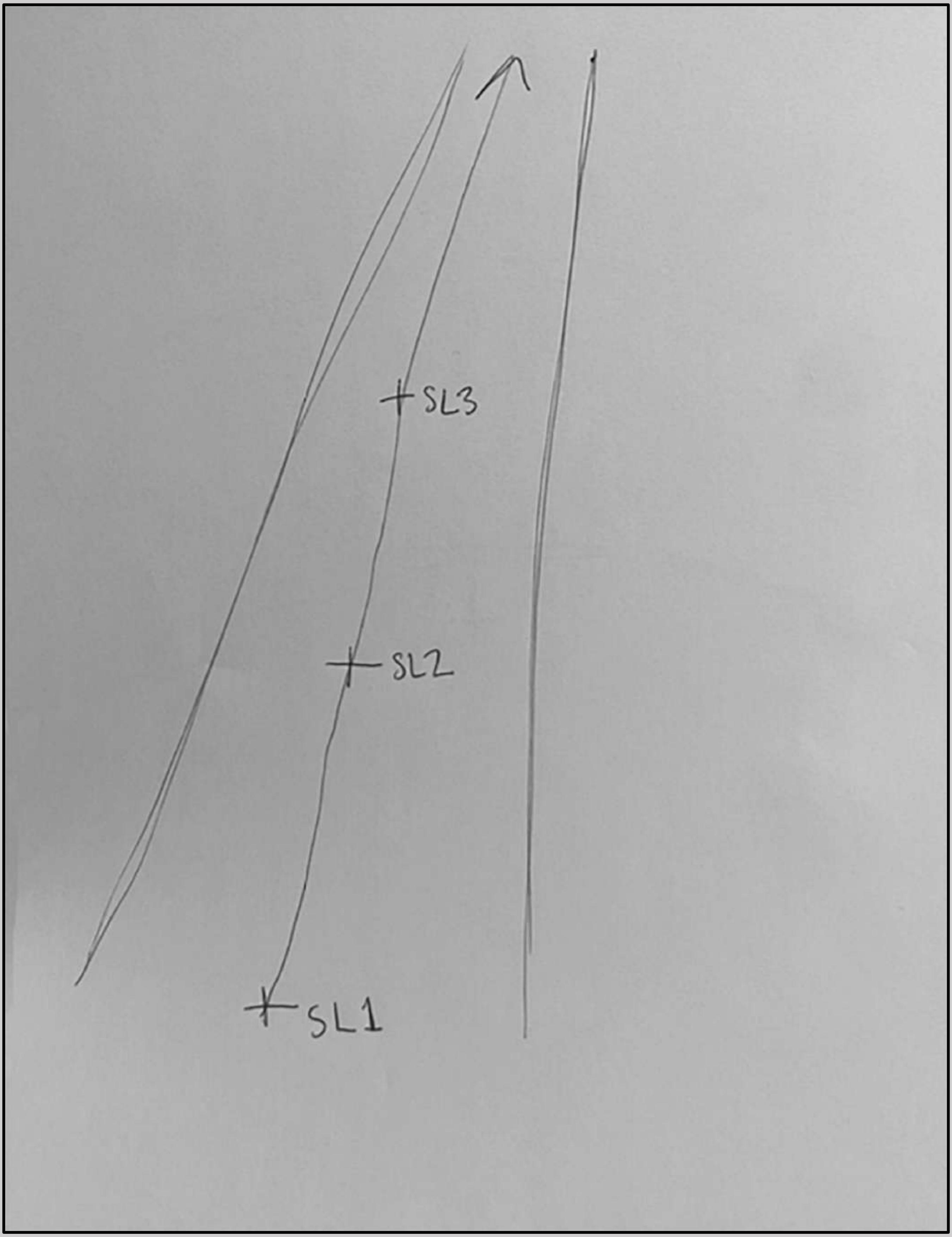

- A8

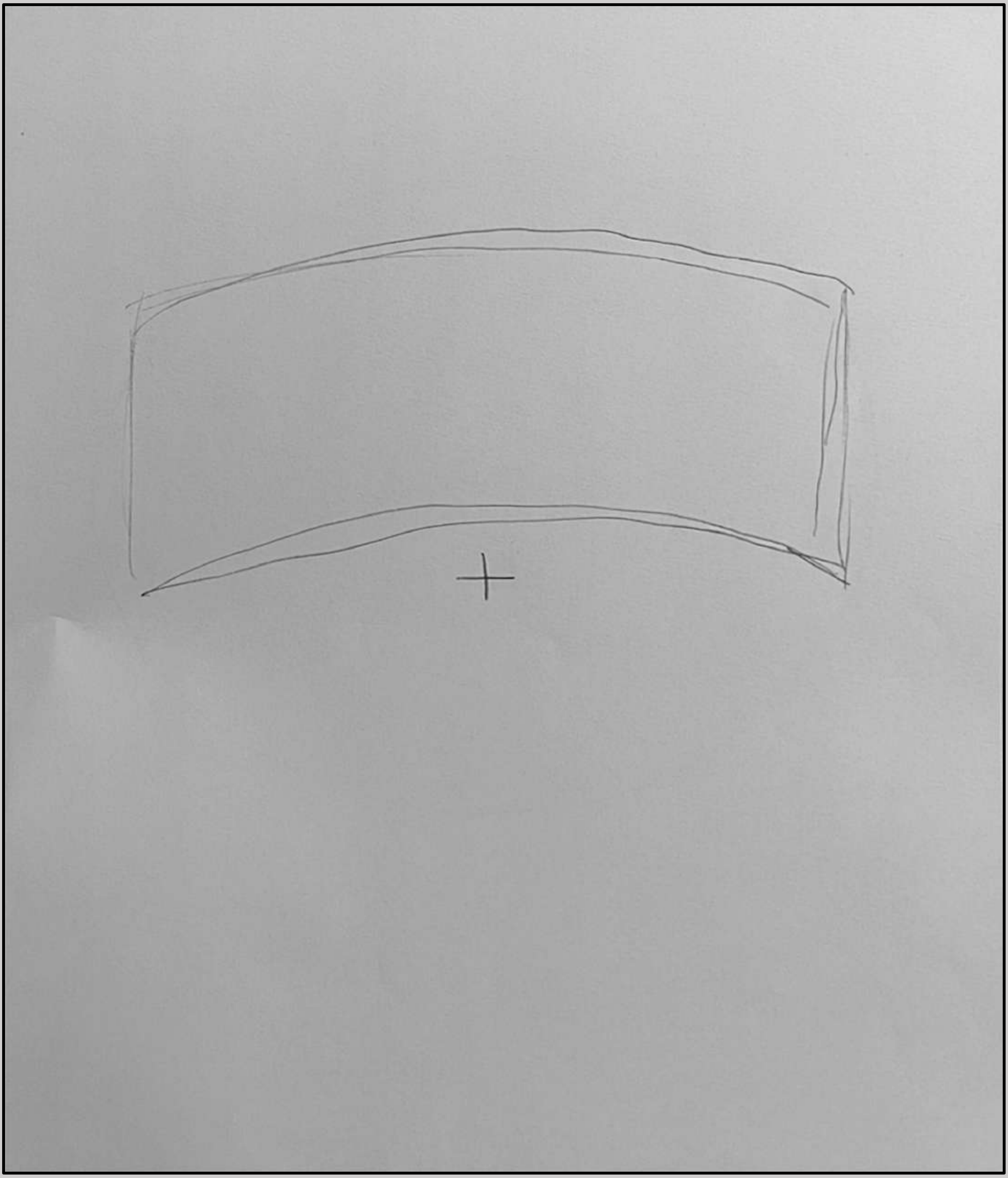

- B6

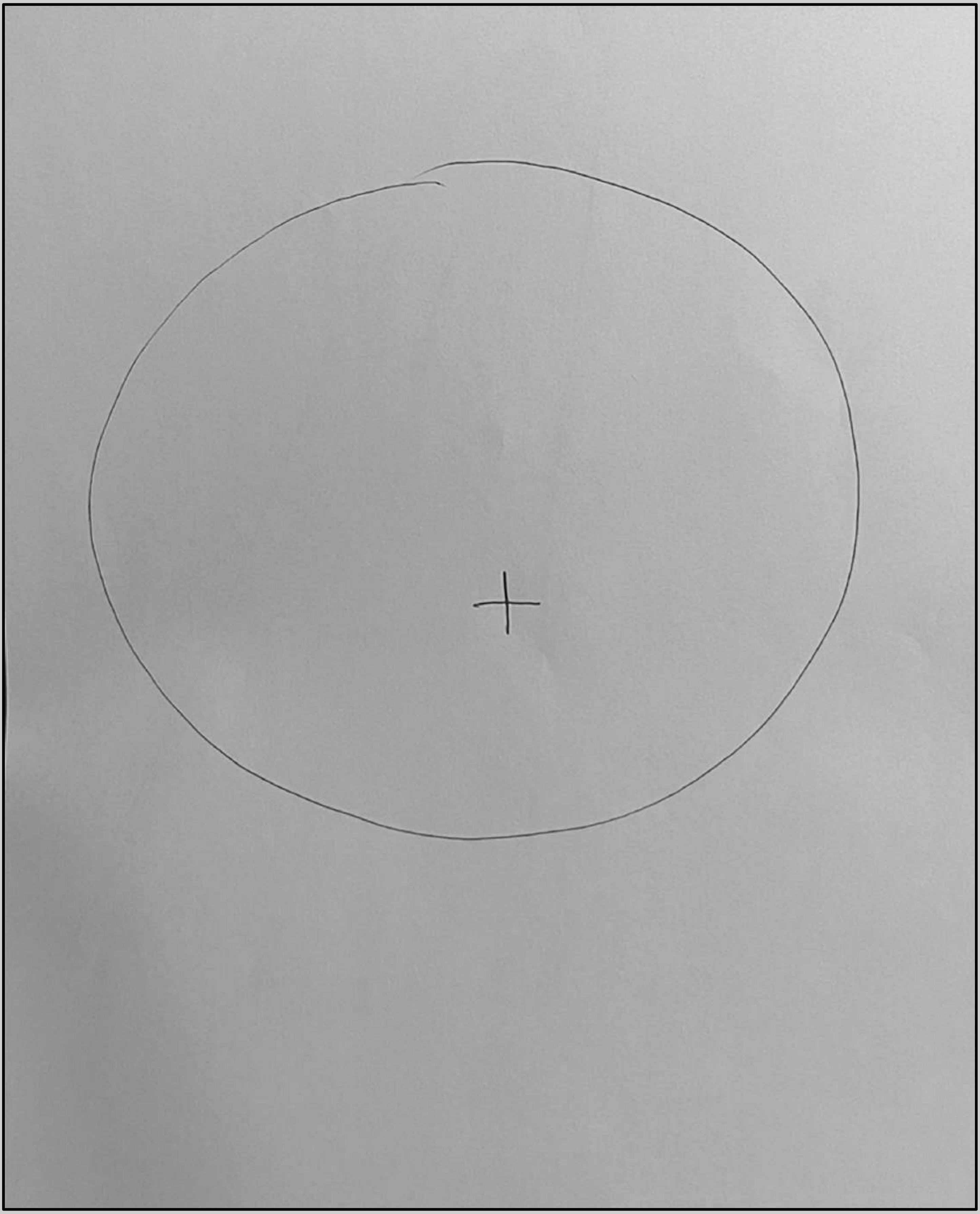

- C5

Subject 19

Sequence: part/space

1 OBE + 1 NDE

- OBE Dissociative
- A8

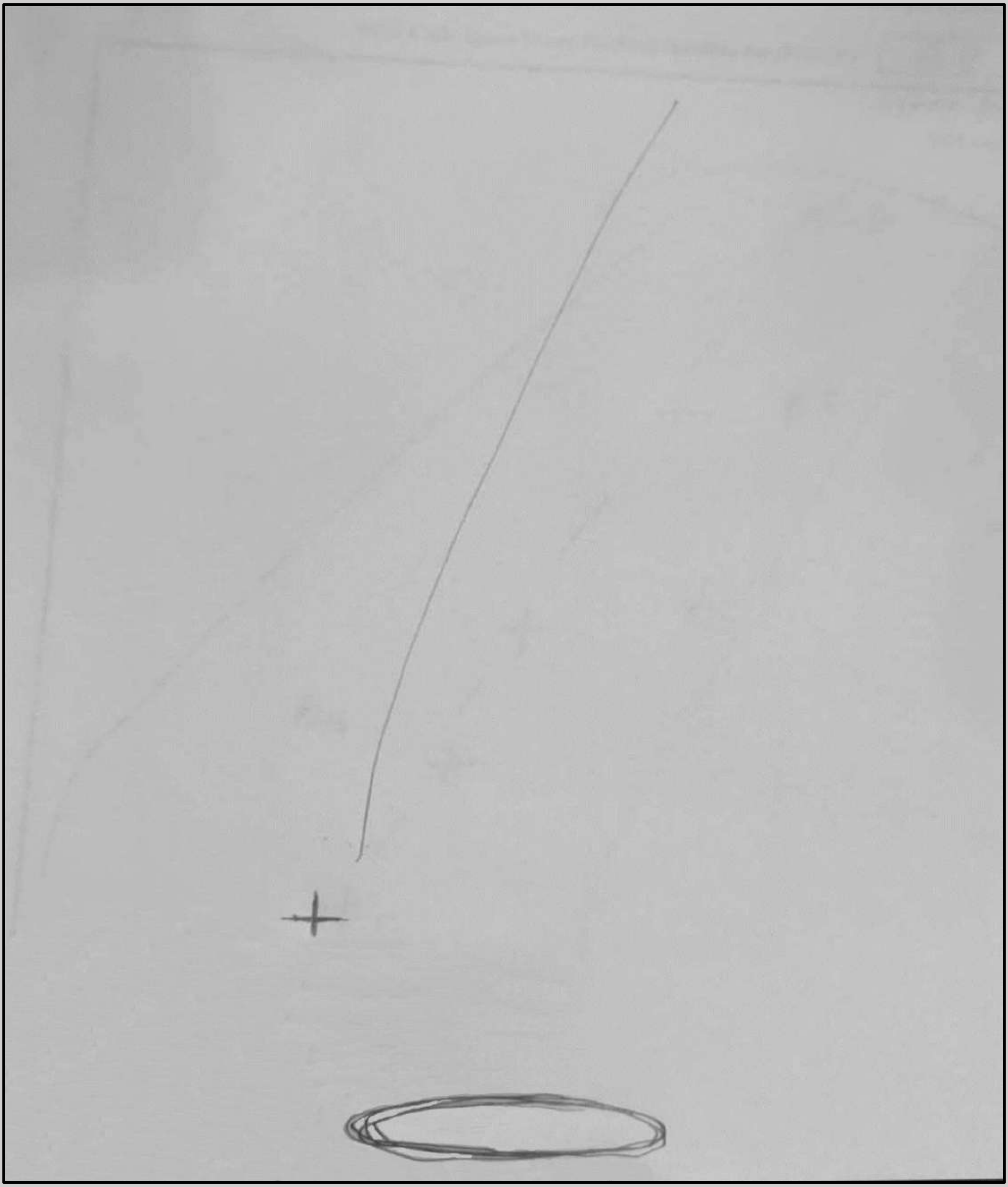

- OBE Dissociative

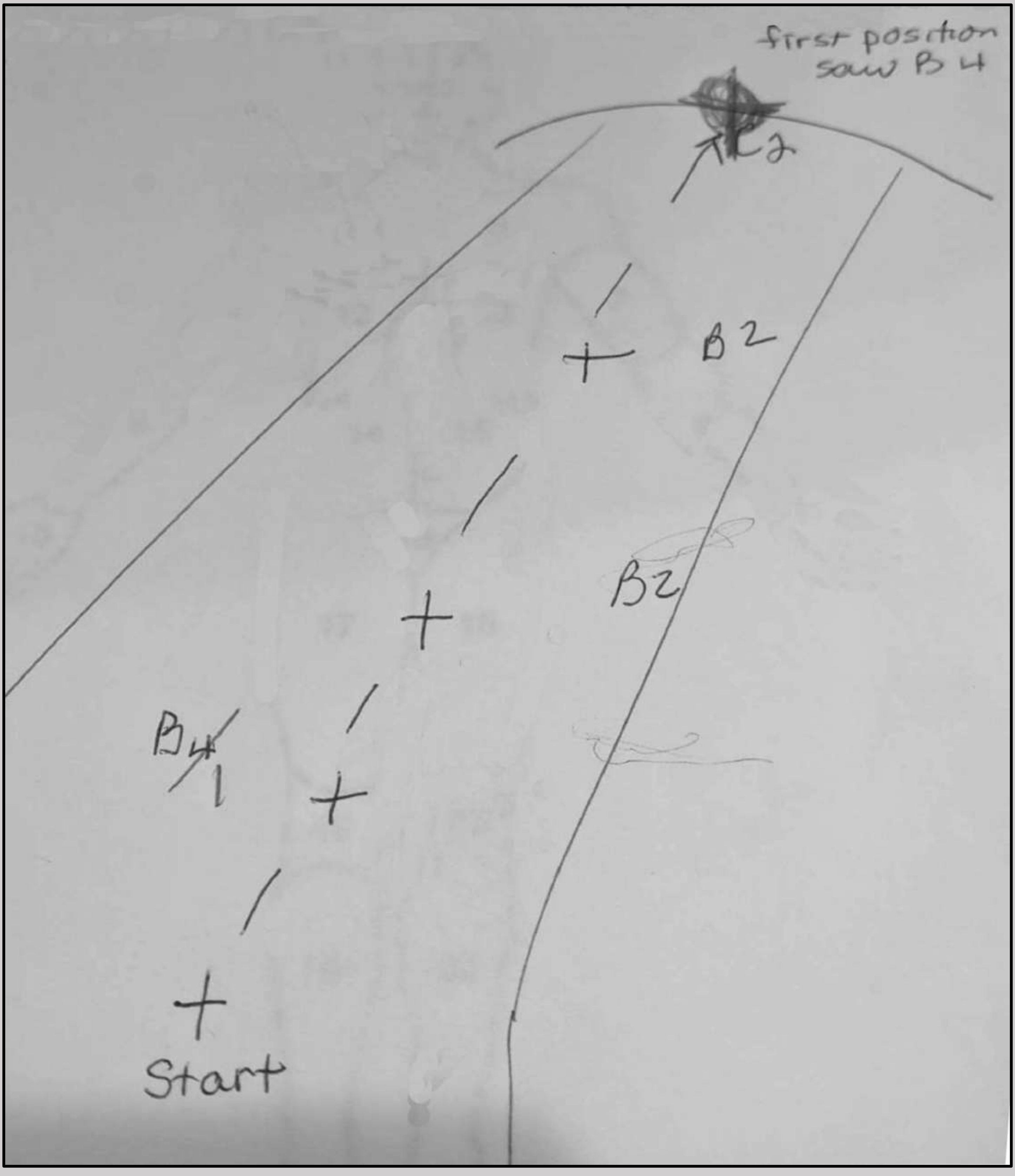

- A8

Subject 20

Sequence: part/space

1 OBE + 4 NDE

- A9
- B6
- C2
- C5
- OBE Associative

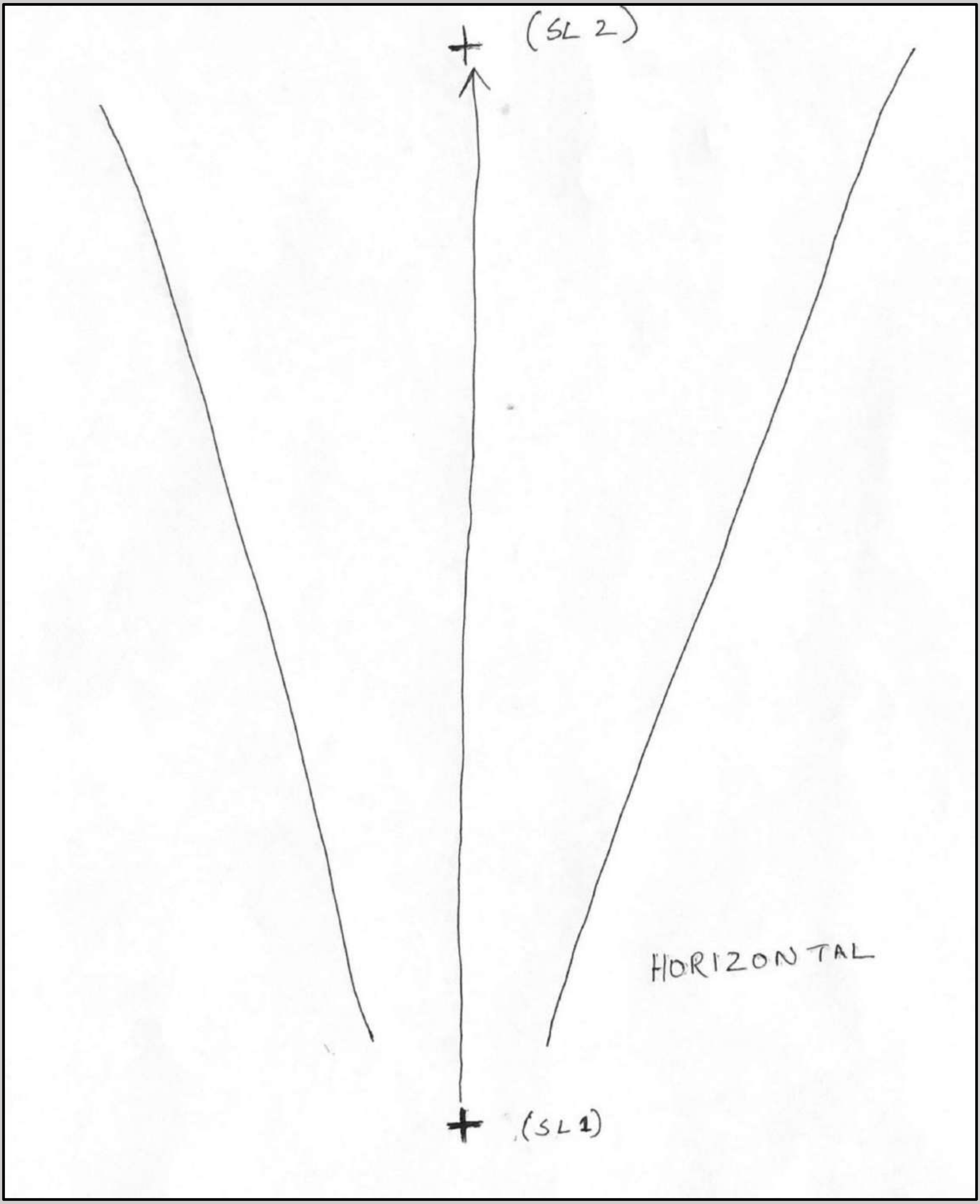

- A9

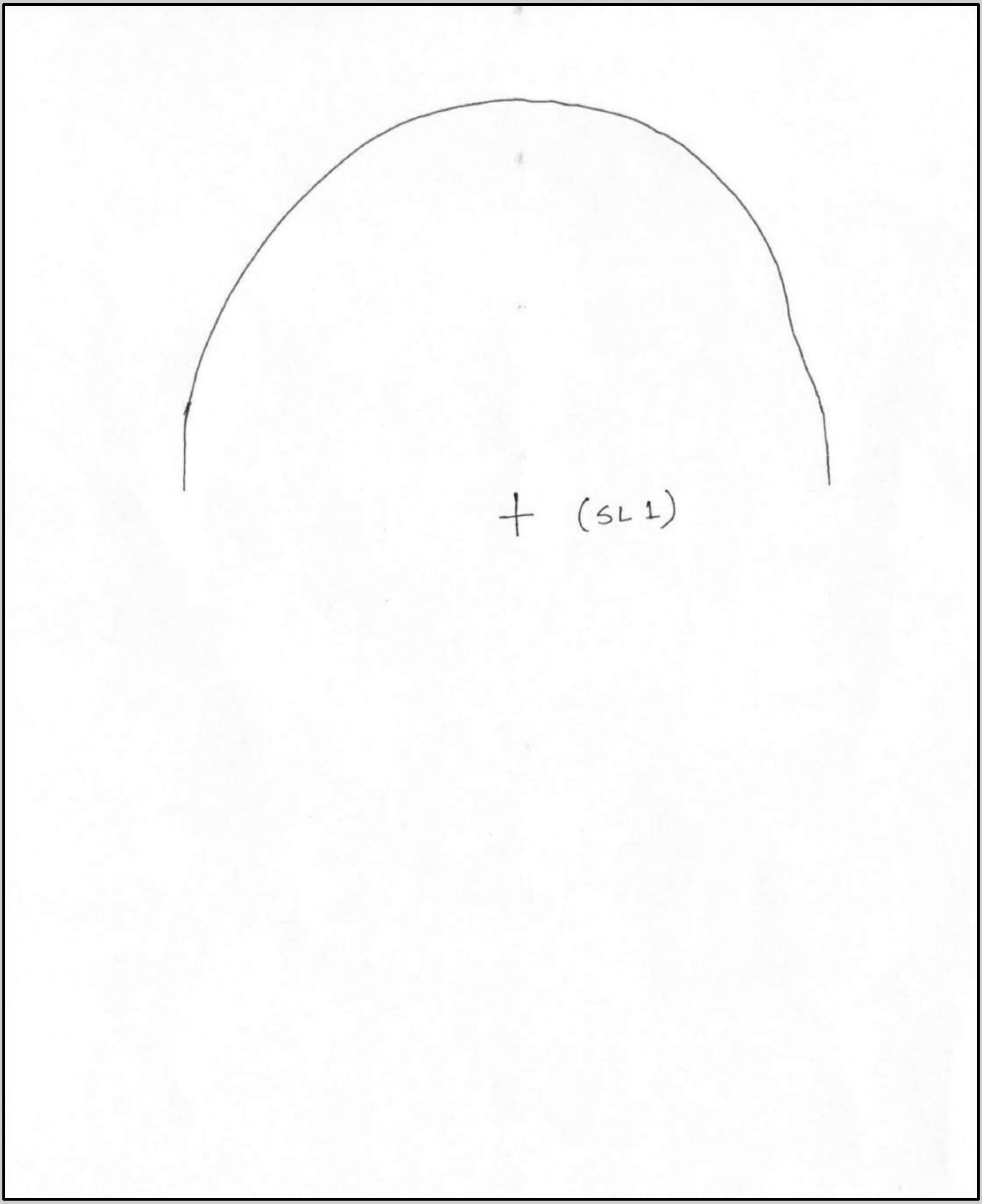

• B6

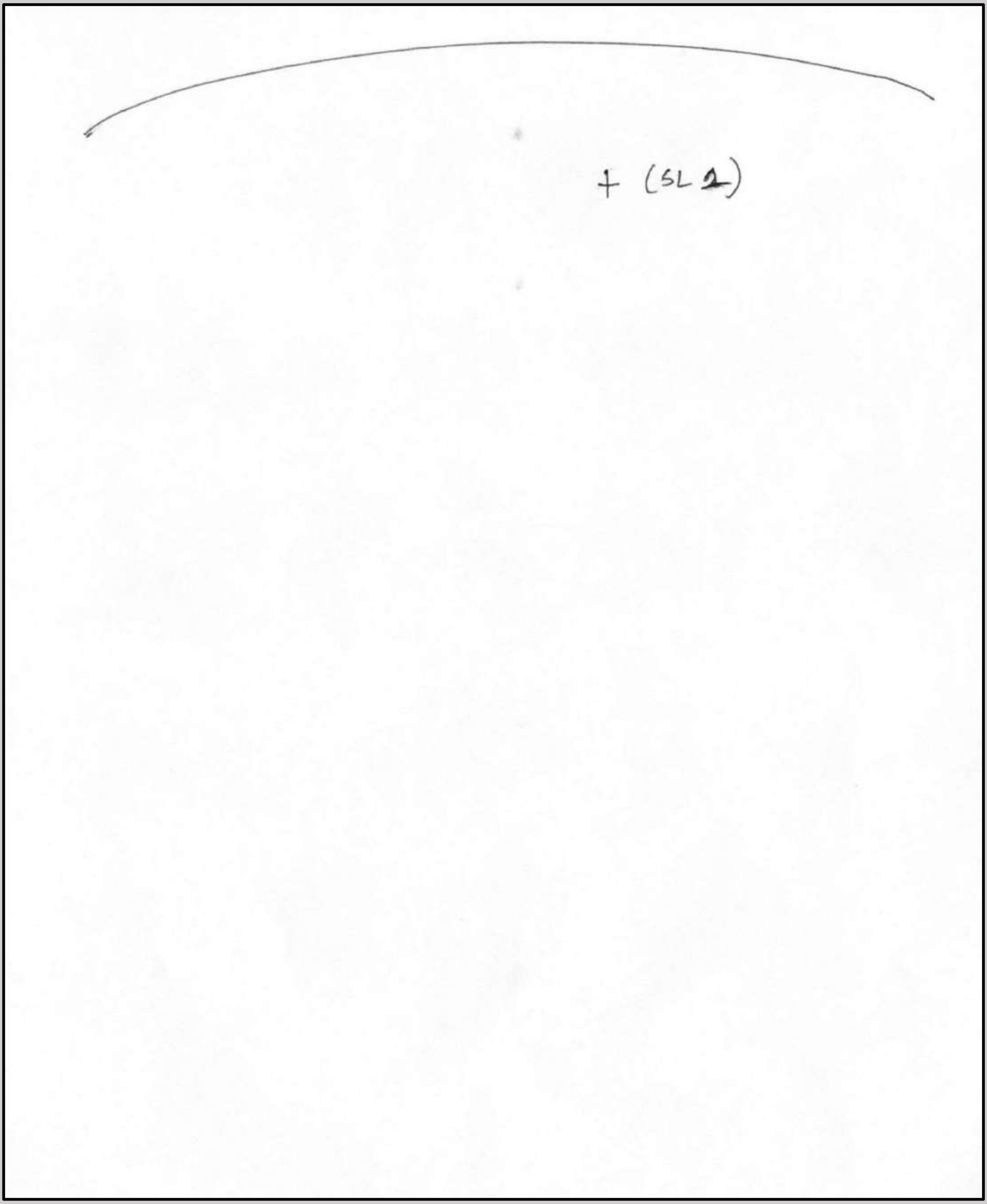

- C2

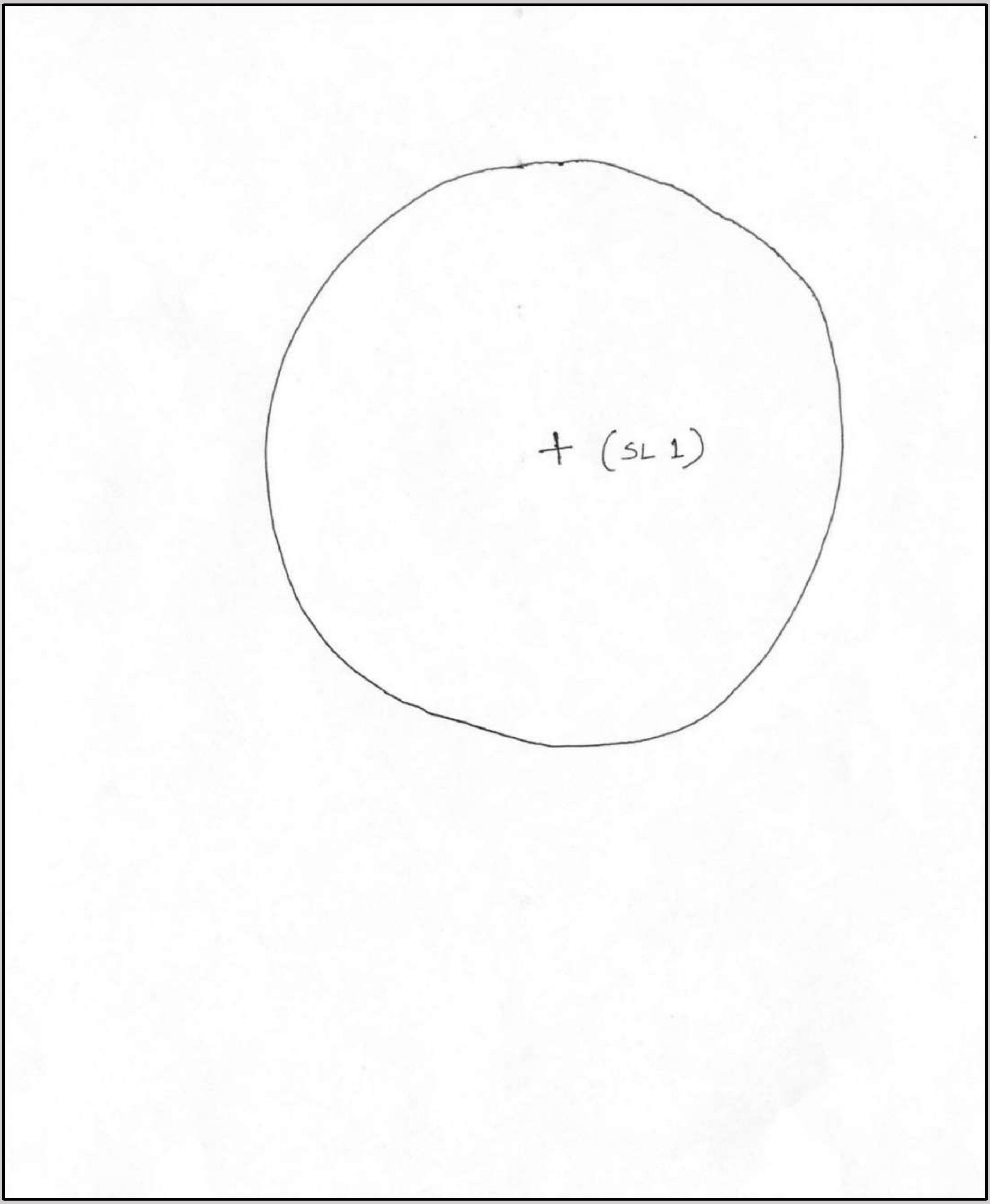

- C5

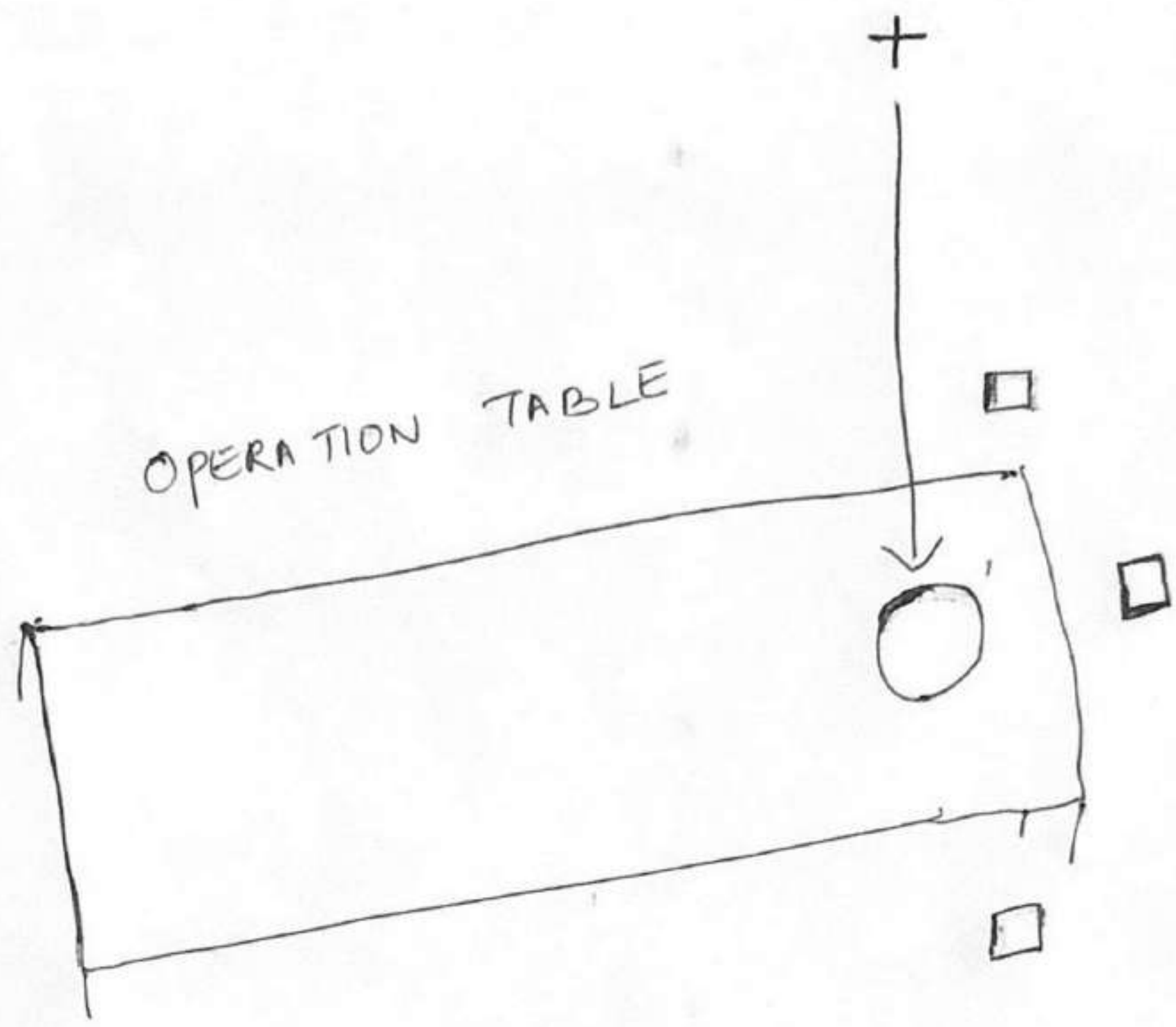

- OBE Associative

Subject 21

Sequence: part/space

1 OBE + 6 NDE

- A9
- C5
- B2
- C3
- C1
- C5
- OBE Associative

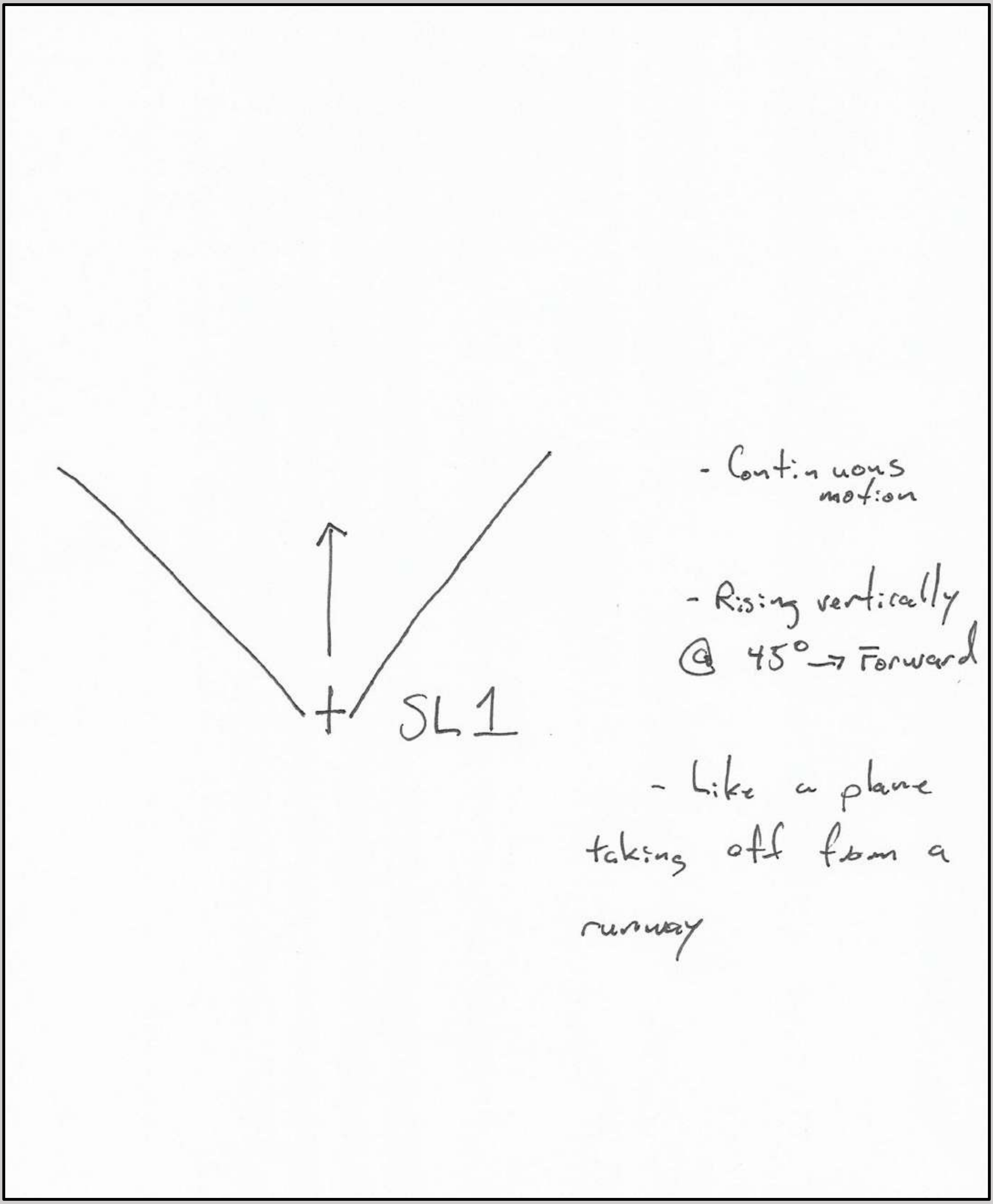
SL1
- Continuous motion
- Rising vertically @ 45° → Forward
- Like a plane taking off from a runway

- A9

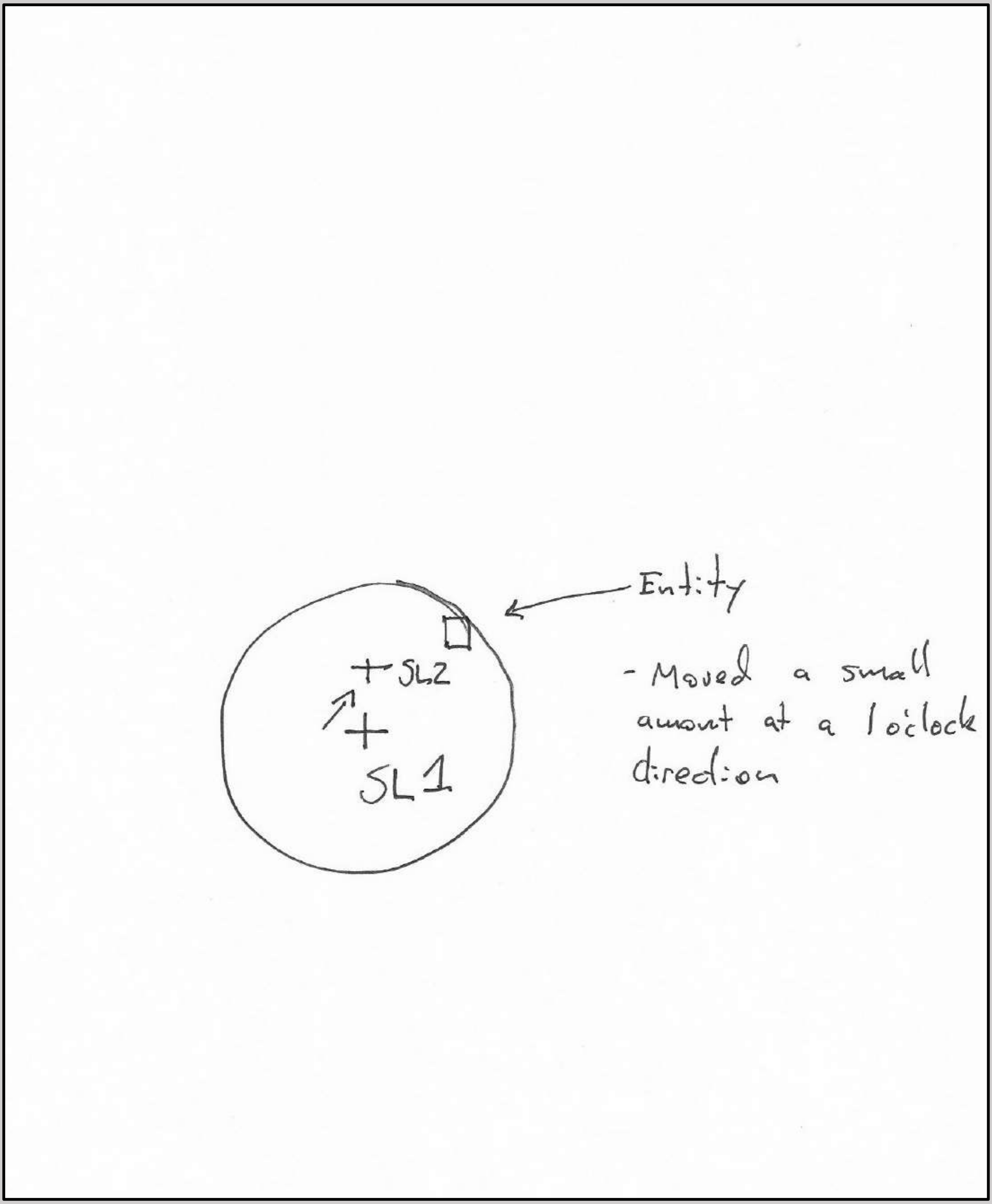

• C5

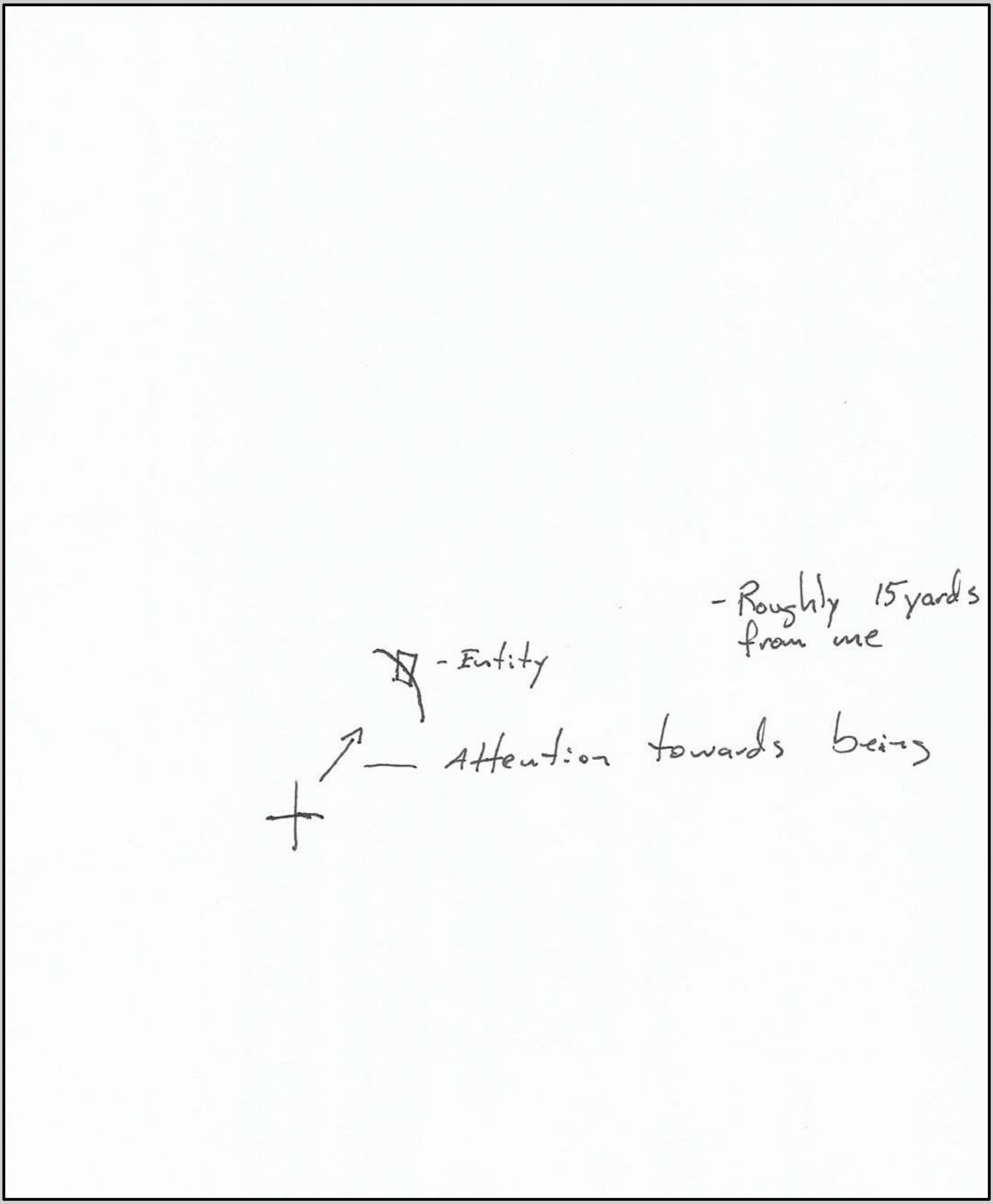

- B2

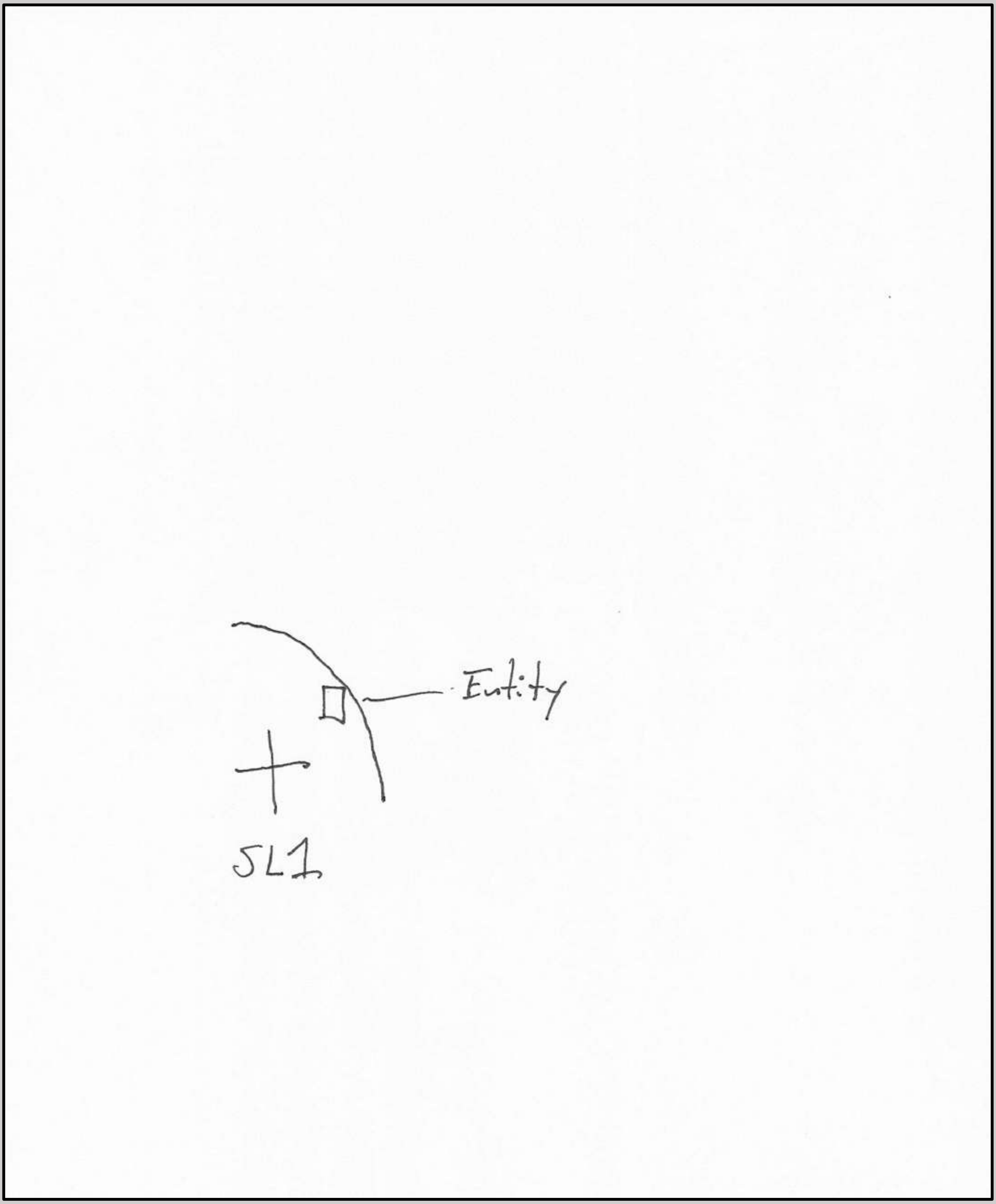

- C3

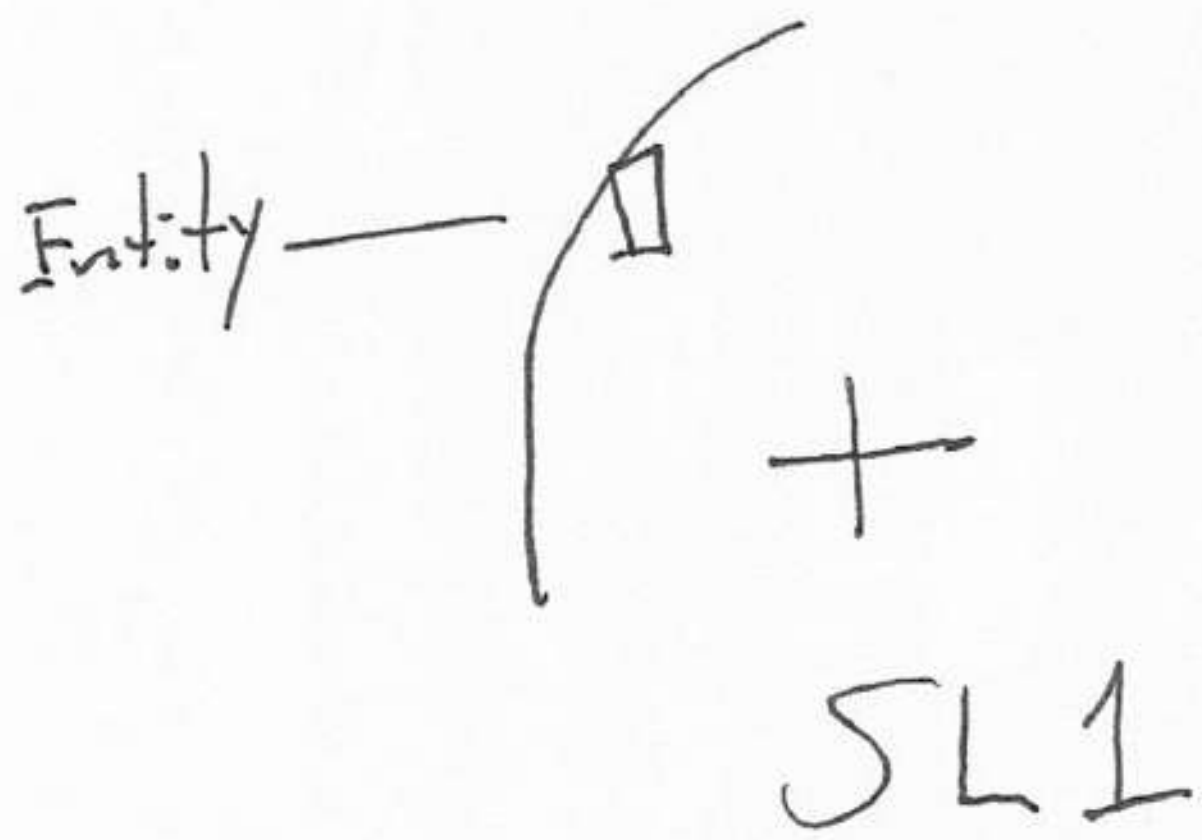

- C1

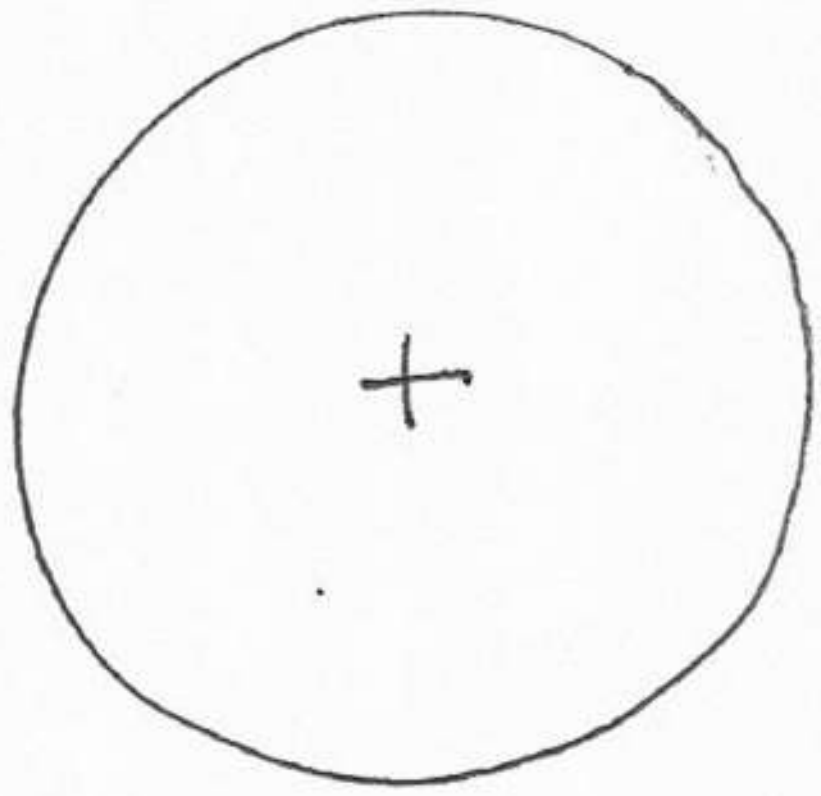

- C5

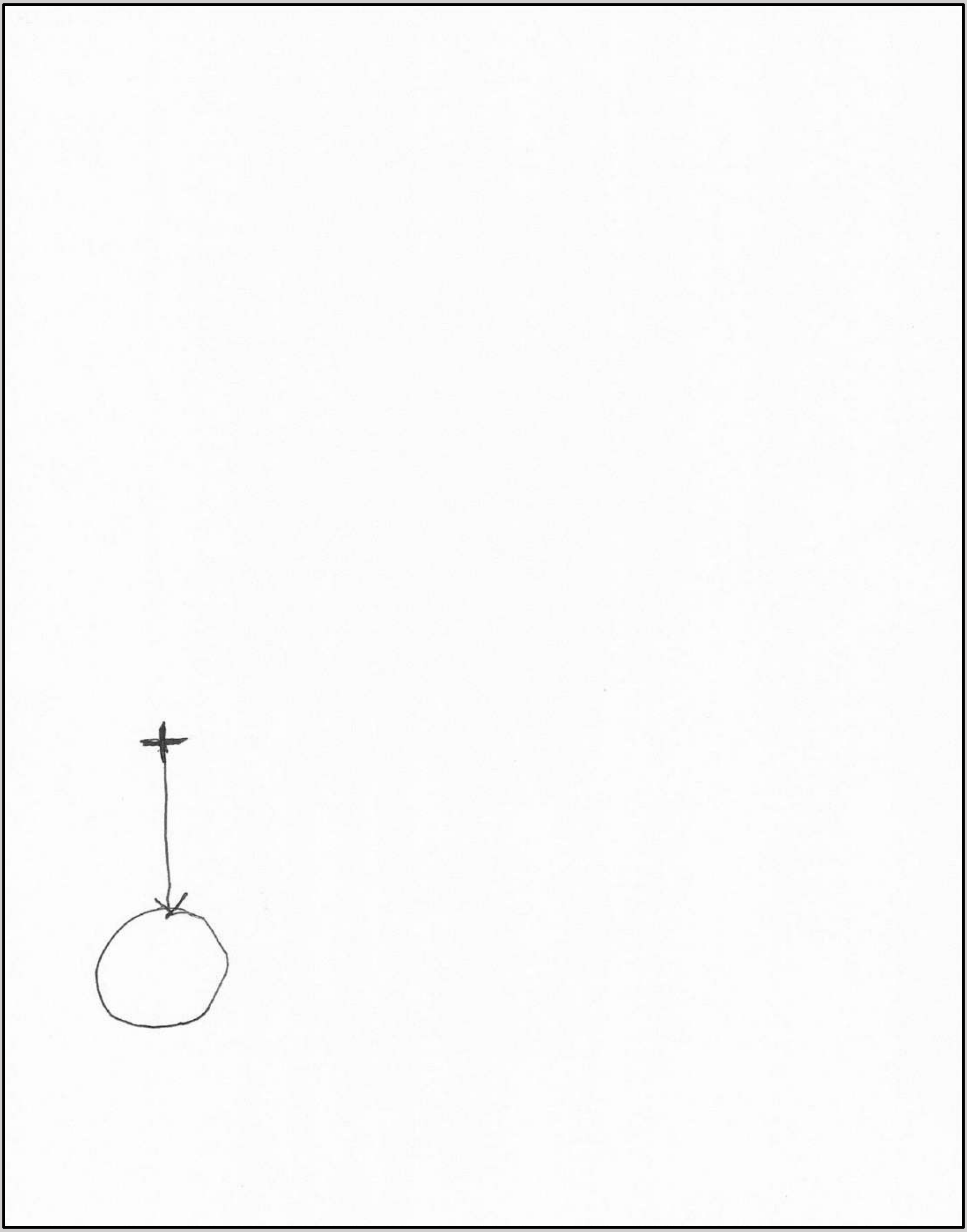

- OBE Associative

Subject 22

Sequence: part/space

2 NDE

- A9
- C5

Calmness^{10}/Well being^{10} -(inself)

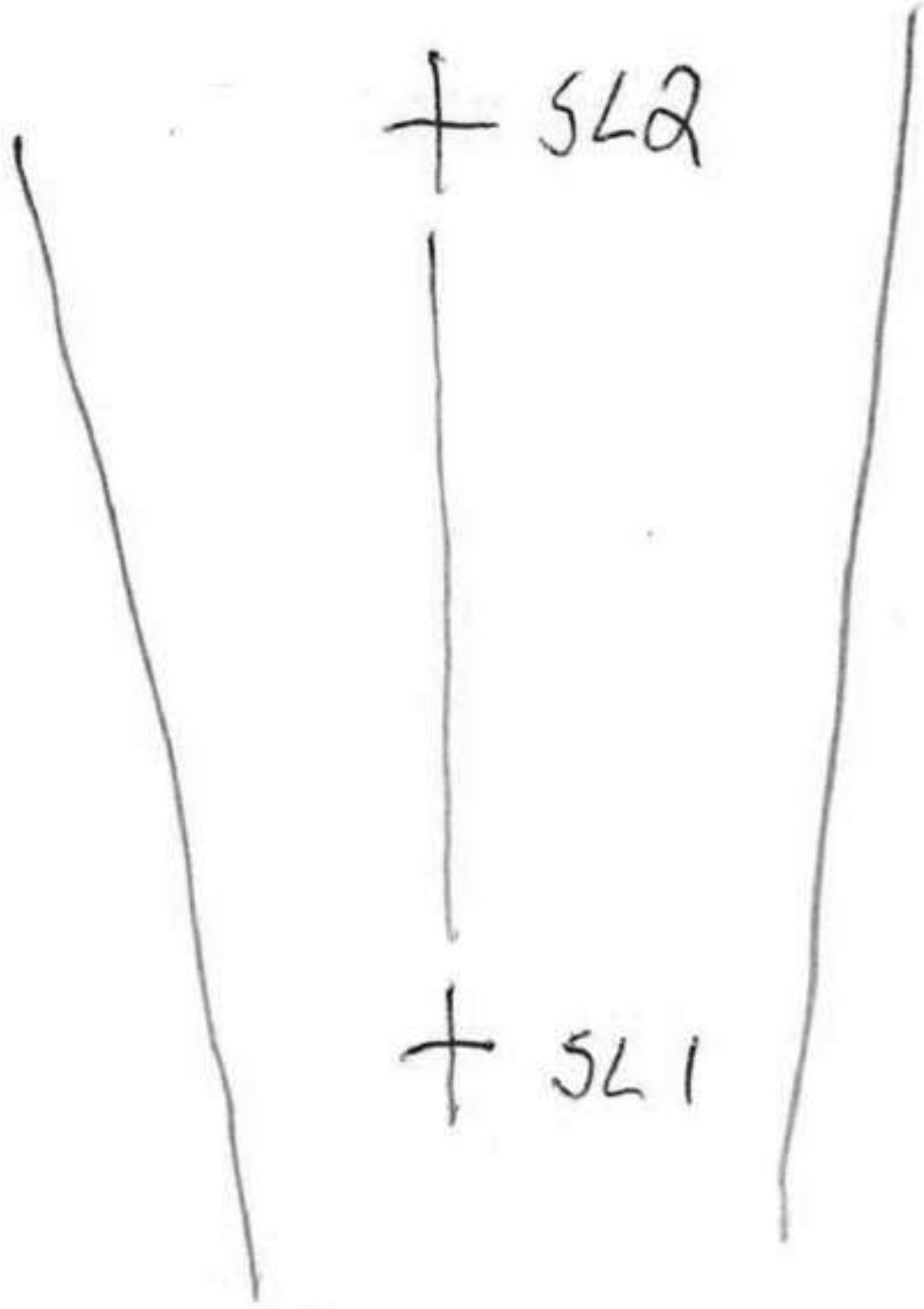

Moved through tunnel, started to lighten as I progressed, started black as night finished as gray early dawn

- A9

Calmness, 10(Self) Like a babe in its mother's arms 10(Self)
Well being 10(Self)
Love 10(Self)
Secure 10(Self)

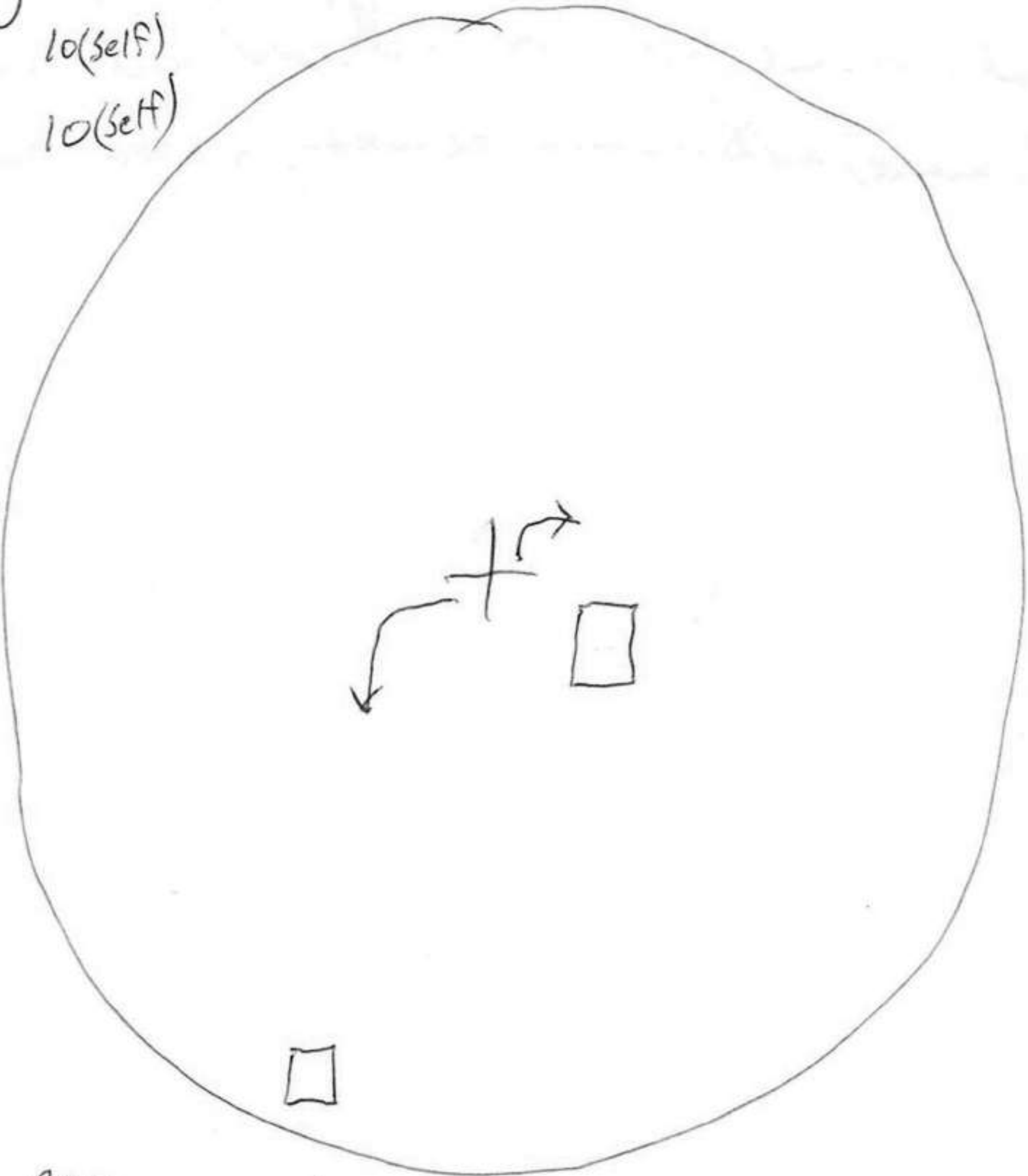

became light, Jesus at my right, bearded robed, there to show me the way out, at that time heard/felt motion/noise behind me, asked what it was, told that I was still loved and could return or move on

saw the sounds and colors

over

2 of 2

a guide appeared at my right shoulder, stated he was there to lead me to next experience (guide was Jesus/love), communicated without words, after seeing lights and sounds associated with my father I asked what they signified, informed that I was still loved and could return if I desired, next experience was awaking from coma

• C5

Subject 23

Sequence: part/space

3 NDE

- A9
- C5
- A8

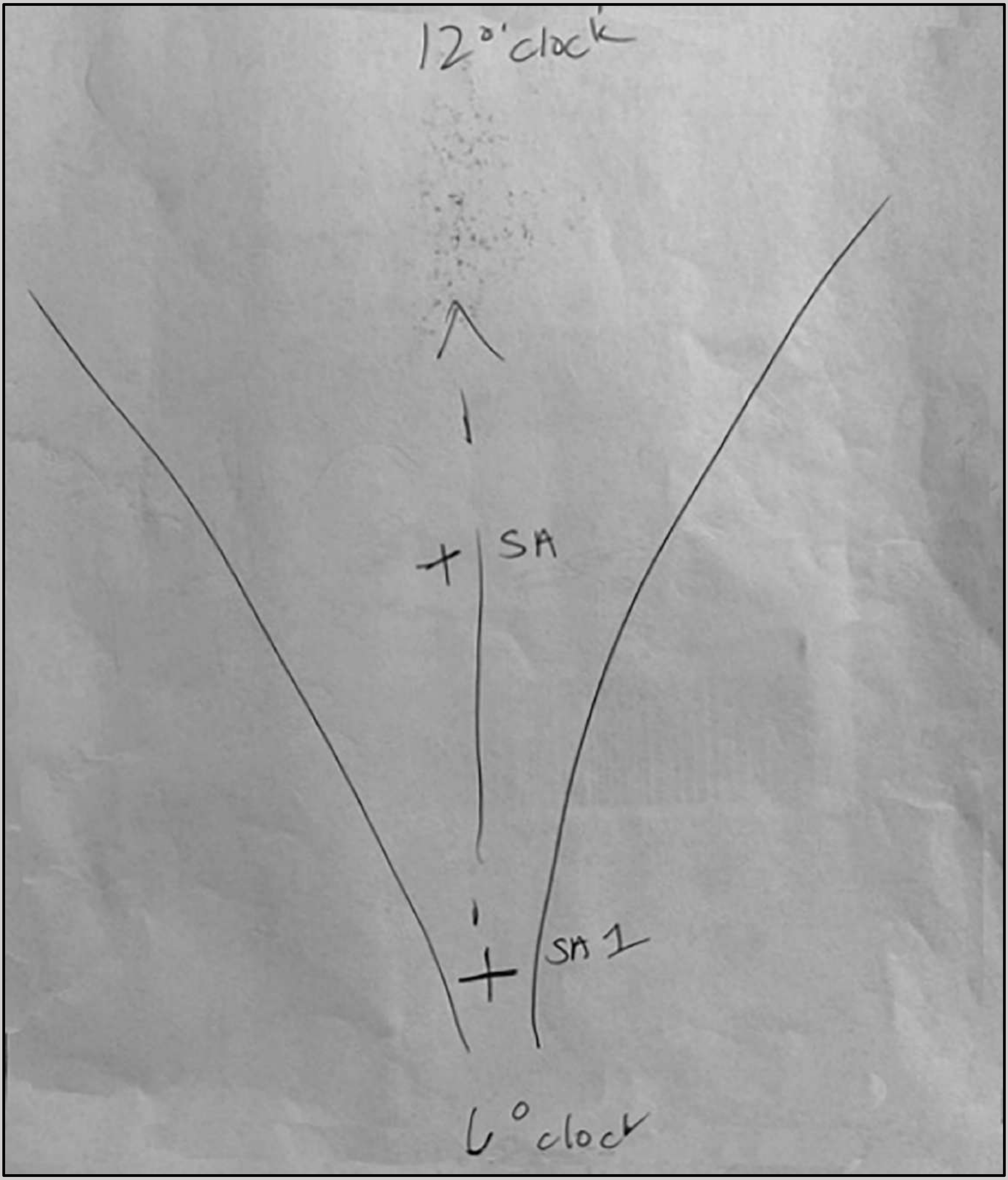

• A9

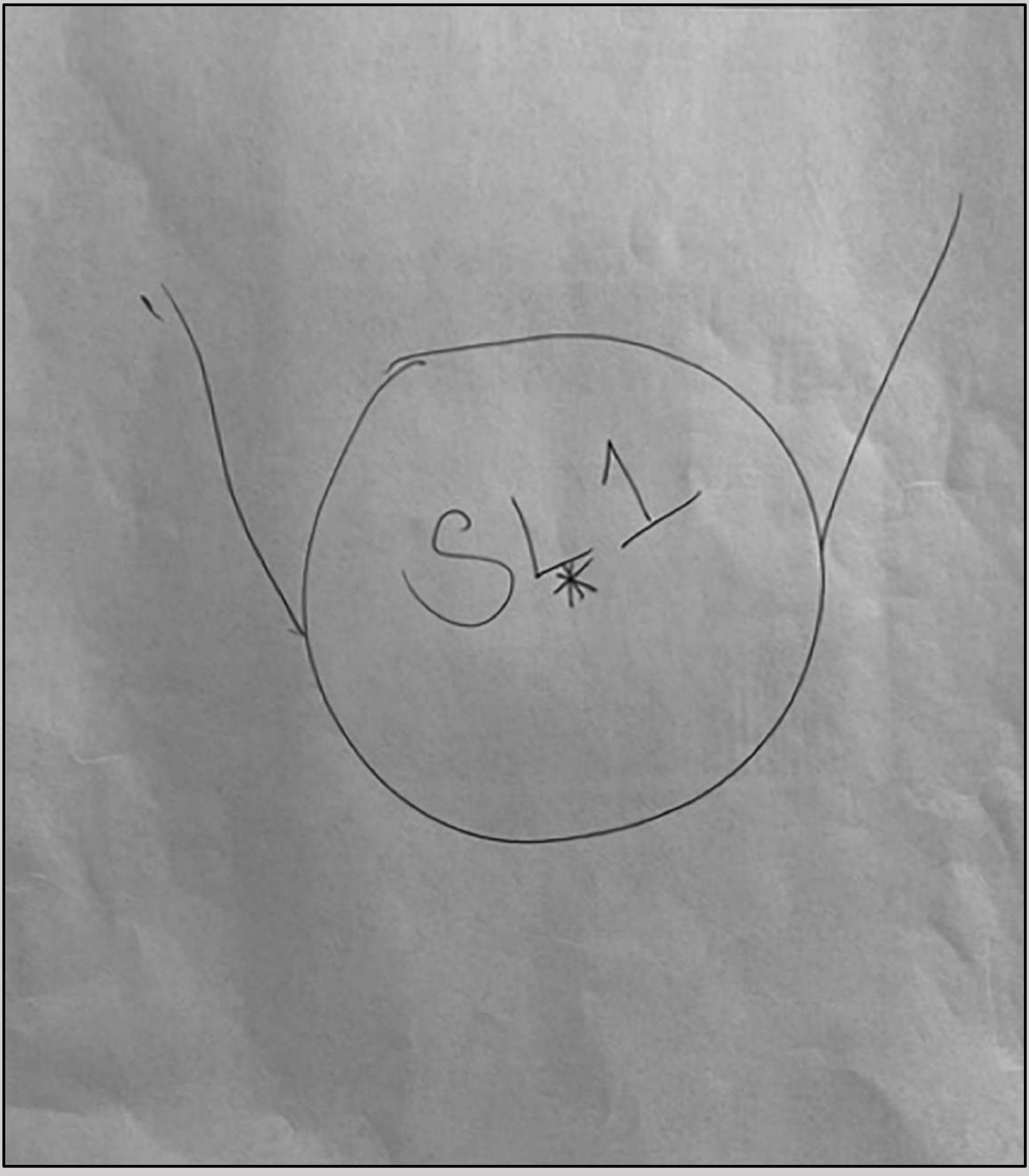

- C5

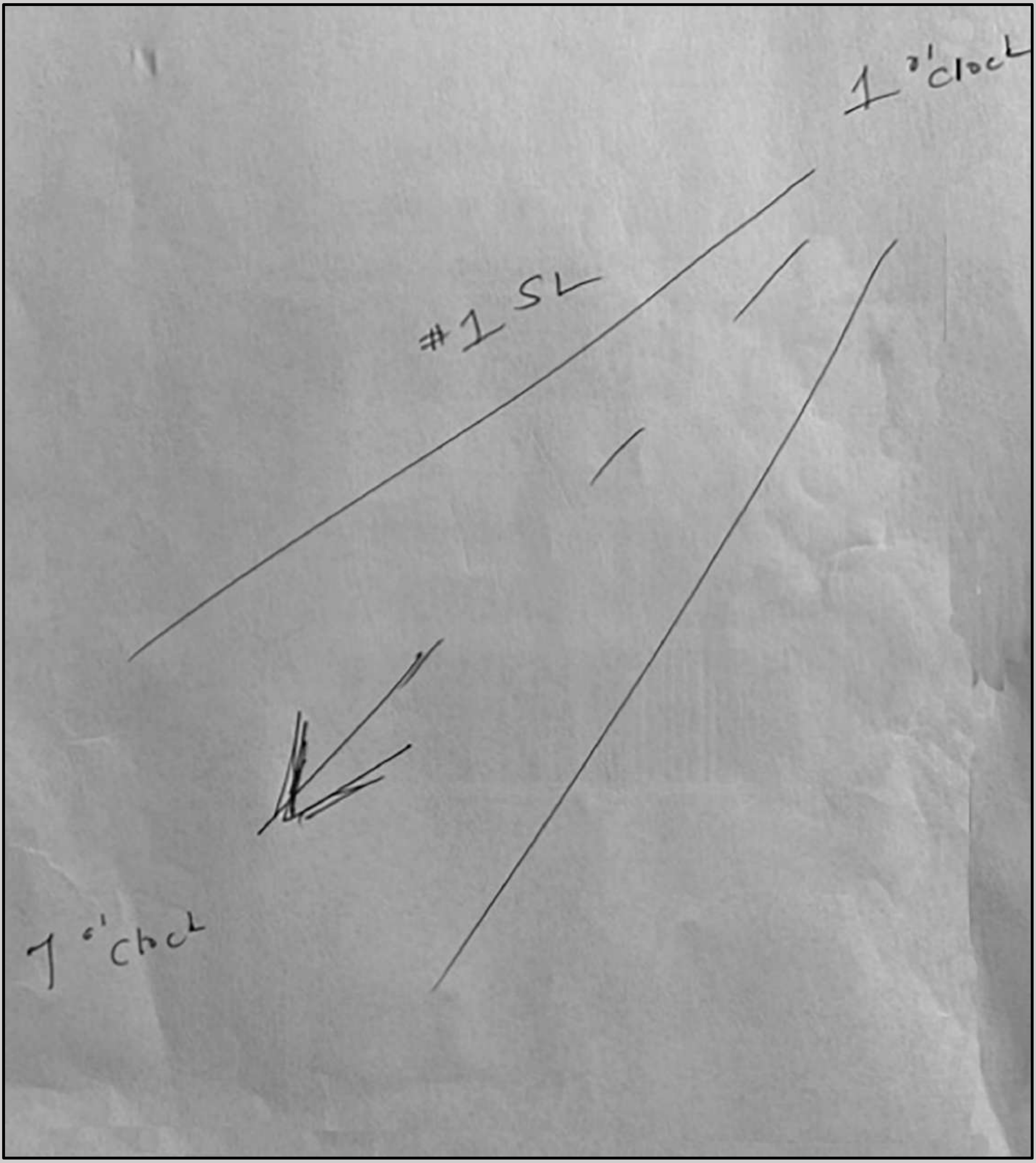

- A8

Subject 24

Sequence: part/space

2 OBE + 1 NDE

- OBE Dissociative
- B1
- OBE Associative

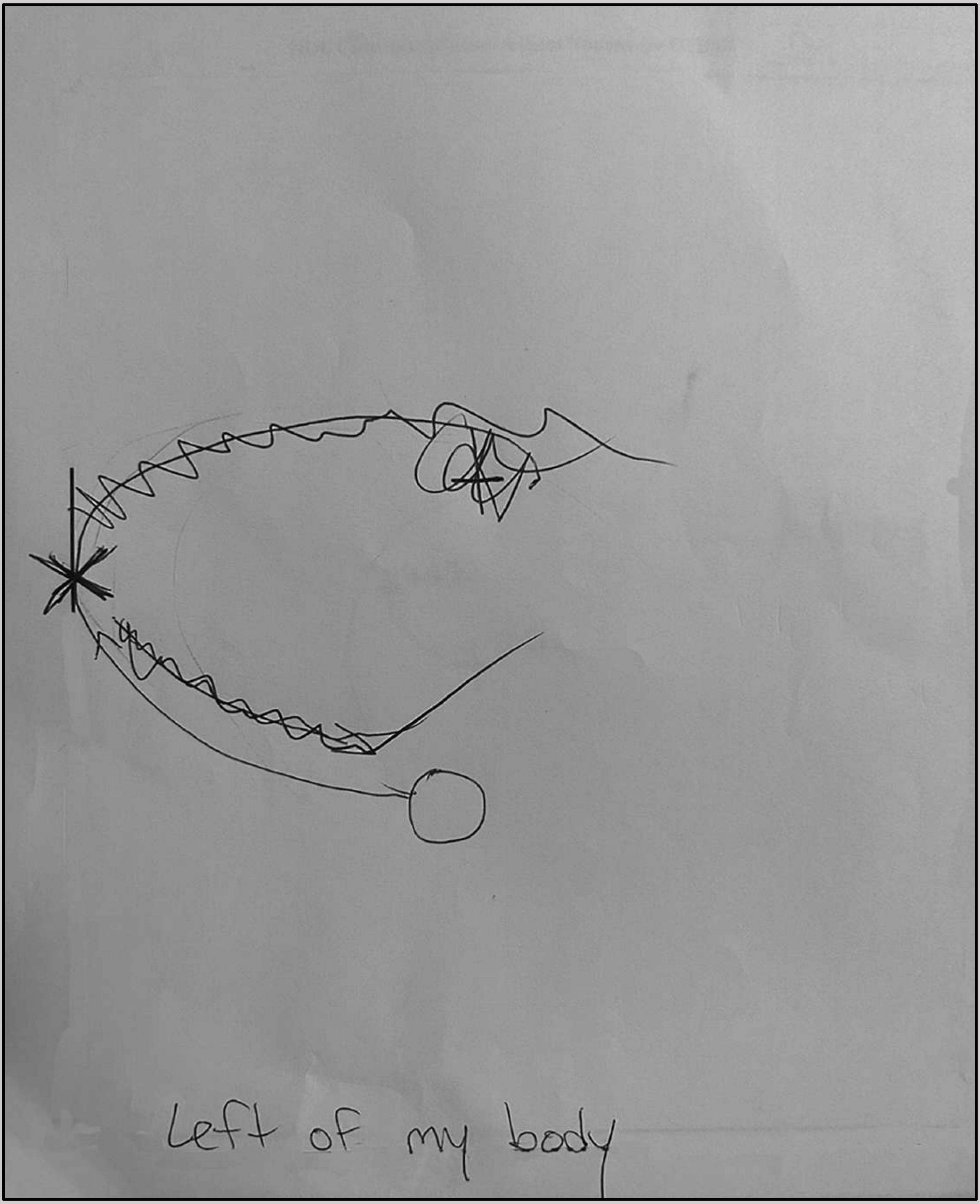

- OBE Dissociative

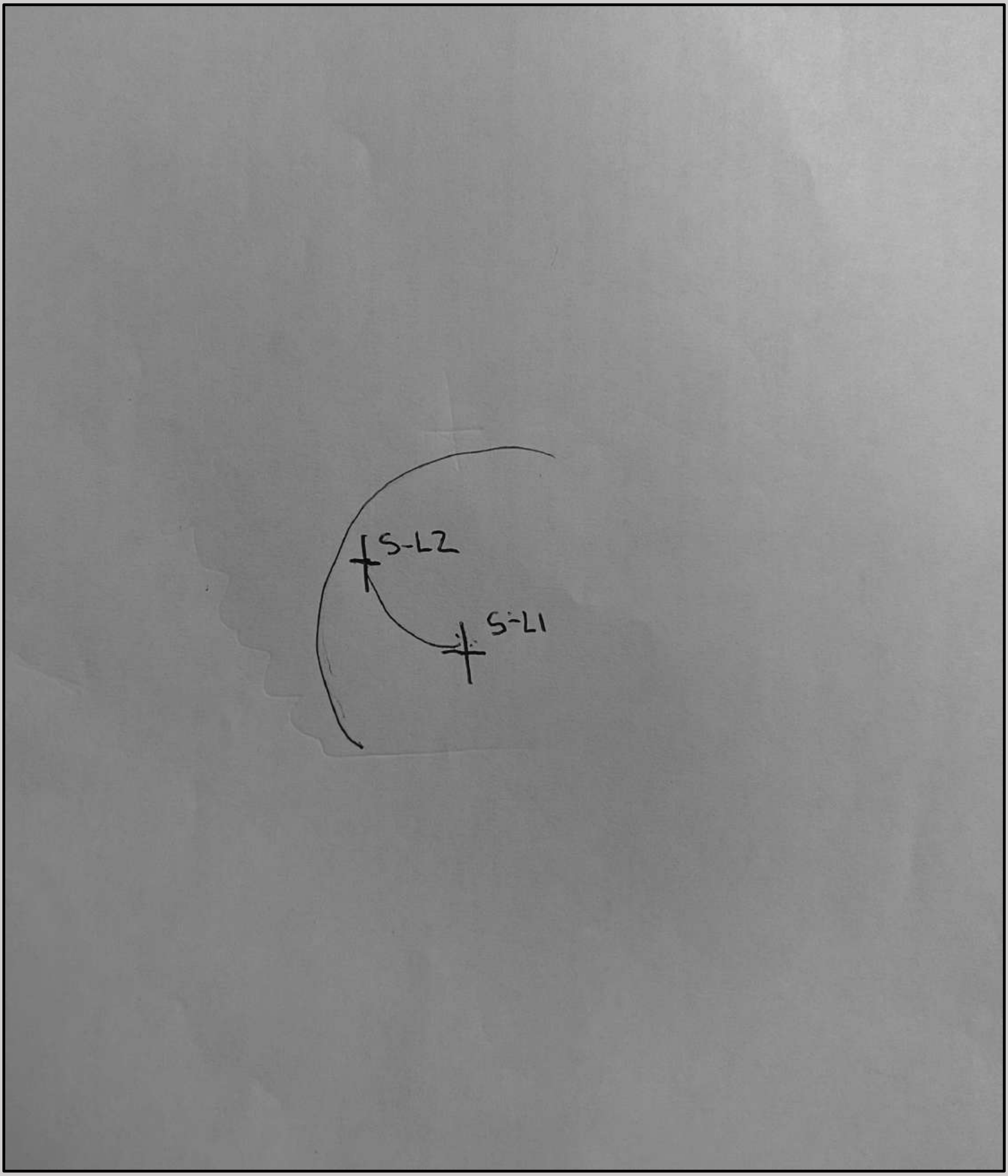

- B1

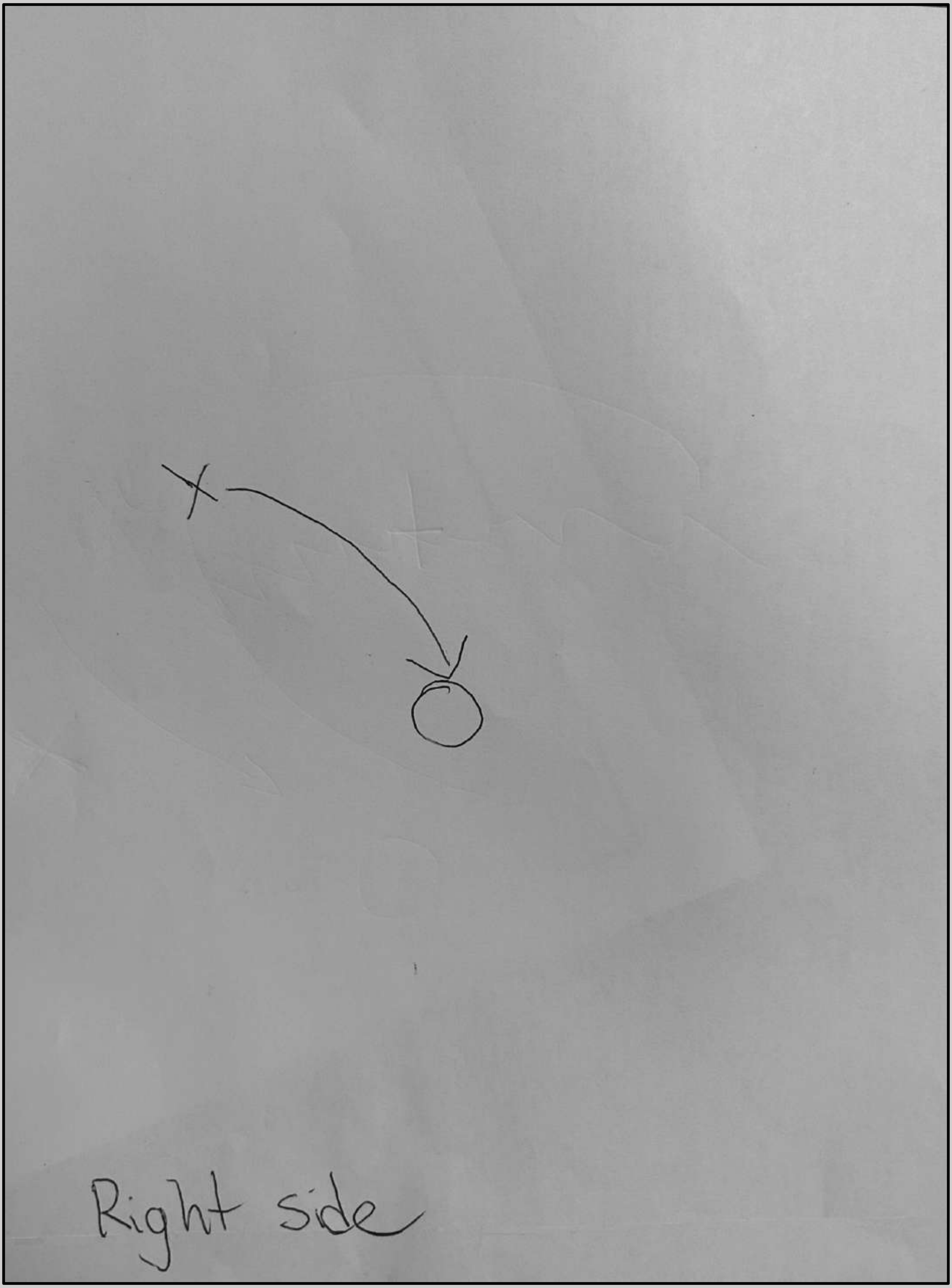

- OBE Associative

Subject 25

Sequence: part/space

4 NDE

- B12
- C5
- B10
- A9

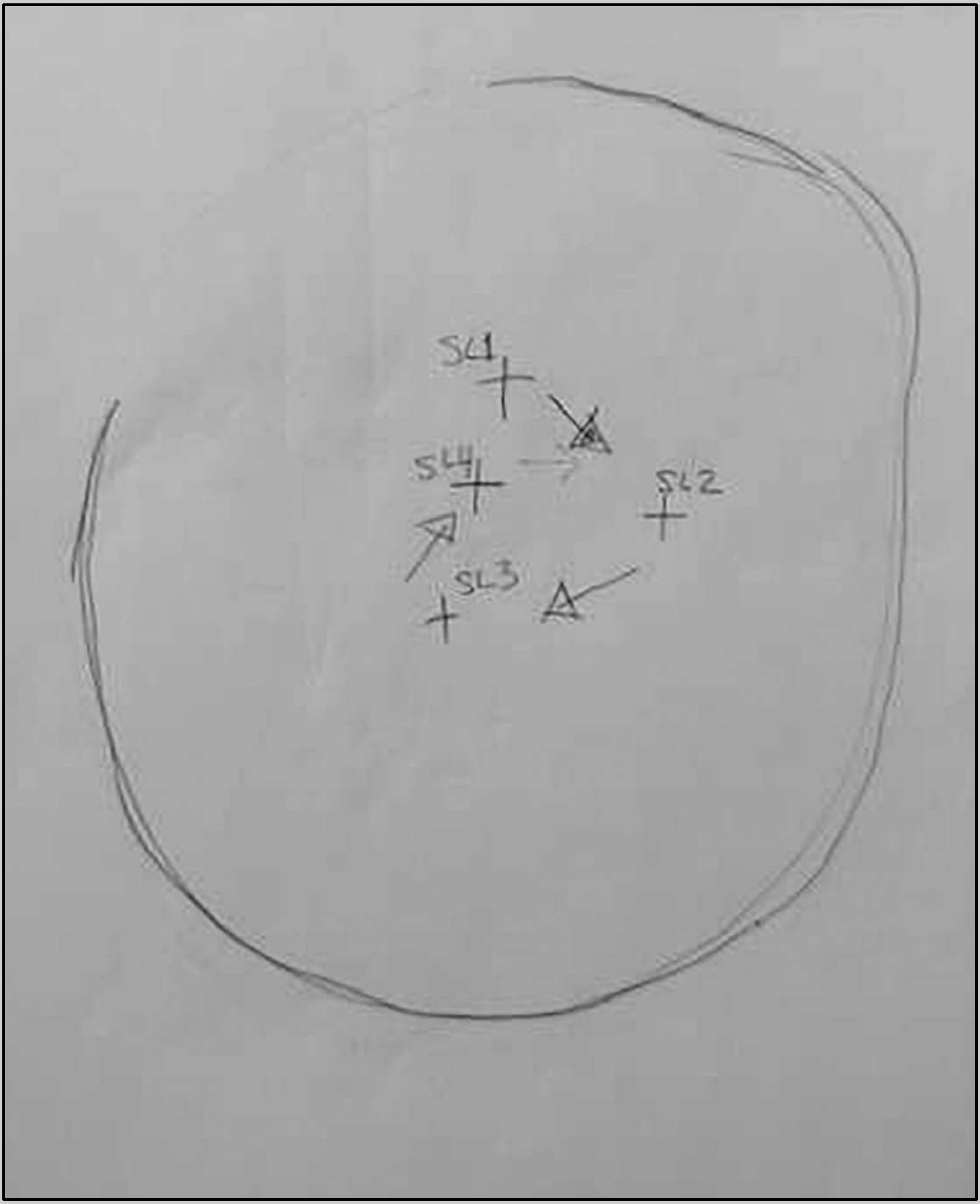

- B12

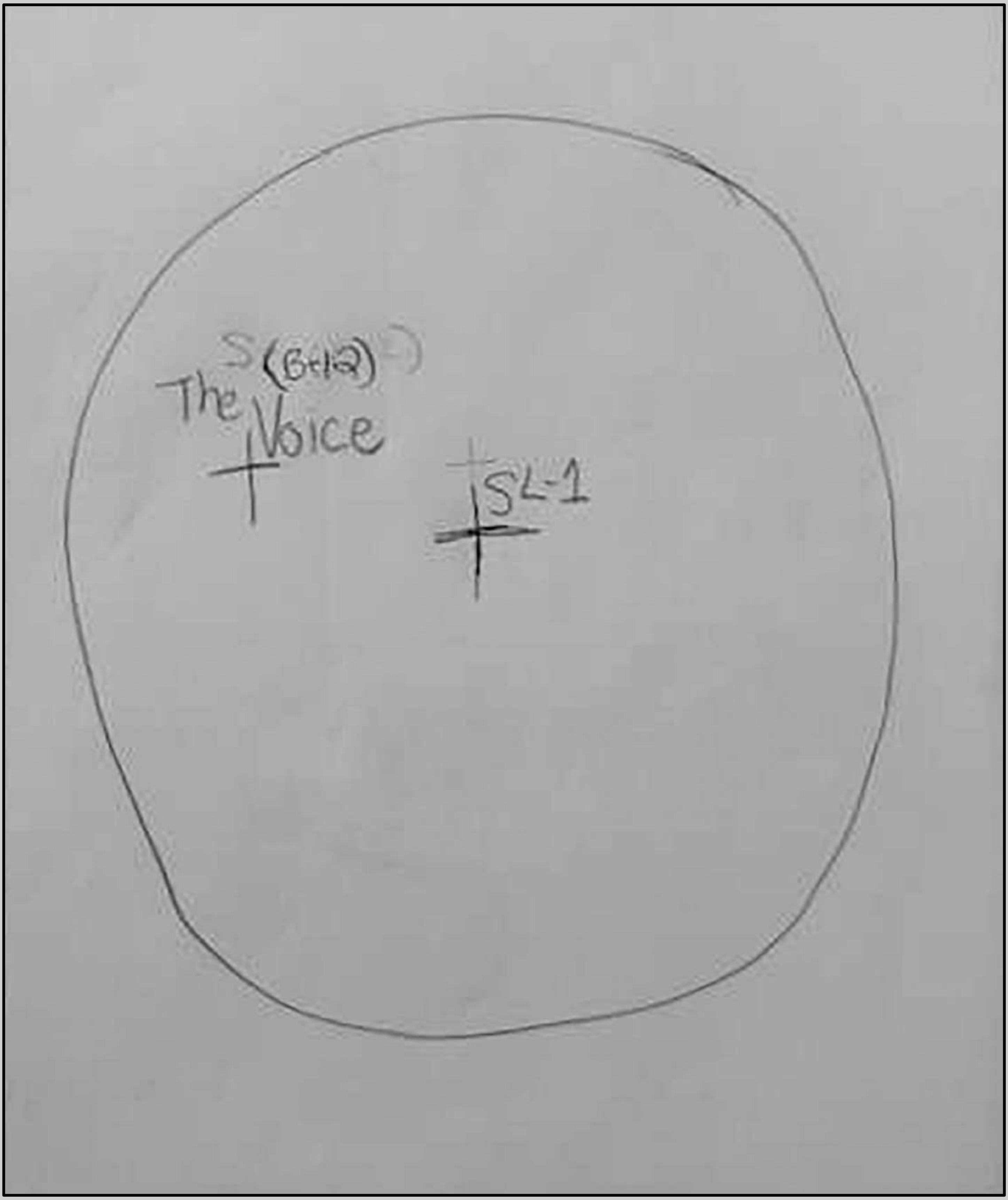

- C5

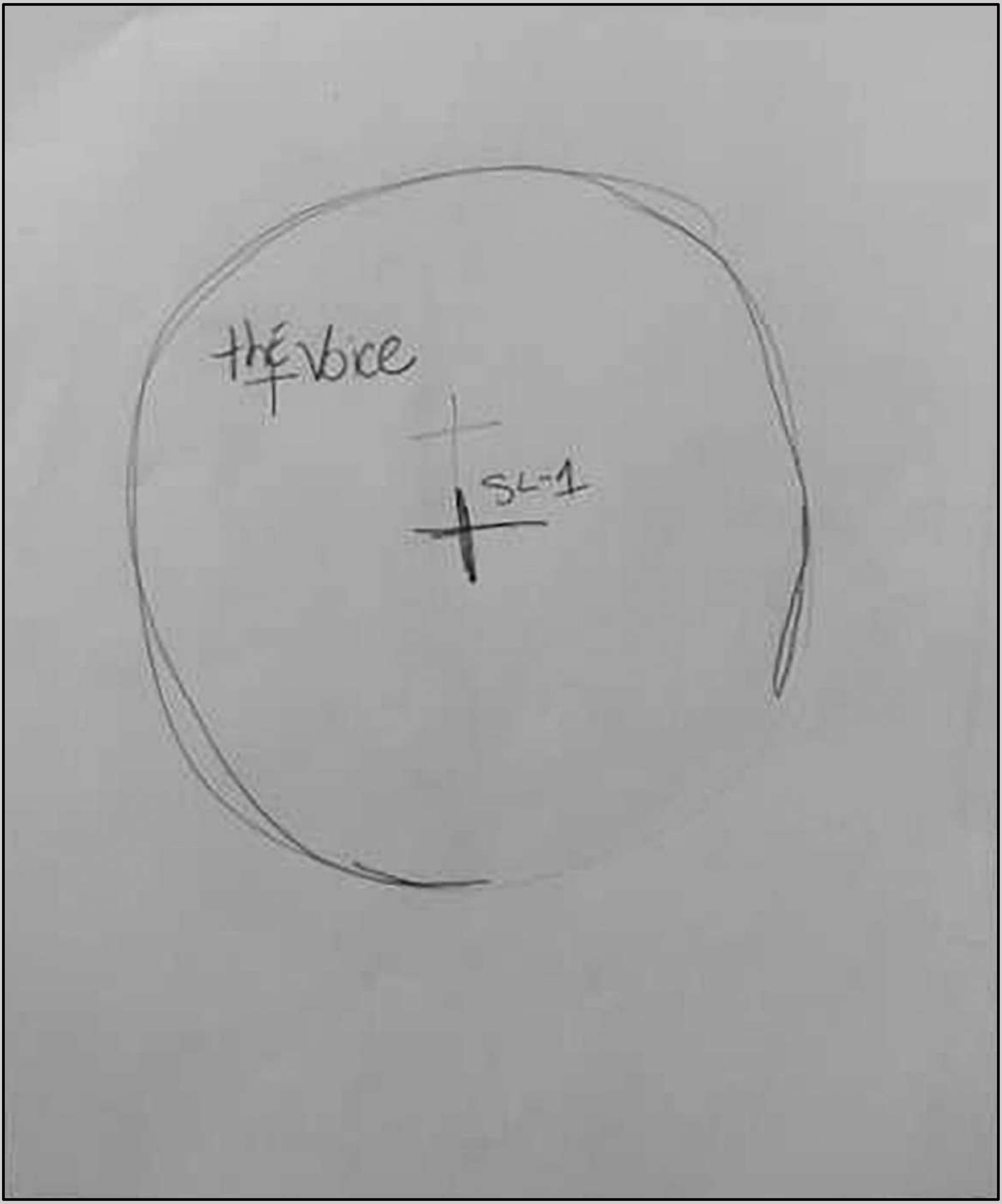

- B10

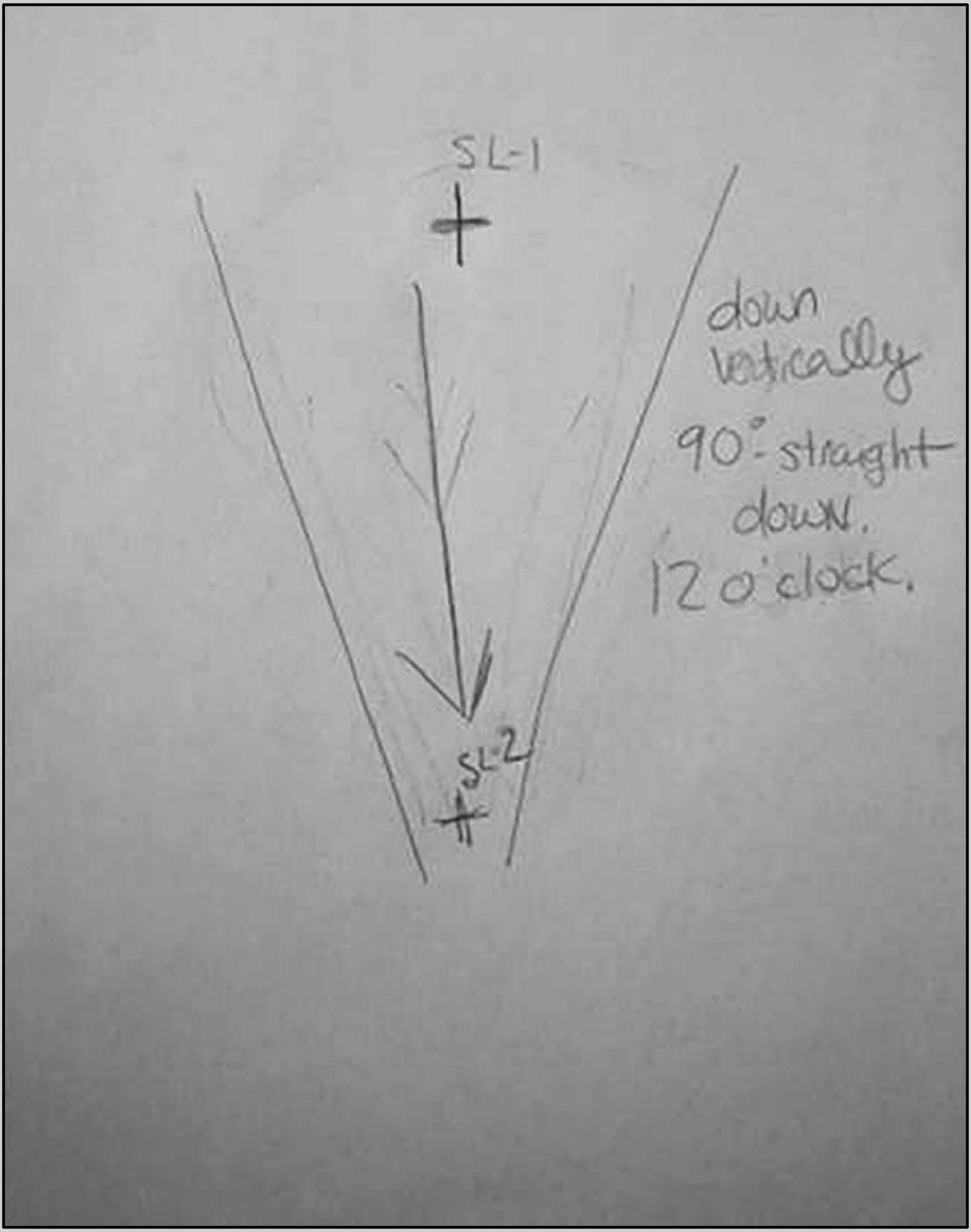

- A9

Subject 26

Sequence: part/space

1 NDE

- B4

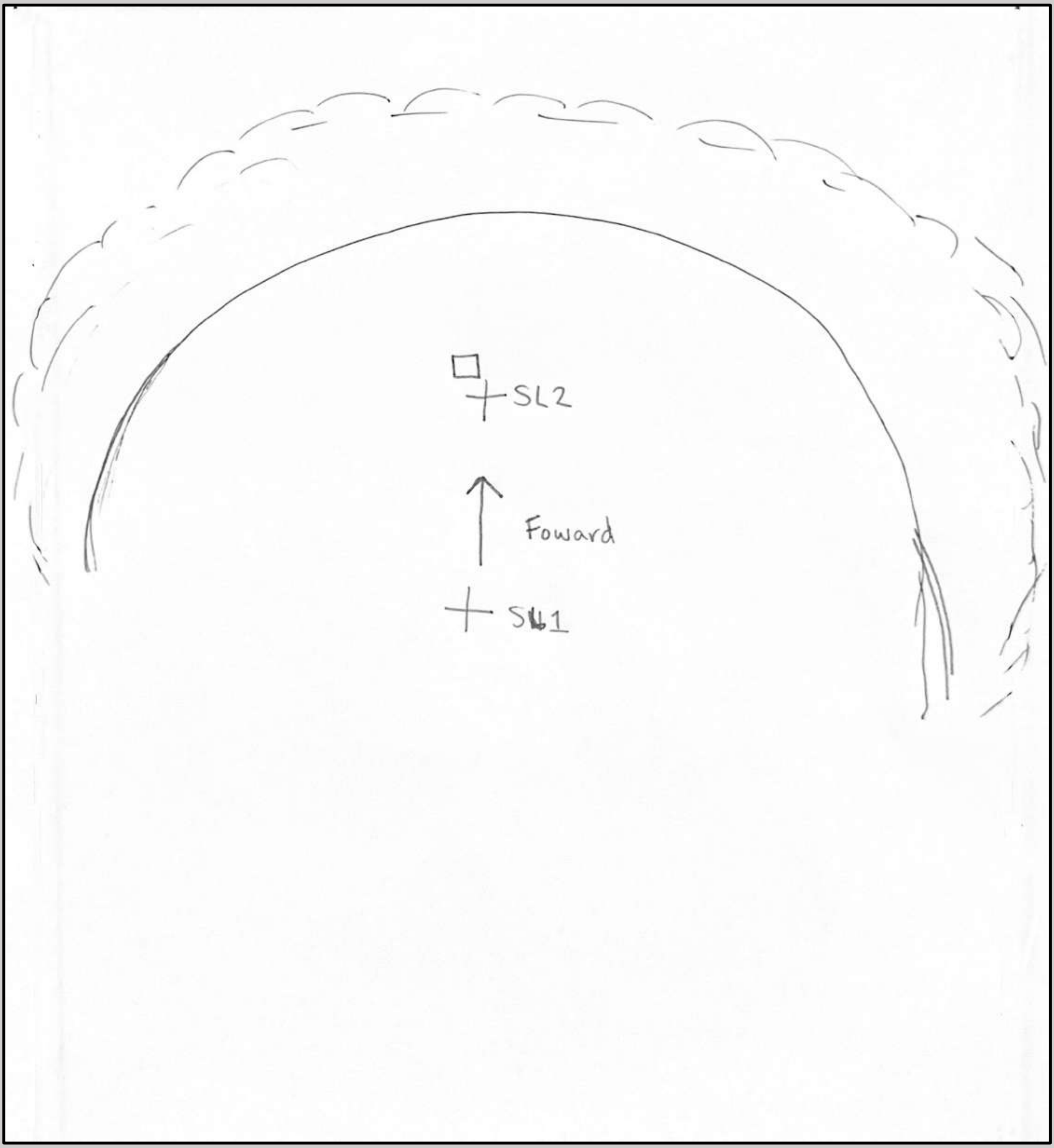

• B4

Subject 27

Sequence: part/space

1 NDE

- B6

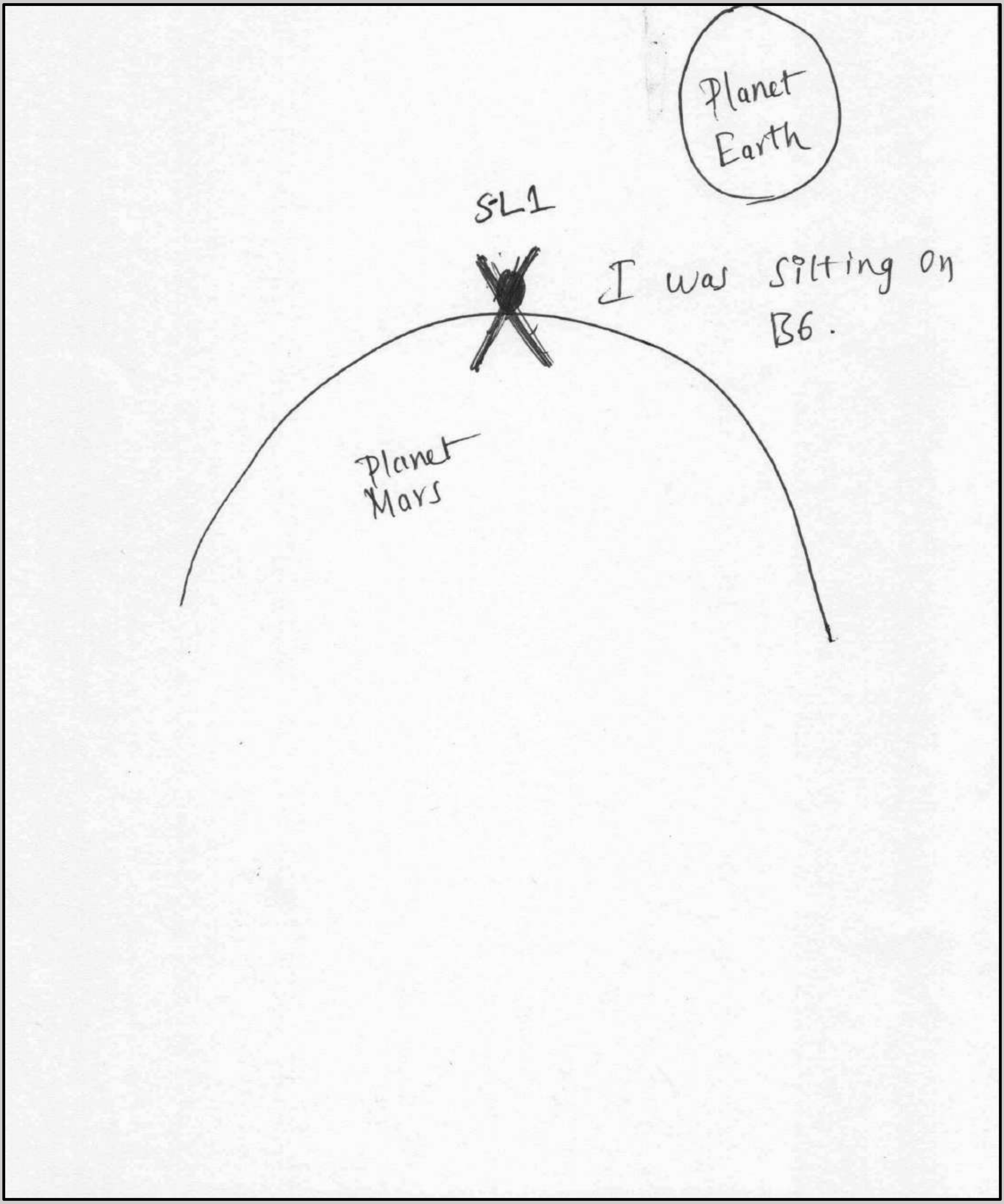

• B6

Subject 28

Sequence: part/space

3 NDE

- B6
- C2
- B11

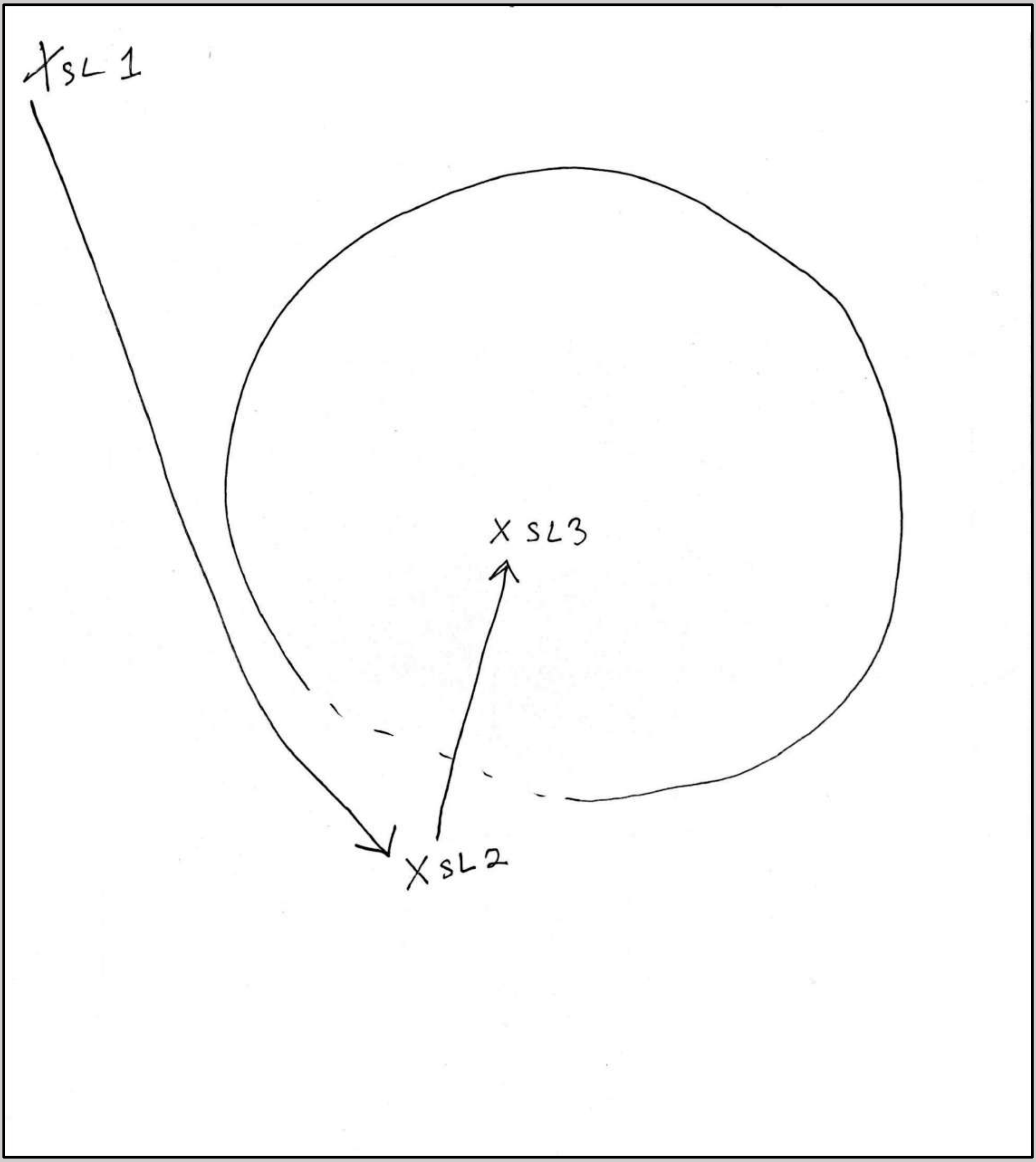

• B6

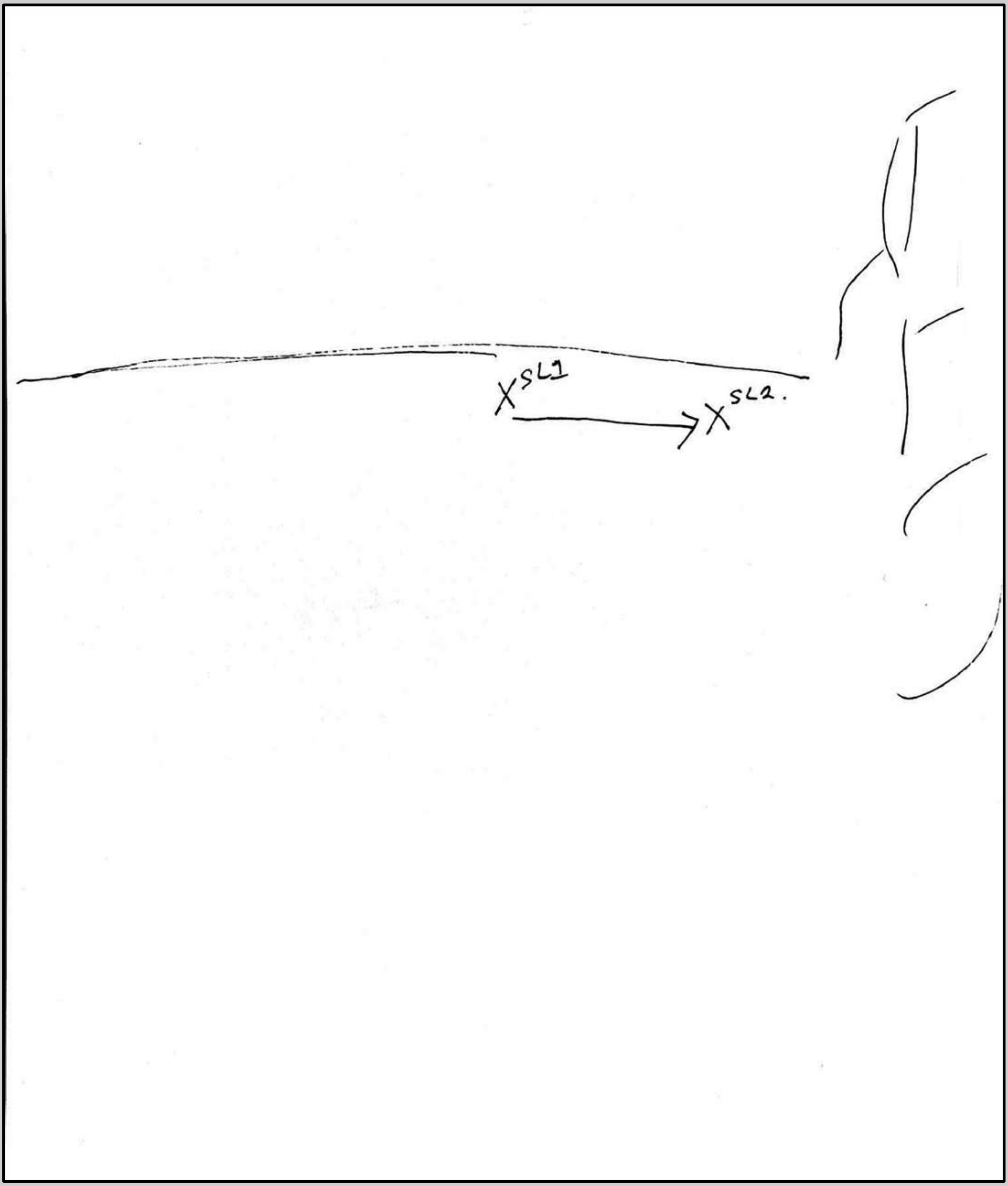

• C2

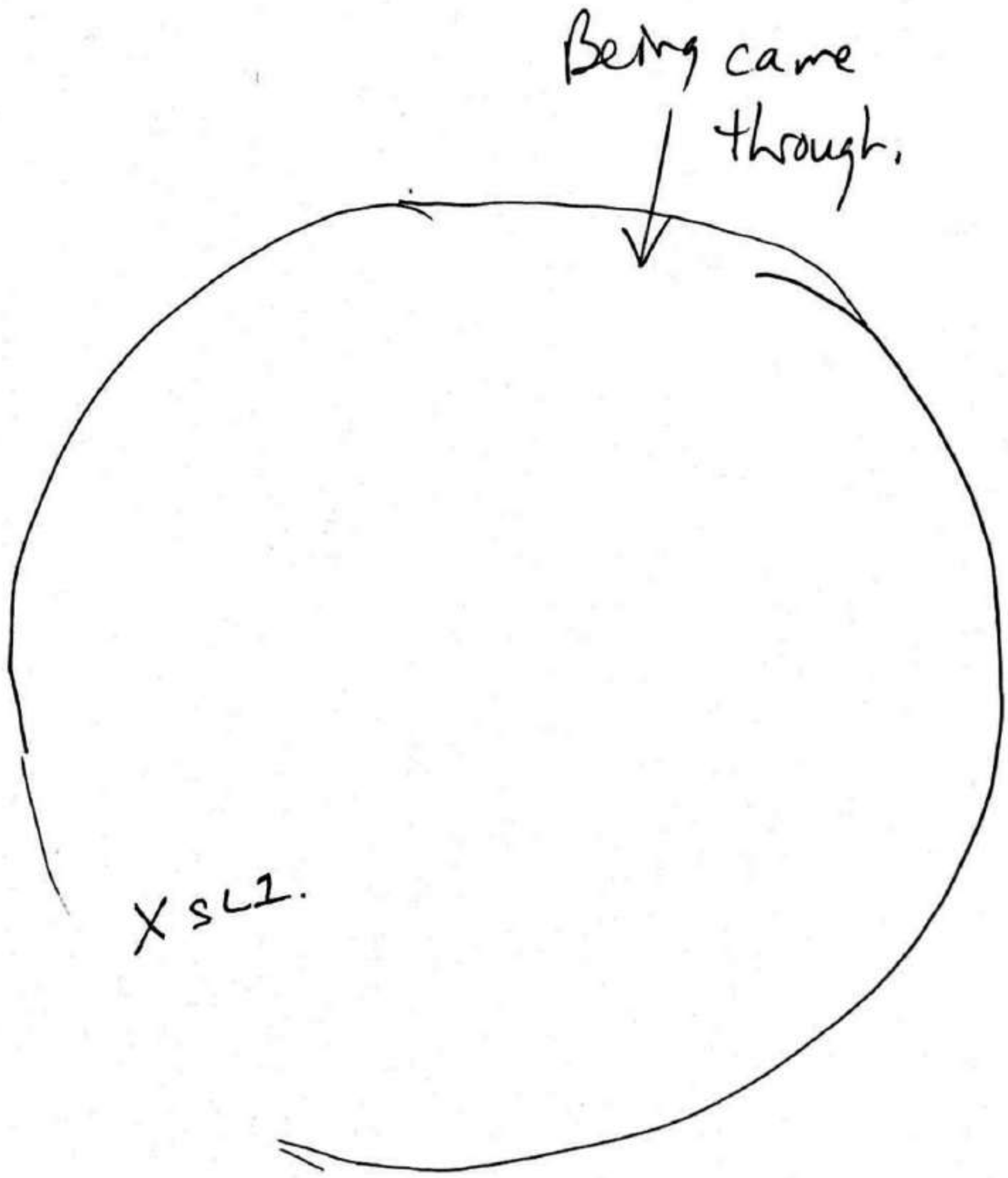

• B11

Subject 29

Sequence: part/space

2 OBE + 3 NDE

- OBE Dissociative
- B9
- B11
- B12
- OBE Associative

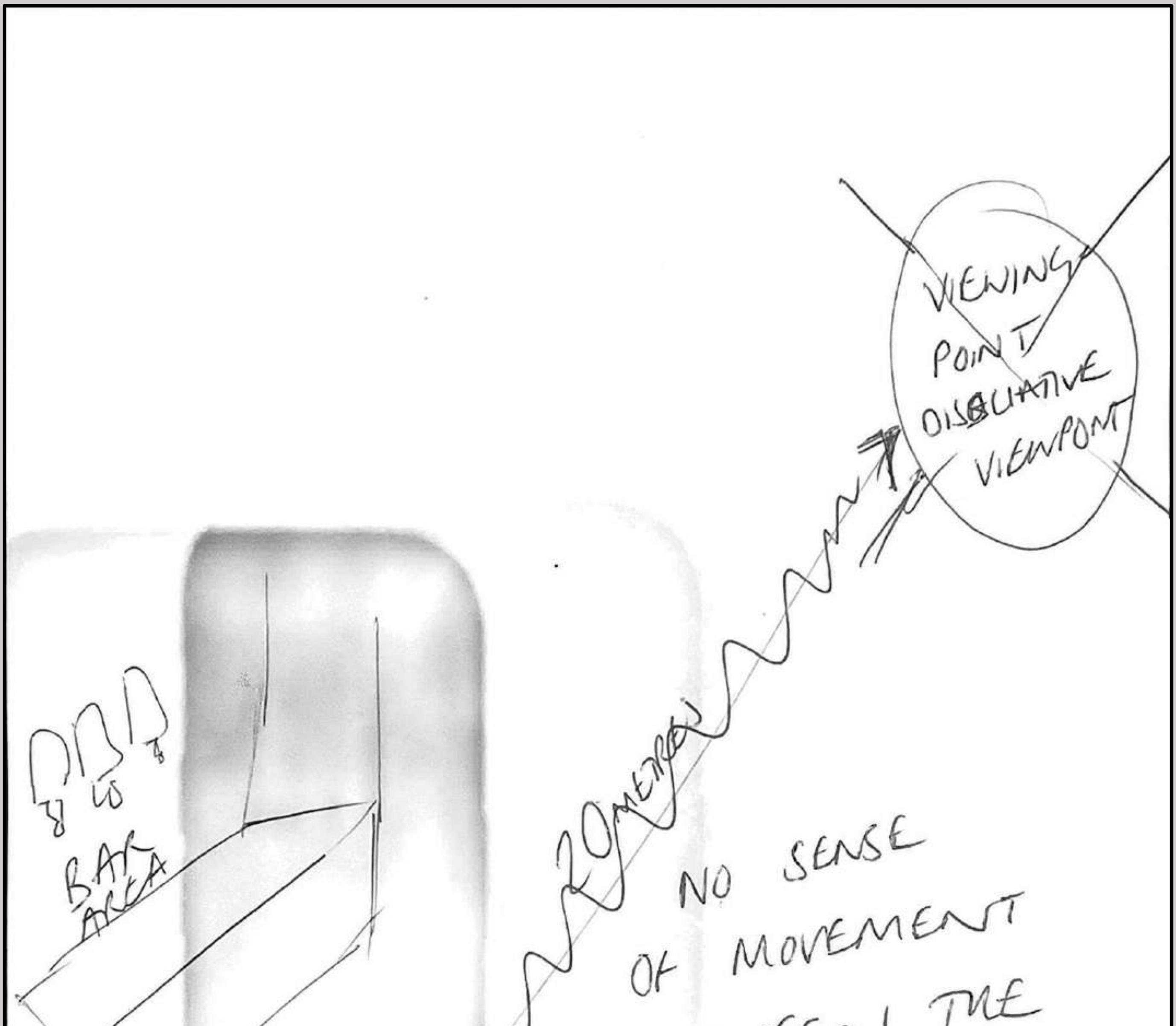

• OBE Dissociative

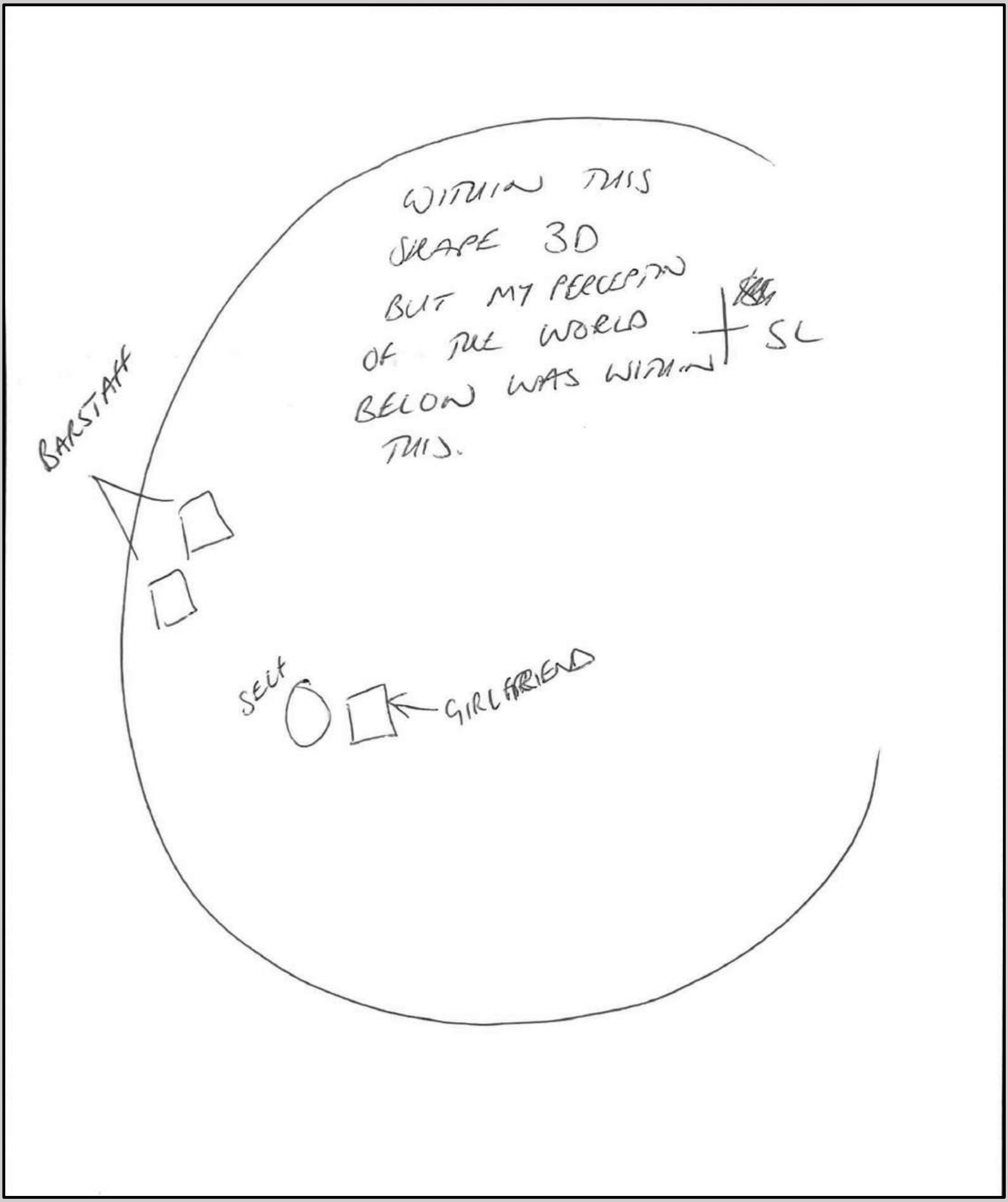
WITHIN THIS
SHAPE 3D
BUT MY PERCEPTION
OF THE WORLD
BELOW WAS WITHIN
THIS.
SL
BARSTAFF
SELF
GIRLFRIEND

• B9

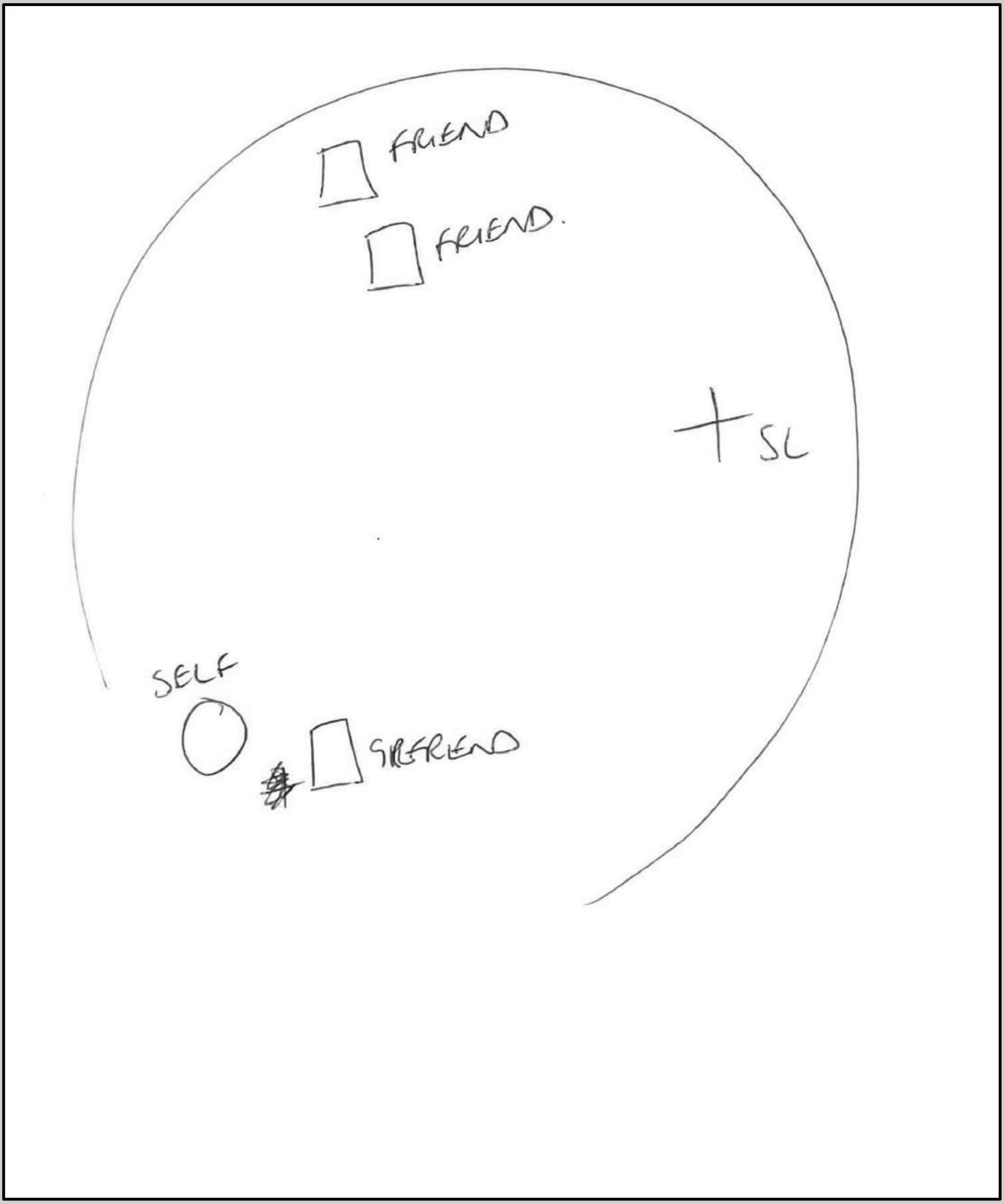

• B11

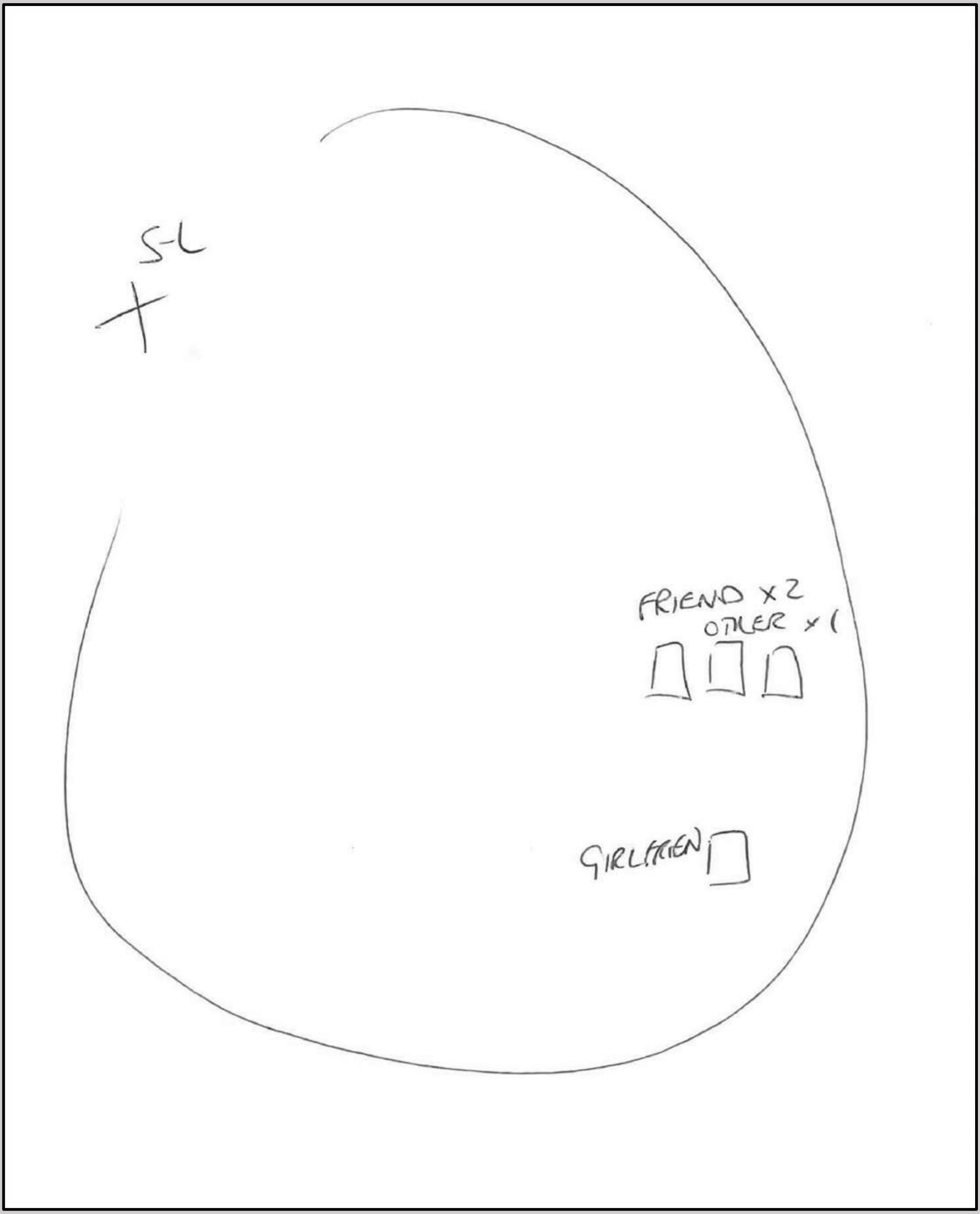

• B12

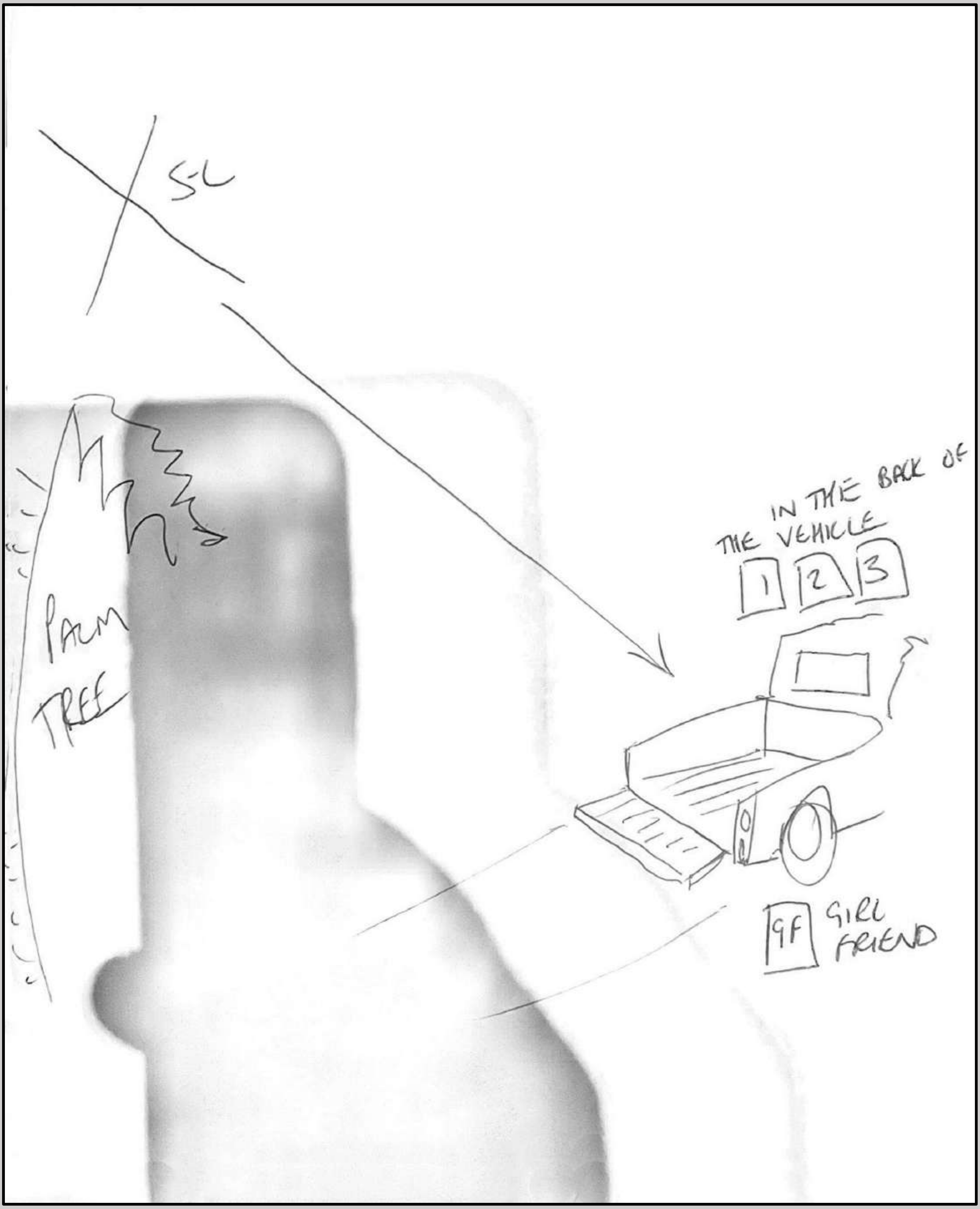

• OBE Associative

Subject 30

Sequence: part/space

2 OBE + 1 NDE

- OBE Dissociative
- C2
- OBE Associative

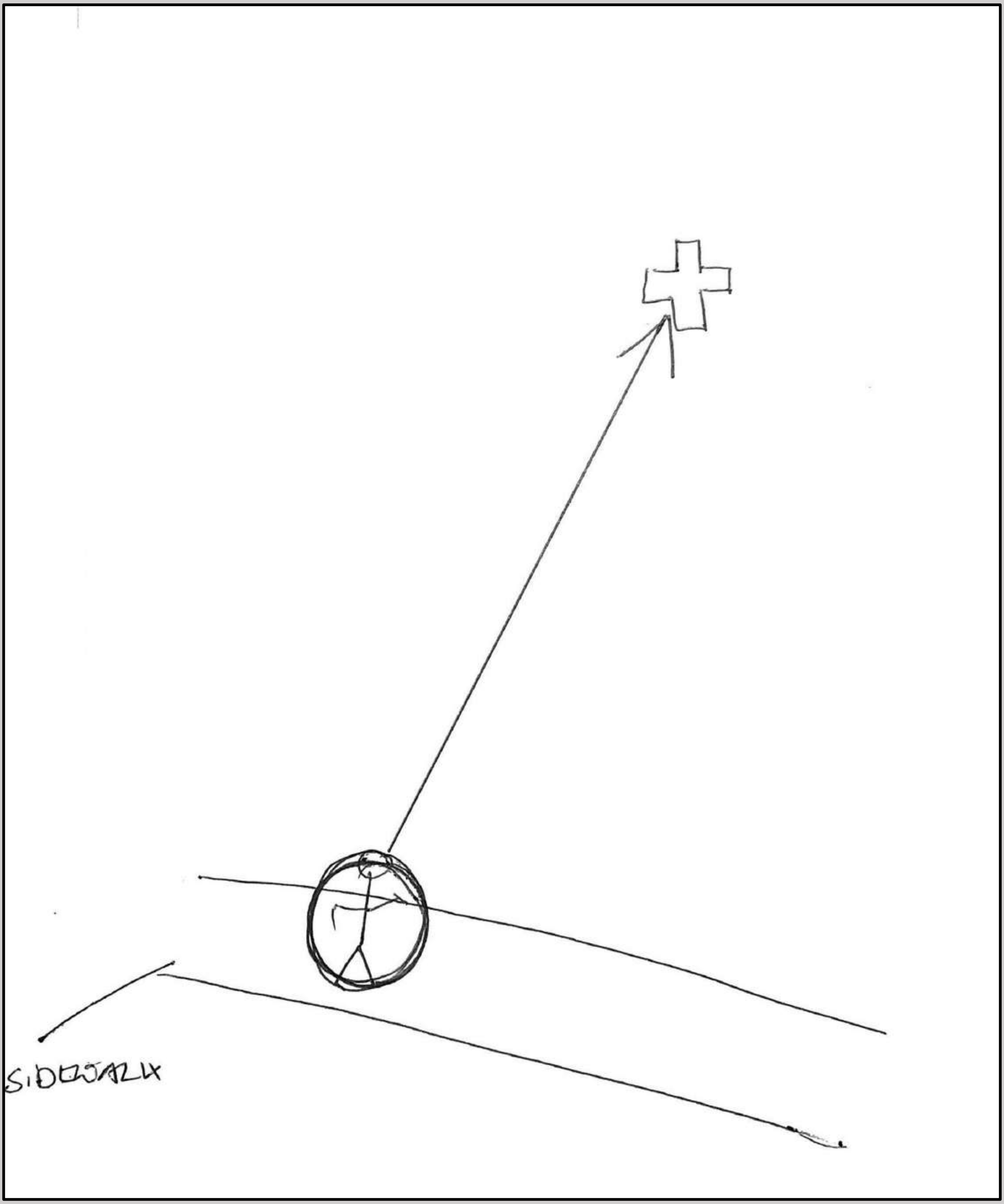

• OBE Dissociative

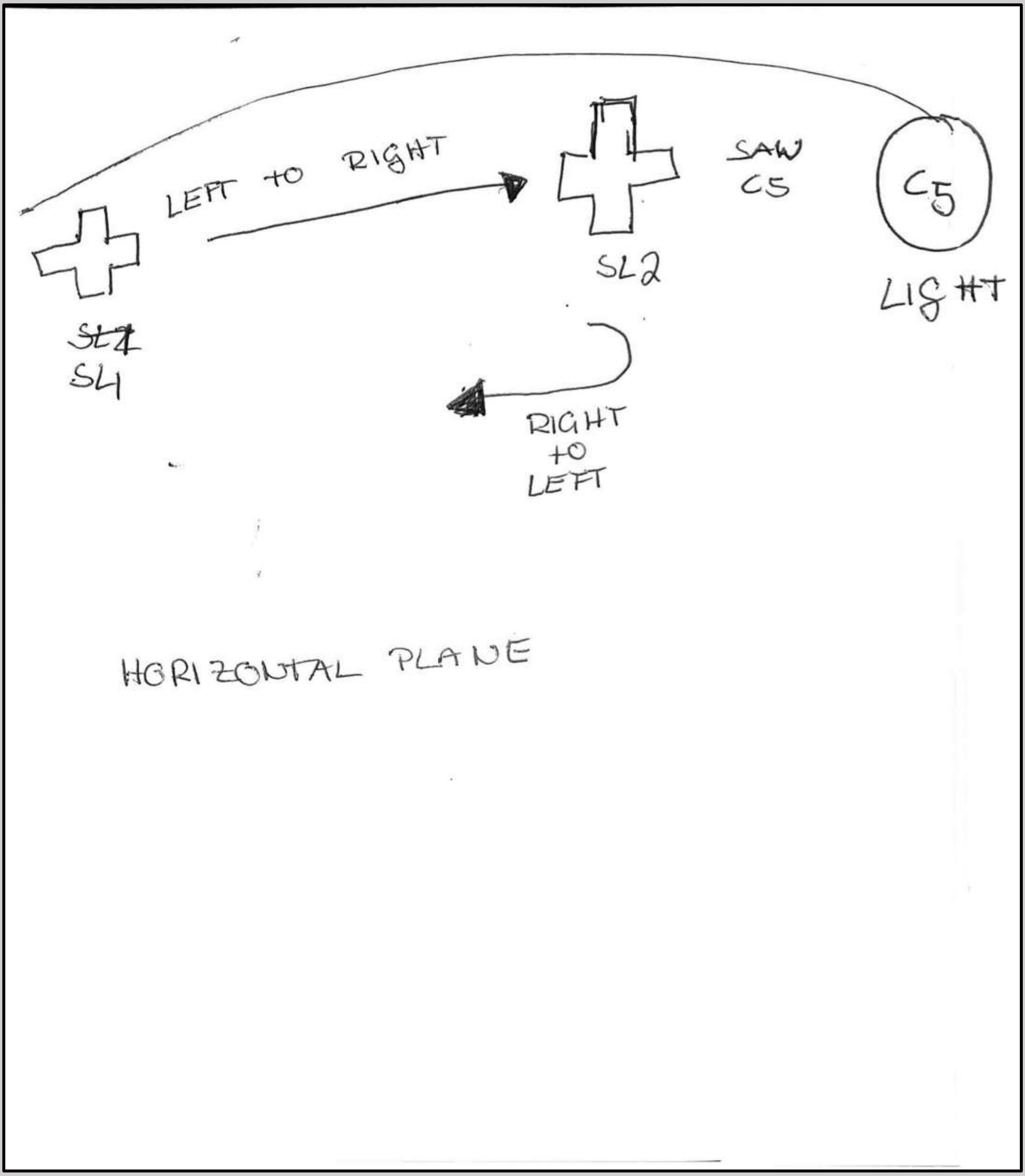
LEFT TO RIGHT
SL1
SL2
SAW C5
C5
LIGHT
RIGHT TO LEFT
HORIZONTAL PLANE

- C2

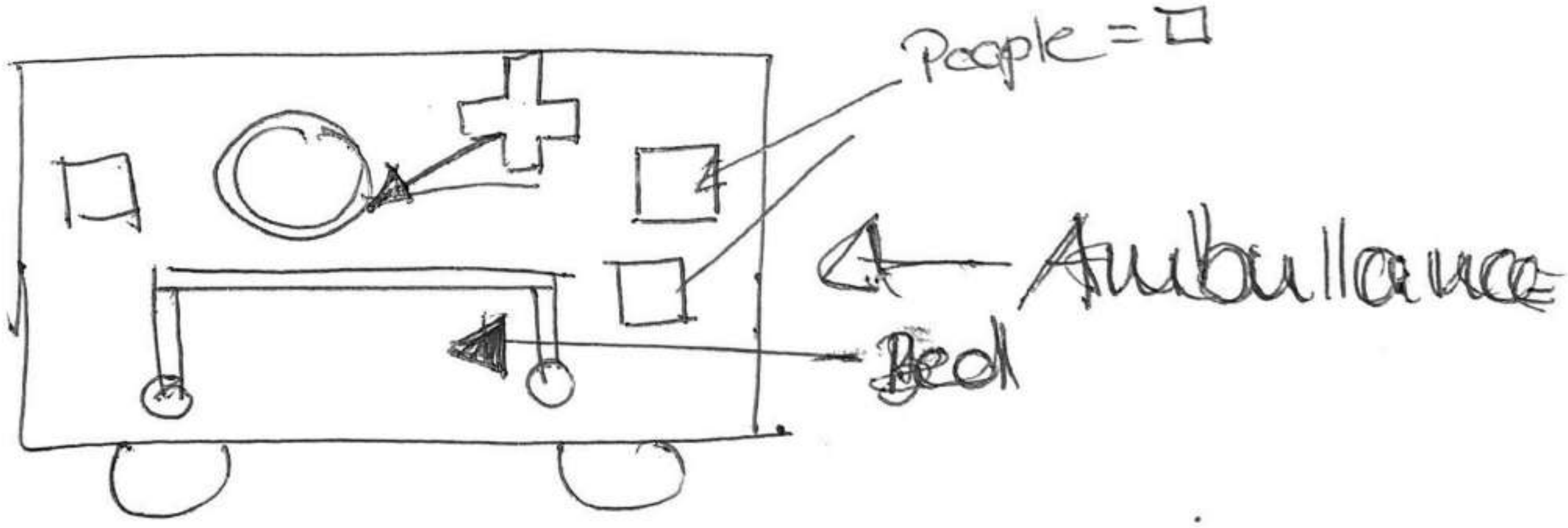

• OBE Associative

Subject 31

Sequence: part/space

1 NDE

- C2

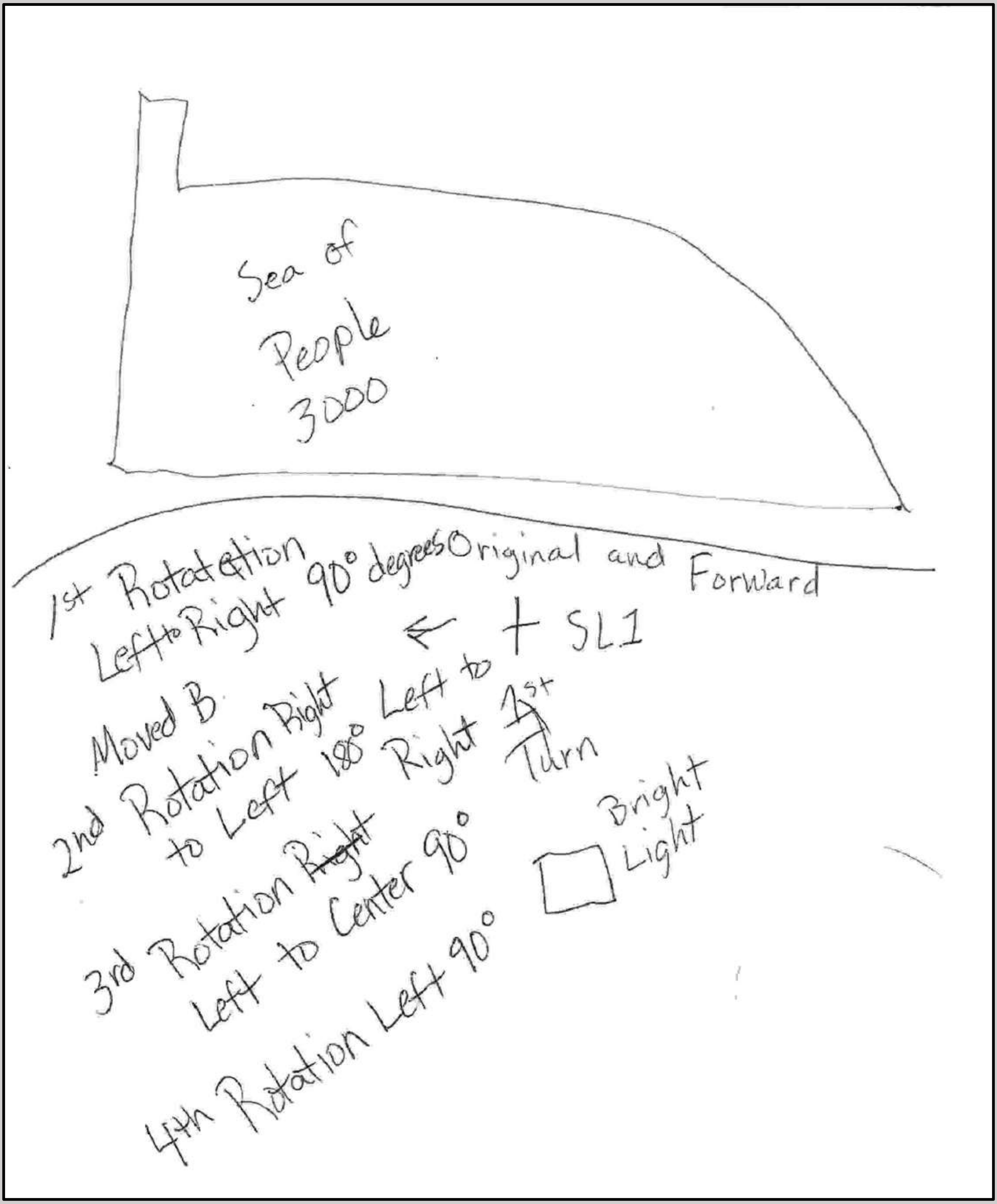
Sea of People 3000
1st Rotatetion Left to Right 90° degrees Original and Forward
SL1
Moved B
2nd Rotation Right to Left 180° Left to Right 1st Turn
3rd Rotation Right Left to Center 90°
Bright Light
4th Rotation Left 90°

- C2

Subject 32

Sequence: part/space

2 NDE

- C2
- A9

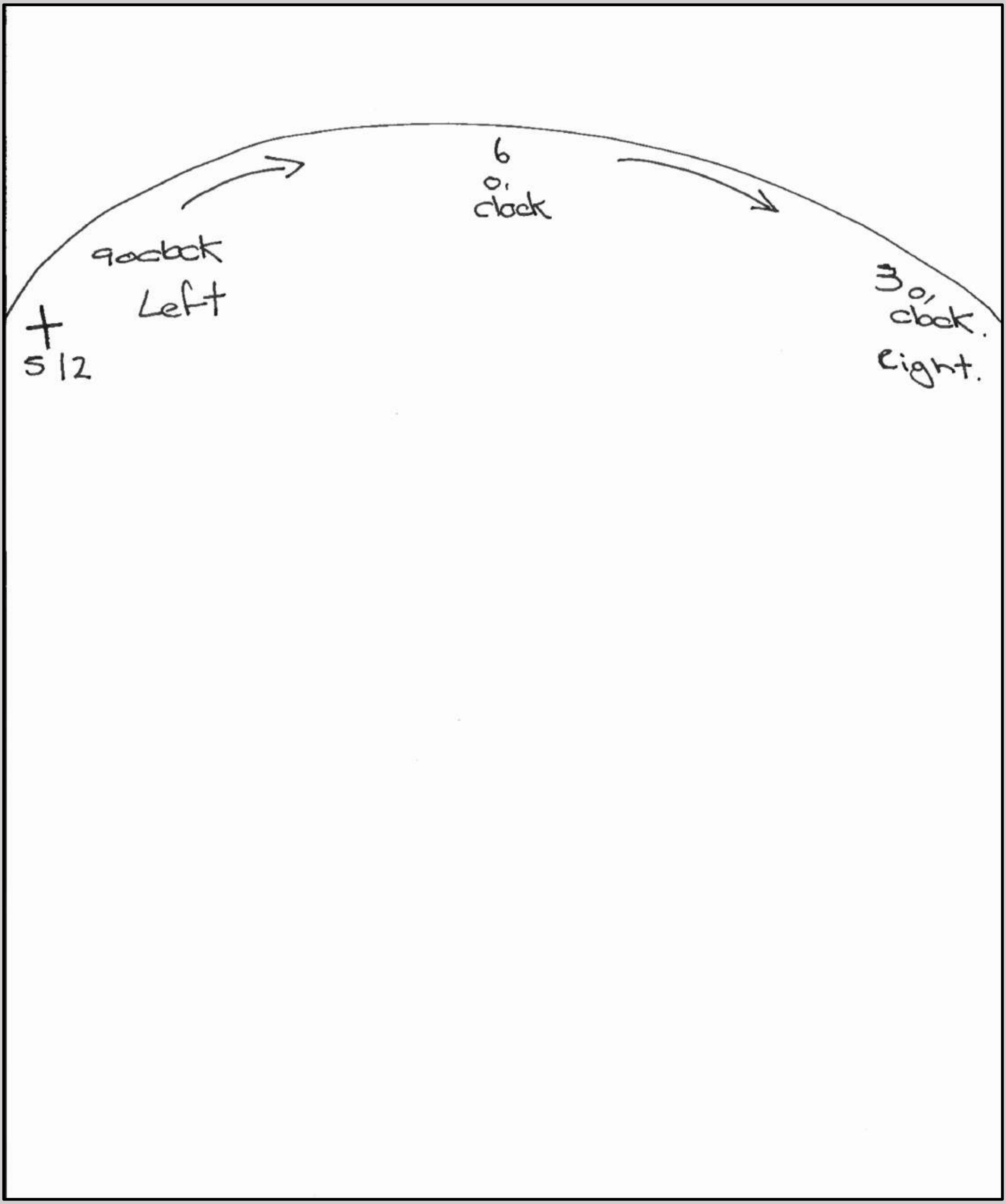

• C2

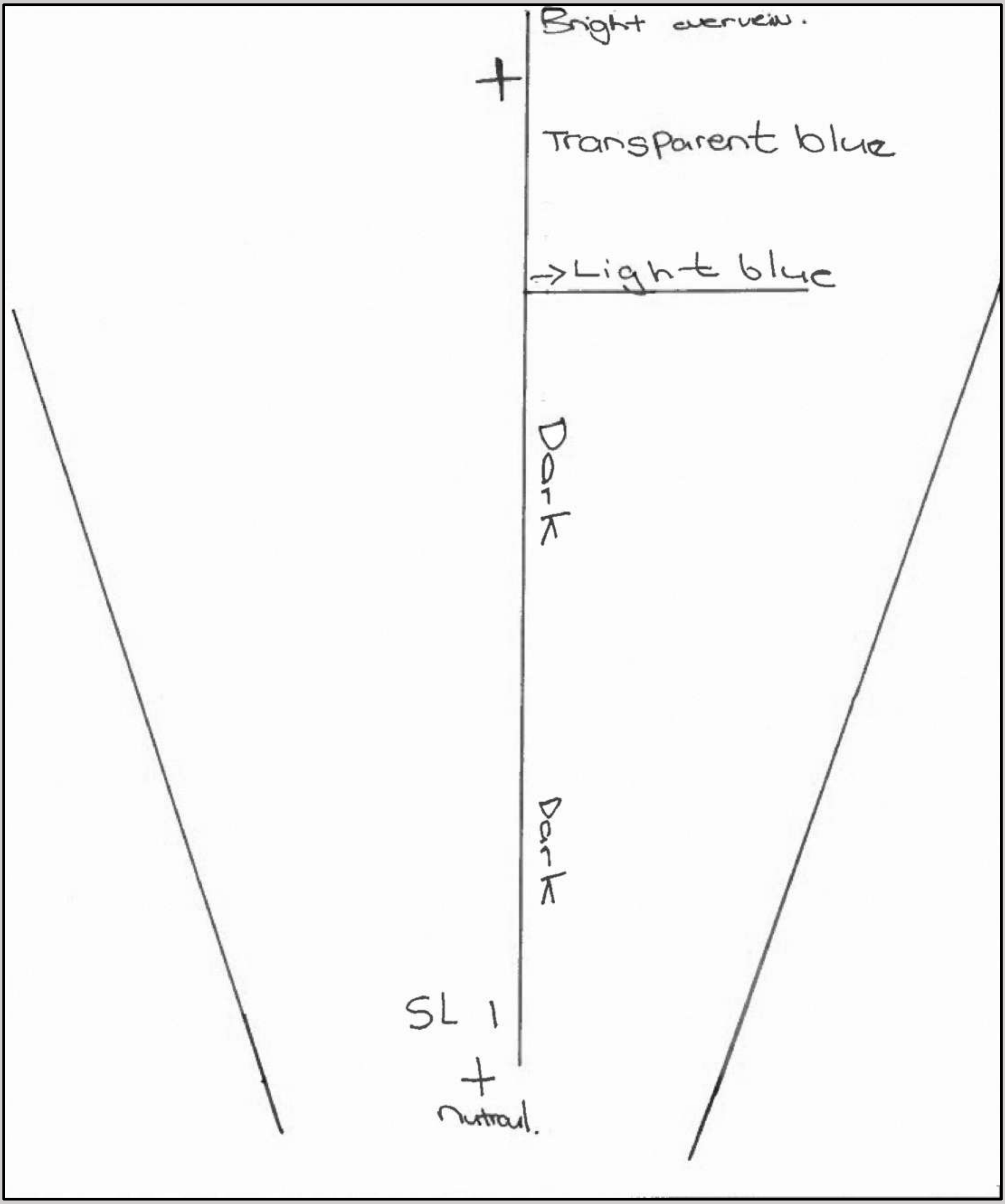
Bright overvein.
+
Transparent blue
->Light blue
Dark
Dark
SL 1
+
nutraul.

• A9

Subject 33

Sequence: part/space

2 OBE + 1 NDE

- OBE Dissociative
- C2
- OBE Associative

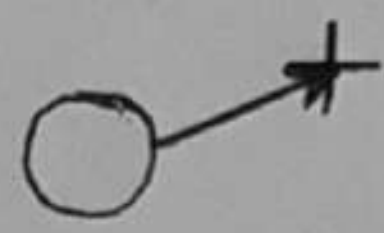

behind, to the right
2-8 back

- OBE Dissociative

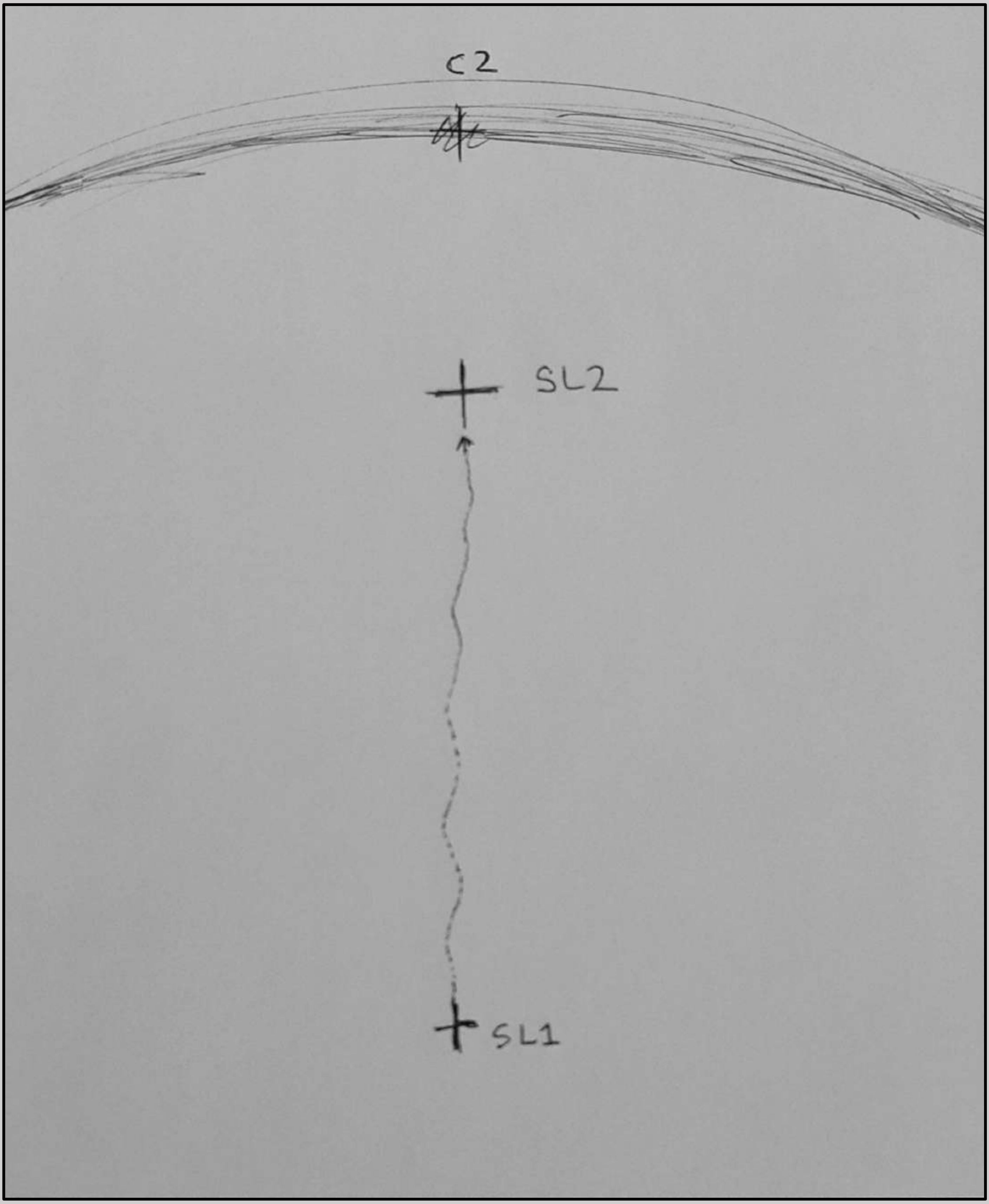

• C2

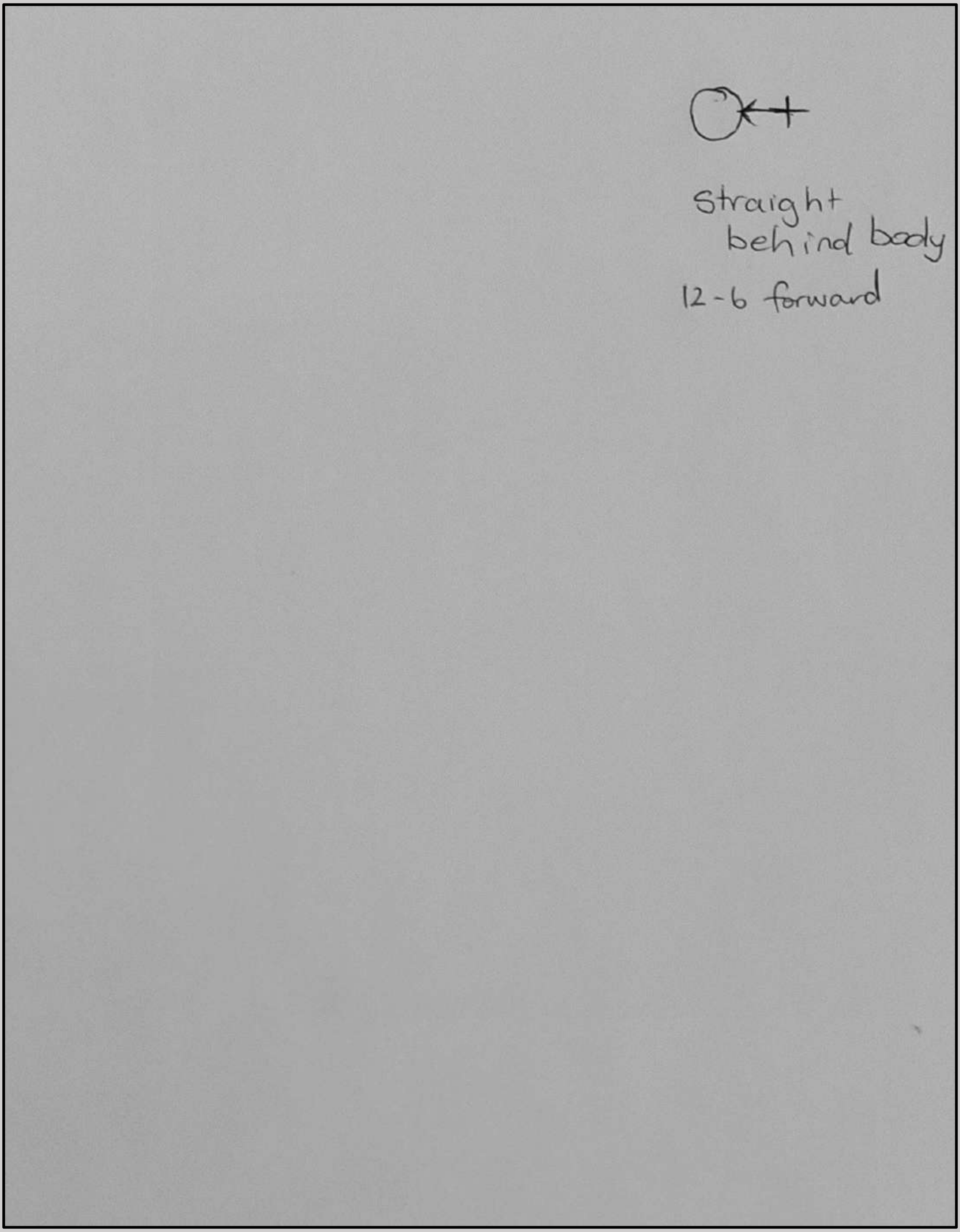

- OBE Associative

Subject 34

Sequence: part/space

2 OBE + 1 NDE

OBE Dissociative

- C2

- OBE Associative

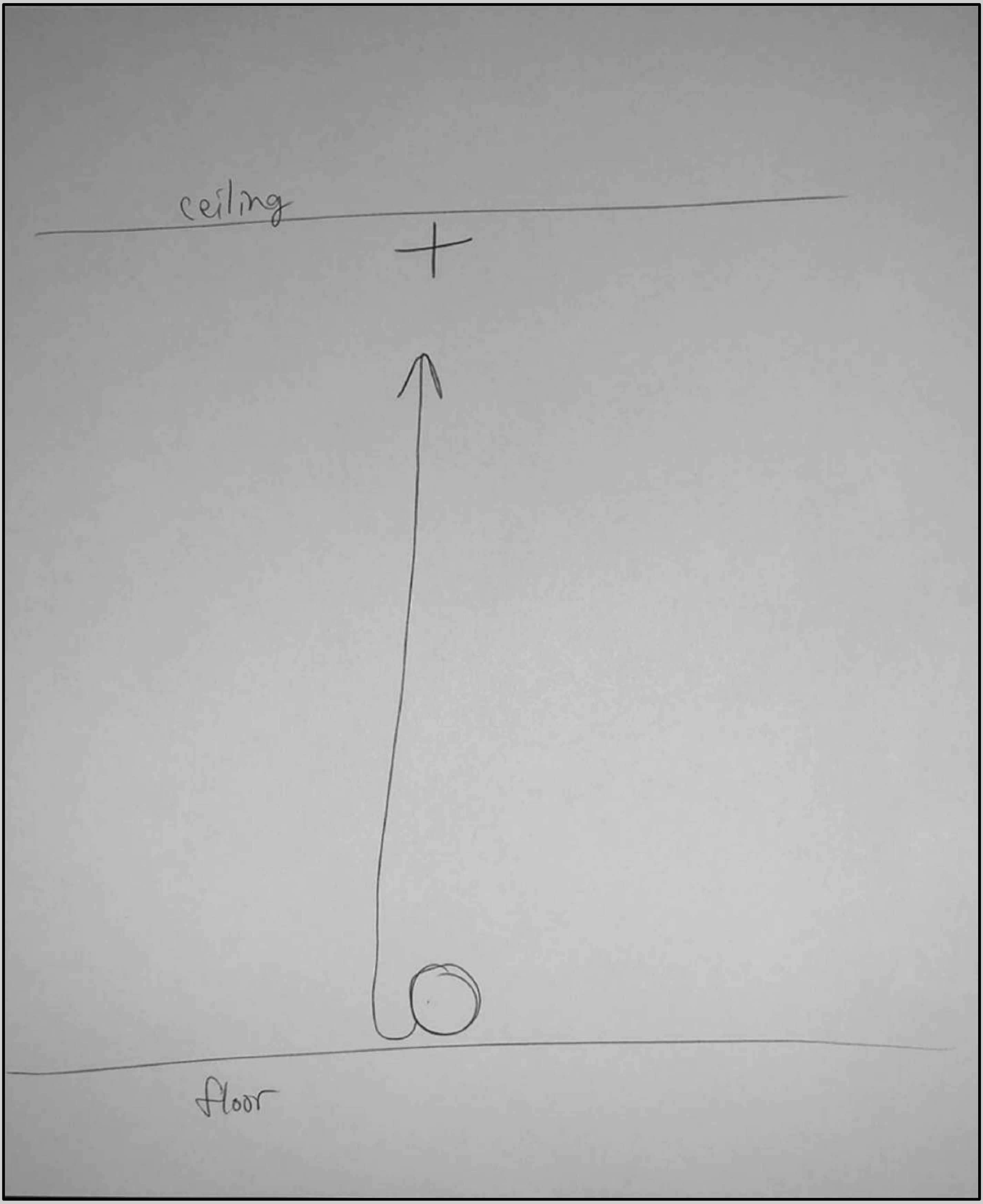

- OBE Dissociative

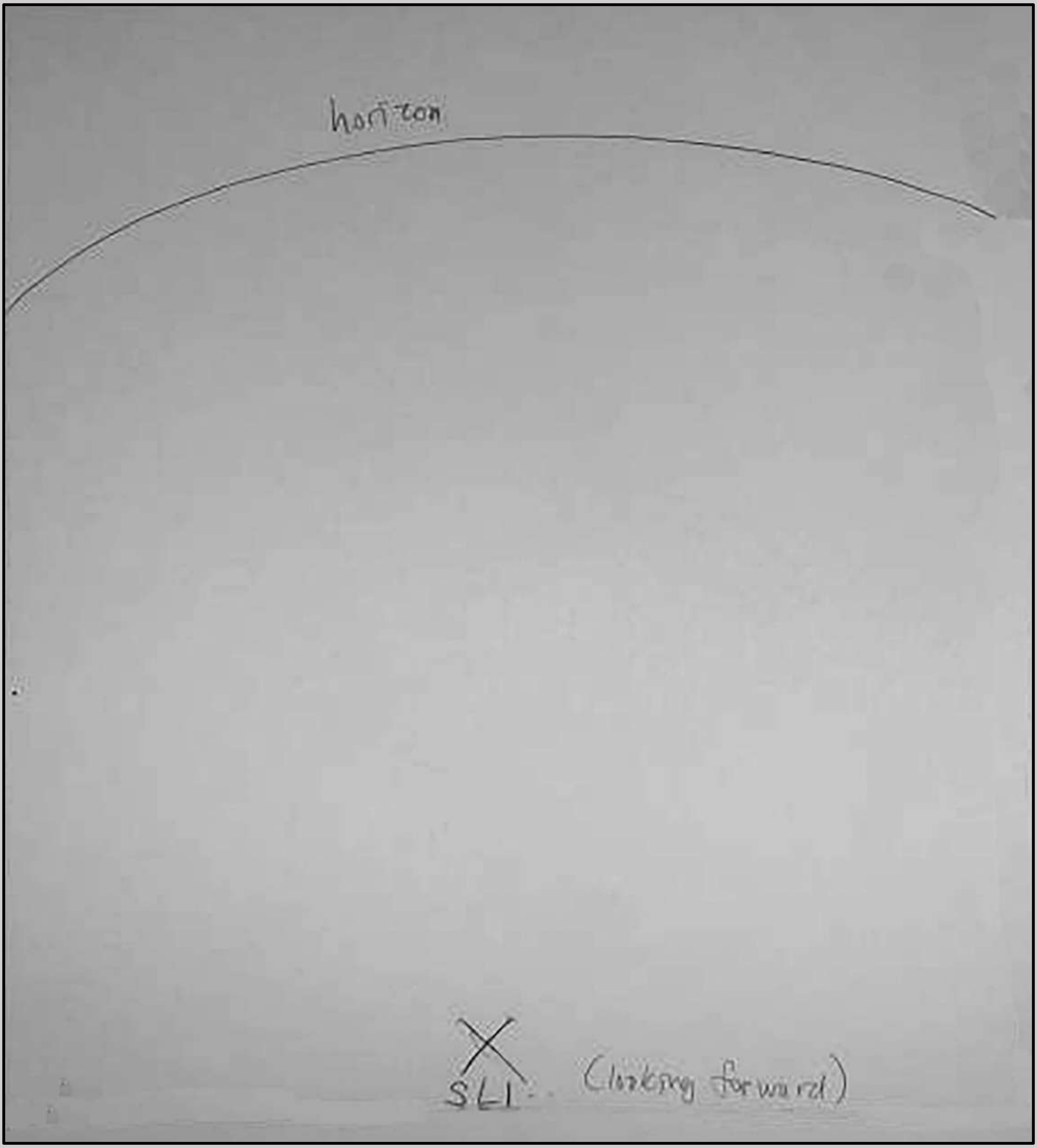

- C2

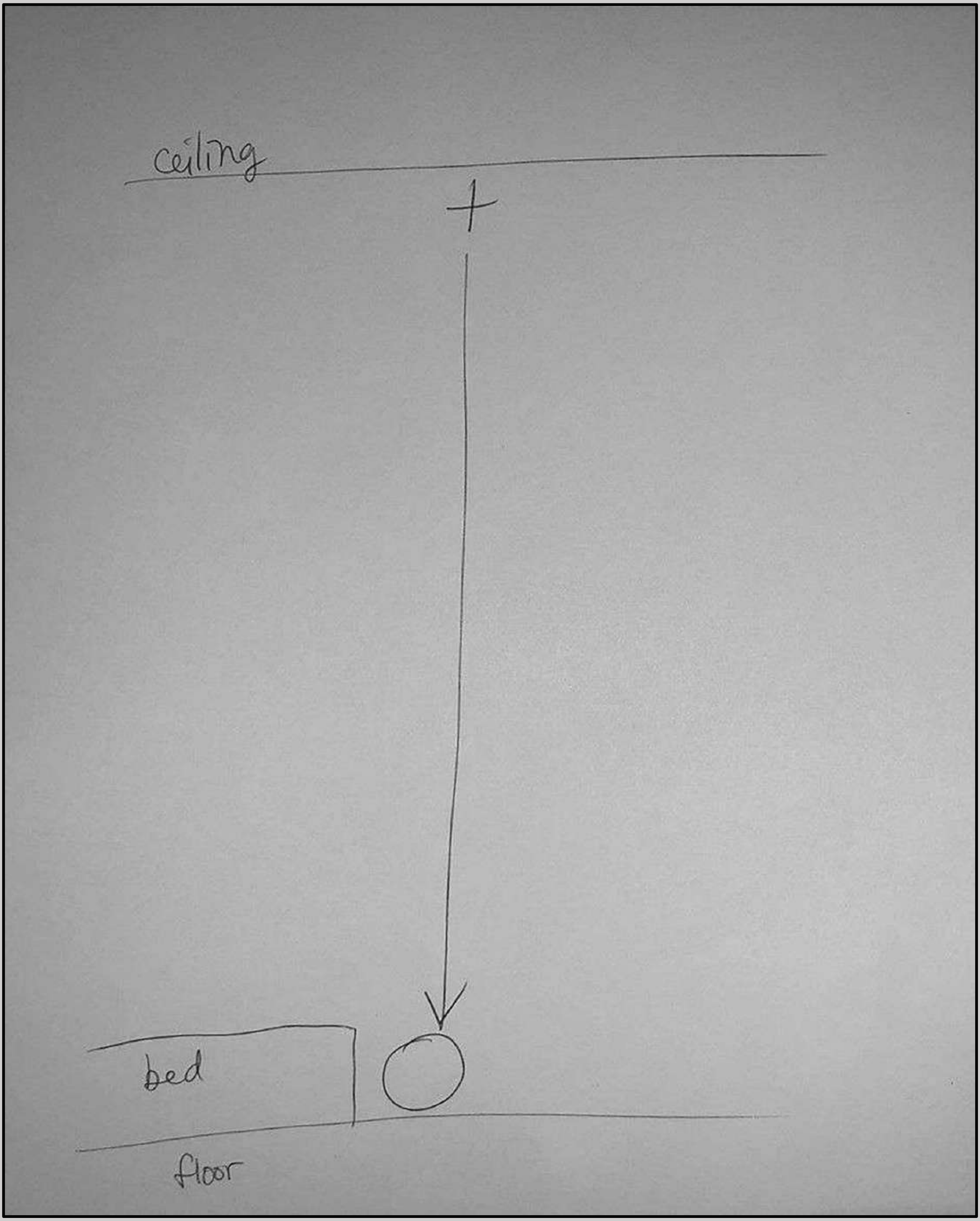

- OBE Associative

Subject 35

Sequence: part/space

1 NDE

- C2

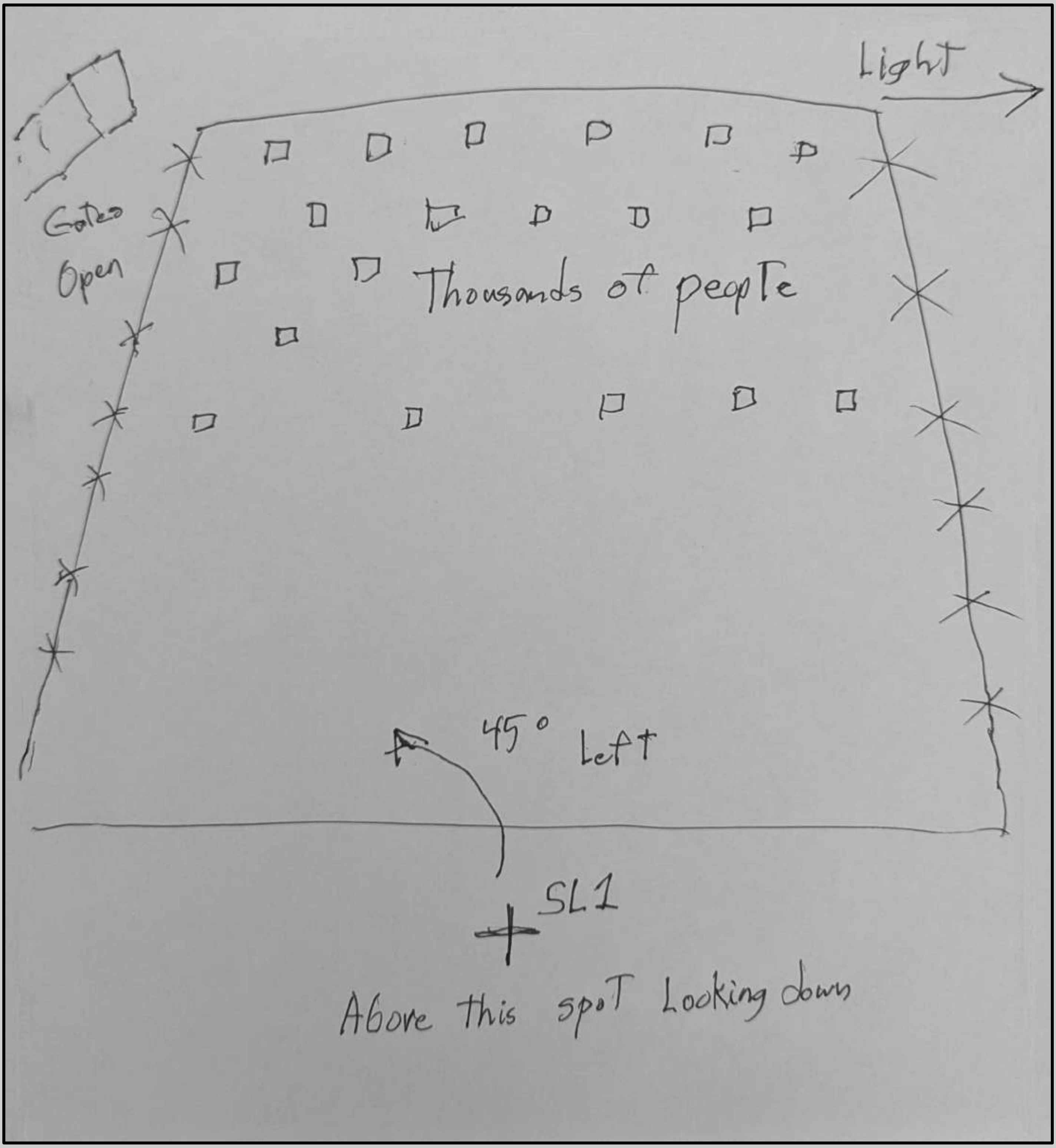

Light
Gates Open
Thousands of peopLe
45° Left
SL1
Above this spoT Looking down

• C2

Subject 36

Sequence: part/space

1 NDE

- C2

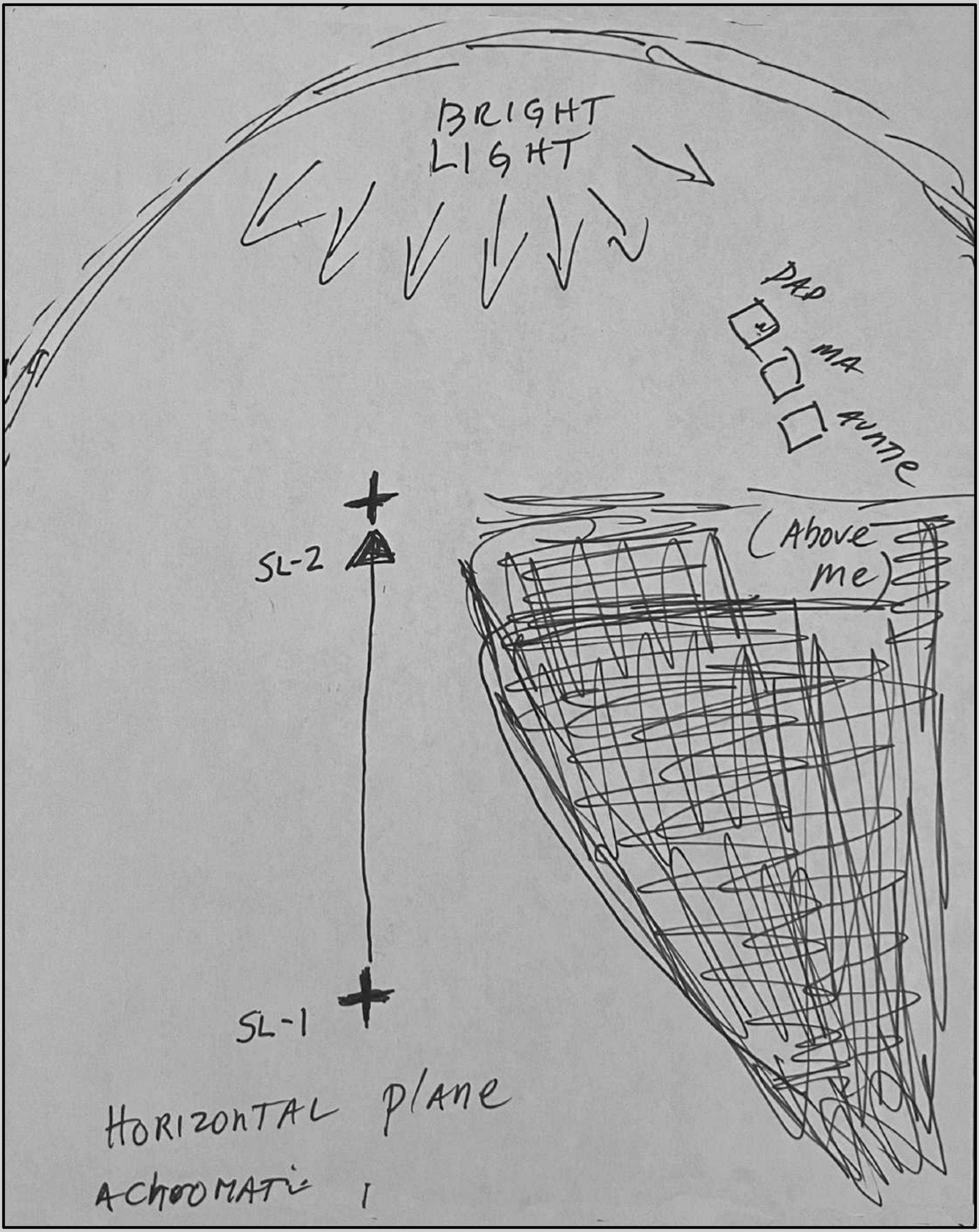
BRIGHT LIGHT
DAD
MA
Auntie
(Above me)
SL-2
SL-1
HORIZONTAL plane

• C2

Subject 37

Sequence: part/space

1 OBE + 1 NDE

- OBE Dissociative
- C3

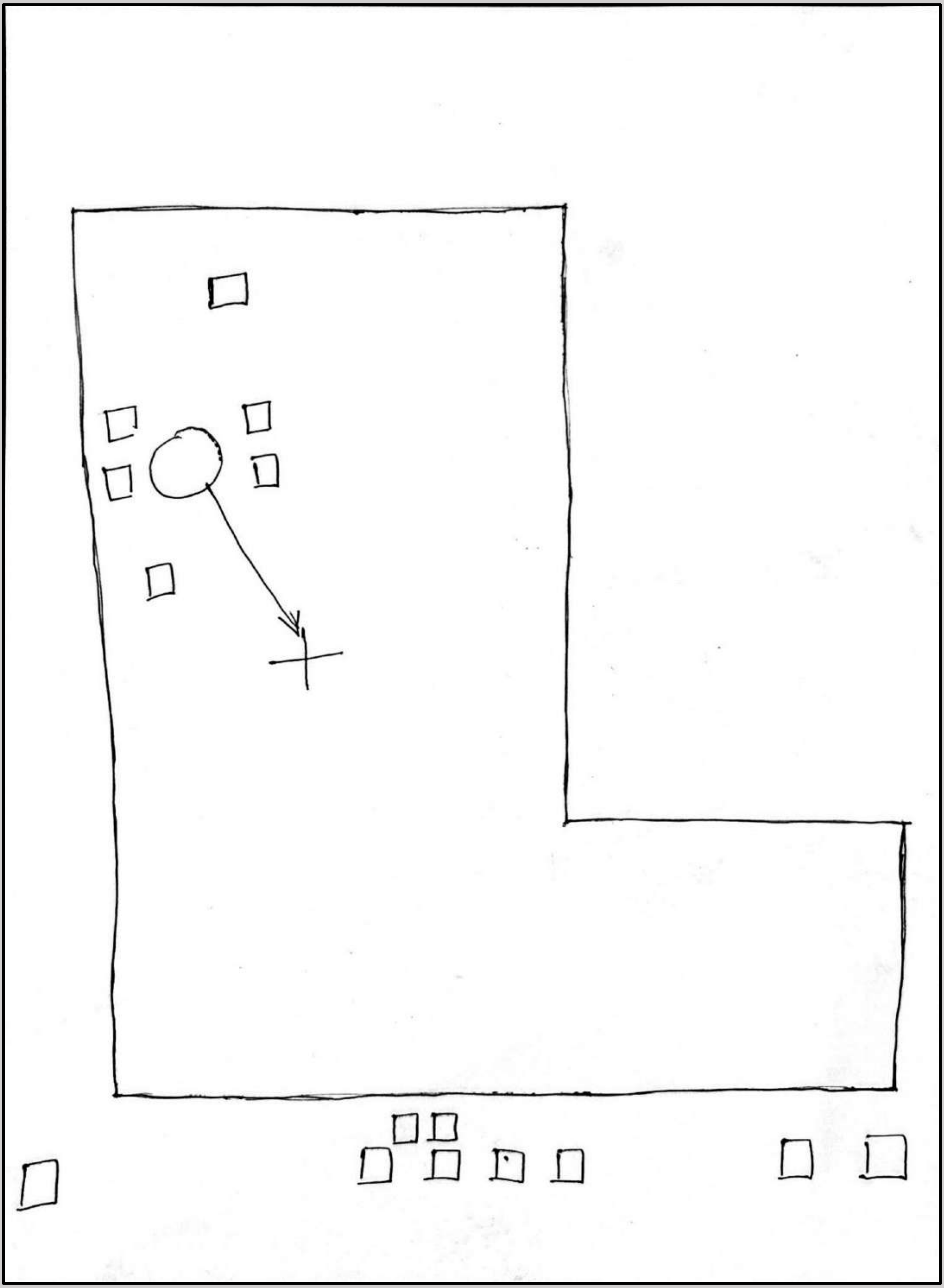

- OBE Dissociative

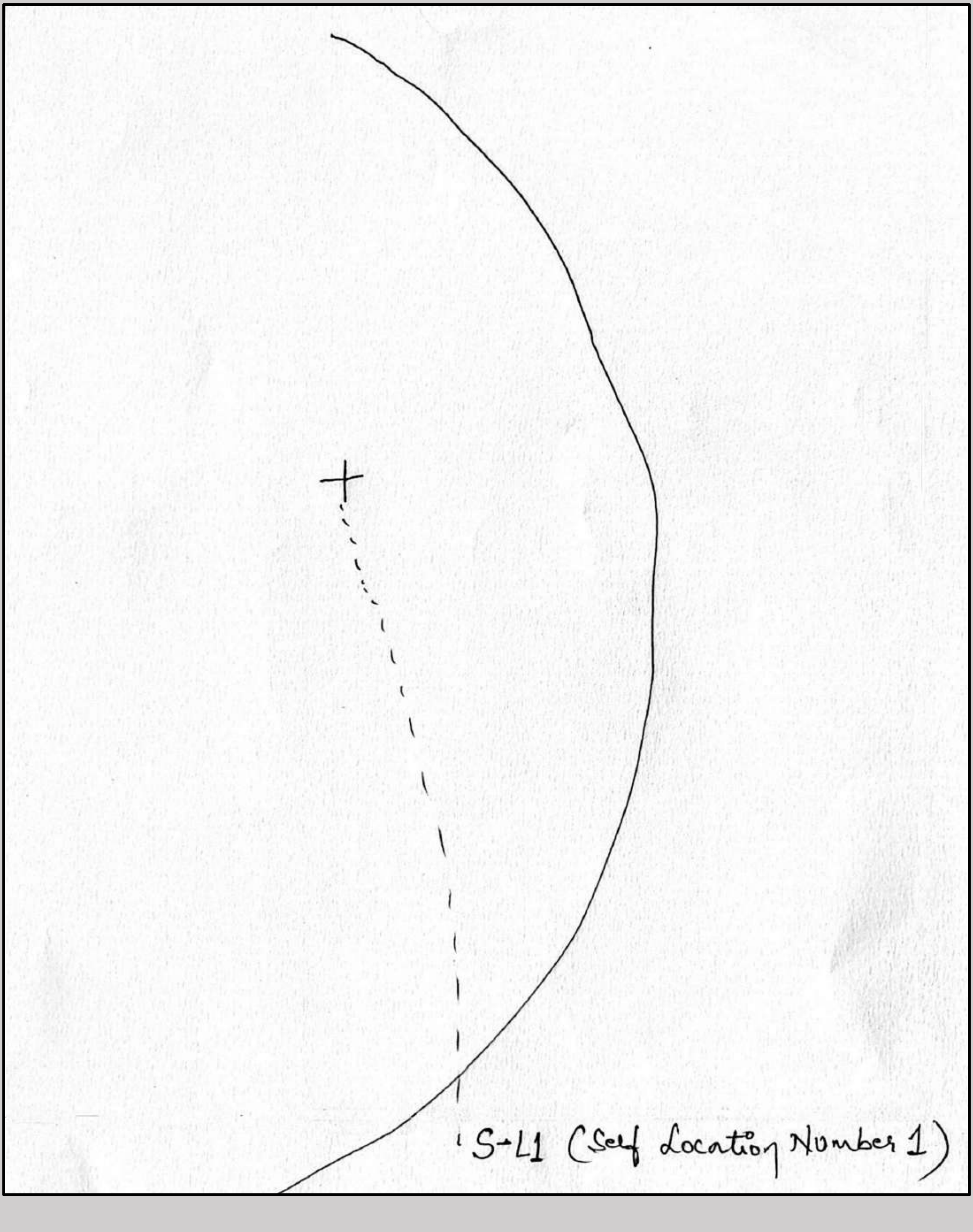

- C3

Subject 38

Sequence: part/space

1 NDE

- C4

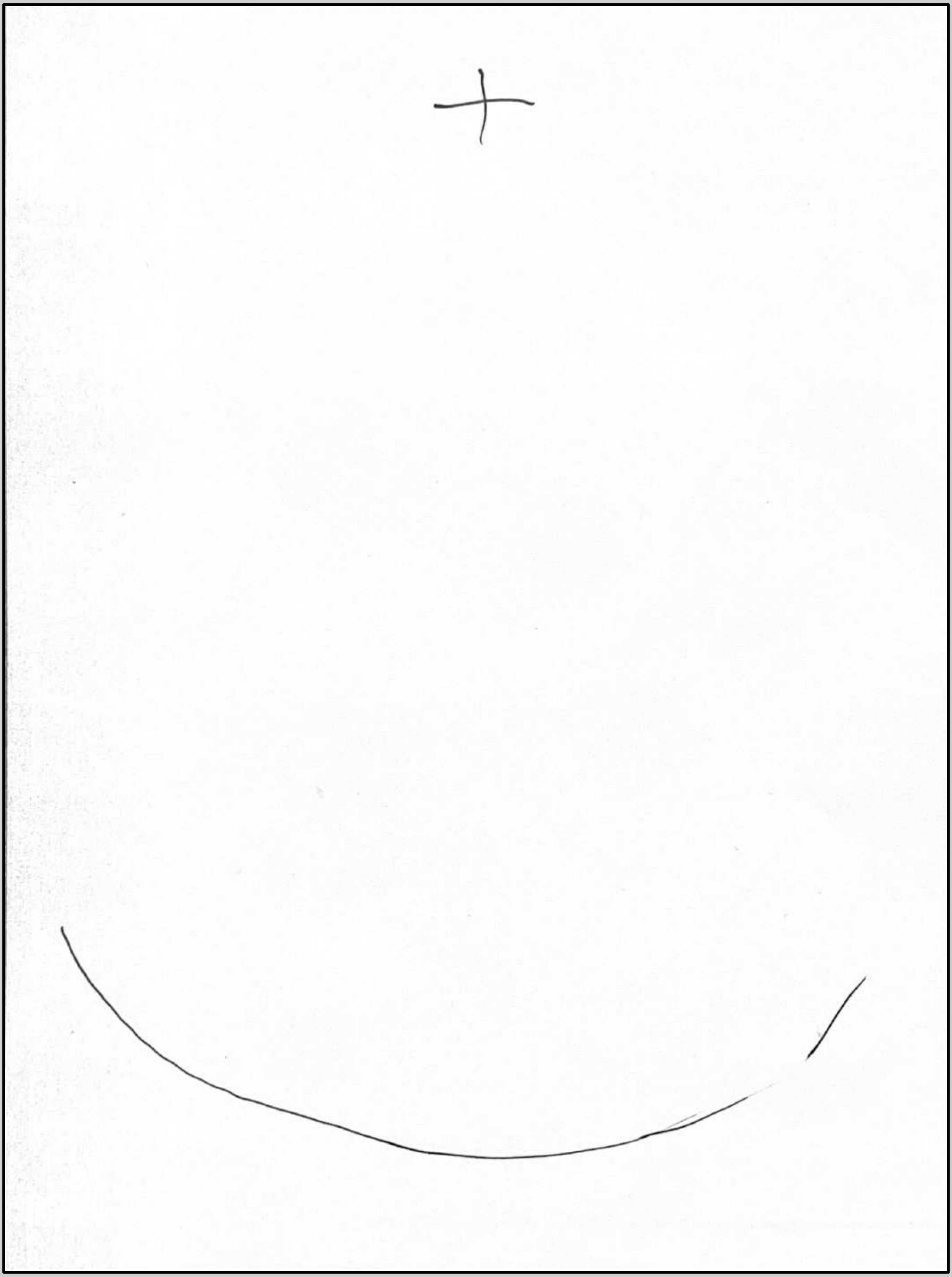

• C4

Subject 39

Sequence: part/space

2 OBE + 1 NDE

- OBE Dissociative
- C5
- OBE Associative

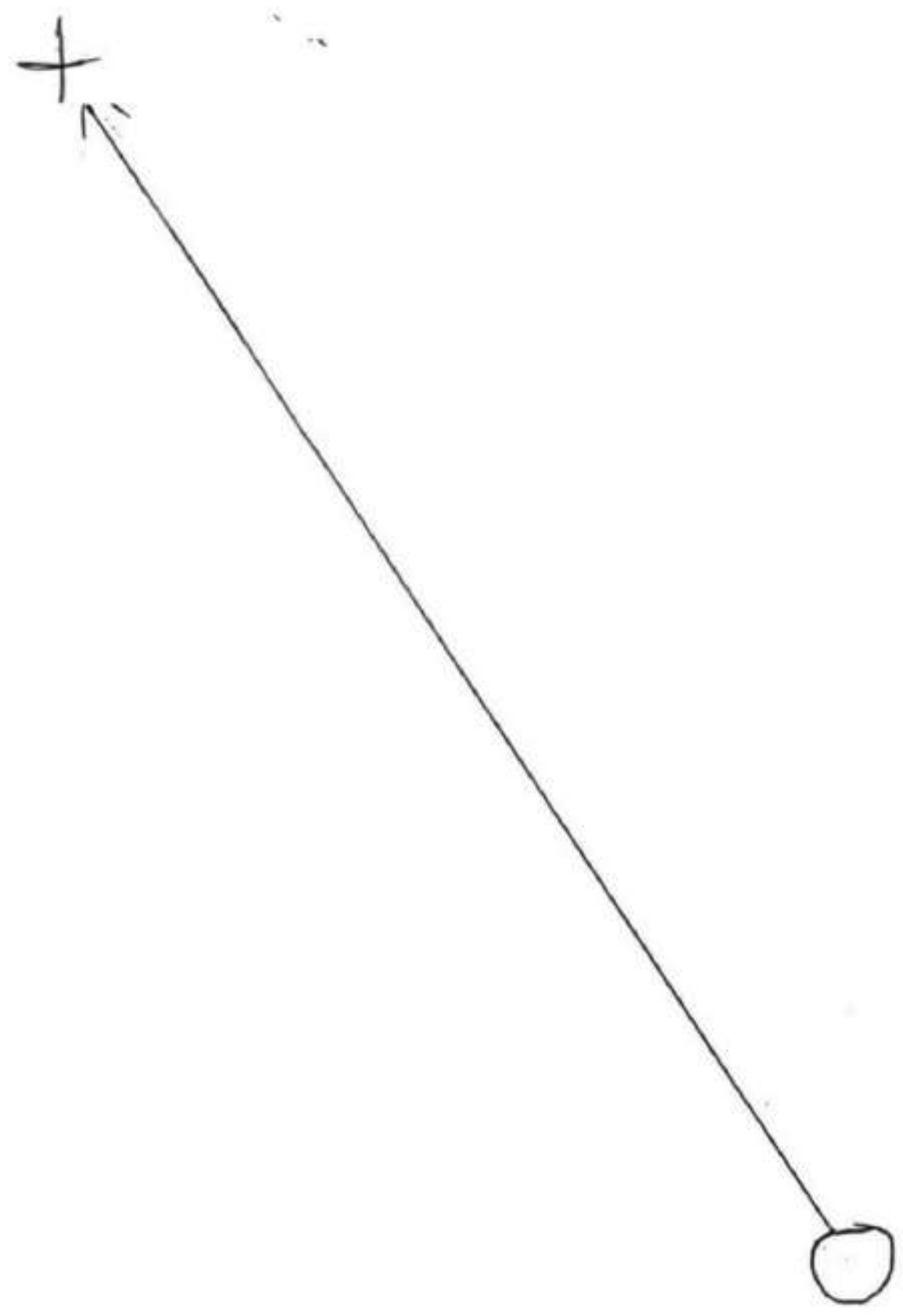

• OBE Dissociative

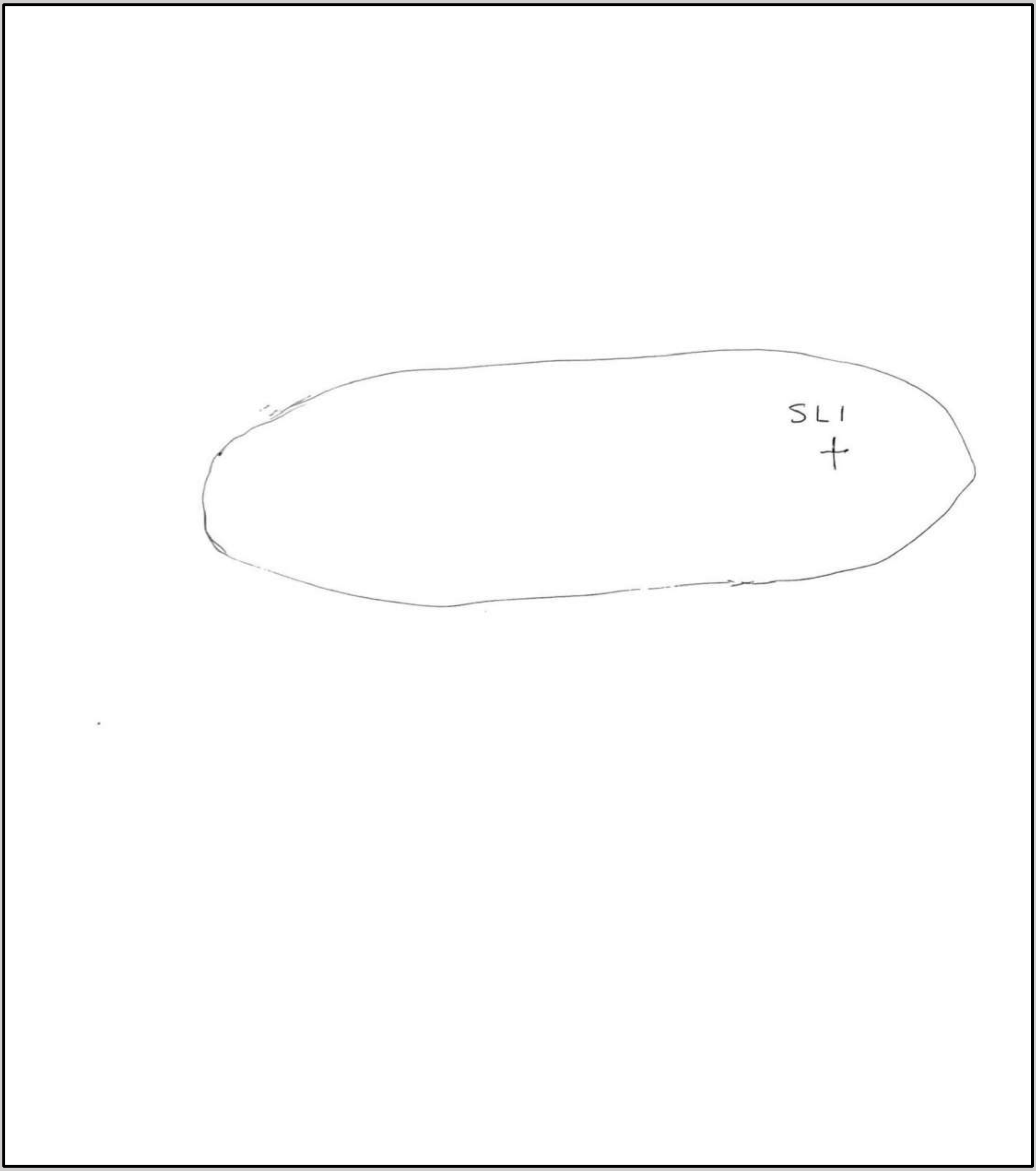

• C5

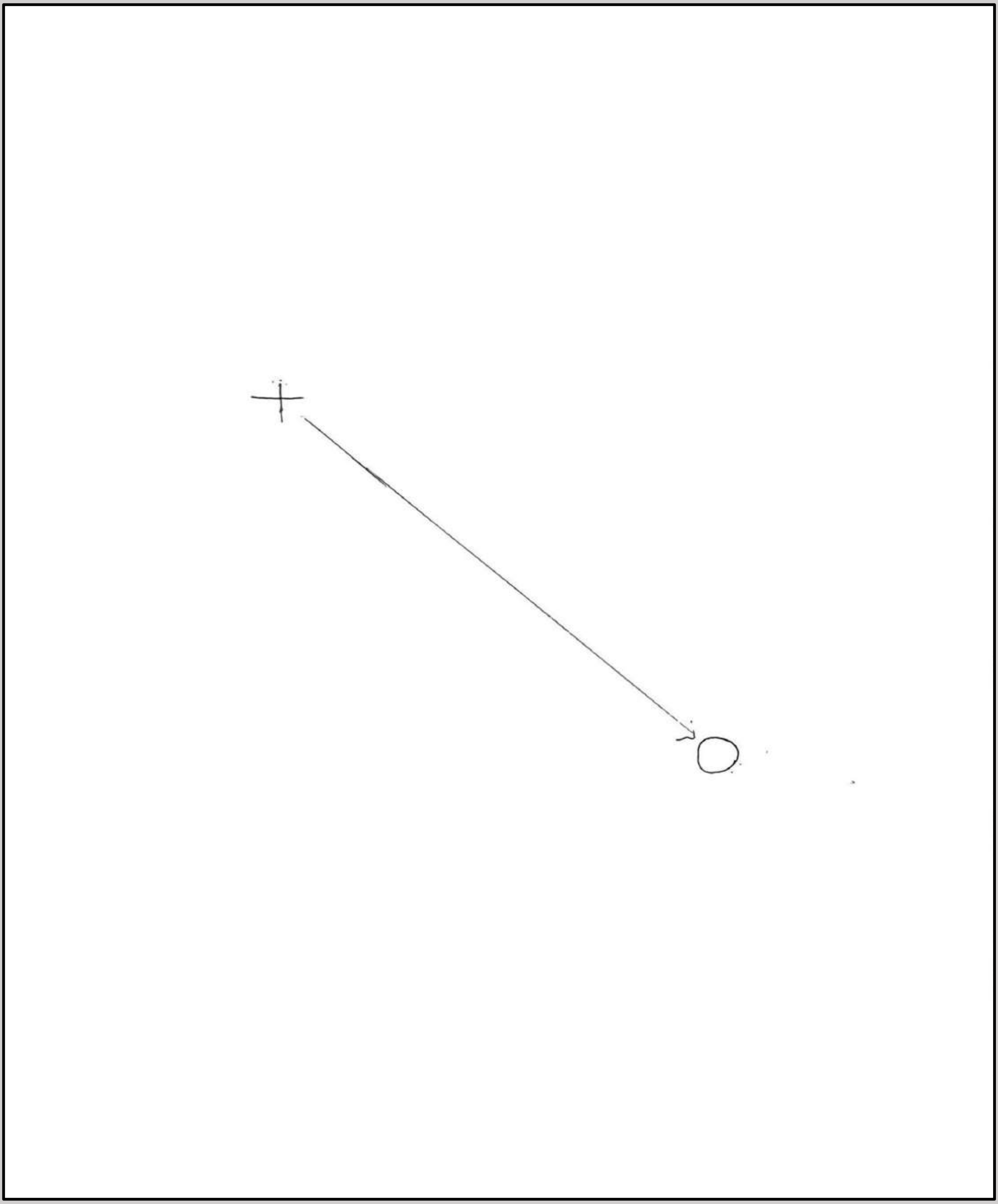

• OBE Associative

Subject 40

Sequence: part/space

1 OBE + 1 NDE

- C5
- OBE Associative

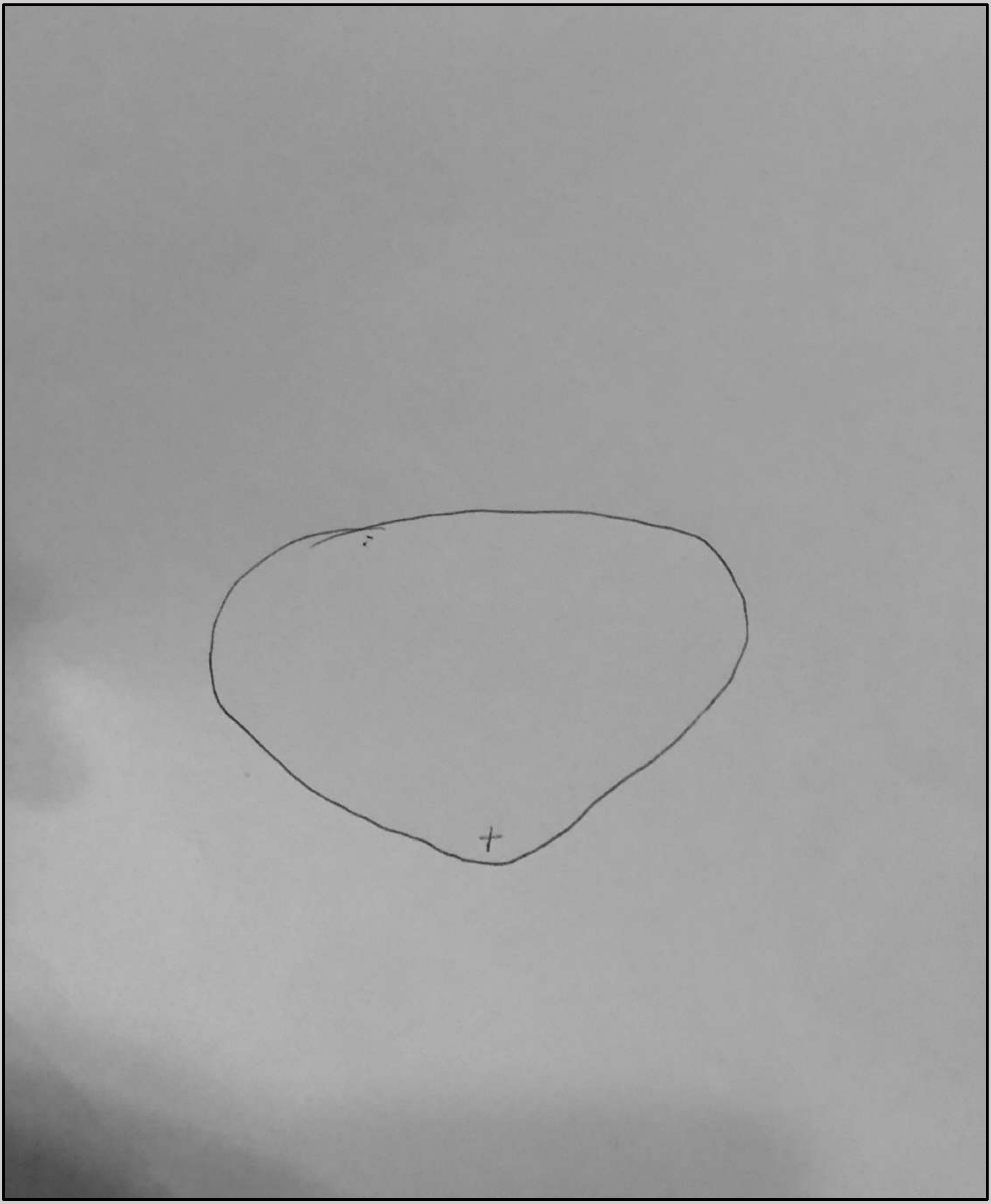

- C5

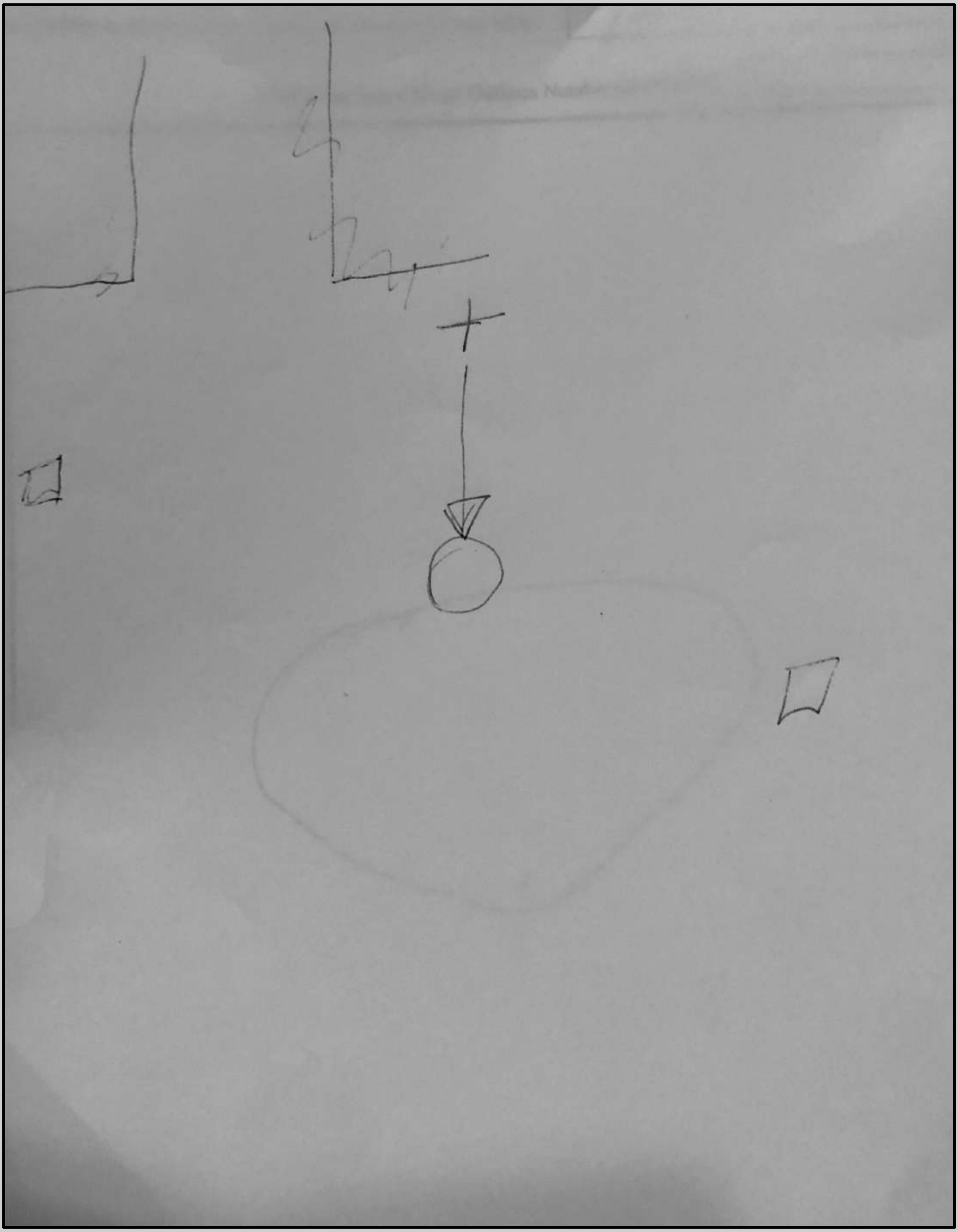

• OBE Associative

Subject 41

Sequence: part/space

1 OBE + 2 NDE

- OBE Dissociative
- C5
- C2

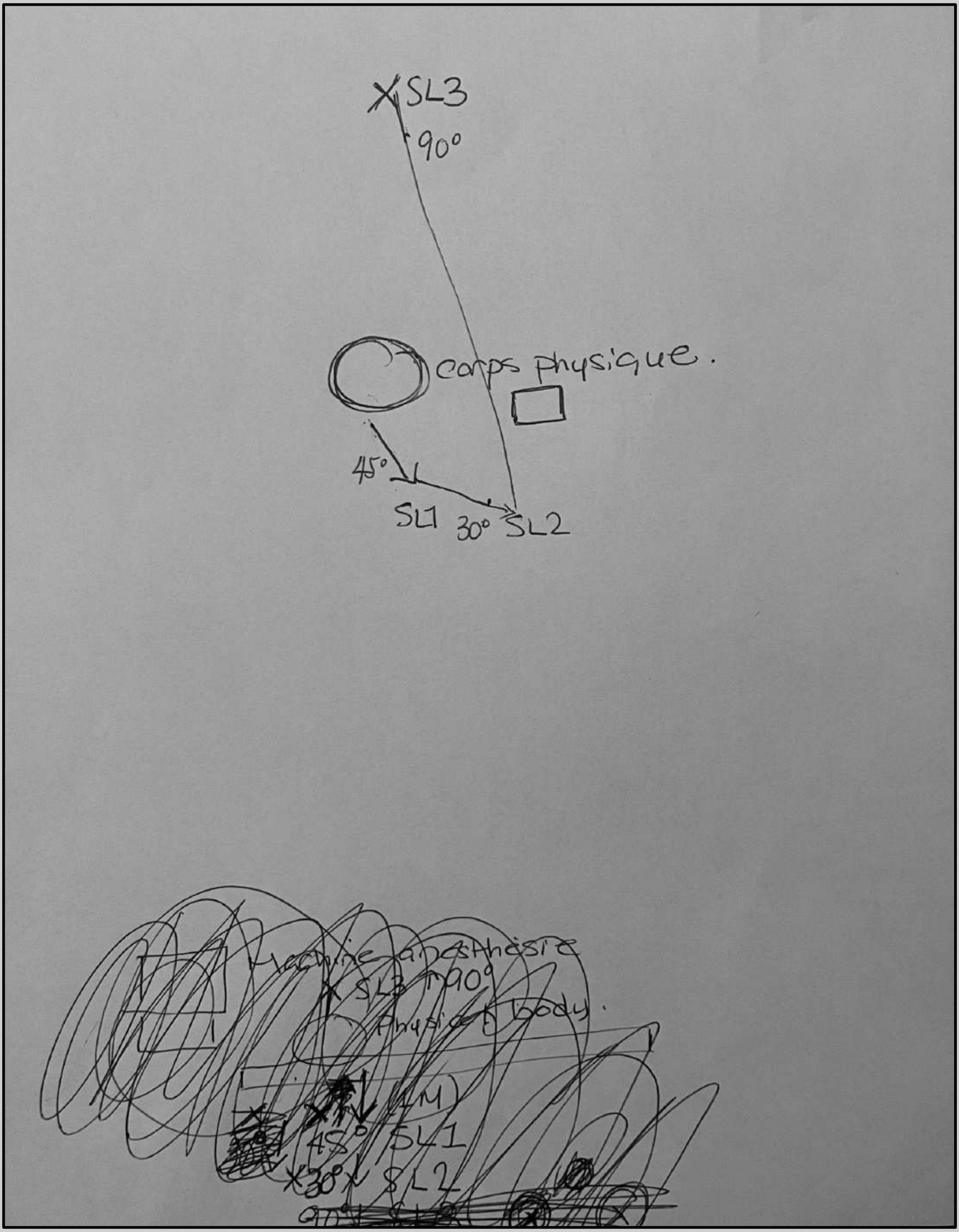

- OBE Dissociative

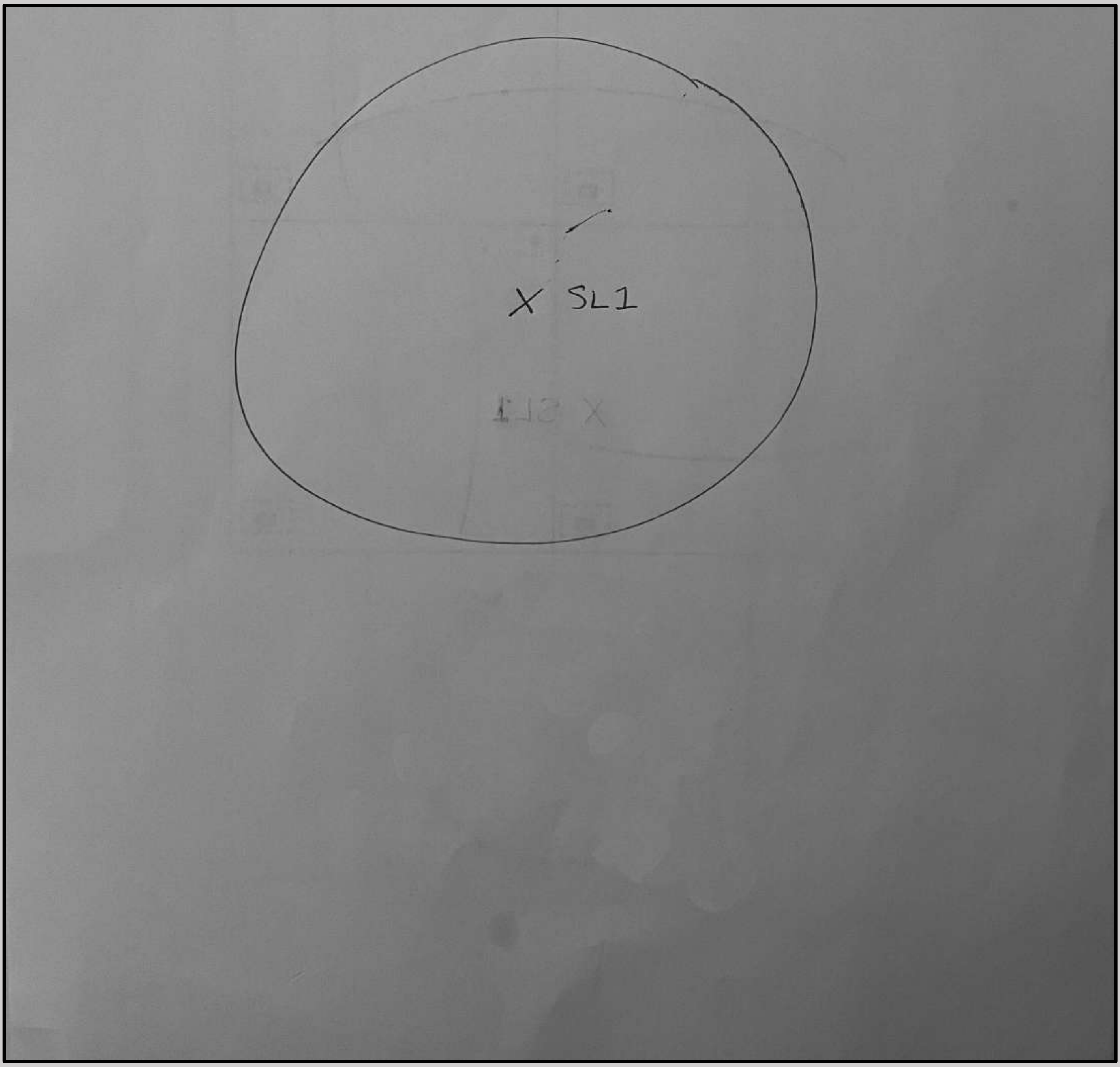

- C5

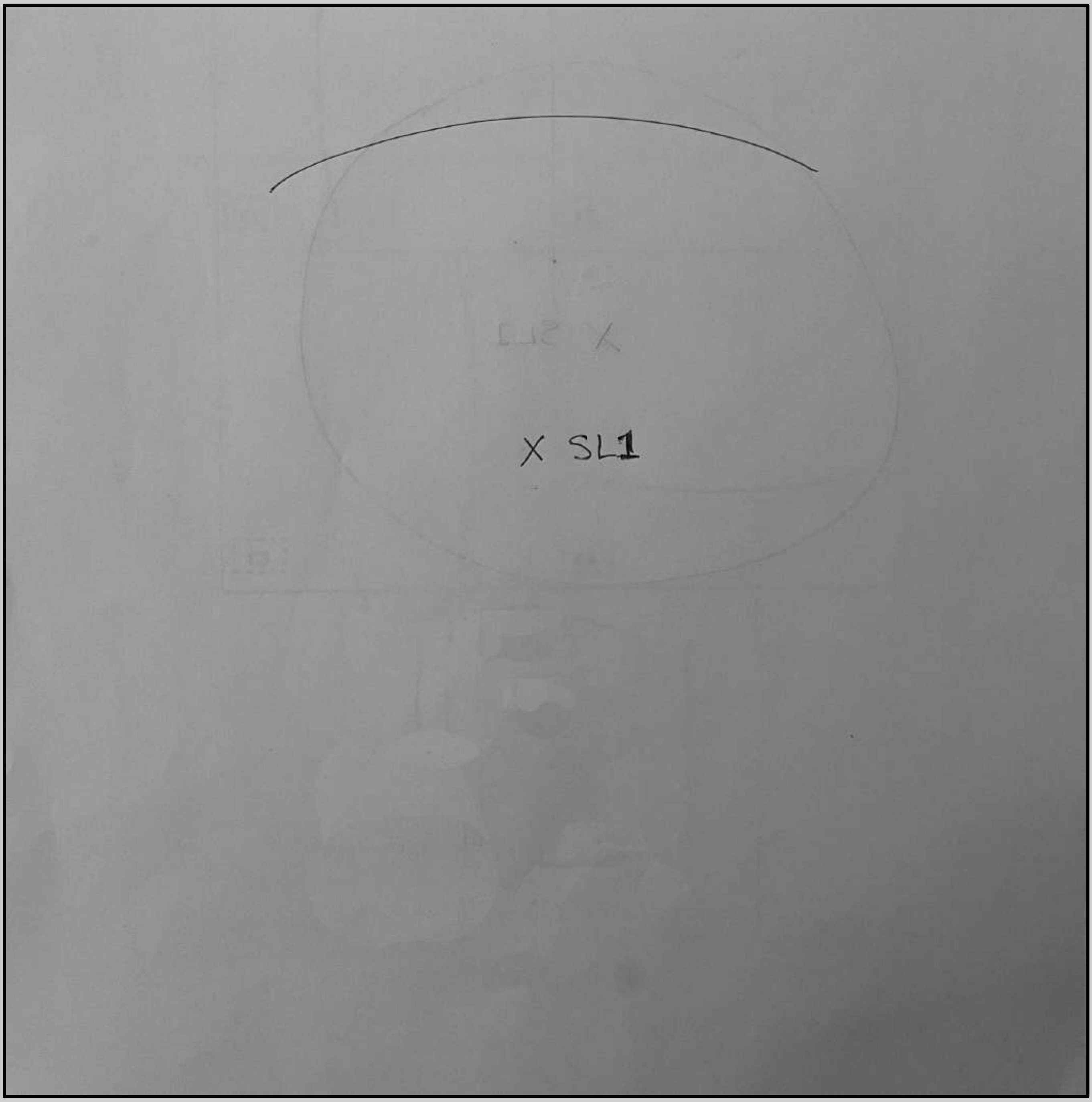

- C2

Subject 42

Sequence: part/space

2 OBE + 3 NDE

- OBE Dissociative
- C5
- A7
- C2
- OBE Associative

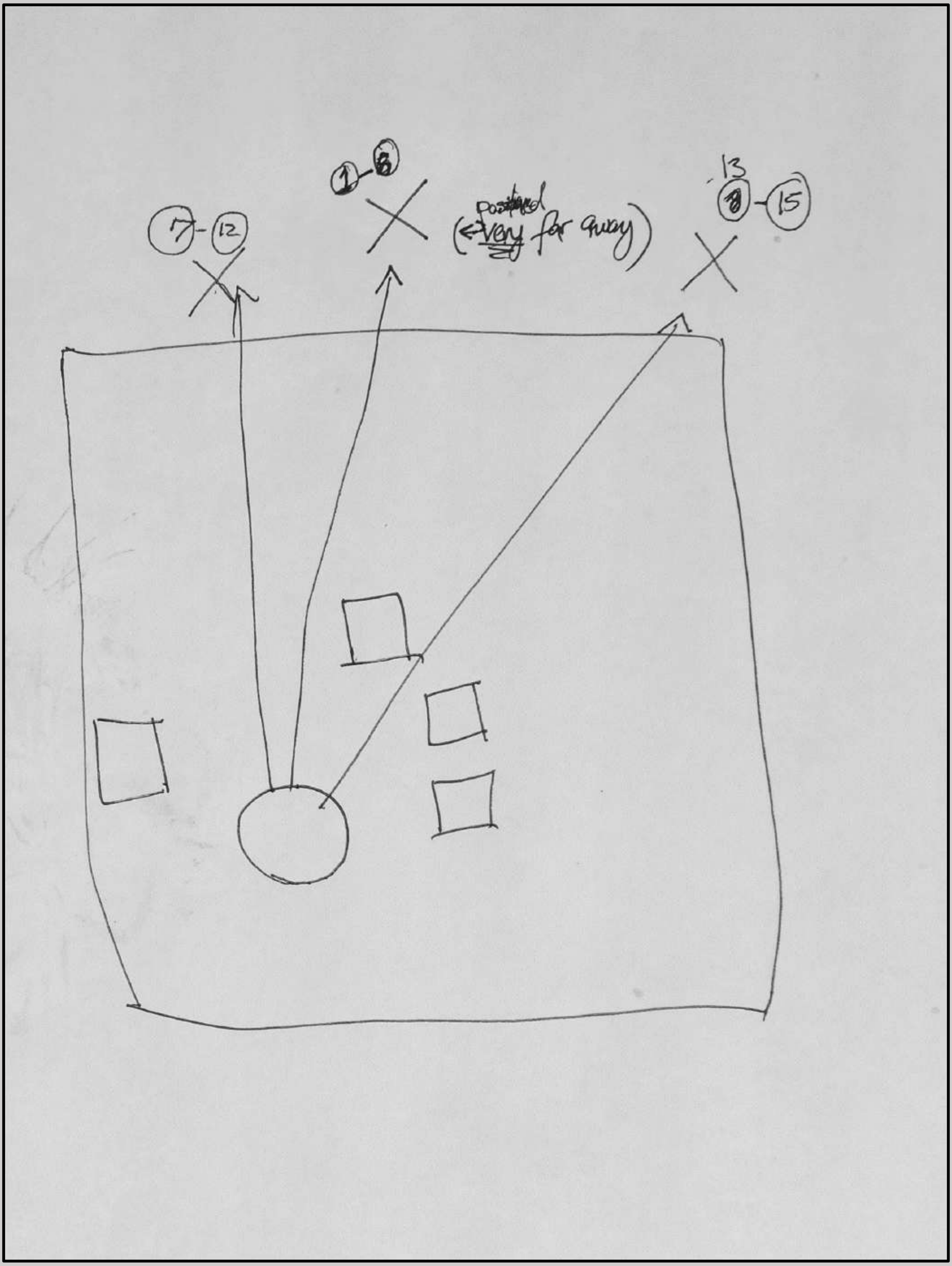

• OBE Dissociative

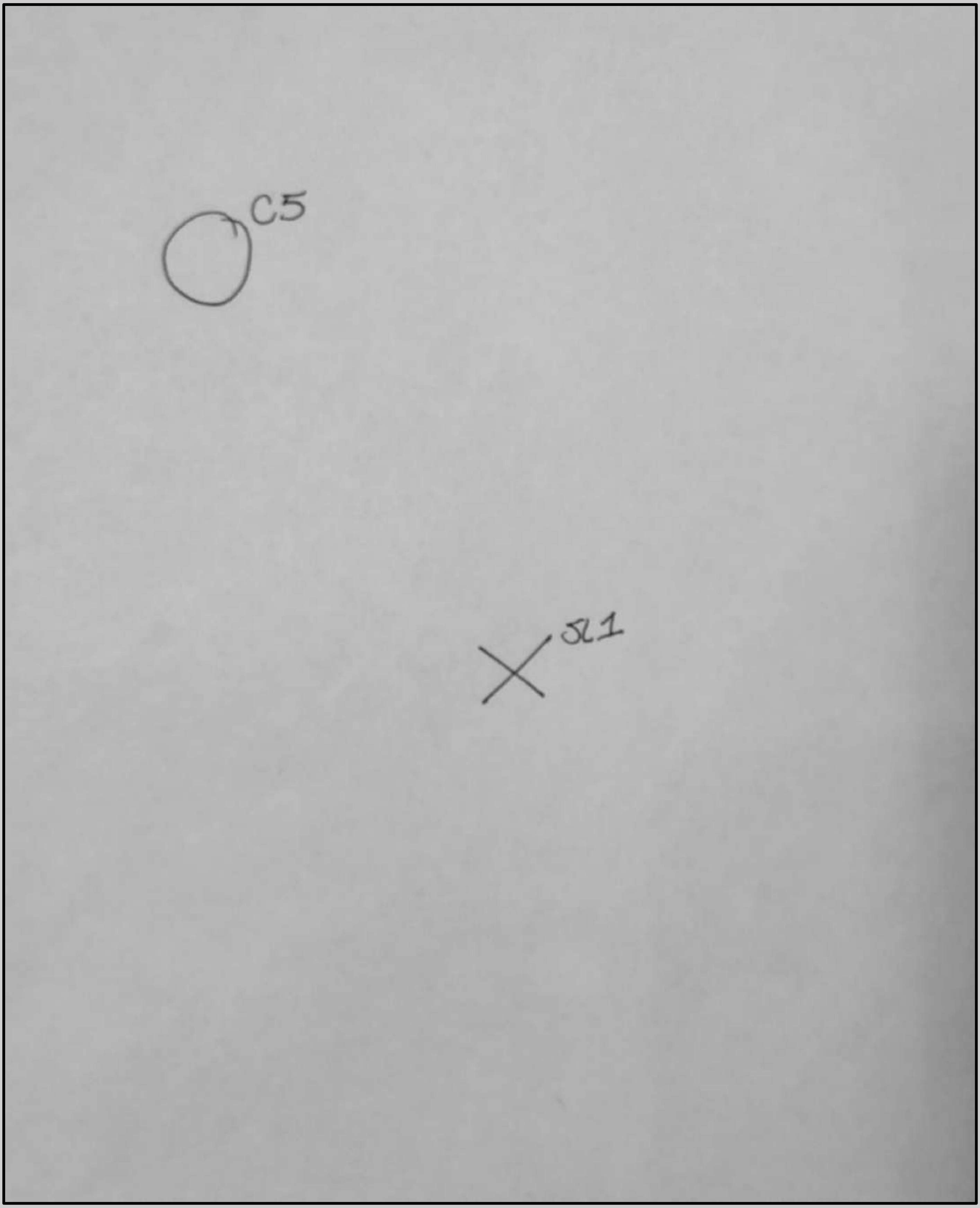

- C5

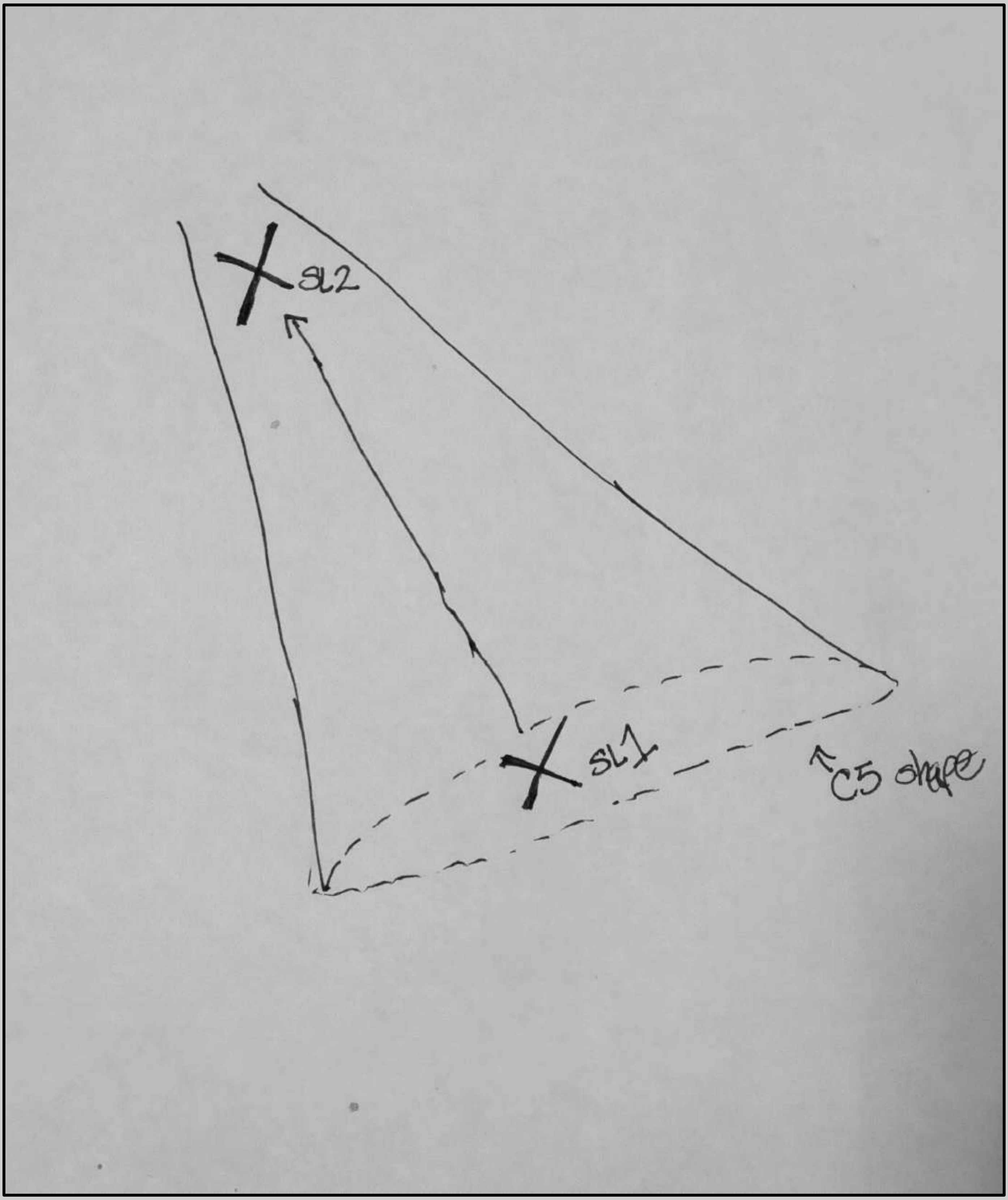

• A7

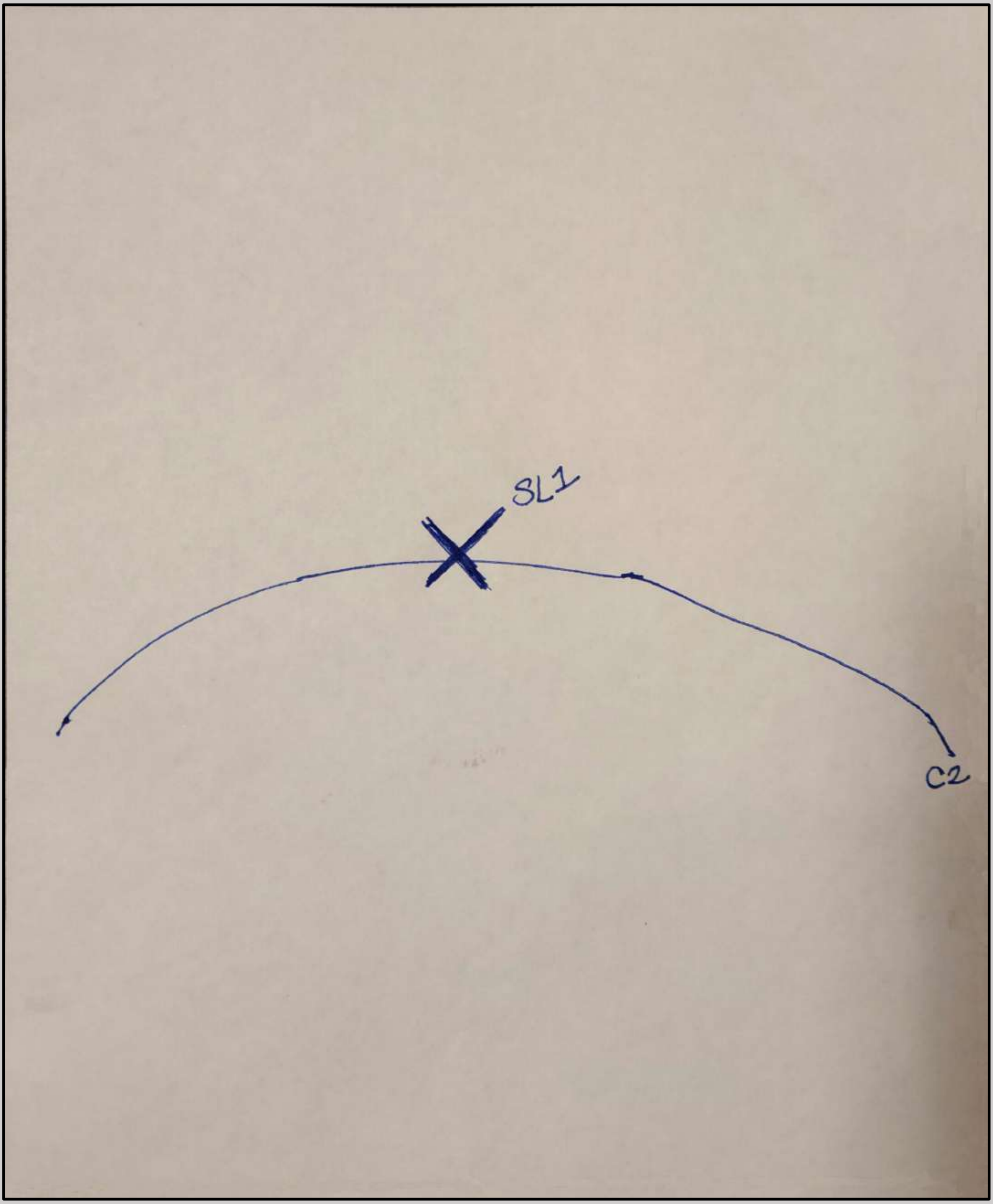

• C2

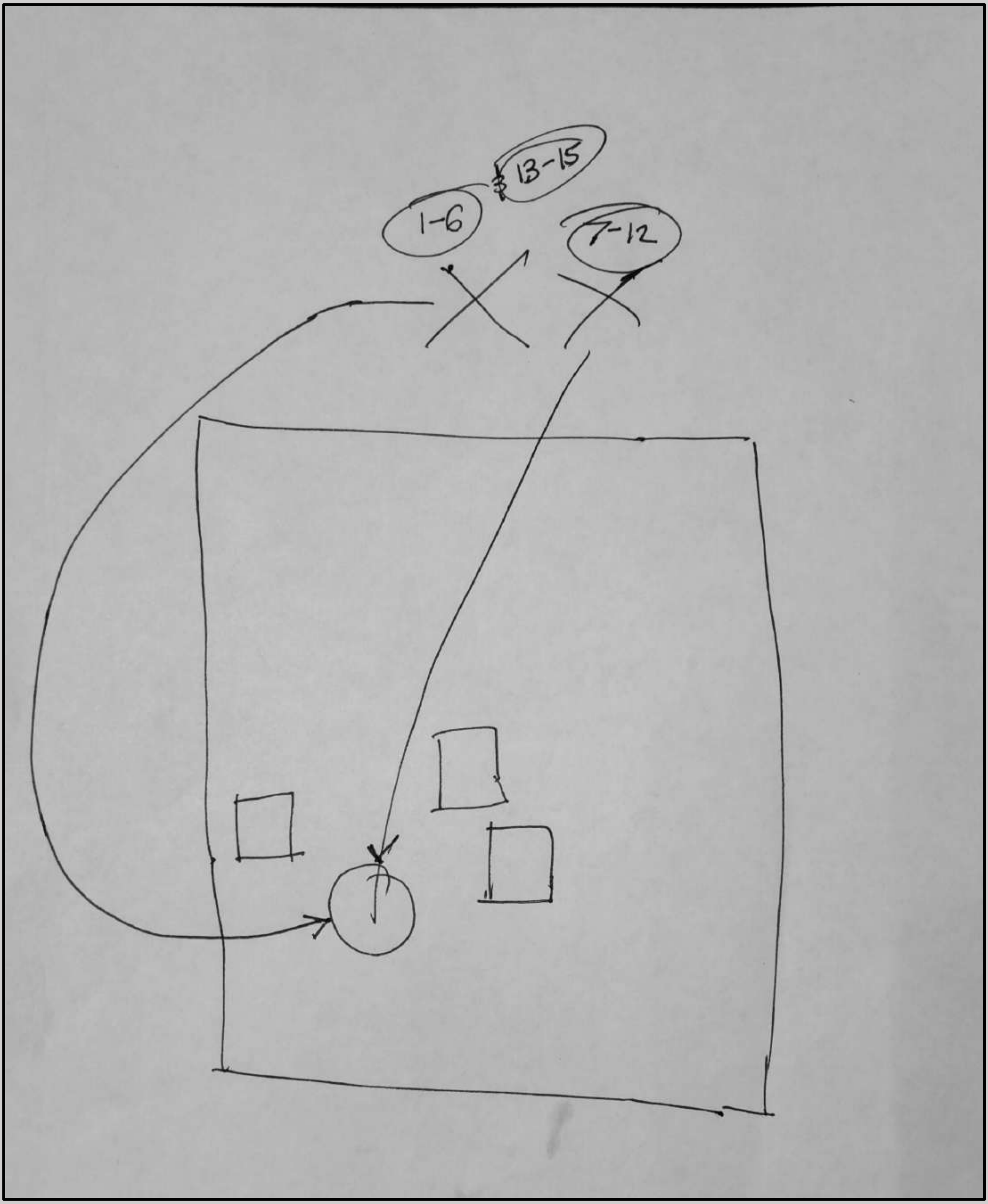

• OBE Associative

Subject 43

Sequence: part/space

2 NDE

- C5
- C5

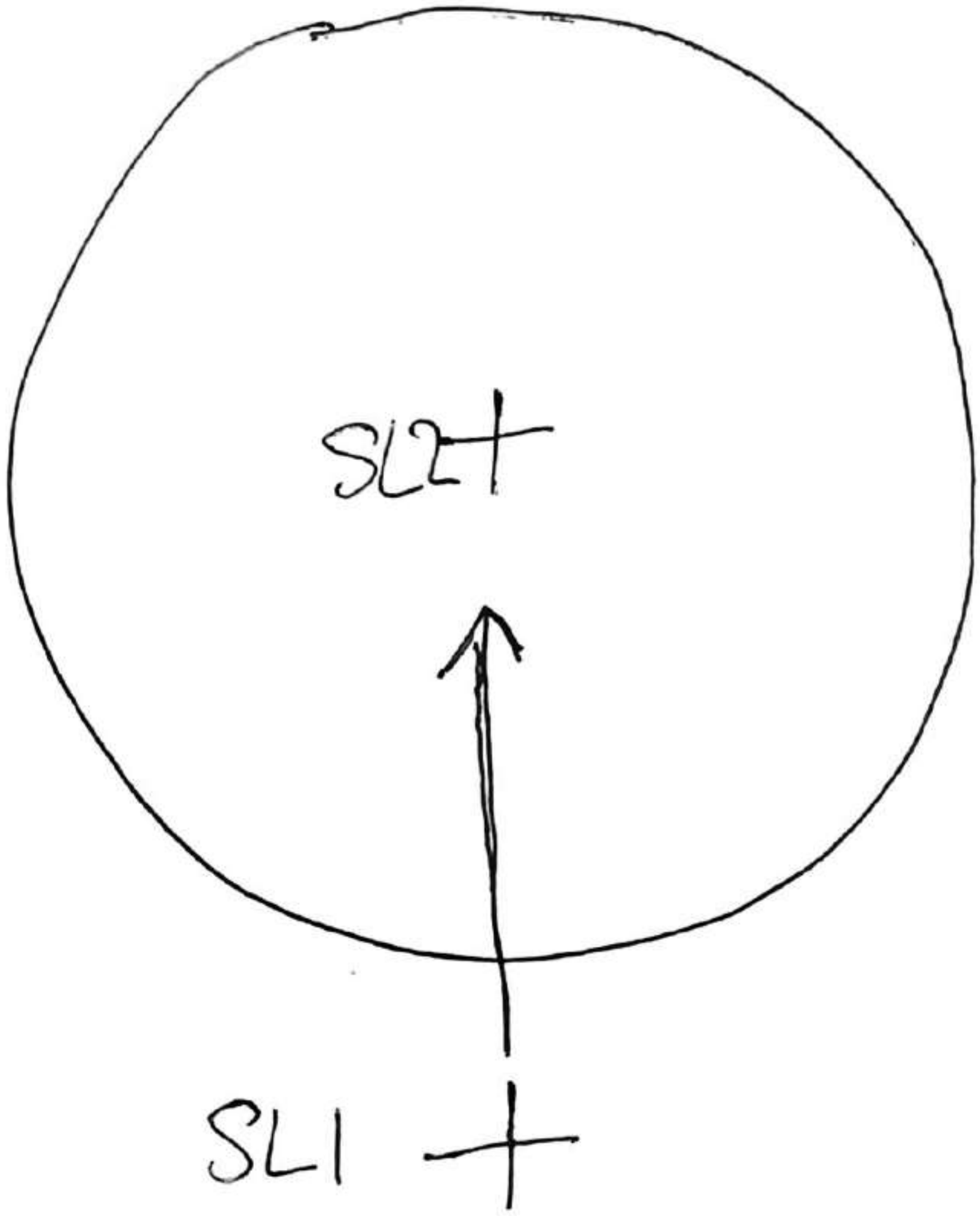

• C5

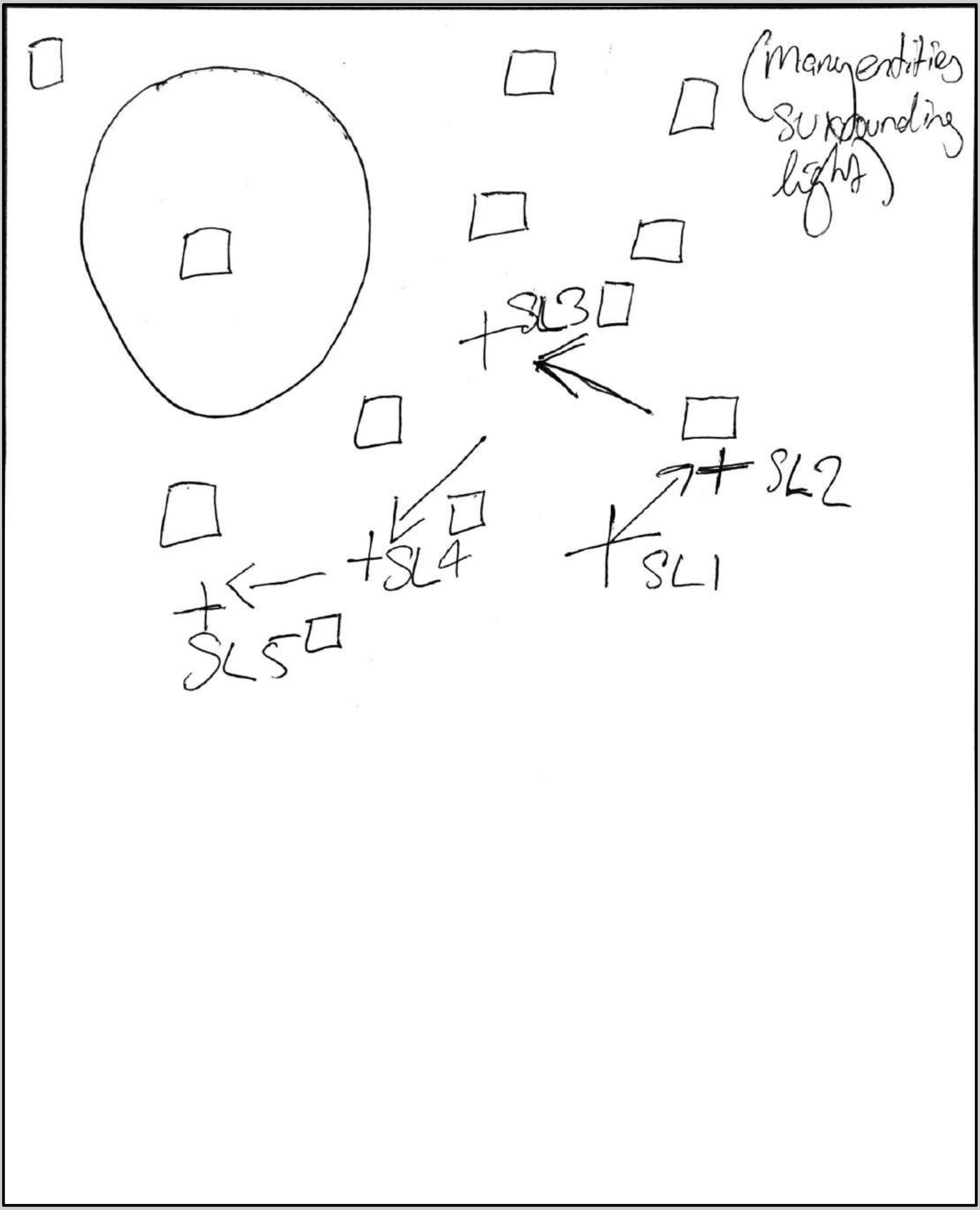

• C5

Subject 44

Sequence: part/space

2 OBE + 1 NDE

- OBE Dissociative
- C5
- OBE Associative

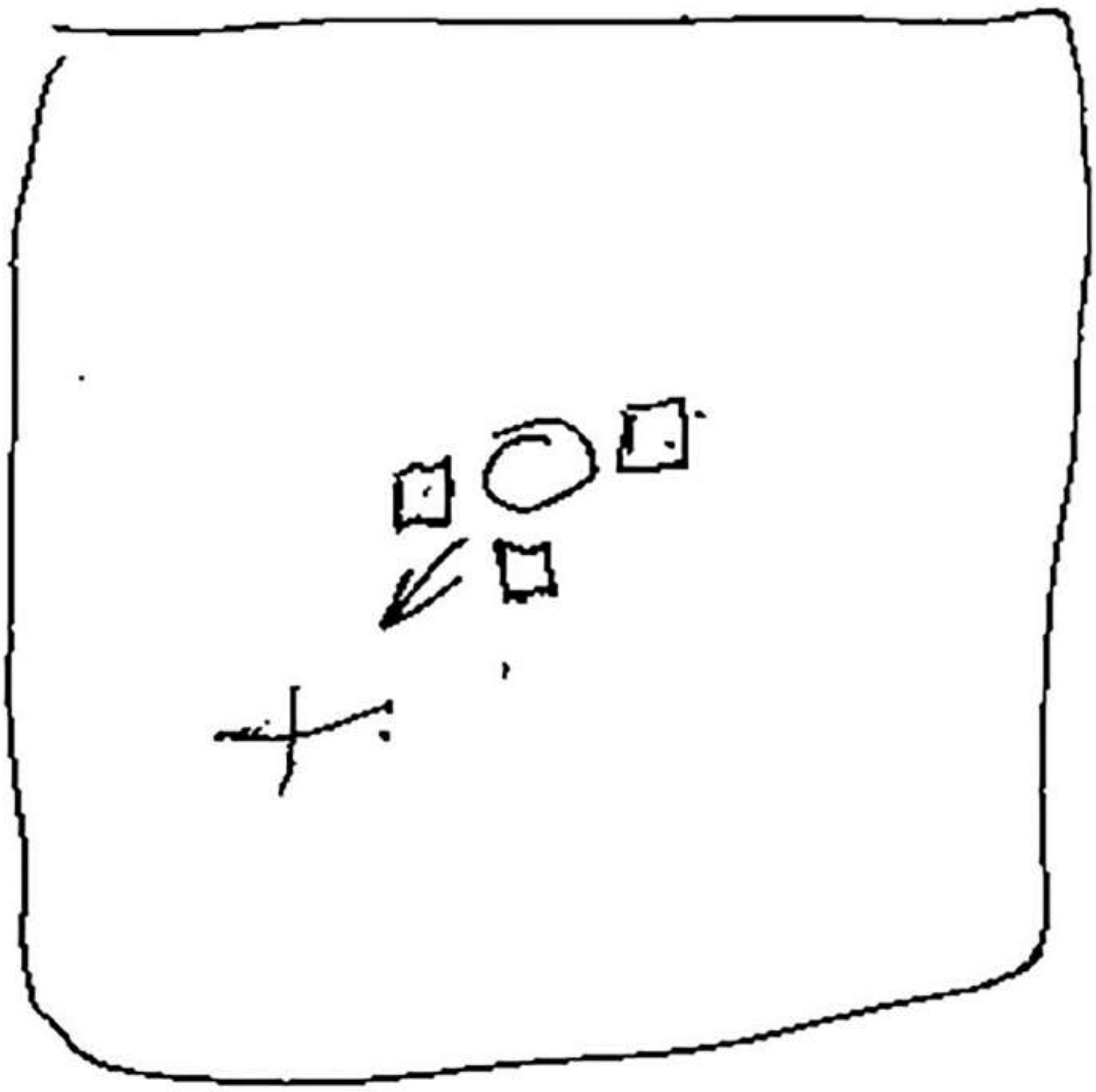

• OBE Dissociative

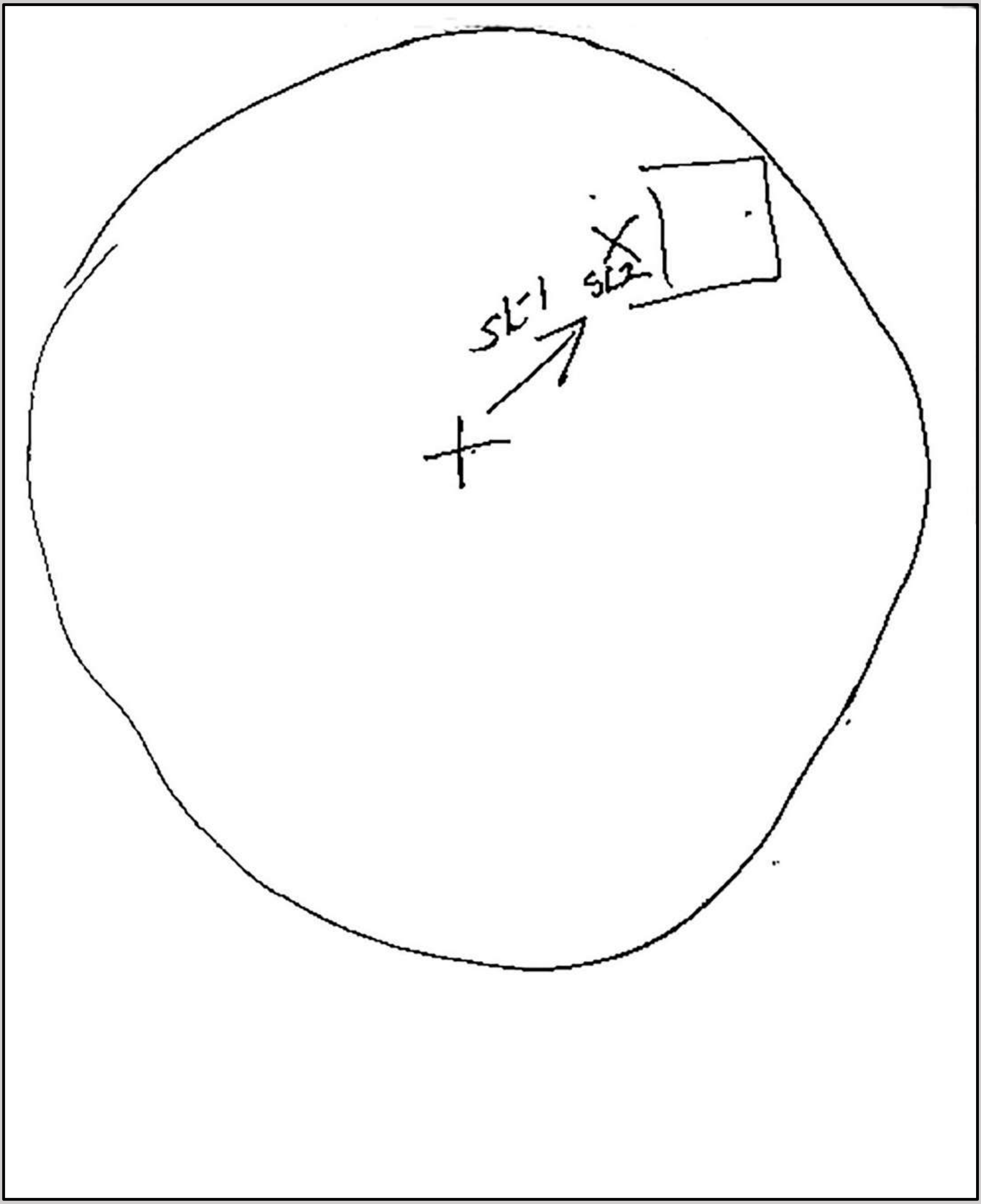

• C5

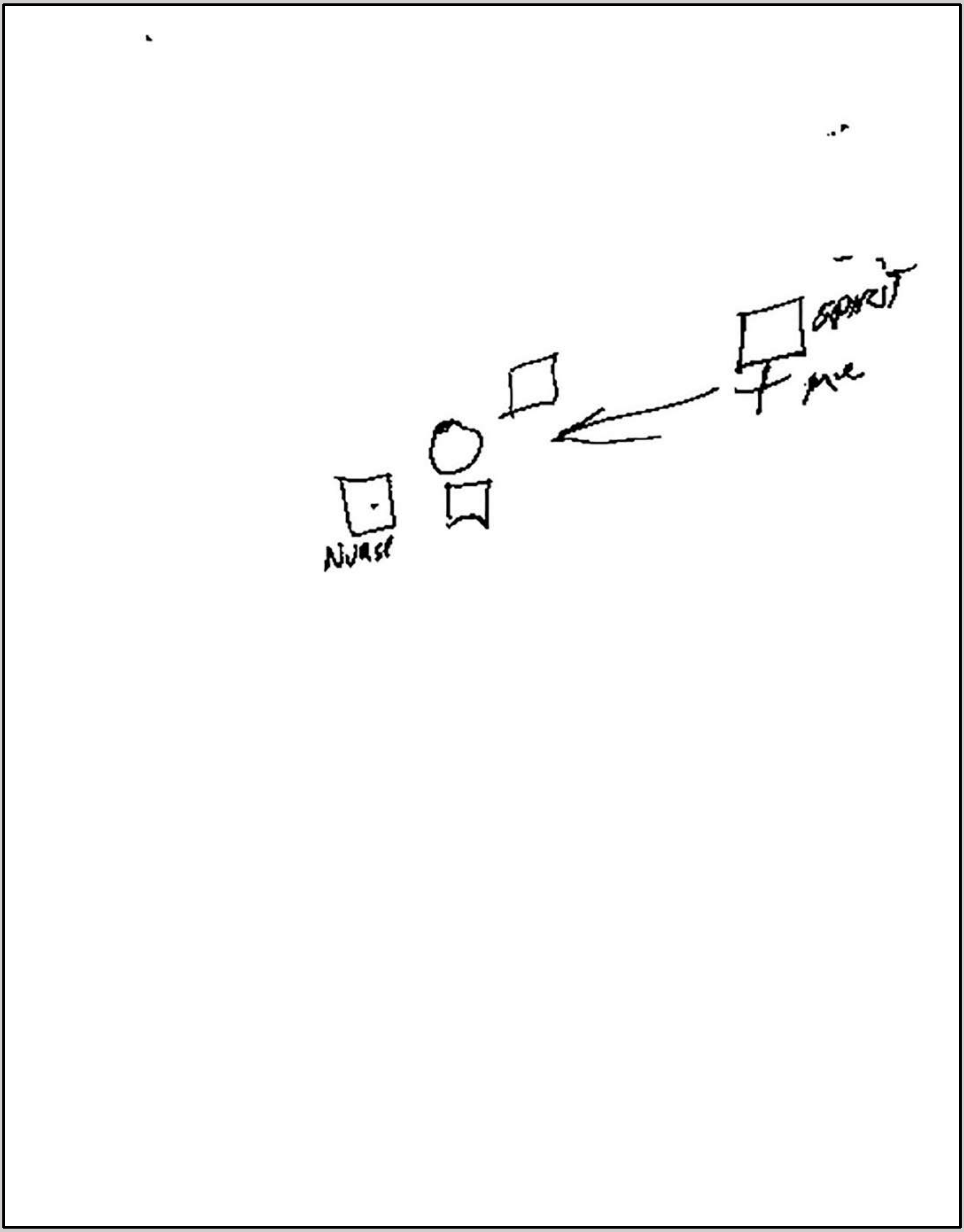

- OBE Associative

Subject 45

Sequence: part/space

1 NDE

- C5

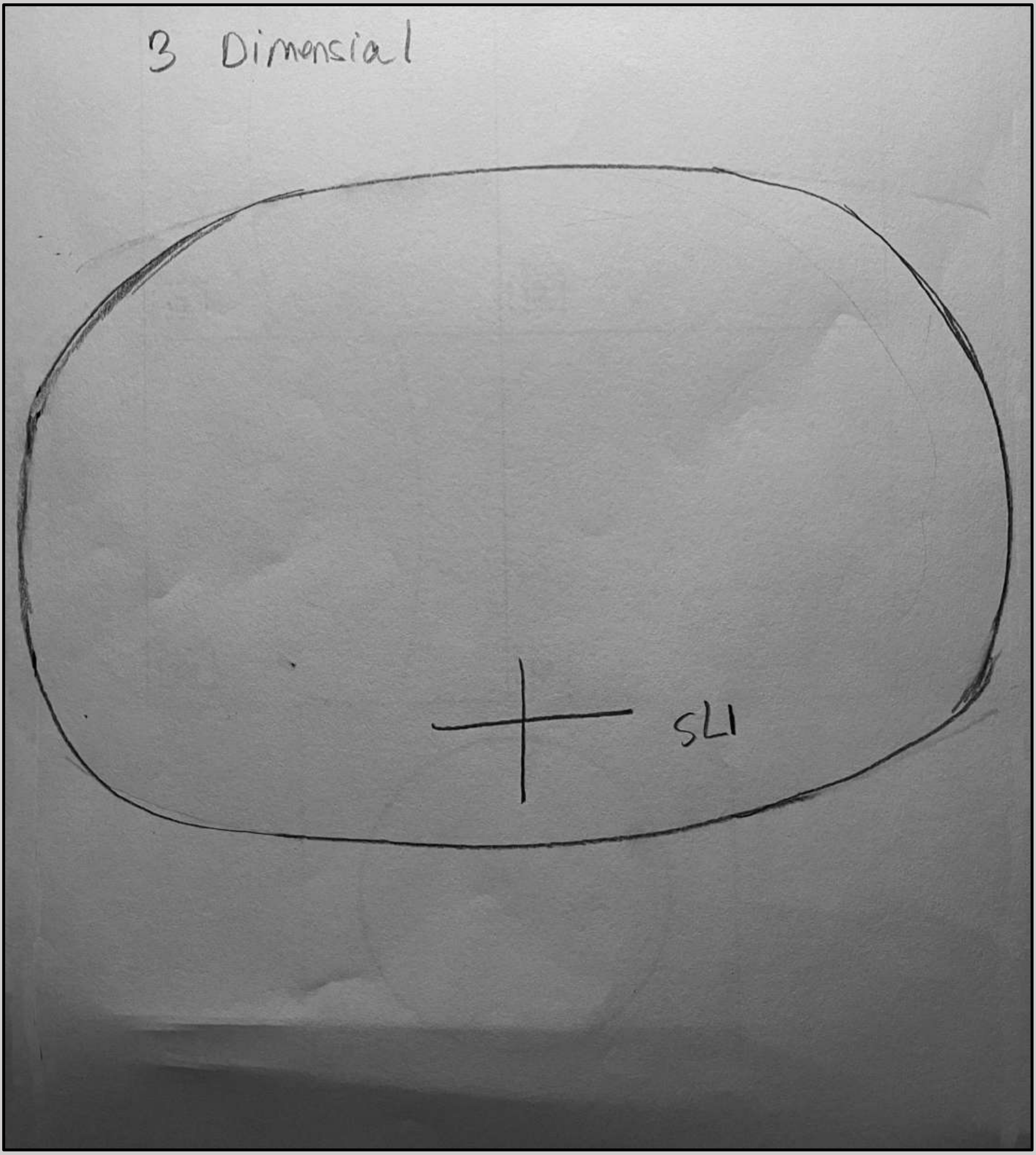

• C5

Subject 46

Sequence: part/space

1 OBE + 2 NDE

- C5
- C5
- OBE Associative

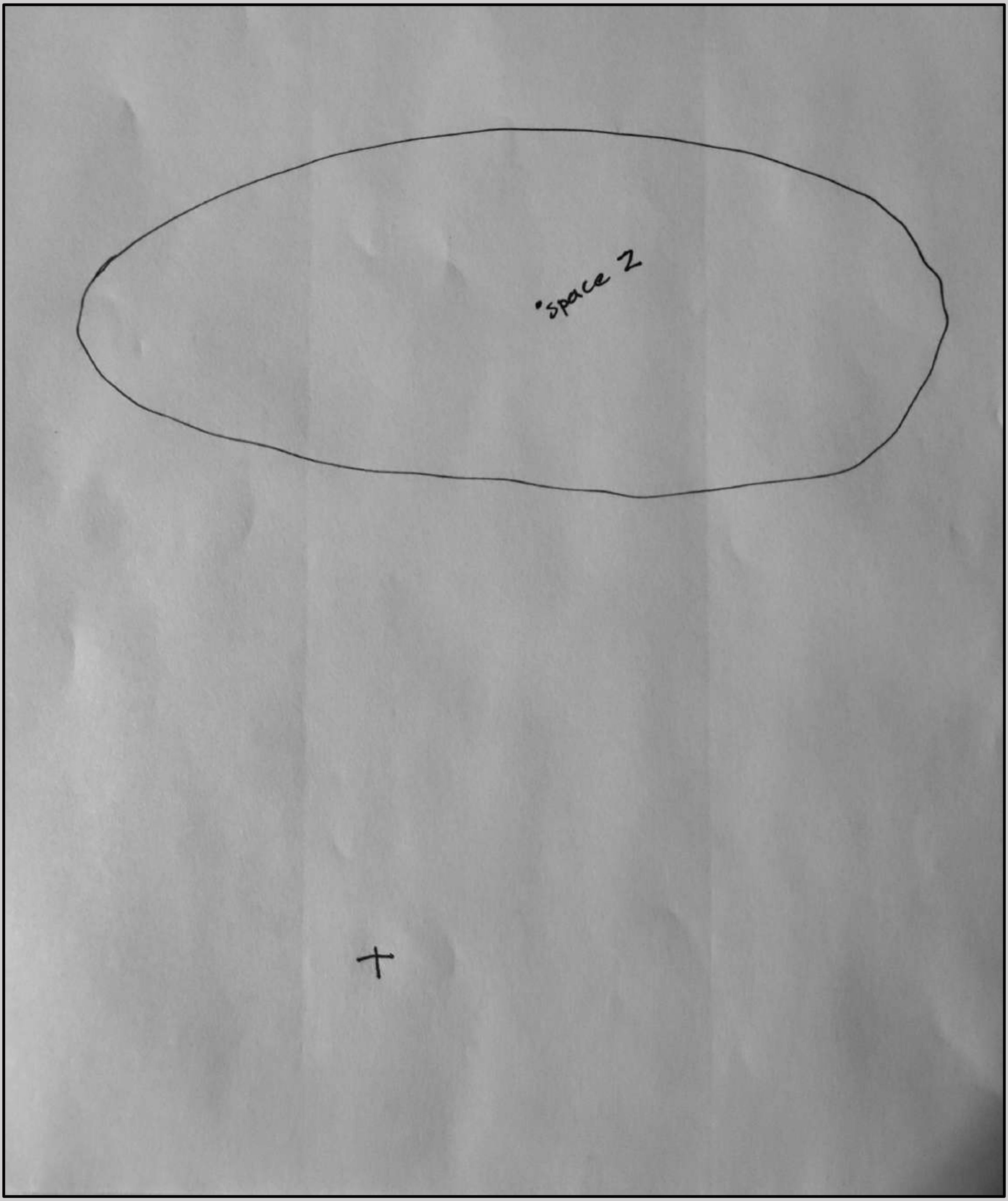

• C5

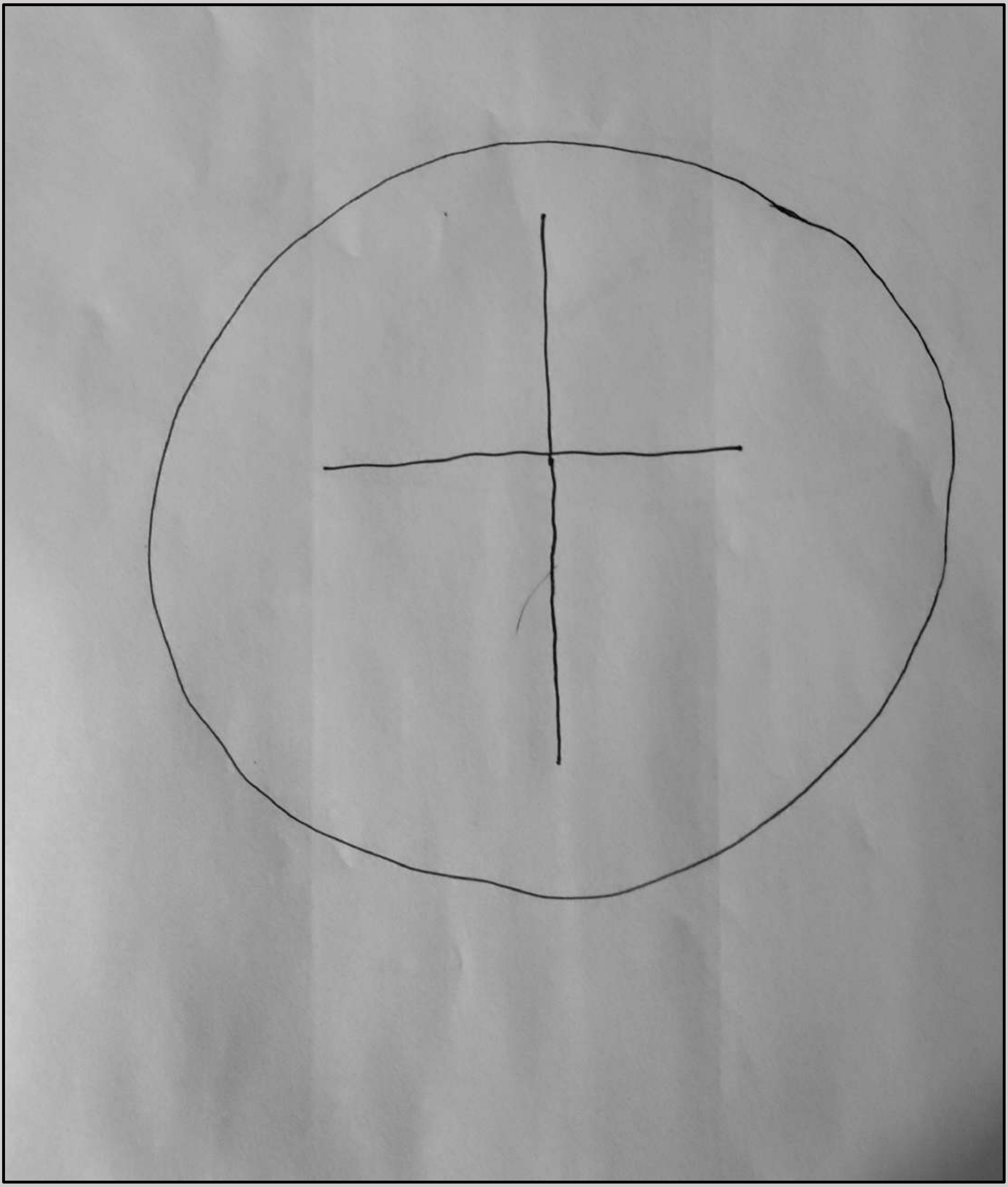

• C5

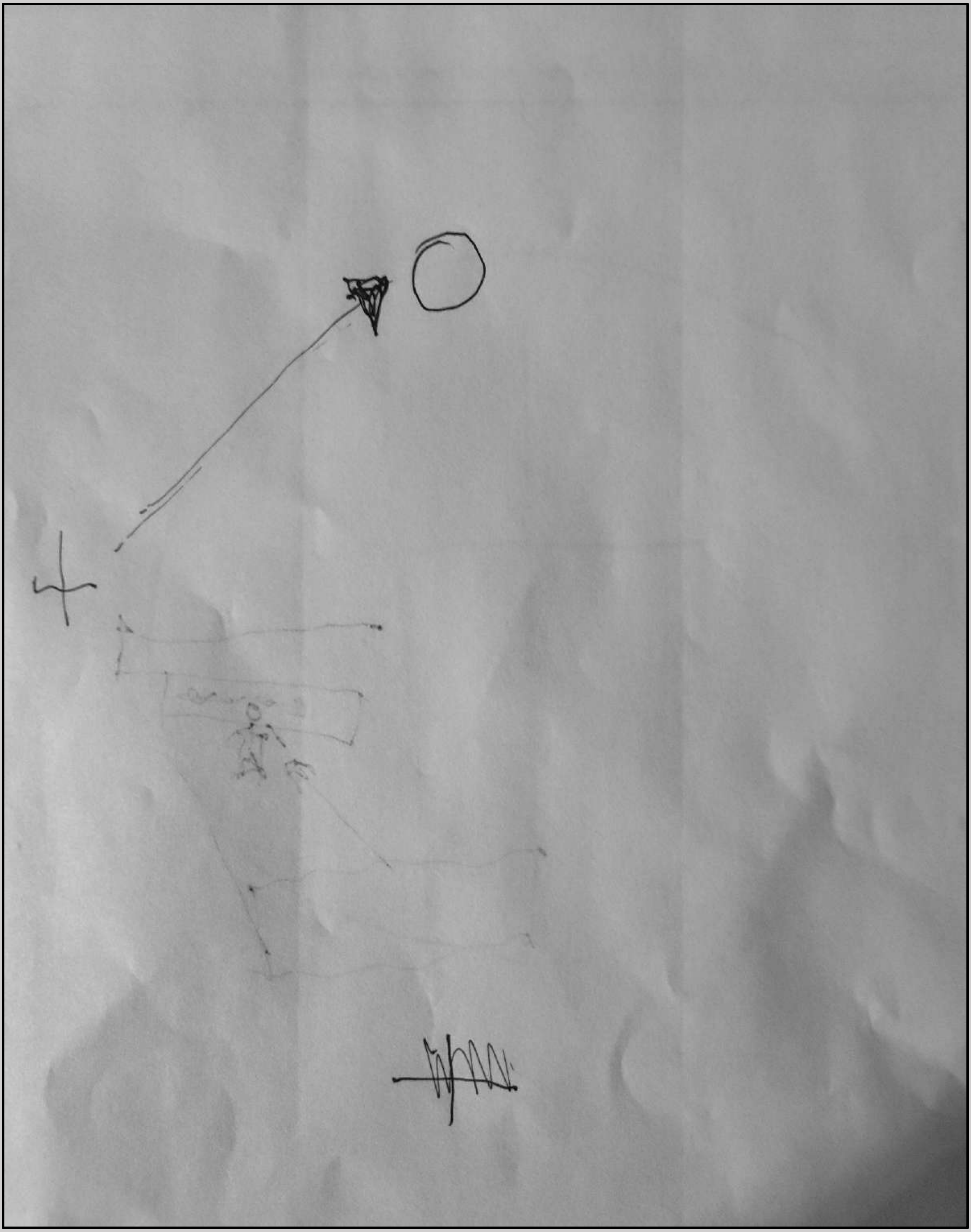

- OBE Associative

Subject 47

Sequence: part/space

1 OBE + 5 NDE

- OBE Dissociative
- C5
- B9
- B10
- B11
- B12

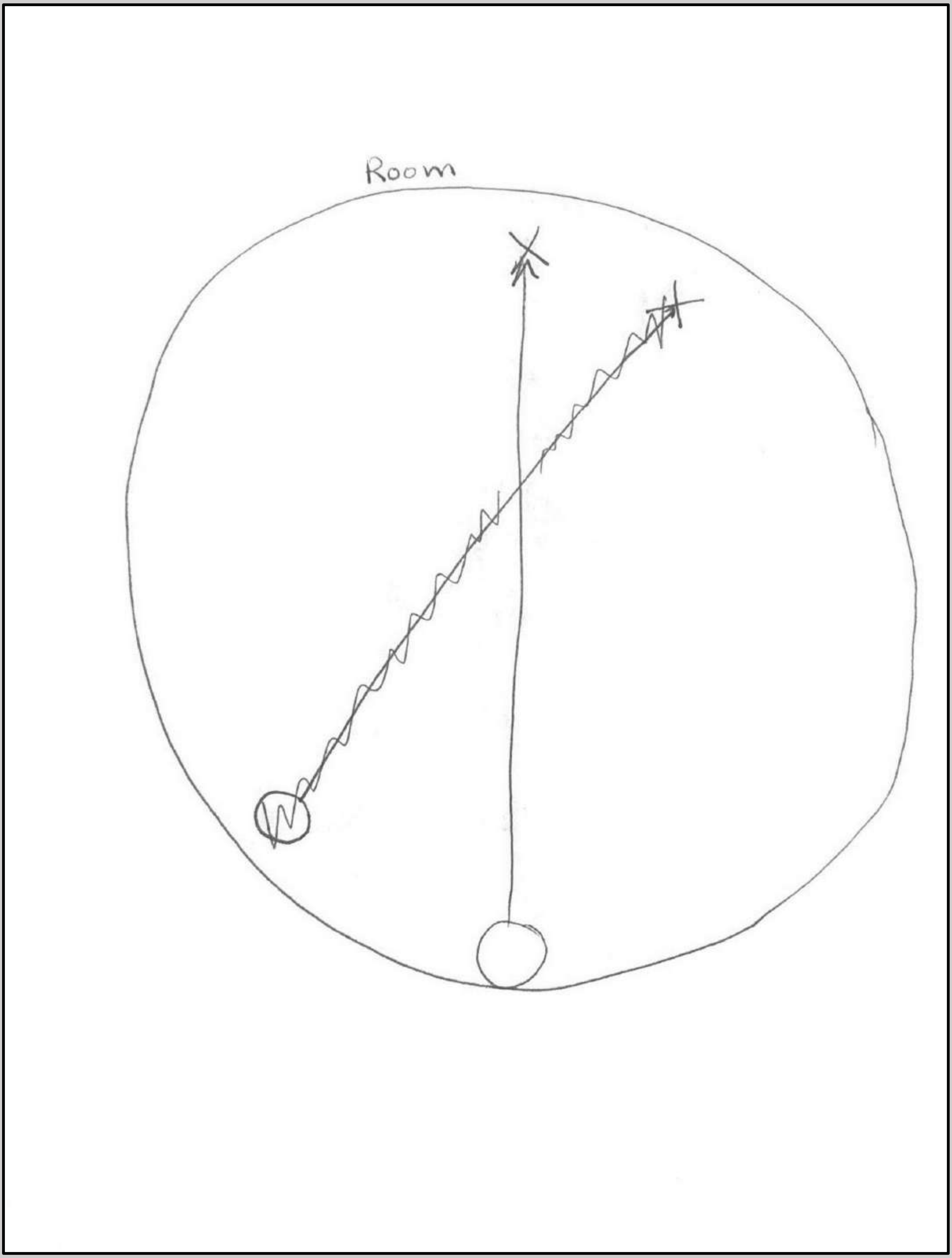

• OBE Dissociative

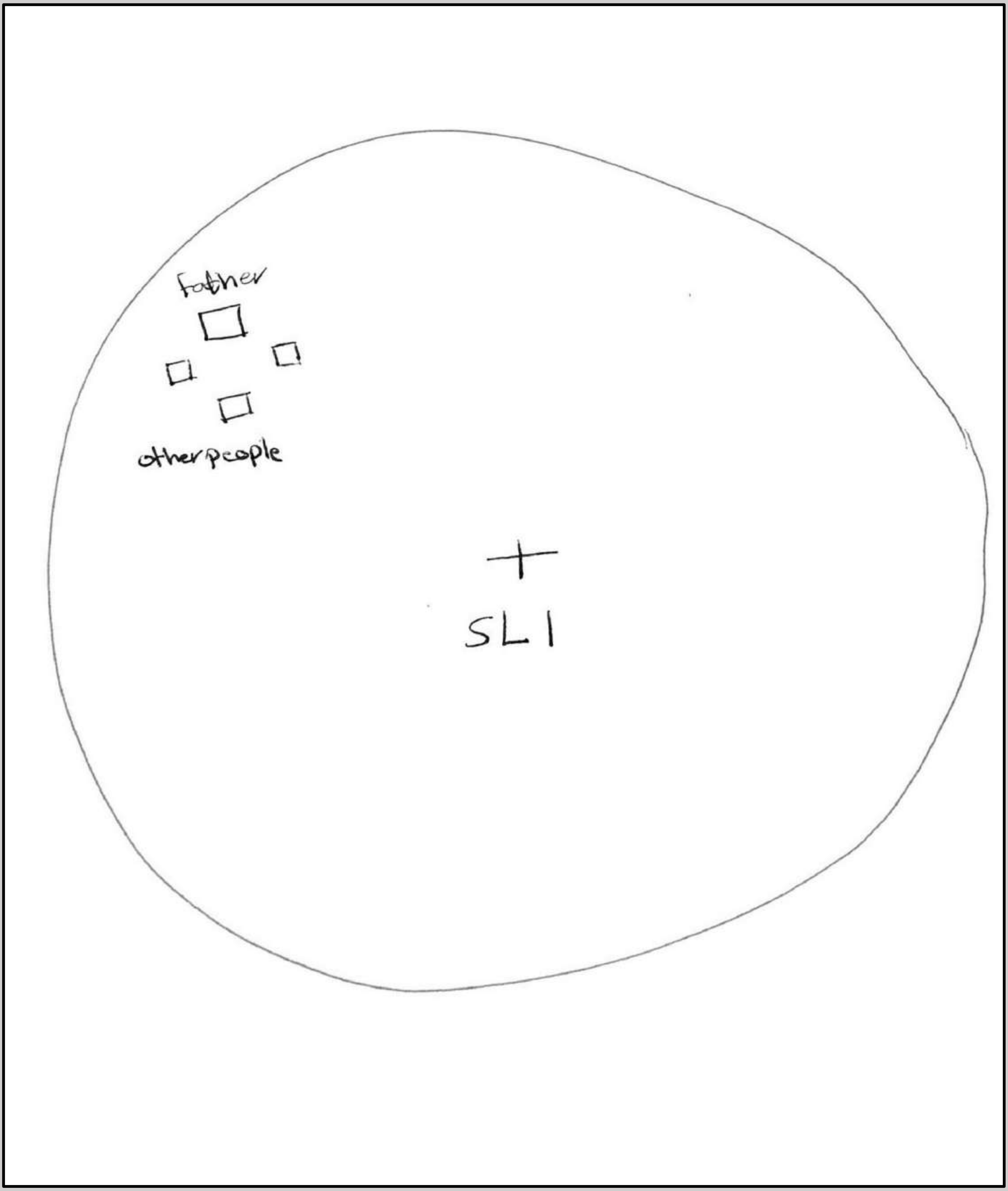

• C5

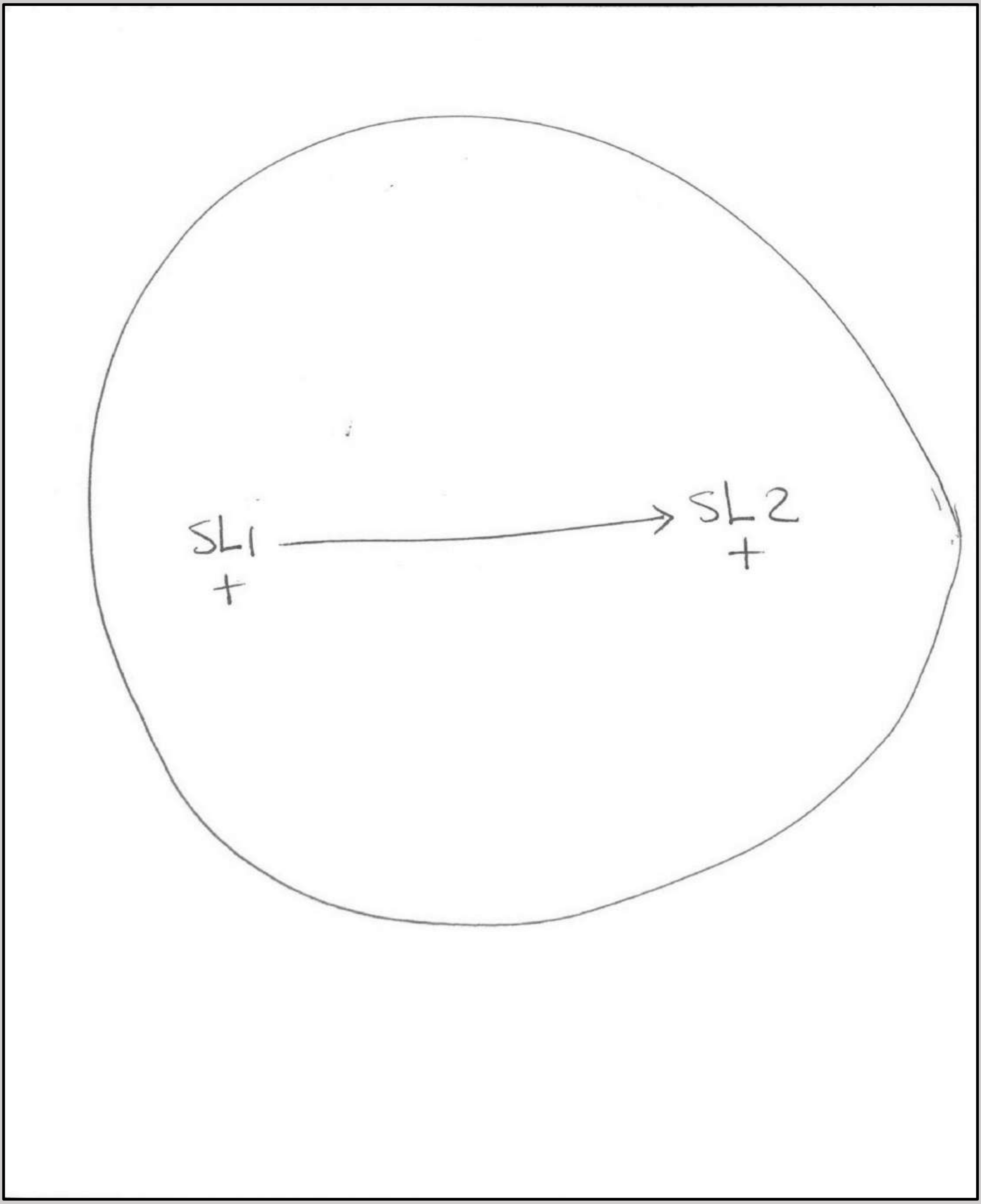

• B9

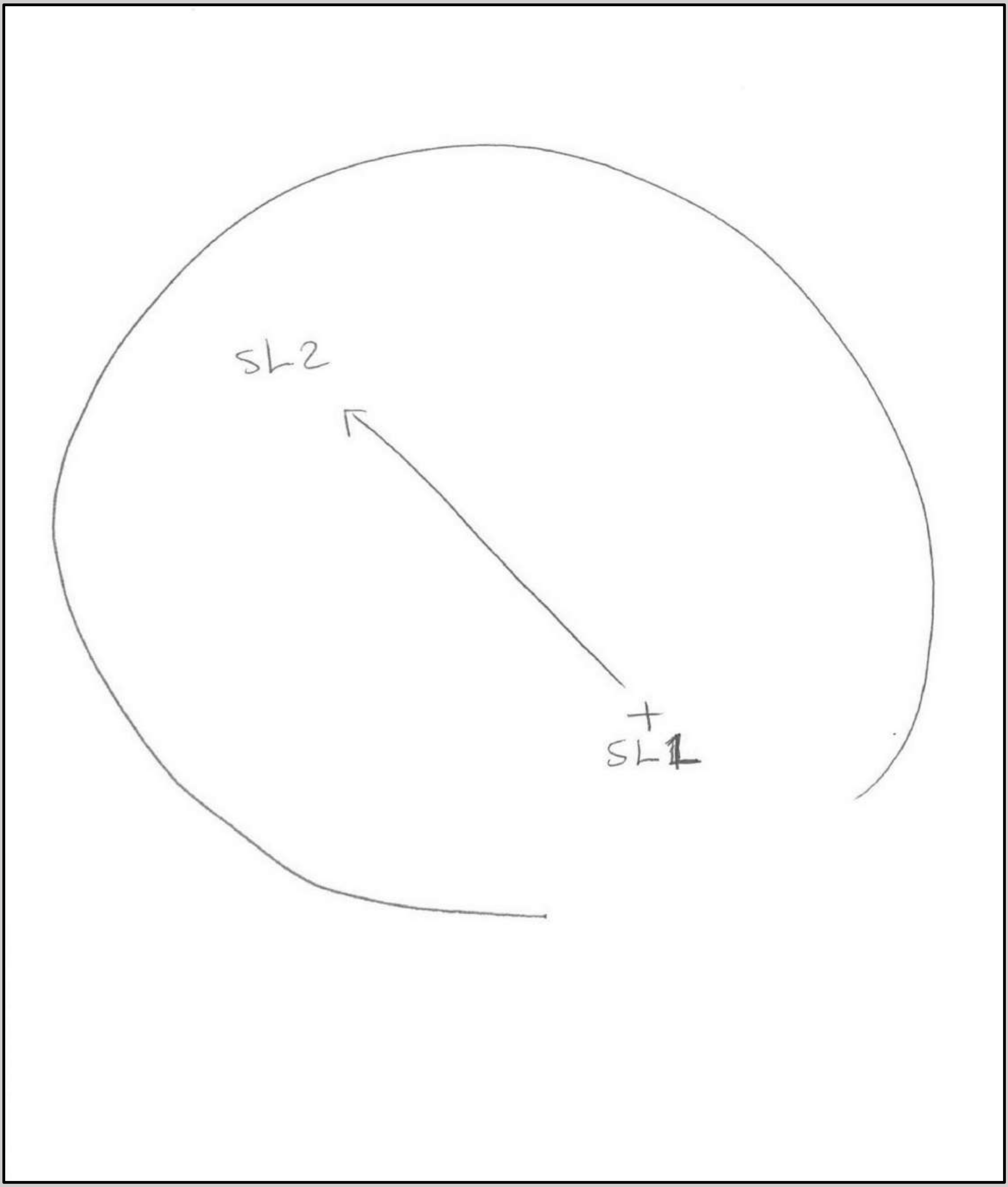

• B10

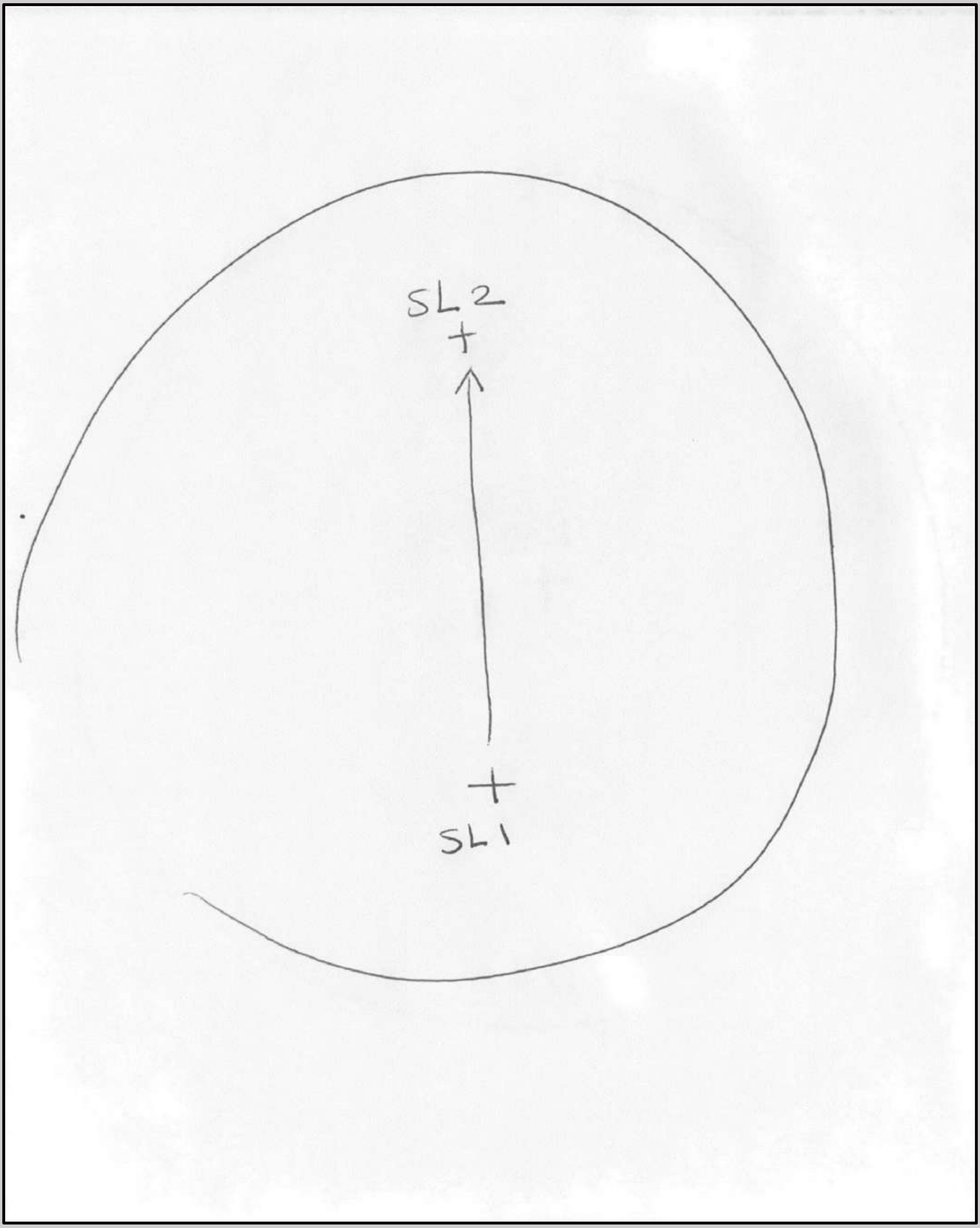

• B11

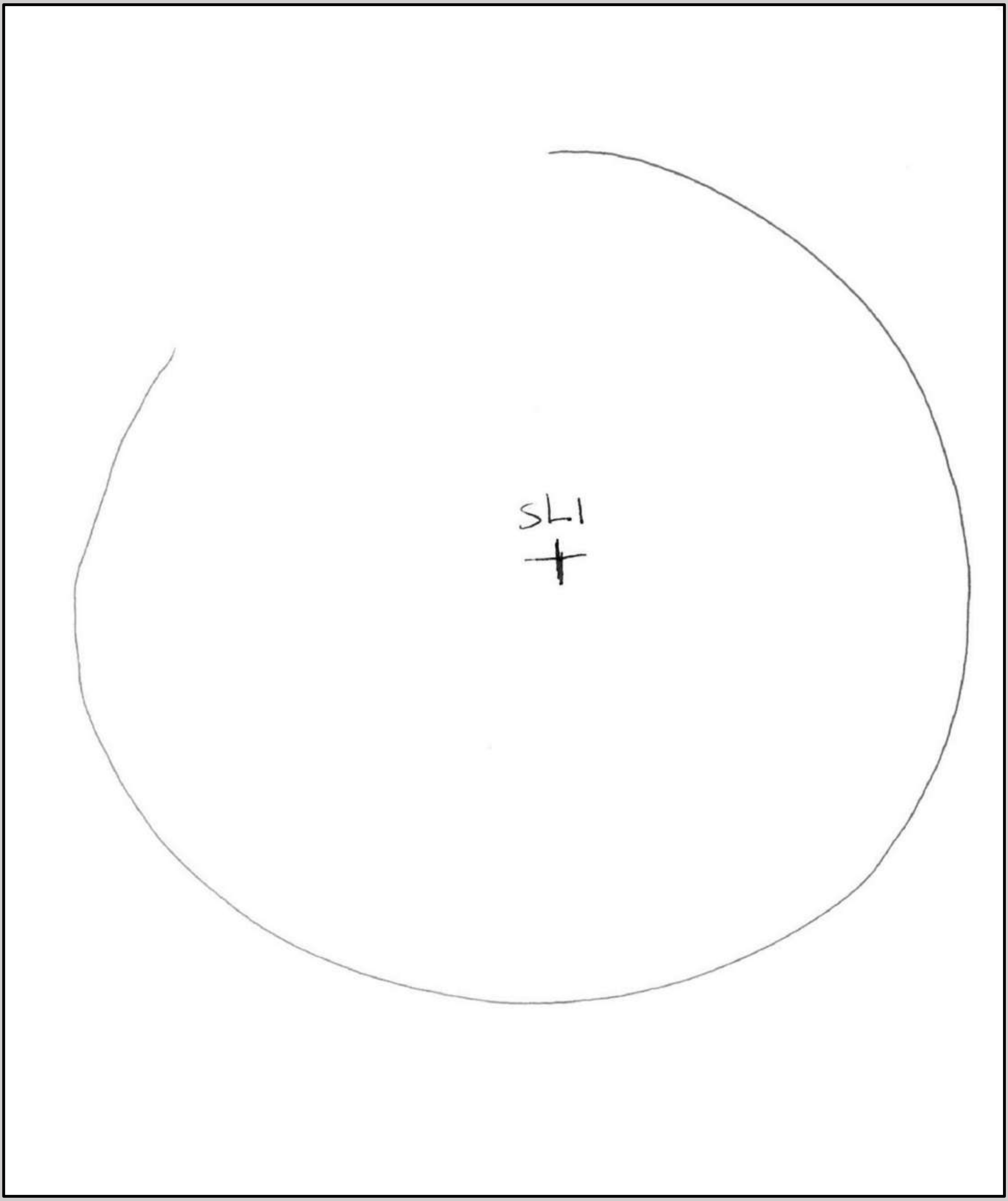

• B12

Subject 48

Sequence: part/space

1 NDE

- C5

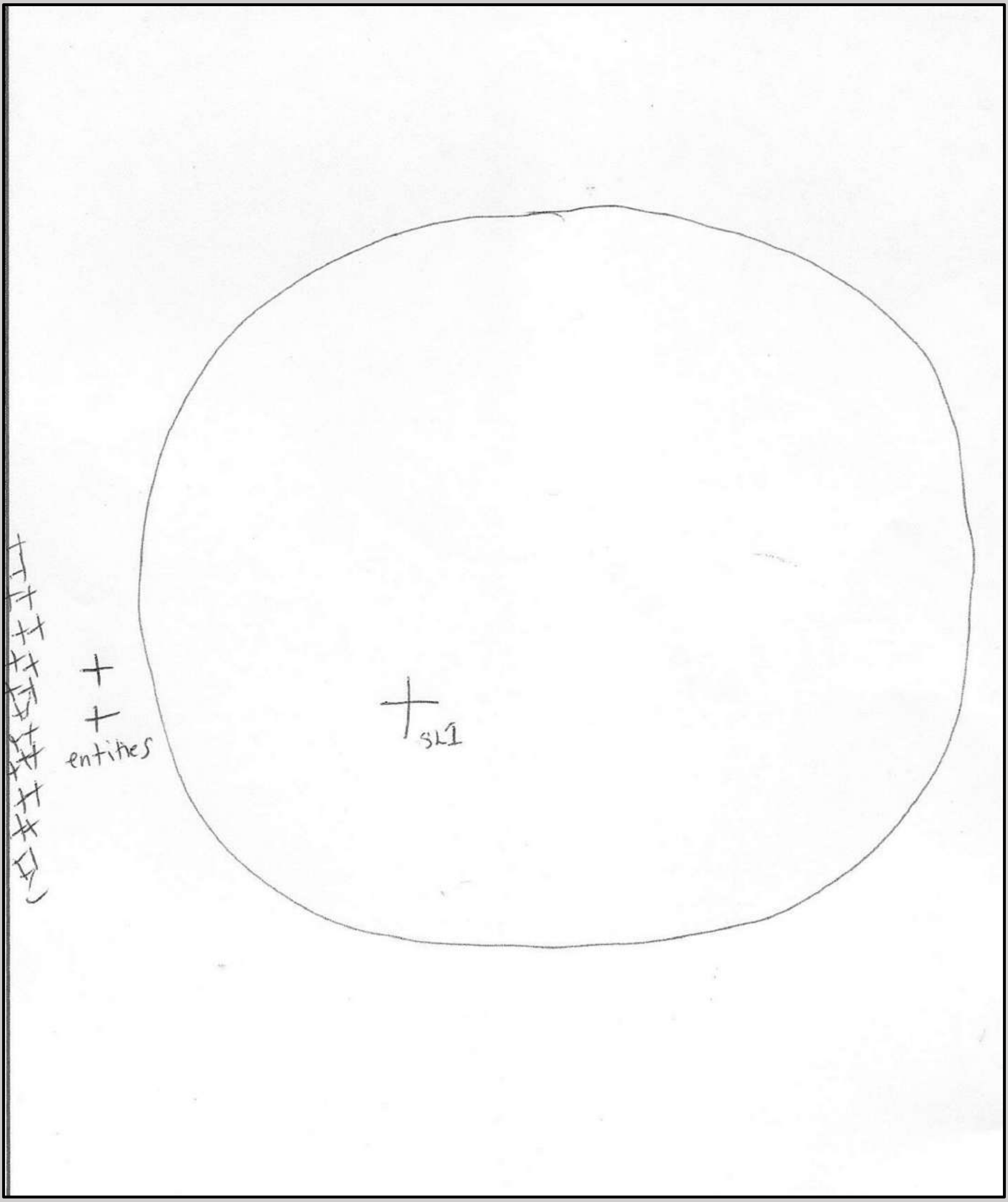

• C5

Subject 49

Sequence: part/space

1 OBE

- OBE Associative

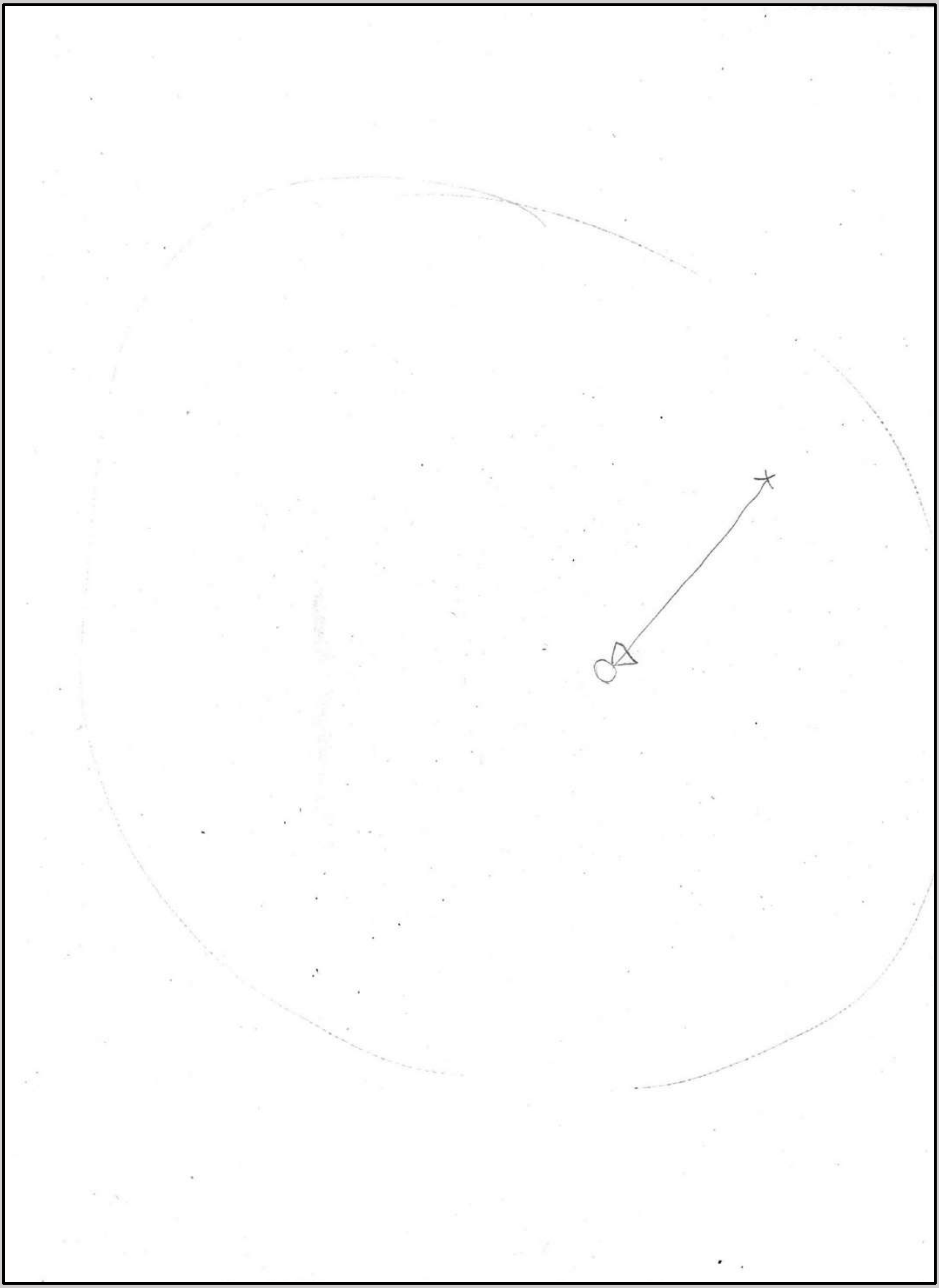

• OBE Associative

Subject 50

Sequence: part/space

1 OBE + 2 NDE

- OBE Dissociative
- Other space (freely hand-drawn)
- Other space (freely hand-drawn)

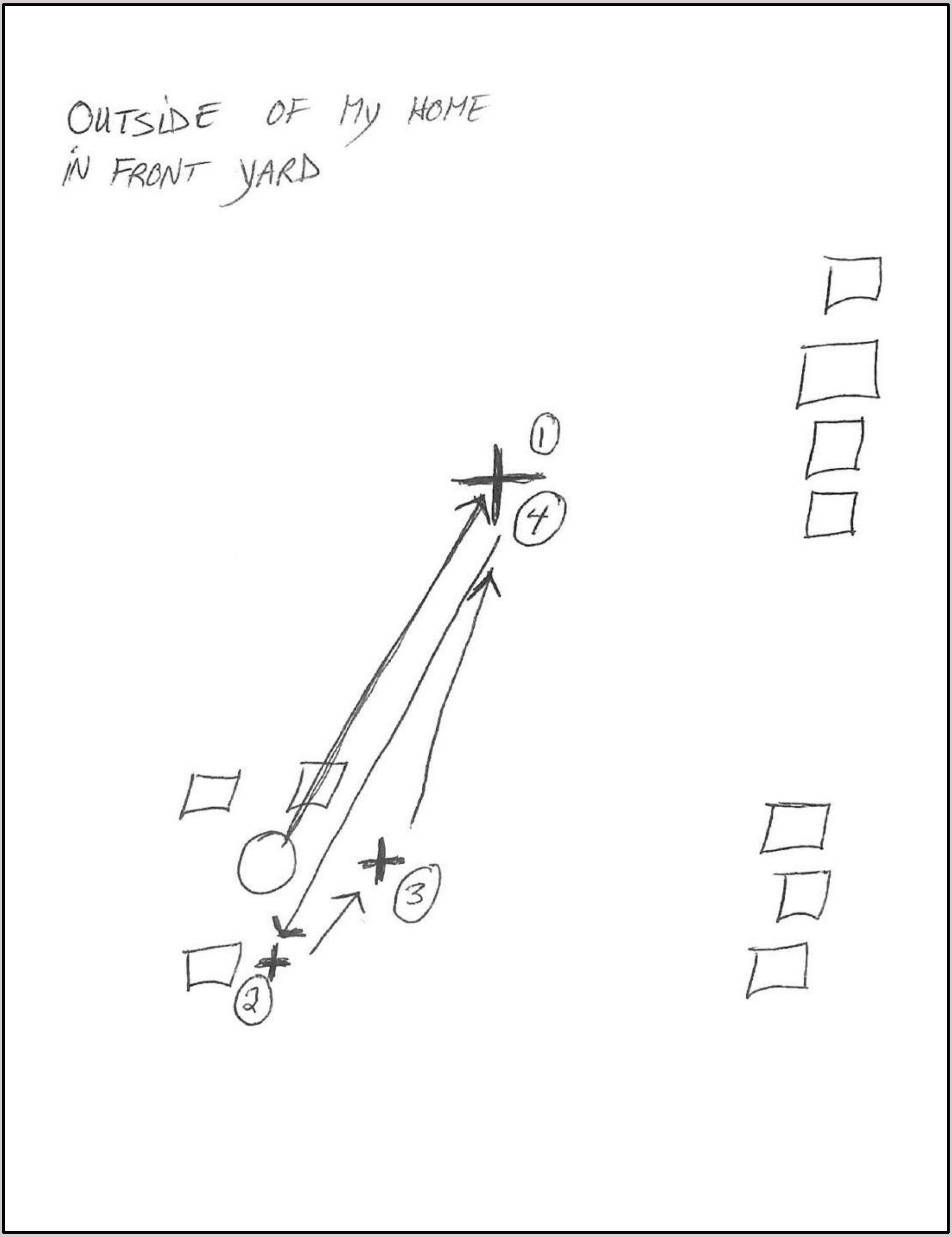

- OBE Dissociative

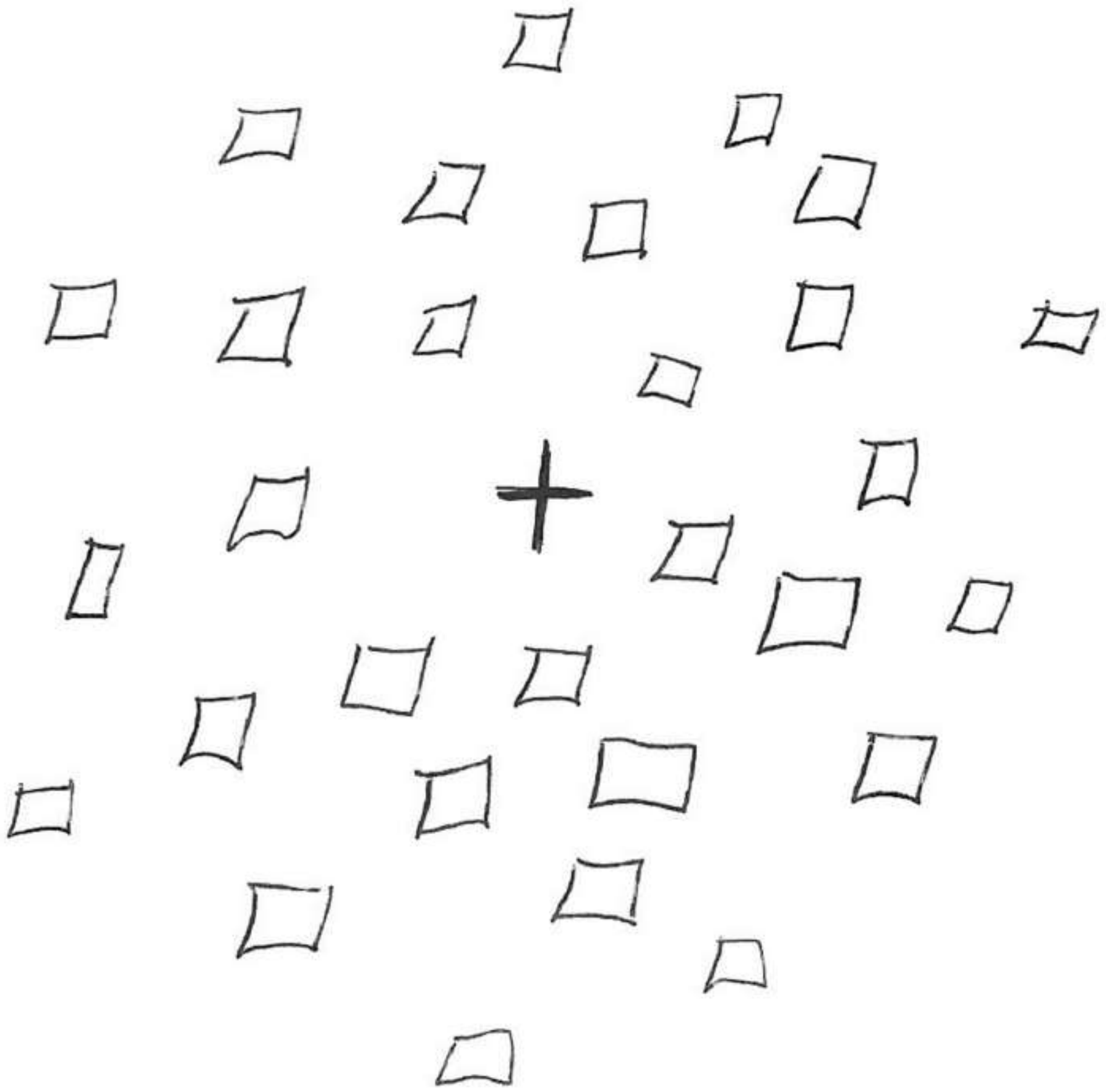

• Other space (freely hand-drawn)

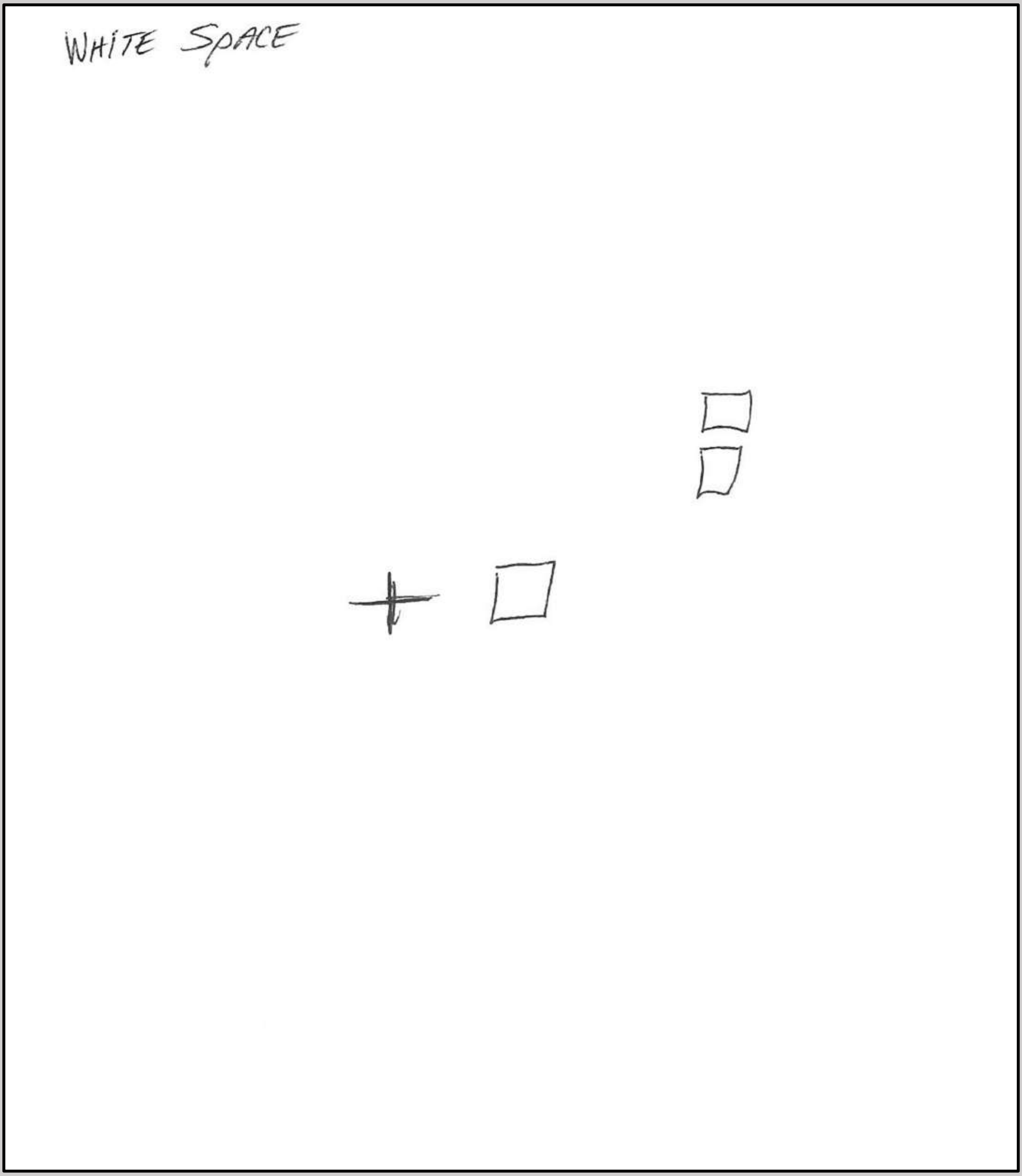

- Other space (freely hand-drawn)
