# Supplementary material for: The perceptual geometry and chromaticity of near-death experience spaces
Source: Front Psychol. 2026 Oct 15;17:1760389. doi: 10.3389/fpsyg.2026.1760389 (PMC13716671; doi:10.3389/fpsyg.2026.1760389)
Supplement: Supplementary file 8 [file Data_Sheet_8.pdf]

Supplementary Tables and Figures

1 TABLES

Table S1. Distribution of shapes for subjects with a single NDE part ($n = 21$).

	A	B	C1–4	C5	Other (freehand)	No space
**Count**	3	3	8	5	1	1
**Relative frequency**	14.3%	14.3%	38.1%	23.8%	4.8%	4.8%

Table S2. Self-Motion orientations (rectilinear) in Dissociative vs. Associative OBEs. Angles measured in a 2D page-fixed frame: 0° = rightward horizontal, 90° = upward vertical. Subjects 6 and 24, whose Self-Motions were rotational, is not included in this table despite reporting both an Associative and a Dissociative OBE.

Subject ID	Dissociative orientation (°)	Associative orientation (°)	Relative angle (°)
5	90.0	-90.0	-180.0
11	45.7	-90.0	-135.7
14	90.0	-90.0	-180.0
29	56.0	-39.7	-95.7
30	62.7	-149.5	-147.8
33	21.2	178.0	156.8
34	90.0	-90.0	-180.0
39	122.2	-40.6	-162.8
42	79.4	-99.0	-178.4
44	-131.7	-166.3	-34.6

Table S3. Relative frequency of shape categories occurring in middle positions of NDE sequences comprising more than one reconstructed NDE space. Middle positions were defined as positions that were neither first nor last in the sequence. This analysis was conducted at the level of individual reconstructed spaces. Fisher's exact tests compare each category with A shapes. Risk differences are expressed in percentage points relative to A shapes. Bonferroni-corrected significance threshold: $p < 0.0167$.

	A	B	C1–4	C5
Total occurrences	28	31	20	29
Middle occurrences	1	16	7	9
Relative frequency	3.6%	51.6%	35.0%	31.0%
95% Wilson CI	[0.6,17.7]	[35.0,68.0]	[18.1,56.7]	[17.3,49.2]
Risk difference vs. A	–	+48.0	+31.4	+27.5
Fisher exact p	–	0.00003	0.006	0.012

Table S4. Distribution of shapes preceding and following C5 ($n = 29$). "None" indicates C5 at sequence boundary (first/last).

	A	B	C1–4	C5	None
Before C5	6	7	4	2	10 (first)
After C5	3	8	1	2	15 (last)

Table S5. Paired occurrences of an A-shape followed by a subsequent ellipsoidal shape (B or C1–4) within the same NDE sequence. Visual Field Extent (VFE) values are given for both the A-shape and the subsequent shape.

Subject ID	Space A	VFE (°)	Subsequent Space	VFE (°)
3	Space 1 (A1)	0°	Space 2 (B11)	291.3°
4	Space 1 (A1)	0°	Space 2 (B10)	312.2°
7	Space 1 (A1)	0°	Space 2 (C2)	92.6°
10	Space 1 (A5)	29.1°	Space 2 (B6)	293°
11	Space 1 (A5)	32.8°	Space 2 (C2)	99.7°
12	Space 1 (A5)	27°	Space 2 (B9)	164.1°
13	Space 1 (A5)	9.7°	Space 2 (B6)	86.6°
15	Space 1 (A5)	19°	Space 2 (B6)	129.5°
17	Space 1 (A7)	27.7°	Space 2 (B10)	256.3°
18	Space 1 (A8)	18.4°	Space 2 (B6)	102°
20	Space 1 (A9)	36.5°	Space 2 (B6)	170.2°
42	Space 2 (A7)	39.6°	Space 3 (C2)	225°

Table S6. Subjects and pages for Dissociative vs. Associative OBE phases (from Supplementary Material: *Graphic Representations*).

Subject ID	Page	OBE Phase
1	3	Dissociative
4	14	Dissociative
5	18	Dissociative
5	21	Associative
6	23	Dissociative
6	30	Associative
7	32	Dissociative
8	36	Dissociative
9	41	Associative
10	48	Associative
11	50	Dissociative
11	59	Associative
12	65	Associative
13	67	Dissociative
14	71	Dissociative
14	74	Associative
15	78	Associative
17	82	Dissociative
19	91	Dissociative
20	98	Associative
21	106	Associative
24	115	Dissociative
24	117	Associative
29	132	Dissociative
29	136	Associative
30	138	Dissociative
30	140	Associative
33	147	Dissociative
33	149	Associative
34	151	Dissociative
34	153	Associative
37	158	Dissociative
39	164	Dissociative
39	166	Associative
40	169	Associative
41	171	Dissociative
42	175	Dissociative
42	179	Associative
44	184	Dissociative
44	186	Associative
46	192	Associative
47	194	Dissociative
49	203	Associative
50	205	Dissociative

2 FIGURES

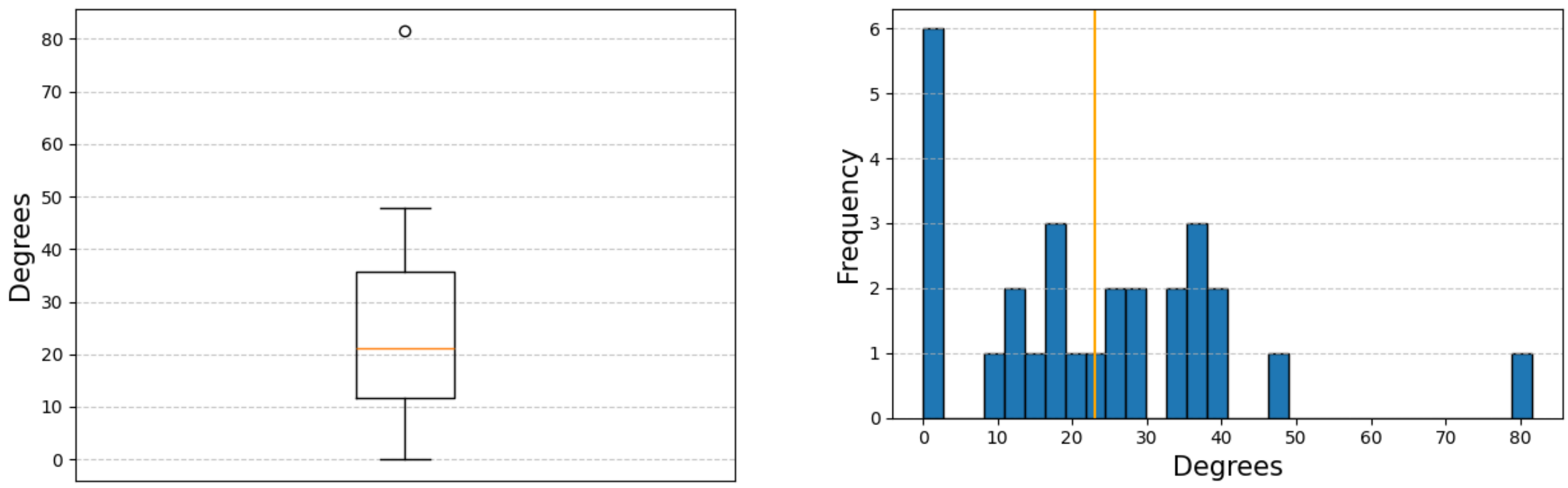

(a) (b)

Figure S1: Distribution of opening angles for A shapes. (a) Box plot showing the median; (b) histogram showing the mean.

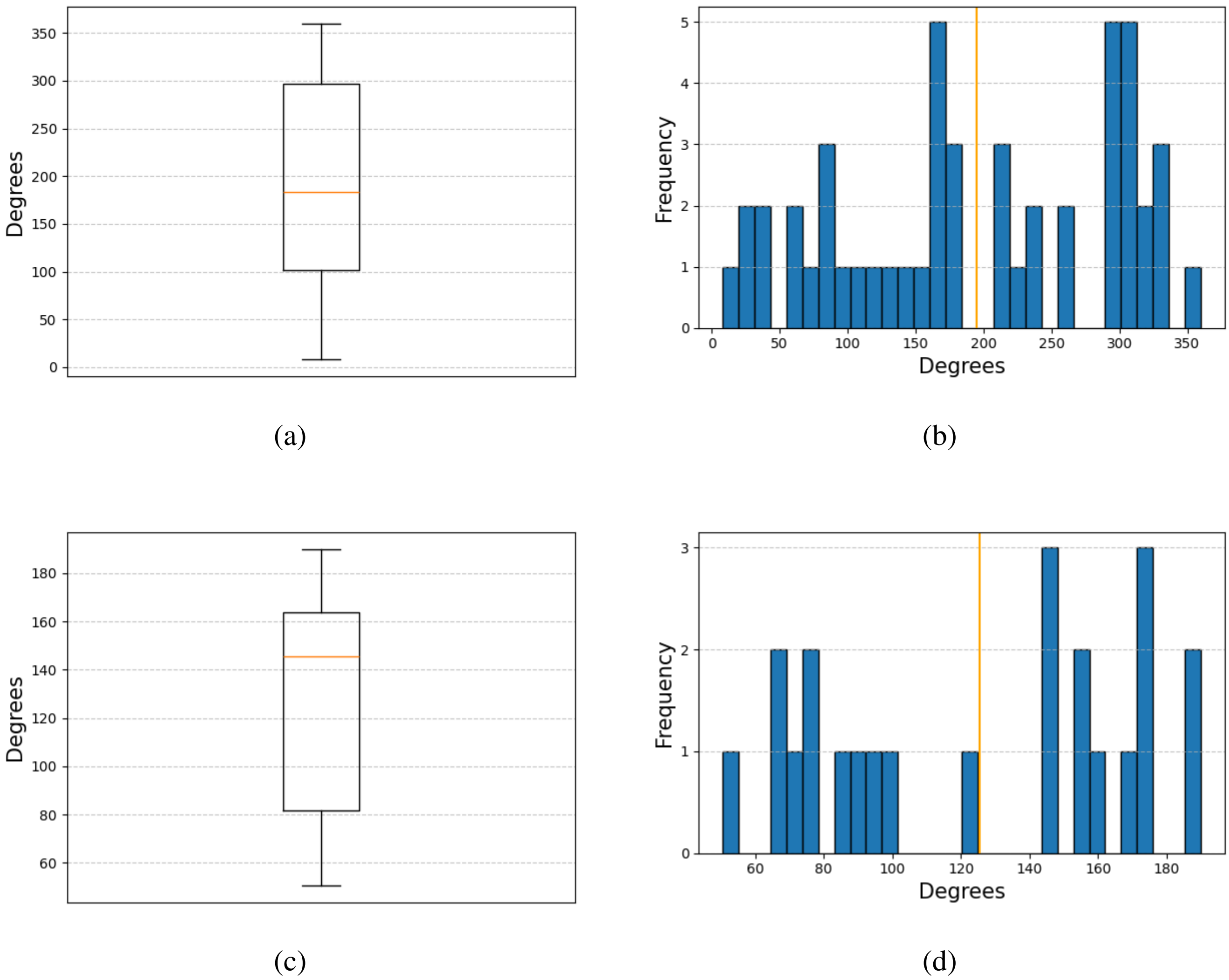

Figure S2: Distribution of Visual Field Extents (VFEs) for B and C1–4 shapes. (a) Box plot of VFEs in B shapes; (b) histogram of VFEs in B shapes; (c) box plot of VFEs in C1–4 shapes; (d) histogram of VFEs in C1–4 shapes.

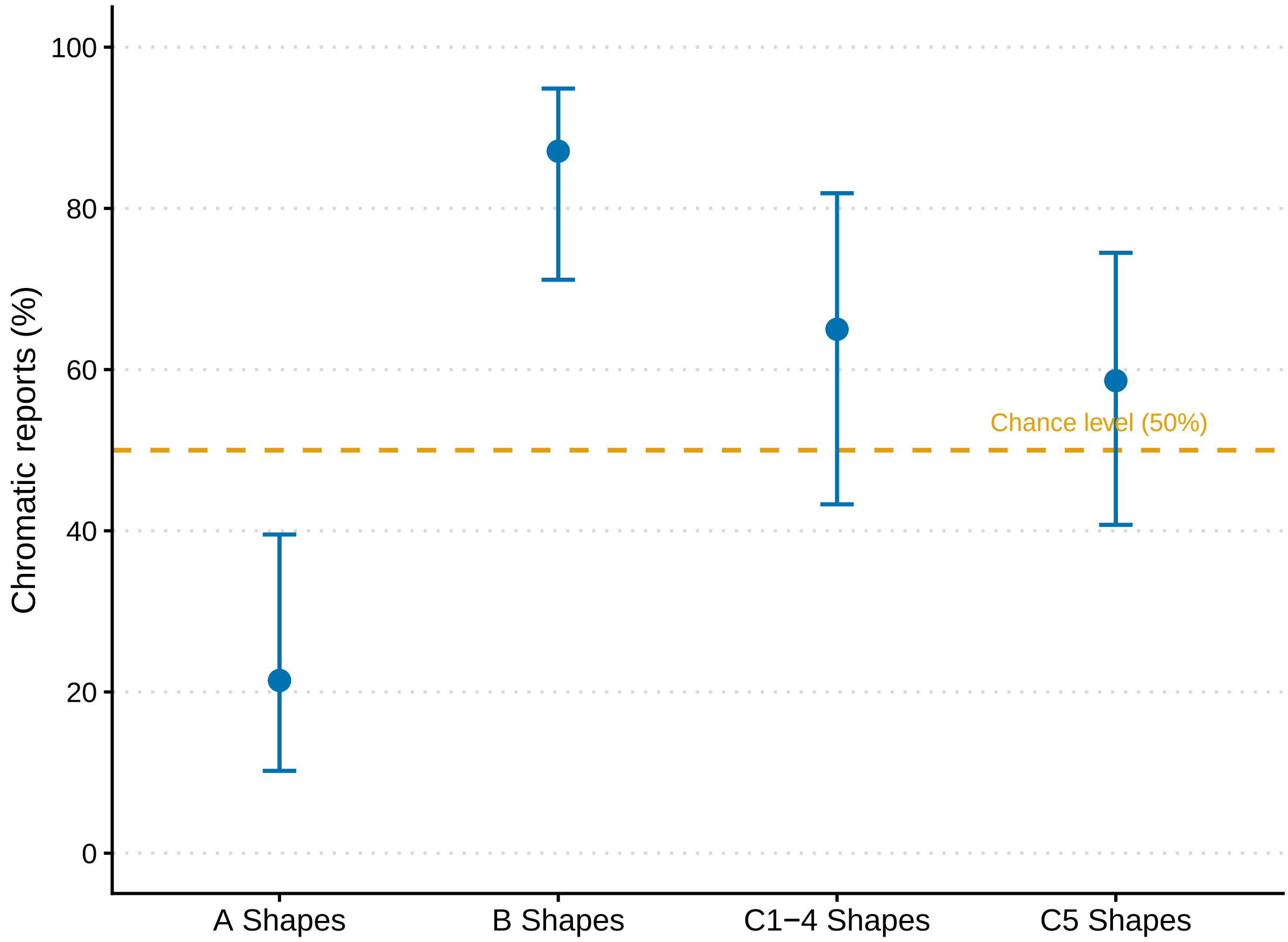

Figure S3: Chromatic proportions across Visual Field categories with 95% Wilson confidence intervals.

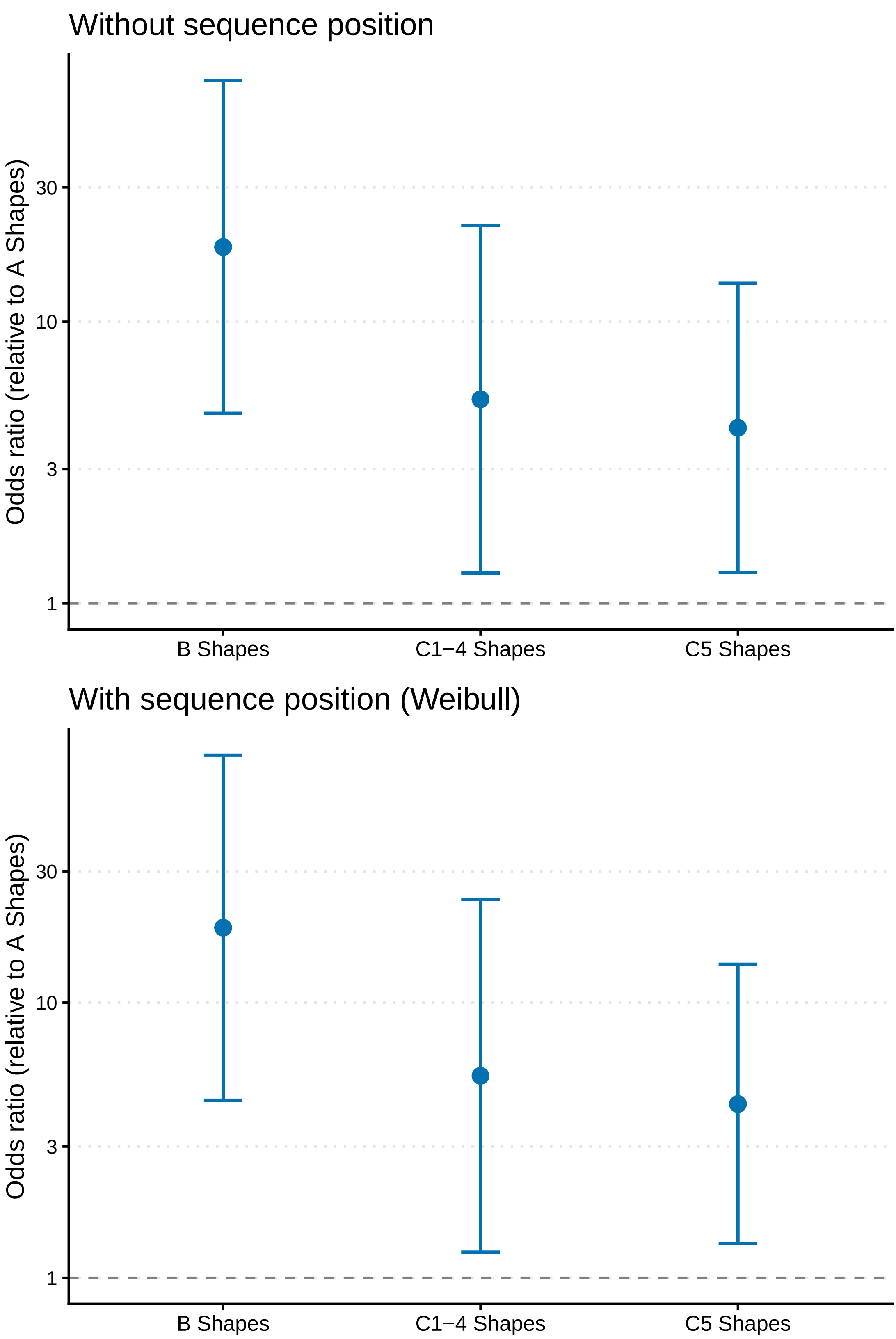

Figure S4: Generalized estimating equation analysis of chromatic reports relative to type A shape spaces. Odds ratios are shown with 95% confidence intervals; the dashed line at 1 indicates no effect.
(a) Model without sequence position.
(b) Model including normalized sequence position (Weibull plotting position).
