# Supplementary material for: The perceptual geometry and chromaticity of near-death experience spaces
Source: Front Psychol. 2026 Oct 15;17:1760389. doi: 10.3389/fpsyg.2026.1760389 (PMC13716671; doi:10.3389/fpsyg.2026.1760389)
Supplement: Supplementary file 9 [file Data_Sheet_9.pdf]

```
#########################################################################
#######
# Supplementary analysis scripts
# The Perceptual Geometry and Chromaticity of Near-Death Experience Spaces
#
# Contents:
#   1. Code-ChromaticityAnalysis.py
#   2. Code-SupFigS3S4.R
#   3. Code-SupTabS5.py
#   4. Code-ShapeScales.py
#########################################################################
#######


#
=======================================================================
=======
# 1. Code-ChromaticityAnalysis.py
# Chromaticity by visual-field geometry.
# Wilson CIs, chi-square, Fisher-Freeman-Halton, pairwise Fisher (Holm),
# GEE (statsmodels) with/without Weibull position, approximate Mancl-DeRouen.
#
=======================================================================
=======

"""
Chromaticity by visual-field geometry.
Wilson CIs, chi-square, Fisher-Freeman-Halton, pairwise Fisher (Holm),
GEE (statsmodels) with/without Weibull position, approximate Mancl-DeRouen.
"""

import numpy as np
import pandas as pd
from scipy import stats
from statsmodels.stats.proportion import proportion_confint
from statsmodels.stats.multitest import multipletests
from statsmodels.genmod.generalized_estimating_equations import GEE
from statsmodels.genmod.cov_struct import Exchangeable
from statsmodels.genmod.families import Binomial
from numpy.linalg import inv, pinv

# Space, Subject, Weibull_position, Shape, Chromatic (Yes/No)
rows = [
(1,1,0.2,"B","Yes"),(2,1,0.4,"B","Yes"),(3,1,0.6,"C5","No"),(4,1,0.8,"A","No"),
(5,3,1/3,"A","No"),(6,3,2/3,"B","No"),
(7,4,1/3,"A","Yes"),(8,4,2/3,"B","Yes"),
(9,5,1/3,"A","No"),(10,5,2/3,"C5","No"),
(11,6,1/7,"A","No"),(12,6,2/7,"C5","Yes"),(13,6,3/7,"B","Yes"),
```

(14,6,4/7,"C","Yes"),(15,6,5/7,"C","Yes"),(16,6,6/7,"C5","Yes"),
(17,7,1/3,"A","Yes"),(18,7,2/3,"C","No"),
(19,8,0.5,"A","No"),
(20,9,1/3,"A","No"),(21,9,2/3,"A","No"),
(22,10,1/6,"A","Yes"),(23,10,2/6,"B","Yes"),(24,10,3/6,"C5","No"),
(25,10,4/6,"B","Yes"),(26,10,5/6,"A","No"),
(27,11,1/9,"A","No"),(28,11,2/9,"C","Yes"),(29,11,3/9,"C5","Yes"),
(30,11,4/9,"B","Yes"),(31,11,5/9,"C5","Yes"),(32,11,6/9,"B","Yes"),
(33,11,7/9,"C5","Yes"),(34,11,8/9,"B","Yes"),
(35,12,0.25,"A","No"),(36,12,0.5,"B","Yes"),(37,12,0.75,"C","Yes"),
(38,13,1/3,"A","No"),(39,13,2/3,"B","Yes"),
(40,14,1/3,"A","No"),(41,14,2/3,"C5","No"),
(42,15,1/3,"A","No"),(43,15,2/3,"B","Yes"),
(44,16,0.5,"A","Yes"),
(45,17,0.25,"A","No"),(46,17,0.5,"B","Yes"),(47,17,0.75,"C5","No"),
(48,18,0.25,"A","No"),(49,18,0.5,"B","Yes"),(50,18,0.75,"C5","Yes"),
(51,19,0.5,"A","Yes"),
(52,20,0.2,"A","No"),(53,20,0.4,"B","No"),(54,20,0.6,"C","No"),(55,20,0.8,"C5","No"),
(56,21,1/7,"A","No"),(57,21,2/7,"C5","Yes"),(58,21,3/7,"B","Yes"),
(59,21,4/7,"C","Yes"),(60,21,5/7,"C","Yes"),(61,21,6/7,"C5","No"),
(62,22,1/3,"A","No"),(63,22,2/3,"C5","Yes"),
(64,23,0.25,"A","No"),(65,23,0.5,"C5","Yes"),(66,23,0.75,"A","No"),
(67,24,0.5,"B","Yes"),
(68,25,0.2,"B","No"),(69,25,0.4,"C5","No"),(70,25,0.6,"B","No"),(71,25,0.8,"A","No"),
(72,26,0.5,"B","Yes"),
(73,27,0.5,"B","Yes"),
(74,28,0.25,"B","Yes"),(75,28,0.5,"C","Yes"),(76,28,0.75,"B","Yes"),
(77,29,0.25,"B","Yes"),(78,29,0.5,"B","Yes"),(79,29,0.75,"B","Yes"),
(80,30,0.5,"C","Yes"),
(81,31,0.5,"C","No"),
(82,32,1/3,"C","No"),(83,32,2/3,"A","No"),
(84,33,0.5,"C","Yes"),
(85,34,0.5,"C","No"),
(86,35,0.5,"C","Yes"),
(87,36,0.5,"C","No"),
(88,37,0.5,"C","Yes"),
(89,38,0.5,"C","Yes"),
(90,39,0.5,"C5","Yes"),
(91,40,0.5,"C5","No"),
(92,41,1/3,"C5","Yes"),(93,41,2/3,"C","Yes"),
(94,42,0.25,"C5","No"),(95,42,0.5,"A","Yes"),(96,42,0.75,"C","No"),
(97,43,1/3,"C5","No"),(98,43,2/3,"C5","Yes"),
(99,44,0.5,"C5","Yes"),
(100,45,0.5,"C5","Yes"),
(101,46,1/3,"C5","Yes"),(102,46,2/3,"C5","Yes"),
(103,47,1/6,"C5","Yes"),(104,47,2/6,"B","Yes"),(105,47,3/6,"B","Yes"),
(106,47,4/6,"B","Yes"),(107,47,5/6,"B","Yes"),
(108,48,0.5,"C5","No"),

```
]

df = pd.DataFrame(rows, columns=["Space", "Subject", "Weibull", "Shape", "Chromatic"])
df["y"] = (df["Chromatic"] == "Yes").astype(int)
df["Shape"] = df["Shape"].replace({"C": "C1-4"})
df["Shape"] = pd.Categorical(df["Shape"], categories=["A", "B", "C1-4", "C5"])

# --- Contingency + Wilson ---
print("=== Contingency table ===")
tab = pd.crosstab(df["Shape"], df["Chromatic"])
print(tab)

print("\n=== Proportions + Wilson 95% CI ===")
for sh in ["A", "B", "C1-4", "C5"]:
    sub = df[df["Shape"] == sh]
    k, n = sub["y"].sum(), len(sub)
    lo, hi = proportion_confint(k, n, method="wilson")
    print(f"{sh:5} {k}/{n} = {100*k/n:.1f}%  CI [{100*lo:.1f}, {100*hi:.1f}]")

# --- Chi-square + Cramer V ---
chi2, p_chi, dof, exp = stats.chi2_contingency(tab.values)
n_tot = tab.values.sum()
v = np.sqrt(chi2 / (n_tot * (min(tab.shape) - 1)))
print(f"\nChi2({dof}) = {chi2:.2f}, p = {p_chi:.3e}, Cramer V = {v:.3f}")
print("Expected counts:\n", np.round(exp, 2))

# --- Fisher-Freeman-Halton ---
print("Fisher-Freeman-Halton: run fisher.test(table) in R for exact p;")
print("Manuscript value: p = 3.71e-6")

# Pairwise Fisher + Holm
pairs = [("A", "B"), ("A", "C1-4"), ("A", "C5"), ("B", "C1-4"), ("B", "C5"), ("C1-4", "C5")]
p_raw = []
for a, b in pairs:
    sub = tab.loc[[a, b], :]
    p_raw.append(stats.fisher_exact(sub.values)[1])
p_holm = multipletests(p_raw, method="holm")[1]
print("\n=== Pairwise Fisher (Holm) ===")
for (a, b), pr, ph in zip(pairs, p_raw, p_holm):
    print(f"{a} vs {b}: p_raw={pr:.4g}, p_Holm={ph:.4g}")

# --- GEE ---
df["Shape_B"] = (df["Shape"] == "B").astype(float)
df["Shape_C14"] = (df["Shape"] == "C1-4").astype(float)
df["Shape_C5"] = (df["Shape"] == "C5").astype(float)
X = df[["Shape_B", "Shape_C14", "Shape_C5"]].copy()
X.insert(0, "Intercept", 1.0)
y = df["y"].values
```

```
groups = df["Subject"].values

fam = Binomial()
cov = Exchangeable()

print("\n=== GEE without position ===")
res0 = GEE(y, X, groups=groups, family=fam, cov_struct=cov).fit()
print(res0.summary())
beta0 = np.asarray(res0.params)
se0 = np.asarray(res0.bse)
for name, b, s in zip(X.columns, beta0, se0):
    if name == "Intercept":
        continue
    or_ = np.exp(b)
    lo, hi = np.exp(b - 1.96 * s), np.exp(b + 1.96 * s)
    z = b / s
    p = 2 * stats.norm.sf(abs(z))
    print(f"{name}: OR={or_:.2f}, 95% CI [{lo:.2f}, {hi:.2f}], p={p:.4g}")

X1 = X.copy()
X1["Weibull"] = df["Weibull"].values
print("\n=== GEE with Weibull position ===")
res1 = GEE(y, X1, groups=groups, family=fam, cov_struct=cov).fit()
print(res1.summary())
for name, b, s in zip(X1.columns, np.asarray(res1.params), np.asarray(res1.bse)):
    if name == "Intercept":
        continue
    or_ = np.exp(b)
    lo, hi = np.exp(b - 1.96 * s), np.exp(b + 1.96 * s)
    z = b / s
    p = 2 * stats.norm.sf(abs(z))
    print(f"{name}: OR={or_:.2f}, 95% CI [{lo:.2f}, {hi:.2f}], p={p:.4g}")

# --- Approximate Mancl-DeRouen (independence-working scores) ---
print("\n=== Approximate Mancl-DeRouen (no-position model) ===")
mu = res0.fittedvalues
unique_g = np.unique(groups)
pdim = X.shape[1]
scores = []
D_list = []
Xv = X.values
for g in unique_g:
    idx = groups == g
    Xg = Xv[idx]
    yg = y[idx]
    mug = mu[idx]
    Vg = mug * (1 - mug)
    scores.append(Xg.T @ (yg - mug))
```

```
    D_list.append(Xg.T @ np.diag(Vg) @ Xg)
scores = np.array(scores)
Amat = sum(D_list)
Ainv = inv(Amat) if np.linalg.matrix_rank(Amat) == pdim else pinv(Amat)
V_md = np.zeros((pdim, pdim))
for i in range(len(unique_g)):
    Hi = Ainv @ D_list[i]
    try:
        corr = inv(np.eye(pdim) - Hi)
    except np.linalg.LinAlgError:
        corr = pinv(np.eye(pdim) - Hi)
    Ui = scores[i]
    V_md += np.outer(corr @ Ui, corr @ Ui)
V_md = Ainv @ V_md @ Ainv
se_md = np.sqrt(np.diag(V_md))
for name, b, s in zip(X.columns, beta0, se_md):
    if name == "Intercept":
        continue
    lo, hi = np.exp(b - 1.96 * s), np.exp(b + 1.96 * s)
    print(f"{name}: OR={np.exp(b):.2f}, MD 95% CI [{lo:.2f}, {hi:.2f}]")

print(f"\nNumber of clusters: {len(unique_g)}")


#
==========================================================================
=======
# 2. Code-SupFigS3S4.R
# Chromatic reports by visual-field geometry
# GEE models with and without sequence position
# Figures S3, S4a and S4b
#
==========================================================================
=======

# ============================================================
# Chromatic reports by visual-field geometry
# GEE models with and without sequence position
# Figures S3, S4a and S4b
# ============================================================

library(tidyverse)
library(geepack)
library(binom)
library(ggplot2)

# ------------------------------------------------------------
# 1. Data
```

```
# ------------------------------------------------------------
dat <- tribble(
  ~Space, ~Subject, ~Weibull_position, ~Shape, ~Chromatic,
  1, 1, 0.2, "B", "Yes",
  2, 1, 0.4, "B", "Yes",
  3, 1, 0.6, "C5", "No",
  4, 1, 0.8, "A", "No",
  5, 3, 0.3333333333, "A", "No",
  6, 3, 0.6666666667, "B", "No",
  7, 4, 0.3333333333, "A", "Yes",
  8, 4, 0.6666666667, "B", "Yes",
  9, 5, 0.3333333333, "A", "No",
  10, 5, 0.6666666667, "C5", "No",
  11, 6, 0.1428571429, "A", "No",
  12, 6, 0.2857142857, "C5", "Yes",
  13, 6, 0.4285714286, "B", "Yes",
  14, 6, 0.5714285714, "C", "Yes",
  15, 6, 0.7142857143, "C", "Yes",
  16, 6, 0.8571428571, "C5", "Yes",
  17, 7, 0.3333333333, "A", "Yes",
  18, 7, 0.6666666667, "C", "No",
  19, 8, 0.5, "A", "No",
  20, 9, 0.3333333333, "A", "No",
  21, 9, 0.6666666667, "A", "No",
  22, 10, 0.1666666667, "A", "Yes",
  23, 10, 0.3333333333, "B", "Yes",
  24, 10, 0.5, "C5", "No",
  25, 10, 0.6666666667, "B", "Yes",
  26, 10, 0.8333333333, "A", "No",
  27, 11, 0.1111111111, "A", "No",
  28, 11, 0.2222222222, "C", "Yes",
  29, 11, 0.3333333333, "C5", "Yes",
  30, 11, 0.4444444444, "B", "Yes",
  31, 11, 0.5555555556, "C5", "Yes",
  32, 11, 0.6666666667, "B", "Yes",
  33, 11, 0.7777777778, "C5", "Yes",
  34, 11, 0.8888888889, "B", "Yes",
  35, 12, 0.25, "A", "No",
  36, 12, 0.5, "B", "Yes",
  37, 12, 0.75, "C", "Yes",
  38, 13, 0.3333333333, "A", "No",
  39, 13, 0.6666666667, "B", "Yes",
  40, 14, 0.3333333333, "A", "No",
  41, 14, 0.6666666667, "C5", "No",
  42, 15, 0.3333333333, "A", "No",
  43, 15, 0.6666666667, "B", "Yes",
  44, 16, 0.5, "A", "Yes",
  45, 17, 0.25, "A", "No",
```

46, 17, 0.5, "B", "Yes",
47, 17, 0.75, "C5", "No",
48, 18, 0.25, "A", "No",
49, 18, 0.5, "B", "Yes",
50, 18, 0.75, "C5", "Yes",
51, 19, 0.5, "A", "Yes",
52, 20, 0.2, "A", "No",
53, 20, 0.4, "B", "No",
54, 20, 0.6, "C", "No",
55, 20, 0.8, "C5", "No",
56, 21, 0.1428571429, "A", "No",
57, 21, 0.2857142857, "C5", "Yes",
58, 21, 0.4285714286, "B", "Yes",
59, 21, 0.5714285714, "C", "Yes",
60, 21, 0.7142857143, "C", "Yes",
61, 21, 0.8571428571, "C5", "No",
62, 22, 0.3333333333, "A", "No",
63, 22, 0.6666666667, "C5", "Yes",
64, 23, 0.25, "A", "No",
65, 23, 0.5, "C5", "Yes",
66, 23, 0.75, "A", "No",
67, 24, 0.5, "B", "Yes",
68, 25, 0.2, "B", "No",
69, 25, 0.4, "C5", "No",
70, 25, 0.6, "B", "No",
71, 25, 0.8, "A", "No",
72, 26, 0.5, "B", "Yes",
73, 27, 0.5, "B", "Yes",
74, 28, 0.25, "B", "Yes",
75, 28, 0.5, "C", "Yes",
76, 28, 0.75, "B", "Yes",
77, 29, 0.25, "B", "Yes",
78, 29, 0.5, "B", "Yes",
79, 29, 0.75, "B", "Yes",
80, 30, 0.5, "C", "Yes",
81, 31, 0.5, "C", "No",
82, 32, 0.3333333333, "C", "No",
83, 32, 0.6666666667, "A", "No",
84, 33, 0.5, "C", "Yes",
85, 34, 0.5, "C", "No",
86, 35, 0.5, "C", "Yes",
87, 36, 0.5, "C", "No",
88, 37, 0.5, "C", "Yes",
89, 38, 0.5, "C", "Yes",
90, 39, 0.5, "C5", "Yes",
91, 40, 0.5, "C5", "No",
92, 41, 0.3333333333, "C5", "Yes",
93, 41, 0.6666666667, "C", "Yes",

```
  94, 42, 0.25, "C5", "No",
  95, 42, 0.5, "A", "Yes",
  96, 42, 0.75, "C", "No",
  97, 43, 0.3333333333, "C5", "No",
  98, 43, 0.6666666667, "C5", "Yes",
  99, 44, 0.5, "C5", "Yes",
  100, 45, 0.5, "C5", "Yes",
  101, 46, 0.3333333333, "C5", "Yes",
  102, 46, 0.6666666667, "C5", "Yes",
  103, 47, 0.1666666667, "C5", "Yes",
  104, 47, 0.3333333333, "B", "Yes",
  105, 47, 0.5, "B", "Yes",
  106, 47, 0.6666666667, "B", "Yes",
  107, 47, 0.8333333333, "B", "Yes",
  108, 48, 0.5, "C5", "No"
)

dat <- dat %>%
  mutate(
    Chromatic_bin = ifelse(Chromatic == "Yes", 1L, 0L),
    Shape = factor(Shape, levels = c("A", "B", "C", "C5")),
    Shape = forcats::fct_recode(Shape, "C1-4" = "C"),
    Subject = factor(Subject)
  )

# -----------------------------------------------------------
# 2. Descriptive proportions + Wilson CIs (Figure S3)
# -----------------------------------------------------------
prop_table <- dat %>%
  group_by(Shape) %>%
  summarise(
    n = n(),
    successes = sum(Chromatic_bin),
    .groups = "drop"
  ) %>%
  rowwise() %>%
  mutate(
    ci = list(binom.confint(successes, n, methods = "wilson")),
    proportion = successes / n,
    lower = ci$lower,
    upper = ci$upper
  ) %>%
  select(Shape, n, successes, proportion, lower, upper)

print(prop_table)

# -----------------------------------------------------------
# 3. GEE models
```

```r
# ------------------------------------------------------------
gee_no_pos <- geeglm(
  Chromatic_bin ~ Shape,
  id = Subject,
  data = dat,
  family = binomial(link = "logit"),
  corstr = "exchangeable"
)

gee_with_pos <- geeglm(
  Chromatic_bin ~ Shape + Weibull_position,
  id = Subject,
  data = dat,
  family = binomial(link = "logit"),
  corstr = "exchangeable"
)

summary(gee_no_pos)
summary(gee_with_pos)

# ------------------------------------------------------------
# 4. Helper for Wald confidence intervals
# ------------------------------------------------------------
confint_geeglm <- function(object, level = 0.95) {
  cc <- coef(summary(object))
  mult <- qnorm((1 + level) / 2)
  citab <- cbind(
    lwr = cc[, "Estimate"] - mult * cc[, "Std.err"],
    upr = cc[, "Estimate"] + mult * cc[, "Std.err"]
  )
  rownames(citab) <- rownames(cc)
  citab
}

# Odds ratios
or_no <- exp(cbind(OR = coef(gee_no_pos), confint_geeglm(gee_no_pos)))
or_with <- exp(cbind(OR = coef(gee_with_pos), confint_geeglm(gee_with_pos)))

or_no_shape   <- or_no[grep("Shape", rownames(or_no)), ]
or_with_shape <- or_with[grep("Shape", rownames(or_with)), ]

print(or_no_shape)
print(or_with_shape)

# ------------------------------------------------------------
# 5. Figure S3 – Proportions with Wilson CIs
# ------------------------------------------------------------
prop_plot <- prop_table %>%
```

```r
  mutate(Shape = factor(Shape,
                        levels = c("A", "B", "C1-4", "C5"),
                        labels = c("A Shapes", "B Shapes", "C1-4 Shapes", "C5 Shapes")))

fig_S3 <- ggplot(prop_plot, aes(x = Shape, y = proportion * 100)) +
  geom_hline(yintercept = 50, linetype = "dashed", colour = "#E69F00", linewidth = 0.8) +
  geom_errorbar(aes(ymin = lower * 100, ymax = upper * 100),
                width = 0.12, colour = "#0072B2", linewidth = 0.7) +
  geom_point(size = 3.2, colour = "#0072B2") +
  scale_y_continuous(limits = c(0, 100), breaks = seq(0, 100, 20)) +
  labs(x = NULL, y = "Chromatic reports (%)") +
  theme_classic(base_size = 12) +
  theme(
    axis.text.x = element_text(size = 11),
    panel.grid.major.y = element_line(colour = "grey85", linetype = "dotted")
  ) +
  annotate("text", x = 3.55, y = 53.5,
           label = "Chance level (50%)",
           colour = "#E69F00", size = 3.3, hjust = 0)

print(fig_S3)
ggsave("Figure_S3.pdf", fig_S3, width = 6.5, height = 4.8)
ggsave("Figure_S3.png", fig_S3, width = 6.5, height = 4.8, dpi = 300)

# ------------------------------------------------------------
# 6. Figure S4a – GEE without sequence position
# ------------------------------------------------------------
or_df_no <- data.frame(
  Shape = c("B Shapes", "C1-4 Shapes", "C5 Shapes"),
  OR    = or_no_shape[, "OR"],
  lower = or_no_shape[, "lwr"],
  upper = or_no_shape[, "upr"]
)

fig_S4a <- ggplot(or_df_no, aes(x = Shape, y = OR)) +
  geom_hline(yintercept = 1, linetype = "dashed", colour = "grey50") +
  geom_errorbar(aes(ymin = lower, ymax = upper),
                width = 0.15, colour = "#0072B2", linewidth = 0.8) +
  geom_point(size = 3.5, colour = "#0072B2") +
  scale_y_log10() +
  labs(x = NULL, y = "Odds ratio (relative to A Shapes)",
     title = "Without sequence position") +
  theme_classic(base_size = 13) +
  theme(
    axis.text.x = element_text(size = 11),
    panel.grid.major.y = element_line(colour = "grey90", linetype = "dotted")
  )
```

```
print(fig_S4a)
ggsave("Figure_S4a_without_position.pdf", fig_S4a, width = 6.5, height = 4.8)
ggsave("Figure_S4a_without_position.png", fig_S4a, width = 6.5, height = 4.8, dpi = 300)

# ------------------------------------------------------------
# 7. Figure S4b – GEE with sequence position
# ------------------------------------------------------------
or_df_with <- data.frame(
  Shape = c("B Shapes", "C1-4 Shapes", "C5 Shapes"),
  OR    = or_with_shape[, "OR"],
  lower = or_with_shape[, "lwr"],
  upper = or_with_shape[, "upr"]
)

fig_S4b <- ggplot(or_df_with, aes(x = Shape, y = OR)) +
  geom_hline(yintercept = 1, linetype = "dashed", colour = "grey50") +
  geom_errorbar(aes(ymin = lower, ymax = upper),
              width = 0.15, colour = "#0072B2", linewidth = 0.8) +
  geom_point(size = 3.5, colour = "#0072B2") +
  scale_y_log10() +
  labs(x = NULL, y = "Odds ratio (relative to A Shapes)",
     title = "With sequence position (Weibull)") +
  theme_classic(base_size = 13) +
  theme(
    axis.text.x = element_text(size = 11),
    panel.grid.major.y = element_line(colour = "grey90", linetype = "dotted")
  )

print(fig_S4b)
ggsave("Figure_S4b_with_position.pdf", fig_S4b, width = 6.5, height = 4.8)
ggsave("Figure_S4b_with_position.png", fig_S4b, width = 6.5, height = 4.8, dpi = 300)

message("All figures saved successfully.")


#
===========================================================================
=======
# 3. Code-SupTabS5.py
# Pearson correlation: A-shape opening angle vs subsequent ellipsoidal VFE.
# n = 12 pairs (Supplementary Table S5).
#
===========================================================================
=======

"""
Pearson correlation: A-shape opening angle vs subsequent ellipsoidal VFE.
n = 12 pairs (Supplementary Table S5).
```

```
"""

import numpy as np
from scipy import stats

A_VFE = np.array([0, 0, 0, 29.1, 32.8, 27, 9.7, 19, 27.7, 18.4, 36.5, 39.6])
Subsequent_VFE = np.array([291.3, 312.2, 92.6, 293, 99.7, 164.1, 86.6, 129.5, 256.3, 102,
170.2, 225])

r, p = stats.pearsonr(A_VFE, Subsequent_VFE)
# Fisher z CI
z = np.arctanh(r)
se = 1 / np.sqrt(len(A_VFE) - 3)
lo, hi = np.tanh(z - 1.96 * se), np.tanh(z + 1.96 * se)

print(f"n = {len(A_VFE)}")
print(f"r = {r:.3f}")
print(f"p = {p:.3f}")
print(f"95% CI = [{lo:.3f}, {hi:.3f}]")


#
===========================================================================
=======
# 4. Code-ShapeScales.py
# NDE intensity (scale totals) vs visual-field shape categories.
# Hedges' g = Cohen's d * J; percentile bootstrap 10,000 resamples; seed 42;
# Welch t-tests; Holm correction across eight comparisons.
#
===========================================================================
=======

"""
NDE intensity (scale totals) vs visual-field shape categories.

Specification (must match Methods / Results):
  - Hedges' g = Cohen's d * J, with J = 1 - 3/(4*df - 1)
  - Percentile bootstrap 95% CIs
  - 10,000 resamples
  - Independent resampling within each group
  - Random seed = 42
  - Welch t-tests (unequal variance)
  - Holm correction across the eight comparisons
"""

import numpy as np
from scipy import stats
from statsmodels.stats.multitest import multipletests
```

```
# Subject, A, B, C1-4, C5, NDE-C total, Greyson total
data = np.array([
    [1, 1, 1, 0, 1, 72, 23],
    [2, 0, 0, 0, 0, 43, 13],
    [3, 1, 1, 0, 0, 58, 19],
    [4, 1, 1, 0, 0, 70, 24],
    [5, 1, 0, 0, 1, 54, 22],
    [6, 1, 1, 1, 1, 76, 28],
    [7, 1, 0, 1, 0, 31, 16],
    [8, 1, 0, 0, 0, 61, 21],
    [9, 1, 0, 0, 0, 46, 12],
    [10, 1, 1, 0, 1, 54, 23],
    [11, 1, 1, 1, 1, 68, 28],
    [12, 1, 1, 1, 0, 67, 23],
    [13, 1, 1, 0, 0, 60, 22],
    [14, 1, 0, 0, 1, 74, 25],
    [15, 1, 1, 0, 0, 41, 15],
    [16, 1, 0, 0, 0, 44, 20],
    [17, 1, 1, 0, 1, 68, 26],
    [18, 1, 1, 0, 1, 59, 15],
    [19, 1, 0, 0, 0, 72, 26],
    [20, 1, 1, 1, 1, 73, 23],
    [21, 1, 1, 1, 1, 67, 29],
    [22, 1, 0, 0, 1, 48, 20],
    [23, 1, 0, 0, 1, 40, 13],
    [24, 0, 1, 0, 0, 72, 25],
    [25, 1, 1, 0, 1, 52, 9],
    [26, 0, 1, 0, 0, 47, 22],
    [27, 0, 1, 0, 0, 55, 24],
    [28, 0, 1, 1, 0, 49, 19],
    [29, 0, 1, 0, 0, 31, 11],
    [30, 0, 0, 1, 0, 50, 17],
    [31, 0, 0, 1, 0, 53, 17],
    [32, 1, 0, 1, 0, 57, 26],
    [33, 0, 0, 1, 0, 76, 20],
    [34, 0, 0, 1, 0, 31, 14],
    [35, 0, 0, 1, 0, 57, 23],
    [36, 0, 0, 1, 0, 46, 14],
    [37, 0, 0, 1, 0, 31, 11],
    [38, 0, 0, 1, 0, 41, 11],
    [39, 0, 0, 0, 1, 53, 18],
    [40, 0, 0, 0, 1, 48, 21],
    [41, 0, 0, 1, 1, 33, 20],
    [42, 1, 0, 1, 1, 67, 23],
    [43, 0, 0, 0, 1, 61, 22],
    [44, 0, 0, 0, 1, 70, 20],
    [45, 0, 0, 0, 1, 51, 14],
```

```
    [46, 0, 0, 0, 1, 66, 26],
    [47, 0, 1, 0, 1, 55, 14],
    [48, 0, 0, 0, 1, 50, 17],
    [49, 0, 0, 0, 0, 38, 7],
    [50, 0, 0, 0, 0, 70, 25],
])

A = data[:, 1]
B = data[:, 2]
C14 = data[:, 3]
C5 = data[:, 4]
ndec = data[:, 5].astype(float)
greyson = data[:, 6].astype(float)

N_BOOT = 10000
SEED = 42


def hedges_g(x, y):
    """Hedges' g = Cohen's d * J, J = 1 - 3/(4*df - 1)."""
    nx, ny = len(x), len(y)
    pooled = np.sqrt(
        ((nx - 1) * x.std(ddof=1) ** 2 + (ny - 1) * y.std(ddof=1) ** 2)
        / (nx + ny - 2)
    )
    d = (x.mean() - y.mean()) / pooled
    J = 1.0 - 3.0 / (4.0 * (nx + ny - 2) - 1.0)
    return d * J


def bootstrap_g_ci(x, y, n_boot, rng):
    """Percentile bootstrap CI; independent resampling within each group."""
    g_obs = hedges_g(x, y)
    boots = np.empty(n_boot)
    for i in range(n_boot):
        xb = rng.choice(x, size=len(x), replace=True)
        yb = rng.choice(y, size=len(y), replace=True)
        boots[i] = hedges_g(xb, yb)
    lo, hi = np.quantile(boots, [0.025, 0.975])
    return g_obs, lo, hi


def main():
    print(
        f"Specification: Hedges' g; percentile bootstrap; "
        f"n_boot={N_BOOT}; seed={SEED}\n"
    )
    print(f"NDE-C overall:   M={ndec.mean():.2f}, SD={ndec.std(ddof=1):.2f}")
```

```
print(f"Greyson overall: M={greyson.mean():.2f}, SD={greyson.std(ddof=1):.2f}\n")

order = [
    ("Greyson", greyson, "A", A),
    ("NDE-C", ndec, "A", A),
    ("Greyson", greyson, "B", B),
    ("NDE-C", ndec, "B", B),
    ("Greyson", greyson, "C1-4", C14),
    ("NDE-C", ndec, "C1-4", C14),
    ("Greyson", greyson, "C5", C5),
    ("NDE-C", ndec, "C5", C5),
]

rng = np.random.default_rng(SEED)
rows = []
pvals = []

for score_name, score, space_name, ind in order:
    yes = score[ind == 1]
    no = score[ind == 0]
    _, p = stats.ttest_ind(yes, no, equal_var=False)
    g, lo, hi = bootstrap_g_ci(yes, no, N_BOOT, rng)
    rows.append({
        "score": score_name,
        "space": space_name,
        "n_yes": len(yes),
        "n_no": len(no),
        "mean_yes": yes.mean(),
        "sd_yes": yes.std(ddof=1),
        "mean_no": no.mean(),
        "sd_no": no.std(ddof=1),
        "g": g,
        "lo": lo,
        "hi": hi,
        "p": p,
    })
    pvals.append(p)

_, p_holm, _, _ = multipletests(pvals, method="holm")

print(
    f"{'score':7} {'space':5} {'n':7} {'M_yes':7} {'M_no':7} "
    f"{'g':7} {'CI_lo':7} {'CI_hi':7} {'p':8} {'p_Holm':8}"
)
for r, ph in zip(rows, p_holm):
    print(
        f"{r['score']:7} {r['space']:5} "
        f"{r['n_yes']:2}/{r['n_no']:<4} "
```

```python
            f"{r['mean_yes']:7.1f} {r['mean_no']:7.1f} "
            f"{r['g']:7.3f} {r['lo']:7.3f} {r['hi']:7.3f} "
            f"{r['p']:8.3f} {ph:8.3f}"
        )

    print("\n--- LaTeX-ready (3 decimals) ---")
    for r in rows:
        print(
            f"{r['score']} {r['space']}: "
            f"g = {r['g']:.3f}, 95% CI [{r['lo']:.3f}, {r['hi']:.3f}], "
            f"p = {r['p']:.3f}"
        )


if __name__ == "__main__":
    main()
```
